# Identification and validation of QTLs for kernel number per spike and spike length in two founder genotypes of wheat

**DOI:** 10.1186/s12870-022-03544-6

**Published:** 2022-03-26

**Authors:** Xin Xu, Xiaojun Li, Dehua Zhang, Jishun Zhao, Xiaoling Jiang, Haili Sun, Zhengang Ru

**Affiliations:** 1 School of Life Sciences and Basic Medicine, Xinxiang University, Xinxiang, 453003 China; 2 School of Life Science and Technology, Collaborative Innovation Center of Modern Biological Breeding, Henan Province, Henan Institute of Science and Technology, Xinxiang, 453003 China

**Keywords:** Kernel number per spike, Spike length, QTL, KASP markers

## Abstract

**Background:**

Kernel number per spike (KNS) and spike length (SL) are important spike-related traits in wheat variety improvement. Discovering genetic loci controlling these traits is necessary to elucidate the genetic basis of wheat yield traits and is very important for marker-assisted selection breeding.

**Results:**

In the present study, we used a recombinant inbred line population with 248 lines derived from the two founder genotypes of wheat, Bima4 and BainongAK58, to construct a high-density genetic map using wheat 55 K genotyping assay. The final genetic linkage map consists of 2356 bin markers (14,812 SNPs) representing all 21 wheat chromosomes, and the entire map spanned 4141.24 cM. A total of 7 and 18 QTLs were identified for KNS and SL, respectively, and they were distributed on 11 chromosomes. The allele effects of the flanking markers for 12 stable QTLs, including four QTLs for KNS and eight QTLs for SL, were estimated based on phenotyping data collected from 15 environments in a diverse wheat panel including 384 elite cultivars and breeding lines. The positive alleles at seven loci, namely, *QKns.his-7D2–1*, *QKns.his-7D2–2*, *QSl.his-4A-1*, *QSl.his-5D1*, *QSl.his-4D2–2*, *QSl.his-5B* and *QSl.his-5A-2*, significantly increased KNS or SL in the diverse panel, suggesting they are more universal in their effects and are valuable for gene pyramiding in breeding programs. The transmission of Bima4 allele indicated that the favorite alleles at five loci (*QKns.his-7D2–1*, *QSl.his-5A-2*, *QSl.his-2D1–1*, *QSl.his-3A-2* and Q*Sl.his-3B*) showed a relatively high frequency or an upward trend following the continuity of generations, suggesting that they underwent rigorous selection during breeding. At two loci (*QKns.his-7D2–1* and *QSl.his-5A-2*) that the positive effects of the Bima4 alleles have been validated in the diverse panel, two and one kompetitive allele-specific PCR (KASP) markers were further developed, respectively, and they are valuable for marker-assisted selection breeding.

**Conclusion:**

Important chromosome regions controlling KNS and SL were identified in the founder parents. Our results are useful for knowing the molecular mechanisms of founder parents and future molecular breeding in wheat.

**Supplementary Information:**

The online version contains supplementary material available at 10.1186/s12870-022-03544-6.

## Background

Wheat (*Triticum aestivum* L.) is a major cereal crop worldwide. The current yield trend in wheat is insufficient to meet the future demand of a growing world population, and wheat yield and total production must be increased continuously. The formation of wheat yield is a complex trait and is affected by three yield components, e.g., spike number per unit area, kernel weight, and kernel number per spike (KNS). Among them, kernel weight and KNS are closely related to spike morphology, which is primarily determined by spike length (SL), spikelet density, and fertile floret number. Previous studies showed that increasing KNS is an effective approach for wheat yield improvements compared to kernel weight [1, 2], and increasing SL without modification of the spikelet density can increase KNS and subsequently raise yield capacity [3]. A positive correlation between SL and yield was also validated in some previous studies [4]. Besides, the long spike is often associated with reduced severity of Fusarium head blight in wheat [5]. Therefore, discovering genetic loci controlling KNS and SL is necessary to elucidate the genetic basis of wheat yield traits and is very important for marker-assisted selection (MAS) breeding.

Like other spike-related traits, KNS and SL are controlled by multiple genes and affected by environments. Quantitative trait loci (QTL) analysis using different genetic populations and diverse wheat panels provides an effective method to study the genes governing these traits. To date, numerous QTLs associated with KNS have been mapped on nearly all the 21 chromosomes in wheat, such as two QTLs identified on 2D and 4A [6], eight QTLs identified on 1A, 1B, 2B, 2D, 3B, 4B, 6A and 7B [7], one QTL identified on 3D [8], one QTL identified on 4A [9], four QTLs identified on 2A, 4B and 7A [10], six QTLs identified on 1D, 2A, 2D, 3A, 4D and 6D [11], three QTLs identified on 1D, 4D and 6B [12], 12 QTLs identified on 1A, 2D, 3B, 4A, 4B, 5A, 5B, 7A and 7B [13], and one QTL identified on 7A [14]. Likewise, many previous studies have proven that almost all the 21 wheat chromosomes harbored factors affecting SL [3, 10, 15–21]. Yao et al. [22] reported that approximately 350 QTLs of SL have been identified currently, and some of them with relatively large effects were distributed on chromosomes 2D, 3A, 4A, 4B, 5A, 6A, 6B, 7A, 7B, and 7D. Briefly, because of the complexity of the wheat genome, although many QTL for KNS and SL have been reported, common QTLs across different mapping populations are limited, and few of them are used in practical wheat breeding.

Founder parents have played particularly crucial roles in the improvement of wheat worldwide. Many QTLs or chromosomal regions associated with important traits have been found in founder genotypes in wheat [23–26]. However, the knowledge of the molecular mechanisms for the formation of founder parents remains unclear. In China, Bima4 is one of the founder parents that played important roles in wheat breeding, used widely in the Yellow and Huai River Facultative Winter Wheat Region between 1950 and 1970 [27]. It was obtained from the cross between another founder parent Mazhamai and Quality from the United States. More than 70 improved cultivars were developed from Bima4, and some of them such as Shijiazhuang54, Jinan2, Beijing8 and Taishan1, had annual maximum acreages over 667,000 ha and were grown for at least 12 years. Similarly, BainongAK58 is a famous cultivar released in 2003 by the Henan Institute of Science and Technology, and its maximum acreage was over 13,333,333 ha. It was also widely utilized as a crossing parent in wheat breeding, from which more than fifty improved cultivars were developed.

In the present study, we used a recombinant inbred line (RIL) population with 248 lines derived from the two founder genotypes of wheat, Bima4 and BainongAK58. The QTL analysis was conducted with a high-density genetic map by using the developed wheat 55 K genotyping assay to identify QTLs responsible for KNS and SL. These QTLs detected were further validated in a diverse wheat panel. Furthermore, we analyzed the transmission of Bima4 alleles to its derivative descendants, and two and one KASP markers for two important loci, *QKns.his-7D2–1* and *QSl.his-5A-2*, were developed. This study is useful for knowing the molecular mechanisms of founder parents and future molecular breeding in wheat.

## Results

### Linkage map construction in the RIL population

Out of 53,063 SNPs in the 55 k Infinium chip, 16,628 SNPs were polymorphic between the two parents and among the RIL population. These 16,628 markers were divided into 2488 bins. Only one marker was chosen to represent each bin for the genetic map construction. The final genetic linkage map consists of 2356 bin markers (14,812 SNPs) representing all 21 wheat chromosomes. Of them, 1147 bins include only one SNP marker, and the remaining comprises two or more SNP markers.

The 2356 bin markers were mapped on 28 linkage maps (Table 1 and Table S1). Each of the chromosomes 1A, 1D, 2D, 3D, 4D, 5D, and 7D was integrated by two linkage groups. The entire map spanned 4141.24 cM with six gaps (> 30 cM) distributed on chromosomes 2D, 6A, 4D, 5B, and 7D. The mean of genetic distance among adjacent bin markers across all chromosomes was 1.76 cM and varied among 28 linkage groups from 0.71 (1A2) to 5.98 (7D1). The bin markers mapped on the A genome (37.9%) were more than those on the B (34.2%) and D (28.0%) genome. Similarly, most of the mapped markers including bin and redundant markers were distributed on A (43.0%) and B genome (36.9%), and only 20.0% of the markers were mapped on D genome. The number of bin markers on 21 chromosomes ranged from 51 on 1D to 177 on 7D, however the number of the mapped markers ranged from 168 on 4D to 1479 on 2A.

**Table 1:** Distribution of markers in the genetic map developed using the RIL population derived from BainongAK58 × Bima4

Chromosome	Group	Number of bin marker	Number of mapped marker	Length (cM)	cM per bin marker
1A	1	8	18	24.69	3.09
	2	124	920	88.63	0.71
1B	1	73	370	88.96	1.22
1D	1	42	261	137.95	3.28
	2	9	41	44.36	4.93
2A	1	102	1479	193.72	1.90
2B	1	138	765	169.44	1.23
2D	1	90	402	269.98	3.00
	2	13	34	61.04	4.70
3A	1	164	994	223.75	1.36
3B	1	119	1218	159.76	1.34
3D	1	10	28	76.02	7.60
	2	81	640	195.84	2.42
4A	1	104	772	171.62	1.65
4B	1	108	1228	140.04	1.30
4D	1	5	10	14.64	2.93
	2	54	158	152.69	2.83
5A	1	165	787	194.44	1.18
5B	1	160	921	209.27	1.31
5D	1	70	217	268.53	3.84
	2	17	90	87.20	5.13
6A	1	55	443	131.77	2.40
6B	1	103	585	133.25	1.29
6D	1	91	232	192.00	2.11
7A	1	170	961	226.19	1.33
7B	1	104	385	155.31	1.49
7D	1	13	61	77.72	5.98
	2	164	792	252.46	1.54
Total	28	2356	14,812	4141.24	1.76

### Phenotypic analysis for KNS and SL in the RIL population

**Fig. 1:**
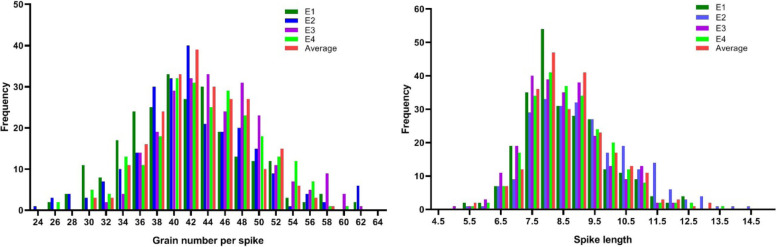
Frequency distributions of the KNS and SL means in the RIL population

**Fig. 2:**
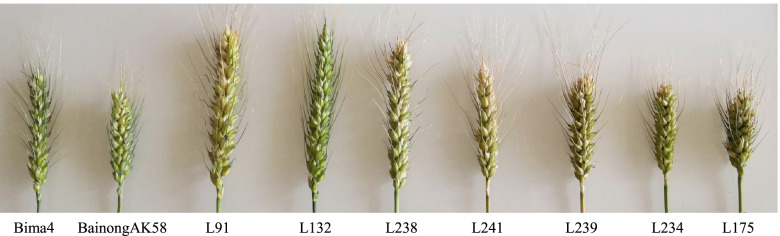
Phenotypes of the two parents (Bima4 and BainongAK58) and partial RILs

These two traits for the RIL populations and the two parents in the four environments are shown in Table S2. The SL and KNS showed inconsistency between the parental lines over environments, indicating strongly affected by the environment. In the RIL populations, the KNS and SL showed normal distributions in all the environments, suggesting the polygenic inheritance of these traits (Fig. 1). The transgressive inheritance was found in certain lines for SL and KNS (Fig. 2). The two traits showed strong correlations with each other in all environments. The correlation coefficients ranged from 0.86 to 0.96 for SL and from 0.50 to 0.86 for KNS. The SL had a strong positive correlation with KNS at 0.24 (*P* < 0.0001) (Table 2). The SL and KNS showed high broad-sense heritability at 0.95 and 0.85, respectively.

**Table 2:** Correlations among different environments for SL and KNS

Trait	2017XI	2018XI	2018HU	2019XI	Average
SL					
2017XI	1.00				
2018XI	0.86^a^	1.00			
2019HU	0.88^a^	0.88^a^	1.00		
2019XI	0.87^a^	0.92^a^	0.89^a^	1.00	
Average	0.94^a^	0.95^a^	0.96^a^	0.96^a^	1.00
KNS					
2017XI	1.00				
2018XI	0.61^a^	1.00			
2019HU	0.50^a^	0.55^a^	1.00		
2019XI	0.63^a^	0.68^a^	0.56^a^	1.00	
Average	0.83^a^	0.84^a^	0.80^a^	0.86^a^	1.00
	Average-SL				
Average-KNS	0.24^a^				

^a^Significance level at 0.0001

### QTL detection for KNS and SL in the RIL population

**Fig. 3:**
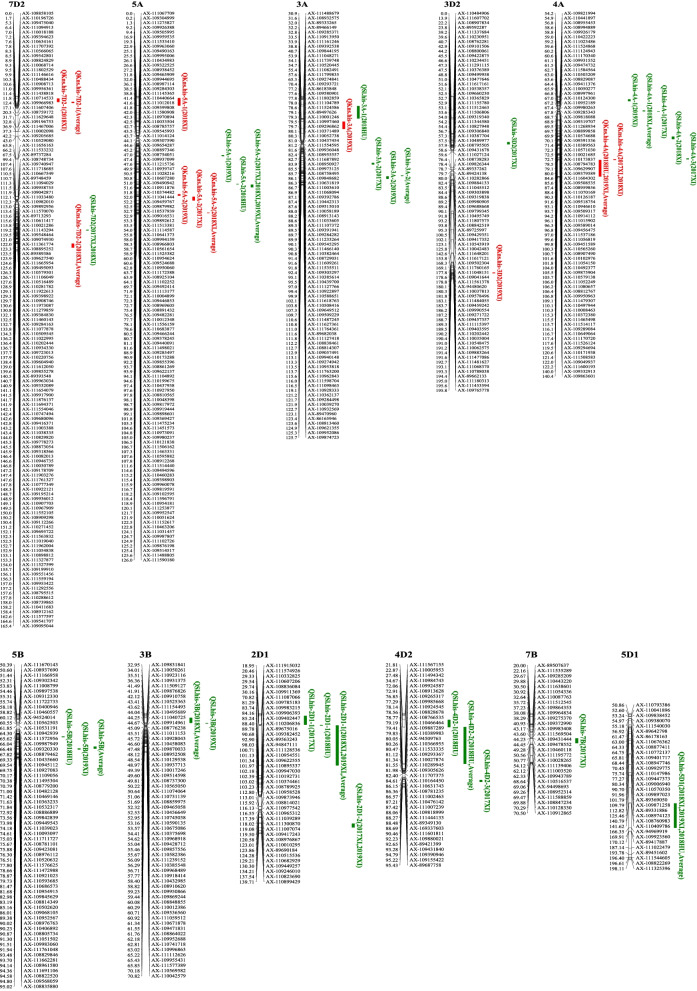
The genetic map of 11 linkage groups and QTL analysis for KNS (red) and SL (green) in the RIL population

A total of seven QTLs were detected for KNS on chromosomes 3A, 3D, 4A, 5A, and 7D (Table 3 and Fig. 3). A major stable QTL, *Qkns.his-4A*, was detected in all four environments and the average value and explained 9.78–24.24% of the phenotypic variance. *Qkns.his-5A-2* was identified in three environments and the average value and explained 3.72–7.01% of phenotypic variation. The positive alleles of *Qkns.his-4A* and *Qkns.his-5A-2* were contributed by Bima4. The two QTLs, *Qkns.his-7D-1* and *Qkns.his-7D-2*, were detected in one environment and the average value, and the positive alleles were contributed by Bima4 and BainongAK58, respectively. The remaining three QTLs, *Qkns.his-3A*, *Qkns.his-3D* and *Qkns.his-5A-1*, were detected in a single environment, and they explain 5.29, 4.22, and 4.01% of the phenotypic variance, respectively.

**Table 3:** Significant QTLs for SL and KNS identified from different environments

Trait	QTL	Environment	Location	Left marker	Right marker	LOD	PVE (%)	Add
KNS	*QKns.his-3A*	2019XI	58	AX-111799835	AX-109274841	3.98	5.29	−1.46
	*QKns.his-3D2*	2019XI	190	AX-109403595	AX-110202442	3.26	4.22	−1.30
	*QKns.his-4A*	Average	123	AX-111600193	AX-109332913	18.16	24.24	−2.80
		2018HU	123	AX-111600193	AX-109332913	5.29	9.78	−2.28
		2018XI	122	AX-111508583	AX-109049937	15.47	17.97	−2.66
		2017XI	122	AX-111508583	AX-109049937	14.35	19.32	−3.17
		2019XI	123	AX-111600193	AX-109332913	12.09	17.68	−2.76
	*QKns.his-5A-1*	2018XI	2	AX-111275827	AX-109926388	3.71	4.01	1.20
	*QKns.his-5A-2*	2019XI	106	AX-109980237	AX-110121838	2.82	3.72	−1.24
		2017XI	111	AX-108912268	AX-111514440	5.40	7.01	−1.86
		Average	125	AX-111102726	AX-109876198	4.67	5.22	−1.25
		2018XI	125	AX-111102726	AX-109876198	6.10	6.51	−1.53
	*QKns.his-7D2–1*	2018XI	3	AX-110196726	AX-109475040	4.72	5.39	−1.40
		Average	6	AX-109475040	AX-111094913	3.38	3.82	−1.07
	*QKns.his-7D2–2*	Average	159	AX-108912162	AX-111577597	5.28	6.11	1.35
		2018XI	159	AX-108912162	AX-111577597	3.45	3.70	1.16
SL	*QSl.his-2D1–1*	2017XI	29	AX-111574926	AX-110332825	11.04	11.03	−0.43
		2018HU	30	AX-108836084	AX-109911369	11.07	11.12	−0.55
		2018XI	37	AX-109911369	AX-111087066	13.95	18.65	−0.62
		2019XI	37	AX-109911369	AX-111087066	19.10	22.31	−0.70
		Average	37	AX-109911369	AX-111087066	15.46	18.73	−0.65
	*QSl.his-2D1–2*	2017XI	131	AX-109449257	AX-109246010	4.13	3.89	0.25
		2019XI	134	AX-109449257	AX-109246010	2.94	2.04	0.21
	*QSl.his-3A-1*	2018HU	53	AX-109844195	AX-111048271	3.88	3.86	−0.32
	*QSl.his-3A-2*	2017XI	103	AX-111618763	AX-110508416	3.90	3.65	−0.25
		2019XI	117	AX-111098463	AX-110928333	3.92	2.82	−0.25
		Average	116	AX-110962843	AX-111598704	3.09	2.18	−0.22
	*QSl.his-3B*	2018XI	41	AX-110931375	AX-111509127	3.44	2.78	−0.24
		2019XI	42	AX-109876826	AX-109910758	4.42	3.13	−0.26
		Average	41	AX-110931375	AX-111509127	3.71	2.77	−0.25
	*QSl.his-3D2*	2017XI	49	AX-109499958	AX-110477646	3.97	3.66	−0.25
	*QSl.his-4A-1*	2019XI	58	AX-108955453	AX-108994889	4.82	3.43	0.28
		2018XI	69	AX-109319707	AX-111268934	9.11	7.69	0.41
		Average	69	AX-109319707	AX-111268934	5.55	4.08	0.31
		2017XI	70	AX-110574688	AX-109391536	6.07	5.77	0.31
	*QSl.his-4A-2*	2018XI	99	AX-111537186	AX-111056819	3.35	2.70	−0.24
	*QSl.his-4A-3*	2017XI	121	AX-110171938	AX-111508583	4.85	4.53	−0.29
	*QSl.his-4D2–1*	2018HU	28	AX-111494342	AX-110984743	2.93	2.83	0.28
	*QSl.his-4D2–2*	Average	57	AX-110984743	AX-109924587	5.02	10.60	0.51
		2018HU	65	AX-110984743	AX-109924587	5.95	10.33	0.55
	*QSl.his-4D2–3*	2017XI	89	AX-169337603	AX-111601811	3.95	3.69	0.25
	*QSl.his-5A-1*	2019XI	63	AX-110950060	AX-111172588	3.58	2.51	0.23
	*QSl.his-5A-2*	2018HU	94	AX-109622137	AX-111104892	5.08	4.84	−0.37
		2018XI	95	AX-110199675	AX-110437938	5.68	4.65	−0.32
		2017XI	96	AX-110199675	AX-110437938	8.46	8.38	−0.38
		2019XI	96	AX-110199675	AX-110437938	9.63	7.24	−0.41
		Average	96	AX-110199675	AX-110437938	6.82	5.16	− 0.35
	*QSl.his-5B*	2018HU	73	AX-108886889	AX-109842839	4.59	4.34	0.34
		2019XI	86	AX-110502620	AX-109068105	3.01	2.06	0.21
		Average	85	AX-108814349	AX-110502620	3.39	2.41	0.23
	*QSl.his-5D1*	2018HU	151	AX-110409786	AX-94969919	5.11	8.21	0.47
		2019XI	157	AX-110409786	AX-94969919	3.29	3.97	0.29
		2018XI	159	AX-110409786	AX-94969919	3.75	4.75	0.31
		Average	156	AX-110409786	AX-94969919	4.11	5.06	0.34
	*QSl.his-7B*	2017XI	49	AX-109478552	AX-110460118	2.94	2.71	−0.21
	*QSl.his-7D2*	2018XI	144	AX-111038335	AX-110829820	3.42	2.68	0.24
		2017XI	144	AX-111038335	AX-110829820	2.82	2.55	0.21

A total of 18 QTLs were detected for SL on chromosomes 2D, 3A, 3B, 3D, 4A, 4D, 5A, 5B, 5D, 7B, and 7D with phenotypic variations ranging from 2.04 to 22.31% (Table 3 and Fig. 3). Among them, eight QTLs were detected in a single environment, explaining 2.93–4.85% of the phenotypic variance. Two stable QTLs, *Qsl.his-2D1–1* and *Qsl.his-5A-2*, were detected in all four environments and the average values, and the positive alleles were from Bima4. Of these, the major QTL, *Qsl.his-2D1–1*, explained 11.03–22.31% of the phenotypic variance. Two QTLs, *Qsl.his-4A-1* and *Qsl.his-5D1*, were identified in three environments and the average values. They accounted for 3.43–8.21% of the phenotypic variance and the positive alleles were contributed by BainongAK58. The two QTLs, *Qsl.his-3A-2* and *Qsl.his-3B*, were detected in three environments and the average values, and they explained 2.18–3.65% of phenotypic variation and the positive alleles were from Bima4.

### Validation of the QTL effects in the diverse wheat panel

The allele effects of the flanking markers for 12 stable QTLs, including four QTLs for KNS and eight QTLs for SL, were estimated based on phenotyping data in the diverse wheat panel. T-test analyses were used to compare the two different allele groups in the same locus (*P* < 0.05). For each QTL investigated, the QTL-associated SNP markers for which differences of phenotypic values showed significance in most environments in the diverse wheat panel were analyzed (Table 4). For KNS, three QTL-associated SNP markers of *QKns.his-7D2–1* were analyzed. The marker AX-110196726 showed significance in 14 environments in the diverse wheat panel, while the other two markers (AX-109475040 and AX-111094913) showed a significant difference of KNS only in six environments, indicating that the former was closer to this QTL compared to the latter. The positive allele of AX-110196726 contributed by Bima4 had a more KNS than BainongAK58 allele in the diverse wheat panel. Similarly, two QTL-associated SNP markers (AX-108912162 and AX-111577597) of *QKns.his-7D2–2* were analyzed. The positive alleles contributed by BainongAK58 showed significant across all 15 environments more KNS at 5.17 and 4.85 than the Bima4 alleles at these two loci in the diverse wheat panel, respectively. In addition, among six QTL-associated SNP markers of *QKns.his-5A-2* where the favourable alleles were from Bima4, four showed significant differences of KNS in 15 environments, respectively. At the same time, the effects on KNS were inverse among them, i.e., the Bima4 alleles showed negative effects at AX-109980237 and AX-110121838, but positive effects at AX-111102726 and AX-109876198. In the same way, of four QTL-associated SNP markers of *QKns.his-4A*, two markers (AX-111508583 and AX-109332913) showed significant differences of KNS only in 6 environments respectively. At the same time, the effects on KNS were inverse between them.

**Table 4:** Effects of the QTL-associated SNP markers in the diverse wheat panel

QTL name	Marker	Position	Allele (origin)	Sample size	2007 SX	2007 JS	2007 HB	2007 SD	2007 SC	2008 SX	2008 JS	2008 HB	2008 SD	2008 SC	2009 SX	2009 JS	2009 HB	2009 SD	2009 SC
*Qkns.his-7D2–1*	AX-110196726	4,390,879	C/C (M^a^)	347	52.185	52.391	57.197	48.319	46.821	51.844	52.094	50.900	41.278	33.542	51.789	51.159	53.268	42.665	35.141
			T/T (N)	22	46.886	45.901	51.568	44.214	40.143	45.905	45.196	44.854	37.971	28.322	46.664	44.824	50.193	37.970	30.949
				*P* value	0.000	0.000	0.011	0.012	0.013	0.000	0.000	0.002	0.001	0.000	0.001	0.001	0.056	0.000	0.005
*Qkns.his-7D2–2*	AX-108912162	526,362,487	G/G (M)	170	49.392	49.051	53.359	45.375	43.675	48.710	49.125	47.614	39.999	30.759	48.493	47.112	50.408	40.163	32.548
			T/T (N^a^)	187	54.036	54.804	60.503	50.546	48.860	54.015	54.224	53.326	42.148	35.288	54.022	54.245	55.895	44.492	36.932
				*P* value	0.000	0.000	0.000	0.000	0.000	0.000	0.000	0.000	0.000	0.000	0.000	0.000	0.000	0.000	0.000
	AX-111577597	530,751,616	C/C (M)	188	49.849	49.422	54.022	45.448	43.797	49.167	49.029	47.573	39.946	30.792	48.920	47.310	50.889	40.772	32.924
			T/T (N^a^)	169	53.776	54.794	60.135	50.660	49.067	53.811	54.571	53.355	42.333	35.309	54.040	54.441	55.505	44.047	36.804
				*P* value	0.000	0.000	0.000	0.000	0.000	0.000	0.000	0.000	0.000	0.000	0.000	0.000	0.000	0.000	0.000
*Qkns.his-5A-2*	AX-109980237	548,422,922	223 (M^a^)	C/C	50.326	49.932	54.381	46.336	44.857	49.888	49.646	48.214	39.950	31.241	49.949	47.895	51.573	40.688	33.411
			136 (N)	G/G	54.087	55.135	60.917	50.949	48.473	53.846	54.736	53.752	42.753	35.923	53.544	54.910	55.529	44.941	36.676
				*P* value	0.000	0.000	0.000	0.000	0.006	0.000	0.000	0.000	0.000	0.000	0.000	0.000	0.000	0.000	0.000
	AX-110121838	549,336,395	191 (M^a^)	A/A	50.857	50.409	54.539	46.159	44.870	50.307	49.808	48.815	40.202	31.528	50.410	48.584	51.558	40.976	33.728
			169 (N)	G/G	52.883	54.064	59.530	50.411	47.616	52.691	53.879	52.474	42.212	35.045	52.444	53.088	54.619	43.860	36.067
				*P* value	0.003	0.000	0.000	0.000	0.024	0.000	0.000	0.000	0.000	0.000	0.004	0.000	0.001	0.000	0.001
	AX-111102726	572,237,027	C/C (M^a^)	321	52.472	52.902	58.148	49.000	47.310	52.064	52.783	51.535	41.388	33.705	52.128	51.760	54.229	42.755	35.367
			T/T (N)	51	48.441	47.278	50.600	42.918	41.280	47.889	45.638	44.843	38.883	29.943	47.629	45.035	47.602	40.180	31.614
				*P* value	0.000	0.000	0.000	0.000	0.001	0.000	0.000	0.000	0.000	0.000	0.000	0.000	0.000	0.002	0.000
	AX-109876198	572,814,730	C/C (M^a^)	319	52.356	52.808	57.938	48.957	47.182	51.947	52.707	51.497	41.420	33.606	52.007	51.693	54.175	42.690	35.307
			A/A (N)	52	48.281	47.134	50.451	42.701	41.549	47.678	45.513	44.037	38.792	29.625	47.344	45.060	47.470	40.196	31.472
				*P* value	0.000	0.000	0.000	0.000	0.002	0.000	0.000	0.000	0.000	0.000	0.000	0.000	0.000	0.002	0.000
*Qkns.his-4A*	AX-109332913	701,527,878	A/A (M^a^)	308	51.574	51.725	56.439	47.916	46.131	51.055	51.173	50.160	41.064	32.767	51.066	50.588	53.144	42.102	34.554
			G/G (N)	61	53.277	53.520	58.883	49.213	48.633	53.648	54.304	51.875	41.384	34.781	53.028	51.949	53.689	43.799	36.126
				*P* value	0.037	0.068	0.060	0.133	0.090	0.001	0.002	0.091	0.328	0.010	0.028	0.139	0.332	0.018	0.067
	AX-111508583	684,899,178	C/C (M^a^)	162	51.999	52.155	58.099	48.859	46.272	52.193	52.416	51.069	40.895	33.222	52.154	50.831	54.176	42.496	35.388
			A/A (N)	186	51.501	51.367	55.978	47.298	46.124	50.748	50.831	49.772	41.073	33.099	50.610	50.706	52.342	42.305	34.286
				*P* value	0.249	0.193	0.039	0.039	0.459	0.017	0.030	0.099	0.373	0.429	0.024	0.448	0.027	0.379	0.083
*Qsl.his-4A-1*	AX-108955453	36,102,562	G/G (M)	71	8.514	9.314	9.246	9.510	8.228	8.213	9.538	8.994	9.276	7.425	8.710	9.404	8.910	8.407	8.031
			A/A (N^a^)	299	9.300	10.121	9.471	10.173	8.855	8.796	10.157	9.210	9.544	7.977	9.226	10.125	9.126	8.943	8.457
				*P* value	0.000	0.000	0.167	0.000	0.001	0.001	0.001	0.202	0.017	0.000	0.012	0.000	0.162	0.001	0.000
*Qsl.his-5D1*	AX-110409786	446,797,256	C/C (M)	229	8.907	9.833	9.388	9.852	8.611	8.544	9.891	9.150	9.388	7.709	8.942	9.803	8.996	8.728	8.287
			A/A (N^a^)	134	9.572	10.152	9.541	10.328	8.925	8.909	10.250	9.202	9.651	8.118	9.403	10.270	9.246	8.988	8.495
				*P* value	0.000	0.018	0.211	0.002	0.029	0.009	0.014	0.404	0.006	0.001	0.007	0.002	0.082	0.037	0.024
*Qsl.his-4D2–2*	AX-109924587	168,781,777	A/A (M)	116	8.752	9.699	9.135	9.559	8.317	8.458	9.726	8.869	9.310	7.524	8.671	9.539	8.788	8.534	8.187
			G/G (N^a^)	245	9.304	10.055	9.545	10.240	8.889	8.772	10.145	9.273	9.572	8.021	9.314	10.174	9.204	8.965	8.469
				*P* value	0.001	0.012	0.019	0.000	0.001	0.025	0.007	0.030	0.008	0.000	0.000	0.000	0.012	0.002	0.004
*Qsl.his-5B*	AX-108886889	490,878,581	C/C (M)	94	8.731	9.557	9.378	9.636	8.384	8.538	9.718	9.195	9.293	7.456	8.951	9.688	9.108	8.617	8.197
			T/T (N^a^)	265	9.285	10.103	9.427	10.160	8.867	8.728	10.142	9.158	9.564	8.011	9.151	10.093	9.077	8.903	8.453
				*P* value	0.001	0.001	0.406	0.002	0.003	0.132	0.010	0.435	0.009	0.000	0.165	0.011	0.437	0.038	0.013
*Qsl.his-5A-2*	AX-109622137	506,645,437	198 (M^a^)	T/T	9.241	10.152	9.689	10.082	8.927	8.823	10.221	9.466	9.578	7.942	9.236	10.156	9.307	8.981	8.353
			175 (N)	G/G	8.961	9.653	9.074	9.895	8.501	8.451	9.736	8.765	9.344	7.711	8.941	9.723	8.791	8.619	8.371
				*P* value	0.033	0.000	0.000	0.107	0.004	0.003	0.001	0.000	0.007	0.023	0.046	0.002	0.001	0.004	0.428
	AX-110199675	511,102,792	C/C (M^a^)	142	9.346	10.339	9.829	10.254	9.032	8.949	10.355	9.619	9.645	8.065	9.376	10.321	9.454	9.134	8.443
			T/T (N)	213	8.992	9.695	9.173	9.866	8.546	8.510	9.786	8.894	9.378	7.742	8.959	9.753	8.846	8.649	8.349
				*P* value	0.016	0.000	0.000	0.009	0.002	0.002	0.000	0.000	0.006	0.006	0.014	0.000	0.000	0.000	0.184
*QSl.his-3A-2*	AX-111618763	599,422,098	T/T (M^a^)	227	8.965	9.778	9.329	9.896	8.630	8.601	9.882	9.140	9.406	7.711	9.000	9.833	9.028	8.759	8.255
			C/C (N)	140	9.354	10.213	9.601	10.203	8.895	8.764	10.207	9.186	9.601	8.088	9.267	10.196	9.165	8.934	8.579
				*P* value	0.009	0.002	0.077	0.029	0.057	0.145	0.024	0.413	0.031	0.002	0.078	0.012	0.223	0.115	0.001
Q*Sl.his-3B*	AX-110931375	47,609,806	A/A (M^a^)	322	9.030	9.856	9.435	9.974	8.663	8.607	9.915	9.122	9.468	7.784	9.080	9.887	9.048	8.754	8.333
			G/G (N)	43	9.895	10.705	9.563	10.560	9.214	9.257	10.892	9.500	9.739	8.489	9.584	10.780	9.378	9.449	8.656
				*P* value	0.000	0.000	0.328	0.009	0.013	0.002	0.000	0.115	0.042	0.000	0.038	0.000	0.110	0.001	0.016
*QSl.his-2D1–1*	AX-110332825	15,841,686	T/T (M^a^)	278	8.924	9.771	9.284	9.857	8.587	8.473	9.847	8.948	9.377	7.735	8.928	9.788	8.908	8.664	8.295
			C/C (N)	85	9.693	10.458	9.838	10.428	9.080	9.302	10.507	9.788	9.827	8.271	9.718	10.522	9.501	9.327	8.569
				*P* value	0.000	0.000	0.005	0.001	0.005	0.000	0.000	0.000	0.000	0.000	0.000	0.000	0.002	0.000	0.009
	AX-108836084	17,230,342	G/G (M^a^)	281	8.915	9.767	9.267	9.856	8.608	8.462	9.830	8.966	9.369	7.719	8.902	9.798	8.915	8.679	8.268
			A/A (N)	88	9.817	10.510	9.845	10.478	9.120	9.338	10.586	9.766	9.893	8.280	9.672	10.539	9.547	9.278	8.636
				*P* value	0.000	0.000	0.003	0.000	0.004	0.000	0.000	0.000	0.000	0.000	0.000	0.000	0.001	0.000	0.001

^a^stands for the positive alleles detected in the RIL population; M and N stand for Bima4 and BainongAK58, respectively; SX, JS, HB, SD, and SC stand for Shaanxi, Jiangsu, Hebei, Shandong, and Sichuan, respectively

For SL, six and two QTL-associated SNP markers were analyzed for *QSl.his-4A-1* and *QSl.his-5D1*, respectively. Only AX-108955453 and AX-110409786 had significant differences of SL in 12 environments in the diverse wheat panel separately, and the positive alleles contributed by BainongAK58 increased 0.52 cm and 0.33 cm SL across these two loci, respectively. For *QSl.his-4D2–2* and *QSl.his-5B*, two and four QTL-associated SNP markers were analyzed, respectively. Significant differences of SL were found at AX-109924587 and AX-108886889 in 15 and 10 environments in the diverse wheat panel, respectively, and the positive alleles from BainongAK58 increased 0.46 cm and 0.31 cm SL across these two loci, respectively. Four QTL-associated SNP markers of *QSl.his-5A-2* were analyzed. Two markers (AX-109622137 and AX-110199675) showed significant differences of SL in 14 and 13 environments in the diverse wheat panel, respectively, and the positive alleles from Bima4 had a higher SL than BainongAK58 alleles. In addition, four QTL-associated SNP markers of *QSl.his-3A-2* and Q*Sl.his-3B* were analyzed, respectively. Only AX-111618763 and AX-110931375 showed significant differences of SL in 8 and 12 environments separately, while the positive alleles obtained from Bima4 across the two loci were unfavorable in the diverse wheat panel. Likewise, of five QTL-associated SNP markers of *QSl.his-2D1–1* analyzed, two had significant differences of SL in 15 environments, whereas the positive alleles from Bima4 across the two loci were unfavorable in the diverse wheat panel.

### Tracking of Bima4 allele in its derivatives and development of KASP markers

**Fig. 4:**
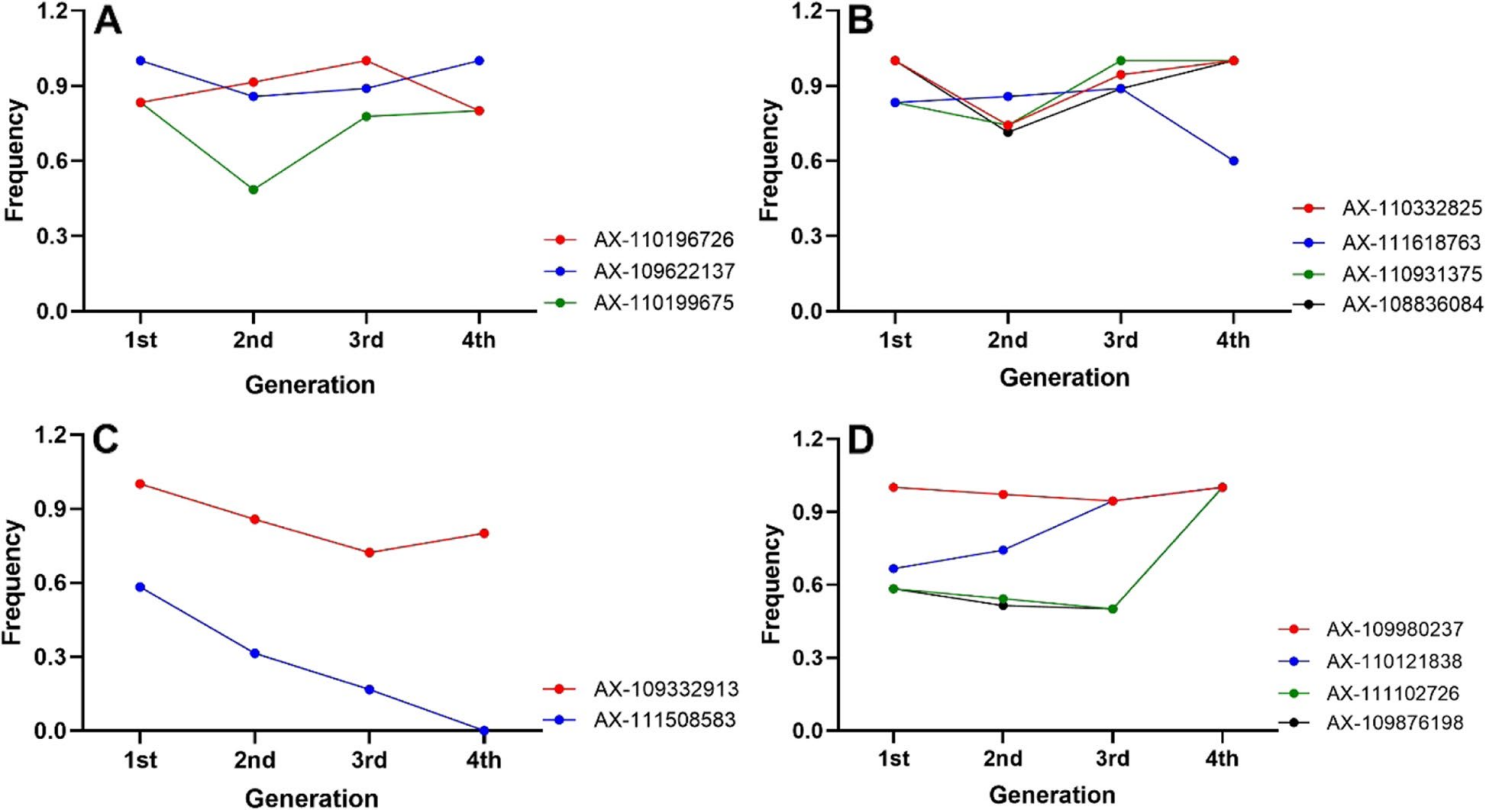
The frequency of Bima4-derived alleles of SNP markers related to the KNS or SL QTLs in four different generations

Of the 12 stable QTLs analyzed above, the positive alleles for 7 QTLs including 3 QTLs of KNS and 4 QTLs of SL were contributed by Bima4 in the RIL population. The transmission of Bima4 alleles in the QTL-associated SNP markers was determined using its 70 descendants. Among the seven QTLs, the transmission of Bima4 alleles at five loci (*QKns.his-7D2–1*, *QSl.his-5A-2*, *QSl.his-2D1–1*, *QSl.his-3A-2*, and Q*Sl.his-3B*) to its derivative descendants showed a relatively high frequency or an upward trend following the continuity of generations. For example, the Bima4 allele at the QTL-associated SNP marker (AX-110196726) of *QKns.his-7D2–1* showed an upward trend following the continuity of generations (Fig. 4A). The Bima4 alleles at two QTL-associated SNP markers (AX-109622137 and AX-110199675) of *QSl.his-5A-2* also presented a relatively high frequency or an upward trend following the continuity of generations (Fig. 4A). For these two QTLs, the positive effects of Bima4 alleles have been validated in the diverse wheat panel. Furthermore, two flanking SNP (AX-110945813 and AX-111490337) of *QKns.his-7D2–1*, which were located in the same bin with AX-110196726 and the physical distances between these two markers and AX-110196726 were only 0.11 Mb and 0.20 Mb, were successfully converted to kompetitive allele-specific PCR (KASP) markers (Table 5). Likewise, a KASP marker was developed from the flanking SNP (AX-108964722) of *QSl.his-5A-2*, which were located in the same bin with AX-109622137 and the interval between them was only 1.57 Mb. Similarly, for the three SL QTLs (*QSl.his-2D1–1*, *QSl.his-3A-2*, and Q*Sl.his-3B*), the Bima4 alleles at four markers (AX-110332825, AX-108836084, AX-111618763, and AX-110931375) showed a high frequency in its four derivate generations, respectively (Fig. 4B). In addition, for *QKns.his-4A*, the Bima4 allele showed a relatively high frequency (100–80.0%) in its four derivate generations at Ax-109332913, but a low frequency (30.0%) across all derivatives at the other marker AX-111508583 (Fig. 4C). A similar result could be observed for *QKns.his-5A-2*, e.g., the Bima4 alleles showed a high frequency or an upward trend following the continuity of generations at two markers (AX-109980237 and AX-110121838) but had a low frequency (50.0%) across all derivatives at another two markers (AX-111102726 and AX-109876198) (Fig. 4D).

**Table 5:** Information of the KASP markers developed in this study

QTL	KASP marker	Primer sequence		
		FAM	VIC	Common
*QKns.his-7D2–1*	KASP-AX-110945813	GTTCATTTTTCTCAGGGTTTGATGTATG	GTTCATTTTTCTCAGGGTTTGATGTATC	AAGCAGCCATGTCAGCTTCTCCTTA
	KASP-AX-111490337	CGTCAACTCGAGCTGTATTGTT	CGTCAACTCGAGCTGTATTGTC	ACGGTGCTGCATCATTTGGACACAA
*QSl.his-5A-2*	KASP-AX-108964722	ACTCGTTTTTGTTTCGGCGGCAA	ACTCGTTTTTGTTTCGGCGGCAG	CGAGAGTGGTACTACCGTCCAAAAT

## Discussion

### Comparison of the QTLs identified for KNS and SL with previous studies

Generally, the major QTLs consistent over environments may play a key role in modulating the agronomic traits of wheat cultivars and have great value for MAS in breeding programs. Based on genetic marker sequence flanking for KNS and SL QTLs and the genome sequence from Chinese Spring wheat (IWGSC V1.0) (http://www.wheatgenome.org/), physical positions of these stable QTLs detected in our study were compared with those reported previously. In the present study, four major QTLs, *Qkns.his-4A*, *Qkns.his-5A-2*, *Qkns.his-7D2–1* and *Qkns.his-7D2–2*, were identified for KNS. Of these, the locus *Qkns.his-4A* in the interval 122.03–122.24 cM on 4A was identified in four environments and the average value, and it was located in the interval 642.37–672.88 Mb. Using a 660 K wheat SNP array, Cui et al. [28] identified a major stable QTL, *qKnps-4A*, for KNS in the interval 680.40–683.64 Mb. Gao et al. [29] detected a QTL, *QKNS.caas-4AL*, in the interval 626.32–660.99 Mb using a 90 K wheat SNP array. Kirigwi et al. [9] identified two simple sequence repeat (SSR) loci, Xwmc89 and Xwmc420, related to KNS at positions 515.85 Mb and 538.22 Mb, respectively. There were also some other reported QTLs for KNS on 4A in previous studies [6, 13, 30]. Nevertheless, further research is needed to identify whether these genes are identical. *Qkns.his-5A-2*, mapped in three environments and the average value in the interval 106.50–125.16 cM on 5A in the present study, was located in the interval 549.34–572.81 Mb, whereas only a minor QTL for KNS reported by Wang et al. [13] in a single environment in marker interval Xgwm126-Xgwm291 on 5A positioned in the interval 671.39–698.19 Mb, indicating that *Qkns.his-5A-2* is likely to be a new KNS QTL. Likewise, *Qkns.his-7D2–1* and *Qkns.his-7D2–2*, mapped in the 2018XI environment and the average values in the interval 3.00–6.00 cM and 159 cM on 7D in the present study separately, were located in the interval 4.39–7.45 Mb and 526.36–530.75 Mb, respectively. There were also some other reported QTLs for KNS on 7D using SSR or RFLP markers [6, 31], but these markers could not be obtained or precisely located in the reference genome. So, we cannot determine whether the loci were nearby or identical with our results or not.

Of 18 QTLs for SL identified in the present study, 10 were detected in at least two environments. Of these, *Qsl.his-2D1–1* was identified in all four environments and the average value. The locus *Qsl.his-2D1–1* at the interval 29.00–37.00 cM explained 11.03–22.31% of the phenotypic variance and was located on 2D in the interval 13.25–36.89 Mb. Wu et al. [32] identified an SL-associated gene, *QSpl.nau-2D*, near position 23.02 Mb. Chai et al. [33] identified two QTLs (*QPht/Sl.cau-2D.1* and *QPht/Sl.cau-2D.2*) with pleiotropic effects on plant height and SL. *QPht/Sl.cau-2D.1* is a novel QTL located between SNP makers BS00022234_51 and BobWhite_rep_c63957_1472 near position 20.77 Mb, whereas *QPht/Sl.cau-2D.2* was located on the same genetic interval of *Rht8*. In addition, Sourdille et al. [15], Kumar et al. [10] and Suenaga et al. [16] identified one SSR locus, Xgwm261, associated with SL on 2D at position 19.6 Mb. The marker Xgwm261 is linked to the dwarf gene *Rht8*. Some previous studies [34–36] indicated that *Rht8* does not affect SL, but contrasting with other recent studies showing that *Rht8* introgression decreased SL with constant spikelet number [37]. Therefore, further research is needed to identify whether *Qsl.his-2D1–1* is nearby or identical with *Rht8*.

In the present study, *Qsl.his-5A-2* was identified in all four environments and the average value. It was at the interval 94.00–96.00 cM explained 4.65–8.38% of the phenotypic variance and was located on 5A in the interval 506.65–524.73 Mb. Fan et al. [38] identified an SL-associated gene, *qSl-5A.3*, in the interval 478.65–541.29 Mb. Kumar et al. [10] identified an SSR locus, Xgwm186, related to SL on 5A at position 471.71 Mb. Cui et al. [3] detected two QTLs related to SL on 5A at positions 444.92 and 682.71 Mb, respectively. In addition, Liu et al. [21] found an SNP maker, IAAV8258, related to SL on 5A at position 572.84 Mb. In our study, the locus *Qsl.his-5D1*, which accounted for 3.97–8.21% of the phenotypic variance and was identified in three environments and the average value at the interval 151–156 cM, was located on 5D1 in the interval 446.80–475.31 Mb. At a similar location to *Qsl.his-5D1*, marker Xgwm182 (439.22 Mb) on 5D affecting SL was reported by Kumar et al. [10]. Deng et al. [20] also reported a QTL, *QSl.sdau-5D*, linked to SL in marker interval Xbarc1097-Xcfd8 on 5D positioned in the interval 287.41–396.41 Mb.

### QTL effects in the diverse wheat panel

In this study, we conducted the allelic analysis based on phenotyping data collected from 15 environments in the diverse wheat panel. As the result showed, the positive alleles of *QKns.his-7D2–1*, *QKns.his-7D2–2*, *QSl.his-4A-1*, *QSl.his-5D1*, *QSl.his-4D2–2*, *QSl.his-5B*, and *QSl.his-5A-2* significantly increased KNS or SL in the diverse panel, suggesting that they are more universal in their effects. These important loci were very beneficial to pyramid breeding in wheat. On the other hand, the positive alleles of *QSl.his-2D1–1*, *QSl.his-3A-2*, and Q*Sl.his-3B* in the RIL population were unfavorable in the diverse wheat panel, indicating they may be population-specific QTL. In addition, for *QKns.his-5A-2* where the favorable allele was obtained from Bima4 in the RIL population, the Bima4 alleles showed negative effects at two loci (AX-109980237 and AX-110121838), but positive effects at another two loci (AX-111102726 and AX-109876198) in the diverse wheat panel. *QKns.his-5A-2* was mapped at the interval 106.00–125.00 cM and the physical distance between the marker AX-110121838 (549336395) and AX-111102726 (572237027) reached 22.90 Mb. These results indicated that there may be a great distance between these flanking markers and the peak markers for *QKns.his-5A-2*.

### Transmission of Bima4 alleles to its derivative descendants

Bima4 possesses many superior agronomic traits, especially high resistance to stripe rust, and it has played a crucial role in Chinese wheat breeding and production. In this study, the transmission of Bima4 alleles which showed positive effects in the RIL population at five loci (*QKns.his-7D2–1*, *QSl.his-5A-2*, *QSl.his-2D1–1*, *QSl.his-3A-2*, and Q*Sl.his-3B*) to its derivative descendants showed a relatively high frequency or an upward trend following the continuity of generations, suggesting that they underwent rigorous selection during breeding. These important loci in Bima4 had a great effect on the improvement of wheat breeding and should be studied intensively. Our results also accorded with previous reports by Guo et al. [23], Li et al. [25], Russell et al. [39], Pestsova and Röder [24] and Sjakste et al. [40], who found that the alleles selected preferentially in progeny were associated with advantageous traits. More importantly, the positive effects of the Bima4 alleles at these two loci *QKns.his-7D2–1* and *QSl.his-5A-2* have been validated in the diverse panel. We further developed two and one KASP markers for these two loci, which are valuable for MAS breeding. Similarly, a few KASP markers were developed in some studies for yield-related traits such as thousand kernel weight [41], grain length [42], productive tiller and fertile spikelet numbers [43], and plant height, SL, and total spikelet number per spike [26]. Compared with conventional molecular markers such as SSR, these KASP markers are more accurate and high-throughput, which can greatly improve the speed and efficiency of genomic selection for MAS breeding [44, 45].

## Conclusions

A high-density genetic map, consisting 2356 bin markers (14,812 SNPs) and spanning 4141.24 cM, was constructed using the wheat 55 K genotyping assay in the RIL population with 248 lines derived from the two founder genotypes of wheat, Bima4 and BainongAK58. A total of seven and 18 QTLs were identified for KNS and SL, respectively, and they were distributed on 11 chromosomes. The allele effects of the flanking markers for 12 stable QTLs including four QTLs for KNS and eight QTLs for SL were estimated based on phenotyping data collected from 15 environments in a diverse wheat panel including 384 elite cultivars and breeding lines. The positive alleles at seven loci significantly increased KNS or SL in the diverse panel, suggesting that they are more universal in their effects and are valuable for gene pyramiding in breeding programs. The transmission of the Bima4 alleles indicated that the favorite alleles at five loci showed a relatively high frequency or an upward trend following the continuity of generations, suggesting that they underwent rigorous selection during breeding. The positive effects of the Bima4 alleles at two loci *QKns.his-7D2–1* and *QSl.his-5A-2* have been validated in the diverse panel, and two and one KASP markers were developed for these two loci. Our results are useful for knowing the molecular mechanisms of founder parents and future molecular breeding in wheat.

## Methods

### Plant materials

The QTL mapping population containing 248 RILs (F_7_) were derived from the F_2_ population of the cross BainongAK58 × Bima4 by the single seed descent method. Bima4 is both an important founder genotype and a widely grown cultivar with high yield potential and wide environmental adaptability. BainongAK58 has many important traits such as lodging resistance, disease resistance, and yield potential. A diverse wheat panel containing 384 elite cultivars and breeding lines was used for QTL validation in this study, and detailed information was described in Li et al. [46]. Seventy cultivars derived from Bima4 were also included (Table S3), and there are 12, 35, 18, and 5 accessions in the first, second, third, and fourth generations of the derivatives, respectively. Seeds of all accessions were provided by the National Crop Gene Bank, Chinese Academy of Agricultural Sciences, Beijing.

### Field trials and data analysis

Field experiments for the RIL population were performed at Xinxiang (117.17°E, 40.69°N) in 2017, 2018 and 2019 (2017XI, 2018XI and 2019XI) and Huixian (116.41°E, 39.91°N) in 2018 (2018HU) in Henan province in a randomized block design. Thirty seeds for each line were evenly planted in two rows of 2 m in length and 25 cm between rows. The main spikes of at least 6 plants in each plot were measured to investigate the SL and KNS when ripening. Broad-sense heritability across different environments was calculated based on the ANOVA model as described by Wu et al. [47].

The diverse wheat panel was planted in randomized complete blocks with two or three replicates in five major wheat ecological regions of China in the 2007, 2008, and 2009 planting seasons as described previously [46], including Yangling (108.08°E, 34.27°N) in Shaanxi Province, Tai′an (117.09°E, 36.21°N) in Shandong Province, Shijiazhuang (114.52°E, 38.05°N) in Hebei Province, Chengdu (104.08°E, 30.66°N) in Sichuan Province, and Yangzhou (119.42°E, 32.40°N) in Jiangsu Province. Two hundred seeds for each cultivar were evenly planted in five rows 2 m long and spaced 30 cm apart. The SL and KNS traits were assessed from 10 spikes randomly sampled from the centre of each plot before harvesting.

### SNP genotyping, linkage map construction and QTL detection

The RIL lines and two parents were genotyped with the high-density Illumina Infinium iSelect 55 K SNP array by China Golden Marker (Beijing, China). The diverse wheat panel was also genotyped using the same SNP array [46]. After excluding the monomorphic markers in the RIL population, markers retained were analyzed using the BIN function of IciMapping 4.2 (http://www.isbreeding.net) based on their segregation patterns with the parameters of “Missing Rates” and “Distortion Value” being set as 20 and 0.001, respectively. Only one marker with the least “Missing Rate” was chosen to represent each bin for constructing genetic maps and QTL mapping in this study. Linkage analysis was performed with IciMapping 4.2 using the default mapping function, and the resulting genetic map was displayed with MapChart v2.2 (http://www.biometris.nl/uk/Software/MapChart/). QTLs for SL and KNS in each environment and the average values across all environments were detected using the inclusive composite interval mapping (ICIM) function of IciMapping 4.2 and LOD score values ≥2.5.

### QTL validation and development of KASP markers

For certain stable QTLs identified for SL and KNS in the RIL population, the QTL-associated flanking markers were validated using the diverse wheat panel. Furthermore, of the stable QTLs analyzed at which the positive alleles were contributed by Bima4 in the RIL population, the transmission of Bima4 alleles at the QTL-associated SNP markers were also determined using its 70 descendants. SNP markers highly associated with a specific QTL were selected and converted to KASP markers.

## Supplementary Information

**Additional file 1: Table S1.** Genetic map of wheat developed using the RIL population derived from the cross between BainongAK58 and Bima4. **Table S2.** Phenotypic variation for SL and KNS in the RIL population. **Table S3.** Seventy wheat cultivars derived from Bima4.

## Data Availability

The main datasets supporting the conclusions of this article are included within the article and its additional file. A small piece of data used for QTL validation in this study, including the diverse wheat panel and their phenotype and SNP genotyping data, is available in this reference which we published before (Li X, Xu X, Liu W, Li X, Yang X, Ru Z, Li L. Dissection of superior alleles for yield-related traits and their distribution in important cultivars of wheat by association mapping. Front Plant Sci. 2020;11: 175. DOI:10.3389/fpls.2020.00175). All datasets are available from the corresponding author on reasonable request.

## Abbreviations

KNS: Kernel number per spike
SL: Spike length
QTL: Quantitative trait loci
RIL: Recombinant inbred line
KASP: Kompetitive allele-specific PCR
MAS: Marker-assisted selection
