# Supplementary material for: Identification and validation of QTLs for kernel number per spike and spike length in two founder genotypes of wheat
Source: BMC Plant Biol. 2022 Mar 26;22:146. doi: 10.1186/s12870-022-03544-6 (PMC8962171; doi:10.1186/s12870-022-03544-6)
Supplement: Supplementary file 1 — Additional file 1: Table S1. Genetic map of wheat developed using the RIL population derived from the cross between BainongAK58 and Bima4. Table S2. Phenotypic variation for SL and KNS in the RIL population. Table S3. Seventy wheat cultivars derived from Bima4. [file 12870_2022_3544_MOESM1_ESM.pdf]

Table S1 Genetic map of wheat developed using the RIL population derived from the cross between BainongAK58 and Bima4

ChromsomeID	Chromsome	MarkerName	Interval(cM)	Position(cM)
1	1A	AX-109551739	0	0
1	1A	AX-110671977	0	0
1	1A	AX-111660453	0	0
1	1A	AX-108914803	0	0
1	1A	AX-111123203	11.3299	11.3299
1	1A	AX-86172827	11.3299	11.3299
1	1A	AX-111707816	6.6744	18.0043
1	1A	AX-109968922	6.6744	18.0043
1	1A	AX-110488387	0.6356	18.64
1	1A	AX-109999652	0.6356	18.64
1	1A	AX-110736420	0.6356	18.64
1	1A	AX-109836978	0.6356	18.64
1	1A	AX-110483730	0.6356	18.64
1	1A	AX-111233876	0.6356	18.64
1	1A	AX-110472710	0.2155	18.8555
1	1A	AX-109894293	2.73	21.5855
1	1A	AX-110167459	2.4573	24.0428
1	1A	AX-109966533	0.6438	24.6866
2	1A	AX-109897215	0	0
2	1A	AX-110511743	0.2058	0.2058
2	1A	AX-108842309	13.7794	13.9852
2	1A	AX-109295825	13.7794	13.9852
2	1A	AX-108770405	3.2453	17.2305
2	1A	AX-108868021	0.4386	17.6691
2	1A	AX-109511562	0.4386	17.6691
2	1A	AX-110388488	0.4386	17.6691
2	1A	AX-108772599	0.2119	17.881
2	1A	AX-110627812	0.2075	18.0884
2	1A	AX-110962638	0.2075	18.0884
2	1A	AX-111560032	0.2075	18.0884
2	1A	AX-94710443	0.2101	18.2985
2	1A	AX-109428041	0.211	18.5095
2	1A	AX-111512097	0.8475	19.357
2	1A	AX-111670056	0.6356	19.9926
2	1A	AX-109585803	3.3532	23.3459
2	1A	AX-111630751	3.3532	23.3459
2	1A	AX-111484005	3.3532	23.3459
2	1A	AX-109927782	1.4835	24.8294
2	1A	AX-111491732	1.4835	24.8294
2	1A	AX-109927153	1.4835	24.8294
2	1A	AX-110911122	0.2075	25.0368

2	1A	AX-109914553	0.2075	25.0368
2	1A	AX-111075987	0.2075	25.0368
2	1A	AX-111081504	0.2092	25.246
2	1A	AX-108896257	0.2092	25.246
2	1A	AX-109372364	0.2092	25.246
2	1A	AX-108881505	0.422	25.668
2	1A	AX-109938673	1.3046	26.9726
2	1A	AX-109378567	0.431	27.4037
2	1A	AX-108780484	0.4274	27.831
2	1A	AX-111015470	0.4274	27.831
2	1A	AX-109864205	0.4274	27.831
2	1A	AX-108877432	0.4274	27.831
2	1A	AX-109075767	0.4274	27.831
2	1A	AX-110549037	0.4237	28.2548
2	1A	AX-111637533	0.4237	28.2548
2	1A	AX-111705658	1.7474	30.0022
2	1A	AX-110501537	1.7474	30.0022
2	1A	AX-110605358	1.7474	30.0022
2	1A	AX-108841557	1.7474	30.0022
2	1A	AX-111176006	1.7474	30.0022
2	1A	AX-109585123	1.7474	30.0022
2	1A	AX-108774722	1.7474	30.0022
2	1A	AX-109083040	1.7474	30.0022
2	1A	AX-109972218	1.7474	30.0022
2	1A	AX-109519556	1.7474	30.0022
2	1A	AX-110714545	1.7474	30.0022
2	1A	AX-89340634	1.7474	30.0022
2	1A	AX-110917923	1.7474	30.0022
2	1A	AX-111484839	1.9747	31.9769
2	1A	AX-110500599	1.7474	33.7244
2	1A	AX-108888974	1.7474	33.7244
2	1A	AX-111139240	1.7474	33.7244
2	1A	AX-110397824	1.7474	33.7244
2	1A	AX-109905095	1.7474	33.7244
2	1A	AX-109938347	1.7474	33.7244
2	1A	AX-110402352	1.7474	33.7244
2	1A	AX-111460308	1.7474	33.7244
2	1A	AX-108913233	1.7474	33.7244
2	1A	AX-108844296	1.7474	33.7244
2	1A	AX-89504717	1.5156	35.24
2	1A	AX-110527733	1.5156	35.24
2	1A	AX-109959060	1.5156	35.24
2	1A	AX-108988313	1.5156	35.24
2	1A	AX-111671664	1.5156	35.24

2	1A	AX-109367744	0.4202	35.6602
2	1A	AX-109954487	0.4202	36.0803
2	1A	AX-109857531	0.4202	36.0803
2	1A	AX-109955303	0.2092	36.2895
2	1A	AX-111057357	0.2092	36.2895
2	1A	AX-111460918	0.2092	36.2895
2	1A	AX-109385015	0.2092	36.2895
2	1A	AX-111692232	0.2092	36.2895
2	1A	AX-109986286	1.2878	37.5774
2	1A	AX-109949918	1.2878	37.5774
2	1A	AX-110944881	1.2878	37.5774
2	1A	AX-109026070	1.2878	37.5774
2	1A	AX-109288274	1.2878	37.5774
2	1A	AX-110493162	1.2878	37.5774
2	1A	AX-109436242	1.2878	37.5774
2	1A	AX-110504930	1.2878	37.5774
2	1A	AX-111592607	1.2878	38.8652
2	1A	AX-109481746	1.2878	40.1531
2	1A	AX-111614695	0.211	40.364
2	1A	AX-110476734	0.4237	40.7878
2	1A	AX-109849289	0.2128	41.0005
2	1A	AX-110917687	0.2128	41.0005
2	1A	AX-111014967	0.4292	41.4297
2	1A	AX-110689839	0.4292	41.4297
2	1A	AX-111716711	0.4292	41.4297
2	1A	AX-111619451	0.4292	41.4297
2	1A	AX-110483935	0.4292	41.4297
2	1A	AX-110019998	0.4292	41.4297
2	1A	AX-109281866	0.4292	41.4297
2	1A	AX-111685647	0.4292	41.4297
2	1A	AX-111472835	0.4292	41.4297
2	1A	AX-111650098	0.4292	41.4297
2	1A	AX-111007356	0.4292	41.4297
2	1A	AX-109900562	0.4292	41.4297
2	1A	AX-110054722	0.4292	41.4297
2	1A	AX-108855105	0.4292	41.4297
2	1A	AX-109335959	0.4292	41.4297
2	1A	AX-110446721	0.4292	41.4297
2	1A	AX-108922498	0.4292	41.4297
2	1A	AX-110010493	0.4292	41.4297
2	1A	AX-111577225	0.4292	41.4297
2	1A	AX-108984979	0.4292	41.4297
2	1A	AX-111187227	0.4292	41.4297
2	1A	AX-109876537	0.4292	41.4297

2	1A	AX-110525765	0.2128	41.6425
2	1A	AX-110906010	0.2128	41.6425
2	1A	AX-109054416	0.2128	41.6425
2	1A	AX-109445371	0.2128	41.6425
2	1A	AX-108933804	0.2128	41.6425
2	1A	AX-110671966	0.2128	41.6425
2	1A	AX-109940152	0.6438	42.2863
2	1A	AX-108747751	0.6438	42.2863
2	1A	AX-109006135	0.6438	42.2863
2	1A	AX-111104213	0.6438	42.2863
2	1A	AX-109356451	0.6438	42.2863
2	1A	AX-110943562	0.6438	42.2863
2	1A	AX-111604545	0.6438	42.2863
2	1A	AX-110372348	0.6438	42.2863
2	1A	AX-86169491	0.6438	42.2863
2	1A	AX-109429970	0.6438	42.2863
2	1A	AX-109837980	0.6438	42.2863
2	1A	AX-109839840	0.6438	42.2863
2	1A	AX-109838548	0.6438	42.2863
2	1A	AX-109388848	0.6438	42.2863
2	1A	AX-109856441	0.6438	42.2863
2	1A	AX-109925531	0.6438	42.2863
2	1A	AX-111120481	0.6438	42.2863
2	1A	AX-108809445	0.6438	42.2863
2	1A	AX-110061509	0.6438	42.2863
2	1A	AX-110506780	0.6438	42.2863
2	1A	AX-111653805	0.6438	42.2863
2	1A	AX-110195336	0.6438	42.2863
2	1A	AX-109831421	0.6438	42.2863
2	1A	AX-94446416	0.6438	42.2863
2	1A	AX-108969133	0.6438	42.2863
2	1A	AX-111717479	0.6438	42.2863
2	1A	AX-111139812	0.6438	42.2863
2	1A	AX-108868514	0.6438	42.2863
2	1A	AX-110384221	0.6438	42.2863
2	1A	AX-111000942	0.6438	42.2863
2	1A	AX-111472392	0.6438	42.2863
2	1A	AX-110988058	0.6438	42.2863
2	1A	AX-109474889	0.6438	42.2863
2	1A	AX-109361287	0.6438	42.2863
2	1A	AX-109894800	0.6438	42.2863
2	1A	AX-109408475	0.6438	42.2863
2	1A	AX-108817901	0.6438	42.2863
2	1A	AX-108943547	0.6438	42.2863

2	1A	AX-109446822	0.6438	42.2863
2	1A	AX-110972193	0.6438	42.2863
2	1A	AX-108836436	0.6438	42.2863
2	1A	AX-109379579	0.6438	42.2863
2	1A	AX-108875042	0.6438	42.2863
2	1A	AX-110406943	0.6438	42.2863
2	1A	AX-110381811	0.6438	42.2863
2	1A	AX-108791013	0.6438	42.2863
2	1A	AX-111554735	0.6438	42.2863
2	1A	AX-110129942	0.6438	42.2863
2	1A	AX-111580125	0.6438	42.2863
2	1A	AX-110364488	0.6438	42.2863
2	1A	AX-111005048	0.6438	42.2863
2	1A	AX-108942357	0.6438	42.2863
2	1A	AX-110006972	0.6438	42.2863
2	1A	AX-110940534	0.6438	42.2863
2	1A	AX-111523338	0.6438	42.2863
2	1A	AX-109964909	0.6438	42.2863
2	1A	AX-109869754	0.6438	42.2863
2	1A	AX-109331801	0.6438	42.2863
2	1A	AX-109451916	0.6438	42.2863
2	1A	AX-109949165	0.6438	42.2863
2	1A	AX-109516179	0.6438	42.2863
2	1A	AX-94408765	0.6438	42.2863
2	1A	AX-108818446	0.6438	42.2863
2	1A	AX-110429962	0.6438	42.2863
2	1A	AX-111544107	0.6438	42.2863
2	1A	AX-86174070	0.6438	42.2863
2	1A	AX-111533772	0.2119	42.4982
2	1A	AX-108792730	0.2119	42.4982
2	1A	AX-111554031	0.2119	42.4982
2	1A	AX-111071459	0.2119	42.4982
2	1A	AX-110994106	0.2119	42.4982
2	1A	AX-108976051	0.2119	42.4982
2	1A	AX-110450556	0.2119	42.4982
2	1A	AX-111491669	0.2119	42.4982
2	1A	AX-109998331	0.2119	42.4982
2	1A	AX-109373293	0.2119	42.4982
2	1A	AX-109296950	0.2119	42.4982
2	1A	AX-111509307	0.2119	42.4982
2	1A	AX-109613064	0.2119	42.4982
2	1A	AX-108830701	0.2119	42.4982
2	1A	AX-111140587	0.2119	42.4982
2	1A	AX-109005483	0.2119	42.4982

2	1A	AX-111572655	0.2119	42.4982
2	1A	AX-110376497	0.2119	42.4982
2	1A	AX-108777813	0.2119	42.4982
2	1A	AX-109299211	0.2119	42.4982
2	1A	AX-108841071	0.2119	42.4982
2	1A	AX-111553287	0.2119	42.4982
2	1A	AX-110475253	0.2119	42.4982
2	1A	AX-109351766	0.2119	42.4982
2	1A	AX-111652012	0.2119	42.4982
2	1A	AX-111196957	0.2119	42.4982
2	1A	AX-110921788	0.2119	42.4982
2	1A	AX-111487409	0.2119	42.4982
2	1A	AX-110546251	0.2119	42.4982
2	1A	AX-109444809	0.2119	42.4982
2	1A	AX-110928652	0.2119	42.4982
2	1A	AX-108927864	0.2119	42.4982
2	1A	AX-110439012	0.2119	42.4982
2	1A	AX-108965988	0.2119	42.4982
2	1A	AX-111516716	0.2119	42.4982
2	1A	AX-109967116	0.2119	42.4982
2	1A	AX-108866053	0.2119	42.4982
2	1A	AX-110511731	0.2119	42.4982
2	1A	AX-111288736	0.2119	42.4982
2	1A	AX-111454083	0.2119	42.4982
2	1A	AX-108968461	0.2119	42.4982
2	1A	AX-110947388	0.2119	42.4982
2	1A	AX-109334569	0.2119	42.4982
2	1A	AX-109388909	0.2119	42.4982
2	1A	AX-110567183	0.2119	42.4982
2	1A	AX-109310093	0.2119	42.4982
2	1A	AX-111525112	0.2119	42.4982
2	1A	AX-110018834	0.2119	42.4982
2	1A	AX-111141894	0.2119	42.4982
2	1A	AX-109971512	0.2119	42.4982
2	1A	AX-109519346	0.2119	42.4982
2	1A	AX-109337231	0.2119	42.4982
2	1A	AX-109328719	0.2119	42.4982
2	1A	AX-111051375	0.2119	42.4982
2	1A	AX-111624842	0.2119	42.4982
2	1A	AX-108820820	0.2119	42.4982
2	1A	AX-110473633	0.2119	42.4982
2	1A	AX-108973818	0.2119	42.4982
2	1A	AX-109326726	0.2119	42.4982
2	1A	AX-95233406	0.2119	42.4982

2	1A	AX-108869027	0.2119	42.4982
2	1A	AX-108906714	0.2119	42.4982
2	1A	AX-108994522	0.2119	42.4982
2	1A	AX-109477032	0.2119	42.4982
2	1A	AX-109522865	0.2119	42.4982
2	1A	AX-111169839	0.2119	42.4982
2	1A	AX-110499477	0.2119	42.4982
2	1A	AX-111701587	0.2119	42.71
2	1A	AX-111736828	0.2119	42.71
2	1A	AX-109032174	0.2119	42.71
2	1A	AX-108800232	0.2119	42.71
2	1A	AX-109385130	0.2119	42.71
2	1A	AX-110593666	0.2119	42.71
2	1A	AX-109382288	0.2119	42.71
2	1A	AX-109304452	0.2119	42.71
2	1A	AX-111458532	0.2119	42.71
2	1A	AX-108886049	0.4274	43.1374
2	1A	AX-111635819	0.4274	43.1374
2	1A	AX-108899002	0.4274	43.1374
2	1A	AX-110516805	0.4274	43.1374
2	1A	AX-108800654	0.4274	43.1374
2	1A	AX-109606033	0.4274	43.1374
2	1A	AX-109980898	0.4274	43.1374
2	1A	AX-111074719	0.4274	43.1374
2	1A	AX-111697970	0.4274	43.1374
2	1A	AX-111464281	0.4274	43.1374
2	1A	AX-111261118	0.4274	43.1374
2	1A	AX-108861860	0.4274	43.1374
2	1A	AX-110488785	0.4274	43.1374
2	1A	AX-108777487	0.4274	43.1374
2	1A	AX-109281345	0.4274	43.1374
2	1A	AX-111727637	0.2128	43.3502
2	1A	AX-110056021	0.2128	43.3502
2	1A	AX-110547230	0.2128	43.3502
2	1A	AX-110043429	0.2128	43.3502
2	1A	AX-111455543	0.2128	43.3502
2	1A	AX-108874109	0.2128	43.3502
2	1A	AX-109843719	0.2128	43.3502
2	1A	AX-110607889	0.2128	43.3502
2	1A	AX-109335291	0.2128	43.3502
2	1A	AX-111052075	0.2128	43.3502
2	1A	AX-110400074	0.2128	43.3502
2	1A	AX-110401529	0.2128	43.3502
2	1A	AX-109902115	0.2128	43.3502

2	1A	AX-111483838	0.2128	43.3502
2	1A	AX-109949075	0.2128	43.3502
2	1A	AX-109527244	0.2128	43.3502
2	1A	AX-111138197	0.2128	43.3502
2	1A	AX-109562522	0.2128	43.3502
2	1A	AX-111217732	0.2128	43.3502
2	1A	AX-109880511	0.2128	43.3502
2	1A	AX-109883215	0.2128	43.3502
2	1A	AX-110001484	0.2128	43.3502
2	1A	AX-111490235	0.2128	43.3502
2	1A	AX-108868008	0.2128	43.3502
2	1A	AX-111696606	0.2128	43.3502
2	1A	AX-109516281	0.2128	43.3502
2	1A	AX-109995513	0.2128	43.3502
2	1A	AX-111018850	0.2128	43.3502
2	1A	AX-109887522	0.2128	43.3502
2	1A	AX-111622699	0.2128	43.3502
2	1A	AX-110024066	0.2128	43.3502
2	1A	AX-111501667	0.2128	43.3502
2	1A	AX-111673303	0.2128	43.3502
2	1A	AX-111057434	0.2128	43.3502
2	1A	AX-109511730	0.2128	43.3502
2	1A	AX-108730735	0.2128	43.3502
2	1A	AX-111495222	0.2128	43.3502
2	1A	AX-110632617	0.2128	43.3502
2	1A	AX-108943724	0.2128	43.3502
2	1A	AX-109470754	0.2128	43.3502
2	1A	AX-108775105	0.2128	43.3502
2	1A	AX-111620530	0.2128	43.3502
2	1A	AX-109424706	0.2128	43.3502
2	1A	AX-111593959	0.2128	43.3502
2	1A	AX-111067521	0.2128	43.3502
2	1A	AX-110415470	0.2128	43.3502
2	1A	AX-110672209	0.2128	43.3502
2	1A	AX-109969238	0.2128	43.3502
2	1A	AX-110121645	0.2128	43.3502
2	1A	AX-109312262	0.2128	43.3502
2	1A	AX-111656703	0.2128	43.3502
2	1A	AX-111585507	0.2128	43.3502
2	1A	AX-108946403	0.2128	43.3502
2	1A	AX-109427606	0.2128	43.3502
2	1A	AX-110014409	0.2128	43.3502
2	1A	AX-109378286	0.2128	43.3502
2	1A	AX-108865536	0.2128	43.3502

2	1A	AX-111017895	0.2128	43.3502
2	1A	AX-109947558	0.2128	43.3502
2	1A	AX-110502923	0.2128	43.3502
2	1A	AX-111482821	0.2128	43.3502
2	1A	AX-109021990	0.2128	43.3502
2	1A	AX-94734074	0.2128	43.3502
2	1A	AX-108845809	0.2128	43.3502
2	1A	AX-109349783	0.2128	43.3502
2	1A	AX-109572531	0.2128	43.3502
2	1A	AX-110044058	0.2128	43.3502
2	1A	AX-110126844	0.2128	43.3502
2	1A	AX-108894614	0.2137	43.5639
2	1A	AX-109994147	0.2137	43.5639
2	1A	AX-110735773	0.2137	43.5639
2	1A	AX-110058611	0.2137	43.5639
2	1A	AX-111586048	0.2137	43.5639
2	1A	AX-108795741	0.2137	43.5639
2	1A	AX-108869577	0.2137	43.5639
2	1A	AX-109928433	0.2137	43.5639
2	1A	AX-108764090	0.2137	43.5639
2	1A	AX-110628967	0.2137	43.5639
2	1A	AX-111163383	0.2137	43.5639
2	1A	AX-110035801	0.2137	43.7775
2	1A	AX-109601331	0.2137	43.7775
2	1A	AX-111017486	0.2137	43.7775
2	1A	AX-110978709	0.2137	43.7775
2	1A	AX-109828311	0.2137	43.7775
2	1A	AX-109036598	0.2137	43.7775
2	1A	AX-110010924	0.2137	43.7775
2	1A	AX-111560636	0.2137	43.7775
2	1A	AX-110940778	0.2137	43.7775
2	1A	AX-109397850	0.2137	43.7775
2	1A	AX-111254001	0.2137	43.7775
2	1A	AX-109384586	0.2137	43.7775
2	1A	AX-111485245	0.2137	43.7775
2	1A	AX-111026946	0.2137	43.7775
2	1A	AX-109490765	0.2137	43.7775
2	1A	AX-109359225	0.2137	43.7775
2	1A	AX-108842045	0.2137	43.7775
2	1A	AX-108764447	0.2137	43.7775
2	1A	AX-109961353	0.2137	43.7775
2	1A	AX-109993983	0.2137	43.7775
2	1A	AX-110531505	0.2137	43.7775
2	1A	AX-111035517	0.2137	43.7775

2	1A	AX-111703139	0.2137	43.7775
2	1A	AX-109455012	0.2137	43.7775
2	1A	AX-109470628	0.2137	43.7775
2	1A	AX-111563293	0.2137	43.7775
2	1A	AX-109822299	0.2222	43.9998
2	1A	AX-111540903	0.6697	44.6694
2	1A	AX-108752091	0.6697	44.6694
2	1A	AX-89694178	0.6697	44.6694
2	1A	AX-111682431	0.6697	44.6694
2	1A	AX-111597054	0.6697	44.6694
2	1A	AX-110083753	0.6697	44.6694
2	1A	AX-108990531	0.6697	44.6694
2	1A	AX-111076721	0.6697	44.6694
2	1A	AX-109424288	0.4274	45.0968
2	1A	AX-111010753	0.4274	45.0968
2	1A	AX-109498847	0.4274	45.0968
2	1A	AX-110999747	0.4274	45.0968
2	1A	AX-111585289	0.4274	45.0968
2	1A	AX-109951554	0.4274	45.0968
2	1A	AX-89488696	0.4274	45.0968
2	1A	AX-109447567	0.4274	45.0968
2	1A	AX-111693378	0.4274	45.0968
2	1A	AX-109905627	0.4274	45.0968
2	1A	AX-110629172	0.4274	45.0968
2	1A	AX-110062407	0.4274	45.0968
2	1A	AX-109985006	0.4274	45.0968
2	1A	AX-108909230	0.4274	45.0968
2	1A	AX-109579999	0.4274	45.0968
2	1A	AX-111053013	0.4274	45.0968
2	1A	AX-108965029	0.4274	45.0968
2	1A	AX-109363171	0.4274	45.0968
2	1A	AX-110049395	0.4274	45.0968
2	1A	AX-108762805	0.4274	45.0968
2	1A	AX-110025207	0.4274	45.0968
2	1A	AX-110391018	0.4274	45.0968
2	1A	AX-94942439	0.4274	45.0968
2	1A	AX-111153921	0.4274	45.0968
2	1A	AX-109986959	0.4274	45.0968
2	1A	AX-110938734	0.4274	45.0968
2	1A	AX-95201266	0.4274	45.0968
2	1A	AX-108968444	0.4274	45.0968
2	1A	AX-110608648	0.6438	45.7406
2	1A	AX-109866086	0.6438	45.7406
2	1A	AX-111533353	0.6438	45.7406

2	1A	AX-110076368	0.6438	45.7406
2	1A	AX-110507437	0.6438	45.7406
2	1A	AX-110918774	0.6438	45.7406
2	1A	AX-108905837	0.6438	45.7406
2	1A	AX-110414497	0.6438	45.7406
2	1A	AX-109827458	0.6438	45.7406
2	1A	AX-111091356	0.6438	45.7406
2	1A	AX-110712805	0.6438	45.7406
2	1A	AX-108797381	0.6438	45.7406
2	1A	AX-109403548	0.6438	45.7406
2	1A	AX-110548582	0.6438	45.7406
2	1A	AX-110483249	0.6438	45.7406
2	1A	AX-108969215	0.6438	45.7406
2	1A	AX-110545296	0.6438	45.7406
2	1A	AX-109284745	0.6438	45.7406
2	1A	AX-109882341	0.6438	45.7406
2	1A	AX-109277966	0.6438	45.7406
2	1A	AX-108954745	0.6438	45.7406
2	1A	AX-108868172	0.6438	45.7406
2	1A	AX-111629871	0.6438	45.7406
2	1A	AX-110395170	0.6438	45.7406
2	1A	AX-109891043	0.6438	45.7406
2	1A	AX-110047605	0.6438	45.7406
2	1A	AX-110470934	0.6438	45.7406
2	1A	AX-109376078	0.6438	45.7406
2	1A	AX-109393672	0.6438	45.7406
2	1A	AX-110030576	0.6438	45.7406
2	1A	AX-110949263	0.6438	45.7406
2	1A	AX-111132934	0.6438	45.7406
2	1A	AX-111732605	0.6438	45.7406
2	1A	AX-95164417	0.6438	45.7406
2	1A	AX-109600894	0.6438	45.7406
2	1A	AX-108954549	0.4329	46.1735
2	1A	AX-110584782	0.2137	46.3872
2	1A	AX-111097762	0.6438	47.031
2	1A	AX-110552588	0.2128	47.2438
2	1A	AX-108731433	0.2128	47.2438
2	1A	AX-109596516	0.2128	47.2438
2	1A	AX-111578749	0.2119	47.4557
2	1A	AX-109387872	0.2119	47.4557
2	1A	AX-109038718	0.2119	47.4557
2	1A	AX-109989656	0.2119	47.4557
2	1A	AX-110640338	0.2119	47.4557
2	1A	AX-110520028	0.2119	47.4557

2	1A	AX-108805501	0.2119	47.4557
2	1A	AX-108998067	0.2119	47.4557
2	1A	AX-110532052	0.4274	47.883
2	1A	AX-110564663	0.4292	48.3122
2	1A	AX-111013535	0.4292	48.3122
2	1A	AX-110554067	0.4292	48.3122
2	1A	AX-109983898	0.4292	48.3122
2	1A	AX-110567101	2.0011	50.3133
2	1A	AX-109044183	2.0011	50.3133
2	1A	AX-110642950	0.2146	50.5279
2	1A	AX-110058737	0.2146	50.5279
2	1A	AX-110380961	0.2146	50.5279
2	1A	AX-110943399	0.2146	50.5279
2	1A	AX-109921850	0.2146	50.5279
2	1A	AX-109384168	0.2155	50.7434
2	1A	AX-110911618	0.4348	51.1782
2	1A	AX-109865440	0.4348	51.1782
2	1A	AX-108893431	0.4348	51.1782
2	1A	AX-110085541	0.4348	51.1782
2	1A	AX-109864216	0.4348	51.1782
2	1A	AX-109982239	0.4348	51.1782
2	1A	AX-109829852	0.4348	51.1782
2	1A	AX-111069240	0.4348	51.1782
2	1A	AX-109521252	0.4348	51.1782
2	1A	AX-110053294	0.4348	51.1782
2	1A	AX-111545796	0.4348	51.1782
2	1A	AX-110399569	0.4348	51.1782
2	1A	AX-111600244	0.4348	51.1782
2	1A	AX-109984878	0.4348	51.1782
2	1A	AX-110426169	0.4348	51.1782
2	1A	AX-109931279	0.4348	51.1782
2	1A	AX-109835479	0.4348	51.1782
2	1A	AX-109339822	0.4348	51.1782
2	1A	AX-111503582	0.4348	51.1782
2	1A	AX-95629020	0.4348	51.1782
2	1A	AX-110926621	0.4292	51.6074
2	1A	AX-109305333	0.4292	51.6074
2	1A	AX-110515835	0.4292	51.6074
2	1A	AX-111467899	0.4292	51.6074
2	1A	AX-111501565	0.4292	51.6074
2	1A	AX-111729375	0.4292	51.6074
2	1A	AX-109447872	0.4292	51.6074
2	1A	AX-108861931	0.4292	51.6074
2	1A	AX-108774966	0.4292	51.6074

2	1A	AX-111668238	0.4292	51.6074
2	1A	AX-111476218	0.4292	51.6074
2	1A	AX-109538739	0.4274	52.0347
2	1A	AX-108804713	0.2101	52.2448
2	1A	AX-111681662	0.2101	52.2448
2	1A	AX-111563037	0.2101	52.2448
2	1A	AX-109823133	0.2101	52.2448
2	1A	AX-109489615	0.2101	52.2448
2	1A	AX-111733108	0.8511	53.096
2	1A	AX-110708678	0.8511	53.096
2	1A	AX-109904153	0.8511	53.096
2	1A	AX-110427428	0.2119	53.3078
2	1A	AX-111586792	0.2119	53.3078
2	1A	AX-110415818	0.2119	53.3078
2	1A	AX-111216517	0.2119	53.3078
2	1A	AX-109303792	0.2119	53.3078
2	1A	AX-111011268	0.2119	53.3078
2	1A	AX-111473072	0.2119	53.3078
2	1A	AX-108978389	0.2119	53.3078
2	1A	AX-110031958	0.2119	53.3078
2	1A	AX-108857594	0.2119	53.3078
2	1A	AX-109858318	0.2119	53.3078
2	1A	AX-110942359	0.2119	53.3078
2	1A	AX-110918237	0.2119	53.3078
2	1A	AX-109016994	0.2119	53.3078
2	1A	AX-111675358	0.2119	53.3078
2	1A	AX-108731995	0.2119	53.3078
2	1A	AX-109823197	0.2119	53.3078
2	1A	AX-111114477	0.2119	53.3078
2	1A	AX-110050378	0.2119	53.3078
2	1A	AX-86171814	0.2119	53.3078
2	1A	AX-111080403	0.2119	53.3078
2	1A	AX-111492139	0.2119	53.3078
2	1A	AX-110125325	0.4237	53.7316
2	1A	AX-108739320	0.4237	53.7316
2	1A	AX-109469627	0.2119	53.9435
2	1A	AX-109447158	0.2119	53.9435
2	1A	AX-109031299	0.2119	53.9435
2	1A	AX-109833636	0.211	54.1544
2	1A	AX-109491193	0.211	54.1544
2	1A	AX-110371895	0.211	54.1544
2	1A	AX-110503827	0.2101	54.3645
2	1A	AX-111120791	0.2101	54.3645
2	1A	AX-109358415	0.2101	54.3645

2	1A	AX-110441035	0.2101	54.3645
2	1A	AX-110991760	0.2101	54.3645
2	1A	AX-110418193	0.2101	54.3645
2	1A	AX-110499408	0.2101	54.3645
2	1A	AX-108765611	0.2101	54.3645
2	1A	AX-110911382	0.2101	54.3645
2	1A	AX-108897448	0.2101	54.3645
2	1A	AX-111803078	0.2101	54.3645
2	1A	AX-111676715	0.2101	54.3645
2	1A	AX-110932807	0.2101	54.3645
2	1A	AX-109565465	0.2101	54.3645
2	1A	AX-108828312	0.2101	54.3645
2	1A	AX-110976553	0.2101	54.3645
2	1A	AX-111157391	0.2101	54.3645
2	1A	AX-108976512	0.2101	54.3645
2	1A	AX-109516730	0.2101	54.3645
2	1A	AX-109336866	0.2101	54.3645
2	1A	AX-110463408	0.2101	54.3645
2	1A	AX-109365768	0.2101	54.3645
2	1A	AX-109838442	0.2101	54.3645
2	1A	AX-110558693	0.2101	54.3645
2	1A	AX-111457419	0.4255	54.7901
2	1A	AX-111767357	0.2119	55.0019
2	1A	AX-108727664	0.8548	55.8567
2	1A	AX-110461795	0.8548	55.8567
2	1A	AX-111455992	0.8548	55.8567
2	1A	AX-109039717	0.8548	55.8567
2	1A	AX-110408723	0.8548	55.8567
2	1A	AX-111005213	0.8548	55.8567
2	1A	AX-108728517	0.8548	55.8567
2	1A	AX-110462732	0.8548	55.8567
2	1A	AX-111687984	0.8548	55.8567
2	1A	AX-109528407	0.2119	56.0686
2	1A	AX-109375456	0.4292	56.4978
2	1A	AX-109852030	0.2128	56.7105
2	1A	AX-108734257	1.5289	58.2394
2	1A	AX-110992831	1.5289	58.2394
2	1A	AX-110463986	0.2119	58.4513
2	1A	AX-109882536	0.211	58.6622
2	1A	AX-109828155	0.211	58.6622
2	1A	AX-108726330	0.211	58.6622
2	1A	AX-109968969	0.211	58.6622
2	1A	AX-110657398	0.4255	59.0878
2	1A	AX-111143912	0.4255	59.0878

2	1A	AX-109499408	0.211	59.2987
2	1A	AX-110944794	0.211	59.2987
2	1A	AX-109384637	0.211	59.2987
2	1A	AX-110028168	0.211	59.2987
2	1A	AX-108975356	0.211	59.2987
2	1A	AX-111047177	0.211	59.2987
2	1A	AX-111700821	0.211	59.5097
2	1A	AX-111022844	0.211	59.5097
2	1A	AX-109290544	0.211	59.5097
2	1A	AX-109848736	0.211	59.5097
2	1A	AX-110912803	0.211	59.5097
2	1A	AX-110557242	0.211	59.5097
2	1A	AX-111235717	0.211	59.5097
2	1A	AX-109330936	0.4237	59.9335
2	1A	AX-109848476	0.4237	59.9335
2	1A	AX-111776783	0.4237	59.9335
2	1A	AX-110424311	0.4237	59.9335
2	1A	AX-111484345	0.4237	59.9335
2	1A	AX-111135532	0.2119	60.1453
2	1A	AX-111504407	0.4274	60.5727
2	1A	AX-111649582	0.4274	60.5727
2	1A	AX-110675424	0.4274	60.5727
2	1A	AX-109056654	0.4274	60.5727
2	1A	AX-110014194	0.4274	60.5727
2	1A	AX-111676237	0.4274	60.5727
2	1A	AX-109999760	0.4274	60.5727
2	1A	AX-108783388	0.4274	60.5727
2	1A	AX-108847778	0.4274	60.5727
2	1A	AX-111114355	0.4274	60.5727
2	1A	AX-111454665	0.4274	60.5727
2	1A	AX-110382794	0.4274	60.5727
2	1A	AX-108835098	0.4274	60.5727
2	1A	AX-109522064	0.4274	60.5727
2	1A	AX-110483420	0.4274	60.5727
2	1A	AX-110957642	0.4274	60.5727
2	1A	AX-110920753	0.4274	60.5727
2	1A	AX-110499917	0.4274	60.5727
2	1A	AX-110674286	0.4274	60.5727
2	1A	AX-108944057	0.4274	60.5727
2	1A	AX-109949764	0.4274	60.5727
2	1A	AX-111481325	0.4274	60.5727
2	1A	AX-109963328	0.4274	60.5727
2	1A	AX-110416222	0.4274	60.5727
2	1A	AX-110997297	0.4274	60.5727

2	1A	AX-108748448	0.4274	60.5727
2	1A	AX-110474826	0.4274	60.5727
2	1A	AX-108766125	0.4274	60.5727
2	1A	AX-109398720	0.4274	60.5727
2	1A	AX-111620880	0.4274	60.5727
2	1A	AX-110637955	0.4274	60.5727
2	1A	AX-111740931	0.4274	60.5727
2	1A	AX-109331040	0.4274	60.5727
2	1A	AX-110046566	0.4274	60.5727
2	1A	AX-109961066	0.4274	60.5727
2	1A	AX-89527966	0.4274	60.5727
2	1A	AX-111650121	0.4274	60.5727
2	1A	AX-111600781	0.4274	60.5727
2	1A	AX-109386906	0.4274	60.5727
2	1A	AX-109491425	0.4274	60.5727
2	1A	AX-111802924	0.4274	60.5727
2	1A	AX-108831269	0.4274	60.5727
2	1A	AX-110433058	0.4274	60.5727
2	1A	AX-109322511	0.4274	60.5727
2	1A	AX-108917595	0.4274	60.5727
2	1A	AX-109290925	0.4274	60.5727
2	1A	AX-110501385	0.4274	61.0001
2	1A	AX-109894388	0.4274	61.0001
2	1A	AX-111607774	0.4274	61.0001
2	1A	AX-110474000	0.4274	61.0001
2	1A	AX-111275530	0.4274	61.0001
2	1A	AX-109886918	0.4274	61.0001
2	1A	AX-110044422	0.4274	61.0001
2	1A	AX-110151688	0.4274	61.0001
2	1A	AX-108789837	0.4274	61.0001
2	1A	AX-109055961	0.4274	61.0001
2	1A	AX-109832349	0.4274	61.0001
2	1A	AX-111646264	0.4274	61.0001
2	1A	AX-111055796	0.4274	61.0001
2	1A	AX-109291490	0.4274	61.0001
2	1A	AX-110431367	0.4274	61.0001
2	1A	AX-108731805	0.4274	61.0001
2	1A	AX-110049856	0.4274	61.0001
2	1A	AX-109518287	0.4274	61.0001
2	1A	AX-110577861	0.4274	61.0001
2	1A	AX-111562602	0.4274	61.0001
2	1A	AX-111594045	0.4274	61.0001
2	1A	AX-111247955	0.4274	61.0001
2	1A	AX-111664657	0.4274	61.0001

2	1A	AX-108974223	0.4255	61.4256
2	1A	AX-108842413	0.4255	61.4256
2	1A	AX-109654913	0.4237	61.8493
2	1A	AX-110972166	0.4237	61.8493
2	1A	AX-109313554	0.4237	61.8493
2	1A	AX-110926703	0.4237	61.8493
2	1A	AX-110421360	0.4237	61.8493
2	1A	AX-111104839	0.4237	61.8493
2	1A	AX-110669842	0.4237	61.8493
2	1A	AX-109924427	0.4237	61.8493
2	1A	AX-110548190	0.4237	61.8493
2	1A	AX-109932769	0.4237	61.8493
2	1A	AX-109289149	0.4237	61.8493
2	1A	AX-111185754	0.4237	61.8493
2	1A	AX-110425174	0.4237	61.8493
2	1A	AX-110531680	0.4237	61.8493
2	1A	AX-110920834	0.4237	61.8493
2	1A	AX-110142052	0.4237	61.8493
2	1A	AX-108742844	0.4237	61.8493
2	1A	AX-108867136	0.4237	61.8493
2	1A	AX-109492416	0.4237	61.8493
2	1A	AX-111069267	0.4237	61.8493
2	1A	AX-111504145	0.4237	61.8493
2	1A	AX-110062154	0.4237	61.8493
2	1A	AX-110548430	0.4237	61.8493
2	1A	AX-111835596	0.4237	61.8493
2	1A	AX-110098461	0.4237	61.8493
2	1A	AX-108726928	0.4237	61.8493
2	1A	AX-108913710	0.4237	61.8493
2	1A	AX-110535560	0.4237	61.8493
2	1A	AX-109404607	0.4237	61.8493
2	1A	AX-110622943	0.4237	61.8493
2	1A	AX-108882911	0.4237	61.8493
2	1A	AX-109274812	0.6356	62.485
2	1A	AX-109888951	0.6356	62.485
2	1A	AX-108969237	0.6356	62.485
2	1A	AX-108790622	0.6383	63.1233
2	1A	AX-110421994	0.6383	63.1233
2	1A	AX-111524059	0.6383	63.1233
2	1A	AX-111643857	0.6383	63.1233
2	1A	AX-111620282	0.6383	63.1233
2	1A	AX-109454021	0.6383	63.1233
2	1A	AX-109964270	0.6383	63.1233
2	1A	AX-108881024	0.6383	63.1233

2	1A	AX-109351333	0.6383	63.1233
2	1A	AX-109365980	0.6383	63.1233
2	1A	AX-111835313	0.6383	63.1233
2	1A	AX-110925138	0.6383	63.1233
2	1A	AX-111092912	0.6383	63.1233
2	1A	AX-110366134	0.6383	63.1233
2	1A	AX-111501752	0.6383	63.1233
2	1A	AX-109335303	0.6383	63.1233
2	1A	AX-89412085	0.6383	63.1233
2	1A	AX-110364500	0.6383	63.1233
2	1A	AX-110044069	0.6383	63.1233
2	1A	AX-109903085	0.6383	63.1233
2	1A	AX-110527550	0.6383	63.1233
2	1A	AX-109882371	0.6383	63.1233
2	1A	AX-108898845	0.6383	63.1233
2	1A	AX-111671181	0.6383	63.1233
2	1A	AX-109869264	0.6383	63.1233
2	1A	AX-111031603	0.6383	63.1233
2	1A	AX-109870507	0.6383	63.1233
2	1A	AX-109412346	0.6383	63.1233
2	1A	AX-109421152	0.6383	63.1233
2	1A	AX-110485750	0.6383	63.1233
2	1A	AX-94618546	0.6383	63.1233
2	1A	AX-110430171	0.211	63.3343
2	1A	AX-108920635	0.211	63.3343
2	1A	AX-110370624	0.211	63.3343
2	1A	AX-109421021	0.211	63.3343
2	1A	AX-110565611	0.211	63.3343
2	1A	AX-110945419	0.211	63.3343
2	1A	AX-109321208	0.211	63.3343
2	1A	AX-110172267	0.211	63.3343
2	1A	AX-109465131	0.211	63.3343
2	1A	AX-109448640	0.211	63.3343
2	1A	AX-110592362	0.211	63.3343
2	1A	AX-110057101	0.211	63.3343
2	1A	AX-109388069	0.211	63.3343
2	1A	AX-109062393	0.211	63.3343
2	1A	AX-109323832	0.211	63.3343
2	1A	AX-111552141	0.2101	63.5444
2	1A	AX-110424576	0.2101	63.5444
2	1A	AX-109345385	0.2101	63.5444
2	1A	AX-111489584	0.2101	63.5444
2	1A	AX-109894911	0.2101	63.7544
2	1A	AX-109864955	0.2101	63.7544

2	1A	AX-109940978	0.2101	63.7544
2	1A	AX-108906570	0.2101	63.7544
2	1A	AX-109626482	0.2101	63.7544
2	1A	AX-110044222	0.2101	63.7544
2	1A	AX-110983052	0.2101	63.7544
2	1A	AX-108801005	0.2101	63.7544
2	1A	AX-111582185	0.2101	63.7544
2	1A	AX-111130454	0.2101	63.7544
2	1A	AX-111140737	0.2101	63.7544
2	1A	AX-110911310	0.2101	63.7544
2	1A	AX-111693554	0.2101	63.7544
2	1A	AX-110905824	0.2101	63.7544
2	1A	AX-110580127	0.2101	63.7544
2	1A	AX-109320444	0.4255	64.18
2	1A	AX-108867098	0.4255	64.18
2	1A	AX-111734804	0.4255	64.18
2	1A	AX-109822323	0.4255	64.18
2	1A	AX-110121885	0.4255	64.18
2	1A	AX-109877312	0.4255	64.18
2	1A	AX-109941876	0.4255	64.18
2	1A	AX-111611028	0.4255	64.18
2	1A	AX-110595621	0.4255	64.18
2	1A	AX-110989752	0.4255	64.18
2	1A	AX-111101980	0.4255	64.18
2	1A	AX-109514069	0.4255	64.18
2	1A	AX-111060515	0.4255	64.18
2	1A	AX-110671993	0.4255	64.18
2	1A	AX-109354879	0.4255	64.18
2	1A	AX-110943566	0.4255	64.18
2	1A	AX-110388843	0.4255	64.18
2	1A	AX-86172240	0.4255	64.18
2	1A	AX-111106193	0.4255	64.18
2	1A	AX-111757297	0.4255	64.18
2	1A	AX-109417723	0.4255	64.18
2	1A	AX-111451910	0.4255	64.18
2	1A	AX-109048694	0.4255	64.18
2	1A	AX-110127899	0.4255	64.18
2	1A	AX-111792975	0.4255	64.18
2	1A	AX-110363483	0.4255	64.18
2	1A	AX-109368433	0.4255	64.18
2	1A	AX-110412551	0.4255	64.18
2	1A	AX-110498098	0.4255	64.18
2	1A	AX-111231878	0.4255	64.18
2	1A	AX-109852682	0.2101	64.3901

2	1A	AX-108914408	0.2101	64.3901
2	1A	AX-111118068	0.2101	64.3901
2	1A	AX-110603804	0.2101	64.3901
2	1A	AX-110447358	0.2101	64.3901
2	1A	AX-111465033	0.2101	64.3901
2	1A	AX-108814432	0.2101	64.3901
2	1A	AX-110954399	0.2101	64.3901
2	1A	AX-111131081	0.2101	64.3901
2	1A	AX-110611419	0.2101	64.3901
2	1A	AX-109905829	0.2101	64.3901
2	1A	AX-109377040	0.2101	64.3901
2	1A	AX-111587201	0.2101	64.3901
2	1A	AX-110910072	0.2101	64.3901
2	1A	AX-111590181	0.2101	64.3901
2	1A	AX-110037641	0.2101	64.3901
2	1A	AX-108765324	0.2101	64.3901
2	1A	AX-109827001	0.2101	64.6001
2	1A	AX-110540781	0.2101	64.6001
2	1A	AX-110122232	0.2101	64.6001
2	1A	AX-111600398	0.2101	64.6001
2	1A	AX-110016390	0.2101	64.6001
2	1A	AX-110513734	0.2101	64.6001
2	1A	AX-110058285	0.2101	64.6001
2	1A	AX-109269923	0.2101	64.6001
2	1A	AX-111523929	0.2101	64.6001
2	1A	AX-109299945	0.2101	64.8102
2	1A	AX-110076355	0.2101	64.8102
2	1A	AX-109904229	0.2101	64.8102
2	1A	AX-111522447	0.2101	64.8102
2	1A	AX-111149806	0.2101	64.8102
2	1A	AX-109986210	0.4237	65.234
2	1A	AX-111160657	0.2119	65.4458
2	1A	AX-109902402	0.2146	65.6604
2	1A	AX-111616300	1.0919	66.7523
2	1A	AX-108738272	1.0919	66.7523
2	1A	AX-111045651	0.4274	67.1797
2	1A	AX-109281194	0.4274	67.1797
2	1A	AX-109355072	0.2119	67.3915
2	1A	AX-108978418	0.2128	67.6043
2	1A	AX-110995984	1.9922	69.5965
2	1A	AX-111453167	0.6697	70.2662
2	1A	AX-111539408	0.4505	70.7167
2	1A	AX-109425297	0.431	71.1477
2	1A	AX-111761395	0.431	71.1477

2	1A	AX-110026141	0.431	71.1477
2	1A	AX-109937019	0.431	71.1477
2	1A	AX-111511867	0.431	71.1477
2	1A	AX-110122042	0.431	71.1477
2	1A	AX-110049263	0.431	71.1477
2	1A	AX-110145411	0.431	71.1477
2	1A	AX-109938803	0.431	71.1477
2	1A	AX-109325378	0.431	71.1477
2	1A	AX-111064559	0.8622	72.0098
2	1A	AX-108782680	0.2137	72.2235
2	1A	AX-109408386	0.4274	72.6509
2	1A	AX-109105167	0.4274	72.6509
2	1A	AX-109863129	0.4274	73.0782
2	1A	AX-111452805	0.4292	73.5074
2	1A	AX-110980250	0.4292	73.5074
2	1A	AX-109982571	0.4292	73.5074
2	1A	AX-110379520	2.4684	75.9758
2	1A	AX-109338114	1.1064	77.0822
2	1A	AX-109580770	1.1064	77.0822
2	1A	AX-109957442	1.1064	77.0822
2	1A	AX-109923808	1.1064	77.0822
2	1A	AX-111456614	0.4484	77.5306
2	1A	AX-109903014	0.4405	77.9712
2	1A	AX-111653348	0.4405	77.9712
2	1A	AX-108740522	0.4405	77.9712
2	1A	AX-110472708	0.4405	77.9712
2	1A	AX-110472728	1.0919	79.063
2	1A	AX-110142186	0.2146	79.2776
2	1A	AX-110451271	0.2146	79.2776
2	1A	AX-108880253	0.2146	79.2776
2	1A	AX-109428760	3.907	83.1846
2	1A	AX-111654095	3.907	83.1846
2	1A	AX-94732625	3.907	83.1846
2	1A	AX-111683984	2.01	85.1946
2	1A	AX-109912468	2.01	85.1946
2	1A	AX-109869791	2.01	85.1946
2	1A	AX-111474637	0.2137	85.4083
2	1A	AX-111213448	0.2137	85.4083
2	1A	AX-109447536	0.2137	85.4083
2	1A	AX-89373031	0.2137	85.4083
2	1A	AX-110414542	0.2137	85.4083
2	1A	AX-108767286	0.2137	85.4083
2	1A	AX-111571300	0.2137	85.4083
2	1A	AX-111065765	0.2137	85.4083

2	1A	AX-111573424	0.2137	85.4083
2	1A	AX-108957555	0.2137	85.4083
2	1A	AX-110044439	0.2137	85.4083
2	1A	AX-111043857	0.2137	85.4083
2	1A	AX-109861530	0.6411	86.0494
2	1A	AX-109506550	0.6411	86.0494
2	1A	AX-110937452	0.2137	86.2631
2	1A	AX-109473031	0.4292	86.6923
2	1A	AX-89427288	0.6466	87.3388
2	1A	AX-110991294	0.4292	87.768
2	1A	AX-110072313	0.8585	88.6265
2	1A	AX-111490895	0.8585	88.6265
2	1A	AX-111099322	0.8585	88.6265
2	1A	AX-110378928	0.8585	88.6265
2	1A	AX-111669740	0.8585	88.6265
2	1A	AX-95659890	0.8585	88.6265
2	1A	AX-109978883	0.8585	88.6265
3	1B	AX-111760595	0	0
3	1B	AX-108899056	0.2128	0.2128
3	1B	AX-110492182	0.2128	0.2128
3	1B	AX-110558071	0.2128	0.2128
3	1B	AX-111564577	0.2128	0.2128
3	1B	AX-109997234	0.2128	0.2128
3	1B	AX-108879617	0.2128	0.2128
3	1B	AX-109279049	0.2128	0.2128
3	1B	AX-108765481	0.2128	0.2128
3	1B	AX-111544717	0.2128	0.2128
3	1B	AX-86174914	0.2128	0.2128
3	1B	AX-109491181	0.2128	0.2128
3	1B	AX-110076399	0.2128	0.2128
3	1B	AX-108842989	0.2128	0.2128
3	1B	AX-111666373	0.2128	0.2128
3	1B	AX-109452517	0.2128	0.2128
3	1B	AX-109326464	0.2128	0.2128
3	1B	AX-109938889	0.2128	0.2128
3	1B	AX-109031582	0.2128	0.2128
3	1B	AX-111488672	0.2128	0.2128
3	1B	AX-109349342	0.2128	0.2128
3	1B	AX-109331081	0.2128	0.2128
3	1B	AX-109898583	0.2128	0.2128
3	1B	AX-108735424	0.2128	0.2128
3	1B	AX-110411189	0.2128	0.2128
3	1B	AX-94508976	0.2128	0.2128
3	1B	AX-111144554	0.2128	0.2128

3	1B	AX-109830149	0.2128	0.2128
3	1B	AX-109392936	0.2128	0.2128
3	1B	AX-109508833	0.2128	0.2128
3	1B	AX-109577331	0.2128	0.2128
3	1B	AX-110091847	0.2128	0.2128
3	1B	AX-110029221	0.2128	0.2128
3	1B	AX-108775647	0.2128	0.2128
3	1B	AX-95180100	0.2128	0.2128
3	1B	AX-110078176	0.2049	0.4177
3	1B	AX-110607797	0.2049	0.4177
3	1B	AX-110087213	0.2049	0.4177
3	1B	AX-109539628	0.2049	0.4177
3	1B	AX-111092429	0.2049	0.4177
3	1B	AX-111122427	0.2049	0.4177
3	1B	AX-111505048	0.2049	0.4177
3	1B	AX-110465097	0.2049	0.4177
3	1B	AX-110040017	0.2049	0.4177
3	1B	AX-111449599	0.2049	0.4177
3	1B	AX-111526936	0.625	1.0427
3	1B	AX-108891924	0.6276	1.6704
3	1B	AX-109890613	0.8404	2.5108
3	1B	AX-94678835	0.8404	2.5108
3	1B	AX-108915846	0.2066	2.7174
3	1B	AX-109414199	0.2066	2.7174
3	1B	AX-110409346	0.2066	2.7174
3	1B	AX-110905431	0.2066	2.7174
3	1B	AX-111526060	0.2066	2.7174
3	1B	AX-109009142	0.2066	2.7174
3	1B	AX-109563613	0.2058	2.9232
3	1B	AX-86175499	0.2058	2.9232
3	1B	AX-109822203	0.6224	3.5456
3	1B	AX-109577957	0.2049	3.7505
3	1B	AX-111530524	0.2049	3.7505
3	1B	AX-110477589	0.2049	3.7505
3	1B	AX-111007044	0.2049	3.7505
3	1B	AX-110966427	0.2049	3.7505
3	1B	AX-111450171	0.2049	3.7505
3	1B	AX-110929557	0.2049	3.7505
3	1B	AX-95253310	0.2049	3.7505
3	1B	AX-109456390	1.2608	5.0113
3	1B	AX-110067372	1.2608	5.0113
3	1B	AX-89635557	1.2608	5.0113
3	1B	AX-111083588	0.4149	5.4262
3	1B	AX-86165622	0.4149	5.4262

3	1B	AX-110378973	0.2058	5.632
3	1B	AX-89694598	0.2058	5.632
3	1B	AX-109281682	0.2058	5.632
3	1B	AX-110964175	0.2058	5.632
3	1B	AX-109457469	0.2058	5.632
3	1B	AX-110044233	0.2058	5.632
3	1B	AX-111467480	0.2058	5.632
3	1B	AX-111734583	0.2058	5.632
3	1B	AX-109337640	0.2058	5.632
3	1B	AX-109396077	3.2798	8.9118
3	1B	AX-111546976	3.2798	8.9118
3	1B	AX-108984254	3.2798	8.9118
3	1B	AX-109534834	3.2798	8.9118
3	1B	AX-110921713	3.2798	8.9118
3	1B	AX-111044282	3.2798	8.9118
3	1B	AX-111671430	0.4132	9.325
3	1B	AX-111484814	0.4132	9.325
3	1B	AX-111669834	0.4132	9.325
3	1B	AX-110437780	0.4132	9.325
3	1B	AX-111525817	0.4132	9.325
3	1B	AX-109932845	0.4132	9.325
3	1B	AX-111504759	0.4132	9.325
3	1B	AX-111625145	0.4132	9.325
3	1B	AX-94958010	0.4132	9.325
3	1B	AX-111545897	0.2049	9.53
3	1B	AX-109882902	0.2049	9.53
3	1B	AX-111093824	0.2049	9.53
3	1B	AX-108882565	0.2049	9.53
3	1B	AX-111625425	0.2049	9.53
3	1B	AX-110572952	0.2049	9.53
3	1B	AX-110908967	0.2049	9.53
3	1B	AX-110006862	0.2049	9.53
3	1B	AX-110361304	0.2049	9.53
3	1B	AX-109429493	0.2049	9.53
3	1B	AX-108963825	0.2049	9.53
3	1B	AX-111061140	0.2049	9.53
3	1B	AX-110494639	0.2049	9.53
3	1B	AX-109306193	0.2049	9.53
3	1B	AX-86174278	0.2049	9.53
3	1B	AX-109817665	0.6199	10.1498
3	1B	AX-110477894	0.6199	10.1498
3	1B	AX-109546448	0.6199	10.1498
3	1B	AX-110923640	0.6199	10.1498
3	1B	AX-109433305	0.6199	10.1498

3	1B	AX-110519906	0.6199	10.1498
3	1B	AX-109868871	0.6199	10.1498
3	1B	AX-111745798	0.2049	10.3547
3	1B	AX-108772985	0.2058	10.5605
3	1B	AX-110564293	0.2058	10.5605
3	1B	AX-108828473	0.2058	10.5605
3	1B	AX-110910638	0.2058	10.7663
3	1B	AX-89553276	1.0462	11.8125
3	1B	AX-109073020	0.4132	12.2257
3	1B	AX-109906095	0.4132	12.2257
3	1B	AX-111135292	0.4132	12.2257
3	1B	AX-111647519	0.4132	12.2257
3	1B	AX-109280812	0.4132	12.2257
3	1B	AX-110546816	0.4132	12.2257
3	1B	AX-111568828	0.4132	12.2257
3	1B	AX-108932276	0.4132	12.2257
3	1B	AX-109577777	0.4132	12.2257
3	1B	AX-109372727	1.0375	13.2632
3	1B	AX-111178072	1.0375	13.2632
3	1B	AX-109878201	1.0375	13.2632
3	1B	AX-109993757	1.0375	13.2632
3	1B	AX-111654753	1.0375	13.2632
3	1B	AX-109483095	1.0375	13.2632
3	1B	AX-109430801	1.0375	13.2632
3	1B	AX-110495582	1.0375	13.2632
3	1B	AX-109359622	1.0375	13.2632
3	1B	AX-109434543	1.0375	13.2632
3	1B	AX-109294196	1.0375	13.2632
3	1B	AX-89370077	1.0375	13.2632
3	1B	AX-108881179	0.2041	13.4673
3	1B	AX-110402993	0.2041	13.4673
3	1B	AX-89662088	0.2041	13.4673
3	1B	AX-110696827	0.4098	13.8771
3	1B	AX-111801574	0.4098	13.8771
3	1B	AX-108745211	0.4098	13.8771
3	1B	AX-111632020	0.4098	13.8771
3	1B	AX-111108496	0.4098	13.8771
3	1B	AX-110575267	0.4098	13.8771
3	1B	AX-111081842	0.4098	13.8771
3	1B	AX-109818908	0.4098	13.8771
3	1B	AX-110975549	0.4098	13.8771
3	1B	AX-109866120	0.4098	13.8771
3	1B	AX-111689186	0.4098	13.8771
3	1B	AX-110526093	0.4098	13.8771

3	1B	AX-109821945	0.4098	13.8771
3	1B	AX-111595814	2.3623	16.2394
3	1B	AX-111011700	2.3623	16.2394
3	1B	AX-110965170	2.3623	16.2394
3	1B	AX-109353940	2.3623	16.2394
3	1B	AX-109850741	2.3623	16.2394
3	1B	AX-109394270	2.3623	16.2394
3	1B	AX-109095584	0.4132	16.6526
3	1B	AX-110922932	0.4132	16.6526
3	1B	AX-111452123	0.4132	16.6526
3	1B	AX-110104013	0.2058	16.8584
3	1B	AX-109407440	0.2058	16.8584
3	1B	AX-109948353	0.2058	17.0642
3	1B	AX-111080509	0.2058	17.0642
3	1B	AX-109449046	0.2058	17.0642
3	1B	AX-109284801	0.2058	17.0642
3	1B	AX-110569213	0.2058	17.0642
3	1B	AX-111062860	0.2058	17.0642
3	1B	AX-110530323	0.2058	17.0642
3	1B	AX-110472942	0.2058	17.0642
3	1B	AX-110977076	0.2058	17.0642
3	1B	AX-109508345	0.4115	17.4757
3	1B	AX-111635387	0.4115	17.4757
3	1B	AX-111215884	0.4115	17.4757
3	1B	AX-111126225	0.4115	17.4757
3	1B	AX-108922026	0.4115	17.4757
3	1B	AX-109870057	0.4115	17.4757
3	1B	AX-109004107	0.4115	17.4757
3	1B	AX-111825076	0.2058	17.6814
3	1B	AX-110915806	0.2058	17.6814
3	1B	AX-110997259	0.2066	17.8881
3	1B	AX-109821715	0.2066	17.8881
3	1B	AX-111592733	0.2066	17.8881
3	1B	AX-110552945	0.2066	17.8881
3	1B	AX-111696684	0.2066	17.8881
3	1B	AX-110366371	0.2066	17.8881
3	1B	AX-111578414	0.2066	17.8881
3	1B	AX-111558163	0.2066	17.8881
3	1B	AX-109848568	0.4132	18.3013
3	1B	AX-111780264	0.4132	18.3013
3	1B	AX-110989879	0.4132	18.3013
3	1B	AX-110018927	0.4132	18.3013
3	1B	AX-109916275	0.4132	18.3013
3	1B	AX-108987978	0.4132	18.3013

3	1B	AX-110453549	0.4132	18.3013
3	1B	AX-111833715	0.4132	18.3013
3	1B	AX-109972860	0.4132	18.3013
3	1B	AX-108894131	0.4132	18.3013
3	1B	AX-109301565	0.4132	18.3013
3	1B	AX-111576821	0.4132	18.3013
3	1B	AX-111641463	0.4132	18.3013
3	1B	AX-109980293	0.4132	18.3013
3	1B	AX-110507994	0.4132	18.3013
3	1B	AX-109928923	0.4132	18.3013
3	1B	AX-111498058	0.4132	18.3013
3	1B	AX-110922826	0.4132	18.3013
3	1B	AX-109832158	0.4132	18.3013
3	1B	AX-108772739	0.4132	18.3013
3	1B	AX-108904194	0.4132	18.3013
3	1B	AX-110974122	0.4132	18.3013
3	1B	AX-110409210	0.4132	18.3013
3	1B	AX-110531613	0.4132	18.3013
3	1B	AX-111778997	0.4132	18.3013
3	1B	AX-109862005	0.4132	18.3013
3	1B	AX-108824644	0.4132	18.3013
3	1B	AX-110091358	0.4132	18.3013
3	1B	AX-89403195	0.4132	18.3013
3	1B	AX-111104674	0.4132	18.3013
3	1B	AX-111588383	0.4132	18.3013
3	1B	AX-110608451	0.4132	18.3013
3	1B	AX-109463291	0.4132	18.3013
3	1B	AX-108822354	0.4132	18.3013
3	1B	AX-110425403	0.4132	18.3013
3	1B	AX-109491892	0.4132	18.3013
3	1B	AX-108826064	0.4132	18.3013
3	1B	AX-110382473	0.4132	18.3013
3	1B	AX-111042449	0.4132	18.3013
3	1B	AX-111569277	0.4132	18.3013
3	1B	AX-109461410	0.4132	18.3013
3	1B	AX-109403182	0.4132	18.3013
3	1B	AX-110067443	0.4132	18.3013
3	1B	AX-94631200	0.4132	18.3013
3	1B	AX-109345781	0.4132	18.3013
3	1B	AX-86162453	0.4132	18.3013
3	1B	AX-108832305	0.4149	18.7162
3	1B	AX-94620168	0.4149	18.7162
3	1B	AX-110476820	0.625	19.3413
3	1B	AX-109379070	0.625	19.3413

3	1B	AX-109875834	0.625	19.3413
3	1B	AX-111506075	0.2058	19.547
3	1B	AX-110375123	0.2058	19.547
3	1B	AX-108792338	5.8404	25.3874
3	1B	AX-111129114	5.8404	25.3874
3	1B	AX-111585122	5.8404	25.3874
3	1B	AX-111057322	5.8404	25.3874
3	1B	AX-110435033	5.8404	25.3874
3	1B	AX-109992552	5.8404	25.3874
3	1B	AX-110976797	5.8404	25.3874
3	1B	AX-111100199	5.8404	25.3874
3	1B	AX-108915108	2.1753	27.5627
3	1B	AX-111457783	2.1753	27.5627
3	1B	AX-111576817	2.1753	27.5627
3	1B	AX-111078071	2.1753	27.5627
3	1B	AX-111508984	2.1753	27.5627
3	1B	AX-109309829	2.1753	27.5627
3	1B	AX-108977901	2.1753	27.5627
3	1B	AX-109490479	2.1753	27.5627
3	1B	AX-109933153	2.1753	27.5627
3	1B	AX-108920629	0.4202	27.9829
3	1B	AX-109583602	1.0685	29.0514
3	1B	AX-111220018	0.8511	29.9026
3	1B	AX-110511453	0.8511	29.9026
3	1B	AX-109302722	0.2092	30.1118
3	1B	AX-111528969	0.2092	30.1118
3	1B	AX-110442183	0.2092	30.1118
3	1B	AX-111491365	0.2092	30.1118
3	1B	AX-110400085	0.2092	30.1118
3	1B	AX-111545752	0.2092	30.1118
3	1B	AX-110486286	0.2092	30.1118
3	1B	AX-109831683	0.2092	30.1118
3	1B	AX-110464799	0.2092	30.1118
3	1B	AX-110486693	0.2092	30.1118
3	1B	AX-110042774	0.2092	30.1118
3	1B	AX-110940226	0.2092	30.1118
3	1B	AX-111159680	0.2092	30.1118
3	1B	AX-110617079	0.2092	30.1118
3	1B	AX-108859006	0.2092	30.1118
3	1B	AX-108806485	0.2092	30.1118
3	1B	AX-111683643	0.2092	30.1118
3	1B	AX-94751515	0.2092	30.1118
3	1B	AX-109276656	0.2092	30.321
3	1B	AX-108898290	0.2092	30.321

3	1B	AX-111220052	0.2092	30.321
3	1B	AX-110517044	0.2092	30.321
3	1B	AX-110575668	0.2092	30.321
3	1B	AX-108880548	0.2092	30.321
3	1B	AX-89332288	0.2092	30.321
3	1B	AX-111561231	0.2092	30.321
3	1B	AX-108867872	0.2092	30.321
3	1B	AX-110050947	0.2092	30.321
3	1B	AX-94464561	0.2092	30.321
3	1B	AX-110691903	0.2092	30.321
3	1B	AX-110553901	0.211	30.532
3	1B	AX-110968810	0.211	30.532
3	1B	AX-110555680	0.4237	30.9557
3	1B	AX-108793067	5.1342	36.0899
3	1B	AX-110406843	5.1342	36.0899
3	1B	AX-111121168	0.4237	36.5137
3	1B	AX-108864427	2.6692	39.1829
3	1B	AX-110581950	1.0685	40.2514
3	1B	AX-111717028	1.0685	40.2514
3	1B	AX-111042529	1.0685	40.2514
3	1B	AX-109971975	1.0685	40.2514
3	1B	AX-109627132	1.0685	40.2514
3	1B	AX-111540680	0.6356	40.887
3	1B	AX-110585482	0.6356	40.887
3	1B	AX-111047000	0.6356	40.887
3	1B	AX-110947408	2.9051	43.7921
3	1B	AX-109872283	0.6411	44.4332
3	1B	AX-109509313	9.4545	53.8877
3	1B	AX-111548801	0.2101	54.0978
3	1B	AX-109917756	0.2128	54.3105
3	1B	AX-110828223	0.2128	54.3105
3	1B	AX-110017315	3.9618	58.2723
3	1B	AX-110972445	1.299	59.5713
3	1B	AX-111056129	0.2119	59.7832
3	1B	AX-110918909	4.1001	63.8832
3	1B	AX-109397003	4.1001	63.8832
3	1B	AX-89316446	4.1001	63.8832
3	1B	AX-111764501	4.1001	63.8832
3	1B	AX-108747070	4.1001	63.8832
3	1B	AX-110567254	4.1001	63.8832
3	1B	AX-109345177	4.1001	63.8832
3	1B	AX-111450994	4.1001	63.8832
3	1B	AX-111019759	4.1001	63.8832
3	1B	AX-109440891	5.527	69.4102

3	1B	AX-109389405	5.527	69.4102
3	1B	AX-89740962	5.527	69.4102
3	1B	AX-108930953	5.527	69.4102
3	1B	AX-109273019	5.527	69.4102
3	1B	AX-108800083	5.527	69.4102
3	1B	AX-94871279	5.527	69.4102
3	1B	AX-109576517	4.063	73.4732
3	1B	AX-110020417	0.4184	73.8916
3	1B	AX-108799967	1.7174	75.609
3	1B	AX-110634220	1.7174	75.609
3	1B	AX-109957126	1.7174	75.609
3	1B	AX-109840266	1.7174	75.609
3	1B	AX-109935558	1.7174	75.609
3	1B	AX-111519781	1.7174	75.609
3	1B	AX-110938295	0.8475	76.4566
3	1B	AX-110384584	0.8475	76.4566
3	1B	AX-111527355	0.8475	76.4566
3	1B	AX-110447268	0.8475	76.4566
3	1B	AX-109374477	0.8475	76.4566
3	1B	AX-109840531	0.2092	76.6658
3	1B	AX-109372356	0.2092	76.6658
3	1B	AX-108733712	0.4202	77.086
3	1B	AX-109423718	0.4202	77.086
3	1B	AX-109966248	0.6329	77.7189
3	1B	AX-109414218	0.6329	77.7189
3	1B	AX-108765712	0.4202	78.1391
3	1B	AX-109973619	0.8512	78.9902
3	1B	AX-111469159	0.8512	78.9902
3	1B	AX-109892648	0.4329	79.4231
3	1B	AX-110000934	4.2156	83.6387
3	1B	AX-109341064	0.2101	83.8488
3	1B	AX-109951074	0.2101	83.8488
3	1B	AX-111130335	0.2101	83.8488
3	1B	AX-111471952	0.2101	83.8488
3	1B	AX-111126182	5.1103	88.9591
4	1D	AX-111211980	0	0
4	1D	AX-108820920	0	0
4	1D	AX-110957998	17.4353	17.4353
4	1D	AX-108911284	17.4353	17.4353
4	1D	AX-108915656	17.4353	17.4353
4	1D	AX-109292164	0.4255	17.8608
4	1D	AX-109872778	0.6383	18.4992
4	1D	AX-111687074	0.6383	18.4992
4	1D	AX-111102983	0.6383	18.4992

4	1D	AX-109587657	6.6426	25.1417
4	1D	AX-109406252	6.6426	25.1417
4	1D	AX-111125663	6.6426	25.1417
4	1D	AX-109725508	6.6426	25.1417
4	1D	AX-109841616	0.2092	25.3509
4	1D	AX-111375978	0.2092	25.3509
4	1D	AX-109739785	0.2092	25.3509
4	1D	AX-108764560	0.2092	25.3509
4	1D	AX-110916476	0.2092	25.3509
4	1D	AX-109309330	0.2092	25.3509
4	1D	AX-110238045	0.2092	25.3509
4	1D	AX-110273474	0.2092	25.3509
4	1D	AX-109183884	0.2092	25.3509
4	1D	AX-109899731	0.2092	25.3509
4	1D	AX-111013786	5.446	30.7969
4	1D	AX-111991641	0.2119	31.0088
4	1D	AX-109439641	0.2119	31.0088
4	1D	AX-109277466	0.2119	31.0088
4	1D	AX-94538909	0.2119	31.0088
4	1D	AX-110455518	0.2119	31.0088
4	1D	AX-111266392	0.2119	31.0088
4	1D	AX-111136367	0.2119	31.0088
4	1D	AX-109453421	0.2119	31.0088
4	1D	AX-109305466	0.2119	31.0088
4	1D	AX-109845657	0.4237	31.4325
4	1D	AX-111048221	0.4237	31.4325
4	1D	AX-109829243	0.4237	31.4325
4	1D	AX-109316083	0.4237	31.4325
4	1D	AX-108953685	0.4237	31.4325
4	1D	AX-109323804	0.4237	31.4325
4	1D	AX-110556932	0.4237	31.4325
4	1D	AX-110436754	0.4237	31.4325
4	1D	AX-110028352	0.211	31.6435
4	1D	AX-110701482	0.211	31.6435
4	1D	AX-109488630	0.211	31.6435
4	1D	AX-108925722	0.211	31.6435
4	1D	AX-109279740	0.211	31.8545
4	1D	AX-110873417	0.211	31.8545
4	1D	AX-110989363	0.211	31.8545
4	1D	AX-111855341	0.211	31.8545
4	1D	AX-109833595	0.211	31.8545
4	1D	AX-110348693	0.211	31.8545
4	1D	AX-109778641	0.211	31.8545
4	1D	AX-110824207	0.211	31.8545

4	1D	AX-109928918	0.211	31.8545
4	1D	AX-108805297	0.211	31.8545
4	1D	AX-110225836	0.211	31.8545
4	1D	AX-109795899	0.211	31.8545
4	1D	AX-111521975	0.211	31.8545
4	1D	AX-110215330	0.211	31.8545
4	1D	AX-110498102	0.211	31.8545
4	1D	AX-111025183	0.211	31.8545
4	1D	AX-110001853	0.211	31.8545
4	1D	AX-109971637	0.211	31.8545
4	1D	AX-110642361	0.211	31.8545
4	1D	AX-111565824	0.211	31.8545
4	1D	AX-111023544	0.211	31.8545
4	1D	AX-110984456	0.211	31.8545
4	1D	AX-109798065	0.211	31.8545
4	1D	AX-110386333	0.211	31.8545
4	1D	AX-111692654	0.211	31.8545
4	1D	AX-109321591	0.211	31.8545
4	1D	AX-95107387	0.211	31.8545
4	1D	AX-110557220	0.211	31.8545
4	1D	AX-110938713	0.211	31.8545
4	1D	AX-110992265	0.211	31.8545
4	1D	AX-111415283	0.211	31.8545
4	1D	AX-110976835	0.211	31.8545
4	1D	AX-109458865	0.211	31.8545
4	1D	AX-111519811	0.211	31.8545
4	1D	AX-111466926	0.211	31.8545
4	1D	AX-109441496	0.211	31.8545
4	1D	AX-108739620	0.211	31.8545
4	1D	AX-111603924	0.211	31.8545
4	1D	AX-108890951	0.211	31.8545
4	1D	AX-111371086	0.211	31.8545
4	1D	AX-111464443	0.211	31.8545
4	1D	AX-109866317	0.211	31.8545
4	1D	AX-109816488	0.211	31.8545
4	1D	AX-109832828	0.211	31.8545
4	1D	AX-111041982	0.211	31.8545
4	1D	AX-111498518	0.211	31.8545
4	1D	AX-110057793	0.211	31.8545
4	1D	AX-108773848	0.211	31.8545
4	1D	AX-89690052	0.211	31.8545
4	1D	AX-110832928	0.211	31.8545
4	1D	AX-110829663	0.211	31.8545
4	1D	AX-109490030	0.211	31.8545

4	1D	AX-110272241	0.211	31.8545
4	1D	AX-110363643	0.211	31.8545
4	1D	AX-110893267	0.211	31.8545
4	1D	AX-111016039	0.211	31.8545
4	1D	AX-89672619	0.211	31.8545
4	1D	AX-110555637	0.211	32.0654
4	1D	AX-109276095	0.211	32.0654
4	1D	AX-111169843	0.211	32.0654
4	1D	AX-110689735	0.211	32.2764
4	1D	AX-109871815	0.211	32.2764
4	1D	AX-111520266	0.211	32.2764
4	1D	AX-108816871	0.211	32.2764
4	1D	AX-111512980	0.211	32.2764
4	1D	AX-108831240	0.2119	32.4883
4	1D	AX-110471446	0.2119	32.4883
4	1D	AX-108819298	0.2119	32.4883
4	1D	AX-109298330	0.2119	32.4883
4	1D	AX-110943880	0.2119	32.4883
4	1D	AX-108778169	0.2119	32.4883
4	1D	AX-109921437	0.2119	32.4883
4	1D	AX-109464635	0.2119	32.4883
4	1D	AX-110916585	0.2119	32.4883
4	1D	AX-111853348	0.2119	32.4883
4	1D	AX-109961653	0.4237	32.912
4	1D	AX-94914889	0.4237	32.912
4	1D	AX-109283402	0.422	33.334
4	1D	AX-109930281	0.422	33.334
4	1D	AX-111121673	0.422	33.334
4	1D	AX-110837208	0.422	33.334
4	1D	AX-110196952	0.422	33.334
4	1D	AX-109043658	0.422	33.334
4	1D	AX-110042335	0.422	33.334
4	1D	AX-108781562	0.422	33.334
4	1D	AX-108728253	0.422	33.334
4	1D	AX-110952118	0.422	33.334
4	1D	AX-111534760	0.422	33.334
4	1D	AX-111036556	0.422	33.334
4	1D	AX-109649039	0.422	33.334
4	1D	AX-110325554	0.422	33.334
4	1D	AX-111298009	0.422	33.334
4	1D	AX-109400324	0.422	33.334
4	1D	AX-109714812	0.422	33.334
4	1D	AX-111485257	0.422	33.334
4	1D	AX-89489627	0.422	33.334

4	1D	AX-109333240	0.422	33.334
4	1D	AX-109057544	0.422	33.334
4	1D	AX-108828080	0.422	33.334
4	1D	AX-109875031	0.422	33.334
4	1D	AX-110591439	0.422	33.334
4	1D	AX-108942669	0.422	33.334
4	1D	AX-110435847	0.422	33.334
4	1D	AX-111504860	0.422	33.334
4	1D	AX-110387208	0.422	33.334
4	1D	AX-110067692	0.422	33.334
4	1D	AX-111536060	0.422	33.334
4	1D	AX-110748646	0.422	33.334
4	1D	AX-110844522	0.422	33.334
4	1D	AX-109278396	0.422	33.334
4	1D	AX-108823893	0.422	33.334
4	1D	AX-110389791	0.422	33.334
4	1D	AX-110026297	0.422	33.334
4	1D	AX-111154755	0.422	33.334
4	1D	AX-110269967	0.422	33.334
4	1D	AX-109629993	0.422	33.334
4	1D	AX-110380305	0.422	33.334
4	1D	AX-110513906	0.422	33.334
4	1D	AX-109914952	0.422	33.334
4	1D	AX-109875856	0.422	33.334
4	1D	AX-110601909	0.422	33.334
4	1D	AX-111589460	0.211	33.5449
4	1D	AX-111515122	7.9084	41.4534
4	1D	AX-109320713	1.299	42.7524
4	1D	AX-109912271	1.299	42.7524
4	1D	AX-110511246	1.299	42.7524
4	1D	AX-110556510	1.299	42.7524
4	1D	AX-110932913	1.299	42.7524
4	1D	AX-95685312	1.299	42.7524
4	1D	AX-109000315	1.299	42.7524
4	1D	AX-108979583	1.299	42.7524
4	1D	AX-110565538	1.299	42.7524
4	1D	AX-111170571	1.299	42.7524
4	1D	AX-109391139	1.299	42.7524
4	1D	AX-108892456	1.299	42.7524
4	1D	AX-110219982	1.299	42.7524
4	1D	AX-112290271	1.299	42.7524
4	1D	AX-110643680	1.299	42.7524
4	1D	AX-111087365	0.4255	43.1779
4	1D	AX-109349990	0.2119	43.3898

4	1D	AX-111944457	0.2119	43.3898
4	1D	AX-111445091	0.2119	43.3898
4	1D	AX-111499949	0.2119	43.3898
4	1D	AX-111172829	0.2119	43.3898
4	1D	AX-111717578	0.2119	43.3898
4	1D	AX-108799531	0.2119	43.3898
4	1D	AX-109223268	0.2119	43.6016
4	1D	AX-109961003	0.2119	43.6016
4	1D	AX-108924215	0.2119	43.6016
4	1D	AX-110837685	0.2119	43.6016
4	1D	AX-109428152	0.2119	43.6016
4	1D	AX-109346292	0.2119	43.6016
4	1D	AX-111739692	0.2119	43.6016
4	1D	AX-111491496	0.2119	43.6016
4	1D	AX-95630130	0.2119	43.6016
4	1D	AX-94882738	0.2119	43.6016
4	1D	AX-111087795	0.2119	43.8135
4	1D	AX-94398233	0.2119	43.8135
4	1D	AX-110438090	1.0731	44.8866
4	1D	AX-108892285	1.0731	44.8866
4	1D	AX-108794352	1.0731	44.8866
4	1D	AX-109020272	1.0731	44.8866
4	1D	AX-111129074	1.0731	44.8866
4	1D	AX-111657285	1.0731	44.8866
4	1D	AX-89352046	1.0731	44.8866
4	1D	AX-94603350	1.0731	44.8866
4	1D	AX-111539796	1.2934	46.18
4	1D	AX-111775358	1.2934	46.18
4	1D	AX-108747591	1.2934	46.18
4	1D	AX-111334634	1.2934	46.18
4	1D	AX-110789232	1.2934	46.18
4	1D	AX-109154181	1.2934	46.18
4	1D	AX-108797722	1.2934	46.18
4	1D	AX-109292966	1.2934	46.18
4	1D	AX-111504007	0.2092	46.3892
4	1D	AX-89575231	0.2092	46.3892
4	1D	AX-111161406	0.2092	46.3892
4	1D	AX-111482260	0.2092	46.3892
4	1D	AX-108942729	0.2092	46.3892
4	1D	AX-89331569	0.2092	46.3892
4	1D	AX-110389611	0.2092	46.3892
4	1D	AX-111157081	0.2092	46.3892
4	1D	AX-111021905	0.2092	46.3892
4	1D	AX-110449657	0.2092	46.3892

4	1D	AX-110782818	0.2092	46.3892
4	1D	AX-111345949	0.2092	46.3892
4	1D	AX-109149937	0.2092	46.3892
4	1D	AX-111658445	0.2092	46.3892
4	1D	AX-109322370	0.2092	46.3892
4	1D	AX-110885924	0.2092	46.3892
4	1D	AX-95216172	0.6356	47.0249
4	1D	AX-86174314	0.2101	47.235
4	1D	AX-110998779	0.2101	47.445
4	1D	AX-110340507	0.2101	47.6551
4	1D	AX-111234221	0.2101	47.6551
4	1D	AX-109403791	0.2101	47.6551
4	1D	AX-109416076	0.2101	47.6551
4	1D	AX-109518722	0.2101	47.6551
4	1D	AX-108760374	0.2101	47.6551
4	1D	AX-111066559	0.2101	47.6551
4	1D	AX-111218609	0.2101	47.6551
4	1D	AX-109998450	0.2092	47.8643
4	1D	AX-110905916	0.2092	47.8643
4	1D	AX-109606349	0.2092	47.8643
4	1D	AX-109276502	0.2092	47.8643
4	1D	AX-109864377	2.4356	50.2999
4	1D	AX-111973796	17.4353	67.7352
4	1D	AX-111534827	0.4202	68.1553
4	1D	AX-89527061	2.3931	70.5485
4	1D	AX-110472474	8.841	79.3895
4	1D	AX-109929813	27.2534	106.6429
4	1D	AX-110948558	5.0866	111.7295
4	1D	AX-109884069	12.3505	124.08
4	1D	AX-108821534	0.2119	124.2918
4	1D	AX-111946510	12.1377	136.4296
4	1D	AX-110581400	1.5156	137.9452
5	1D	AX-94995317	0	0
5	1D	AX-110464533	7.2978	7.2978
5	1D	AX-109423485	7.2978	7.2978
5	1D	AX-109484501	7.2978	7.2978
5	1D	AX-109101747	7.2978	7.2978
5	1D	AX-111727588	7.2978	7.2978
5	1D	AX-111600301	7.2978	7.2978
5	1D	AX-111063123	8.4967	15.7945
5	1D	AX-94386020	6.2829	22.0773
5	1D	AX-111755996	6.2525	28.3298
5	1D	AX-108814409	6.2525	28.3298
5	1D	AX-94725964	0.6494	28.9792

5	1D	AX-94495205	0.4255	29.4047
5	1D	AX-109341366	0.4255	29.4047
5	1D	AX-109456415	0.4255	29.4047
5	1D	AX-110395979	0.4255	29.4047
5	1D	AX-89423498	0.4255	29.4047
5	1D	AX-110937281	0.4255	29.4047
5	1D	AX-110462513	0.4255	29.4047
5	1D	AX-109873900	0.4255	29.4047
5	1D	AX-110991168	0.4255	29.4047
5	1D	AX-94919998	0.4255	29.4047
5	1D	AX-110563128	0.4255	29.4047
5	1D	AX-110496168	1.3103	30.7151
5	1D	AX-109274407	1.3103	30.7151
5	1D	AX-109354064	1.3103	30.7151
5	1D	AX-111613063	13.6441	44.3592
5	1D	AX-111149608	13.6441	44.3592
5	1D	AX-109341206	13.6441	44.3592
5	1D	AX-110986688	13.6441	44.3592
5	1D	AX-110551474	13.6441	44.3592
5	1D	AX-111151046	13.6441	44.3592
5	1D	AX-108850971	13.6441	44.3592
5	1D	AX-109370468	13.6441	44.3592
5	1D	AX-111582916	13.6441	44.3592
5	1D	AX-111570550	13.6441	44.3592
5	1D	AX-111688135	13.6441	44.3592
5	1D	AX-108734580	13.6441	44.3592
5	1D	AX-109400819	13.6441	44.3592
5	1D	AX-109650855	13.6441	44.3592
5	1D	AX-109328365	13.6441	44.3592
6	2A	AX-110402581	0	0
6	2A	AX-111557864	15.4163	15.4163
6	2A	AX-109368419	15.4163	15.4163
6	2A	AX-109887260	1.0506	16.4669
6	2A	AX-94554771	1.0506	16.4669
6	2A	AX-110961126	14.8612	31.3281
6	2A	AX-110608557	14.8612	31.3281
6	2A	AX-111064589	14.8612	31.3281
6	2A	AX-110433540	0.6329	31.961
6	2A	AX-110948843	0.6329	31.961
6	2A	AX-108966684	0.6329	31.961
6	2A	AX-110567710	0.6329	31.961
6	2A	AX-110950179	0.6329	31.961
6	2A	AX-110026721	4.3093	36.2703
6	2A	AX-111650990	0.6303	36.9006

6	2A	AX-109957471	0.8404	37.741
6	2A	AX-110466112	0.8404	37.741
6	2A	AX-109900454	0.4167	38.1577
6	2A	AX-110495160	0.4167	38.1577
6	2A	AX-109405059	0.4167	38.1577
6	2A	AX-111018125	5.6847	43.8424
6	2A	AX-110099691	5.6847	43.8424
6	2A	AX-110490034	4.4302	48.2726
6	2A	AX-110944608	2.2237	50.4962
6	2A	AX-110364988	2.2237	50.4962
6	2A	AX-86173374	2.2237	50.4962
6	2A	AX-111596051	2.2336	52.7299
6	2A	AX-109993386	8.9322	61.6621
6	2A	AX-110514157	0.6383	62.3004
6	2A	AX-109913873	0.6383	62.3004
6	2A	AX-109972523	0.6383	62.3004
6	2A	AX-109993653	0.2092	62.5096
6	2A	AX-110924609	15.1713	77.6809
6	2A	AX-111526140	15.1713	77.6809
6	2A	AX-111586952	15.1713	77.6809
6	2A	AX-111084249	15.1713	77.6809
6	2A	AX-108877841	15.1713	77.6809
6	2A	AX-109988477	15.1713	77.6809
6	2A	AX-108851673	15.1713	77.6809
6	2A	AX-110067600	15.1713	77.6809
6	2A	AX-109422889	15.1713	77.6809
6	2A	AX-108898880	15.1713	77.6809
6	2A	AX-109440330	7.4066	85.0876
6	2A	AX-111167599	0.6383	85.7259
6	2A	AX-110426678	0.6383	85.7259
6	2A	AX-109844516	0.6383	85.7259
6	2A	AX-109301633	0.6383	85.7259
6	2A	AX-109074831	0.6383	85.7259
6	2A	AX-111595047	0.6383	85.7259
6	2A	AX-111196881	0.6383	85.7259
6	2A	AX-109999298	0.6383	85.7259
6	2A	AX-109893073	0.6383	85.7259
6	2A	AX-110904772	0.6383	85.7259
6	2A	AX-110362583	0.6383	85.7259
6	2A	AX-109527495	0.6383	85.7259
6	2A	AX-108751507	1.0685	86.7944
6	2A	AX-111109887	1.0685	86.7944
6	2A	AX-110058817	1.0685	86.7944
6	2A	AX-111011197	1.0685	86.7944

6	2A	AX-108788038	1.0685	86.7944
6	2A	AX-108797998	1.0685	86.7944
6	2A	AX-110493070	1.0685	86.7944
6	2A	AX-110570107	1.0685	86.7944
6	2A	AX-109851355	1.0685	86.7944
6	2A	AX-111666193	1.0685	86.7944
6	2A	AX-109853810	1.0685	86.7944
6	2A	AX-110084490	1.0685	86.7944
6	2A	AX-108783476	1.0685	86.7944
6	2A	AX-110627106	1.0685	86.7944
6	2A	AX-111539062	1.0685	86.7944
6	2A	AX-109898538	1.0685	86.7944
6	2A	AX-110450612	1.0685	86.7944
6	2A	AX-109885725	1.0685	86.7944
6	2A	AX-110181609	1.0685	86.7944
6	2A	AX-110479454	1.0685	86.7944
6	2A	AX-110438187	1.0685	86.7944
6	2A	AX-110450470	1.0685	86.7944
6	2A	AX-110651546	1.0685	86.7944
6	2A	AX-110925367	1.0685	86.7944
6	2A	AX-111636351	1.0685	86.7944
6	2A	AX-108879775	1.0685	86.7944
6	2A	AX-110634829	1.0685	86.7944
6	2A	AX-110449054	1.0685	86.7944
6	2A	AX-109502752	1.0685	86.7944
6	2A	AX-111588837	1.0685	86.7944
6	2A	AX-110485233	1.0685	86.7944
6	2A	AX-109315949	1.0685	86.7944
6	2A	AX-110019462	1.0685	86.7944
6	2A	AX-108748890	1.0685	86.7944
6	2A	AX-109484676	1.0685	86.7944
6	2A	AX-108891438	1.0685	86.7944
6	2A	AX-109498580	1.0685	86.7944
6	2A	AX-109509615	1.0685	86.7944
6	2A	AX-109964835	1.0685	86.7944
6	2A	AX-109431636	1.0685	86.7944
6	2A	AX-111003064	1.0685	86.7944
6	2A	AX-109990785	1.0685	86.7944
6	2A	AX-111013049	0.2101	87.0045
6	2A	AX-109467610	0.2101	87.0045
6	2A	AX-109275420	0.2101	87.0045
6	2A	AX-111461372	0.2101	87.0045
6	2A	AX-109605731	0.2101	87.0045
6	2A	AX-111056196	0.2101	87.0045

6	2A	AX-109341427	0.2101	87.0045
6	2A	AX-109943229	0.2101	87.0045
6	2A	AX-109034287	0.4202	87.4247
6	2A	AX-110108660	0.4202	87.4247
6	2A	AX-111553350	0.4202	87.4247
6	2A	AX-109890821	0.4202	87.4247
6	2A	AX-108768059	0.4202	87.4247
6	2A	AX-109436132	0.4202	87.4247
6	2A	AX-108964849	0.4202	87.4247
6	2A	AX-108880421	0.4202	87.4247
6	2A	AX-111007077	0.4202	87.4247
6	2A	AX-108910006	0.4202	87.4247
6	2A	AX-109910035	0.4202	87.4247
6	2A	AX-108755192	0.4202	87.4247
6	2A	AX-110545577	0.4202	87.4247
6	2A	AX-109652661	0.4202	87.4247
6	2A	AX-108761048	0.4202	87.4247
6	2A	AX-109508374	0.4202	87.4247
6	2A	AX-109553016	0.4202	87.4247
6	2A	AX-110431759	0.4202	87.4247
6	2A	AX-110526044	0.4202	87.4247
6	2A	AX-110045094	0.4202	87.4247
6	2A	AX-110529331	0.4202	87.4247
6	2A	AX-110047893	0.4202	87.4247
6	2A	AX-108951713	0.4202	87.4247
6	2A	AX-111460267	0.4202	87.4247
6	2A	AX-108927217	0.4202	87.4247
6	2A	AX-109399923	0.4202	87.4247
6	2A	AX-108978733	0.4202	87.4247
6	2A	AX-111681607	0.4202	87.4247
6	2A	AX-111700083	0.4202	87.4247
6	2A	AX-108918440	0.4202	87.4247
6	2A	AX-111018597	0.4202	87.4247
6	2A	AX-109332211	0.4202	87.4247
6	2A	AX-110512183	0.4202	87.4247
6	2A	AX-111130827	0.4202	87.4247
6	2A	AX-109374174	0.4202	87.4247
6	2A	AX-108897922	0.4202	87.4247
6	2A	AX-110042217	0.4202	87.4247
6	2A	AX-109516561	0.4202	87.4247
6	2A	AX-110196180	0.4202	87.4247
6	2A	AX-110691711	0.4202	87.4247
6	2A	AX-108780084	0.4202	87.4247
6	2A	AX-111221730	0.4202	87.4247

6	2A	AX-111261001	0.4202	87.4247
6	2A	AX-109932416	0.2083	87.633
6	2A	AX-108875047	0.2083	87.633
6	2A	AX-110390878	0.2083	87.633
6	2A	AX-111547712	0.2083	87.633
6	2A	AX-111586543	0.2083	87.633
6	2A	AX-110651741	0.2083	87.633
6	2A	AX-109850034	0.2083	87.633
6	2A	AX-110960394	0.2083	87.633
6	2A	AX-110649347	0.2083	87.633
6	2A	AX-86174248	0.2083	87.633
6	2A	AX-108734450	0.4149	88.048
6	2A	AX-109312754	0.4149	88.048
6	2A	AX-109393412	0.4149	88.048
6	2A	AX-108873118	0.4149	88.048
6	2A	AX-109325569	0.4149	88.048
6	2A	AX-111635700	0.4149	88.048
6	2A	AX-108933784	0.4149	88.048
6	2A	AX-110394261	0.4149	88.048
6	2A	AX-111514968	0.4149	88.048
6	2A	AX-111591509	0.4149	88.048
6	2A	AX-110717327	0.4149	88.048
6	2A	AX-110390793	0.4149	88.048
6	2A	AX-111570872	0.4149	88.048
6	2A	AX-109895548	0.4149	88.048
6	2A	AX-110921635	0.4149	88.048
6	2A	AX-109970784	0.4149	88.048
6	2A	AX-111566257	0.4149	88.048
6	2A	AX-109835594	0.4149	88.048
6	2A	AX-109318123	0.4149	88.048
6	2A	AX-111523061	0.4149	88.048
6	2A	AX-110976245	0.4149	88.048
6	2A	AX-111193925	0.4149	88.048
6	2A	AX-110035165	0.4149	88.048
6	2A	AX-111037464	0.4149	88.048
6	2A	AX-110058982	0.4149	88.048
6	2A	AX-109298080	0.4149	88.048
6	2A	AX-109826841	0.4149	88.048
6	2A	AX-108960874	0.4149	88.048
6	2A	AX-110412443	0.4149	88.048
6	2A	AX-109287035	0.4149	88.048
6	2A	AX-108743158	0.4149	88.048
6	2A	AX-110146561	0.4149	88.048
6	2A	AX-111515391	0.4149	88.048

6	2A	AX-108900730	0.4149	88.048
6	2A	AX-110937361	0.4149	88.048
6	2A	AX-94570263	0.4149	88.048
6	2A	AX-109321766	0.2049	88.2529
6	2A	AX-111622284	0.2049	88.2529
6	2A	AX-111171797	0.2049	88.2529
6	2A	AX-109834294	0.2049	88.2529
6	2A	AX-111074204	0.2049	88.2529
6	2A	AX-110502353	0.2049	88.2529
6	2A	AX-109988415	0.2049	88.2529
6	2A	AX-108855326	0.2049	88.2529
6	2A	AX-110002595	0.2049	88.2529
6	2A	AX-111757633	0.2049	88.2529
6	2A	AX-109372711	0.2049	88.2529
6	2A	AX-108817032	0.2049	88.2529
6	2A	AX-110609861	0.2049	88.2529
6	2A	AX-111066910	0.2049	88.2529
6	2A	AX-109471644	0.2049	88.2529
6	2A	AX-109923344	0.2049	88.2529
6	2A	AX-110388498	0.2049	88.2529
6	2A	AX-111639409	0.2049	88.2529
6	2A	AX-110669349	0.2049	88.2529
6	2A	AX-109394913	0.2049	88.2529
6	2A	AX-108873286	0.2049	88.2529
6	2A	AX-110671547	0.2049	88.2529
6	2A	AX-108770965	0.2049	88.2529
6	2A	AX-109941791	0.2049	88.2529
6	2A	AX-108976390	0.2049	88.2529
6	2A	AX-111032286	0.2049	88.2529
6	2A	AX-111054566	0.2049	88.2529
6	2A	AX-109328841	0.2049	88.2529
6	2A	AX-108805248	0.2075	88.4604
6	2A	AX-109969172	0.2075	88.4604
6	2A	AX-110906351	0.2075	88.4604
6	2A	AX-110054856	0.2075	88.4604
6	2A	AX-111021698	0.2075	88.4604
6	2A	AX-109958799	0.2075	88.4604
6	2A	AX-111000415	0.2075	88.4604
6	2A	AX-110606051	0.2075	88.4604
6	2A	AX-109395596	0.2075	88.4604
6	2A	AX-109367272	0.2075	88.4604
6	2A	AX-110609555	0.2075	88.4604
6	2A	AX-110555819	0.2075	88.4604
6	2A	AX-111731252	0.2075	88.4604

6	2A	AX-108920539	0.2075	88.4604
6	2A	AX-110026988	0.2075	88.4604
6	2A	AX-110464227	0.2075	88.4604
6	2A	AX-110474801	0.2075	88.4604
6	2A	AX-108858837	0.4149	88.8753
6	2A	AX-110451812	0.4149	88.8753
6	2A	AX-109631196	0.4184	89.2937
6	2A	AX-109043229	0.4184	89.2937
6	2A	AX-108932182	0.4184	89.2937
6	2A	AX-110151691	0.4184	89.2937
6	2A	AX-109869866	0.4184	89.2937
6	2A	AX-110905666	0.4184	89.2937
6	2A	AX-109307445	0.4184	89.2937
6	2A	AX-111470472	0.4184	89.2937
6	2A	AX-111585729	0.4184	89.2937
6	2A	AX-111140886	0.4184	89.2937
6	2A	AX-109586321	0.4184	89.2937
6	2A	AX-110061165	0.4184	89.2937
6	2A	AX-108933054	0.4184	89.2937
6	2A	AX-111458944	0.4184	89.2937
6	2A	AX-110698025	0.4184	89.2937
6	2A	AX-108912261	0.4184	89.2937
6	2A	AX-109832980	0.4184	89.2937
6	2A	AX-109893684	0.4184	89.2937
6	2A	AX-111463139	0.4184	89.2937
6	2A	AX-108817473	0.4184	89.2937
6	2A	AX-110417901	0.4184	89.2937
6	2A	AX-110455728	0.4184	89.2937
6	2A	AX-111567066	0.4184	89.2937
6	2A	AX-94395588	0.4184	89.2937
6	2A	AX-108732604	0.4184	89.2937
6	2A	AX-109383295	0.4184	89.2937
6	2A	AX-109564453	0.4184	89.2937
6	2A	AX-109653951	0.4184	89.2937
6	2A	AX-110951431	0.4184	89.2937
6	2A	AX-86172558	0.4184	89.2937
6	2A	AX-108911487	0.4184	89.2937
6	2A	AX-110568447	0.4184	89.2937
6	2A	AX-111007412	0.2083	89.5021
6	2A	AX-111130098	0.2083	89.5021
6	2A	AX-109914824	0.4184	89.9205
6	2A	AX-110910159	0.4184	89.9205
6	2A	AX-109602702	0.4184	89.9205
6	2A	AX-111619384	0.4184	89.9205

6	2A	AX-109996903	0.4184	89.9205
6	2A	AX-110943070	0.4184	89.9205
6	2A	AX-110175989	0.4184	89.9205
6	2A	AX-109873286	0.4184	89.9205
6	2A	AX-109900862	0.4184	89.9205
6	2A	AX-111464687	0.625	90.5455
6	2A	AX-111007395	0.625	90.5455
6	2A	AX-109086246	0.625	90.5455
6	2A	AX-111257078	0.625	90.5455
6	2A	AX-110379646	0.625	90.5455
6	2A	AX-109053822	0.625	90.5455
6	2A	AX-108882834	0.625	90.5455
6	2A	AX-110546154	0.625	90.5455
6	2A	AX-110719789	0.625	90.5455
6	2A	AX-109850616	0.625	90.5455
6	2A	AX-111016158	0.625	90.5455
6	2A	AX-110412734	0.625	90.5455
6	2A	AX-110003263	0.625	90.5455
6	2A	AX-109072294	0.625	90.5455
6	2A	AX-109830632	0.625	90.5455
6	2A	AX-110471740	0.625	90.5455
6	2A	AX-108945962	0.625	90.5455
6	2A	AX-109356488	0.625	90.5455
6	2A	AX-108854984	0.625	90.5455
6	2A	AX-111485909	0.625	90.5455
6	2A	AX-108910927	0.625	90.5455
6	2A	AX-110394610	0.625	90.5455
6	2A	AX-109901852	0.625	90.5455
6	2A	AX-109418767	0.625	90.5455
6	2A	AX-110632246	0.625	90.5455
6	2A	AX-110478420	0.625	90.5455
6	2A	AX-110147472	0.625	90.5455
6	2A	AX-109277016	0.625	90.5455
6	2A	AX-108794432	0.625	90.5455
6	2A	AX-109829935	0.625	90.5455
6	2A	AX-109973577	0.625	90.5455
6	2A	AX-109373394	0.625	90.5455
6	2A	AX-111040351	0.625	90.5455
6	2A	AX-111094411	0.625	90.5455
6	2A	AX-110694861	0.625	90.5455
6	2A	AX-111657676	0.625	90.5455
6	2A	AX-108954440	0.625	90.5455
6	2A	AX-111125805	0.625	90.5455
6	2A	AX-111749091	0.625	90.5455

6	2A	AX-110169092	0.625	90.5455
6	2A	AX-109915370	0.625	90.5455
6	2A	AX-111527018	0.625	90.5455
6	2A	AX-111731444	0.625	90.5455
6	2A	AX-109401711	0.625	90.5455
6	2A	AX-110485184	0.625	90.5455
6	2A	AX-111068517	0.625	90.5455
6	2A	AX-109909628	0.625	90.5455
6	2A	AX-109361813	0.625	90.5455
6	2A	AX-111470590	0.625	90.5455
6	2A	AX-108892507	0.625	90.5455
6	2A	AX-111265585	0.625	90.5455
6	2A	AX-111782903	0.625	90.5455
6	2A	AX-111212811	0.625	90.5455
6	2A	AX-109482647	0.625	90.5455
6	2A	AX-110382368	0.625	90.5455
6	2A	AX-109475658	0.625	90.5455
6	2A	AX-111155549	0.625	90.5455
6	2A	AX-108836839	0.625	90.5455
6	2A	AX-111595769	0.625	90.5455
6	2A	AX-109477365	0.625	90.5455
6	2A	AX-110554729	0.625	90.5455
6	2A	AX-111684371	0.625	90.5455
6	2A	AX-110583742	0.625	90.5455
6	2A	AX-110653553	0.625	90.5455
6	2A	AX-110059855	0.625	90.5455
6	2A	AX-109418062	0.625	90.5455
6	2A	AX-111238502	0.625	90.5455
6	2A	AX-111464917	0.625	90.5455
6	2A	AX-109876134	0.625	90.5455
6	2A	AX-109559783	0.625	90.5455
6	2A	AX-110742192	0.625	90.5455
6	2A	AX-110440886	0.625	90.5455
6	2A	AX-111638706	0.625	90.5455
6	2A	AX-109877670	0.625	90.5455
6	2A	AX-111090636	0.625	90.5455
6	2A	AX-110708733	0.625	90.5455
6	2A	AX-109277365	0.625	90.5455
6	2A	AX-109467781	0.625	90.5455
6	2A	AX-109071428	0.625	90.5455
6	2A	AX-108927083	0.625	90.5455
6	2A	AX-111132644	0.625	90.5455
6	2A	AX-108924975	0.625	90.5455
6	2A	AX-110516346	0.625	90.5455

6	2A	AX-110530076	0.625	90.5455
6	2A	AX-111220378	0.625	90.5455
6	2A	AX-111022234	0.625	90.5455
6	2A	AX-110543101	0.625	90.5455
6	2A	AX-110975429	0.625	90.5455
6	2A	AX-110472753	0.625	90.5455
6	2A	AX-110150261	0.625	90.5455
6	2A	AX-109030912	0.625	90.5455
6	2A	AX-109582553	0.625	90.5455
6	2A	AX-109934830	0.625	90.5455
6	2A	AX-110927273	0.625	90.5455
6	2A	AX-109277967	0.625	90.5455
6	2A	AX-109980094	0.625	90.5455
6	2A	AX-111284386	0.625	90.5455
6	2A	AX-109408043	0.625	90.5455
6	2A	AX-108736302	0.625	90.5455
6	2A	AX-110192450	0.625	90.5455
6	2A	AX-110965021	0.625	90.5455
6	2A	AX-109445787	0.625	90.5455
6	2A	AX-109899257	0.625	90.5455
6	2A	AX-108892822	0.625	90.5455
6	2A	AX-109090246	0.625	90.5455
6	2A	AX-110049033	0.625	90.5455
6	2A	AX-110692204	0.625	90.5455
6	2A	AX-111081952	0.625	90.5455
6	2A	AX-110585397	0.625	90.5455
6	2A	AX-109882102	0.625	90.5455
6	2A	AX-111032823	0.625	90.5455
6	2A	AX-110147247	0.625	90.5455
6	2A	AX-109837902	0.625	90.5455
6	2A	AX-109878735	0.625	90.5455
6	2A	AX-108861819	0.625	90.5455
6	2A	AX-111768353	0.625	90.5455
6	2A	AX-109384817	0.625	90.5455
6	2A	AX-108962576	0.625	90.5455
6	2A	AX-110696803	0.625	90.5455
6	2A	AX-109085552	0.625	90.5455
6	2A	AX-109939217	0.625	90.5455
6	2A	AX-108743908	0.625	90.5455
6	2A	AX-111264899	0.625	90.5455
6	2A	AX-108946709	0.625	90.5455
6	2A	AX-111218906	0.625	90.5455
6	2A	AX-110479718	0.625	90.5455
6	2A	AX-109335888	0.625	90.5455

6	2A	AX-109860401	0.625	90.5455
6	2A	AX-110130221	0.625	90.5455
6	2A	AX-111238686	0.625	90.5455
6	2A	AX-111530205	0.625	90.5455
6	2A	AX-110673342	0.625	90.5455
6	2A	AX-110036156	0.625	90.5455
6	2A	AX-110716623	0.625	90.5455
6	2A	AX-109926576	0.625	90.5455
6	2A	AX-109496411	0.625	90.5455
6	2A	AX-111722928	0.625	90.5455
6	2A	AX-108811110	0.625	90.5455
6	2A	AX-110714670	0.625	90.5455
6	2A	AX-110498777	0.625	90.5455
6	2A	AX-111586673	0.625	90.5455
6	2A	AX-111459334	0.625	90.5455
6	2A	AX-109297163	0.625	90.5455
6	2A	AX-111242166	0.625	90.5455
6	2A	AX-108817652	0.625	90.5455
6	2A	AX-108909646	0.625	90.5455
6	2A	AX-110672179	0.625	90.5455
6	2A	AX-110400114	0.625	90.5455
6	2A	AX-109403967	0.625	90.5455
6	2A	AX-111826932	0.625	90.5455
6	2A	AX-111671636	0.625	90.5455
6	2A	AX-109073823	0.625	90.5455
6	2A	AX-110491180	0.625	90.5455
6	2A	AX-109650324	0.625	90.5455
6	2A	AX-110925566	0.625	90.5455
6	2A	AX-109933407	0.625	90.5455
6	2A	AX-108876507	0.625	90.5455
6	2A	AX-110063014	0.625	90.5455
6	2A	AX-110002114	0.625	90.5455
6	2A	AX-110561012	0.625	90.5455
6	2A	AX-110552326	0.625	90.5455
6	2A	AX-110925974	0.625	90.5455
6	2A	AX-110679241	0.625	90.5455
6	2A	AX-110676697	0.625	90.5455
6	2A	AX-108826504	0.625	90.5455
6	2A	AX-109591113	0.625	90.5455
6	2A	AX-110432905	0.625	90.5455
6	2A	AX-108954150	0.625	90.5455
6	2A	AX-110169741	0.625	90.5455
6	2A	AX-108882789	0.625	90.5455
6	2A	AX-111118460	0.625	90.5455

6	2A	AX-109456028	0.625	90.5455
6	2A	AX-110669523	0.625	90.5455
6	2A	AX-111217913	0.625	90.5455
6	2A	AX-111581418	0.625	90.5455
6	2A	AX-111008886	0.625	90.5455
6	2A	AX-109084178	0.625	90.5455
6	2A	AX-109468797	0.625	90.5455
6	2A	AX-109884562	0.625	90.5455
6	2A	AX-110557396	0.625	90.5455
6	2A	AX-111699369	0.625	90.5455
6	2A	AX-109868486	0.625	90.5455
6	2A	AX-108747872	0.625	90.5455
6	2A	AX-111019000	0.625	90.5455
6	2A	AX-108926095	0.625	90.5455
6	2A	AX-110644055	0.625	90.5455
6	2A	AX-111603318	0.625	90.5455
6	2A	AX-109079321	0.625	90.5455
6	2A	AX-108876649	0.625	90.5455
6	2A	AX-111484684	0.625	90.5455
6	2A	AX-108937843	0.625	90.5455
6	2A	AX-110196019	0.625	90.5455
6	2A	AX-109836175	0.625	90.5455
6	2A	AX-111026642	0.625	90.5455
6	2A	AX-109941729	0.625	90.5455
6	2A	AX-111656956	0.625	90.5455
6	2A	AX-110474150	0.625	90.5455
6	2A	AX-109034195	0.625	90.5455
6	2A	AX-111552277	0.625	90.5455
6	2A	AX-109988946	0.625	90.5455
6	2A	AX-111058559	0.625	90.5455
6	2A	AX-111288101	0.625	90.5455
6	2A	AX-109304717	0.625	90.5455
6	2A	AX-110065993	0.625	90.5455
6	2A	AX-110996099	0.625	90.5455
6	2A	AX-108903952	0.625	90.5455
6	2A	AX-111516691	0.625	90.5455
6	2A	AX-109378564	0.625	90.5455
6	2A	AX-109289597	0.625	90.5455
6	2A	AX-109976287	0.625	90.5455
6	2A	AX-109647435	0.625	90.5455
6	2A	AX-111584023	0.625	90.5455
6	2A	AX-108815063	0.625	90.5455
6	2A	AX-111464925	0.625	90.5455
6	2A	AX-110157501	0.625	90.5455

6	2A	AX-109398143	0.625	90.5455
6	2A	AX-110565470	0.625	90.5455
6	2A	AX-111567270	0.625	90.5455
6	2A	AX-108803612	0.625	90.5455
6	2A	AX-110120699	0.625	90.5455
6	2A	AX-110708348	0.625	90.5455
6	2A	AX-108915431	0.625	90.5455
6	2A	AX-110592325	0.625	90.5455
6	2A	AX-108743906	0.625	90.5455
6	2A	AX-110198758	0.625	90.5455
6	2A	AX-108908596	0.625	90.5455
6	2A	AX-109291396	0.625	90.5455
6	2A	AX-109279245	0.625	90.5455
6	2A	AX-108956873	0.625	90.5455
6	2A	AX-110185039	0.625	90.5455
6	2A	AX-111214511	0.625	90.5455
6	2A	AX-111057129	0.625	90.5455
6	2A	AX-110583762	0.625	90.5455
6	2A	AX-111165260	0.625	90.5455
6	2A	AX-108869234	0.625	90.5455
6	2A	AX-108928694	0.625	90.5455
6	2A	AX-109931170	0.625	90.5455
6	2A	AX-108837723	0.625	90.5455
6	2A	AX-110999662	0.625	90.5455
6	2A	AX-109954720	0.625	90.5455
6	2A	AX-109604990	0.625	90.5455
6	2A	AX-108903034	0.625	90.5455
6	2A	AX-110918811	0.625	90.5455
6	2A	AX-111509528	0.625	90.5455
6	2A	AX-110372616	0.625	90.5455
6	2A	AX-110135938	0.625	90.5455
6	2A	AX-108814760	0.625	90.5455
6	2A	AX-108854295	0.625	90.5455
6	2A	AX-110577447	0.625	90.5455
6	2A	AX-110513170	0.625	90.5455
6	2A	AX-110962419	0.625	90.5455
6	2A	AX-110153487	0.625	90.5455
6	2A	AX-109306130	0.625	90.5455
6	2A	AX-111253579	0.625	90.5455
6	2A	AX-109352664	0.625	90.5455
6	2A	AX-109035005	0.625	90.5455
6	2A	AX-108788939	0.625	90.5455
6	2A	AX-111691812	0.625	90.5455
6	2A	AX-110559636	0.625	90.5455

6	2A	AX-109375671	0.625	90.5455
6	2A	AX-111549881	0.625	90.5455
6	2A	AX-110033280	0.625	90.5455
6	2A	AX-110057669	0.625	90.5455
6	2A	AX-109033788	0.625	90.5455
6	2A	AX-108972816	0.625	90.5455
6	2A	AX-109507695	0.625	90.5455
6	2A	AX-110721647	0.625	90.5455
6	2A	AX-108900044	0.625	90.5455
6	2A	AX-109299918	0.625	90.5455
6	2A	AX-109579697	0.625	90.5455
6	2A	AX-111785996	0.625	90.5455
6	2A	AX-109041147	0.625	90.5455
6	2A	AX-110048469	0.625	90.5455
6	2A	AX-111064483	0.625	90.5455
6	2A	AX-109283824	0.625	90.5455
6	2A	AX-110932583	0.625	90.5455
6	2A	AX-109869056	0.625	90.5455
6	2A	AX-110927323	0.625	90.5455
6	2A	AX-110363192	0.625	90.5455
6	2A	AX-109338654	0.625	90.5455
6	2A	AX-111148663	0.625	90.5455
6	2A	AX-110141854	0.625	90.5455
6	2A	AX-109459963	0.625	90.5455
6	2A	AX-110044406	0.625	90.5455
6	2A	AX-108809516	0.625	90.5455
6	2A	AX-108792986	0.625	90.5455
6	2A	AX-111690116	0.625	90.5455
6	2A	AX-109858269	0.625	90.5455
6	2A	AX-109510302	0.625	90.5455
6	2A	AX-110194285	0.625	90.5455
6	2A	AX-111680684	0.625	90.5455
6	2A	AX-111649225	0.625	90.5455
6	2A	AX-109503517	0.625	90.5455
6	2A	AX-109836312	0.625	90.5455
6	2A	AX-110915656	0.625	90.5455
6	2A	AX-111097686	0.625	90.5455
6	2A	AX-110515571	0.625	90.5455
6	2A	AX-108860676	0.625	90.5455
6	2A	AX-109400399	0.625	90.5455
6	2A	AX-109063395	0.625	90.5455
6	2A	AX-108886184	0.625	90.5455
6	2A	AX-110532348	0.625	90.5455
6	2A	AX-110068273	0.625	90.5455

6	2A	AX-110720610	0.625	90.5455
6	2A	AX-109823388	0.625	90.5455
6	2A	AX-108967360	0.625	90.5455
6	2A	AX-109444216	0.625	90.5455
6	2A	AX-109850613	0.625	90.5455
6	2A	AX-109446004	0.625	90.5455
6	2A	AX-109903824	0.625	90.5455
6	2A	AX-110929627	0.625	90.5455
6	2A	AX-110669808	0.625	90.5455
6	2A	AX-110153178	0.625	90.5455
6	2A	AX-110445859	0.625	90.5455
6	2A	AX-109897381	0.625	90.5455
6	2A	AX-109818463	0.625	90.5455
6	2A	AX-109366881	0.625	90.5455
6	2A	AX-111484682	0.625	90.5455
6	2A	AX-110669624	0.625	90.5455
6	2A	AX-111544530	0.625	90.5455
6	2A	AX-111697465	0.625	90.5455
6	2A	AX-110927984	0.625	90.5455
6	2A	AX-109890556	0.625	90.5455
6	2A	AX-111220524	0.625	90.5455
6	2A	AX-111214495	0.625	90.5455
6	2A	AX-111572763	0.625	90.5455
6	2A	AX-110998549	0.625	90.5455
6	2A	AX-110510575	0.625	90.5455
6	2A	AX-110916299	0.625	90.5455
6	2A	AX-110017836	0.625	90.5455
6	2A	AX-110559109	0.625	90.5455
6	2A	AX-108854649	0.625	90.5455
6	2A	AX-110430948	0.625	90.5455
6	2A	AX-109078601	0.625	90.5455
6	2A	AX-110481502	0.625	90.5455
6	2A	AX-109956214	0.625	90.5455
6	2A	AX-111618474	0.625	90.5455
6	2A	AX-109858272	0.625	90.5455
6	2A	AX-109600052	0.625	90.5455
6	2A	AX-110061580	0.625	90.5455
6	2A	AX-108728406	0.625	90.5455
6	2A	AX-110423518	0.625	90.5455
6	2A	AX-109896710	0.625	90.5455
6	2A	AX-109299441	0.625	90.5455
6	2A	AX-111765284	0.625	90.5455
6	2A	AX-111690158	0.625	90.5455
6	2A	AX-111578463	0.625	90.5455

6	2A	AX-108862282	0.625	90.5455
6	2A	AX-109275379	0.625	90.5455
6	2A	AX-109621388	0.625	90.5455
6	2A	AX-110581405	0.625	90.5455
6	2A	AX-109413241	0.625	90.5455
6	2A	AX-111641006	0.625	90.5455
6	2A	AX-111639653	0.625	90.5455
6	2A	AX-109284079	0.625	90.5455
6	2A	AX-111118433	0.625	90.5455
6	2A	AX-110453126	0.625	90.5455
6	2A	AX-111680369	0.625	90.5455
6	2A	AX-110537008	0.625	90.5455
6	2A	AX-109960906	0.625	90.5455
6	2A	AX-109057121	0.625	90.5455
6	2A	AX-109043963	0.625	90.5455
6	2A	AX-111621295	0.625	90.5455
6	2A	AX-110909088	0.625	90.5455
6	2A	AX-110998550	0.625	90.5455
6	2A	AX-110744451	0.625	90.5455
6	2A	AX-111784223	0.625	90.5455
6	2A	AX-110174582	0.625	90.5455
6	2A	AX-111288309	0.625	90.5455
6	2A	AX-109310474	0.625	90.5455
6	2A	AX-109411968	0.625	90.5455
6	2A	AX-111262822	0.625	90.5455
6	2A	AX-108878388	0.625	90.5455
6	2A	AX-110399206	0.625	90.5455
6	2A	AX-111073319	0.625	90.5455
6	2A	AX-108752491	0.625	90.5455
6	2A	AX-110969387	0.625	90.5455
6	2A	AX-108741200	0.625	90.5455
6	2A	AX-110720000	0.625	90.5455
6	2A	AX-108917349	0.625	90.5455
6	2A	AX-111070197	0.625	90.5455
6	2A	AX-109083208	0.625	90.5455
6	2A	AX-110740143	0.625	90.5455
6	2A	AX-111597758	0.625	90.5455
6	2A	AX-111563239	0.625	90.5455
6	2A	AX-108833062	0.625	90.5455
6	2A	AX-110151586	0.625	90.5455
6	2A	AX-110597324	0.625	90.5455
6	2A	AX-111485302	0.625	90.5455
6	2A	AX-111545880	0.625	90.5455
6	2A	AX-108730182	0.625	90.5455

6	2A	AX-111508962	0.625	90.5455
6	2A	AX-111262222	0.625	90.5455
6	2A	AX-109502758	0.625	90.5455
6	2A	AX-109866149	0.625	90.5455
6	2A	AX-110521002	0.625	90.5455
6	2A	AX-110913928	0.625	90.5455
6	2A	AX-109330307	0.625	90.5455
6	2A	AX-110415267	0.625	90.5455
6	2A	AX-109892024	0.625	90.5455
6	2A	AX-108954762	0.625	90.5455
6	2A	AX-111553378	0.625	90.5455
6	2A	AX-108869522	0.625	90.5455
6	2A	AX-111012954	0.625	90.5455
6	2A	AX-108790800	0.625	90.5455
6	2A	AX-111026519	0.625	90.5455
6	2A	AX-109601027	0.625	90.5455
6	2A	AX-111103294	0.625	90.5455
6	2A	AX-109058027	0.625	90.5455
6	2A	AX-109816949	0.625	90.5455
6	2A	AX-108878007	0.625	90.5455
6	2A	AX-111156458	0.625	90.5455
6	2A	AX-109897380	0.625	90.5455
6	2A	AX-109450469	0.625	90.5455
6	2A	AX-110477514	0.625	90.5455
6	2A	AX-109919012	0.625	90.5455
6	2A	AX-111652794	0.625	90.5455
6	2A	AX-111136195	0.625	90.5455
6	2A	AX-109471104	0.625	90.5455
6	2A	AX-109448375	0.625	90.5455
6	2A	AX-108896226	0.625	90.5455
6	2A	AX-108931530	0.625	90.5455
6	2A	AX-111053237	0.625	90.5455
6	2A	AX-110744470	0.625	90.5455
6	2A	AX-109324324	0.625	90.5455
6	2A	AX-111580131	0.625	90.5455
6	2A	AX-110462758	0.625	90.5455
6	2A	AX-108853586	0.625	90.5455
6	2A	AX-110969922	0.625	90.5455
6	2A	AX-111262343	0.625	90.5455
6	2A	AX-111701120	0.625	90.5455
6	2A	AX-110527543	0.625	90.5455
6	2A	AX-110169041	0.625	90.5455
6	2A	AX-111657610	0.625	90.5455
6	2A	AX-109603623	0.625	90.5455

6	2A	AX-109063085	0.625	90.5455
6	2A	AX-108747172	0.625	90.5455
6	2A	AX-110710029	0.625	90.5455
6	2A	AX-110172251	0.625	90.5455
6	2A	AX-111623050	0.625	90.5455
6	2A	AX-111550478	0.625	90.5455
6	2A	AX-109927828	0.625	90.5455
6	2A	AX-110455711	0.625	90.5455
6	2A	AX-109086075	0.625	90.5455
6	2A	AX-108748554	0.625	90.5455
6	2A	AX-110416732	0.625	90.5455
6	2A	AX-111056637	0.625	90.5455
6	2A	AX-110016406	0.625	90.5455
6	2A	AX-108964479	0.625	90.5455
6	2A	AX-109373395	0.625	90.5455
6	2A	AX-111214397	0.625	90.5455
6	2A	AX-110459819	0.625	90.5455
6	2A	AX-111238933	0.625	90.5455
6	2A	AX-110045346	0.625	90.5455
6	2A	AX-111068092	0.625	90.5455
6	2A	AX-110490284	0.625	90.5455
6	2A	AX-108959793	0.625	90.5455
6	2A	AX-111674816	0.625	90.5455
6	2A	AX-110163595	0.625	90.5455
6	2A	AX-110058101	0.625	90.5455
6	2A	AX-108862836	0.625	90.5455
6	2A	AX-110059104	0.625	90.5455
6	2A	AX-110579450	0.625	90.5455
6	2A	AX-110020887	0.625	90.5455
6	2A	AX-109361547	0.625	90.5455
6	2A	AX-109401273	0.625	90.5455
6	2A	AX-108903966	0.625	90.5455
6	2A	AX-111755751	0.625	90.5455
6	2A	AX-110053982	0.625	90.5455
6	2A	AX-109876714	0.625	90.5455
6	2A	AX-110623468	0.625	90.5455
6	2A	AX-109295100	0.625	90.5455
6	2A	AX-109344640	0.625	90.5455
6	2A	AX-110019663	0.625	90.5455
6	2A	AX-108983956	0.625	90.5455
6	2A	AX-111779019	0.625	90.5455
6	2A	AX-111767850	0.625	90.5455
6	2A	AX-111759429	0.625	90.5455
6	2A	AX-109920901	0.625	90.5455

6	2A	AX-109076259	0.625	90.5455
6	2A	AX-111030367	0.625	90.5455
6	2A	AX-108736328	0.625	90.5455
6	2A	AX-109955648	0.625	90.5455
6	2A	AX-109416223	0.625	90.5455
6	2A	AX-109329799	0.625	90.5455
6	2A	AX-110176836	0.625	90.5455
6	2A	AX-109905049	0.625	90.5455
6	2A	AX-111228648	0.625	90.5455
6	2A	AX-109319676	0.625	90.5455
6	2A	AX-111117874	0.625	90.5455
6	2A	AX-110366059	0.625	90.5455
6	2A	AX-111035108	0.625	90.5455
6	2A	AX-110913680	0.625	90.5455
6	2A	AX-111009331	0.625	90.5455
6	2A	AX-110976804	0.625	90.5455
6	2A	AX-108971965	0.625	90.5455
6	2A	AX-109312993	0.625	90.5455
6	2A	AX-108758209	0.625	90.5455
6	2A	AX-111664433	0.625	90.5455
6	2A	AX-109873677	0.625	90.5455
6	2A	AX-110958587	0.625	90.5455
6	2A	AX-111026420	0.625	90.5455
6	2A	AX-111625842	0.625	90.5455
6	2A	AX-111015337	0.625	90.5455
6	2A	AX-111065275	0.625	90.5455
6	2A	AX-111284810	0.625	90.5455
6	2A	AX-111141039	0.625	90.5455
6	2A	AX-111285276	0.625	90.5455
6	2A	AX-109871489	0.625	90.5455
6	2A	AX-109881303	0.625	90.5455
6	2A	AX-111560557	0.625	90.5455
6	2A	AX-108938046	0.625	90.5455
6	2A	AX-111566928	0.625	90.5455
6	2A	AX-108877039	0.625	90.5455
6	2A	AX-111630591	0.625	90.5455
6	2A	AX-111457249	0.625	90.5455
6	2A	AX-109626883	0.625	90.5455
6	2A	AX-109985672	0.625	90.5455
6	2A	AX-108850995	0.625	90.5455
6	2A	AX-110508793	0.625	90.5455
6	2A	AX-108801333	0.625	90.5455
6	2A	AX-111091519	0.625	90.5455
6	2A	AX-109517867	0.625	90.5455

6	2A	AX-109093629	0.625	90.5455
6	2A	AX-111596818	0.625	90.5455
6	2A	AX-109074222	0.625	90.5455
6	2A	AX-110913520	0.625	90.5455
6	2A	AX-109583404	0.625	90.5455
6	2A	AX-110972003	0.625	90.5455
6	2A	AX-110939807	0.625	90.5455
6	2A	AX-111083078	0.625	90.5455
6	2A	AX-110544376	0.625	90.5455
6	2A	AX-110609638	0.625	90.5455
6	2A	AX-110991261	0.625	90.5455
6	2A	AX-109366289	0.625	90.5455
6	2A	AX-110491572	0.625	90.5455
6	2A	AX-109599637	0.625	90.5455
6	2A	AX-111461481	0.625	90.5455
6	2A	AX-109607975	0.625	90.5455
6	2A	AX-109583065	0.625	90.5455
6	2A	AX-111543568	0.625	90.5455
6	2A	AX-110572142	0.625	90.5455
6	2A	AX-109302182	0.625	90.5455
6	2A	AX-109971709	0.625	90.5455
6	2A	AX-111282949	0.625	90.5455
6	2A	AX-109879355	0.625	90.5455
6	2A	AX-110921711	0.625	90.5455
6	2A	AX-109650885	0.625	90.5455
6	2A	AX-108907142	0.625	90.5455
6	2A	AX-109085886	0.625	90.5455
6	2A	AX-111684190	0.625	90.5455
6	2A	AX-110909026	0.625	90.5455
6	2A	AX-110430992	0.625	90.5455
6	2A	AX-109373867	0.625	90.5455
6	2A	AX-108791813	0.625	90.5455
6	2A	AX-109325429	0.625	90.5455
6	2A	AX-111065497	0.625	90.5455
6	2A	AX-111223319	0.625	90.5455
6	2A	AX-109620782	0.625	90.5455
6	2A	AX-108804984	0.625	90.5455
6	2A	AX-109277069	0.625	90.5455
6	2A	AX-108828777	0.625	90.5455
6	2A	AX-110415685	0.625	90.5455
6	2A	AX-111667636	0.625	90.5455
6	2A	AX-109616806	0.625	90.5455
6	2A	AX-111504995	0.625	90.5455
6	2A	AX-111507148	0.625	90.5455

6	2A	AX-109847055	0.625	90.5455
6	2A	AX-108757908	0.625	90.5455
6	2A	AX-108970972	0.625	90.5455
6	2A	AX-110398073	0.625	90.5455
6	2A	AX-111078761	0.625	90.5455
6	2A	AX-110410791	0.625	90.5455
6	2A	AX-109454425	0.625	90.5455
6	2A	AX-109301052	0.625	90.5455
6	2A	AX-110669013	0.625	90.5455
6	2A	AX-111768772	0.625	90.5455
6	2A	AX-110107350	0.625	90.5455
6	2A	AX-109998907	0.625	90.5455
6	2A	AX-108847910	0.625	90.5455
6	2A	AX-111286508	0.625	90.5455
6	2A	AX-110505110	0.625	90.5455
6	2A	AX-110499869	0.625	90.5455
6	2A	AX-110417428	0.625	90.5455
6	2A	AX-109886254	0.625	90.5455
6	2A	AX-109545508	0.625	90.5455
6	2A	AX-109859569	0.625	90.5455
6	2A	AX-109053578	0.625	90.5455
6	2A	AX-111281798	0.625	90.5455
6	2A	AX-109815538	0.625	90.5455
6	2A	AX-111630730	0.625	90.5455
6	2A	AX-109104663	0.625	90.5455
6	2A	AX-110374006	0.625	90.5455
6	2A	AX-109317866	0.625	90.5455
6	2A	AX-110926480	0.625	90.5455
6	2A	AX-110554034	0.625	90.5455
6	2A	AX-110402816	0.625	90.5455
6	2A	AX-111283155	0.625	90.5455
6	2A	AX-110174773	0.625	90.5455
6	2A	AX-108913105	0.625	90.5455
6	2A	AX-110197096	0.625	90.5455
6	2A	AX-109486647	0.625	90.5455
6	2A	AX-110427477	0.625	90.5455
6	2A	AX-111500008	0.625	90.5455
6	2A	AX-110686364	0.625	90.5455
6	2A	AX-111051222	0.625	90.5455
6	2A	AX-109469294	0.625	90.5455
6	2A	AX-111262220	0.625	90.5455
6	2A	AX-109340653	0.625	90.5455
6	2A	AX-111285724	0.625	90.5455
6	2A	AX-110009192	0.625	90.5455

6	2A	AX-109624356	0.625	90.5455
6	2A	AX-111530909	0.625	90.5455
6	2A	AX-110985529	0.625	90.5455
6	2A	AX-111512103	0.625	90.5455
6	2A	AX-110961221	0.625	90.5455
6	2A	AX-111522943	0.625	90.5455
6	2A	AX-109616389	0.625	90.5455
6	2A	AX-110692671	0.625	90.5455
6	2A	AX-111242899	0.625	90.5455
6	2A	AX-110177140	0.625	90.5455
6	2A	AX-110609000	0.625	90.5455
6	2A	AX-109271338	0.625	90.5455
6	2A	AX-110920298	0.625	90.5455
6	2A	AX-109652254	0.625	90.5455
6	2A	AX-108762870	0.625	90.5455
6	2A	AX-110739933	0.625	90.5455
6	2A	AX-110063279	0.625	90.5455
6	2A	AX-111578437	0.625	90.5455
6	2A	AX-108893790	0.625	90.5455
6	2A	AX-110688838	0.625	90.5455
6	2A	AX-111614940	0.625	90.5455
6	2A	AX-110197924	0.625	90.5455
6	2A	AX-109997141	0.625	90.5455
6	2A	AX-110409039	0.625	90.5455
6	2A	AX-109470982	0.625	90.5455
6	2A	AX-108858033	0.625	90.5455
6	2A	AX-111581338	0.625	90.5455
6	2A	AX-109937507	0.625	90.5455
6	2A	AX-111027219	0.625	90.5455
6	2A	AX-109997815	0.625	90.5455
6	2A	AX-110534211	0.625	90.5455
6	2A	AX-109062806	0.625	90.5455
6	2A	AX-111262818	0.625	90.5455
6	2A	AX-109067983	0.625	90.5455
6	2A	AX-109929191	0.625	90.5455
6	2A	AX-110050490	0.625	90.5455
6	2A	AX-109463882	0.625	90.5455
6	2A	AX-110703807	0.625	90.5455
6	2A	AX-108845229	0.625	90.5455
6	2A	AX-110489135	0.625	90.5455
6	2A	AX-110598472	0.625	90.5455
6	2A	AX-110441746	0.625	90.5455
6	2A	AX-111681492	0.625	90.5455
6	2A	AX-110061792	0.625	90.5455

6	2A	AX-111137740	0.625	90.5455
6	2A	AX-111243448	0.625	90.5455
6	2A	AX-108894342	0.625	90.5455
6	2A	AX-108912825	0.625	90.5455
6	2A	AX-109838371	0.625	90.5455
6	2A	AX-109523372	0.625	90.5455
6	2A	AX-110581120	0.625	90.5455
6	2A	AX-111083554	0.625	90.5455
6	2A	AX-110709171	0.625	90.5455
6	2A	AX-109410313	0.625	90.5455
6	2A	AX-110388862	0.625	90.5455
6	2A	AX-111264284	0.625	90.5455
6	2A	AX-109630721	0.625	90.5455
6	2A	AX-111038917	0.625	90.5455
6	2A	AX-110465041	0.625	90.5455
6	2A	AX-111287963	0.625	90.5455
6	2A	AX-111287755	0.625	90.5455
6	2A	AX-110564146	0.625	90.5455
6	2A	AX-110563624	0.625	90.5455
6	2A	AX-110524391	0.625	90.5455
6	2A	AX-111508694	0.625	90.5455
6	2A	AX-111192076	0.625	90.5455
6	2A	AX-111075799	0.625	90.5455
6	2A	AX-109531973	0.625	90.5455
6	2A	AX-109549603	0.625	90.5455
6	2A	AX-109585515	0.625	90.5455
6	2A	AX-109909993	0.625	90.5455
6	2A	AX-108807207	0.625	90.5455
6	2A	AX-108900180	0.625	90.5455
6	2A	AX-108816422	0.625	90.5455
6	2A	AX-111224360	0.625	90.5455
6	2A	AX-110718413	0.625	90.5455
6	2A	AX-110555924	0.625	90.5455
6	2A	AX-109502452	0.625	90.5455
6	2A	AX-110595908	0.625	90.5455
6	2A	AX-110178035	0.625	90.5455
6	2A	AX-111652150	0.625	90.5455
6	2A	AX-110193768	0.625	90.5455
6	2A	AX-110381897	0.625	90.5455
6	2A	AX-111039692	0.625	90.5455
6	2A	AX-111164884	0.625	90.5455
6	2A	AX-110708719	0.625	90.5455
6	2A	AX-108943315	0.625	90.5455
6	2A	AX-110041636	0.625	90.5455

6	2A	AX-111526455	0.625	90.5455
6	2A	AX-109651144	0.625	90.5455
6	2A	AX-109419206	0.625	90.5455
6	2A	AX-110667877	0.625	90.5455
6	2A	AX-110688368	0.625	90.5455
6	2A	AX-111019035	0.625	90.5455
6	2A	AX-111033486	0.625	90.5455
6	2A	AX-110043094	0.625	90.5455
6	2A	AX-111055834	0.625	90.5455
6	2A	AX-108817096	0.625	90.5455
6	2A	AX-111490124	0.625	90.5455
6	2A	AX-110586972	0.625	90.5455
6	2A	AX-108825419	0.625	90.5455
6	2A	AX-110546165	0.625	90.5455
6	2A	AX-110718093	0.625	90.5455
6	2A	AX-111506912	0.625	90.5455
6	2A	AX-108778918	0.625	90.5455
6	2A	AX-110062376	0.625	90.5455
6	2A	AX-110933192	0.625	90.5455
6	2A	AX-110089394	0.625	90.5455
6	2A	AX-109041372	0.625	90.5455
6	2A	AX-110975068	0.625	90.5455
6	2A	AX-111047072	0.625	90.5455
6	2A	AX-109320983	0.625	90.5455
6	2A	AX-108915428	0.625	90.5455
6	2A	AX-109331389	0.625	90.5455
6	2A	AX-110394391	0.625	90.5455
6	2A	AX-108909435	0.625	90.5455
6	2A	AX-111232569	0.625	90.5455
6	2A	AX-110559623	0.625	90.5455
6	2A	AX-111629853	0.625	90.5455
6	2A	AX-111657808	0.625	90.5455
6	2A	AX-111158592	0.625	90.5455
6	2A	AX-108772116	0.625	90.5455
6	2A	AX-111547548	0.625	90.5455
6	2A	AX-110559582	0.625	90.5455
6	2A	AX-111670674	0.625	90.5455
6	2A	AX-109460053	0.625	90.5455
6	2A	AX-111510724	0.625	90.5455
6	2A	AX-109105530	0.625	90.5455
6	2A	AX-111613762	0.625	90.5455
6	2A	AX-111219462	0.625	90.5455
6	2A	AX-110624678	0.625	90.5455
6	2A	AX-109328674	0.625	90.5455

6	2A	AX-111487576	0.625	90.5455
6	2A	AX-111112782	0.625	90.5455
6	2A	AX-108798996	0.625	90.5455
6	2A	AX-110382047	0.625	90.5455
6	2A	AX-109643852	0.625	90.5455
6	2A	AX-110459357	0.625	90.5455
6	2A	AX-110577165	0.625	90.5455
6	2A	AX-109855643	0.625	90.5455
6	2A	AX-110365158	0.625	90.5455
6	2A	AX-111582189	0.625	90.5455
6	2A	AX-110151330	0.625	90.5455
6	2A	AX-110505632	0.625	90.5455
6	2A	AX-110714623	0.625	90.5455
6	2A	AX-110990310	0.625	90.5455
6	2A	AX-111505002	0.625	90.5455
6	2A	AX-111764525	0.625	90.5455
6	2A	AX-109872000	0.625	90.5455
6	2A	AX-109064856	0.625	90.5455
6	2A	AX-109101952	0.625	90.5455
6	2A	AX-110130289	0.625	90.5455
6	2A	AX-109448888	0.625	90.5455
6	2A	AX-110477940	0.625	90.5455
6	2A	AX-109827871	0.625	90.5455
6	2A	AX-111068711	0.625	90.5455
6	2A	AX-110695080	0.625	90.5455
6	2A	AX-110589998	0.625	90.5455
6	2A	AX-111000829	0.625	90.5455
6	2A	AX-109062065	0.625	90.5455
6	2A	AX-110028942	0.625	90.5455
6	2A	AX-110155331	0.625	90.5455
6	2A	AX-109325916	0.625	90.5455
6	2A	AX-110479571	0.625	90.5455
6	2A	AX-109275497	0.625	90.5455
6	2A	AX-109271165	0.625	90.5455
6	2A	AX-111559693	0.625	90.5455
6	2A	AX-110573881	0.625	90.5455
6	2A	AX-110413682	0.625	90.5455
6	2A	AX-110909648	0.625	90.5455
6	2A	AX-108738683	0.625	90.5455
6	2A	AX-109271412	0.625	90.5455
6	2A	AX-111736280	0.625	90.5455
6	2A	AX-109969414	0.625	90.5455
6	2A	AX-109835476	0.625	90.5455
6	2A	AX-109995402	0.625	90.5455

6	2A	AX-110584642	0.625	90.5455
6	2A	AX-108863808	0.625	90.5455
6	2A	AX-108987688	0.625	90.5455
6	2A	AX-108849403	0.625	90.5455
6	2A	AX-108879055	0.625	90.5455
6	2A	AX-110923291	0.625	90.5455
6	2A	AX-111258957	0.625	90.5455
6	2A	AX-108830384	0.625	90.5455
6	2A	AX-109559909	0.625	90.5455
6	2A	AX-110457355	0.625	90.5455
6	2A	AX-110667524	0.625	90.5455
6	2A	AX-109817515	0.625	90.5455
6	2A	AX-109900390	0.625	90.5455
6	2A	AX-109849116	0.625	90.5455
6	2A	AX-109653705	0.625	90.5455
6	2A	AX-109383532	0.625	90.5455
6	2A	AX-111551108	0.625	90.5455
6	2A	AX-110434181	0.625	90.5455
6	2A	AX-109306666	0.625	90.5455
6	2A	AX-110386046	0.625	90.5455
6	2A	AX-109588375	0.625	90.5455
6	2A	AX-110381183	0.625	90.5455
6	2A	AX-109517261	0.625	90.5455
6	2A	AX-110698782	0.625	90.5455
6	2A	AX-109927143	0.625	90.5455
6	2A	AX-111126498	0.625	90.5455
6	2A	AX-110073682	0.625	90.5455
6	2A	AX-110499865	0.625	90.5455
6	2A	AX-108889973	0.625	90.5455
6	2A	AX-109929227	0.625	90.5455
6	2A	AX-110976760	0.625	90.5455
6	2A	AX-109403111	0.625	90.5455
6	2A	AX-111638420	0.625	90.5455
6	2A	AX-110678673	0.625	90.5455
6	2A	AX-111630220	0.625	90.5455
6	2A	AX-108847952	0.625	90.5455
6	2A	AX-111140617	0.625	90.5455
6	2A	AX-109977770	0.625	90.5455
6	2A	AX-109930556	0.625	90.5455
6	2A	AX-110055867	0.625	90.5455
6	2A	AX-108780782	0.625	90.5455
6	2A	AX-110129598	0.625	90.5455
6	2A	AX-111462525	0.625	90.5455
6	2A	AX-111489548	0.625	90.5455

6	2A	AX-110160850	0.625	90.5455
6	2A	AX-108850738	0.625	90.5455
6	2A	AX-109833484	0.625	90.5455
6	2A	AX-110949993	0.625	90.5455
6	2A	AX-110465803	0.625	90.5455
6	2A	AX-111289043	0.625	90.5455
6	2A	AX-109375320	0.625	90.5455
6	2A	AX-109495253	0.625	90.5455
6	2A	AX-110561545	0.625	90.5455
6	2A	AX-108826483	0.625	90.5455
6	2A	AX-109916507	0.625	90.5455
6	2A	AX-110547058	0.625	90.5455
6	2A	AX-111631256	0.625	90.5455
6	2A	AX-110160594	0.625	90.5455
6	2A	AX-109070714	0.625	90.5455
6	2A	AX-109521183	0.625	90.5455
6	2A	AX-111687024	0.625	90.5455
6	2A	AX-109401405	0.625	90.5455
6	2A	AX-111216544	0.625	90.5455
6	2A	AX-109627720	0.625	90.5455
6	2A	AX-109582132	0.625	90.5455
6	2A	AX-111091913	0.625	90.5455
6	2A	AX-108831697	0.625	90.5455
6	2A	AX-111139916	0.625	90.5455
6	2A	AX-111650354	0.625	90.5455
6	2A	AX-109034269	0.625	90.5455
6	2A	AX-111602144	0.625	90.5455
6	2A	AX-110029947	0.625	90.5455
6	2A	AX-111097048	0.625	90.5455
6	2A	AX-110925180	0.625	90.5455
6	2A	AX-109851494	0.625	90.5455
6	2A	AX-111023281	0.625	90.5455
6	2A	AX-111103541	0.625	90.5455
6	2A	AX-111596544	0.625	90.5455
6	2A	AX-109923106	0.625	90.5455
6	2A	AX-110671173	0.625	90.5455
6	2A	AX-110970966	0.625	90.5455
6	2A	AX-110133939	0.625	90.5455
6	2A	AX-109433302	0.625	90.5455
6	2A	AX-111647555	0.625	90.5455
6	2A	AX-108941983	0.625	90.5455
6	2A	AX-110029366	0.625	90.5455
6	2A	AX-111077411	0.625	90.5455
6	2A	AX-111130517	0.625	90.5455

6	2A	AX-111098709	0.625	90.5455
6	2A	AX-108760728	0.625	90.5455
6	2A	AX-108779901	0.625	90.5455
6	2A	AX-111028313	0.625	90.5455
6	2A	AX-109920888	0.625	90.5455
6	2A	AX-108782406	0.625	90.5455
6	2A	AX-110039272	0.625	90.5455
6	2A	AX-110507459	0.625	90.5455
6	2A	AX-108888578	0.625	90.5455
6	2A	AX-108786899	0.625	90.5455
6	2A	AX-110080389	0.625	90.5455
6	2A	AX-111059769	0.625	90.5455
6	2A	AX-111477441	0.625	90.5455
6	2A	AX-110019955	0.625	90.5455
6	2A	AX-109395562	0.625	90.5455
6	2A	AX-108728206	0.625	90.5455
6	2A	AX-108870568	0.625	90.5455
6	2A	AX-108933051	0.625	90.5455
6	2A	AX-111018581	0.625	90.5455
6	2A	AX-110671887	0.625	90.5455
6	2A	AX-110404658	0.625	90.5455
6	2A	AX-111141180	0.625	90.5455
6	2A	AX-111262091	0.625	90.5455
6	2A	AX-110393474	0.625	90.5455
6	2A	AX-109586368	0.625	90.5455
6	2A	AX-111643740	0.625	90.5455
6	2A	AX-108866825	0.625	90.5455
6	2A	AX-109062665	0.625	90.5455
6	2A	AX-109952008	0.625	90.5455
6	2A	AX-111517246	0.625	90.5455
6	2A	AX-111462442	0.625	90.5455
6	2A	AX-111091065	0.625	90.5455
6	2A	AX-110392854	0.625	90.5455
6	2A	AX-111072317	0.625	90.5455
6	2A	AX-109881536	0.625	90.5455
6	2A	AX-110036203	0.625	90.5455
6	2A	AX-109302733	0.625	90.5455
6	2A	AX-109973196	0.625	90.5455
6	2A	AX-109075563	0.625	90.5455
6	2A	AX-110971333	0.625	90.5455
6	2A	AX-110380823	0.625	90.5455
6	2A	AX-111155519	0.625	90.5455
6	2A	AX-110585582	0.625	90.5455
6	2A	AX-110508620	0.625	90.5455

6	2A	AX-111037738	0.625	90.5455
6	2A	AX-110451488	0.625	90.5455
6	2A	AX-110560084	0.625	90.5455
6	2A	AX-111613263	0.625	90.5455
6	2A	AX-108728949	0.625	90.5455
6	2A	AX-109492934	0.625	90.5455
6	2A	AX-111489990	0.625	90.5455
6	2A	AX-111640815	0.625	90.5455
6	2A	AX-111026274	0.625	90.5455
6	2A	AX-109085075	0.625	90.5455
6	2A	AX-111765585	0.625	90.5455
6	2A	AX-110698399	0.625	90.5455
6	2A	AX-110566442	0.625	90.5455
6	2A	AX-110965894	0.625	90.5455
6	2A	AX-109038250	0.625	90.5455
6	2A	AX-110457215	0.625	90.5455
6	2A	AX-109342183	0.625	90.5455
6	2A	AX-109886325	0.625	90.5455
6	2A	AX-110068305	0.625	90.5455
6	2A	AX-109999453	0.625	90.5455
6	2A	AX-111467607	0.625	90.5455
6	2A	AX-108730878	0.625	90.5455
6	2A	AX-111241893	0.625	90.5455
6	2A	AX-111004768	0.625	90.5455
6	2A	AX-111603754	0.625	90.5455
6	2A	AX-109958037	0.625	90.5455
6	2A	AX-108797845	0.625	90.5455
6	2A	AX-109102653	0.625	90.5455
6	2A	AX-109360560	0.625	90.5455
6	2A	AX-111485155	0.625	90.5455
6	2A	AX-109279815	0.625	90.5455
6	2A	AX-108764087	0.625	90.5455
6	2A	AX-110972387	0.625	90.5455
6	2A	AX-109043882	0.625	90.5455
6	2A	AX-109031304	0.625	90.5455
6	2A	AX-111265916	0.625	90.5455
6	2A	AX-109982523	0.625	90.5455
6	2A	AX-111568841	0.625	90.5455
6	2A	AX-110163828	0.625	90.5455
6	2A	AX-109104513	0.625	90.5455
6	2A	AX-110923434	0.625	90.5455
6	2A	AX-111502994	0.625	90.5455
6	2A	AX-109286120	0.625	90.5455
6	2A	AX-108922870	0.625	90.5455

6	2A	AX-109520877	0.625	90.5455
6	2A	AX-111553006	0.625	90.5455
6	2A	AX-111155358	0.625	90.5455
6	2A	AX-108970659	0.625	90.5455
6	2A	AX-109988843	0.625	90.5455
6	2A	AX-109653539	0.625	90.5455
6	2A	AX-109081995	0.625	90.5455
6	2A	AX-109630363	0.625	90.5455
6	2A	AX-109920137	0.625	90.5455
6	2A	AX-110485707	0.625	90.5455
6	2A	AX-109622666	0.625	90.5455
6	2A	AX-111543141	0.625	90.5455
6	2A	AX-111017366	0.625	90.5455
6	2A	AX-109035014	0.625	90.5455
6	2A	AX-110043968	0.625	90.5455
6	2A	AX-109989452	0.625	90.5455
6	2A	AX-108767108	0.625	90.5455
6	2A	AX-109298057	0.625	90.5455
6	2A	AX-111115584	0.625	90.5455
6	2A	AX-108930977	0.625	90.5455
6	2A	AX-109581999	0.625	90.5455
6	2A	AX-109857771	0.625	90.5455
6	2A	AX-109905904	0.625	90.5455
6	2A	AX-109989080	0.625	90.5455
6	2A	AX-110529157	0.625	90.5455
6	2A	AX-110709242	0.625	90.5455
6	2A	AX-110949894	0.625	90.5455
6	2A	AX-110997878	0.625	90.5455
6	2A	AX-111036564	0.625	90.5455
6	2A	AX-111105467	0.625	90.5455
6	2A	AX-111131258	0.625	90.5455
6	2A	AX-111626336	0.625	90.5455
6	2A	AX-112291006	0.625	90.5455
6	2A	AX-110090134	0.625	90.5455
6	2A	AX-108887710	0.625	90.5455
6	2A	AX-110078728	0.625	90.5455
6	2A	AX-110544645	0.625	90.5455
6	2A	AX-111031948	0.625	90.5455
6	2A	AX-111690842	0.625	90.5455
6	2A	AX-108978267	0.625	90.5455
6	2A	AX-109365674	0.625	90.5455
6	2A	AX-109444990	0.625	90.5455
6	2A	AX-110076863	0.625	90.5455
6	2A	AX-111197098	0.625	90.5455

6	2A	AX-109893700	0.625	90.5455
6	2A	AX-109974323	0.625	90.5455
6	2A	AX-109012616	0.625	90.5455
6	2A	AX-108836166	0.625	90.5455
6	2A	AX-109299982	0.625	90.5455
6	2A	AX-109331502	0.625	90.5455
6	2A	AX-109918185	0.625	90.5455
6	2A	AX-109967934	0.625	90.5455
6	2A	AX-110035313	0.625	90.5455
6	2A	AX-110070793	0.625	90.5455
6	2A	AX-110080324	0.625	90.5455
6	2A	AX-110092716	0.625	90.5455
6	2A	AX-110094629	0.625	90.5455
6	2A	AX-110454216	0.625	90.5455
6	2A	AX-110496686	0.625	90.5455
6	2A	AX-110529090	0.625	90.5455
6	2A	AX-110585884	0.625	90.5455
6	2A	AX-110605084	0.625	90.5455
6	2A	AX-110606225	0.625	90.5455
6	2A	AX-110641228	0.625	90.5455
6	2A	AX-110926501	0.625	90.5455
6	2A	AX-110981231	0.625	90.5455
6	2A	AX-111002736	0.625	90.5455
6	2A	AX-111172530	0.625	90.5455
6	2A	AX-111655607	0.625	90.5455
6	2A	AX-111736346	0.625	90.5455
6	2A	AX-112286990	0.625	90.5455
6	2A	AX-110006993	0.625	90.5455
6	2A	AX-108735544	0.625	90.5455
6	2A	AX-111064747	0.625	90.5455
6	2A	AX-109382057	0.625	90.5455
6	2A	AX-108958093	0.625	90.5455
6	2A	AX-111150323	0.625	90.5455
6	2A	AX-109084018	0.625	90.5455
6	2A	AX-109847732	0.625	90.5455
6	2A	AX-110589754	0.625	90.5455
6	2A	AX-86184112	0.625	90.5455
6	2A	AX-108815438	0.4202	90.9657
6	2A	AX-109521440	0.4202	91.3859
6	2A	AX-111484908	0.4202	91.3859
6	2A	AX-111759678	0.4202	91.3859
6	2A	AX-111563490	0.4202	91.3859
6	2A	AX-110052411	0.4202	91.3859
6	2A	AX-109966387	0.4202	91.3859

6	2A	AX-111005260	0.4202	91.3859
6	2A	AX-111645780	0.4202	91.3859
6	2A	AX-110438432	0.4202	91.3859
6	2A	AX-111243070	0.4202	91.3859
6	2A	AX-111646438	0.4202	91.3859
6	2A	AX-108808143	0.4202	91.3859
6	2A	AX-109468954	0.2101	91.5959
6	2A	AX-108829822	0.8548	92.4507
6	2A	AX-108827004	0.2101	92.6608
6	2A	AX-111715216	0.4202	93.081
6	2A	AX-109469762	0.4202	93.5012
6	2A	AX-110126474	2.1944	95.6955
6	2A	AX-109343948	0.4255	96.1211
6	2A	AX-110961222	0.4255	96.1211
6	2A	AX-110489401	0.8735	96.9945
6	2A	AX-111581446	0.6466	97.6411
6	2A	AX-110451187	1.4155	99.0566
6	2A	AX-109290429	0.4673	99.5239
6	2A	AX-111709747	0.4673	99.5239
6	2A	AX-109325520	0.422	99.9458
6	2A	AX-109455008	0.422	99.9458
6	2A	AX-110432131	0.422	99.9458
6	2A	AX-110510308	0.422	99.9458
6	2A	AX-108837102	0.422	99.9458
6	2A	AX-109283226	0.422	99.9458
6	2A	AX-86179766	0.2092	100.1551
6	2A	AX-109303703	2.8921	103.0472
6	2A	AX-108856880	2.2041	105.2512
6	2A	AX-109958635	2.1944	107.4456
6	2A	AX-109576676	2.1944	107.4456
6	2A	AX-109983647	2.1944	107.4456
6	2A	AX-109996565	4.7655	112.2111
6	2A	AX-110139178	4.7655	112.2111
6	2A	AX-110166470	4.7655	112.2111
6	2A	AX-110022563	4.7655	112.2111
6	2A	AX-110155009	0.2066	112.4177
6	2A	AX-109275825	0.2066	112.4177
6	2A	AX-109301431	5.3694	117.7871
6	2A	AX-108865385	3.1151	120.9023
6	2A	AX-109843419	0.6276	121.5299
6	2A	AX-109425314	0.6303	122.1602
6	2A	AX-110468560	0.6303	122.1602
6	2A	AX-110398168	0.8512	123.0113
6	2A	AX-108740395	3.8714	126.8827

6	2A	AX-111145618	3.8714	126.8827
6	2A	AX-109417714	0.8512	127.7338
6	2A	AX-109339560	0.8512	127.7338
6	2A	AX-108733196	0.2101	127.9439
6	2A	AX-109904634	0.2101	127.9439
6	2A	AX-109390851	0.2101	127.9439
6	2A	AX-108751272	0.2101	127.9439
6	2A	AX-109082816	0.2101	127.9439
6	2A	AX-110455923	0.2101	127.9439
6	2A	AX-110017739	0.8659	128.8098
6	2A	AX-109043113	0.6438	129.4536
6	2A	AX-109924380	0.6438	129.4536
6	2A	AX-108758337	0.6438	129.4536
6	2A	AX-111026125	2.6692	132.1228
6	2A	AX-110972990	0.8512	132.974
6	2A	AX-108852182	0.8512	132.974
6	2A	AX-109277755	0.8512	132.974
6	2A	AX-108940749	1.2934	134.2674
6	2A	AX-110567094	1.2934	134.2674
6	2A	AX-110033325	1.2934	134.2674
6	2A	AX-111450487	1.2934	134.2674
6	2A	AX-109861211	1.2934	134.2674
6	2A	AX-111168960	1.2934	134.2674
6	2A	AX-111487746	1.2934	134.2674
6	2A	AX-111528968	1.2934	134.2674
6	2A	AX-110463753	1.2934	134.2674
6	2A	AX-109822798	1.2934	134.2674
6	2A	AX-111283384	1.2934	134.2674
6	2A	AX-111136221	1.2934	134.2674
6	2A	AX-108878043	1.2934	134.2674
6	2A	AX-111512257	1.2934	134.2674
6	2A	AX-109271372	1.2934	134.2674
6	2A	AX-110457737	1.2934	134.2674
6	2A	AX-108772729	1.2934	134.2674
6	2A	AX-109595208	1.2934	134.2674
6	2A	AX-109852228	1.2934	134.2674
6	2A	AX-110449568	1.2934	134.2674
6	2A	AX-109397555	1.2934	134.2674
6	2A	AX-110494237	1.2934	134.2674
6	2A	AX-110122578	1.299	135.5664
6	2A	AX-110418781	0.211	135.7774
6	2A	AX-109359227	0.211	135.7774
6	2A	AX-110429464	0.211	135.9883
6	2A	AX-111141805	3.1717	139.16

6	2A	AX-110379064	3.1717	139.16
6	2A	AX-111546127	0.422	139.5819
6	2A	AX-110498851	0.422	139.5819
6	2A	AX-108847326	0.422	139.5819
6	2A	AX-109917880	0.422	139.5819
6	2A	AX-109542304	1.0731	140.6551
6	2A	AX-109556015	0.431	141.0861
6	2A	AX-108777020	4.2156	145.3017
6	2A	AX-109969747	7.8304	153.132
6	2A	AX-110105126	1.055	154.1871
6	2A	AX-111720537	1.055	154.1871
6	2A	AX-111030654	0.2075	154.3945
6	2A	AX-111633954	0.2075	154.3945
6	2A	AX-109321357	1.055	155.4495
6	2A	AX-110109766	5.8957	161.3453
6	2A	AX-109311865	2.4248	163.7701
6	2A	AX-109038782	0.4149	164.185
6	2A	AX-111667876	0.4149	164.185
6	2A	AX-108904491	0.4149	164.185
6	2A	AX-108855103	1.2823	165.4673
6	2A	AX-109448404	2.4573	167.9247
6	2A	AX-110946129	0.8584	168.7831
6	2A	AX-110417390	0.8584	168.7831
6	2A	AX-110949248	1.949	170.7322
6	2A	AX-110060429	1.9323	172.6645
6	2A	AX-110554191	1.9323	172.6645
6	2A	AX-110448758	1.9323	172.6645
6	2A	AX-110979451	0.2083	172.8728
6	2A	AX-108937736	0.2101	173.0829
6	2A	AX-111036826	0.4184	173.5013
6	2A	AX-111462211	0.4184	173.5013
6	2A	AX-110458961	1.2715	174.7728
6	2A	AX-108933205	1.2715	174.7728
6	2A	AX-110449033	9.6169	184.3896
6	2A	AX-110936116	0.4367	184.8263
6	2A	AX-109355803	0.2164	185.0428
6	2A	AX-109504931	0.6277	185.6704
6	2A	AX-110600847	0.6277	185.6704
6	2A	AX-110624465	0.6277	185.6704
6	2A	AX-110991672	0.2075	185.8779
6	2A	AX-109842387	0.2075	185.8779
6	2A	AX-109982059	0.2075	185.8779
6	2A	AX-110134544	0.2075	185.8779
6	2A	AX-109488051	0.8404	186.7183

6	2A	AX-109286381	0.8404	186.7183
6	2A	AX-111663929	0.8404	186.7183
6	2A	AX-109378069	0.6303	187.3486
6	2A	AX-110410190	1.055	188.4036
6	2A	AX-109483522	1.2769	189.6805
6	2A	AX-110086823	1.2769	189.6805
6	2A	AX-110403000	0.6329	190.3134
6	2A	AX-109404528	0.8475	191.161
6	2A	AX-110097396	0.6303	191.7913
6	2A	AX-109416251	1.7174	193.5087
6	2A	AX-111503840	1.7174	193.5087
6	2A	AX-109990976	0.211	193.7197
7	2B	AX-111559149	0	0
7	2B	AX-110425950	0	0
7	2B	AX-111466210	0.4274	0.4274
7	2B	AX-111072033	0.4274	0.4274
7	2B	AX-111606522	0.4274	0.8547
7	2B	AX-110067675	5.3947	6.2494
7	2B	AX-110406750	5.3947	6.2494
7	2B	AX-111614429	0.4202	6.6696
7	2B	AX-109835884	0.6329	7.3025
7	2B	AX-111099555	0.8475	8.1501
7	2B	AX-109852366	0.8475	8.1501
7	2B	AX-108924817	0.8475	8.1501
7	2B	AX-110123426	0.422	8.572
7	2B	AX-109111314	0.422	8.994
7	2B	AX-111234495	0.422	8.994
7	2B	AX-109362868	0.422	8.994
7	2B	AX-111605121	0.422	8.994
7	2B	AX-111500033	3.8363	12.8303
7	2B	AX-109905665	10.9988	23.8291
7	2B	AX-110598098	0.4292	24.2583
7	2B	AX-110038911	3.9433	28.2016
7	2B	AX-110554347	0.2128	28.4144
7	2B	AX-109916461	4.157	32.5714
7	2B	AX-109849847	0.4255	32.9969
7	2B	AX-111825402	0.4255	32.9969
7	2B	AX-109313285	0.2119	33.2088
7	2B	AX-108771065	0.2119	33.2088
7	2B	AX-110953404	6.0096	39.2184
7	2B	AX-108730087	1.5356	40.754
7	2B	AX-109074044	1.5356	40.754
7	2B	AX-111626953	1.5356	40.754
7	2B	AX-109907473	1.5356	40.754

7	2B	AX-108792801	1.5356	40.754
7	2B	AX-108776765	1.5356	40.754
7	2B	AX-110007238	1.5356	40.754
7	2B	AX-109052483	1.5356	40.754
7	2B	AX-89508183	1.5356	40.754
7	2B	AX-110431065	1.5356	40.754
7	2B	AX-108735894	1.5356	40.754
7	2B	AX-109524269	1.5356	40.754
7	2B	AX-110077377	1.5356	40.754
7	2B	AX-111066207	1.5356	40.754
7	2B	AX-111715789	1.5356	40.754
7	2B	AX-108764701	1.5356	40.754
7	2B	AX-111086726	1.5356	40.754
7	2B	AX-110923125	1.5356	40.754
7	2B	AX-108820732	1.5356	40.754
7	2B	AX-109879178	1.5356	40.754
7	2B	AX-108761839	1.5356	40.754
7	2B	AX-110906223	1.5356	40.754
7	2B	AX-109826335	1.5356	40.754
7	2B	AX-109514141	1.5356	40.754
7	2B	AX-111070467	0.6383	41.3923
7	2B	AX-110050024	0.6383	41.3923
7	2B	AX-109322320	0.6383	41.3923
7	2B	AX-109395848	0.6383	41.3923
7	2B	AX-110672203	0.6383	41.3923
7	2B	AX-109979773	0.6383	41.3923
7	2B	AX-109019008	0.6383	41.3923
7	2B	AX-109291168	0.6383	41.3923
7	2B	AX-109284834	0.6383	41.3923
7	2B	AX-111511948	0.6383	41.3923
7	2B	AX-109432408	0.6383	41.3923
7	2B	AX-109348066	0.6383	41.3923
7	2B	AX-110547055	0.6383	41.3923
7	2B	AX-110564422	0.6383	41.3923
7	2B	AX-109825605	0.6383	41.3923
7	2B	AX-108782495	0.6383	41.3923
7	2B	AX-109442463	0.6383	41.3923
7	2B	AX-111210060	0.6383	41.3923
7	2B	AX-89603164	0.6383	41.3923
7	2B	AX-111622432	0.6411	42.0334
7	2B	AX-109817055	0.6411	42.0334
7	2B	AX-110530369	0.6411	42.0334
7	2B	AX-108775737	0.6411	42.0334
7	2B	AX-109425224	0.6411	42.0334

7	2B	AX-108891357	0.6411	42.0334
7	2B	AX-111031470	0.6411	42.0334
7	2B	AX-110492180	0.6411	42.0334
7	2B	AX-109469548	0.6411	42.0334
7	2B	AX-111484090	0.6411	42.0334
7	2B	AX-109845878	0.6411	42.0334
7	2B	AX-111485123	0.6411	42.0334
7	2B	AX-108765393	0.6411	42.0334
7	2B	AX-111779433	0.6411	42.0334
7	2B	AX-111459223	0.6411	42.0334
7	2B	AX-111170778	0.6411	42.0334
7	2B	AX-109952610	0.6411	42.0334
7	2B	AX-110447950	0.6411	42.0334
7	2B	AX-109447136	0.6411	42.0334
7	2B	AX-109370333	0.6411	42.0334
7	2B	AX-109962927	0.6411	42.0334
7	2B	AX-111512423	0.6411	42.0334
7	2B	AX-108738641	0.6411	42.0334
7	2B	AX-109038086	0.6411	42.0334
7	2B	AX-109361861	0.6411	42.0334
7	2B	AX-110003907	0.6411	42.0334
7	2B	AX-111584445	0.4255	42.4589
7	2B	AX-109989078	1.5156	43.9746
7	2B	AX-110383086	1.5156	43.9746
7	2B	AX-108942420	1.5156	43.9746
7	2B	AX-108955097	0.6329	44.6075
7	2B	AX-109305292	0.6329	44.6075
7	2B	AX-109976389	0.6329	44.6075
7	2B	AX-108744297	0.6329	44.6075
7	2B	AX-86176576	0.6329	44.6075
7	2B	AX-109457132	0.4184	45.0259
7	2B	AX-110143958	0.4184	45.0259
7	2B	AX-111139522	0.4184	45.0259
7	2B	AX-110542456	0.4184	45.0259
7	2B	AX-111193111	0.4184	45.0259
7	2B	AX-111212091	0.4184	45.0259
7	2B	AX-109275410	0.4184	45.0259
7	2B	AX-110706315	0.4184	45.0259
7	2B	AX-110457407	0.4184	45.0259
7	2B	AX-110673128	0.4184	45.0259
7	2B	AX-109855470	0.4184	45.0259
7	2B	AX-111029413	0.4184	45.0259
7	2B	AX-110936856	0.4184	45.0259
7	2B	AX-108838208	0.4184	45.0259

7	2B	AX-110965907	0.422	45.4479
7	2B	AX-111031232	0.422	45.4479
7	2B	AX-108739525	0.422	45.4479
7	2B	AX-111640258	8.1599	53.6078
7	2B	AX-108972533	8.1599	53.6078
7	2B	AX-110363228	0.2075	53.8153
7	2B	AX-110673669	0.2075	53.8153
7	2B	AX-108785816	5.8957	59.711
7	2B	AX-111648112	1.3046	61.0156
7	2B	AX-109022733	0.4274	61.443
7	2B	AX-111023892	0.4274	61.443
7	2B	AX-108937984	0.4274	61.443
7	2B	AX-110408247	0.4274	61.443
7	2B	AX-110477689	0.4274	61.8703
7	2B	AX-111689702	0.4274	62.2977
7	2B	AX-110574571	0.4255	62.7232
7	2B	AX-109371038	0.4255	62.7232
7	2B	AX-111608888	0.4255	62.7232
7	2B	AX-110913830	0.4255	62.7232
7	2B	AX-109976421	0.4255	62.7232
7	2B	AX-110002715	0.4255	62.7232
7	2B	AX-108922108	0.4255	62.7232
7	2B	AX-109278921	0.4255	62.7232
7	2B	AX-110548545	0.4255	62.7232
7	2B	AX-109036574	0.4255	62.7232
7	2B	AX-109858757	0.4255	62.7232
7	2B	AX-110386272	0.4255	62.7232
7	2B	AX-109515918	0.4255	62.7232
7	2B	AX-111262600	0.4255	62.7232
7	2B	AX-108749546	0.4255	62.7232
7	2B	AX-108815190	0.4255	62.7232
7	2B	AX-110058714	0.211	62.9342
7	2B	AX-109457111	0.4237	63.358
7	2B	AX-108970178	0.4237	63.358
7	2B	AX-109971644	0.4237	63.358
7	2B	AX-108799311	0.4237	63.358
7	2B	AX-109422034	0.4237	63.358
7	2B	AX-108890992	0.4237	63.358
7	2B	AX-111567428	0.4237	63.358
7	2B	AX-111458861	0.4237	63.358
7	2B	AX-111665206	0.4237	63.358
7	2B	AX-111580040	0.4237	63.358
7	2B	AX-108799498	0.4237	63.358
7	2B	AX-110951329	0.4237	63.358

7	2B	AX-109031140	0.4237	63.358
7	2B	AX-111252057	0.4237	63.358
7	2B	AX-109865965	0.4237	63.358
7	2B	AX-111570677	0.4237	63.358
7	2B	AX-110539196	0.4237	63.358
7	2B	AX-111109669	0.4237	63.358
7	2B	AX-109908767	0.422	63.7799
7	2B	AX-108800290	0.422	63.7799
7	2B	AX-109928673	0.422	63.7799
7	2B	AX-109909891	0.422	63.7799
7	2B	AX-110951531	0.422	63.7799
7	2B	AX-111131556	0.2101	63.99
7	2B	AX-109303300	0.2101	63.99
7	2B	AX-111453640	0.2101	63.99
7	2B	AX-110909274	0.2101	64.2001
7	2B	AX-109032267	0.2101	64.2001
7	2B	AX-108905803	0.2101	64.2001
7	2B	AX-110033945	0.2101	64.2001
7	2B	AX-110529248	0.2101	64.2001
7	2B	AX-109639586	0.2101	64.2001
7	2B	AX-110937016	0.2101	64.2001
7	2B	AX-111542380	0.2101	64.2001
7	2B	AX-108857903	0.2101	64.2001
7	2B	AX-111488005	0.2101	64.2001
7	2B	AX-108895254	0.2101	64.2001
7	2B	AX-110563191	0.2101	64.2001
7	2B	AX-110192248	0.2101	64.2001
7	2B	AX-109933035	0.2101	64.2001
7	2B	AX-108824710	0.2101	64.2001
7	2B	AX-110416969	0.2101	64.2001
7	2B	AX-109442678	0.2101	64.2001
7	2B	AX-109873712	0.2101	64.2001
7	2B	AX-109426374	0.2101	64.2001
7	2B	AX-110978417	0.2101	64.2001
7	2B	AX-110978672	0.2101	64.2001
7	2B	AX-111592076	0.2101	64.2001
7	2B	AX-110185075	0.2101	64.2001
7	2B	AX-110712146	0.2101	64.2001
7	2B	AX-110524970	0.2101	64.2001
7	2B	AX-111699966	0.2101	64.2001
7	2B	AX-110130548	0.2101	64.2001
7	2B	AX-111063087	0.2101	64.2001
7	2B	AX-111109259	0.2101	64.2001
7	2B	AX-108732698	0.2101	64.2001

7	2B	AX-109931115	0.2101	64.2001
7	2B	AX-108844363	0.2101	64.2001
7	2B	AX-109918062	0.2101	64.2001
7	2B	AX-109914436	0.2101	64.2001
7	2B	AX-108827959	0.2101	64.2001
7	2B	AX-109893105	0.2101	64.2001
7	2B	AX-110389802	0.422	64.622
7	2B	AX-111581694	0.422	64.622
7	2B	AX-109429343	0.422	64.622
7	2B	AX-110408514	0.422	64.622
7	2B	AX-111472623	0.422	64.622
7	2B	AX-110938800	0.422	64.622
7	2B	AX-109360265	0.422	64.622
7	2B	AX-110368285	0.422	64.622
7	2B	AX-110925172	0.422	64.622
7	2B	AX-108865163	0.422	64.622
7	2B	AX-110544183	0.422	64.622
7	2B	AX-109877755	0.8512	65.4732
7	2B	AX-110641130	0.8512	65.4732
7	2B	AX-111643433	0.8512	65.4732
7	2B	AX-109919626	0.8512	65.4732
7	2B	AX-111489510	0.8512	65.4732
7	2B	AX-111480841	0.2101	65.6833
7	2B	AX-109879291	0.2101	65.6833
7	2B	AX-110500540	0.2101	65.6833
7	2B	AX-111073595	2.9852	68.6685
7	2B	AX-109302093	0.2165	68.8849
7	2B	AX-110644789	0.2165	68.8849
7	2B	AX-109070541	0.6466	69.5315
7	2B	AX-111684176	1.0824	70.6139
7	2B	AX-110366150	1.0824	70.6139
7	2B	AX-111487427	1.0824	70.6139
7	2B	AX-110495015	1.0824	70.6139
7	2B	AX-109992822	1.0824	70.6139
7	2B	AX-108896991	1.0824	70.6139
7	2B	AX-110653326	1.0824	70.6139
7	2B	AX-111642329	1.0824	70.6139
7	2B	AX-109432306	1.0824	70.6139
7	2B	AX-110004064	1.0824	70.6139
7	2B	AX-108825214	1.0824	70.6139
7	2B	AX-110391760	1.0824	70.6139
7	2B	AX-109909609	1.0824	70.6139
7	2B	AX-111657169	1.0824	70.6139
7	2B	AX-109571162	1.0824	70.6139

7	2B	AX-109865668	1.0824	70.6139
7	2B	AX-111515188	1.0824	70.6139
7	2B	AX-108937964	1.0824	70.6139
7	2B	AX-110097691	1.0824	70.6139
7	2B	AX-111164344	1.0824	70.6139
7	2B	AX-109878775	0.6303	71.2442
7	2B	AX-108874445	0.2083	71.4525
7	2B	AX-110366650	0.2083	71.4525
7	2B	AX-111572581	0.2101	71.6626
7	2B	AX-111013672	0.211	71.8736
7	2B	AX-110912515	0.211	71.8736
7	2B	AX-108799915	0.2092	72.0828
7	2B	AX-109490827	0.2092	72.0828
7	2B	AX-108951642	0.2092	72.0828
7	2B	AX-111168610	0.2092	72.0828
7	2B	AX-111236146	0.2092	72.0828
7	2B	AX-111089389	0.2092	72.0828
7	2B	AX-110455974	0.2092	72.0828
7	2B	AX-111049367	0.2092	72.0828
7	2B	AX-111592114	0.2092	72.0828
7	2B	AX-111038901	0.2092	72.0828
7	2B	AX-108904662	0.2092	72.0828
7	2B	AX-109530577	0.2092	72.0828
7	2B	AX-108915998	0.2092	72.0828
7	2B	AX-110521511	0.2092	72.0828
7	2B	AX-110200039	0.2092	72.0828
7	2B	AX-111463532	0.2092	72.0828
7	2B	AX-109309431	0.2092	72.0828
7	2B	AX-109288940	0.2092	72.0828
7	2B	AX-110494473	0.2092	72.0828
7	2B	AX-109587703	0.2092	72.0828
7	2B	AX-110026150	0.2092	72.0828
7	2B	AX-110669215	0.2092	72.0828
7	2B	AX-108896729	0.2092	72.0828
7	2B	AX-109979166	0.2092	72.0828
7	2B	AX-111549201	0.4202	72.503
7	2B	AX-111709620	0.4202	72.503
7	2B	AX-110411690	0.4202	72.503
7	2B	AX-108893530	0.4202	72.503
7	2B	AX-109993116	0.2137	72.7167
7	2B	AX-89700544	0.2193	72.936
7	2B	AX-94790995	0.6466	73.5826
7	2B	AX-111569735	0.6466	73.5826
7	2B	AX-111089108	0.2092	73.7918

7	2B	AX-111565799	0.4184	74.2102
7	2B	AX-109308718	0.4184	74.2102
7	2B	AX-108966669	0.4184	74.2102
7	2B	AX-110152107	0.4184	74.2102
7	2B	AX-110943249	0.4184	74.2102
7	2B	AX-111612966	0.4184	74.2102
7	2B	AX-111264930	0.2083	74.4185
7	2B	AX-111711178	0.2083	74.4185
7	2B	AX-110448941	0.4202	74.8387
7	2B	AX-111478321	0.2128	75.0515
7	2B	AX-89672508	0.2119	75.2633
7	2B	AX-110448824	0.2119	75.2633
7	2B	AX-109966089	0.2119	75.2633
7	2B	AX-110959236	0.2119	75.2633
7	2B	AX-109341201	0.8475	76.1109
7	2B	AX-109330872	0.8475	76.1109
7	2B	AX-109431885	0.8475	76.1109
7	2B	AX-109909119	0.8475	76.1109
7	2B	AX-109999734	0.8475	76.1109
7	2B	AX-110972149	0.8475	76.1109
7	2B	AX-109547533	0.8475	76.1109
7	2B	AX-109335572	0.2083	76.3192
7	2B	AX-110520808	0.2083	76.3192
7	2B	AX-109318462	0.2083	76.3192
7	2B	AX-111605296	0.2083	76.3192
7	2B	AX-111068233	0.2083	76.3192
7	2B	AX-109865811	0.2083	76.3192
7	2B	AX-108758236	0.2083	76.3192
7	2B	AX-108859133	0.2083	76.3192
7	2B	AX-109491877	0.2083	76.3192
7	2B	AX-108834963	0.2083	76.3192
7	2B	AX-108758077	0.2083	76.3192
7	2B	AX-110911052	0.2083	76.3192
7	2B	AX-109507548	0.2083	76.3192
7	2B	AX-109973772	0.2083	76.3192
7	2B	AX-89410672	0.2083	76.3192
7	2B	AX-110412968	0.422	76.7411
7	2B	AX-109423083	0.422	76.7411
7	2B	AX-111169343	0.422	77.1631
7	2B	AX-109955500	0.422	77.1631
7	2B	AX-109517924	0.422	77.1631
7	2B	AX-110443918	0.211	77.3741
7	2B	AX-110965851	0.6383	78.0124
7	2B	AX-109496660	0.6383	78.0124

7	2B	AX-109506225	0.6383	78.0124
7	2B	AX-111682299	0.6383	78.0124
7	2B	AX-111147889	0.6383	78.0124
7	2B	AX-111456290	0.6383	78.0124
7	2B	AX-111474631	0.6383	78.0124
7	2B	AX-108781411	0.6383	78.0124
7	2B	AX-111257016	0.6383	78.0124
7	2B	AX-111591681	0.6383	78.0124
7	2B	AX-109383212	0.6383	78.0124
7	2B	AX-110977166	0.6383	78.0124
7	2B	AX-109445394	0.6383	78.0124
7	2B	AX-109864445	0.6383	78.0124
7	2B	AX-111562468	0.6383	78.0124
7	2B	AX-110594487	0.6383	78.0124
7	2B	AX-110438573	0.6383	78.0124
7	2B	AX-111624003	0.6383	78.0124
7	2B	AX-110427983	0.6383	78.0124
7	2B	AX-108990842	0.6383	78.0124
7	2B	AX-109974976	0.6383	78.0124
7	2B	AX-110467187	0.6383	78.0124
7	2B	AX-109921058	0.6383	78.0124
7	2B	AX-109859017	0.6383	78.0124
7	2B	AX-109403291	0.6383	78.0124
7	2B	AX-111056394	0.6383	78.0124
7	2B	AX-111563313	0.6383	78.0124
7	2B	AX-86173321	0.6383	78.0124
7	2B	AX-111109976	0.6383	78.0124
7	2B	AX-110478128	0.6383	78.0124
7	2B	AX-110705685	0.6383	78.0124
7	2B	AX-110002376	0.6383	78.0124
7	2B	AX-109535680	0.6383	78.0124
7	2B	AX-109911629	0.6383	78.0124
7	2B	AX-111518284	0.6383	78.0124
7	2B	AX-110525277	0.6383	78.0124
7	2B	AX-108834670	0.6383	78.0124
7	2B	AX-111646161	0.6383	78.0124
7	2B	AX-111558630	0.6383	78.0124
7	2B	AX-111483767	0.6383	78.0124
7	2B	AX-109426471	0.6383	78.0124
7	2B	AX-111653991	0.6383	78.0124
7	2B	AX-111094608	0.6383	78.0124
7	2B	AX-111140614	0.6383	78.0124
7	2B	AX-108791243	0.6383	78.0124
7	2B	AX-110023509	0.6383	78.0124

7	2B	AX-109421637	0.6383	78.0124
7	2B	AX-110502938	0.6383	78.0124
7	2B	AX-110066081	0.6383	78.0124
7	2B	AX-110390653	0.6383	78.0124
7	2B	AX-110587385	0.6383	78.0124
7	2B	AX-111554611	0.6383	78.0124
7	2B	AX-109340415	0.6383	78.0124
7	2B	AX-111121837	0.6383	78.0124
7	2B	AX-108783200	0.6383	78.0124
7	2B	AX-109048108	0.6383	78.0124
7	2B	AX-109970454	0.6383	78.0124
7	2B	AX-108836063	0.6383	78.0124
7	2B	AX-111060300	0.6383	78.0124
7	2B	AX-109582956	0.6383	78.0124
7	2B	AX-111681174	0.6383	78.0124
7	2B	AX-108819005	0.6383	78.0124
7	2B	AX-109299867	0.6383	78.0124
7	2B	AX-111022642	0.6383	78.0124
7	2B	AX-111561046	0.6383	78.0124
7	2B	AX-109454494	0.6383	78.0124
7	2B	AX-110563057	0.6383	78.0124
7	2B	AX-111676422	0.6383	78.0124
7	2B	AX-111026848	0.6383	78.0124
7	2B	AX-109514162	0.6383	78.0124
7	2B	AX-110557100	0.6383	78.0124
7	2B	AX-109600921	0.6383	78.0124
7	2B	AX-111503986	0.6383	78.0124
7	2B	AX-111069050	0.6383	78.0124
7	2B	AX-110416576	0.6383	78.0124
7	2B	AX-109392008	0.6383	78.0124
7	2B	AX-110908678	0.6383	78.0124
7	2B	AX-111492829	0.6383	78.0124
7	2B	AX-111156102	0.6383	78.0124
7	2B	AX-110603724	0.6383	78.0124
7	2B	AX-109653980	0.6383	78.0124
7	2B	AX-108745349	0.6383	78.0124
7	2B	AX-109888683	0.6383	78.0124
7	2B	AX-109290889	0.6383	78.0124
7	2B	AX-109941234	0.6383	78.0124
7	2B	AX-109928587	0.6383	78.0124
7	2B	AX-111008823	0.6383	78.0124
7	2B	AX-109281733	0.6383	78.0124
7	2B	AX-109336055	0.6383	78.0124
7	2B	AX-110388944	0.6383	78.0124

7	2B	AX-110411480	0.6383	78.0124
7	2B	AX-110507446	0.6383	78.0124
7	2B	AX-111688164	0.6383	78.0124
7	2B	AX-111110736	0.6383	78.0124
7	2B	AX-111530235	0.6383	78.0124
7	2B	AX-110383328	0.6383	78.0124
7	2B	AX-108739195	0.6383	78.0124
7	2B	AX-108956436	0.6383	78.0124
7	2B	AX-109495813	0.6383	78.0124
7	2B	AX-110157800	0.6383	78.0124
7	2B	AX-111735530	0.6383	78.0124
7	2B	AX-109893335	0.6383	78.0124
7	2B	AX-110400548	0.6383	78.0124
7	2B	AX-109911888	0.6383	78.0124
7	2B	AX-109512824	0.6383	78.0124
7	2B	AX-111531051	0.6383	78.0124
7	2B	AX-110959136	0.6383	78.0124
7	2B	AX-111461166	0.6383	78.0124
7	2B	AX-111637145	0.6383	78.0124
7	2B	AX-109992485	0.6383	78.0124
7	2B	AX-111604961	0.6383	78.0124
7	2B	AX-111122455	0.6383	78.0124
7	2B	AX-108740331	0.6383	78.0124
7	2B	AX-111177658	0.6383	78.0124
7	2B	AX-111613913	0.6383	78.0124
7	2B	AX-110389432	0.6383	78.0124
7	2B	AX-111113120	0.6383	78.0124
7	2B	AX-109897074	0.6383	78.0124
7	2B	AX-111089695	0.6383	78.0124
7	2B	AX-110519757	0.6383	78.0124
7	2B	AX-109502334	0.6383	78.0124
7	2B	AX-111762842	0.6383	78.0124
7	2B	AX-111775738	0.6383	78.0124
7	2B	AX-111228210	0.6383	78.0124
7	2B	AX-111760323	0.6383	78.0124
7	2B	AX-111043882	0.6383	78.0124
7	2B	AX-110525009	0.6383	78.0124
7	2B	AX-109324552	0.6383	78.0124
7	2B	AX-109904652	0.6383	78.0124
7	2B	AX-111644386	0.6383	78.0124
7	2B	AX-109911080	0.6383	78.0124
7	2B	AX-108860388	0.6383	78.0124
7	2B	AX-110557166	0.6383	78.0124
7	2B	AX-109944832	0.6383	78.0124

7	2B	AX-111147657	0.6383	78.0124
7	2B	AX-110429052	0.6383	78.0124
7	2B	AX-109485345	0.6383	78.0124
7	2B	AX-109476790	0.6383	78.0124
7	2B	AX-110936751	0.6383	78.0124
7	2B	AX-109947314	0.6383	78.0124
7	2B	AX-110957773	0.6383	78.0124
7	2B	AX-109852183	0.6383	78.0124
7	2B	AX-111045653	0.6383	78.0124
7	2B	AX-108756115	0.6383	78.0124
7	2B	AX-110016162	0.6383	78.0124
7	2B	AX-111007493	0.6383	78.0124
7	2B	AX-108868942	0.6383	78.0124
7	2B	AX-109473987	0.6383	78.0124
7	2B	AX-108849306	0.6383	78.0124
7	2B	AX-109884684	0.6383	78.0124
7	2B	AX-108869024	0.6383	78.0124
7	2B	AX-109010755	0.6383	78.0124
7	2B	AX-110103931	0.6383	78.0124
7	2B	AX-110396084	0.6383	78.0124
7	2B	AX-110574697	0.6383	78.0124
7	2B	AX-110443251	0.2101	78.2225
7	2B	AX-109923867	0.6356	78.8581
7	2B	AX-111038927	0.6356	78.8581
7	2B	AX-109980528	0.6356	78.8581
7	2B	AX-110382254	0.6356	78.8581
7	2B	AX-111781100	0.6356	78.8581
7	2B	AX-110481288	0.6356	78.8581
7	2B	AX-111677026	0.6356	78.8581
7	2B	AX-108913586	0.6356	78.8581
7	2B	AX-110436357	0.6356	78.8581
7	2B	AX-109996886	0.6356	78.8581
7	2B	AX-109504123	0.6356	78.8581
7	2B	AX-108740887	0.6356	78.8581
7	2B	AX-108878084	0.6356	78.8581
7	2B	AX-109911305	0.6356	78.8581
7	2B	AX-94500354	0.6356	78.8581
7	2B	AX-109948970	0.6356	78.8581
7	2B	AX-111092595	0.6356	78.8581
7	2B	AX-109498043	0.6356	78.8581
7	2B	AX-111086670	0.4237	79.2819
7	2B	AX-111579036	0.8548	80.1366
7	2B	AX-109403355	0.8548	80.1366
7	2B	AX-110496007	0.8548	80.1366

7	2B	AX-110906506	0.8548	80.1366
7	2B	AX-110431393	0.8548	80.1366
7	2B	AX-109031412	0.6303	80.7669
7	2B	AX-111110572	0.6303	80.7669
7	2B	AX-111735245	0.6303	80.7669
7	2B	AX-109496677	0.6303	80.7669
7	2B	AX-110417277	0.6303	80.7669
7	2B	AX-111561925	0.6303	80.7669
7	2B	AX-109973557	0.6303	80.7669
7	2B	AX-110689474	0.6303	80.7669
7	2B	AX-110457367	0.6303	80.7669
7	2B	AX-109318596	0.6303	80.7669
7	2B	AX-108900955	0.8622	81.6291
7	2B	AX-108790673	0.2155	81.8446
7	2B	AX-111757791	0.4237	82.2683
7	2B	AX-89354175	0.2119	82.4802
7	2B	AX-111716247	0.2128	82.693
7	2B	AX-109279699	0.6356	83.3286
7	2B	AX-111001071	0.2101	83.5387
7	2B	AX-109541446	0.2101	83.5387
7	2B	AX-111213493	0.4292	83.9679
7	2B	AX-110511761	0.4292	83.9679
7	2B	AX-111567291	0.2128	84.1806
7	2B	AX-110511445	0.2128	84.1806
7	2B	AX-111191751	0.211	84.3916
7	2B	AX-110191174	0.211	84.3916
7	2B	AX-110054557	0.211	84.3916
7	2B	AX-109576932	0.211	84.3916
7	2B	AX-109444013	0.211	84.3916
7	2B	AX-108950967	0.211	84.3916
7	2B	AX-110951840	0.211	84.3916
7	2B	AX-109850498	0.211	84.3916
7	2B	AX-111211623	0.211	84.3916
7	2B	AX-110527602	0.211	84.3916
7	2B	AX-108764386	0.211	84.3916
7	2B	AX-111283705	0.211	84.3916
7	2B	AX-110930369	0.211	84.3916
7	2B	AX-110053838	0.211	84.3916
7	2B	AX-111597567	0.211	84.3916
7	2B	AX-110989894	0.211	84.3916
7	2B	AX-108996955	0.211	84.3916
7	2B	AX-111596419	0.211	84.3916
7	2B	AX-111026216	0.211	84.3916
7	2B	AX-111706818	0.211	84.3916

7	2B	AX-111645122	0.211	84.3916
7	2B	AX-109645815	0.211	84.3916
7	2B	AX-109415147	0.211	84.3916
7	2B	AX-111825613	0.211	84.3916
7	2B	AX-110127830	0.211	84.3916
7	2B	AX-110064241	0.211	84.3916
7	2B	AX-108770888	0.211	84.3916
7	2B	AX-110712832	0.211	84.3916
7	2B	AX-110611359	0.211	84.3916
7	2B	AX-110583932	0.211	84.3916
7	2B	AX-110599847	0.211	84.3916
7	2B	AX-109886762	0.211	84.3916
7	2B	AX-109944391	0.211	84.3916
7	2B	AX-110378841	0.211	84.3916
7	2B	AX-111614519	0.211	84.3916
7	2B	AX-110637613	0.211	84.3916
7	2B	AX-110667200	0.211	84.3916
7	2B	AX-94417157	0.211	84.3916
7	2B	AX-108787238	0.211	84.3916
7	2B	AX-109851592	0.211	84.3916
7	2B	AX-111638666	0.211	84.3916
7	2B	AX-111690608	0.211	84.3916
7	2B	AX-111075093	0.211	84.3916
7	2B	AX-109979686	0.211	84.6026
7	2B	AX-110462869	0.211	84.6026
7	2B	AX-109862245	0.4255	85.0281
7	2B	AX-111167874	1.7628	86.791
7	2B	AX-89734740	0.2119	87.0028
7	2B	AX-111499395	1.5026	88.5054
7	2B	AX-108906314	1.5026	88.5054
7	2B	AX-111572959	1.5026	88.5054
7	2B	AX-109846380	1.5026	88.5054
7	2B	AX-111763421	1.5026	88.5054
7	2B	AX-109476020	1.5026	88.5054
7	2B	AX-108964063	1.5026	88.5054
7	2B	AX-109399247	1.5026	88.5054
7	2B	AX-109944548	1.5026	88.5054
7	2B	AX-110026143	1.5026	88.5054
7	2B	AX-111535487	1.5026	88.5054
7	2B	AX-108942798	1.5026	88.5054
7	2B	AX-108767773	1.5026	88.5054
7	2B	AX-111485001	1.5026	88.5054
7	2B	AX-89566661	0.2092	88.7146
7	2B	AX-109327076	0.2092	88.7146

7	2B	AX-111012832	0.2092	88.7146
7	2B	AX-110933437	0.2092	88.7146
7	2B	AX-109386446	0.2092	88.7146
7	2B	AX-110406705	0.2092	88.7146
7	2B	AX-111655842	0.2092	88.7146
7	2B	AX-111662766	0.2092	88.7146
7	2B	AX-109582793	0.2092	88.7146
7	2B	AX-110561698	0.2092	88.7146
7	2B	AX-111061445	0.2092	88.7146
7	2B	AX-89471404	0.2092	88.7146
7	2B	AX-111544566	0.2101	88.9247
7	2B	AX-89358007	1.0967	90.0214
7	2B	AX-110412386	0.2146	90.236
7	2B	AX-109393067	0.2146	90.236
7	2B	AX-108982891	0.2146	90.236
7	2B	AX-109886651	0.2146	90.236
7	2B	AX-109544284	0.2146	90.236
7	2B	AX-109880288	0.2146	90.236
7	2B	AX-109853315	0.2146	90.236
7	2B	AX-109896333	0.2146	90.236
7	2B	AX-111697061	0.2146	90.236
7	2B	AX-111590492	0.2146	90.236
7	2B	AX-109310914	0.2146	90.236
7	2B	AX-110919208	0.2146	90.236
7	2B	AX-108952153	0.2146	90.236
7	2B	AX-109925565	0.2146	90.236
7	2B	AX-108833859	0.2146	90.236
7	2B	AX-111183247	0.2146	90.236
7	2B	AX-111564173	0.2146	90.236
7	2B	AX-110928500	0.2146	90.236
7	2B	AX-111555027	0.6356	90.8716
7	2B	AX-109820944	0.2092	91.0808
7	2B	AX-94493672	1.2823	92.3632
7	2B	AX-111102966	1.2823	92.3632
7	2B	AX-110530754	1.2823	92.3632
7	2B	AX-110998841	1.2823	92.3632
7	2B	AX-110372738	1.2823	92.3632
7	2B	AX-111195176	1.2823	92.3632
7	2B	AX-111010839	1.2823	92.3632
7	2B	AX-94637067	1.2823	92.3632
7	2B	AX-111765215	0.2083	92.5715
7	2B	AX-111054244	0.2083	92.5715
7	2B	AX-110431742	0.2092	92.7807
7	2B	AX-110611175	0.2092	92.7807

7	2B	AX-109934076	0.2092	92.7807
7	2B	AX-110634292	0.2092	92.9899
7	2B	AX-111715350	0.4184	93.4083
7	2B	AX-89336171	0.4184	93.4083
7	2B	AX-108816748	0.4167	93.825
7	2B	AX-111096186	0.4167	93.825
7	2B	AX-111556518	0.4167	93.825
7	2B	AX-109819344	0.4167	93.825
7	2B	AX-109822114	0.4167	93.825
7	2B	AX-109887100	0.4167	93.825
7	2B	AX-108854561	0.4167	93.825
7	2B	AX-108898684	0.4167	93.825
7	2B	AX-111542084	0.4167	93.825
7	2B	AX-89717263	0.4167	93.825
7	2B	AX-110599730	0.4167	93.825
7	2B	AX-111540604	0.4167	93.825
7	2B	AX-109517040	0.4167	93.825
7	2B	AX-108759679	0.4167	93.825
7	2B	AX-108983182	0.4167	93.825
7	2B	AX-109289199	0.4167	93.825
7	2B	AX-110924488	0.4167	93.825
7	2B	AX-108933991	0.4167	93.825
7	2B	AX-109893424	0.4167	93.825
7	2B	AX-108926581	1.4962	95.3212
7	2B	AX-109857426	1.4962	95.3212
7	2B	AX-109452628	1.4962	95.3212
7	2B	AX-109856351	1.4962	95.3212
7	2B	AX-111710161	1.4962	95.3212
7	2B	AX-108750482	1.4962	95.3212
7	2B	AX-94596953	0.6356	95.9568
7	2B	AX-109980782	0.6356	95.9568
7	2B	AX-111007675	0.6356	95.9568
7	2B	AX-109306158	0.6356	95.9568
7	2B	AX-94544933	0.6356	95.9568
7	2B	AX-109481904	0.6356	95.9568
7	2B	AX-110933465	0.422	96.3788
7	2B	AX-111540676	0.422	96.3788
7	2B	AX-111501420	0.422	96.3788
7	2B	AX-109279807	0.422	96.8007
7	2B	AX-111676734	0.422	96.8007
7	2B	AX-89769839	0.4202	97.2209
7	2B	AX-111553009	0.4202	97.2209
7	2B	AX-110434368	0.4202	97.2209
7	2B	AX-111040705	0.2092	97.4301

7	2B	AX-110010926	0.2092	97.4301
7	2B	AX-110120266	0.6356	98.0657
7	2B	AX-111087039	0.2092	98.2749
7	2B	AX-111590094	0.2092	98.2749
7	2B	AX-110430167	0.2092	98.2749
7	2B	AX-109847936	0.2092	98.2749
7	2B	AX-110968135	0.2092	98.2749
7	2B	AX-110640825	0.2092	98.2749
7	2B	AX-110015793	0.2092	98.2749
7	2B	AX-89723442	0.2092	98.2749
7	2B	AX-111478580	0.2083	98.4833
7	2B	AX-110935265	0.2083	98.4833
7	2B	AX-86173854	0.2083	98.4833
7	2B	AX-109386138	0.2092	98.6925
7	2B	AX-110396703	0.2092	98.6925
7	2B	AX-109331624	0.2092	98.6925
7	2B	AX-108866613	3.3383	102.0307
7	2B	AX-109853469	3.3383	102.0307
7	2B	AX-110388252	0.2066	102.2374
7	2B	AX-110914510	0.2066	102.2374
7	2B	AX-111537364	0.2066	102.2374
7	2B	AX-111127090	0.2066	102.2374
7	2B	AX-110049360	0.2066	102.2374
7	2B	AX-110160944	0.2066	102.2374
7	2B	AX-111728700	0.2066	102.2374
7	2B	AX-109883577	0.2066	102.2374
7	2B	AX-110396077	0.2066	102.2374
7	2B	AX-109352812	2.6111	104.8484
7	2B	AX-111639840	1.924	106.7724
7	2B	AX-89589127	1.924	106.7724
7	2B	AX-111049538	1.0595	107.8319
7	2B	AX-110627742	1.0595	108.8914
7	2B	AX-109107523	0.844	109.7354
7	2B	AX-109877981	0.8404	110.5758
7	2B	AX-89365114	0.8404	110.5758
7	2B	AX-111080590	0.4167	110.9925
7	2B	AX-109300203	0.6494	111.6419
7	2B	AX-109938690	1.5423	113.1842
7	2B	AX-110420288	1.7248	114.909
7	2B	AX-111497324	1.7248	114.909
7	2B	AX-111460542	2.854	117.763
7	2B	AX-111003935	2.854	117.763
7	2B	AX-110673774	2.3725	120.1355
7	2B	AX-89458348	0.4167	120.5522

7	2B	AX-109332296	0.4167	120.9688
7	2B	AX-109882373	0.4167	120.9688
7	2B	AX-111645293	0.2101	121.1789
7	2B	AX-109481686	3.3383	124.5172
7	2B	AX-109980364	1.0462	125.5634
7	2B	AX-109451490	1.0462	125.5634
7	2B	AX-111453795	1.0462	125.5634
7	2B	AX-111077755	1.0462	125.5634
7	2B	AX-111557818	1.0462	125.5634
7	2B	AX-110624066	1.924	127.4874
7	2B	AX-109849872	1.924	127.4874
7	2B	AX-108945173	1.924	127.4874
7	2B	AX-108796024	3.0606	130.548
7	2B	AX-108843179	3.0606	130.548
7	2B	AX-111572412	0.2058	130.7538
7	2B	AX-111531916	0.2066	130.9604
7	2B	AX-109521596	8.1599	139.1203
7	2B	AX-111174359	8.1599	139.1203
7	2B	AX-111547699	0.4274	139.5477
7	2B	AX-109993892	1.3046	140.8523
7	2B	AX-110516843	1.3046	140.8523
7	2B	AX-111614697	1.3046	140.8523
7	2B	AX-110906940	1.3046	140.8523
7	2B	AX-108773041	1.3046	140.8523
7	2B	AX-108907234	1.3046	140.8523
7	2B	AX-109285936	0.4237	141.276
7	2B	AX-109477763	6.0388	147.3148
7	2B	AX-111676781	22.1219	169.4367
7	2B	AX-110663094	22.1219	169.4367
8	2D	AX-109464511	0	0
8	2D	AX-111759825	1.924	1.924
8	2D	AX-108762451	1.924	1.924
8	2D	AX-111214360	4.7873	6.7113
8	2D	AX-108926113	8.1194	14.8307
8	2D	AX-111915032	4.1189	18.9496
8	2D	AX-111574926	1.5091	20.4587
8	2D	AX-111630781	8.8725	29.3312
8	2D	AX-111474893	8.8725	29.3312
8	2D	AX-109355233	8.8725	29.3312
8	2D	AX-108907613	8.8725	29.3312
8	2D	AX-111513301	8.8725	29.3312
8	2D	AX-108737397	8.8725	29.3312
8	2D	AX-110332825	8.8725	29.3312
8	2D	AX-110873406	8.8725	29.3312

8	2D	AX-110993844	8.8725	29.3312
8	2D	AX-111623423	0.2058	29.5369
8	2D	AX-110607206	0.2058	29.5369
8	2D	AX-108735235	0.2058	29.5369
8	2D	AX-110384168	0.2058	29.5369
8	2D	AX-108813684	0.2058	29.7427
8	2D	AX-111513562	0.2058	29.7427
8	2D	AX-109218031	0.2058	29.7427
8	2D	AX-110816831	0.2058	29.7427
8	2D	AX-109294560	0.2058	29.7427
8	2D	AX-109292255	0.2058	29.7427
8	2D	AX-108836084	0.2058	29.7427
8	2D	AX-108986429	0.2058	29.7427
8	2D	AX-109839294	0.2058	29.7427
8	2D	AX-111767820	0.2058	29.7427
8	2D	AX-111445772	0.2058	29.7427
8	2D	AX-94814880	0.2058	29.7427
8	2D	AX-109278452	0.2058	29.7427
8	2D	AX-110399827	0.2058	29.7427
8	2D	AX-109356141	0.2058	29.7427
8	2D	AX-109606960	0.4132	30.1559
8	2D	AX-109911369	0.4132	30.1559
8	2D	AX-111087066	40.667	70.8229
8	2D	AX-109785183	10.4678	81.2907
8	2D	AX-109983215	2.4464	83.7371
8	2D	AX-109906393	0.4274	84.1644
8	2D	AX-109402447	1.0778	85.2422
8	2D	AX-110206836	3.1573	88.3996
8	2D	AX-89675016	1.3765	89.776
8	2D	AX-109382452	0.9051	90.6811
8	2D	AX-89563243	2.2138	92.895
8	2D	AX-108882530	5.1342	98.0292
8	2D	AX-111741075	5.1342	98.0292
8	2D	AX-111304532	5.1342	98.0292
8	2D	AX-111102842	5.1342	98.0292
8	2D	AX-109368727	5.1342	98.0292
8	2D	AX-109889290	5.1342	98.0292
8	2D	AX-110605022	5.1342	98.0292
8	2D	AX-108941636	5.1342	98.0292
8	2D	AX-111763561	5.1342	98.0292
8	2D	AX-108988111	5.1342	98.0292
8	2D	AX-109838059	5.1342	98.0292
8	2D	AX-110781588	5.1342	98.0292
8	2D	AX-111573913	5.1342	98.0292

8	2D	AX-110969528	5.1342	98.0292
8	2D	AX-109560947	5.1342	98.0292
8	2D	AX-111611865	5.1342	98.0292
8	2D	AX-109106343	5.1342	98.0292
8	2D	AX-110827866	5.1342	98.0292
8	2D	AX-111093325	5.1342	98.0292
8	2D	AX-110067256	5.1342	98.0292
8	2D	AX-110436671	5.1342	98.0292
8	2D	AX-109442574	5.1342	98.0292
8	2D	AX-109505594	5.1342	98.0292
8	2D	AX-109897046	5.1342	98.0292
8	2D	AX-109479108	5.1342	98.0292
8	2D	AX-109823691	5.1342	98.0292
8	2D	AX-110392640	5.1342	98.0292
8	2D	AX-111692707	5.1342	98.0292
8	2D	AX-109194011	5.1342	98.0292
8	2D	AX-109347546	5.1342	98.0292
8	2D	AX-110567263	5.1342	98.0292
8	2D	AX-109937546	5.1342	98.0292
8	2D	AX-94847111	5.1342	98.0292
8	2D	AX-110768168	5.1342	98.0292
8	2D	AX-110832749	5.1342	98.0292
8	2D	AX-110233688	5.1342	98.0292
8	2D	AX-109309762	5.1342	98.0292
8	2D	AX-110246892	5.1342	98.0292
8	2D	AX-109797326	5.1342	98.0292
8	2D	AX-110406301	5.1342	98.0292
8	2D	AX-111104805	5.1342	98.0292
8	2D	AX-108764231	5.1342	98.0292
8	2D	AX-111111065	5.1342	98.0292
8	2D	AX-109382762	5.1342	98.0292
8	2D	AX-110480427	5.1342	98.0292
8	2D	AX-110613876	5.1342	98.0292
8	2D	AX-111570425	5.1342	98.0292
8	2D	AX-111527344	5.1342	98.0292
8	2D	AX-111863591	5.1342	98.0292
8	2D	AX-111477618	5.1342	98.0292
8	2D	AX-109370958	5.1342	98.0292
8	2D	AX-111357505	5.1342	98.0292
8	2D	AX-110407698	5.1342	98.0292
8	2D	AX-109871706	5.1342	98.0292
8	2D	AX-110468697	5.1342	98.0292
8	2D	AX-111914694	5.1342	98.0292
8	2D	AX-110366748	5.1342	98.0292

8	2D	AX-110667216	5.1342	98.0292
8	2D	AX-110556050	5.1342	98.0292
8	2D	AX-109927945	5.1342	98.0292
8	2D	AX-110538423	5.1342	98.0292
8	2D	AX-111649274	5.1342	98.0292
8	2D	AX-110470645	5.1342	98.0292
8	2D	AX-111420806	5.1342	98.0292
8	2D	AX-110772731	5.1342	98.0292
8	2D	AX-110415488	5.1342	98.0292
8	2D	AX-109944574	5.1342	98.0292
8	2D	AX-108974373	5.1342	98.0292
8	2D	AX-108748325	5.1342	98.0292
8	2D	AX-109311105	5.1342	98.0292
8	2D	AX-109310007	5.1342	98.0292
8	2D	AX-111562993	5.1342	98.0292
8	2D	AX-111525682	5.1342	98.0292
8	2D	AX-108874546	5.1342	98.0292
8	2D	AX-109957403	5.1342	98.0292
8	2D	AX-111062635	5.1342	98.0292
8	2D	AX-111667649	5.1342	98.0292
8	2D	AX-109697988	5.1342	98.0292
8	2D	AX-110703619	5.1342	98.0292
8	2D	AX-111343237	5.1342	98.0292
8	2D	AX-111913088	5.1342	98.0292
8	2D	AX-111587227	5.1342	98.0292
8	2D	AX-110881708	5.1342	98.0292
8	2D	AX-110271360	5.1342	98.0292
8	2D	AX-109989076	5.1342	98.0292
8	2D	AX-110432387	5.1342	98.0292
8	2D	AX-111512487	5.1342	98.0292
8	2D	AX-108736723	5.1342	98.0292
8	2D	AX-108748017	5.1342	98.0292
8	2D	AX-111515549	5.1342	98.0292
8	2D	AX-109032163	5.1342	98.0292
8	2D	AX-110932576	5.1342	98.0292
8	2D	AX-111569162	5.1342	98.0292
8	2D	AX-111801519	5.1342	98.0292
8	2D	AX-111884029	5.1342	98.0292
8	2D	AX-108873150	5.1342	98.0292
8	2D	AX-110509398	5.1342	98.0292
8	2D	AX-110589428	5.1342	98.0292
8	2D	AX-109405881	5.1342	98.0292
8	2D	AX-109904386	5.1342	98.0292
8	2D	AX-111912958	5.1342	98.0292

8	2D	AX-110750300	5.1342	98.0292
8	2D	AX-109736651	5.1342	98.0292
8	2D	AX-111567765	5.1342	98.0292
8	2D	AX-111554868	5.1342	98.0292
8	2D	AX-111454560	5.1342	98.0292
8	2D	AX-111541330	5.1342	98.0292
8	2D	AX-109955778	5.1342	98.0292
8	2D	AX-111378437	5.1342	98.0292
8	2D	AX-111027339	5.1342	98.0292
8	2D	AX-109888836	5.1342	98.0292
8	2D	AX-108818140	5.1342	98.0292
8	2D	AX-109451341	5.1342	98.0292
8	2D	AX-109071906	5.1342	98.0292
8	2D	AX-110941358	5.1342	98.0292
8	2D	AX-111596411	5.1342	98.0292
8	2D	AX-110832801	5.1342	98.0292
8	2D	AX-111087159	5.1342	98.0292
8	2D	AX-110686232	5.1342	98.0292
8	2D	AX-111013641	5.1342	98.0292
8	2D	AX-110470900	5.1342	98.0292
8	2D	AX-109976434	5.1342	98.0292
8	2D	AX-109371141	5.1342	98.0292
8	2D	AX-110497873	5.1342	98.0292
8	2D	AX-109733848	5.1342	98.0292
8	2D	AX-111592735	5.1342	98.0292
8	2D	AX-110759554	5.1342	98.0292
8	2D	AX-109332224	5.1342	98.0292
8	2D	AX-109373043	5.1342	98.0292
8	2D	AX-109344118	5.1342	98.0292
8	2D	AX-94507001	5.1342	98.0292
8	2D	AX-109909451	5.1342	98.0292
8	2D	AX-109866353	5.1342	98.0292
8	2D	AX-109686586	5.1342	98.0292
8	2D	AX-110049124	5.1342	98.0292
8	2D	AX-111360156	5.1342	98.0292
8	2D	AX-109505172	5.1342	98.0292
8	2D	AX-111159698	5.1342	98.0292
8	2D	AX-110226863	5.1342	98.0292
8	2D	AX-111617765	5.1342	98.0292
8	2D	AX-111466264	5.1342	98.0292
8	2D	AX-109278936	5.1342	98.0292
8	2D	AX-109954190	5.1342	98.0292
8	2D	AX-109149641	5.1342	98.0292
8	2D	AX-110233894	5.1342	98.0292

8	2D	AX-109836774	5.1342	98.0292
8	2D	AX-89504874	5.1342	98.0292
8	2D	AX-110108624	5.1342	98.0292
8	2D	AX-109844870	5.1342	98.0292
8	2D	AX-111426570	5.1342	98.0292
8	2D	AX-108795156	5.1342	98.0292
8	2D	AX-109894326	5.1342	98.0292
8	2D	AX-110386671	5.1342	98.0292
8	2D	AX-111296979	5.1342	98.0292
8	2D	AX-111609336	5.1342	98.0292
8	2D	AX-109668767	5.1342	98.0292
8	2D	AX-109914710	5.1342	98.0292
8	2D	AX-110382680	5.1342	98.0292
8	2D	AX-111801037	5.1342	98.0292
8	2D	AX-110359910	5.1342	98.0292
8	2D	AX-108811411	5.1342	98.0292
8	2D	AX-108995095	5.1342	98.0292
8	2D	AX-109300636	5.1342	98.0292
8	2D	AX-109789836	5.1342	98.0292
8	2D	AX-111013389	5.1342	98.0292
8	2D	AX-111184406	5.1342	98.0292
8	2D	AX-108858945	5.1342	98.0292
8	2D	AX-111128536	2.6811	100.7103
8	2D	AX-110954551	0.422	101.1323
8	2D	AX-111726271	0.422	101.1323
8	2D	AX-109007985	0.422	101.1323
8	2D	AX-111708562	0.422	101.1323
8	2D	AX-109032551	0.422	101.1323
8	2D	AX-109300248	0.422	101.1323
8	2D	AX-109731670	0.2092	101.3415
8	2D	AX-109622555	0.2092	101.3415
8	2D	AX-110898898	0.2092	101.3415
8	2D	AX-110038812	0.2092	101.3415
8	2D	AX-110697826	0.2092	101.3415
8	2D	AX-109286158	0.2092	101.3415
8	2D	AX-110895337	0.6329	101.9745
8	2D	AX-109982080	0.6329	101.9745
8	2D	AX-109899332	0.6329	101.9745
8	2D	AX-94536025	0.6329	101.9745
8	2D	AX-111545463	0.2101	102.1845
8	2D	AX-109525831	0.2101	102.1845
8	2D	AX-109947030	0.2101	102.1845
8	2D	AX-110905532	0.2101	102.3946
8	2D	AX-86168790	0.2101	102.3946

8	2D	AX-94569767	0.2101	102.3946
8	2D	AX-110636997	0.2101	102.3946
8	2D	AX-110192731	0.2101	102.3946
8	2D	AX-111171635	0.2101	102.3946
8	2D	AX-94488983	0.6303	103.0249
8	2D	AX-111591148	0.6303	103.0249
8	2D	AX-111906186	0.6303	103.0249
8	2D	AX-110374461	0.6303	103.0249
8	2D	AX-109903618	0.6303	103.0249
8	2D	AX-108735612	0.6303	103.0249
8	2D	AX-108810643	0.6303	103.0249
8	2D	AX-110883351	0.6303	103.0249
8	2D	AX-109908258	0.6303	103.0249
8	2D	AX-110752353	0.6303	103.0249
8	2D	AX-110815691	0.6303	103.0249
8	2D	AX-109818223	0.6303	103.0249
8	2D	AX-111569781	0.6303	103.0249
8	2D	AX-108804358	0.6303	103.0249
8	2D	AX-111188329	0.6303	103.0249
8	2D	AX-111297034	0.6303	103.0249
8	2D	AX-109140621	0.6303	103.0249
8	2D	AX-111763473	0.6303	103.0249
8	2D	AX-110002051	0.6303	103.0249
8	2D	AX-110431006	0.6303	103.0249
8	2D	AX-109185514	0.6303	103.0249
8	2D	AX-109687504	0.6303	103.0249
8	2D	AX-108897531	0.6303	103.0249
8	2D	AX-110415035	0.6303	103.0249
8	2D	AX-109375042	0.6303	103.0249
8	2D	AX-110460468	0.6303	103.0249
8	2D	AX-110951950	0.6303	103.0249
8	2D	AX-111134444	0.6303	103.0249
8	2D	AX-110368761	0.6303	103.0249
8	2D	AX-109876533	0.6303	103.0249
8	2D	AX-110000610	0.6303	103.0249
8	2D	AX-109845140	0.2083	103.2332
8	2D	AX-110074832	0.2083	103.2332
8	2D	AX-108789925	0.2083	103.2332
8	2D	AX-111920104	0.2083	103.2332
8	2D	AX-109895152	0.2083	103.2332
8	2D	AX-110958528	9.5678	112.8011
8	2D	AX-109873946	0.2101	113.0112
8	2D	AX-108814021	2.905	115.9162
8	2D	AX-109438413	2.905	115.9162

8	2D	AX-109455709	2.905	115.9162
8	2D	AX-110470890	2.905	115.9162
8	2D	AX-110219513	2.905	115.9162
8	2D	AX-108793260	0.2092	116.1254
8	2D	AX-108820900	0.2092	116.1254
8	2D	AX-110977542	0.2092	116.1254
8	2D	AX-111969098	0.2092	116.1254
8	2D	AX-110965312	0.4202	116.5456
8	2D	AX-111039289	0.8475	117.3931
8	2D	AX-111430851	0.8475	117.3931
8	2D	AX-109302441	0.6303	118.0234
8	2D	AX-111083601	0.6303	118.0234
8	2D	AX-109276654	0.6303	118.0234
8	2D	AX-109913646	0.6303	118.0234
8	2D	AX-111300870	0.6303	118.0234
8	2D	AX-111116269	0.6303	118.0234
8	2D	AX-110185454	0.6303	118.0234
8	2D	AX-111838598	0.6303	118.0234
8	2D	AX-111976176	1.0595	119.0829
8	2D	AX-111007074	1.0595	119.0829
8	2D	AX-109119158	1.0595	119.0829
8	2D	AX-109417243	0.4202	119.5031
8	2D	AX-108976867	1.0731	120.5762
8	2D	AX-110010295	2.4355	123.0118
8	2D	AX-89690184	0.8512	123.8629
8	2D	AX-110515536	0.422	124.2849
8	2D	AX-110682929	4.9224	129.2072
8	2D	AX-109449257	1.0919	130.2991
8	2D	AX-109246010	3.907	134.2061
8	2D	AX-110823690	3.3383	137.5444
8	2D	AX-110899429	2.1659	139.7103
8	2D	AX-110411457	2.1659	139.7103
8	2D	AX-110929471	0.8512	140.5614
8	2D	AX-109847853	0.2119	140.7733
8	2D	AX-109779577	0.2119	140.7733
8	2D	AX-110485212	0.2119	140.7733
8	2D	AX-110423675	0.2119	140.9852
8	2D	AX-109509798	0.2119	140.9852
8	2D	AX-109985782	0.2119	140.9852
8	2D	AX-110833961	0.4237	141.4089
8	2D	AX-108926296	0.211	141.6199
8	2D	AX-109911091	0.211	141.6199
8	2D	AX-111684087	0.211	141.6199
8	2D	AX-109274813	0.211	141.6199

8	2D	AX-109419238	0.211	141.6199
8	2D	AX-94427799	0.4292	142.0491
8	2D	AX-109615884	0.431	142.4801
8	2D	AX-109059601	0.4255	142.9056
8	2D	AX-109783146	0.4255	142.9056
8	2D	AX-111491026	0.4255	143.3312
8	2D	AX-110002863	0.4255	143.3312
8	2D	AX-110007515	0.2137	143.5449
8	2D	AX-109645731	0.2232	143.7681
8	2D	AX-111857979	0.2232	143.9913
8	2D	AX-109459272	0.4292	144.4205
8	2D	AX-111601893	0.4292	144.4205
8	2D	AX-111318850	1.1064	145.5269
8	2D	AX-109412860	0.2155	145.7424
8	2D	AX-110465469	0.2155	145.7424
8	2D	AX-110573324	0.4292	146.1716
8	2D	AX-111141477	0.6411	146.8126
8	2D	AX-111341034	1.7474	148.5601
8	2D	AX-109295840	1.7474	148.5601
8	2D	AX-109945455	1.7474	148.5601
8	2D	AX-109421761	1.7474	148.5601
8	2D	AX-109351097	0.6356	149.1957
8	2D	AX-109035593	0.8585	150.0541
8	2D	AX-111915441	0.4255	150.4797
8	2D	AX-109914489	0.4237	150.9034
8	2D	AX-89364729	0.4237	150.9034
8	2D	AX-111453536	1.0731	151.9765
8	2D	AX-110961575	0.4255	152.4021
8	2D	AX-109438856	0.4255	152.8276
8	2D	AX-109724216	0.4255	152.8276
8	2D	AX-108775408	0.4255	152.8276
8	2D	AX-109311184	0.4274	153.255
8	2D	AX-111467211	2.4355	155.6905
8	2D	AX-110434103	2.4355	155.6905
8	2D	AX-108893965	0.2119	155.9024
8	2D	AX-110952458	2.1944	158.0968
8	2D	AX-109546756	17.1195	175.2162
8	2D	AX-110052579	17.1195	175.2162
8	2D	AX-108914422	23.7182	198.9344
8	2D	AX-109894807	0.4237	199.3582
8	2D	AX-109399693	0.4237	199.3582
8	2D	AX-111379159	4.8993	204.2575
8	2D	AX-110201452	4.8993	204.2575
8	2D	AX-111761440	4.8993	204.2575

8	2D	AX-110051330	4.8993	204.2575
8	2D	AX-110095870	0.2092	204.4667
8	2D	AX-109723270	0.2092	204.4667
8	2D	AX-110970313	0.2092	204.4667
8	2D	AX-111096304	0.8512	205.3178
8	2D	AX-109471795	0.8512	205.3178
8	2D	AX-111530828	0.4237	205.7416
8	2D	AX-110478994	0.4237	205.7416
8	2D	AX-94493243	0.4237	205.7416
8	2D	AX-110518548	0.4237	205.7416
8	2D	AX-94613237	0.4237	205.7416
8	2D	AX-110530896	0.4237	205.7416
8	2D	AX-108733686	0.4237	205.7416
8	2D	AX-109334464	0.4237	205.7416
8	2D	AX-108730273	0.4237	205.7416
8	2D	AX-110245078	0.4237	205.7416
8	2D	AX-110987764	0.4237	205.7416
8	2D	AX-108979916	0.4237	205.7416
8	2D	AX-110638595	0.4237	205.7416
8	2D	AX-109585965	0.4237	205.7416
8	2D	AX-111134328	0.4237	205.7416
8	2D	AX-109727475	0.4237	205.7416
8	2D	AX-109182806	0.4237	205.7416
8	2D	AX-108783762	0.4237	205.7416
8	2D	AX-109876278	0.4237	205.7416
8	2D	AX-110906716	26.8403	232.5819
8	2D	AX-108827772	0.4255	233.0074
8	2D	AX-111705929	1.5771	234.5845
8	2D	AX-109259942	12.7706	247.3551
8	2D	AX-111382276	12.7706	247.3551
8	2D	AX-110422811	1.7551	249.1102
8	2D	AX-110041364	0.211	249.3212
8	2D	AX-89696347	0.8585	250.1797
8	2D	AX-110484083	19.797	269.9767
8	2D	AX-110075544	19.797	269.9767
9	2D	AX-108976644	0	0
9	2D	AX-108887461	0	0
9	2D	AX-109371788	20.8979	20.8979
9	2D	AX-111020675	20.8979	20.8979
9	2D	AX-109436151	20.8979	20.8979
9	2D	AX-108940544	20.8979	20.8979
9	2D	AX-110461477	3.3532	24.2512
9	2D	AX-110387848	3.3532	24.2512
9	2D	AX-109577269	3.3532	24.2512

9	2D	AX-111487816	3.3532	24.2512
9	2D	AX-109825368	3.3532	24.2512
9	2D	AX-111576943	3.1013	27.3525
9	2D	AX-112289924	0.211	27.5635
9	2D	AX-111571339	0.6356	28.1991
9	2D	AX-111147948	0.6356	28.1991
9	2D	AX-108939876	0.6356	28.1991
9	2D	AX-110068988	0.6383	28.8374
9	2D	AX-111123423	0.8585	29.6959
9	2D	AX-110976029	0.8585	29.6959
9	2D	AX-108851541	0.8585	29.6959
9	2D	AX-109389978	0.8585	29.6959
9	2D	AX-109876288	0.8585	29.6959
9	2D	AX-110385746	0.8585	29.6959
9	2D	AX-110963024	0.8585	29.6959
9	2D	AX-109359061	0.8585	29.6959
9	2D	AX-110940091	0.8585	29.6959
9	2D	AX-94506151	0.8585	29.6959
9	2D	AX-110983186	5.6578	35.3537
9	2D	AX-110430269	5.6578	35.3537
9	2D	AX-94593560	5.6578	35.3537
9	2D	AX-110051842	10.6159	45.9696
9	2D	AX-109414589	3.3532	49.3228
9	2D	AX-109913269	7.37	56.6928
9	2D	AX-110620117	4.3488	61.0416
10	3A	AX-108832448	0	0
10	3A	AX-111212311	6.7066	6.7066
10	3A	AX-110451043	2.634	9.3406
10	3A	AX-109959182	2.634	9.3406
10	3A	AX-110038636	0.2083	9.5489
10	3A	AX-109877370	0.2083	9.5489
10	3A	AX-111656687	0.2083	9.5489
10	3A	AX-108787734	0.844	10.3929
10	3A	AX-109307216	0.844	10.3929
10	3A	AX-111070416	1.949	12.3419
10	3A	AX-109465246	1.949	12.3419
10	3A	AX-109354760	1.949	12.3419
10	3A	AX-110379411	1.949	12.3419
10	3A	AX-110687537	1.949	12.3419
10	3A	AX-111241198	1.949	12.3419
10	3A	AX-108866571	1.949	12.3419
10	3A	AX-111682547	1.949	12.3419
10	3A	AX-109815587	1.949	12.3419
10	3A	AX-108889075	1.949	12.3419

10	3A	AX-111129296	1.5222	13.8642
10	3A	AX-110587728	1.0778	14.9419
10	3A	AX-109397875	1.949	16.891
10	3A	AX-111802923	1.949	16.891
10	3A	AX-111636702	1.949	16.891
10	3A	AX-111153975	2.634	19.525
10	3A	AX-109407758	2.634	19.525
10	3A	AX-109968860	2.634	19.525
10	3A	AX-111558727	2.634	19.525
10	3A	AX-111495688	2.634	19.525
10	3A	AX-111613775	0.2092	19.7342
10	3A	AX-108833841	2.1848	21.919
10	3A	AX-111138337	0.6303	22.5493
10	3A	AX-111449972	0.6303	22.5493
10	3A	AX-111766017	1.064	23.6132
10	3A	AX-108954219	2.1753	25.7885
10	3A	AX-109286748	1.949	27.7376
10	3A	AX-110612410	1.949	27.7376
10	3A	AX-109393734	1.949	27.7376
10	3A	AX-108827798	1.949	27.7376
10	3A	AX-109622330	1.949	27.7376
10	3A	AX-109971543	1.949	27.7376
10	3A	AX-108793165	1.949	27.7376
10	3A	AX-111095542	1.949	27.7376
10	3A	AX-111633510	1.949	27.7376
10	3A	AX-111035296	1.949	27.7376
10	3A	AX-110127212	0.4202	28.1577
10	3A	AX-108803731	0.4202	28.1577
10	3A	AX-109584722	0.4202	28.1577
10	3A	AX-110374052	0.4202	28.1577
10	3A	AX-109378967	0.4202	28.1577
10	3A	AX-110729949	0.4202	28.1577
10	3A	AX-111058659	0.4202	28.1577
10	3A	AX-110620789	0.4202	28.1577
10	3A	AX-110531758	0.4202	28.1577
10	3A	AX-108885585	0.4202	28.1577
10	3A	AX-111235554	0.4202	28.1577
10	3A	AX-109456169	0.4202	28.1577
10	3A	AX-111576314	0.4202	28.1577
10	3A	AX-110984933	0.4202	28.1577
10	3A	AX-111451084	0.211	28.3687
10	3A	AX-108881921	0.2101	28.5788
10	3A	AX-111792223	0.6356	29.2144
10	3A	AX-111488679	1.7323	30.9467

10	3A	AX-108932575	0.6383	31.5851
10	3A	AX-109281389	0.6383	31.5851
10	3A	AX-110967458	0.422	32.007
10	3A	AX-89333265	0.422	32.007
10	3A	AX-110634761	0.422	32.007
10	3A	AX-89466149	0.2092	32.2162
10	3A	AX-109956153	0.2092	32.2162
10	3A	AX-109285371	0.6329	32.8492
10	3A	AX-110913950	0.6303	33.4795
10	3A	AX-94399040	0.6303	33.4795
10	3A	AX-111161246	0.4202	33.8996
10	3A	AX-109392538	4.8765	38.7761
10	3A	AX-110996461	4.8765	38.7761
10	3A	AX-111053837	4.8765	38.7761
10	3A	AX-109589492	4.8765	38.7761
10	3A	AX-111602190	4.8765	38.7761
10	3A	AX-109844195	1.9661	40.7422
10	3A	AX-111074009	13.1278	53.87
10	3A	AX-111048271	13.1278	53.87
10	3A	AX-109417164	13.1278	53.87
10	3A	AX-109334040	13.1278	53.87
10	3A	AX-109425107	13.1278	53.87
10	3A	AX-109994592	13.1278	53.87
10	3A	AX-111122043	13.1278	53.87
10	3A	AX-109422028	13.1278	53.87
10	3A	AX-110565823	13.1278	53.87
10	3A	AX-110480148	13.1278	53.87
10	3A	AX-111739748	0.2101	54.0801
10	3A	AX-111666570	0.2101	54.0801
10	3A	AX-111653361	0.2101	54.0801
10	3A	AX-110520445	0.6411	54.7212
10	3A	AX-109103063	0.6411	55.3622
10	3A	AX-111082491	0.6411	55.3622
10	3A	AX-110049779	0.6411	55.3622
10	3A	AX-111611367	0.6411	55.3622
10	3A	AX-109350657	0.6411	55.3622
10	3A	AX-111799835	2.2041	57.5663
10	3A	AX-109274841	7.6957	65.262
10	3A	AX-109293721	5.1828	70.4448
10	3A	AX-86183848	6.7066	77.1514
10	3A	AX-110016134	6.7066	77.1514
10	3A	AX-109580901	0.4237	77.5751
10	3A	AX-111802859	0.2128	77.7879
10	3A	AX-111688182	0.2128	77.7879

10	3A	AX-111463818	0.2128	78.0006
10	3A	AX-111211642	0.2128	78.0006
10	3A	AX-111104789	0.2128	78.0006
10	3A	AX-111051542	0.2128	78.0006
10	3A	AX-111761529	0.2128	78.0006
10	3A	AX-111532230	0.2128	78.0006
10	3A	AX-111524586	0.6356	78.6363
10	3A	AX-111468754	0.6356	78.6363
10	3A	AX-110401758	0.6356	78.6363
10	3A	AX-110670767	0.6356	78.6363
10	3A	AX-109440744	0.6356	78.6363
10	3A	AX-111591696	0.422	79.0582
10	3A	AX-89497626	0.422	79.0582
10	3A	AX-108829349	0.422	79.0582
10	3A	AX-108783249	0.2119	79.2701
10	3A	AX-110001246	0.2119	79.2701
10	3A	AX-111612053	0.211	79.481
10	3A	AX-109839765	0.211	79.481
10	3A	AX-110069195	0.211	79.481
10	3A	AX-110427771	0.211	79.481
10	3A	AX-110997460	0.211	79.481
10	3A	AX-111490400	0.211	79.481
10	3A	AX-111101000	0.211	79.481
10	3A	AX-109901092	0.211	79.481
10	3A	AX-110003002	0.211	79.481
10	3A	AX-108807052	0.211	79.481
10	3A	AX-111217388	0.211	79.481
10	3A	AX-109035069	0.211	79.481
10	3A	AX-111760062	0.211	79.481
10	3A	AX-109296862	0.2101	79.6911
10	3A	AX-110371489	0.4255	80.1167
10	3A	AX-110052738	0.2119	80.3285
10	3A	AX-110437454	0.6356	80.9642
10	3A	AX-111734585	0.6356	80.9642
10	3A	AX-109035969	0.2092	81.1734
10	3A	AX-108885694	0.2092	81.1734
10	3A	AX-109905422	0.2092	81.1734
10	3A	AX-111554595	0.2092	81.1734
10	3A	AX-110474919	0.2092	81.1734
10	3A	AX-109904743	0.2092	81.1734
10	3A	AX-109452897	0.2092	81.1734
10	3A	AX-110611074	0.2092	81.1734
10	3A	AX-109957890	0.2092	81.1734
10	3A	AX-111538534	0.2092	81.1734

10	3A	AX-110452812	0.2092	81.1734
10	3A	AX-111125073	0.2092	81.1734
10	3A	AX-110441621	0.2092	81.1734
10	3A	AX-109971973	0.2092	81.1734
10	3A	AX-95011264	0.2092	81.1734
10	3A	AX-110489629	0.4202	81.5935
10	3A	AX-110422245	0.4202	81.5935
10	3A	AX-110475164	0.4202	81.5935
10	3A	AX-89348435	0.4202	81.5935
10	3A	AX-111045060	0.4202	81.5935
10	3A	AX-109875023	0.4202	81.5935
10	3A	AX-109295136	0.4202	81.5935
10	3A	AX-109936048	0.4202	81.5935
10	3A	AX-109483543	0.4202	81.5935
10	3A	AX-109817800	0.4202	81.5935
10	3A	AX-111168955	0.4202	81.5935
10	3A	AX-110954210	0.4202	81.5935
10	3A	AX-109879188	0.4202	81.5935
10	3A	AX-111091518	0.4202	81.5935
10	3A	AX-109845445	0.4202	81.5935
10	3A	AX-110512523	0.4202	81.5935
10	3A	AX-111505762	0.4202	81.5935
10	3A	AX-108803621	0.4202	81.5935
10	3A	AX-109408044	0.4202	81.5935
10	3A	AX-108758496	0.4202	81.5935
10	3A	AX-109528875	0.4202	81.5935
10	3A	AX-109482628	0.4202	81.5935
10	3A	AX-109885501	0.4202	81.5935
10	3A	AX-109442780	0.4202	81.5935
10	3A	AX-86170848	0.4202	81.5935
10	3A	AX-110393239	0.4202	81.5935
10	3A	AX-110735133	0.4202	81.5935
10	3A	AX-111575718	0.4202	81.5935
10	3A	AX-109378241	0.4202	81.5935
10	3A	AX-110122182	0.4202	81.5935
10	3A	AX-111564924	0.4202	81.5935
10	3A	AX-108923506	0.4202	81.5935
10	3A	AX-110372846	0.4202	81.5935
10	3A	AX-110736133	0.4202	81.5935
10	3A	AX-109839982	0.4202	81.5935
10	3A	AX-109863037	0.4202	81.5935
10	3A	AX-109868656	0.4202	81.5935
10	3A	AX-110688259	0.4202	81.5935
10	3A	AX-108863292	0.4202	81.5935

10	3A	AX-109908411	0.4202	81.5935
10	3A	AX-108726616	0.4202	81.5935
10	3A	AX-111551307	0.4202	81.5935
10	3A	AX-110985936	0.4202	81.5935
10	3A	AX-108942438	0.4202	81.5935
10	3A	AX-111096752	0.4202	81.5935
10	3A	AX-108947258	0.4202	81.5935
10	3A	AX-108934431	0.4202	81.5935
10	3A	AX-111074800	0.4202	81.5935
10	3A	AX-111136569	0.4202	81.5935
10	3A	AX-108779523	0.4202	81.5935
10	3A	AX-110578732	0.4202	81.5935
10	3A	AX-111778608	0.4202	81.5935
10	3A	AX-108965832	0.4202	81.5935
10	3A	AX-110038316	0.4202	81.5935
10	3A	AX-108807007	0.4202	81.5935
10	3A	AX-110005900	0.4202	81.5935
10	3A	AX-108982447	0.4202	81.5935
10	3A	AX-111214616	0.4202	81.5935
10	3A	AX-109991064	0.4202	81.5935
10	3A	AX-109558612	0.4202	81.5935
10	3A	AX-110067819	0.4202	81.5935
10	3A	AX-109439635	0.4202	81.5935
10	3A	AX-110426891	0.4202	81.5935
10	3A	AX-111157535	0.4202	81.5935
10	3A	AX-109924761	0.4202	81.5935
10	3A	AX-89523568	0.4202	81.5935
10	3A	AX-111110553	0.4202	81.5935
10	3A	AX-109596960	0.4202	81.5935
10	3A	AX-108749058	0.4202	81.5935
10	3A	AX-109900165	0.4202	81.5935
10	3A	AX-89650480	0.4202	81.5935
10	3A	AX-108921816	0.4202	81.5935
10	3A	AX-110953100	0.4202	81.5935
10	3A	AX-110965999	0.4202	81.5935
10	3A	AX-110586458	0.4202	81.5935
10	3A	AX-108887003	0.4202	81.5935
10	3A	AX-109302659	0.4202	81.5935
10	3A	AX-110673531	0.4202	81.5935
10	3A	AX-110948185	0.4202	81.5935
10	3A	AX-110364070	0.4202	81.5935
10	3A	AX-110746465	0.4202	81.5935
10	3A	AX-111520556	0.4202	81.5935
10	3A	AX-111685510	0.4202	81.5935

10	3A	AX-110486710	0.4202	81.5935
10	3A	AX-109915920	0.4202	81.5935
10	3A	AX-109885319	0.4202	81.5935
10	3A	AX-109577355	0.4202	81.5935
10	3A	AX-110924505	0.4202	81.5935
10	3A	AX-111290671	0.4202	81.5935
10	3A	AX-110453801	0.4202	81.5935
10	3A	AX-110143218	0.4202	81.5935
10	3A	AX-111235003	0.4202	81.5935
10	3A	AX-110421854	0.4202	81.5935
10	3A	AX-111060245	0.4202	81.5935
10	3A	AX-111517073	0.4202	81.5935
10	3A	AX-108851173	0.4202	81.5935
10	3A	AX-109378201	0.4202	81.5935
10	3A	AX-109315252	0.4202	81.5935
10	3A	AX-108903233	0.4202	81.5935
10	3A	AX-111543244	0.4202	81.5935
10	3A	AX-110436508	0.4202	81.5935
10	3A	AX-111632793	0.4202	81.5935
10	3A	AX-110908981	0.4202	81.5935
10	3A	AX-109279741	0.4202	81.5935
10	3A	AX-110417308	0.4202	81.5935
10	3A	AX-110502824	0.4202	81.5935
10	3A	AX-109495465	0.4202	81.5935
10	3A	AX-111211857	0.4202	81.5935
10	3A	AX-109479343	0.4202	81.5935
10	3A	AX-111001129	0.4202	81.5935
10	3A	AX-109082264	0.4202	81.5935
10	3A	AX-109924523	0.4202	81.5935
10	3A	AX-111592057	0.4202	81.5935
10	3A	AX-111064659	0.4202	81.5935
10	3A	AX-111013594	0.4202	81.5935
10	3A	AX-109924667	0.4202	81.5935
10	3A	AX-111485003	0.4202	81.5935
10	3A	AX-109936929	0.4202	81.5935
10	3A	AX-109294975	0.4202	81.5935
10	3A	AX-109933135	0.4202	81.5935
10	3A	AX-110130005	0.4202	81.5935
10	3A	AX-111555163	0.4202	81.5935
10	3A	AX-108975984	0.4202	81.5935
10	3A	AX-110411691	0.4202	81.5935
10	3A	AX-110926053	0.4202	81.5935
10	3A	AX-111134876	0.4202	81.5935
10	3A	AX-109311933	0.4202	81.5935

10	3A	AX-108952371	0.4202	81.5935
10	3A	AX-109337059	0.4202	81.5935
10	3A	AX-109881647	0.4202	81.5935
10	3A	AX-109943071	0.4202	81.5935
10	3A	AX-109963078	0.4202	81.5935
10	3A	AX-110567176	0.4202	81.5935
10	3A	AX-108969393	0.4202	81.5935
10	3A	AX-110015690	0.4202	81.5935
10	3A	AX-111571396	0.4202	81.5935
10	3A	AX-109958833	0.4202	81.5935
10	3A	AX-108830145	0.4202	81.5935
10	3A	AX-109878195	0.4202	81.5935
10	3A	AX-108800361	0.4202	81.5935
10	3A	AX-109355304	0.4202	81.5935
10	3A	AX-110962885	0.4202	81.5935
10	3A	AX-109401219	0.4202	81.5935
10	3A	AX-110969224	0.4202	81.5935
10	3A	AX-108840867	0.4202	81.5935
10	3A	AX-111218586	0.4202	81.5935
10	3A	AX-94541365	0.4202	81.5935
10	3A	AX-110482168	0.4202	81.5935
10	3A	AX-110941450	0.4202	81.5935
10	3A	AX-109954227	0.4202	81.5935
10	3A	AX-110512340	0.4202	81.5935
10	3A	AX-108895821	0.4202	81.5935
10	3A	AX-110041975	0.4202	81.5935
10	3A	AX-111199773	0.4202	81.5935
10	3A	AX-110040352	0.4202	81.5935
10	3A	AX-108737998	0.4202	81.5935
10	3A	AX-111777043	0.4202	81.5935
10	3A	AX-111572124	0.4202	81.5935
10	3A	AX-111087059	0.4202	81.5935
10	3A	AX-110485469	0.4202	81.5935
10	3A	AX-110485390	0.4202	81.5935
10	3A	AX-109998067	0.4202	81.5935
10	3A	AX-108844656	0.4202	81.5935
10	3A	AX-109285404	0.4202	81.5935
10	3A	AX-109342640	0.4202	81.5935
10	3A	AX-110441365	0.4202	81.5935
10	3A	AX-109501195	0.4202	81.5935
10	3A	AX-109412321	0.4202	81.5935
10	3A	AX-95168777	0.4202	81.5935
10	3A	AX-108869225	0.4202	81.5935
10	3A	AX-109287306	0.4202	81.5935

10	3A	AX-108942559	0.4202	81.5935
10	3A	AX-110012003	0.4202	81.5935
10	3A	AX-110414853	0.4202	81.5935
10	3A	AX-110985242	0.4202	81.5935
10	3A	AX-109390563	0.4202	81.5935
10	3A	AX-109834553	0.4202	81.5935
10	3A	AX-109285480	0.4202	81.5935
10	3A	AX-110539312	0.4202	81.5935
10	3A	AX-110941825	0.4202	81.5935
10	3A	AX-111759901	0.4202	81.5935
10	3A	AX-111487412	0.4202	81.5935
10	3A	AX-111456639	0.4202	81.5935
10	3A	AX-109976177	0.4202	81.5935
10	3A	AX-109476436	0.4202	81.5935
10	3A	AX-108868048	0.4202	81.5935
10	3A	AX-108888023	0.4202	81.5935
10	3A	AX-109332836	0.4202	81.5935
10	3A	AX-109869486	0.4202	81.5935
10	3A	AX-109931935	0.4202	81.5935
10	3A	AX-111056113	0.4202	81.5935
10	3A	AX-111498234	0.4202	81.5935
10	3A	AX-94465976	0.4202	81.5935
10	3A	AX-111451671	0.4202	81.5935
10	3A	AX-95629428	0.4202	81.5935
10	3A	AX-108876721	0.4202	81.5935
10	3A	AX-108955557	0.8475	82.4411
10	3A	AX-94473921	0.8475	82.4411
10	3A	AX-109972641	0.8475	82.4411
10	3A	AX-110455859	0.8475	82.4411
10	3A	AX-109627023	0.8475	82.4411
10	3A	AX-110423324	0.8475	82.4411
10	3A	AX-109584650	0.2092	82.6503
10	3A	AX-109035608	0.2092	82.6503
10	3A	AX-111559124	0.2092	82.6503
10	3A	AX-111569332	0.2092	82.6503
10	3A	AX-109983808	0.2092	82.6503
10	3A	AX-111687892	0.2092	82.6503
10	3A	AX-108827063	0.2092	82.6503
10	3A	AX-111054546	0.2092	82.6503
10	3A	AX-108860378	0.2092	82.6503
10	3A	AX-111454706	1.2878	83.9381
10	3A	AX-108950927	1.2878	83.9381
10	3A	AX-111024665	1.2878	83.9381
10	3A	AX-111008632	1.2878	83.9381

10	3A	AX-108850427	1.5222	85.4603
10	3A	AX-110672868	1.5222	85.4603
10	3A	AX-109973125	1.5222	85.4603
10	3A	AX-108758495	0.2119	85.6722
10	3A	AX-94520713	0.2119	85.6722
10	3A	AX-109942889	0.2119	85.6722
10	3A	AX-108892911	0.4237	86.0959
10	3A	AX-110547087	0.4237	86.0959
10	3A	AX-109351295	0.4237	86.0959
10	3A	AX-109848682	0.4237	86.0959
10	3A	AX-110469543	0.4237	86.0959
10	3A	AX-110423880	0.4237	86.0959
10	3A	AX-111082589	0.4237	86.0959
10	3A	AX-108738069	0.4237	86.0959
10	3A	AX-108840691	0.4237	86.0959
10	3A	AX-109626456	0.4237	86.0959
10	3A	AX-110434299	0.4237	86.0959
10	3A	AX-110631819	0.2092	86.3052
10	3A	AX-109345729	0.2092	86.3052
10	3A	AX-111627621	0.2092	86.3052
10	3A	AX-110675345	0.2092	86.3052
10	3A	AX-111003610	0.4202	86.7253
10	3A	AX-108879785	0.4202	86.7253
10	3A	AX-111067228	0.4202	86.7253
10	3A	AX-111133365	0.4202	86.7253
10	3A	AX-94764371	0.4202	86.7253
10	3A	AX-111786035	0.2092	86.9345
10	3A	AX-109051215	0.2092	86.9345
10	3A	AX-108866756	0.2092	86.9345
10	3A	AX-111065125	0.2092	86.9345
10	3A	AX-109817897	0.2092	86.9345
10	3A	AX-111585689	0.2092	86.9345
10	3A	AX-111450204	0.2092	86.9345
10	3A	AX-110978381	0.2092	86.9345
10	3A	AX-111487970	0.2092	86.9345
10	3A	AX-111743642	0.2092	86.9345
10	3A	AX-111632398	0.2092	86.9345
10	3A	AX-110458041	0.2092	86.9345
10	3A	AX-110392318	0.2092	86.9345
10	3A	AX-109855683	0.2092	86.9345
10	3A	AX-111068094	0.2092	86.9345
10	3A	AX-110508456	0.2092	86.9345
10	3A	AX-109963225	0.2092	86.9345
10	3A	AX-111216862	0.2092	86.9345

10	3A	AX-109392786	0.2092	87.1437
10	3A	AX-110578860	0.2092	87.1437
10	3A	AX-110447030	0.2092	87.1437
10	3A	AX-108736767	0.2092	87.1437
10	3A	AX-89776892	0.2092	87.1437
10	3A	AX-111086171	0.2092	87.1437
10	3A	AX-110192525	0.2092	87.1437
10	3A	AX-109039871	0.2092	87.1437
10	3A	AX-108830725	0.2092	87.1437
10	3A	AX-89748893	0.2092	87.1437
10	3A	AX-109979703	0.2092	87.1437
10	3A	AX-110917484	0.2092	87.1437
10	3A	AX-109612565	0.2092	87.1437
10	3A	AX-109316902	0.2092	87.1437
10	3A	AX-109901738	0.2092	87.1437
10	3A	AX-109830190	0.2092	87.1437
10	3A	AX-109390259	0.2092	87.1437
10	3A	AX-108850482	0.2092	87.1437
10	3A	AX-111551830	0.2092	87.1437
10	3A	AX-108781901	0.2092	87.1437
10	3A	AX-110442313	0.211	87.3547
10	3A	AX-110963001	0.211	87.3547
10	3A	AX-109966493	0.211	87.3547
10	3A	AX-109515010	0.2155	87.5702
10	3A	AX-111498188	0.4367	88.0069
10	3A	AX-110058189	0.4367	88.0069
10	3A	AX-109828373	0.4367	88.0069
10	3A	AX-108913143	0.211	88.2179
10	3A	AX-111522081	0.211	88.2179
10	3A	AX-109654551	0.211	88.2179
10	3A	AX-111211777	0.211	88.2179
10	3A	AX-89763178	0.211	88.2179
10	3A	AX-110578705	0.211	88.2179
10	3A	AX-108881231	0.211	88.2179
10	3A	AX-109865940	0.211	88.2179
10	3A	AX-111091610	0.211	88.2179
10	3A	AX-111033605	0.4202	88.6381
10	3A	AX-111107372	0.6438	89.2819
10	3A	AX-109391941	0.2119	89.4938
10	3A	AX-111540256	0.2119	89.4938
10	3A	AX-110399323	0.2119	89.4938
10	3A	AX-110051666	0.2119	89.4938
10	3A	AX-110953010	0.2119	89.4938
10	3A	AX-109912704	0.2119	89.4938

10	3A	AX-111559142	0.2119	89.4938
10	3A	AX-110904521	0.2119	89.4938
10	3A	AX-109342565	0.2119	89.4938
10	3A	AX-111713473	0.2119	89.4938
10	3A	AX-109284282	0.2092	89.703
10	3A	AX-111475100	0.2092	89.703
10	3A	AX-109992491	0.2092	89.703
10	3A	AX-109910491	0.2092	89.703
10	3A	AX-111233264	0.2092	89.9122
10	3A	AX-110371883	0.2092	89.9122
10	3A	AX-110185298	0.2092	89.9122
10	3A	AX-109923607	0.2092	89.9122
10	3A	AX-94935938	0.2092	89.9122
10	3A	AX-108757118	0.2092	90.1214
10	3A	AX-110969746	0.2092	90.1214
10	3A	AX-109985035	0.2092	90.1214
10	3A	AX-111599886	0.2092	90.1214
10	3A	AX-109545295	0.2092	90.1214
10	3A	AX-111575525	0.2092	90.1214
10	3A	AX-110529370	0.2092	90.1214
10	3A	AX-108837960	0.2092	90.1214
10	3A	AX-109927497	0.2092	90.1214
10	3A	AX-110522868	0.2092	90.1214
10	3A	AX-110624809	0.2092	90.1214
10	3A	AX-110384514	0.2092	90.1214
10	3A	AX-110145801	0.2092	90.1214
10	3A	AX-109078331	0.2092	90.1214
10	3A	AX-108938752	0.2092	90.1214
10	3A	AX-111100488	0.2092	90.1214
10	3A	AX-110415316	0.2092	90.1214
10	3A	AX-111819796	0.2092	90.1214
10	3A	AX-109620716	0.2092	90.1214
10	3A	AX-110481070	0.2092	90.1214
10	3A	AX-109421791	0.2092	90.1214
10	3A	AX-109355730	0.2092	90.1214
10	3A	AX-110042178	0.2092	90.1214
10	3A	AX-110046162	0.2092	90.1214
10	3A	AX-109055131	0.2092	90.1214
10	3A	AX-109461751	0.2092	90.1214
10	3A	AX-109904680	0.2092	90.1214
10	3A	AX-108977066	0.2092	90.1214
10	3A	AX-108925131	0.2092	90.1214
10	3A	AX-111578164	0.2092	90.1214
10	3A	AX-109405061	0.2092	90.1214

10	3A	AX-110594869	0.2092	90.1214
10	3A	AX-89634969	0.2092	90.1214
10	3A	AX-111467215	0.2092	90.1214
10	3A	AX-109997099	0.2092	90.1214
10	3A	AX-109595129	0.2092	90.1214
10	3A	AX-110145693	0.2092	90.1214
10	3A	AX-111041459	0.2092	90.1214
10	3A	AX-111551340	0.2092	90.1214
10	3A	AX-109822868	0.2092	90.1214
10	3A	AX-108852855	0.2092	90.1214
10	3A	AX-109882532	0.2092	90.1214
10	3A	AX-109998040	0.2092	90.1214
10	3A	AX-109298754	0.2092	90.1214
10	3A	AX-110445703	0.2092	90.1214
10	3A	AX-111008215	0.2092	90.1214
10	3A	AX-108861464	0.2092	90.1214
10	3A	AX-110381378	0.2092	90.1214
10	3A	AX-110904729	0.2092	90.1214
10	3A	AX-109443040	0.2101	90.3315
10	3A	AX-111466140	0.2101	90.3315
10	3A	AX-110075296	0.2101	90.3315
10	3A	AX-111079565	0.422	90.7534
10	3A	AX-110382464	0.422	90.7534
10	3A	AX-111068989	0.422	90.7534
10	3A	AX-111625123	0.422	90.7534
10	3A	AX-108898525	0.422	90.7534
10	3A	AX-108890014	0.422	90.7534
10	3A	AX-110495702	0.422	90.7534
10	3A	AX-108921945	0.422	90.7534
10	3A	AX-110448936	0.422	90.7534
10	3A	AX-109382699	0.422	90.7534
10	3A	AX-110013052	0.422	90.7534
10	3A	AX-111602060	0.422	90.7534
10	3A	AX-110566672	0.422	90.7534
10	3A	AX-109060938	0.422	90.7534
10	3A	AX-108849146	0.422	90.7534
10	3A	AX-111052686	0.422	90.7534
10	3A	AX-111460264	0.422	90.7534
10	3A	AX-109053080	0.422	90.7534
10	3A	AX-110534076	0.422	90.7534
10	3A	AX-110414233	0.422	90.7534
10	3A	AX-111164276	0.422	90.7534
10	3A	AX-110534642	0.422	90.7534
10	3A	AX-108907438	0.422	90.7534

10	3A	AX-110171908	0.422	90.7534
10	3A	AX-111698315	0.422	90.7534
10	3A	AX-110602564	0.422	90.7534
10	3A	AX-111653822	0.422	90.7534
10	3A	AX-108728793	0.422	90.7534
10	3A	AX-110409623	0.422	90.7534
10	3A	AX-110591632	0.422	90.7534
10	3A	AX-110365258	0.422	90.7534
10	3A	AX-111072779	0.422	90.7534
10	3A	AX-95632294	0.422	90.7534
10	3A	AX-110383566	0.422	90.7534
10	3A	AX-108774646	0.422	90.7534
10	3A	AX-111569074	0.422	90.7534
10	3A	AX-109431655	0.422	90.7534
10	3A	AX-110949831	0.422	90.7534
10	3A	AX-94599436	0.422	90.7534
10	3A	AX-109499609	0.422	90.7534
10	3A	AX-111100766	0.422	90.7534
10	3A	AX-108729031	0.6329	91.3864
10	3A	AX-108772543	0.6329	91.3864
10	3A	AX-111609261	0.2128	91.5991
10	3A	AX-111087163	0.2128	91.8119
10	3A	AX-109866065	0.2128	91.8119
10	3A	AX-111535511	0.2128	91.8119
10	3A	AX-110122555	0.6356	92.4475
10	3A	AX-110668155	0.6356	92.4475
10	3A	AX-109305297	0.6356	92.4475
10	3A	AX-110005443	0.6356	92.4475
10	3A	AX-108754298	0.6356	92.4475
10	3A	AX-110554752	0.6356	92.4475
10	3A	AX-110925756	0.6356	92.4475
10	3A	AX-111527540	0.6356	92.4475
10	3A	AX-109936405	0.6356	92.4475
10	3A	AX-111591393	0.6356	92.4475
10	3A	AX-109275027	0.6356	92.4475
10	3A	AX-95631882	0.6356	92.4475
10	3A	AX-111690011	0.6356	92.4475
10	3A	AX-110417260	0.6356	92.4475
10	3A	AX-108965032	0.6356	92.4475
10	3A	AX-111643106	0.6356	92.4475
10	3A	AX-86175080	0.6356	92.4475
10	3A	AX-94599381	0.6356	92.4475
10	3A	AX-110385614	2.6456	95.0932
10	3A	AX-109301075	0.4202	95.5133

10	3A	AX-110439700	0.4202	95.5133
10	3A	AX-110558769	0.4202	95.5133
10	3A	AX-111127766	1.5026	97.0159
10	3A	AX-110922897	2.4142	99.4301
10	3A	AX-110951668	2.4142	99.4301
10	3A	AX-111464284	2.4142	99.4301
10	3A	AX-109949596	0.4202	99.8503
10	3A	AX-111037462	0.4202	99.8503
10	3A	AX-110521451	0.4202	99.8503
10	3A	AX-110588651	0.4202	99.8503
10	3A	AX-108783340	0.4202	99.8503
10	3A	AX-109110362	0.4202	99.8503
10	3A	AX-111055674	0.4202	99.8503
10	3A	AX-111507302	0.4202	99.8503
10	3A	AX-111778082	0.4202	99.8503
10	3A	AX-111052976	0.4202	99.8503
10	3A	AX-110713850	0.4202	99.8503
10	3A	AX-111618763	2.2041	102.0543
10	3A	AX-110508416	1.7785	103.8329
10	3A	AX-109649512	3.43	107.2629
10	3A	AX-109509229	2.4464	109.7093
10	3A	AX-111487245	0.8585	110.5677
10	3A	AX-108944536	0.211	110.7787
10	3A	AX-110480551	0.211	110.7787
10	3A	AX-109343667	0.211	110.7787
10	3A	AX-108805602	0.211	110.7787
10	3A	AX-111556286	0.211	110.7787
10	3A	AX-111627361	0.211	110.7787
10	3A	AX-109835767	0.211	110.7787
10	3A	AX-111806403	0.211	110.7787
10	3A	AX-109892060	0.211	110.7787
10	3A	AX-111734511	0.211	110.7787
10	3A	AX-109931570	0.211	110.7787
10	3A	AX-111764361	0.2066	110.9853
10	3A	AX-109270909	0.2066	110.9853
10	3A	AX-108827999	0.2066	110.9853
10	3A	AX-110123295	0.2066	110.9853
10	3A	AX-110571205	0.2066	110.9853
10	3A	AX-111595959	0.2066	110.9853
10	3A	AX-110511174	0.2066	110.9853
10	3A	AX-111521043	0.2066	110.9853
10	3A	AX-95630253	0.2066	110.9853
10	3A	AX-111173842	0.2066	110.9853
10	3A	AX-108766533	0.2066	110.9853

10	3A	AX-89682038	0.6276	111.613
10	3A	AX-111127418	0.2075	111.8204
10	3A	AX-110134737	0.2075	111.8204
10	3A	AX-110365215	0.2075	111.8204
10	3A	AX-109298595	0.4149	112.2354
10	3A	AX-110507631	0.4149	112.2354
10	3A	AX-111056980	0.4149	112.2354
10	3A	AX-109272514	0.4149	112.2354
10	3A	AX-111678200	0.4149	112.2354
10	3A	AX-108838461	0.4149	112.2354
10	3A	AX-110017175	0.4149	112.2354
10	3A	AX-111491294	0.4149	112.2354
10	3A	AX-110514614	0.4149	112.2354
10	3A	AX-111498546	0.4149	112.2354
10	3A	AX-108814307	0.4149	112.6503
10	3A	AX-108814601	0.4149	112.6503
10	3A	AX-109997329	0.4149	112.6503
10	3A	AX-109998276	0.2075	112.8578
10	3A	AX-109035418	0.2075	112.8578
10	3A	AX-109110953	0.2075	112.8578
10	3A	AX-109414232	0.2075	112.8578
10	3A	AX-109867594	0.2075	112.8578
10	3A	AX-109845803	0.2075	112.8578
10	3A	AX-111756960	0.2075	112.8578
10	3A	AX-111564357	0.2075	112.8578
10	3A	AX-110558316	0.2075	112.8578
10	3A	AX-111528212	0.2075	112.8578
10	3A	AX-110979750	0.2075	112.8578
10	3A	AX-111063595	0.2075	112.8578
10	3A	AX-110387354	0.2075	112.8578
10	3A	AX-108871948	0.2075	112.8578
10	3A	AX-111686377	0.2075	112.8578
10	3A	AX-110613113	0.2075	112.8578
10	3A	AX-111462081	0.2075	112.8578
10	3A	AX-86163680	0.2075	112.8578
10	3A	AX-110015968	0.2075	112.8578
10	3A	AX-110988358	0.2075	112.8578
10	3A	AX-110608830	0.2075	112.8578
10	3A	AX-108787762	0.2075	112.8578
10	3A	AX-109647694	0.2075	112.8578
10	3A	AX-109057491	0.2075	112.8578
10	3A	AX-109986495	0.2075	112.8578
10	3A	AX-108898556	0.2075	112.8578
10	3A	AX-109656347	0.2075	112.8578

10	3A	AX-110606671	0.2075	112.8578
10	3A	AX-108954076	0.2075	112.8578
10	3A	AX-111631244	0.2075	112.8578
10	3A	AX-95684139	0.2075	112.8578
10	3A	AX-110920168	0.2075	113.0653
10	3A	AX-108765449	0.2075	113.0653
10	3A	AX-109940148	0.2075	113.0653
10	3A	AX-94858894	0.2075	113.0653
10	3A	AX-110420719	0.2075	113.0653
10	3A	AX-94411903	0.2075	113.0653
10	3A	AX-111459082	0.2075	113.0653
10	3A	AX-110479868	0.2075	113.0653
10	3A	AX-109274942	0.2066	113.2719
10	3A	AX-110953818	0.2075	113.4794
10	3A	AX-111763200	0.2075	113.6868
10	3A	AX-111471151	0.2075	113.6868
10	3A	AX-110962843	1.9323	115.6191
10	3A	AX-111598704	0.4167	116.0358
10	3A	AX-110029105	0.4167	116.0358
10	3A	AX-110512245	0.4167	116.0358
10	3A	AX-111098463	0.4149	116.4508
10	3A	AX-109418113	0.4149	116.4508
10	3A	AX-111090824	0.4149	116.4508
10	3A	AX-110928333	2.5997	119.0505
10	3A	AX-94509782	2.1848	121.2353
10	3A	AX-108732808	2.1848	121.2353
10	3A	AX-110362137	2.1848	121.2353
10	3A	AX-110364206	0.4255	121.6608
10	3A	AX-109870615	0.4255	121.6608
10	3A	AX-109333342	0.4255	121.6608
10	3A	AX-110928526	0.4255	121.6608
10	3A	AX-110399053	0.4255	121.6608
10	3A	AX-109526541	0.4255	121.6608
10	3A	AX-108751869	0.4255	121.6608
10	3A	AX-109956297	0.4255	121.6608
10	3A	AX-110518818	0.4255	121.6608
10	3A	AX-109877809	0.4255	121.6608
10	3A	AX-110941225	0.4255	121.6608
10	3A	AX-109917464	0.4255	121.6608
10	3A	AX-109284498	0.4255	121.6608
10	3A	AX-109855185	0.4255	121.6608
10	3A	AX-111055491	0.4255	121.6608
10	3A	AX-110532671	0.4255	121.6608
10	3A	AX-86174147	0.4255	121.6608

10	3A	AX-109480907	0.4255	121.6608
10	3A	AX-110957923	0.4255	121.6608
10	3A	AX-110486965	0.4255	121.6608
10	3A	AX-110624307	0.4255	121.6608
10	3A	AX-109464655	0.4255	121.6608
10	3A	AX-110975740	0.4255	121.6608
10	3A	AX-110967878	0.4255	121.6608
10	3A	AX-86166063	0.4255	121.6608
10	3A	AX-111142801	0.4255	121.6608
10	3A	AX-108814802	0.4255	121.6608
10	3A	AX-111529655	0.4255	121.6608
10	3A	AX-108910272	0.4255	121.6608
10	3A	AX-110379719	0.4255	121.6608
10	3A	AX-108808975	0.4255	121.6608
10	3A	AX-89599170	0.4255	121.6608
10	3A	AX-111590354	0.4255	121.6608
10	3A	AX-111224300	0.4255	121.6608
10	3A	AX-110378496	0.4255	121.6608
10	3A	AX-110419402	0.4255	121.6608
10	3A	AX-110673749	0.4255	121.6608
10	3A	AX-109522711	0.4255	121.6608
10	3A	AX-108755143	0.4255	121.6608
10	3A	AX-109479904	0.4255	121.6608
10	3A	AX-111471085	0.4255	121.6608
10	3A	AX-110414447	0.4255	121.6608
10	3A	AX-110640222	0.4255	121.6608
10	3A	AX-95661168	0.4255	121.6608
10	3A	AX-86177642	0.4255	121.6608
10	3A	AX-89620558	0.4255	121.6608
10	3A	AX-94763063	0.4255	121.6608
10	3A	AX-94769660	0.4255	121.6608
10	3A	AX-95127670	0.4255	121.6608
10	3A	AX-110039270	0.211	121.8718
10	3A	AX-111482824	0.211	121.8718
10	3A	AX-110441678	0.211	121.8718
10	3A	AX-108861923	0.211	121.8718
10	3A	AX-108732195	0.211	121.8718
10	3A	AX-94782712	0.211	121.8718
10	3A	AX-110932569	0.8475	122.7194
10	3A	AX-110065941	0.8475	122.7194
10	3A	AX-111086224	0.8475	122.7194
10	3A	AX-89470960	0.4255	123.1449
10	3A	AX-110597622	0.2119	123.3568
10	3A	AX-86165946	0.2119	123.3568

10	3A	AX-94655492	0.2119	123.3568
10	3A	AX-108813460	0.211	123.5677
10	3A	AX-109621355	1.3046	124.8724
10	3A	AX-94476007	0.422	125.2943
10	3A	AX-109952086	0.422	125.2943
10	3A	AX-110909814	0.422	125.2943
10	3A	AX-109993347	0.422	125.2943
10	3A	AX-111736201	0.422	125.2943
10	3A	AX-108733790	0.422	125.2943
10	3A	AX-108970620	0.422	125.2943
10	3A	AX-111013595	0.422	125.2943
10	3A	AX-108967828	0.422	125.2943
10	3A	AX-111701437	0.422	125.2943
10	3A	AX-109910042	0.422	125.2943
10	3A	AX-110926255	0.422	125.2943
10	3A	AX-108839822	0.422	125.2943
10	3A	AX-110949316	0.422	125.2943
10	3A	AX-109449932	0.4237	125.7181
10	3A	AX-111045734	0.4237	125.7181
10	3A	AX-109874723	0.4237	125.7181
10	3A	AX-110375681	0.4237	125.7181
10	3A	AX-109449103	0.4237	125.7181
10	3A	AX-110364919	0.4237	125.7181
10	3A	AX-108946027	0.4237	125.7181
10	3A	AX-110394855	0.4237	125.7181
10	3A	AX-109490619	0.4237	125.7181
10	3A	AX-111072580	0.4237	125.7181
10	3A	AX-111252658	0.4237	125.7181
10	3A	AX-110172906	0.4237	125.7181
10	3A	AX-111528638	0.4237	125.7181
10	3A	AX-110373725	0.4237	125.7181
10	3A	AX-111095464	0.4237	125.7181
10	3A	AX-109415896	0.4237	125.7181
10	3A	AX-111216285	0.4237	125.7181
10	3A	AX-109526533	0.4237	125.7181
10	3A	AX-110172746	0.4237	125.7181
10	3A	AX-94713013	0.4237	125.7181
10	3A	AX-109414456	0.4237	125.7181
10	3A	AX-110919471	0.4237	125.7181
10	3A	AX-109354054	0.4237	125.7181
10	3A	AX-111116170	0.4237	125.7181
10	3A	AX-111463786	7.0859	132.804
10	3A	AX-110413216	0.8512	133.6551
10	3A	AX-109415257	0.211	133.8661

10	3A	AX-109344781	0.4255	134.2916
10	3A	AX-110907467	0.2119	134.5035
10	3A	AX-110363397	0.2119	134.5035
10	3A	AX-111761653	0.4274	134.9308
10	3A	AX-111541125	0.4274	134.9308
10	3A	AX-110948863	0.4274	134.9308
10	3A	AX-110016006	12.0713	147.0021
10	3A	AX-109490522	12.0713	147.0021
10	3A	AX-108863620	12.0713	147.0021
10	3A	AX-109387633	9.7164	156.7186
10	3A	AX-110698095	0.211	156.9296
10	3A	AX-110968019	0.211	156.9296
10	3A	AX-110674296	1.5289	158.4584
10	3A	AX-109621685	0.6438	159.1022
10	3A	AX-109351685	0.6438	159.1022
10	3A	AX-109932282	0.6438	159.1022
10	3A	AX-111141273	0.6438	159.1022
10	3A	AX-109874039	0.6438	159.1022
10	3A	AX-111095578	0.6438	159.1022
10	3A	AX-109284407	0.6438	159.1022
10	3A	AX-109280637	0.6438	159.1022
10	3A	AX-110478177	0.6438	159.1022
10	3A	AX-109365022	0.6438	159.1022
10	3A	AX-108823792	0.6438	159.1022
10	3A	AX-110409374	0.8584	159.9607
10	3A	AX-108737367	0.8584	159.9607
10	3A	AX-110186255	0.8584	159.9607
10	3A	AX-110377162	0.8584	159.9607
10	3A	AX-108850899	0.8584	159.9607
10	3A	AX-110066085	0.2128	160.1734
10	3A	AX-109428004	1.5423	161.7158
10	3A	AX-108781745	1.3161	163.0319
10	3A	AX-111106126	1.3161	163.0319
10	3A	AX-110158453	1.3161	163.0319
10	3A	AX-108965218	1.3161	163.0319
10	3A	AX-110433065	1.3161	163.0319
10	3A	AX-109466225	1.5423	164.5742
10	3A	AX-109631089	1.5423	164.5742
10	3A	AX-109357760	1.5423	164.5742
10	3A	AX-108919244	1.5423	164.5742
10	3A	AX-110048194	1.5423	164.5742
10	3A	AX-110081917	1.5423	164.5742
10	3A	AX-109859072	1.5423	164.5742
10	3A	AX-109886693	1.5423	164.5742

10	3A	AX-110432815	1.5423	164.5742
10	3A	AX-110039199	1.5423	164.5742
10	3A	AX-111109340	1.5423	164.5742
10	3A	AX-109497350	1.5423	164.5742
10	3A	AX-109036813	1.5423	164.5742
10	3A	AX-109913456	1.5423	164.5742
10	3A	AX-108973024	1.5423	164.5742
10	3A	AX-108811701	1.5423	164.5742
10	3A	AX-108759720	1.5423	164.5742
10	3A	AX-111470684	1.5423	164.5742
10	3A	AX-111507203	1.5423	164.5742
10	3A	AX-110456639	1.5423	164.5742
10	3A	AX-109460055	1.5423	164.5742
10	3A	AX-109086566	1.5423	164.5742
10	3A	AX-111500231	1.5423	164.5742
10	3A	AX-110929858	1.5423	164.5742
10	3A	AX-110382105	1.5423	164.5742
10	3A	AX-110476552	1.5423	164.5742
10	3A	AX-109867729	1.5423	164.5742
10	3A	AX-110512449	1.5423	164.5742
10	3A	AX-111526427	1.5423	164.5742
10	3A	AX-111572609	1.5423	164.5742
10	3A	AX-108929699	1.5423	164.5742
10	3A	AX-111010138	1.5423	164.5742
10	3A	AX-111719506	1.5423	164.5742
10	3A	AX-110978395	1.5423	164.5742
10	3A	AX-111799298	1.5423	164.5742
10	3A	AX-109828609	5.3866	169.9608
10	3A	AX-111699446	0.2193	170.1801
10	3A	AX-111108822	0.6466	170.8267
10	3A	AX-108729295	3.7105	174.5372
10	3A	AX-108861590	3.7105	174.5372
10	3A	AX-108797523	1.8107	176.3479
10	3A	AX-110395436	6.9408	183.2887
10	3A	AX-110121815	6.9408	183.2887
10	3A	AX-110375888	6.9408	183.2887
10	3A	AX-109951132	6.9408	183.2887
10	3A	AX-110379871	6.9408	183.2887
10	3A	AX-110551308	6.9408	183.2887
10	3A	AX-108907048	6.9408	183.2887
10	3A	AX-109428087	6.9408	183.2887
10	3A	AX-108815173	6.9408	183.2887
10	3A	AX-110957470	6.9408	183.2887
10	3A	AX-108809593	6.9408	183.2887

10	3A	AX-108919606	0.2119	183.5006
10	3A	AX-109371200	0.2119	183.5006
10	3A	AX-111087037	0.2119	183.5006
10	3A	AX-108850461	0.2119	183.5006
10	3A	AX-110402974	0.2119	183.5006
10	3A	AX-110965672	0.2119	183.5006
10	3A	AX-109817782	0.2119	183.5006
10	3A	AX-110982523	0.2119	183.5006
10	3A	AX-110533420	0.2119	183.5006
10	3A	AX-109354334	0.2119	183.5006
10	3A	AX-111587576	0.2119	183.5006
10	3A	AX-111542499	0.2119	183.5006
10	3A	AX-109318483	0.2137	183.7143
10	3A	AX-110479983	0.2137	183.7143
10	3A	AX-111137661	0.2137	183.7143
10	3A	AX-108757676	0.2137	183.7143
10	3A	AX-111701890	0.2137	183.7143
10	3A	AX-109604806	0.2137	183.928
10	3A	AX-110964176	0.2137	183.928
10	3A	AX-111540625	0.2137	184.1417
10	3A	AX-110470707	0.2137	184.1417
10	3A	AX-109311057	0.6438	184.7855
10	3A	AX-94700897	0.6466	185.4321
10	3A	AX-111579209	1.3336	186.7657
10	3A	AX-110390986	0.6494	187.4151
10	3A	AX-108978242	0.6494	187.4151
10	3A	AX-89644172	0.6494	187.4151
10	3A	AX-111005153	0.6494	187.4151
10	3A	AX-110363242	0.6494	187.4151
10	3A	AX-109372178	0.211	187.6261
10	3A	AX-111119192	0.4237	188.0498
10	3A	AX-110468339	0.4237	188.0498
10	3A	AX-109360546	0.4237	188.0498
10	3A	AX-110573441	0.4237	188.0498
10	3A	AX-109417465	0.4237	188.0498
10	3A	AX-108944202	0.4237	188.0498
10	3A	AX-111541089	0.4237	188.0498
10	3A	AX-111450442	0.4237	188.0498
10	3A	AX-111762406	1.9747	190.0245
10	3A	AX-110396446	1.9747	190.0245
10	3A	AX-109406442	1.9747	190.0245
10	3A	AX-109008186	0.6438	190.6683
10	3A	AX-108819775	0.6438	190.6683
10	3A	AX-109486695	0.4274	191.0957

10	3A	AX-111576809	0.8584	191.9541
10	3A	AX-111047459	0.211	192.1651
10	3A	AX-109847054	0.211	192.1651
10	3A	AX-111183935	0.211	192.1651
10	3A	AX-109329110	1.7323	193.8974
10	3A	AX-110007045	1.7323	193.8974
10	3A	AX-108887673	0.6383	194.5357
10	3A	AX-108818126	1.0778	195.6135
10	3A	AX-111101599	1.0778	195.6135
10	3A	AX-108734284	1.0778	195.6135
10	3A	AX-111497717	1.0778	195.6135
10	3A	AX-111642272	1.0778	195.6135
10	3A	AX-110122824	1.0778	195.6135
10	3A	AX-108965336	1.0778	195.6135
10	3A	AX-109513525	1.0778	195.6135
10	3A	AX-108912589	1.0778	195.6135
10	3A	AX-109838994	1.0778	195.6135
10	3A	AX-110492559	1.0778	195.6135
10	3A	AX-111038943	1.0778	195.6135
10	3A	AX-108755294	1.0778	195.6135
10	3A	AX-108957313	1.0778	195.6135
10	3A	AX-109402107	1.0778	195.6135
10	3A	AX-111685619	1.0778	195.6135
10	3A	AX-109882628	1.0778	195.6135
10	3A	AX-109956095	1.0778	195.6135
10	3A	AX-111471436	9.3101	204.9236
10	3A	AX-108726395	9.3101	204.9236
10	3A	AX-109473165	9.3101	204.9236
10	3A	AX-110641862	9.3101	204.9236
10	3A	AX-108850617	9.3101	204.9236
10	3A	AX-110988477	0.2119	205.1355
10	3A	AX-109298605	0.2119	205.3473
10	3A	AX-108891558	0.2128	205.5601
10	3A	AX-111048647	0.2128	205.5601
10	3A	AX-109275090	0.2128	205.5601
10	3A	AX-111534401	0.2128	205.5601
10	3A	AX-110384667	0.2128	205.5601
10	3A	AX-111113527	0.2137	205.7738
10	3A	AX-108873891	0.4292	206.203
10	3A	AX-109314435	13.0609	219.2639
10	3A	AX-109523942	0.2128	219.4767
10	3A	AX-110002525	1.0778	220.5544
10	3A	AX-109964112	0.6383	221.1928
10	3A	AX-111215766	0.6383	221.1928

10	3A	AX-109470830	0.6383	221.1928
10	3A	AX-110374538	0.6383	221.1928
10	3A	AX-110388942	0.6383	221.1928
10	3A	AX-109061253	0.6383	221.1928
10	3A	AX-111014188	0.6356	221.8284
10	3A	AX-111570726	0.6329	222.4613
10	3A	AX-111687227	1.2934	223.7547
11	3B	AX-110365263	0	0
11	3B	AX-108849053	5.9521	5.9521
11	3B	AX-109366463	5.9521	5.9521
11	3B	AX-111127596	0.8622	6.8143
11	3B	AX-110583116	0.2128	7.0271
11	3B	AX-109478402	2.6811	9.7082
11	3B	AX-109000201	1.5561	11.2643
11	3B	AX-109583405	0.6466	11.9108
11	3B	AX-110001564	0.2101	12.1209
11	3B	AX-111121461	0.6438	12.7647
11	3B	AX-109430505	0.8622	13.6269
11	3B	AX-109933033	0.8622	13.6269
11	3B	AX-109445076	0.8622	13.6269
11	3B	AX-110505751	0.8622	13.6269
11	3B	AX-110393614	0.8622	13.6269
11	3B	AX-110926345	0.4274	14.0543
11	3B	AX-109308174	0.4274	14.0543
11	3B	AX-111477581	0.4274	14.0543
11	3B	AX-108805441	0.4274	14.0543
11	3B	AX-110399975	0.6383	14.6926
11	3B	AX-110435611	0.6383	14.6926
11	3B	AX-110625472	0.6383	14.6926
11	3B	AX-89666287	0.6383	14.6926
11	3B	AX-111547083	2.2041	16.8967
11	3B	AX-110909933	2.2041	16.8967
11	3B	AX-109488169	10.672	27.5686
11	3B	AX-110629944	0.2119	27.7805
11	3B	AX-111089329	0.2119	27.7805
11	3B	AX-110386267	0.2119	27.7805
11	3B	AX-111114319	0.2119	27.7805
11	3B	AX-110033392	0.2119	27.7805
11	3B	AX-108761861	0.2119	27.7805
11	3B	AX-109272975	0.2119	27.7805
11	3B	AX-109915590	0.2119	27.7805
11	3B	AX-109892929	0.2119	27.7805
11	3B	AX-109456037	0.2119	27.7805
11	3B	AX-109818685	0.2119	27.7805

11	3B	AX-109406606	0.2119	27.7805
11	3B	AX-110400116	0.2119	27.7805
11	3B	AX-108773562	0.8548	28.6353
11	3B	AX-111134888	0.8548	28.6353
11	3B	AX-109473860	0.8548	28.6353
11	3B	AX-110086057	0.8548	28.6353
11	3B	AX-109521816	0.8548	28.6353
11	3B	AX-109817727	0.8548	28.6353
11	3B	AX-108903511	0.8548	28.6353
11	3B	AX-110119324	0.422	29.0573
11	3B	AX-110029041	0.422	29.0573
11	3B	AX-110926001	0.422	29.0573
11	3B	AX-110499399	0.422	29.0573
11	3B	AX-111510471	0.422	29.0573
11	3B	AX-110402635	0.422	29.0573
11	3B	AX-111531028	0.422	29.0573
11	3B	AX-94853252	0.422	29.0573
11	3B	AX-108974397	1.7323	30.7896
11	3B	AX-109050064	1.949	32.7386
11	3B	AX-109831841	0.2083	32.9469
11	3B	AX-110928522	1.0595	34.0064
11	3B	AX-110050261	1.0595	34.0064
11	3B	AX-110923116	1.5026	35.509
11	3B	AX-109360516	1.5026	35.509
11	3B	AX-110920832	1.5026	35.509
11	3B	AX-110979247	1.5026	35.509
11	3B	AX-109940356	1.5026	35.509
11	3B	AX-111518365	1.5026	35.509
11	3B	AX-111170197	1.5026	35.509
11	3B	AX-110931375	0.8512	36.3602
11	3B	AX-109868743	0.8512	36.3602
11	3B	AX-111509127	5.1342	41.4944
11	3B	AX-109876826	0.4202	41.9146
11	3B	AX-111080011	0.4202	41.9146
11	3B	AX-109910758	0.2101	42.1247
11	3B	AX-110525363	1.2823	43.407
11	3B	AX-89403943	1.2823	43.407
11	3B	AX-111154493	0.2083	43.6153
11	3B	AX-111290314	0.2083	43.6153
11	3B	AX-110495689	0.422	44.0373
11	3B	AX-111040725	0.2101	44.2474
11	3B	AX-109914961	0.4202	44.6675
11	3B	AX-111214708	0.4202	44.6675
11	3B	AX-110402305	0.4202	44.6675

11	3B	AX-108802615	0.4202	44.6675
11	3B	AX-111112609	0.4202	44.6675
11	3B	AX-110978498	0.4202	44.6675
11	3B	AX-111128552	0.4202	44.6675
11	3B	AX-94555292	0.422	45.0895
11	3B	AX-109361439	0.422	45.0895
11	3B	AX-110940471	0.422	45.0895
11	3B	AX-110010083	0.422	45.0895
11	3B	AX-109517771	0.422	45.0895
11	3B	AX-109445929	0.422	45.0895
11	3B	AX-109376235	0.422	45.0895
11	3B	AX-110692697	0.422	45.0895
11	3B	AX-108772336	0.422	45.0895
11	3B	AX-110481138	0.422	45.0895
11	3B	AX-108968612	0.422	45.0895
11	3B	AX-109289037	0.422	45.0895
11	3B	AX-109980037	0.422	45.0895
11	3B	AX-108792106	0.422	45.0895
11	3B	AX-109585543	0.422	45.0895
11	3B	AX-110646335	0.422	45.0895
11	3B	AX-110420501	0.422	45.0895
11	3B	AX-110416923	0.422	45.0895
11	3B	AX-108788611	0.422	45.0895
11	3B	AX-109816573	0.422	45.0895
11	3B	AX-110040585	0.422	45.0895
11	3B	AX-110057613	0.422	45.0895
11	3B	AX-109652025	0.422	45.0895
11	3B	AX-110368451	0.422	45.0895
11	3B	AX-110392375	0.422	45.0895
11	3B	AX-111120784	0.422	45.0895
11	3B	AX-109106230	0.422	45.0895
11	3B	AX-109944644	0.422	45.0895
11	3B	AX-109327486	0.422	45.0895
11	3B	AX-108821658	0.422	45.0895
11	3B	AX-110369850	0.422	45.0895
11	3B	AX-111085224	0.422	45.0895
11	3B	AX-110046410	0.422	45.0895
11	3B	AX-110153348	0.422	45.0895
11	3B	AX-109961453	0.422	45.0895
11	3B	AX-108776238	0.422	45.0895
11	3B	AX-110164603	0.422	45.0895
11	3B	AX-110934652	0.422	45.0895
11	3B	AX-108942316	0.422	45.0895
11	3B	AX-110129931	0.422	45.0895

11	3B	AX-108759684	0.422	45.0895
11	3B	AX-111489536	0.422	45.0895
11	3B	AX-110042136	0.422	45.0895
11	3B	AX-110023853	0.422	45.0895
11	3B	AX-111078759	0.422	45.0895
11	3B	AX-108903094	0.422	45.0895
11	3B	AX-109421815	0.422	45.0895
11	3B	AX-110367089	0.422	45.0895
11	3B	AX-110610514	0.422	45.0895
11	3B	AX-111555111	0.422	45.0895
11	3B	AX-110907745	0.422	45.0895
11	3B	AX-109876637	0.422	45.0895
11	3B	AX-110489162	0.422	45.0895
11	3B	AX-109539881	0.422	45.0895
11	3B	AX-108845040	0.422	45.0895
11	3B	AX-108865914	0.422	45.0895
11	3B	AX-109287109	0.422	45.0895
11	3B	AX-110143353	0.422	45.0895
11	3B	AX-110552610	0.422	45.0895
11	3B	AX-110535477	0.422	45.0895
11	3B	AX-108811309	0.422	45.0895
11	3B	AX-111034690	0.422	45.0895
11	3B	AX-111049794	0.422	45.0895
11	3B	AX-110035964	0.422	45.0895
11	3B	AX-109508801	0.422	45.0895
11	3B	AX-110921730	0.422	45.0895
11	3B	AX-109914938	0.422	45.0895
11	3B	AX-94929224	0.422	45.0895
11	3B	AX-111036129	0.422	45.0895
11	3B	AX-95631336	0.422	45.0895
11	3B	AX-94568436	0.422	45.0895
11	3B	AX-94526408	0.4202	45.5097
11	3B	AX-95223925	0.4202	45.5097
11	3B	AX-111121613	0.4202	45.5097
11	3B	AX-110913143	0.4202	45.5097
11	3B	AX-109330497	0.4202	45.5097
11	3B	AX-109334330	0.4202	45.5097
11	3B	AX-110392716	0.4202	45.5097
11	3B	AX-111460388	0.4202	45.5097
11	3B	AX-111496060	0.4202	45.5097
11	3B	AX-108930935	0.4202	45.5097
11	3B	AX-110045438	0.4202	45.5097
11	3B	AX-110503452	0.4202	45.5097
11	3B	AX-110506853	0.4202	45.5097

11	3B	AX-110001847	0.4202	45.5097
11	3B	AX-109407568	0.4202	45.5097
11	3B	AX-110061413	0.4202	45.5097
11	3B	AX-109988625	0.4202	45.5097
11	3B	AX-109818516	0.4202	45.5097
11	3B	AX-110016120	0.4202	45.5097
11	3B	AX-111231201	0.4202	45.5097
11	3B	AX-111143672	0.4202	45.5097
11	3B	AX-111011153	0.4202	45.5097
11	3B	AX-109955330	0.4202	45.5097
11	3B	AX-110014525	0.4202	45.5097
11	3B	AX-111468261	0.4202	45.5097
11	3B	AX-109411766	0.4202	45.5097
11	3B	AX-110921591	0.4202	45.5097
11	3B	AX-110556599	0.4202	45.5097
11	3B	AX-110512489	0.4202	45.5097
11	3B	AX-111017737	0.4202	45.5097
11	3B	AX-111017923	0.4202	45.5097
11	3B	AX-111002003	0.4202	45.5097
11	3B	AX-109487668	0.4202	45.5097
11	3B	AX-109521332	0.4202	45.5097
11	3B	AX-110380153	0.4202	45.5097
11	3B	AX-110574071	0.4202	45.5097
11	3B	AX-108841942	0.4202	45.5097
11	3B	AX-109359639	0.4202	45.5097
11	3B	AX-109323893	0.4202	45.5097
11	3B	AX-109432323	0.4202	45.5097
11	3B	AX-109898213	0.4202	45.5097
11	3B	AX-111147362	0.4202	45.5097
11	3B	AX-108959853	0.4202	45.5097
11	3B	AX-110985823	0.4202	45.5097
11	3B	AX-108803764	0.4202	45.5097
11	3B	AX-111482942	0.4202	45.5097
11	3B	AX-110730762	0.4202	45.5097
11	3B	AX-109979554	0.4202	45.5097
11	3B	AX-111535296	0.4202	45.5097
11	3B	AX-109859165	0.4202	45.5097
11	3B	AX-111458053	0.4202	45.5097
11	3B	AX-111497180	0.4202	45.5097
11	3B	AX-109338627	0.4202	45.5097
11	3B	AX-108855009	0.4202	45.5097
11	3B	AX-109416500	0.4202	45.5097
11	3B	AX-109506024	0.4202	45.5097
11	3B	AX-110401384	0.4202	45.5097

11	3B	AX-109395116	0.4202	45.5097
11	3B	AX-110006460	0.4202	45.5097
11	3B	AX-111230957	0.4202	45.5097
11	3B	AX-110675221	0.4202	45.5097
11	3B	AX-108965410	0.4202	45.5097
11	3B	AX-111529871	0.4202	45.5097
11	3B	AX-109840450	0.4202	45.5097
11	3B	AX-110007345	0.4202	45.5097
11	3B	AX-110563639	0.4202	45.5097
11	3B	AX-111236299	0.4202	45.5097
11	3B	AX-109941419	0.4202	45.5097
11	3B	AX-109422148	0.4202	45.5097
11	3B	AX-109281372	0.4202	45.5097
11	3B	AX-109523441	0.4202	45.5097
11	3B	AX-109466306	0.4202	45.5097
11	3B	AX-110927165	0.4202	45.5097
11	3B	AX-110485573	0.4202	45.5097
11	3B	AX-111047357	0.4202	45.5097
11	3B	AX-111490441	0.4202	45.5097
11	3B	AX-108774135	0.4202	45.5097
11	3B	AX-110545203	0.4202	45.5097
11	3B	AX-110603468	0.4202	45.5097
11	3B	AX-109975998	0.4202	45.5097
11	3B	AX-94972165	0.4202	45.5097
11	3B	AX-95016211	0.4202	45.5097
11	3B	AX-95659378	0.4202	45.5097
11	3B	AX-110043431	0.2092	45.7189
11	3B	AX-110928043	0.2092	45.7189
11	3B	AX-111579849	0.2092	45.7189
11	3B	AX-94686079	0.2092	45.7189
11	3B	AX-111201366	0.2092	45.7189
11	3B	AX-110458083	0.8773	46.5962
11	3B	AX-109870033	0.8811	47.4773
11	3B	AX-109946276	0.8811	47.4773
11	3B	AX-110595716	0.6411	48.1184
11	3B	AX-108952508	0.6411	48.1184
11	3B	AX-109035807	0.6411	48.1184
11	3B	AX-111516935	0.6411	48.1184
11	3B	AX-111027983	0.4255	48.5439
11	3B	AX-110129538	0.4255	48.5439
11	3B	AX-94695933	0.4237	48.9676
11	3B	AX-108812052	0.4237	48.9676
11	3B	AX-94784828	0.4237	48.9676
11	3B	AX-109823534	0.4237	48.9676

11	3B	AX-110931130	0.4237	48.9676
11	3B	AX-111474329	0.4237	48.9676
11	3B	AX-109456714	0.4237	48.9676
11	3B	AX-108781175	0.4237	48.9676
11	3B	AX-111182805	0.4237	48.9676
11	3B	AX-109576499	0.4237	48.9676
11	3B	AX-110126647	0.4237	48.9676
11	3B	AX-108742032	0.4237	48.9676
11	3B	AX-111037456	0.4237	48.9676
11	3B	AX-109321465	0.4237	48.9676
11	3B	AX-111126803	0.4237	48.9676
11	3B	AX-109982848	0.4237	48.9676
11	3B	AX-110529388	0.4237	48.9676
11	3B	AX-110399054	0.4237	48.9676
11	3B	AX-109425524	0.4237	48.9676
11	3B	AX-110532165	0.4237	48.9676
11	3B	AX-109444338	0.4237	48.9676
11	3B	AX-109336043	0.4237	48.9676
11	3B	AX-110469505	0.4237	48.9676
11	3B	AX-110567159	0.4237	48.9676
11	3B	AX-110417705	0.4237	48.9676
11	3B	AX-109516173	0.4237	48.9676
11	3B	AX-111220100	0.4237	48.9676
11	3B	AX-108836743	0.4237	48.9676
11	3B	AX-109353769	0.4237	48.9676
11	3B	AX-111450635	0.4237	48.9676
11	3B	AX-110053617	0.4237	48.9676
11	3B	AX-109487828	0.4237	48.9676
11	3B	AX-111489807	0.4237	48.9676
11	3B	AX-111230323	0.4237	48.9676
11	3B	AX-111039785	0.4237	48.9676
11	3B	AX-111082807	0.4237	48.9676
11	3B	AX-108811119	0.4237	48.9676
11	3B	AX-109500334	0.4237	48.9676
11	3B	AX-109885946	0.4237	48.9676
11	3B	AX-110969574	0.4237	48.9676
11	3B	AX-109850126	0.4237	48.9676
11	3B	AX-108929619	0.4237	48.9676
11	3B	AX-108769886	0.4237	48.9676
11	3B	AX-110960588	0.4237	48.9676
11	3B	AX-110691897	0.4237	48.9676
11	3B	AX-108770644	0.4237	48.9676
11	3B	AX-108748429	0.4237	48.9676
11	3B	AX-110409674	0.4237	48.9676

11	3B	AX-109958603	0.4237	48.9676
11	3B	AX-108939150	0.4237	48.9676
11	3B	AX-111126495	0.4237	48.9676
11	3B	AX-111005371	0.4237	48.9676
11	3B	AX-111043275	0.4237	48.9676
11	3B	AX-111087735	0.4237	48.9676
11	3B	AX-109975369	0.4237	48.9676
11	3B	AX-110981721	0.4237	48.9676
11	3B	AX-109289396	0.4237	48.9676
11	3B	AX-109304063	0.4237	48.9676
11	3B	AX-109053338	0.4237	48.9676
11	3B	AX-110125172	0.4237	48.9676
11	3B	AX-111095983	0.4237	48.9676
11	3B	AX-110578964	0.4237	48.9676
11	3B	AX-110057698	0.4237	48.9676
11	3B	AX-108853995	0.4237	48.9676
11	3B	AX-109941178	0.4237	48.9676
11	3B	AX-111466951	0.4237	48.9676
11	3B	AX-110408411	0.4237	48.9676
11	3B	AX-110632321	0.4237	48.9676
11	3B	AX-111095124	0.4237	48.9676
11	3B	AX-109989549	0.4237	48.9676
11	3B	AX-111520472	0.4237	48.9676
11	3B	AX-109998246	0.4237	48.9676
11	3B	AX-111005986	0.4237	48.9676
11	3B	AX-110587212	0.4237	48.9676
11	3B	AX-109933220	0.4237	48.9676
11	3B	AX-108959409	0.4237	48.9676
11	3B	AX-110194202	0.4237	48.9676
11	3B	AX-111018348	0.4237	48.9676
11	3B	AX-109060963	0.4237	48.9676
11	3B	AX-111212296	0.4237	48.9676
11	3B	AX-111140183	0.4237	48.9676
11	3B	AX-109362087	0.4237	48.9676
11	3B	AX-109505504	0.4237	48.9676
11	3B	AX-109348047	0.4237	48.9676
11	3B	AX-110947001	0.4237	48.9676
11	3B	AX-111217864	0.4237	48.9676
11	3B	AX-109936276	0.4237	48.9676
11	3B	AX-109052327	0.4237	48.9676
11	3B	AX-109351273	0.4237	48.9676
11	3B	AX-108785727	0.4237	48.9676
11	3B	AX-110610867	0.4237	48.9676
11	3B	AX-108936649	0.4237	48.9676

11	3B	AX-110027914	0.4237	48.9676
11	3B	AX-109900809	0.4237	48.9676
11	3B	AX-109941614	0.4237	48.9676
11	3B	AX-110506327	0.4237	48.9676
11	3B	AX-109371379	0.4237	48.9676
11	3B	AX-109101572	0.4237	48.9676
11	3B	AX-111536646	0.4237	48.9676
11	3B	AX-111520325	0.4237	48.9676
11	3B	AX-110365018	0.4237	48.9676
11	3B	AX-108877020	0.4237	48.9676
11	3B	AX-111467893	0.4237	48.9676
11	3B	AX-110010702	0.4237	48.9676
11	3B	AX-110936863	0.4237	48.9676
11	3B	AX-109923863	0.4237	48.9676
11	3B	AX-111156957	0.4237	48.9676
11	3B	AX-109905953	0.4237	48.9676
11	3B	AX-109868685	0.4237	48.9676
11	3B	AX-110703410	0.4237	48.9676
11	3B	AX-109900683	0.4237	48.9676
11	3B	AX-109037819	0.4237	48.9676
11	3B	AX-109516728	0.4237	48.9676
11	3B	AX-108895898	0.4237	48.9676
11	3B	AX-109597708	0.4237	48.9676
11	3B	AX-110457630	0.4237	48.9676
11	3B	AX-109972139	0.4237	48.9676
11	3B	AX-111222212	0.4237	48.9676
11	3B	AX-109343842	0.4237	48.9676
11	3B	AX-110985949	0.4237	48.9676
11	3B	AX-111496597	0.4237	48.9676
11	3B	AX-109373836	0.4237	48.9676
11	3B	AX-111005286	0.4237	48.9676
11	3B	AX-109054847	0.4237	48.9676
11	3B	AX-109074171	0.4237	48.9676
11	3B	AX-109403706	0.4237	48.9676
11	3B	AX-110122476	0.4237	48.9676
11	3B	AX-110042506	0.4237	48.9676
11	3B	AX-109330351	0.4237	48.9676
11	3B	AX-111139744	0.4237	48.9676
11	3B	AX-109860276	0.4237	48.9676
11	3B	AX-111069058	0.4237	48.9676
11	3B	AX-110667055	0.4237	48.9676
11	3B	AX-111065057	0.4237	48.9676
11	3B	AX-110937713	0.4237	48.9676
11	3B	AX-110504986	0.4237	48.9676

11	3B	AX-109429993	0.4237	48.9676
11	3B	AX-110010332	0.4237	48.9676
11	3B	AX-109930776	0.4237	48.9676
11	3B	AX-109041172	0.4237	48.9676
11	3B	AX-108978268	0.4237	48.9676
11	3B	AX-109859344	0.4237	48.9676
11	3B	AX-109468595	0.4237	48.9676
11	3B	AX-110950334	0.4237	48.9676
11	3B	AX-108770824	0.4237	48.9676
11	3B	AX-108780104	0.4237	48.9676
11	3B	AX-109830170	0.4237	48.9676
11	3B	AX-110948009	0.4237	48.9676
11	3B	AX-109354761	0.4237	48.9676
11	3B	AX-110745662	0.4237	48.9676
11	3B	AX-110920908	0.4237	48.9676
11	3B	AX-108890482	0.4237	48.9676
11	3B	AX-109487230	0.4237	48.9676
11	3B	AX-110998126	0.4237	48.9676
11	3B	AX-111455185	0.4237	48.9676
11	3B	AX-109359680	0.4237	48.9676
11	3B	AX-110995995	0.4237	48.9676
11	3B	AX-109966349	0.4237	48.9676
11	3B	AX-110537088	0.4237	48.9676
11	3B	AX-110519257	0.4237	48.9676
11	3B	AX-111078172	0.4237	48.9676
11	3B	AX-109962929	0.4237	48.9676
11	3B	AX-108838258	0.4237	48.9676
11	3B	AX-110736452	0.4237	48.9676
11	3B	AX-111100099	0.4237	48.9676
11	3B	AX-111048049	0.4237	48.9676
11	3B	AX-109988611	0.4237	48.9676
11	3B	AX-109872345	0.4237	48.9676
11	3B	AX-110685688	0.4237	48.9676
11	3B	AX-111068805	0.4237	48.9676
11	3B	AX-110582482	0.4237	48.9676
11	3B	AX-108944245	0.4237	48.9676
11	3B	AX-109837828	0.4237	48.9676
11	3B	AX-110477245	0.4237	48.9676
11	3B	AX-109439087	0.4237	48.9676
11	3B	AX-109858753	0.4237	48.9676
11	3B	AX-110731274	0.4237	48.9676
11	3B	AX-109056163	0.4237	48.9676
11	3B	AX-111250295	0.4237	48.9676
11	3B	AX-110596546	0.4237	48.9676

11	3B	AX-109480581	0.4237	48.9676
11	3B	AX-111154295	0.4237	48.9676
11	3B	AX-111564611	0.4237	48.9676
11	3B	AX-109851888	0.4237	48.9676
11	3B	AX-109857534	0.4237	48.9676
11	3B	AX-109390753	0.4237	48.9676
11	3B	AX-108793109	0.4237	48.9676
11	3B	AX-108963257	0.4237	48.9676
11	3B	AX-108857214	0.4237	48.9676
11	3B	AX-108838550	0.4237	48.9676
11	3B	AX-110925227	0.4237	48.9676
11	3B	AX-109031417	0.4237	48.9676
11	3B	AX-108765398	0.4237	48.9676
11	3B	AX-108888746	0.4237	48.9676
11	3B	AX-111472588	0.4237	48.9676
11	3B	AX-111011393	0.4237	48.9676
11	3B	AX-110452912	0.4237	48.9676
11	3B	AX-111571837	0.4237	48.9676
11	3B	AX-108754456	0.4237	48.9676
11	3B	AX-111575709	0.4237	48.9676
11	3B	AX-110577655	0.4237	48.9676
11	3B	AX-110712487	0.4237	48.9676
11	3B	AX-111065820	0.4237	48.9676
11	3B	AX-111039597	0.4237	48.9676
11	3B	AX-108951729	0.4237	48.9676
11	3B	AX-110976494	0.4237	48.9676
11	3B	AX-109857767	0.4237	48.9676
11	3B	AX-108925408	0.4237	48.9676
11	3B	AX-110460782	0.4237	48.9676
11	3B	AX-111241221	0.4237	48.9676
11	3B	AX-109043534	0.4237	48.9676
11	3B	AX-109361035	0.4237	48.9676
11	3B	AX-110170923	0.4237	48.9676
11	3B	AX-110696526	0.4237	48.9676
11	3B	AX-110525576	0.4237	48.9676
11	3B	AX-110482098	0.4237	48.9676
11	3B	AX-109850408	0.4237	48.9676
11	3B	AX-110594860	0.4237	48.9676
11	3B	AX-110919158	0.4237	48.9676
11	3B	AX-111536030	0.4237	48.9676
11	3B	AX-111180833	0.4237	48.9676
11	3B	AX-108852134	0.4237	48.9676
11	3B	AX-108935132	0.4237	48.9676
11	3B	AX-111471417	0.4237	48.9676

11	3B	AX-108976842	0.4237	48.9676
11	3B	AX-110955739	0.4237	48.9676
11	3B	AX-109478632	0.4237	48.9676
11	3B	AX-110591510	0.4237	48.9676
11	3B	AX-111178300	0.4237	48.9676
11	3B	AX-109377767	0.4237	48.9676
11	3B	AX-110396359	0.4237	48.9676
11	3B	AX-108730347	0.4237	48.9676
11	3B	AX-109919819	0.4237	48.9676
11	3B	AX-110083317	0.4237	48.9676
11	3B	AX-110417233	0.4237	48.9676
11	3B	AX-109884780	0.4237	48.9676
11	3B	AX-109996559	0.4237	48.9676
11	3B	AX-109434649	0.4237	48.9676
11	3B	AX-109452498	0.4237	48.9676
11	3B	AX-110913046	0.4237	48.9676
11	3B	AX-110985979	0.4237	48.9676
11	3B	AX-111536904	0.4237	48.9676
11	3B	AX-108949528	0.4237	48.9676
11	3B	AX-111543187	0.4237	48.9676
11	3B	AX-111569625	0.4237	48.9676
11	3B	AX-109451396	0.4237	48.9676
11	3B	AX-110547011	0.4237	48.9676
11	3B	AX-110545804	0.4237	48.9676
11	3B	AX-111181384	0.4237	48.9676
11	3B	AX-111534040	0.4237	48.9676
11	3B	AX-109639680	0.4237	48.9676
11	3B	AX-110004359	0.4237	48.9676
11	3B	AX-109909251	0.4237	48.9676
11	3B	AX-110977565	0.4237	48.9676
11	3B	AX-111043221	0.4237	48.9676
11	3B	AX-111496055	0.4237	48.9676
11	3B	AX-109103577	0.4237	48.9676
11	3B	AX-109416426	0.4237	48.9676
11	3B	AX-109348139	0.4237	48.9676
11	3B	AX-108815378	0.4237	48.9676
11	3B	AX-111169677	0.4237	48.9676
11	3B	AX-109843429	0.4237	48.9676
11	3B	AX-109283006	0.4237	48.9676
11	3B	AX-108773853	0.4237	48.9676
11	3B	AX-110402355	0.4237	48.9676
11	3B	AX-110138500	0.4237	48.9676
11	3B	AX-108949563	0.4237	48.9676
11	3B	AX-110631996	0.4237	48.9676

11	3B	AX-108865513	0.4237	48.9676
11	3B	AX-110924792	0.4237	48.9676
11	3B	AX-109509459	0.4237	48.9676
11	3B	AX-111021820	0.4237	48.9676
11	3B	AX-110512664	0.4237	48.9676
11	3B	AX-108854568	0.4237	48.9676
11	3B	AX-111217095	0.4237	48.9676
11	3B	AX-111568483	0.4237	48.9676
11	3B	AX-109485929	0.4237	48.9676
11	3B	AX-108977600	0.4237	48.9676
11	3B	AX-109282755	0.4237	48.9676
11	3B	AX-110043806	0.4237	48.9676
11	3B	AX-108829779	0.4237	48.9676
11	3B	AX-111028537	0.4237	48.9676
11	3B	AX-110537855	0.4237	48.9676
11	3B	AX-109873916	0.4237	48.9676
11	3B	AX-109309771	0.4237	48.9676
11	3B	AX-111140314	0.4237	48.9676
11	3B	AX-108810996	0.4237	48.9676
11	3B	AX-110367725	0.4237	48.9676
11	3B	AX-109556014	0.4237	48.9676
11	3B	AX-109337179	0.4237	48.9676
11	3B	AX-109501367	0.4237	48.9676
11	3B	AX-111172898	0.4237	48.9676
11	3B	AX-109010691	0.4237	48.9676
11	3B	AX-108736667	0.4237	48.9676
11	3B	AX-108979839	0.4237	48.9676
11	3B	AX-109314274	0.4237	48.9676
11	3B	AX-109954993	0.4237	48.9676
11	3B	AX-111585605	0.4237	48.9676
11	3B	AX-94919989	0.4237	48.9676
11	3B	AX-109333921	0.4237	48.9676
11	3B	AX-110129582	0.4237	48.9676
11	3B	AX-108754665	0.4237	48.9676
11	3B	AX-108823918	0.4237	48.9676
11	3B	AX-108890047	0.4237	48.9676
11	3B	AX-108911331	0.4237	48.9676
11	3B	AX-108915848	0.4237	48.9676
11	3B	AX-108991749	0.4237	48.9676
11	3B	AX-109009643	0.4237	48.9676
11	3B	AX-109397820	0.4237	48.9676
11	3B	AX-109420344	0.4237	48.9676
11	3B	AX-109458458	0.4237	48.9676
11	3B	AX-109536348	0.4237	48.9676

11	3B	AX-109854721	0.4237	48.9676
11	3B	AX-109884461	0.4237	48.9676
11	3B	AX-109923644	0.4237	48.9676
11	3B	AX-109947693	0.4237	48.9676
11	3B	AX-109975984	0.4237	48.9676
11	3B	AX-109991011	0.4237	48.9676
11	3B	AX-110055986	0.4237	48.9676
11	3B	AX-110425431	0.4237	48.9676
11	3B	AX-110426249	0.4237	48.9676
11	3B	AX-110640031	0.4237	48.9676
11	3B	AX-110640082	0.4237	48.9676
11	3B	AX-110663457	0.4237	48.9676
11	3B	AX-110946512	0.4237	48.9676
11	3B	AX-110948028	0.4237	48.9676
11	3B	AX-110951420	0.4237	48.9676
11	3B	AX-110953384	0.4237	48.9676
11	3B	AX-111112415	0.4237	48.9676
11	3B	AX-111116881	0.4237	48.9676
11	3B	AX-111120602	0.4237	48.9676
11	3B	AX-111151072	0.4237	48.9676
11	3B	AX-111164157	0.4237	48.9676
11	3B	AX-111194433	0.4237	48.9676
11	3B	AX-111564447	0.4237	48.9676
11	3B	AX-111585035	0.4237	48.9676
11	3B	AX-111681874	0.4237	48.9676
11	3B	AX-111703903	0.4237	48.9676
11	3B	AX-94546097	0.4237	48.9676
11	3B	AX-94596379	0.4237	48.9676
11	3B	AX-94695984	0.4237	48.9676
11	3B	AX-94744628	0.4237	48.9676
11	3B	AX-94935653	0.4237	48.9676
11	3B	AX-95234083	0.4237	48.9676
11	3B	AX-95634629	0.4237	48.9676
11	3B	AX-110989906	0.4237	48.9676
11	3B	AX-109082779	0.4237	48.9676
11	3B	AX-109958480	0.4237	48.9676
11	3B	AX-110658819	0.4237	48.9676
11	3B	AX-111235812	0.4237	48.9676
11	3B	AX-109875232	0.422	49.3896
11	3B	AX-110017246	0.422	49.3896
11	3B	AX-108731812	0.422	49.3896
11	3B	AX-108799283	0.2083	49.5979
11	3B	AX-109514598	0.2083	49.5979
11	3B	AX-109843703	0.2083	49.5979

11	3B	AX-110976564	0.2083	49.5979
11	3B	AX-108993437	0.2083	49.5979
11	3B	AX-110040152	0.2083	49.5979
11	3B	AX-109436922	0.2083	49.8063
11	3B	AX-110628192	0.2083	49.8063
11	3B	AX-109438175	0.2083	49.8063
11	3B	AX-110046510	0.2083	49.8063
11	3B	AX-110046985	0.2083	49.8063
11	3B	AX-109953325	0.2083	49.8063
11	3B	AX-110988076	0.2083	49.8063
11	3B	AX-111012079	0.2083	49.8063
11	3B	AX-109974785	0.2083	49.8063
11	3B	AX-108737300	0.2083	49.8063
11	3B	AX-110379065	0.2083	49.8063
11	3B	AX-108808483	0.2083	49.8063
11	3B	AX-108820265	0.2083	49.8063
11	3B	AX-108975699	0.2083	49.8063
11	3B	AX-110020100	0.2083	49.8063
11	3B	AX-110537937	0.2083	49.8063
11	3B	AX-110565050	0.4184	50.2247
11	3B	AX-109353708	0.4184	50.6431
11	3B	AX-110942176	0.4184	50.6431
11	3B	AX-110912867	0.4184	50.6431
11	3B	AX-109316650	0.4184	50.6431
11	3B	AX-109949731	0.4184	50.6431
11	3B	AX-109409398	0.4184	50.6431
11	3B	AX-111074064	0.4184	50.6431
11	3B	AX-109922388	0.4184	50.6431
11	3B	AX-108812369	0.4184	50.6431
11	3B	AX-110672966	0.4184	50.6431
11	3B	AX-111567565	0.4184	50.6431
11	3B	AX-109354553	0.4184	50.6431
11	3B	AX-111580302	0.4184	50.6431
11	3B	AX-94918637	0.4184	50.6431
11	3B	AX-95198989	0.4184	50.6431
11	3B	AX-110731139	0.4184	51.0615
11	3B	AX-110417640	0.4184	51.0615
11	3B	AX-109470072	0.4184	51.0615
11	3B	AX-110467038	0.4184	51.0615
11	3B	AX-109980472	0.4184	51.0615
11	3B	AX-108992105	0.6303	51.6918
11	3B	AX-109280251	0.6303	51.6918
11	3B	AX-110479223	0.6303	51.6918
11	3B	AX-110405182	0.6303	51.6918

11	3B	AX-110693349	0.6303	51.6918
11	3B	AX-108859975	0.6303	51.6918
11	3B	AX-111241676	0.6303	51.6918
11	3B	AX-111517231	0.6303	51.6918
11	3B	AX-108856897	0.6303	51.6918
11	3B	AX-109915518	0.6303	51.6918
11	3B	AX-110046844	0.6303	51.6918
11	3B	AX-111092736	0.6303	51.6918
11	3B	AX-110512367	0.6303	51.6918
11	3B	AX-109646382	0.6303	51.6918
11	3B	AX-109107259	0.6303	51.6918
11	3B	AX-109035695	0.6303	51.6918
11	3B	AX-108905095	0.6303	51.6918
11	3B	AX-109465058	0.6303	52.3221
11	3B	AX-109948058	0.2083	52.5304
11	3B	AX-110977295	0.2083	52.5304
11	3B	AX-111460004	0.2083	52.5304
11	3B	AX-108750488	0.2083	52.5304
11	3B	AX-109289484	0.2083	52.5304
11	3B	AX-109616006	0.2083	52.5304
11	3B	AX-110924608	0.2083	52.5304
11	3B	AX-110695608	0.2083	52.5304
11	3B	AX-110390609	0.2083	52.5304
11	3B	AX-109031301	0.2083	52.5304
11	3B	AX-109596657	0.2083	52.5304
11	3B	AX-110616793	0.2083	52.5304
11	3B	AX-109920172	0.2083	52.5304
11	3B	AX-109824459	0.2083	52.5304
11	3B	AX-110456649	0.2083	52.5304
11	3B	AX-109940395	0.2083	52.5304
11	3B	AX-108794413	0.2083	52.5304
11	3B	AX-110555684	0.2083	52.5304
11	3B	AX-111067566	0.2083	52.5304
11	3B	AX-110947273	0.2083	52.5304
11	3B	AX-108761147	0.2083	52.5304
11	3B	AX-108882384	0.2083	52.5304
11	3B	AX-110676695	0.2083	52.5304
11	3B	AX-109477109	0.2083	52.5304
11	3B	AX-108985787	0.2083	52.5304
11	3B	AX-111535675	0.2083	52.5304
11	3B	AX-109486600	0.2083	52.5304
11	3B	AX-110046781	0.2083	52.5304
11	3B	AX-110918860	0.2083	52.5304
11	3B	AX-109271834	0.2083	52.5304

11	3B	AX-109378944	0.2083	52.5304
11	3B	AX-110053956	0.2083	52.5304
11	3B	AX-111557528	0.2083	52.5304
11	3B	AX-108894802	0.2083	52.5304
11	3B	AX-111550253	0.2083	52.5304
11	3B	AX-109486568	0.2083	52.5304
11	3B	AX-110001622	0.2083	52.5304
11	3B	AX-109041122	0.2083	52.5304
11	3B	AX-110375097	0.2083	52.5304
11	3B	AX-109897957	0.2083	52.5304
11	3B	AX-109958594	0.2083	52.5304
11	3B	AX-111464292	0.2083	52.5304
11	3B	AX-111031364	0.2083	52.5304
11	3B	AX-110522947	0.2083	52.5304
11	3B	AX-108864420	0.2083	52.5304
11	3B	AX-110003886	0.2083	52.5304
11	3B	AX-108907758	0.2083	52.5304
11	3B	AX-109331842	0.2083	52.5304
11	3B	AX-110555991	0.2083	52.5304
11	3B	AX-111520322	0.2083	52.5304
11	3B	AX-109900555	0.2083	52.5304
11	3B	AX-109539149	0.2083	52.5304
11	3B	AX-111452618	0.2083	52.5304
11	3B	AX-109315241	0.2083	52.5304
11	3B	AX-110944855	0.2083	52.5304
11	3B	AX-109960445	0.2083	52.5304
11	3B	AX-109363759	0.2083	52.5304
11	3B	AX-108880481	0.2083	52.5304
11	3B	AX-108842651	0.2083	52.5304
11	3B	AX-108863232	0.2083	52.5304
11	3B	AX-109474707	0.2083	52.5304
11	3B	AX-110692065	0.2083	52.5304
11	3B	AX-108965184	0.2083	52.5304
11	3B	AX-109496562	0.2083	52.5304
11	3B	AX-110434901	0.2083	52.5304
11	3B	AX-109312590	0.2083	52.5304
11	3B	AX-109515103	0.2083	52.5304
11	3B	AX-108826347	0.2083	52.5304
11	3B	AX-110446018	0.2083	52.5304
11	3B	AX-110518110	0.2083	52.5304
11	3B	AX-110687552	0.2083	52.5304
11	3B	AX-108859797	0.2083	52.5304
11	3B	AX-111012654	0.2083	52.5304
11	3B	AX-109365701	0.2083	52.5304

11	3B	AX-111483131	0.2083	52.5304
11	3B	AX-108755924	0.2083	52.5304
11	3B	AX-108978295	0.2083	52.5304
11	3B	AX-109319236	0.2083	52.5304
11	3B	AX-109481554	0.2083	52.5304
11	3B	AX-94466684	0.2083	52.5304
11	3B	AX-110377440	0.4184	52.9488
11	3B	AX-110011356	0.4184	52.9488
11	3B	AX-110745038	0.4184	52.9488
11	3B	AX-109279439	0.4184	52.9488
11	3B	AX-109280081	0.2083	53.1572
11	3B	AX-109921414	0.2083	53.1572
11	3B	AX-111520192	0.2083	53.1572
11	3B	AX-111118939	0.2083	53.1572
11	3B	AX-110623371	0.2083	53.1572
11	3B	AX-110590135	0.2083	53.1572
11	3B	AX-108902166	0.2083	53.1572
11	3B	AX-110100415	0.2083	53.1572
11	3B	AX-111134381	0.2083	53.1572
11	3B	AX-110492052	0.2083	53.1572
11	3B	AX-111491044	0.2083	53.1572
11	3B	AX-110675086	0.4167	53.5739
11	3B	AX-110958769	0.4167	53.5739
11	3B	AX-109488286	0.4167	53.5739
11	3B	AX-109014241	0.4167	53.5739
11	3B	AX-110375698	0.8404	54.4143
11	3B	AX-111231131	0.8404	54.4143
11	3B	AX-110907069	0.8404	54.4143
11	3B	AX-110449094	0.8404	54.4143
11	3B	AX-108881477	0.8404	54.4143
11	3B	AX-111039598	0.8404	54.4143
11	3B	AX-111496230	0.8404	54.4143
11	3B	AX-108881958	0.8404	54.4143
11	3B	AX-111108803	0.8404	54.4143
11	3B	AX-110539005	0.8404	54.4143
11	3B	AX-108739060	0.8404	54.4143
11	3B	AX-110501722	0.8404	54.4143
11	3B	AX-110968918	0.2075	54.6217
11	3B	AX-109428712	0.4184	55.0402
11	3B	AX-111513641	0.4184	55.4586
11	3B	AX-109857556	0.4184	55.4586
11	3B	AX-110038027	0.4184	55.4586
11	3B	AX-111562830	0.4184	55.4586
11	3B	AX-110603776	0.4184	55.4586

11	3B	AX-110951388	0.4184	55.4586
11	3B	AX-108848922	0.4184	55.4586
11	3B	AX-111476277	0.4184	55.4586
11	3B	AX-110573587	0.4184	55.4586
11	3B	AX-109436590	0.4184	55.4586
11	3B	AX-110604106	0.4184	55.4586
11	3B	AX-111088814	0.4184	55.4586
11	3B	AX-111286422	0.4184	55.4586
11	3B	AX-110582386	0.2083	55.6669
11	3B	AX-110490439	0.2083	55.6669
11	3B	AX-108884614	0.2083	55.6669
11	3B	AX-111078613	0.2083	55.6669
11	3B	AX-111057940	0.2083	55.6669
11	3B	AX-109836361	0.4184	56.0853
11	3B	AX-111239152	0.4184	56.0853
11	3B	AX-110927096	0.2075	56.2928
11	3B	AX-110385548	0.2075	56.2928
11	3B	AX-110514463	0.2075	56.2928
11	3B	AX-111556675	0.2075	56.2928
11	3B	AX-109407787	0.2075	56.2928
11	3B	AX-109943346	0.2075	56.2928
11	3B	AX-111002034	0.2075	56.2928
11	3B	AX-108842692	0.2075	56.2928
11	3B	AX-111176886	0.2075	56.2928
11	3B	AX-109968489	0.4167	56.7095
11	3B	AX-111036738	0.4167	56.7095
11	3B	AX-110918414	1.0595	57.769
11	3B	AX-110432985	0.6303	58.3993
11	3B	AX-111720444	0.6303	58.3993
11	3B	AX-110561047	0.4167	58.8159
11	3B	AX-108910620	0.4167	58.8159
11	3B	AX-111519405	0.4184	59.2344
11	3B	AX-109950866	0.4184	59.2344
11	3B	AX-109869244	0.2083	59.4427
11	3B	AX-111240831	0.2083	59.4427
11	3B	AX-109508240	0.2083	59.4427
11	3B	AX-109839571	0.2083	59.4427
11	3B	AX-94385658	0.2083	59.4427
11	3B	AX-108848855	0.6329	60.0757
11	3B	AX-108949651	0.6329	60.0757
11	3B	AX-109469895	0.6329	60.0757
11	3B	AX-110560967	0.6329	60.0757
11	3B	AX-110440656	0.6329	60.0757
11	3B	AX-110012386	0.211	60.2866

11	3B	AX-109536560	0.4237	60.7104
11	3B	AX-109363741	0.2101	60.9204
11	3B	AX-111059512	0.2101	60.9204
11	3B	AX-109353206	0.2101	60.9204
11	3B	AX-110492252	0.2101	60.9204
11	3B	AX-94431472	0.2101	60.9204
11	3B	AX-109375754	0.2101	60.9204
11	3B	AX-110671878	0.4184	61.3389
11	3B	AX-109471831	0.2075	61.5463
11	3B	AX-109815102	0.2075	61.5463
11	3B	AX-110968381	0.2075	61.5463
11	3B	AX-108864022	0.2101	61.7564
11	3B	AX-110589570	0.4202	62.1766
11	3B	AX-109952688	0.4202	62.1766
11	3B	AX-109032080	0.4202	62.1766
11	3B	AX-110034575	0.4202	62.1766
11	3B	AX-110984852	0.4202	62.1766
11	3B	AX-109991407	0.4202	62.1766
11	3B	AX-110741718	0.6303	62.8069
11	3B	AX-110669772	0.211	63.0179
11	3B	AX-110931611	0.211	63.0179
11	3B	AX-110448973	0.211	63.0179
11	3B	AX-108952050	0.211	63.0179
11	3B	AX-109649145	0.211	63.0179
11	3B	AX-110110252	0.211	63.0179
11	3B	AX-110551666	0.211	63.0179
11	3B	AX-109598181	0.211	63.0179
11	3B	AX-109103607	0.211	63.0179
11	3B	AX-111045227	0.211	63.0179
11	3B	AX-110996863	0.211	63.0179
11	3B	AX-111083331	0.211	63.0179
11	3B	AX-109035368	0.211	63.0179
11	3B	AX-111112626	2.2041	65.2219
11	3B	AX-94975578	0.2083	65.4303
11	3B	AX-108881120	0.2083	65.4303
11	3B	AX-109329185	0.2083	65.4303
11	3B	AX-111577177	0.2083	65.4303
11	3B	AX-108869068	0.2083	65.4303
11	3B	AX-111490078	0.2083	65.4303
11	3B	AX-108886984	0.2083	65.4303
11	3B	AX-109955431	0.2083	65.4303
11	3B	AX-110928753	0.2083	65.4303
11	3B	AX-110949919	0.2083	65.4303
11	3B	AX-108736477	0.2083	65.4303

11	3B	AX-110441307	0.2083	65.4303
11	3B	AX-111108738	0.2083	65.4303
11	3B	AX-109449661	0.2083	65.4303
11	3B	AX-109461374	0.2083	65.4303
11	3B	AX-110956424	0.2083	65.4303
11	3B	AX-111488531	0.2083	65.4303
11	3B	AX-110142073	0.2083	65.4303
11	3B	AX-109442417	0.2083	65.4303
11	3B	AX-94522514	0.2083	65.4303
11	3B	AX-110555702	0.2083	65.4303
11	3B	AX-110993272	0.2083	65.4303
11	3B	AX-111703504	0.2083	65.4303
11	3B	AX-110386581	0.4202	65.8504
11	3B	AX-109883543	0.4202	65.8504
11	3B	AX-108758726	0.4202	65.8504
11	3B	AX-110065841	0.4202	65.8504
11	3B	AX-110083987	0.4202	65.8504
11	3B	AX-111481902	0.4202	65.8504
11	3B	AX-111491656	0.4202	65.8504
11	3B	AX-111577389	0.4202	65.8504
11	3B	AX-109386512	0.4202	65.8504
11	3B	AX-110503503	0.4202	65.8504
11	3B	AX-110971226	0.4202	65.8504
11	3B	AX-109035003	0.4202	65.8504
11	3B	AX-109033816	0.4202	65.8504
11	3B	AX-110375013	4.329	70.1794
11	3B	AX-111089489	4.329	70.1794
11	3B	AX-109430645	4.329	70.1794
11	3B	AX-110569582	4.329	70.1794
11	3B	AX-109865540	4.329	70.1794
11	3B	AX-109413743	4.329	70.1794
11	3B	AX-95113669	4.329	70.1794
11	3B	AX-110042579	0.6383	70.8177
11	3B	AX-111109401	0.2083	71.0261
11	3B	AX-109436690	0.2083	71.0261
11	3B	AX-109002048	0.2083	71.0261
11	3B	AX-109961989	0.2083	71.0261
11	3B	AX-111452789	0.2083	71.0261
11	3B	AX-108972049	0.2083	71.0261
11	3B	AX-110457687	0.2083	71.0261
11	3B	AX-109442410	0.2083	71.0261
11	3B	AX-110578538	0.2083	71.0261
11	3B	AX-109528767	0.2083	71.0261
11	3B	AX-111505119	0.2083	71.0261

11	3B	AX-109462479	0.2083	71.0261
11	3B	AX-109944478	0.2083	71.0261
11	3B	AX-109517593	0.2083	71.0261
11	3B	AX-111523674	0.2083	71.0261
11	3B	AX-108825863	0.2083	71.0261
11	3B	AX-109993944	0.2083	71.0261
11	3B	AX-111002755	0.2083	71.0261
11	3B	AX-110414599	0.2083	71.0261
11	3B	AX-108959255	0.2083	71.0261
11	3B	AX-111038130	0.2083	71.0261
11	3B	AX-110947218	0.2083	71.0261
11	3B	AX-110926414	0.2083	71.0261
11	3B	AX-109299215	0.2083	71.0261
11	3B	AX-109326955	0.2083	71.0261
11	3B	AX-110991543	0.2083	71.0261
11	3B	AX-110434759	0.2083	71.0261
11	3B	AX-108916567	0.2083	71.0261
11	3B	AX-110567919	0.2083	71.0261
11	3B	AX-108784121	0.2083	71.0261
11	3B	AX-111217974	0.2083	71.0261
11	3B	AX-108898842	0.2083	71.0261
11	3B	AX-110064449	0.2083	71.0261
11	3B	AX-108834969	0.2083	71.0261
11	3B	AX-109865948	0.2083	71.0261
11	3B	AX-110454818	0.2083	71.0261
11	3B	AX-111472497	0.2083	71.0261
11	3B	AX-111119091	0.2083	71.0261
11	3B	AX-108928246	0.2083	71.0261
11	3B	AX-110025120	0.2083	71.0261
11	3B	AX-110372104	0.2083	71.0261
11	3B	AX-111565000	0.2083	71.0261
11	3B	AX-110475726	0.2083	71.0261
11	3B	AX-108949787	0.2083	71.0261
11	3B	AX-111537791	0.2083	71.0261
11	3B	AX-111544749	0.2083	71.0261
11	3B	AX-110000511	0.2083	71.0261
11	3B	AX-111017683	0.2083	71.0261
11	3B	AX-108746674	0.2083	71.0261
11	3B	AX-109355855	0.2083	71.0261
11	3B	AX-109911914	0.2083	71.0261
11	3B	AX-108738488	0.2083	71.0261
11	3B	AX-108929232	0.2083	71.0261
11	3B	AX-111503678	0.2083	71.0261
11	3B	AX-108894913	0.2083	71.0261

11	3B	AX-109373871	0.2083	71.0261
11	3B	AX-109548653	0.2083	71.0261
11	3B	AX-109033473	0.2083	71.0261
11	3B	AX-108907251	0.2083	71.0261
11	3B	AX-110992117	0.2083	71.0261
11	3B	AX-110919590	0.2083	71.0261
11	3B	AX-109594903	0.2083	71.0261
11	3B	AX-109413480	0.2083	71.0261
11	3B	AX-108964920	0.2083	71.0261
11	3B	AX-109576658	0.2083	71.0261
11	3B	AX-109050207	0.2083	71.0261
11	3B	AX-111487965	0.2083	71.0261
11	3B	AX-110029307	0.2083	71.0261
11	3B	AX-110978583	0.2083	71.0261
11	3B	AX-108730983	0.2083	71.0261
11	3B	AX-111250740	0.2083	71.0261
11	3B	AX-110919285	0.2083	71.0261
11	3B	AX-109314845	0.2083	71.0261
11	3B	AX-110429482	0.2083	71.0261
11	3B	AX-109845984	0.2083	71.0261
11	3B	AX-108800704	0.2083	71.0261
11	3B	AX-108731073	0.2083	71.0261
11	3B	AX-108856856	0.2083	71.0261
11	3B	AX-111148547	0.2083	71.0261
11	3B	AX-110654207	0.2083	71.0261
11	3B	AX-109574102	0.2083	71.0261
11	3B	AX-111565090	0.2083	71.0261
11	3B	AX-95659062	0.2083	71.0261
11	3B	AX-108981372	0.2083	71.0261
11	3B	AX-110379930	0.8475	71.8736
11	3B	AX-109368009	0.8475	71.8736
11	3B	AX-109339345	0.8475	71.8736
11	3B	AX-109369935	0.8475	71.8736
11	3B	AX-111498640	0.8475	71.8736
11	3B	AX-109820619	0.8475	71.8736
11	3B	AX-109434274	0.8475	71.8736
11	3B	AX-111473006	0.8475	71.8736
11	3B	AX-109058000	0.8475	71.8736
11	3B	AX-111014208	0.8475	71.8736
11	3B	AX-110589424	0.4202	72.2938
11	3B	AX-108994185	0.4202	72.2938
11	3B	AX-110163403	0.4202	72.2938
11	3B	AX-110669263	0.4202	72.2938
11	3B	AX-108977506	0.4202	72.2938

11	3B	AX-109577590	0.4202	72.2938
11	3B	AX-110391041	0.4202	72.2938
11	3B	AX-109084787	0.4202	72.2938
11	3B	AX-110935708	0.4202	72.2938
11	3B	AX-110388826	0.4202	72.2938
11	3B	AX-110709238	0.4202	72.2938
11	3B	AX-109857363	0.4202	72.2938
11	3B	AX-108825715	0.4202	72.2938
11	3B	AX-109522041	0.4202	72.2938
11	3B	AX-110368902	0.4202	72.2938
11	3B	AX-110416259	0.4202	72.2938
11	3B	AX-109871023	0.4202	72.2938
11	3B	AX-109074938	0.4202	72.2938
11	3B	AX-110020051	0.4202	72.2938
11	3B	AX-108851757	0.4202	72.2938
11	3B	AX-109331447	0.4202	72.2938
11	3B	AX-109622191	0.4202	72.2938
11	3B	AX-108857657	0.4202	72.2938
11	3B	AX-109581980	0.4202	72.2938
11	3B	AX-110023769	0.4202	72.2938
11	3B	AX-111517228	0.4202	72.2938
11	3B	AX-110433023	0.4202	72.2938
11	3B	AX-108864422	0.4202	72.2938
11	3B	AX-110126884	0.4202	72.2938
11	3B	AX-109871156	0.4202	72.2938
11	3B	AX-111547999	0.4202	72.2938
11	3B	AX-110405792	0.4202	72.2938
11	3B	AX-110971908	0.4202	72.2938
11	3B	AX-108892036	0.4202	72.2938
11	3B	AX-108972818	0.4202	72.2938
11	3B	AX-109355690	0.4202	72.2938
11	3B	AX-109890378	0.4202	72.2938
11	3B	AX-108738400	0.4202	72.2938
11	3B	AX-109339000	0.4202	72.2938
11	3B	AX-108818269	0.4202	72.2938
11	3B	AX-110985429	0.4202	72.2938
11	3B	AX-108796704	0.4202	72.2938
11	3B	AX-111127517	0.4202	72.2938
11	3B	AX-111527521	0.4202	72.2938
11	3B	AX-111082594	0.4202	72.2938
11	3B	AX-108793027	0.4202	72.2938
11	3B	AX-108790818	0.4202	72.2938
11	3B	AX-109870020	0.4202	72.2938
11	3B	AX-110909691	0.4202	72.2938

11	3B	AX-110390360	0.4202	72.2938
11	3B	AX-109962603	0.4202	72.2938
11	3B	AX-109344648	0.4202	72.2938
11	3B	AX-110645854	0.4202	72.2938
11	3B	AX-110633697	0.4202	72.2938
11	3B	AX-109336712	0.4202	72.2938
11	3B	AX-108792250	0.4202	72.2938
11	3B	AX-110477681	0.4202	72.2938
11	3B	AX-109294180	0.4202	72.2938
11	3B	AX-110020080	0.4202	72.2938
11	3B	AX-109354550	0.4202	72.2938
11	3B	AX-108795061	0.4202	72.2938
11	3B	AX-109452154	0.4202	72.2938
11	3B	AX-110129604	0.4202	72.2938
11	3B	AX-109342725	0.4202	72.2938
11	3B	AX-108822347	0.4202	72.2938
11	3B	AX-110369837	0.4202	72.2938
11	3B	AX-111105945	0.4202	72.2938
11	3B	AX-108727907	0.4202	72.2938
11	3B	AX-109352341	0.4202	72.2938
11	3B	AX-111562102	0.4202	72.2938
11	3B	AX-110039601	0.4202	72.2938
11	3B	AX-110081412	0.4202	72.2938
11	3B	AX-108837731	0.4202	72.2938
11	3B	AX-109291758	0.4167	72.7105
11	3B	AX-109035036	0.4167	72.7105
11	3B	AX-109465849	0.4167	72.7105
11	3B	AX-110507612	0.4167	72.7105
11	3B	AX-110441007	0.4167	72.7105
11	3B	AX-108842324	0.4167	72.7105
11	3B	AX-108913127	0.4167	72.7105
11	3B	AX-110506270	0.4167	72.7105
11	3B	AX-110991528	0.4167	72.7105
11	3B	AX-110023083	0.4167	72.7105
11	3B	AX-111522150	0.4167	72.7105
11	3B	AX-108843975	0.4167	72.7105
11	3B	AX-108756958	0.4167	72.7105
11	3B	AX-109300864	0.4167	72.7105
11	3B	AX-110469464	0.4167	72.7105
11	3B	AX-110380092	0.4167	72.7105
11	3B	AX-108903394	0.4167	72.7105
11	3B	AX-111495472	0.4167	72.7105
11	3B	AX-110497472	0.4167	72.7105
11	3B	AX-108763662	0.4167	72.7105

11	3B	AX-111130632	0.4167	72.7105
11	3B	AX-109350957	0.4167	72.7105
11	3B	AX-109994038	0.4167	72.7105
11	3B	AX-110053102	0.4167	72.7105
11	3B	AX-109429203	0.4167	72.7105
11	3B	AX-108943578	0.4167	72.7105
11	3B	AX-110538297	0.4167	72.7105
11	3B	AX-110604092	0.4167	72.7105
11	3B	AX-110464232	0.4167	72.7105
11	3B	AX-111060191	0.4167	72.7105
11	3B	AX-110503365	0.4167	72.7105
11	3B	AX-110376058	0.4167	72.7105
11	3B	AX-109836635	0.4167	72.7105
11	3B	AX-111495595	0.4167	72.7105
11	3B	AX-109889436	0.4167	72.7105
11	3B	AX-108773796	0.4167	72.7105
11	3B	AX-109600737	0.4167	72.7105
11	3B	AX-110963711	0.4167	72.7105
11	3B	AX-110122344	0.4167	72.7105
11	3B	AX-111527380	0.4167	72.7105
11	3B	AX-110024763	0.4167	72.7105
11	3B	AX-111284346	0.4167	72.7105
11	3B	AX-111686153	0.4167	72.7105
11	3B	AX-94933156	0.4167	72.7105
11	3B	AX-95216883	0.4167	72.7105
11	3B	AX-111563863	0.4184	73.1289
11	3B	AX-109577069	0.2083	73.3372
11	3B	AX-111200675	0.2083	73.3372
11	3B	AX-110924649	0.2083	73.3372
11	3B	AX-110481088	0.2083	73.3372
11	3B	AX-111147897	0.2083	73.5456
11	3B	AX-111151634	0.2083	73.5456
11	3B	AX-111133121	0.2083	73.5456
11	3B	AX-109834254	0.2083	73.5456
11	3B	AX-111191528	0.625	74.1706
11	3B	AX-109830120	1.2661	75.4367
11	3B	AX-110420045	1.2661	75.4367
11	3B	AX-108815081	1.2661	75.4367
11	3B	AX-109520698	1.2661	75.4367
11	3B	AX-109461877	1.2661	75.4367
11	3B	AX-110127873	1.2661	75.4367
11	3B	AX-109374827	1.2661	75.4367
11	3B	AX-110030743	1.4898	76.9265
11	3B	AX-95012984	1.4898	76.9265

11	3B	AX-109979646	0.2083	77.1348
11	3B	AX-109329844	0.2101	77.3449
11	3B	AX-111082190	0.2101	77.3449
11	3B	AX-111580329	0.2092	77.5541
11	3B	AX-109483112	0.2092	77.5541
11	3B	AX-111628771	0.2092	77.5541
11	3B	AX-110918765	0.4237	77.9779
11	3B	AX-111007091	0.211	78.1888
11	3B	AX-109365374	0.211	78.1888
11	3B	AX-109466416	0.211	78.1888
11	3B	AX-111005502	0.211	78.1888
11	3B	AX-109966505	0.211	78.1888
11	3B	AX-109993095	0.2083	78.3972
11	3B	AX-110476879	0.2083	78.3972
11	3B	AX-108725963	0.2083	78.3972
11	3B	AX-111562340	0.2083	78.3972
11	3B	AX-111033374	0.2083	78.3972
11	3B	AX-110126449	0.2092	78.6064
11	3B	AX-111503846	0.2092	78.6064
11	3B	AX-109496631	0.2092	78.6064
11	3B	AX-110467360	0.2092	78.6064
11	3B	AX-108806083	0.2092	78.6064
11	3B	AX-110999535	0.422	79.0283
11	3B	AX-109951572	0.6329	79.6613
11	3B	AX-109529345	0.6329	79.6613
11	3B	AX-110063738	0.6329	79.6613
11	3B	AX-110464771	0.6329	79.6613
11	3B	AX-110552499	0.6329	79.6613
11	3B	AX-108805727	0.6329	79.6613
11	3B	AX-109923656	0.6329	79.6613
11	3B	AX-109339690	0.6329	79.6613
11	3B	AX-109581614	0.6329	79.6613
11	3B	AX-111030999	0.6329	79.6613
11	3B	AX-108760591	0.6329	79.6613
11	3B	AX-110670863	0.6329	79.6613
11	3B	AX-109108992	0.2128	79.874
11	3B	AX-108859880	7.1563	87.0303
11	3B	AX-109382680	2.854	89.8843
11	3B	AX-108829868	2.854	89.8843
11	3B	AX-109362175	2.854	89.8843
11	3B	AX-111096238	2.854	89.8843
11	3B	AX-111515231	0.6303	90.5146
11	3B	AX-109953953	0.2075	90.7221
11	3B	AX-109997810	2.4356	93.1576

11	3B	AX-109310077	2.4356	93.1576
11	3B	AX-111589926	0.2101	93.3677
11	3B	AX-109277293	0.2101	93.3677
11	3B	AX-109396253	0.422	93.7897
11	3B	AX-109457476	0.422	93.7897
11	3B	AX-110566919	0.422	93.7897
11	3B	AX-109910869	0.8548	94.6445
11	3B	AX-109615874	0.8548	94.6445
11	3B	AX-110568568	0.8548	94.6445
11	3B	AX-110482650	0.8548	94.6445
11	3B	AX-110629601	0.8548	94.6445
11	3B	AX-109917444	2.1944	96.8388
11	3B	AX-110917789	2.1944	96.8388
11	3B	AX-108951629	6.7391	103.5779
11	3B	AX-110125816	6.7391	103.5779
11	3B	AX-111107270	0.4184	103.9963
11	3B	AX-110438189	2.4248	106.4211
11	3B	AX-109949824	6.1631	112.5842
11	3B	AX-109454137	6.1631	112.5842
11	3B	AX-108759829	6.1631	112.5842
11	3B	AX-108837323	6.1631	112.5842
11	3B	AX-110146078	6.1631	112.5842
11	3B	AX-109301820	0.2083	112.7926
11	3B	AX-111565289	0.2083	112.7926
11	3B	AX-109338678	0.4184	113.211
11	3B	AX-109932454	0.4184	113.211
11	3B	AX-108783900	0.4184	113.211
11	3B	AX-109441777	0.2075	113.4185
11	3B	AX-108744295	0.4202	113.8386
11	3B	AX-109328969	0.4202	113.8386
11	3B	AX-110399835	0.4202	113.8386
11	3B	AX-111502226	26.6284	140.4671
11	3B	AX-109279813	18.871	159.3381
11	3B	AX-111464020	18.871	159.3381
11	3B	AX-109312574	18.871	159.3381
11	3B	AX-110370133	18.871	159.3381
11	3B	AX-110428478	0.4202	159.7583
12	3D	AX-95632197	0	0
12	3D	AX-109299632	7.7137	7.7137
12	3D	AX-94682394	0.8548	8.5685
12	3D	AX-109820293	2.2138	10.7823
12	3D	AX-109440542	11.0576	21.8399
12	3D	AX-111503477	11.0576	21.8399
12	3D	AX-109894867	11.0576	21.8399

12	3D	AX-111579842	3.3835	25.2235
12	3D	AX-109282391	3.3835	25.2235
12	3D	AX-111450196	3.3835	25.2235
12	3D	AX-110944177	12.2166	37.4401
12	3D	AX-110611062	12.2166	37.4401
12	3D	AX-109412819	12.2166	37.4401
12	3D	AX-111112489	12.2166	37.4401
12	3D	AX-110165975	12.2166	37.4401
12	3D	AX-109999648	12.2166	37.4401
12	3D	AX-109036557	12.2166	37.4401
12	3D	AX-109393346	12.2166	37.4401
12	3D	AX-109399079	12.2166	37.4401
12	3D	AX-110010771	12.2166	37.4401
12	3D	AX-110930966	12.2166	37.4401
12	3D	AX-112291131	12.2166	37.4401
12	3D	AX-109915790	1.9406	39.3807
12	3D	AX-109281651	1.9406	39.3807
12	3D	AX-94619709	1.9406	39.3807
12	3D	AX-95111256	18.871	58.2517
12	3D	AX-110588202	18.871	58.2517
12	3D	AX-110363430	17.7635	76.0152
13	3D	AX-108870700	0	0
13	3D	AX-109334027	0	0
13	3D	AX-110484906	0	0
13	3D	AX-108971176	0	0
13	3D	AX-111607702	13.937	13.937
13	3D	AX-108907834	8.2839	22.2209
13	3D	AX-89592287	1.5356	23.7565
13	3D	AX-111337684	15.4449	39.2015
13	3D	AX-110230951	0.4292	39.6307
13	3D	AX-108762281	1.0778	40.7084
13	3D	AX-111080889	2.1944	42.9028
13	3D	AX-109101506	2.1944	42.9028
13	3D	AX-108800061	1.2934	44.1962
13	3D	AX-109422875	1.3161	45.5123
13	3D	AX-110234451	1.1015	46.6138
13	3D	AX-111291115	0.6579	47.2717
13	3D	AX-110376389	0.2164	47.4882
13	3D	AX-109499958	1.3046	48.7928
13	3D	AX-110477646	2.2041	50.9969
13	3D	AX-111617161	0.6466	51.6435
13	3D	AX-111556201	0.6466	51.6435
13	3D	AX-108815110	0.6466	51.6435
13	3D	AX-109504334	0.6466	51.6435

13	3D	AX-110372611	0.4274	52.0709
13	3D	AX-110538357	0.4274	52.0709
13	3D	AX-109909862	0.4274	52.0709
13	3D	AX-109660230	0.431	52.5019
13	3D	AX-108791525	0.2146	52.7165
13	3D	AX-110365829	0.2146	52.7165
13	3D	AX-109537884	0.2137	52.9302
13	3D	AX-111155780	0.2137	52.9302
13	3D	AX-110594376	0.2137	52.9302
13	3D	AX-111522143	0.2137	52.9302
13	3D	AX-111371048	0.2137	52.9302
13	3D	AX-109317471	0.2137	52.9302
13	3D	AX-109143936	0.2137	52.9302
13	3D	AX-110214968	0.2137	52.9302
13	3D	AX-111666894	0.2137	52.9302
13	3D	AX-110336377	0.2146	53.1448
13	3D	AX-111512463	0.2146	53.1448
13	3D	AX-109332060	0.2146	53.1448
13	3D	AX-110450605	0.2146	53.1448
13	3D	AX-108938799	0.2146	53.1448
13	3D	AX-108859112	0.2146	53.1448
13	3D	AX-110857044	0.2146	53.1448
13	3D	AX-109284474	0.2146	53.1448
13	3D	AX-111708993	0.431	53.5758
13	3D	AX-109513823	0.431	53.5758
13	3D	AX-110529732	0.431	53.5758
13	3D	AX-111591851	0.431	53.5758
13	3D	AX-111528152	0.431	53.5758
13	3D	AX-110372784	0.431	53.5758
13	3D	AX-111776296	0.431	53.5758
13	3D	AX-108857574	0.431	53.5758
13	3D	AX-111585374	0.431	53.5758
13	3D	AX-109874802	0.431	53.5758
13	3D	AX-110363017	0.431	53.5758
13	3D	AX-111916230	0.431	53.5758
13	3D	AX-109383254	0.431	53.5758
13	3D	AX-111538290	0.431	53.5758
13	3D	AX-108756011	0.431	53.5758
13	3D	AX-108874896	0.431	53.5758
13	3D	AX-108898785	0.431	53.5758
13	3D	AX-108856163	0.431	53.5758
13	3D	AX-109116080	0.431	53.5758
13	3D	AX-109723086	0.431	53.5758
13	3D	AX-109380273	0.431	53.5758

13	3D	AX-110877291	0.431	53.5758
13	3D	AX-109120346	0.431	53.5758
13	3D	AX-109743167	0.431	53.5758
13	3D	AX-111362517	0.431	53.5758
13	3D	AX-111030957	0.431	53.5758
13	3D	AX-111054517	0.431	53.5758
13	3D	AX-108895977	0.431	53.5758
13	3D	AX-111675563	0.431	53.5758
13	3D	AX-110008196	0.431	53.5758
13	3D	AX-108895854	0.431	53.5758
13	3D	AX-111762331	0.431	53.5758
13	3D	AX-108877579	0.431	53.5758
13	3D	AX-110004416	0.431	53.5758
13	3D	AX-109732364	0.431	53.5758
13	3D	AX-108778625	0.431	53.5758
13	3D	AX-109745596	0.431	53.5758
13	3D	AX-110769313	0.431	53.5758
13	3D	AX-111379661	0.431	53.5758
13	3D	AX-111131164	0.431	53.5758
13	3D	AX-110929772	0.431	53.5758
13	3D	AX-111510604	0.431	53.5758
13	3D	AX-111506806	0.431	53.5758
13	3D	AX-110260977	0.431	53.5758
13	3D	AX-108899105	0.431	53.5758
13	3D	AX-109976094	0.431	53.5758
13	3D	AX-109074021	0.431	53.5758
13	3D	AX-108854836	0.431	53.5758
13	3D	AX-109517575	0.431	53.5758
13	3D	AX-111648558	0.431	53.5758
13	3D	AX-109038709	0.431	53.5758
13	3D	AX-108830038	0.431	53.5758
13	3D	AX-110354859	0.431	53.5758
13	3D	AX-111504450	0.431	53.5758
13	3D	AX-110827822	0.431	53.5758
13	3D	AX-109477772	0.431	53.5758
13	3D	AX-111779817	0.431	53.5758
13	3D	AX-109191215	0.431	53.5758
13	3D	AX-110269943	0.431	53.5758
13	3D	AX-110520367	0.431	53.5758
13	3D	AX-111460869	0.431	53.5758
13	3D	AX-108841875	0.431	53.5758
13	3D	AX-109936706	0.431	53.5758
13	3D	AX-111113250	0.431	53.5758
13	3D	AX-109889579	0.431	53.5758

13	3D	AX-110673653	0.431	53.5758
13	3D	AX-110521614	0.431	53.5758
13	3D	AX-109990662	0.431	53.5758
13	3D	AX-109786468	0.431	53.5758
13	3D	AX-109394931	0.431	53.5758
13	3D	AX-109724938	0.431	53.5758
13	3D	AX-110015572	0.431	53.5758
13	3D	AX-109163107	0.431	53.5758
13	3D	AX-110542392	0.431	53.5758
13	3D	AX-109273377	0.431	53.5758
13	3D	AX-110278918	0.431	53.5758
13	3D	AX-110926251	0.431	53.5758
13	3D	AX-110674002	0.431	53.5758
13	3D	AX-111331372	0.431	53.5758
13	3D	AX-109037401	0.431	53.5758
13	3D	AX-110065383	0.431	53.5758
13	3D	AX-110516071	0.431	53.5758
13	3D	AX-110321035	0.431	53.5758
13	3D	AX-110823805	0.431	53.5758
13	3D	AX-111532875	0.431	53.5758
13	3D	AX-110970464	0.431	53.5758
13	3D	AX-110596103	0.431	53.5758
13	3D	AX-109923833	0.431	53.5758
13	3D	AX-110533567	0.431	53.5758
13	3D	AX-110004254	0.431	53.5758
13	3D	AX-108830026	0.431	53.5758
13	3D	AX-110441918	0.431	53.5758
13	3D	AX-110127556	0.431	53.5758
13	3D	AX-109067542	0.431	53.5758
13	3D	AX-111640045	0.431	53.5758
13	3D	AX-109399234	0.431	53.5758
13	3D	AX-109710003	0.431	53.5758
13	3D	AX-110913237	0.431	53.5758
13	3D	AX-109779736	0.431	53.5758
13	3D	AX-110407934	0.431	53.5758
13	3D	AX-109374312	0.431	53.5758
13	3D	AX-109206172	0.431	53.5758
13	3D	AX-111689571	0.431	53.5758
13	3D	AX-110918749	0.431	53.5758
13	3D	AX-108989119	0.431	53.5758
13	3D	AX-111010706	0.431	53.5758
13	3D	AX-109841503	0.431	53.5758
13	3D	AX-110959583	0.431	53.5758
13	3D	AX-110581591	0.431	53.5758

13	3D	AX-108903689	0.431	53.5758
13	3D	AX-109505110	0.431	53.5758
13	3D	AX-108899974	0.431	53.5758
13	3D	AX-110237186	0.431	53.5758
13	3D	AX-111571032	0.431	53.5758
13	3D	AX-110915537	0.431	53.5758
13	3D	AX-110289490	0.431	53.5758
13	3D	AX-109550406	0.431	53.5758
13	3D	AX-110771985	0.431	53.5758
13	3D	AX-111504843	0.431	53.5758
13	3D	AX-109847390	0.431	53.5758
13	3D	AX-109783478	0.431	53.5758
13	3D	AX-108901772	0.431	53.5758
13	3D	AX-109348356	0.431	53.5758
13	3D	AX-109852628	0.431	53.5758
13	3D	AX-109735361	0.431	53.5758
13	3D	AX-109286470	0.431	53.5758
13	3D	AX-111216480	0.431	53.5758
13	3D	AX-110381932	0.431	53.5758
13	3D	AX-111502769	0.431	53.5758
13	3D	AX-111075785	0.431	53.5758
13	3D	AX-108765980	0.431	53.5758
13	3D	AX-109954183	0.431	53.5758
13	3D	AX-111507826	0.431	53.5758
13	3D	AX-110449684	0.431	53.5758
13	3D	AX-111131844	0.431	53.5758
13	3D	AX-109383325	0.431	53.5758
13	3D	AX-110511459	0.431	53.5758
13	3D	AX-108796887	0.431	53.5758
13	3D	AX-109379099	0.431	53.5758
13	3D	AX-110958097	0.431	53.5758
13	3D	AX-111096826	0.431	53.5758
13	3D	AX-110288100	0.431	53.5758
13	3D	AX-110029250	0.431	53.5758
13	3D	AX-108926470	0.431	53.5758
13	3D	AX-108958991	0.431	53.5758
13	3D	AX-109889311	0.431	53.5758
13	3D	AX-109744083	0.431	53.5758
13	3D	AX-110373248	0.431	53.5758
13	3D	AX-110894943	0.431	53.5758
13	3D	AX-109052291	0.431	53.5758
13	3D	AX-109292473	0.431	53.5758
13	3D	AX-109928549	0.431	53.5758
13	3D	AX-111322575	0.431	53.5758

13	3D	AX-111719709	0.431	53.5758
13	3D	AX-111840601	0.431	53.5758
13	3D	AX-110941541	0.4292	54.005
13	3D	AX-109538535	0.4292	54.005
13	3D	AX-111921159	0.4292	54.005
13	3D	AX-110960738	0.4292	54.005
13	3D	AX-109319340	0.4292	54.005
13	3D	AX-110986033	0.2128	54.2178
13	3D	AX-111543321	0.2128	54.2178
13	3D	AX-111344580	0.2128	54.2178
13	3D	AX-110827948	0.2137	54.4315
13	3D	AX-109693734	0.2137	54.4315
13	3D	AX-109135722	0.2137	54.4315
13	3D	AX-109843198	0.2137	54.4315
13	3D	AX-111091672	0.2137	54.4315
13	3D	AX-109278916	0.2137	54.4315
13	3D	AX-110752656	0.2137	54.4315
13	3D	AX-110857561	0.2137	54.4315
13	3D	AX-109306840	2.2237	56.6552
13	3D	AX-110398405	2.2237	56.6552
13	3D	AX-109830194	2.2237	56.6552
13	3D	AX-109341147	2.2237	56.6552
13	3D	AX-109131732	2.2237	56.6552
13	3D	AX-111601540	0.6329	57.2881
13	3D	AX-110387704	0.6329	57.2881
13	3D	AX-110586143	0.6329	57.2881
13	3D	AX-110489977	0.2101	57.4982
13	3D	AX-111159927	0.2101	57.4982
13	3D	AX-108795505	0.4274	57.9256
13	3D	AX-109431678	0.6438	58.5694
13	3D	AX-111027124	1.0685	59.6379
13	3D	AX-108738283	16.7979	76.4358
13	3D	AX-109826344	2.01	78.4458
13	3D	AX-89337262	0.2164	78.6622
13	3D	AX-89424138	1.0778	79.74
13	3D	AX-110282866	0.2128	79.9528
13	3D	AX-109884133	2.4684	82.4211
13	3D	AX-109957718	2.4684	82.4211
13	3D	AX-111045512	1.1015	83.5226
13	3D	AX-111600632	1.1015	83.5226
13	3D	AX-109303898	0.8697	84.3923
13	3D	AX-109319838	4.1959	88.5882
13	3D	AX-111484465	0.6411	89.2292
13	3D	AX-109980905	0.6411	89.2292

13	3D	AX-109688668	0.2146	89.4438
13	3D	AX-109799345	0.6494	90.0932
13	3D	AX-110495749	1.7551	91.8483
13	3D	AX-111807575	1.3046	93.1529
13	3D	AX-108842519	0.6466	93.7995
13	3D	AX-89725997	1.5423	95.3419
13	3D	AX-109429351	5.2074	100.5493
13	3D	AX-109417352	1.5492	102.0985
13	3D	AX-110515593	21.0392	123.1376
13	3D	AX-110543919	21.0392	123.1376
13	3D	AX-110042483	2.6692	125.8068
13	3D	AX-111648201	16.7887	142.5955
13	3D	AX-111617121	11.1773	153.7728
13	3D	AX-109502304	14.5258	168.2986
13	3D	AX-111760165	0.8511	169.1497
13	3D	AX-109329567	8.621	177.7707
13	3D	AX-109971254	8.621	177.7707
13	3D	AX-111048151	8.621	177.7707
13	3D	AX-110600123	8.621	177.7707
13	3D	AX-110397184	0.844	178.6147
13	3D	AX-109041644	0.844	178.6147
13	3D	AX-109988926	0.844	178.6147
13	3D	AX-111802381	0.844	178.6147
13	3D	AX-111572552	0.844	178.6147
13	3D	AX-111088384	0.2083	178.823
13	3D	AX-111561378	0.2083	178.823
13	3D	AX-110613747	0.2083	178.823
13	3D	AX-108773502	0.2083	178.823
13	3D	AX-110742388	0.2083	178.823
13	3D	AX-109304130	0.2083	178.823
13	3D	AX-111620986	0.2083	178.823
13	3D	AX-109369770	0.2083	178.823
13	3D	AX-95106971	0.2083	178.823
13	3D	AX-111897546	0.2083	178.823
13	3D	AX-110811858	0.2083	178.823
13	3D	AX-109405015	0.2083	178.823
13	3D	AX-110944677	0.2083	178.823
13	3D	AX-94480620	1.2878	180.1109
13	3D	AX-110037813	0.2092	180.3201
13	3D	AX-109578496	1.5026	181.8227
13	3D	AX-111484055	1.2823	183.105
13	3D	AX-109459242	0.6356	183.7406
13	3D	AX-109990554	2.4142	186.1548
13	3D	AX-109271722	1.0595	187.2142

13	3D	AX-109457357	1.4962	188.7104
13	3D	AX-111490104	0.6224	189.3329
13	3D	AX-111115397	0.6224	189.3329
13	3D	AX-109836636	0.6224	189.3329
13	3D	AX-109403595	0.2075	189.5403
13	3D	AX-110374472	0.6329	190.1732
13	3D	AX-108976456	0.6329	190.1732
13	3D	AX-111675440	0.6329	190.1732
13	3D	AX-109277375	0.6329	190.1732
13	3D	AX-109294647	0.6329	190.1732
13	3D	AX-95164518	0.6329	190.1732
13	3D	AX-109941813	0.6329	190.1732
13	3D	AX-110941549	0.6329	190.1732
13	3D	AX-111575053	0.6329	190.1732
13	3D	AX-110454890	0.6329	190.1732
13	3D	AX-110254140	0.6329	190.1732
13	3D	AX-110043708	0.6329	190.1732
13	3D	AX-111724524	0.6329	190.1732
13	3D	AX-110567532	0.6329	190.1732
13	3D	AX-109615930	0.6329	190.1732
13	3D	AX-108904616	0.6329	190.1732
13	3D	AX-111017532	0.6329	190.1732
13	3D	AX-89337986	0.6329	190.1732
13	3D	AX-109739493	0.6329	190.1732
13	3D	AX-110202442	0.6329	190.1732
13	3D	AX-111058272	0.6329	190.1732
13	3D	AX-108967944	0.6329	190.1732
13	3D	AX-111322167	0.6329	190.1732
13	3D	AX-111699435	0.6329	190.1732
13	3D	AX-108783562	0.6329	190.1732
13	3D	AX-110580049	0.6329	190.1732
13	3D	AX-111959893	0.6329	190.1732
13	3D	AX-108851401	0.6329	190.1732
13	3D	AX-94541936	0.6329	190.1732
13	3D	AX-110928557	0.6329	190.1732
13	3D	AX-94894879	0.6329	190.1732
13	3D	AX-108765097	0.6329	190.1732
13	3D	AX-110092249	0.6329	190.1732
13	3D	AX-110889598	0.6329	190.1732
13	3D	AX-111649610	0.6329	190.1732
13	3D	AX-109272357	0.6329	190.1732
13	3D	AX-109430377	0.6329	190.1732
13	3D	AX-108838201	0.6329	190.1732
13	3D	AX-110035004	0.2083	190.3816

13	3D	AX-109584587	0.6303	191.0119
13	3D	AX-111070267	0.6303	191.0119
13	3D	AX-111483682	0.6303	191.0119
13	3D	AX-108747586	0.6303	191.0119
13	3D	AX-108834675	0.6303	191.0119
13	3D	AX-110960716	0.6303	191.0119
13	3D	AX-109744476	0.6303	191.0119
13	3D	AX-111700213	0.6303	191.0119
13	3D	AX-110570184	0.6303	191.0119
13	3D	AX-110548475	0.6303	191.0119
13	3D	AX-111764702	0.6303	191.0119
13	3D	AX-108965210	0.6303	191.0119
13	3D	AX-111664962	0.6303	191.0119
13	3D	AX-108956520	0.6303	191.0119
13	3D	AX-111825805	0.6303	191.0119
13	3D	AX-108796256	0.6303	191.0119
13	3D	AX-109972844	0.6303	191.0119
13	3D	AX-108947253	0.6303	191.0119
13	3D	AX-109149217	0.6303	191.0119
13	3D	AX-109244474	0.6303	191.0119
13	3D	AX-109723480	0.6303	191.0119
13	3D	AX-109927547	0.6303	191.0119
13	3D	AX-108765756	0.6303	191.0119
13	3D	AX-109369028	0.6303	191.0119
13	3D	AX-111906245	0.6303	191.0119
13	3D	AX-111044528	0.6303	191.0119
13	3D	AX-108828486	0.6303	191.0119
13	3D	AX-109879177	0.6303	191.0119
13	3D	AX-110667777	0.6303	191.0119
13	3D	AX-109982139	0.6303	191.0119
13	3D	AX-110471609	0.2075	191.2193
13	3D	AX-110062575	0.2075	191.2193
13	3D	AX-95164655	0.2075	191.2193
13	3D	AX-110458739	0.2075	191.2193
13	3D	AX-109306356	0.2075	191.2193
13	3D	AX-109883264	0.2075	191.4268
13	3D	AX-110492007	0.2075	191.4268
13	3D	AX-89400808	0.2075	191.4268
13	3D	AX-108823065	0.2075	191.4268
13	3D	AX-109465437	0.2075	191.4268
13	3D	AX-110487595	0.2075	191.4268
13	3D	AX-111061802	0.2075	191.4268
13	3D	AX-110996112	0.2075	191.4268
13	3D	AX-110989777	0.2075	191.4268

13	3D	AX-94486074	0.2075	191.4268
13	3D	AX-110443023	0.2075	191.4268
13	3D	AX-109283120	0.2075	191.4268
13	3D	AX-110398066	0.2075	191.4268
13	3D	AX-111151787	0.2075	191.4268
13	3D	AX-110997964	0.2075	191.4268
13	3D	AX-110605387	0.2075	191.4268
13	3D	AX-111757734	0.2075	191.4268
13	3D	AX-109836095	0.2075	191.4268
13	3D	AX-110937136	0.2075	191.4268
13	3D	AX-94427303	0.2075	191.4268
13	3D	AX-111125474	0.2075	191.4268
13	3D	AX-108821825	0.2075	191.4268
13	3D	AX-109277576	0.2075	191.4268
13	3D	AX-110932084	0.2066	191.6334
13	3D	AX-111510480	0.2066	191.6334
13	3D	AX-111477086	0.2066	191.6334
13	3D	AX-110928305	0.2066	191.6334
13	3D	AX-110387822	0.2066	191.6334
13	3D	AX-110588659	0.2066	191.6334
13	3D	AX-109907925	0.2066	191.6334
13	3D	AX-111552250	0.2066	191.6334
13	3D	AX-111533206	0.2066	191.6334
13	3D	AX-110695238	0.2066	191.6334
13	3D	AX-111555293	0.2066	191.6334
13	3D	AX-108760016	0.2066	191.6334
13	3D	AX-110824697	0.2066	191.6334
13	3D	AX-109860677	0.2066	191.6334
13	3D	AX-111502521	0.2066	191.6334
13	3D	AX-110323427	0.2066	191.6334
13	3D	AX-109781651	0.2066	191.6334
13	3D	AX-109741209	0.2066	191.6334
13	3D	AX-111920409	0.2066	191.6334
13	3D	AX-110010735	0.2066	191.6334
13	3D	AX-111672631	0.2066	191.6334
13	3D	AX-110474974	0.2066	191.6334
13	3D	AX-111160499	0.2066	191.6334
13	3D	AX-109654071	0.2066	191.6334
13	3D	AX-110449739	0.2066	191.6334
13	3D	AX-110320468	0.2066	191.6334
13	3D	AX-111162584	0.2066	191.6334
13	3D	AX-109334718	0.2066	191.6334
13	3D	AX-109892637	0.2066	191.6334
13	3D	AX-111538354	0.2066	191.6334

13	3D	AX-109378487	0.2066	191.6334
13	3D	AX-111623698	0.2066	191.6334
13	3D	AX-111481627	1.055	192.6884
13	3D	AX-111068370	0.4202	193.1086
13	3D	AX-110788038	0.2092	193.3178
13	3D	AX-89662133	1.064	194.3818
13	3D	AX-111180313	0.6303	195.0121
13	3D	AX-109462253	0.6303	195.0121
13	3D	AX-110963637	0.6303	195.0121
13	3D	AX-111696889	0.6303	195.0121
13	3D	AX-110380716	0.6303	195.0121
13	3D	AX-108729438	0.6303	195.0121
13	3D	AX-95138711	0.625	195.6371
13	3D	AX-108843751	0.625	195.6371
13	3D	AX-109358582	0.625	195.6371
13	3D	AX-109207920	0.625	195.6371
13	3D	AX-109364302	0.625	195.6371
13	3D	AX-111930751	0.625	195.6371
13	3D	AX-109356419	0.625	195.6371
13	3D	AX-110439533	0.625	195.6371
13	3D	AX-111258512	0.625	195.6371
13	3D	AX-110697862	0.625	195.6371
13	3D	AX-108863807	0.625	195.6371
13	3D	AX-110390397	0.625	195.6371
13	3D	AX-110919265	0.625	195.6371
13	3D	AX-109347975	0.625	195.6371
13	3D	AX-110377452	0.625	195.6371
13	3D	AX-109925435	0.625	195.6371
13	3D	AX-110484477	0.625	195.6371
13	3D	AX-110953293	0.625	195.6371
13	3D	AX-108857193	0.625	195.6371
13	3D	AX-111788551	0.625	195.6371
13	3D	AX-111058233	0.625	195.6371
13	3D	AX-109816205	0.625	195.6371
13	3D	AX-109406220	0.625	195.6371
13	3D	AX-110946077	0.625	195.6371
13	3D	AX-109860041	0.625	195.6371
13	3D	AX-109336587	0.625	195.6371
13	3D	AX-94383777	0.625	195.6371
13	3D	AX-110441399	0.625	195.6371
13	3D	AX-108787732	0.625	195.6371
13	3D	AX-109394590	0.625	195.6371
13	3D	AX-108729443	0.625	195.6371
13	3D	AX-108736363	0.625	195.6371

13	3D	AX-94712923	0.625	195.6371
13	3D	AX-111408918	0.625	195.6371
13	3D	AX-111905493	0.625	195.6371
13	3D	AX-108878108	0.625	195.6371
13	3D	AX-111601432	0.625	195.6371
13	3D	AX-109674640	0.625	195.6371
13	3D	AX-110417092	0.625	195.6371
13	3D	AX-110542579	0.625	195.6371
13	3D	AX-110404366	0.625	195.6371
13	3D	AX-111552226	0.625	195.6371
13	3D	AX-109446151	0.625	195.6371
13	3D	AX-111544988	0.625	195.6371
13	3D	AX-110925820	0.625	195.6371
13	3D	AX-109957103	0.625	195.6371
13	3D	AX-109844706	0.625	195.6371
13	3D	AX-110199689	0.625	195.6371
13	3D	AX-108804689	0.625	195.6371
13	3D	AX-108952747	0.625	195.6371
13	3D	AX-109918674	0.625	195.6371
13	3D	AX-108776470	0.625	195.6371
13	3D	AX-111010219	0.625	195.6371
13	3D	AX-110492898	0.625	195.6371
13	3D	AX-109378379	0.625	195.6371
13	3D	AX-109517353	0.625	195.6371
13	3D	AX-109604328	0.625	195.6371
13	3D	AX-109845836	0.625	195.6371
13	3D	AX-109892255	0.625	195.6371
13	3D	AX-109933048	0.625	195.6371
13	3D	AX-110473263	0.625	195.6371
13	3D	AX-110365670	0.625	195.6371
13	3D	AX-111376561	0.625	195.6371
13	3D	AX-110595788	0.625	195.6371
13	3D	AX-110291421	0.625	195.6371
13	3D	AX-108883535	0.625	195.6371
13	3D	AX-108789892	0.625	195.6371
13	3D	AX-111462032	0.625	195.6371
13	3D	AX-109469981	0.625	195.6371
13	3D	AX-111076394	0.625	195.6371
13	3D	AX-109873179	0.625	195.6371
13	3D	AX-109852251	0.625	195.6371
13	3D	AX-108830927	0.625	195.6371
13	3D	AX-110946337	0.625	195.6371
13	3D	AX-109357535	0.625	195.6371
13	3D	AX-111588234	0.625	195.6371

13	3D	AX-109037296	0.625	195.6371
13	3D	AX-108801722	0.625	195.6371
13	3D	AX-111501091	0.625	195.6371
13	3D	AX-109986750	0.625	195.6371
13	3D	AX-110904912	0.625	195.6371
13	3D	AX-109990300	0.625	195.6371
13	3D	AX-110614729	0.625	195.6371
13	3D	AX-111656746	0.625	195.6371
13	3D	AX-110443973	0.625	195.6371
13	3D	AX-111713538	0.625	195.6371
13	3D	AX-109965035	0.625	195.6371
13	3D	AX-110941738	0.625	195.6371
13	3D	AX-109427321	0.625	195.6371
13	3D	AX-110614380	0.625	195.6371
13	3D	AX-109883347	0.625	195.6371
13	3D	AX-108837398	0.625	195.6371
13	3D	AX-109070519	0.625	195.6371
13	3D	AX-109867219	0.625	195.6371
13	3D	AX-110458694	0.625	195.6371
13	3D	AX-109680288	0.625	195.6371
13	3D	AX-109970404	0.625	195.6371
13	3D	AX-111636633	0.625	195.6371
13	3D	AX-109184287	0.625	195.6371
13	3D	AX-110536191	0.625	195.6371
13	3D	AX-110500298	0.625	195.6371
13	3D	AX-110674320	0.625	195.6371
13	3D	AX-110675479	0.625	195.6371
13	3D	AX-109372173	0.625	195.6371
13	3D	AX-109922315	0.625	195.6371
13	3D	AX-111163626	0.625	195.6371
13	3D	AX-109958726	0.625	195.6371
13	3D	AX-110768675	0.625	195.6371
13	3D	AX-109584432	0.625	195.6371
13	3D	AX-110904891	0.625	195.6371
13	3D	AX-108896957	0.625	195.6371
13	3D	AX-110539269	0.625	195.6371
13	3D	AX-110457255	0.625	195.6371
13	3D	AX-109494245	0.625	195.6371
13	3D	AX-110939237	0.625	195.6371
13	3D	AX-111453594	0.625	195.6371
13	3D	AX-110588848	0.625	195.6371
13	3D	AX-111002950	0.625	195.6371
13	3D	AX-111373231	0.625	195.6371
13	3D	AX-110409385	0.625	195.6371

13	3D	AX-109408582	0.625	195.6371
13	3D	AX-108976511	0.625	195.6371
13	3D	AX-111215689	0.625	195.6371
13	3D	AX-110211661	0.625	195.6371
13	3D	AX-110024196	0.625	195.6371
13	3D	AX-109465614	0.625	195.6371
13	3D	AX-111629157	0.625	195.6371
13	3D	AX-109835960	0.625	195.6371
13	3D	AX-110000365	0.625	195.6371
13	3D	AX-110271347	0.625	195.6371
13	3D	AX-111011888	0.625	195.6371
13	3D	AX-108752087	0.625	195.6371
13	3D	AX-108835424	0.625	195.6371
13	3D	AX-109450262	0.625	195.6371
13	3D	AX-109503229	0.625	195.6371
13	3D	AX-109640261	0.625	195.6371
13	3D	AX-111171142	0.625	195.6371
13	3D	AX-111619124	0.625	195.6371
13	3D	AX-110924127	0.2049	195.842
13	3D	AX-110917154	0.2049	195.842
13	3D	AX-111027145	0.2049	195.842
13	3D	AX-110450306	0.2049	195.842
13	3D	AX-110408939	0.2049	195.842
13	3D	AX-108728766	0.2049	195.842
13	3D	AX-111648451	0.2049	195.842
13	3D	AX-109181584	0.2049	195.842
13	3D	AX-109440982	0.2049	195.842
13	3D	AX-109873102	0.2049	195.842
13	3D	AX-109163139	0.2049	195.842
13	3D	AX-109864940	0.2049	195.842
13	3D	AX-111131666	0.2049	195.842
13	3D	AX-109399883	0.2049	195.842
13	3D	AX-110237521	0.2049	195.842
13	3D	AX-110987710	0.2049	195.842
13	3D	AX-110502546	0.2049	195.842
13	3D	AX-110951495	0.2049	195.842
13	3D	AX-108761723	0.2049	195.842
13	3D	AX-111559002	0.2049	195.842
13	3D	AX-108779450	0.2049	195.842
13	3D	AX-109731594	0.2049	195.842
13	3D	AX-110509782	0.2049	195.842
13	3D	AX-110535689	0.2049	195.842
13	3D	AX-108744309	0.2049	195.842
13	3D	AX-86178858	0.2049	195.842

13	3D	AX-110960653	0.2049	195.842
13	3D	AX-94550605	0.2049	195.842
13	3D	AX-109192426	0.2049	195.842
13	3D	AX-109298013	0.2049	195.842
13	3D	AX-111760407	0.2049	195.842
13	3D	AX-108729723	0.2049	195.842
13	3D	AX-110943935	0.2049	195.842
13	3D	AX-111059658	0.2049	195.842
13	3D	AX-95632485	0.2049	195.842
13	3D	AX-109206972	0.2049	195.842
13	3D	AX-109696871	0.2049	195.842
13	3D	AX-110060687	0.2049	195.842
13	3D	AX-110520298	0.2049	195.842
13	3D	AX-109811547	0.2049	195.842
13	3D	AX-111548568	0.2049	195.842
13	3D	AX-95630873	0.2049	195.842
13	3D	AX-109328189	0.2049	195.842
13	3D	AX-109467143	0.2049	195.842
13	3D	AX-111452045	0.2049	195.842
13	3D	AX-111656195	0.2049	195.842
13	3D	AX-108926832	0.2049	195.842
13	3D	AX-94923751	0.2049	195.842
13	3D	AX-111905040	0.2049	195.842
13	3D	AX-109765778	0.2049	195.842
13	3D	AX-111048798	0.2049	195.842
13	3D	AX-109331230	0.2049	195.842
13	3D	AX-110392760	0.2049	195.842
13	3D	AX-111072023	0.2049	195.842
13	3D	AX-110004881	0.2049	195.842
13	3D	AX-109657682	0.2049	195.842
13	3D	AX-108872079	0.2049	195.842
13	3D	AX-110533153	0.2049	195.842
13	3D	AX-111366210	0.2049	195.842
13	3D	AX-111536462	0.2049	195.842
13	3D	AX-109376383	0.2049	195.842
13	3D	AX-109387530	0.2049	195.842
13	3D	AX-109934737	0.2049	195.842
13	3D	AX-109354257	0.2049	195.842
13	3D	AX-109490029	0.2049	195.842
13	3D	AX-111524627	0.2049	195.842
13	3D	AX-109790588	0.2049	195.842
13	3D	AX-109930626	0.2049	195.842
13	3D	AX-110385364	0.2049	195.842
13	3D	AX-109518377	0.2049	195.842

13	3D	AX-94812690	0.2049	195.842
14	4A	AX-108744862	0	0
14	4A	AX-108891576	0	0
14	4A	AX-108771516	4.6429	4.6429
14	4A	AX-108785329	29.956	34.599
14	4A	AX-111099057	1.0919	35.6909
14	4A	AX-109435078	2.7176	38.4085
14	4A	AX-110502952	2.7176	38.4085
14	4A	AX-111495728	2.7176	38.4085
14	4A	AX-110731612	2.7176	38.4085
14	4A	AX-110486280	2.7176	38.4085
14	4A	AX-109454520	2.7176	38.4085
14	4A	AX-111081911	2.7176	38.4085
14	4A	AX-110580622	2.7176	38.4085
14	4A	AX-109583703	2.7176	38.4085
14	4A	AX-111518497	0.4292	38.8377
14	4A	AX-89767898	0.4292	38.8377
14	4A	AX-110387713	1.3103	40.148
14	4A	AX-109426161	0.2137	40.3617
14	4A	AX-108798424	0.2137	40.3617
14	4A	AX-110939318	2.2138	42.5755
14	4A	AX-110524110	2.2138	42.5755
14	4A	AX-111645255	2.2138	42.5755
14	4A	AX-109094457	0.6466	43.2221
14	4A	AX-110447967	0.6466	43.2221
14	4A	AX-109931953	0.6466	43.2221
14	4A	AX-109913427	4.157	47.3791
14	4A	AX-109505335	4.157	47.3791
14	4A	AX-108944004	4.157	47.3791
14	4A	AX-86164084	4.157	47.3791
14	4A	AX-110916302	0.8548	48.2339
14	4A	AX-111215202	0.8548	48.2339
14	4A	AX-110678770	0.8548	48.2339
14	4A	AX-109847237	0.8548	48.2339
14	4A	AX-111475297	0.8548	48.2339
14	4A	AX-110008111	0.8548	48.2339
14	4A	AX-108836837	0.8548	48.2339
14	4A	AX-109060506	0.8548	48.2339
14	4A	AX-108835358	0.8548	48.2339
14	4A	AX-110914376	0.8475	49.0815
14	4A	AX-109417674	0.8475	49.0815
14	4A	AX-108819593	1.7474	50.8289
14	4A	AX-109281664	0.6383	51.4672
14	4A	AX-111077580	0.6383	51.4672

14	4A	AX-111643250	0.6383	51.4672
14	4A	AX-108765759	0.6383	51.4672
14	4A	AX-110025778	0.6383	51.4672
14	4A	AX-109058636	0.6383	51.4672
14	4A	AX-111462216	0.6383	51.4672
14	4A	AX-110994102	0.6383	51.4672
14	4A	AX-110494698	0.6383	51.4672
14	4A	AX-111524720	0.6383	51.4672
14	4A	AX-111478895	0.6383	51.4672
14	4A	AX-110124177	0.6383	51.4672
14	4A	AX-111019523	0.6383	51.4672
14	4A	AX-109919545	0.6383	51.4672
14	4A	AX-111034384	0.4202	51.8874
14	4A	AX-110604181	0.4202	51.8874
14	4A	AX-110677765	0.4202	51.8874
14	4A	AX-109825947	0.4202	51.8874
14	4A	AX-109491044	0.4202	51.8874
14	4A	AX-108995050	0.8475	52.7349
14	4A	AX-109968652	0.6383	53.3733
14	4A	AX-108939255	0.8511	54.2244
14	4A	AX-109352388	0.8511	54.2244
14	4A	AX-110426823	0.8511	54.2244
14	4A	AX-109949162	0.8511	54.2244
14	4A	AX-109461332	0.8511	54.2244
14	4A	AX-108766824	0.8511	54.2244
14	4A	AX-110537499	0.8511	54.2244
14	4A	AX-110374554	0.8511	54.2244
14	4A	AX-111213019	0.8511	54.2244
14	4A	AX-109407902	0.8511	54.2244
14	4A	AX-108954112	0.8511	54.2244
14	4A	AX-110057904	0.8511	54.2244
14	4A	AX-109316361	0.8511	54.2244
14	4A	AX-109821994	0.8511	54.2244
14	4A	AX-111665247	0.8511	54.2244
14	4A	AX-110423307	0.8511	54.2244
14	4A	AX-111168060	0.8511	54.2244
14	4A	AX-110441897	0.6356	54.86
14	4A	AX-109375154	1.9747	56.8347
14	4A	AX-108955453	1.9747	56.8347
14	4A	AX-108994889	1.7248	58.5596
14	4A	AX-109926179	0.2083	58.7679
14	4A	AX-110422223	0.2083	58.9762
14	4A	AX-111023486	0.2066	59.1828
14	4A	AX-111524868	0.4167	59.5995

14	4A	AX-111124943	0.6303	60.2298
14	4A	AX-111170340	0.4167	60.6465
14	4A	AX-108883521	0.4167	60.6465
14	4A	AX-109505967	0.4167	60.6465
14	4A	AX-111140411	0.4167	60.6465
14	4A	AX-108889870	0.4167	60.6465
14	4A	AX-111114543	0.4167	60.6465
14	4A	AX-108772504	0.4167	60.6465
14	4A	AX-111500116	0.4167	60.6465
14	4A	AX-111634994	0.4167	60.6465
14	4A	AX-109931552	0.4167	61.0632
14	4A	AX-108746349	0.4167	61.0632
14	4A	AX-109474732	0.4167	61.4798
14	4A	AX-111584564	1.2661	62.7459
14	4A	AX-110503209	0.2066	62.9525
14	4A	AX-109865536	0.625	63.5776
14	4A	AX-111062067	0.625	63.5776
14	4A	AX-108829087	0.625	63.5776
14	4A	AX-110030140	0.625	63.5776
14	4A	AX-111031745	0.625	63.5776
14	4A	AX-108751023	0.625	63.5776
14	4A	AX-108878533	0.625	63.5776
14	4A	AX-109411376	0.844	64.4215
14	4A	AX-110050277	1.4962	65.9177
14	4A	AX-108997961	0.8369	66.7546
14	4A	AX-108853924	0.8369	66.7546
14	4A	AX-109479872	0.8369	66.7546
14	4A	AX-112288640	0.8369	66.7546
14	4A	AX-110124300	0.2066	66.9612
14	4A	AX-110734264	0.2066	66.9612
14	4A	AX-108887304	0.2066	66.9612
14	4A	AX-109841999	0.2066	66.9612
14	4A	AX-109302214	0.2066	66.9612
14	4A	AX-110956232	0.2066	67.1678
14	4A	AX-110952189	0.2066	67.1678
14	4A	AX-109915676	0.2066	67.1678
14	4A	AX-111464620	0.2066	67.1678
14	4A	AX-109485046	0.2066	67.1678
14	4A	AX-111492756	0.2066	67.1678
14	4A	AX-109325747	0.2066	67.1678
14	4A	AX-111147041	0.2066	67.1678
14	4A	AX-110741288	0.2083	67.3762
14	4A	AX-109980263	0.2083	67.3762
14	4A	AX-95220409	0.4202	67.7963

14	4A	AX-111679727	0.4202	67.7963
14	4A	AX-111282900	0.4202	67.7963
14	4A	AX-110566565	0.4202	67.7963
14	4A	AX-111190701	0.4202	67.7963
14	4A	AX-110709253	0.4202	67.7963
14	4A	AX-111072987	0.4202	67.7963
14	4A	AX-110125311	0.4202	67.7963
14	4A	AX-108880838	0.4202	67.7963
14	4A	AX-109323225	0.4202	67.7963
14	4A	AX-111587091	0.4202	67.7963
14	4A	AX-110544730	0.4202	67.7963
14	4A	AX-109940925	0.4202	67.7963
14	4A	AX-108891704	0.4202	67.7963
14	4A	AX-109872431	0.4202	67.7963
14	4A	AX-111635462	0.4202	67.7963
14	4A	AX-110541338	0.4202	67.7963
14	4A	AX-109042131	0.4202	67.7963
14	4A	AX-109306055	0.4202	67.7963
14	4A	AX-110493583	0.4202	67.7963
14	4A	AX-109908394	0.4202	67.7963
14	4A	AX-109983703	0.4202	67.7963
14	4A	AX-109965750	0.4202	67.7963
14	4A	AX-110912744	0.4202	67.7963
14	4A	AX-111474643	0.4202	67.7963
14	4A	AX-109581996	0.4202	67.7963
14	4A	AX-108899021	0.4202	67.7963
14	4A	AX-110554754	0.4202	67.7963
14	4A	AX-111517822	0.4202	67.7963
14	4A	AX-109818545	0.4202	67.7963
14	4A	AX-109506032	0.4202	67.7963
14	4A	AX-111586855	0.4202	67.7963
14	4A	AX-110951928	0.4202	67.7963
14	4A	AX-111546095	0.4202	67.7963
14	4A	AX-109275919	0.4202	67.7963
14	4A	AX-111666924	0.4202	67.7963
14	4A	AX-108915433	0.4202	67.7963
14	4A	AX-108744833	0.4202	67.7963
14	4A	AX-111280267	0.4202	67.7963
14	4A	AX-109834878	0.4202	67.7963
14	4A	AX-108735691	0.4202	67.7963
14	4A	AX-110474640	0.4202	67.7963
14	4A	AX-110959847	0.4202	67.7963
14	4A	AX-109470652	0.4202	67.7963
14	4A	AX-110950494	0.4202	67.7963

14	4A	AX-110453458	0.4202	67.7963
14	4A	AX-109386626	0.4202	67.7963
14	4A	AX-109866293	0.4202	67.7963
14	4A	AX-108749529	0.4202	67.7963
14	4A	AX-110667203	0.4202	67.7963
14	4A	AX-111609312	0.4202	67.7963
14	4A	AX-110407962	0.4202	67.7963
14	4A	AX-108906873	0.4202	67.7963
14	4A	AX-109354728	0.4202	67.7963
14	4A	AX-111013232	0.4202	67.7963
14	4A	AX-108899041	0.4202	67.7963
14	4A	AX-108795861	0.4202	67.7963
14	4A	AX-109981085	0.4202	67.7963
14	4A	AX-111122406	0.4202	67.7963
14	4A	AX-111027313	0.4202	67.7963
14	4A	AX-111039466	0.4202	67.7963
14	4A	AX-109352963	0.4202	67.7963
14	4A	AX-110474907	0.4202	67.7963
14	4A	AX-108988478	0.4202	67.7963
14	4A	AX-109330895	0.4202	67.7963
14	4A	AX-110013403	0.4202	67.7963
14	4A	AX-110994848	0.4202	67.7963
14	4A	AX-110517636	0.4202	67.7963
14	4A	AX-108776922	0.4202	67.7963
14	4A	AX-110173175	0.4202	67.7963
14	4A	AX-108762310	0.4202	67.7963
14	4A	AX-109459388	0.4202	67.7963
14	4A	AX-109304437	0.4202	67.7963
14	4A	AX-109849616	0.4202	67.7963
14	4A	AX-109906466	0.4202	67.7963
14	4A	AX-111009407	0.4202	67.7963
14	4A	AX-110404514	0.4202	67.7963
14	4A	AX-108806138	0.4202	67.7963
14	4A	AX-108843258	0.4202	67.7963
14	4A	AX-110667940	0.4202	67.7963
14	4A	AX-110016372	0.4202	67.7963
14	4A	AX-110417495	0.4202	67.7963
14	4A	AX-108841569	0.4202	67.7963
14	4A	AX-110410959	0.4202	67.7963
14	4A	AX-111067996	0.4202	67.7963
14	4A	AX-110626009	0.4202	67.7963
14	4A	AX-109624156	0.4202	67.7963
14	4A	AX-110435839	0.4202	67.7963
14	4A	AX-108925777	0.4202	67.7963

14	4A	AX-110001522	0.4202	67.7963
14	4A	AX-109374723	0.4202	67.7963
14	4A	AX-110981982	0.4202	67.7963
14	4A	AX-111550497	0.4202	67.7963
14	4A	AX-108843338	0.4202	67.7963
14	4A	AX-111452463	0.4202	67.7963
14	4A	AX-108842133	0.4202	67.7963
14	4A	AX-111637782	0.4202	67.7963
14	4A	AX-111604874	0.4202	67.7963
14	4A	AX-109366065	0.4202	67.7963
14	4A	AX-110934206	0.4202	67.7963
14	4A	AX-110649830	0.4202	67.7963
14	4A	AX-109853543	0.4202	67.7963
14	4A	AX-108903142	0.4202	67.7963
14	4A	AX-108761059	0.4202	67.7963
14	4A	AX-110923992	0.4202	67.7963
14	4A	AX-111566856	0.4202	67.7963
14	4A	AX-111072858	0.4202	67.7963
14	4A	AX-108997566	0.4202	67.7963
14	4A	AX-109559120	0.4202	67.7963
14	4A	AX-109327802	0.4202	67.7963
14	4A	AX-108878081	0.4202	67.7963
14	4A	AX-111099020	0.4202	67.7963
14	4A	AX-111113230	0.4202	67.7963
14	4A	AX-109038221	0.4202	67.7963
14	4A	AX-111622831	0.4202	67.7963
14	4A	AX-110570482	0.4202	67.7963
14	4A	AX-110414049	0.4202	67.7963
14	4A	AX-111043895	0.4202	67.7963
14	4A	AX-109974355	0.4202	67.7963
14	4A	AX-110089605	0.4202	67.7963
14	4A	AX-109586259	0.4202	67.7963
14	4A	AX-109037326	0.4202	67.7963
14	4A	AX-111562303	0.4202	67.7963
14	4A	AX-109998679	0.4202	67.7963
14	4A	AX-111006003	0.4202	67.7963
14	4A	AX-110411500	0.4202	67.7963
14	4A	AX-109940003	0.4202	67.7963
14	4A	AX-110426548	0.4202	67.7963
14	4A	AX-109012904	0.4202	67.7963
14	4A	AX-111083842	0.4202	67.7963
14	4A	AX-108791064	0.4202	67.7963
14	4A	AX-108816968	0.4202	67.7963
14	4A	AX-108751386	0.4202	67.7963

14	4A	AX-110364487	0.4202	67.7963
14	4A	AX-109491833	0.4202	67.7963
14	4A	AX-110376411	0.4202	67.7963
14	4A	AX-109448064	0.4202	67.7963
14	4A	AX-110360228	0.4202	67.7963
14	4A	AX-109445606	0.4202	67.7963
14	4A	AX-110695054	0.4202	67.7963
14	4A	AX-111066000	0.4202	67.7963
14	4A	AX-109418813	0.4202	67.7963
14	4A	AX-109627140	0.4202	67.7963
14	4A	AX-111000772	0.4202	67.7963
14	4A	AX-111660154	0.4202	67.7963
14	4A	AX-108799881	0.4202	67.7963
14	4A	AX-110039247	0.4202	67.7963
14	4A	AX-109324565	0.4202	67.7963
14	4A	AX-110944795	0.4202	67.7963
14	4A	AX-110037099	0.4202	67.7963
14	4A	AX-108829120	0.4202	67.7963
14	4A	AX-109904490	0.4202	67.7963
14	4A	AX-110954224	0.4202	67.7963
14	4A	AX-109897504	0.4202	67.7963
14	4A	AX-110448656	0.4202	67.7963
14	4A	AX-109055595	0.4202	67.7963
14	4A	AX-111261997	0.4202	67.7963
14	4A	AX-109507052	0.4202	67.7963
14	4A	AX-111647014	0.4202	67.7963
14	4A	AX-108759750	0.4202	67.7963
14	4A	AX-111532701	0.4202	67.7963
14	4A	AX-110122140	0.4202	67.7963
14	4A	AX-108969300	0.4202	67.7963
14	4A	AX-110385550	0.4202	67.7963
14	4A	AX-110585645	0.4202	67.7963
14	4A	AX-111156065	0.4202	67.7963
14	4A	AX-110595501	0.4202	67.7963
14	4A	AX-111540931	0.4202	67.7963
14	4A	AX-108949564	0.4202	67.7963
14	4A	AX-110520060	0.4202	67.7963
14	4A	AX-110557442	0.4202	67.7963
14	4A	AX-111035342	0.4202	67.7963
14	4A	AX-111126855	0.4202	67.7963
14	4A	AX-108974129	0.4202	67.7963
14	4A	AX-108798137	0.4202	67.7963
14	4A	AX-111564930	0.4202	67.7963
14	4A	AX-110145331	0.4202	67.7963

14	4A	AX-110465475	0.4202	67.7963
14	4A	AX-111027522	0.4202	67.7963
14	4A	AX-111644366	0.4202	67.7963
14	4A	AX-110548489	0.4202	67.7963
14	4A	AX-110987247	0.4202	67.7963
14	4A	AX-111117403	0.4202	67.7963
14	4A	AX-109420461	0.4202	67.7963
14	4A	AX-110561097	0.4202	67.7963
14	4A	AX-109932163	0.4202	67.7963
14	4A	AX-110417316	0.4202	67.7963
14	4A	AX-111097741	0.4202	67.7963
14	4A	AX-110431442	0.4202	67.7963
14	4A	AX-109829683	0.4202	67.7963
14	4A	AX-108792582	0.4202	67.7963
14	4A	AX-109863042	0.4202	67.7963
14	4A	AX-111631790	0.4202	67.7963
14	4A	AX-110501591	0.4202	67.7963
14	4A	AX-111255507	0.4202	67.7963
14	4A	AX-109992103	0.4202	67.7963
14	4A	AX-110712137	0.4202	67.7963
14	4A	AX-108759857	0.4202	67.7963
14	4A	AX-110575653	0.4202	67.7963
14	4A	AX-109500939	0.4202	67.7963
14	4A	AX-111039534	0.4202	67.7963
14	4A	AX-109911541	0.4202	67.7963
14	4A	AX-111614310	0.4202	67.7963
14	4A	AX-111061317	0.4202	67.7963
14	4A	AX-109382518	0.4202	67.7963
14	4A	AX-110982793	0.4202	67.7963
14	4A	AX-109272207	0.4202	67.7963
14	4A	AX-110563448	0.4202	67.7963
14	4A	AX-111631423	0.4202	67.7963
14	4A	AX-110686803	0.4202	67.7963
14	4A	AX-108827687	0.4202	67.7963
14	4A	AX-108892728	0.4202	67.7963
14	4A	AX-108806720	0.4202	67.7963
14	4A	AX-109417418	0.4202	67.7963
14	4A	AX-110991493	0.4202	67.7963
14	4A	AX-109285343	0.4202	67.7963
14	4A	AX-111612796	0.4202	67.7963
14	4A	AX-111143353	0.4202	67.7963
14	4A	AX-110924773	0.4202	67.7963
14	4A	AX-109274569	0.4202	67.7963
14	4A	AX-111162220	0.4202	67.7963

14	4A	AX-110075308	0.4202	67.7963
14	4A	AX-109536201	0.4202	67.7963
14	4A	AX-110366357	0.4202	67.7963
14	4A	AX-110955621	0.4202	67.7963
14	4A	AX-110927258	0.4202	67.7963
14	4A	AX-108931664	0.4202	67.7963
14	4A	AX-111492177	0.4202	67.7963
14	4A	AX-110474951	0.4202	67.7963
14	4A	AX-110015660	0.4202	67.7963
14	4A	AX-108902669	0.4202	67.7963
14	4A	AX-108995460	0.4202	67.7963
14	4A	AX-109948751	0.4202	67.7963
14	4A	AX-111653083	0.4202	67.7963
14	4A	AX-111558102	0.4202	67.7963
14	4A	AX-111012476	0.4202	67.7963
14	4A	AX-109466221	0.4202	67.7963
14	4A	AX-109930426	0.4202	67.7963
14	4A	AX-109901431	0.4202	67.7963
14	4A	AX-108827637	0.4202	67.7963
14	4A	AX-108956519	0.4202	67.7963
14	4A	AX-111165448	0.4202	67.7963
14	4A	AX-110142768	0.4202	67.7963
14	4A	AX-111582445	0.4202	67.7963
14	4A	AX-111042590	0.4202	67.7963
14	4A	AX-111082825	0.4202	67.7963
14	4A	AX-109335943	0.4202	67.7963
14	4A	AX-111494122	0.4202	67.7963
14	4A	AX-111218915	0.4202	67.7963
14	4A	AX-109522359	0.4202	67.7963
14	4A	AX-110564633	0.4202	67.7963
14	4A	AX-108800004	0.4202	67.7963
14	4A	AX-109987959	0.4202	67.7963
14	4A	AX-110954698	0.4202	67.7963
14	4A	AX-108985287	0.4202	67.7963
14	4A	AX-108770779	0.4202	67.7963
14	4A	AX-111452507	0.4202	67.7963
14	4A	AX-108903206	0.4202	67.7963
14	4A	AX-109649762	0.4202	67.7963
14	4A	AX-109936618	0.4202	67.7963
14	4A	AX-110054269	0.4202	67.7963
14	4A	AX-109465075	0.4202	67.7963
14	4A	AX-111028663	0.4202	67.7963
14	4A	AX-109389796	0.4202	67.7963
14	4A	AX-109443898	0.4202	67.7963

14	4A	AX-111504010	0.4202	67.7963
14	4A	AX-111215500	0.4202	67.7963
14	4A	AX-109578468	0.4202	67.7963
14	4A	AX-109988396	0.4202	67.7963
14	4A	AX-110964053	0.4202	67.7963
14	4A	AX-110564410	0.4202	67.7963
14	4A	AX-111577888	0.4202	67.7963
14	4A	AX-109478972	0.4202	67.7963
14	4A	AX-110603698	0.4202	67.7963
14	4A	AX-110546873	0.4202	67.7963
14	4A	AX-108942360	0.4202	67.7963
14	4A	AX-111510741	0.4202	67.7963
14	4A	AX-109883746	0.4202	67.7963
14	4A	AX-109836972	0.4202	67.7963
14	4A	AX-110906570	0.4202	67.7963
14	4A	AX-109831483	0.4202	67.7963
14	4A	AX-109844186	0.4202	67.7963
14	4A	AX-109059831	0.4202	67.7963
14	4A	AX-109490587	0.4202	67.7963
14	4A	AX-108851532	0.4202	67.7963
14	4A	AX-111520514	0.4202	67.7963
14	4A	AX-111010105	0.4202	67.7963
14	4A	AX-110063347	0.4202	67.7963
14	4A	AX-109940964	0.4202	67.7963
14	4A	AX-109899762	0.4202	67.7963
14	4A	AX-110452830	0.4202	67.7963
14	4A	AX-111110092	0.4202	67.7963
14	4A	AX-111550922	0.4202	67.7963
14	4A	AX-110443047	0.4202	67.7963
14	4A	AX-109479061	0.4202	67.7963
14	4A	AX-110140989	0.4202	67.7963
14	4A	AX-108810926	0.4202	67.7963
14	4A	AX-110485569	0.4202	67.7963
14	4A	AX-109473564	0.4202	67.7963
14	4A	AX-109887699	0.4202	67.7963
14	4A	AX-109557696	0.4202	67.7963
14	4A	AX-109394720	0.4202	67.7963
14	4A	AX-111658485	0.4202	67.7963
14	4A	AX-110972423	0.4202	67.7963
14	4A	AX-111456706	0.4202	67.7963
14	4A	AX-110930247	0.4202	67.7963
14	4A	AX-109385165	0.4202	67.7963
14	4A	AX-108734424	0.4202	67.7963
14	4A	AX-109321866	0.4202	67.7963

14	4A	AX-111627667	0.4202	67.7963
14	4A	AX-111009968	0.4202	67.7963
14	4A	AX-109366086	0.4202	67.7963
14	4A	AX-111517366	0.4202	67.7963
14	4A	AX-109033918	0.4202	67.7963
14	4A	AX-110086222	0.4202	67.7963
14	4A	AX-110930608	0.4202	67.7963
14	4A	AX-108785895	0.4202	67.7963
14	4A	AX-111091521	0.4202	67.7963
14	4A	AX-110460536	0.4202	67.7963
14	4A	AX-110568569	0.4202	67.7963
14	4A	AX-111047367	0.4202	67.7963
14	4A	AX-108948430	0.4202	67.7963
14	4A	AX-109478824	0.4202	67.7963
14	4A	AX-111487803	0.4202	67.7963
14	4A	AX-110439281	0.4202	67.7963
14	4A	AX-111455414	0.4202	67.7963
14	4A	AX-110467452	0.4202	67.7963
14	4A	AX-110383389	0.4202	67.7963
14	4A	AX-110990362	0.4202	67.7963
14	4A	AX-111039793	0.4202	67.7963
14	4A	AX-111527637	0.4202	67.7963
14	4A	AX-110448759	0.4202	67.7963
14	4A	AX-110484897	0.4202	67.7963
14	4A	AX-109310591	0.4202	67.7963
14	4A	AX-109951289	0.4202	67.7963
14	4A	AX-109917369	0.4202	67.7963
14	4A	AX-109835894	0.4202	67.7963
14	4A	AX-110989445	0.4202	67.7963
14	4A	AX-111591589	0.4202	67.7963
14	4A	AX-111123263	0.4202	67.7963
14	4A	AX-109919834	0.4202	67.7963
14	4A	AX-111626548	0.4202	67.7963
14	4A	AX-108791065	0.4202	67.7963
14	4A	AX-111004006	0.4202	67.7963
14	4A	AX-108806672	0.4202	67.7963
14	4A	AX-111139264	0.4202	67.7963
14	4A	AX-110645577	0.4202	67.7963
14	4A	AX-110578111	0.4202	67.7963
14	4A	AX-110939894	0.4202	67.7963
14	4A	AX-111625042	0.4202	67.7963
14	4A	AX-109301139	0.4202	67.7963
14	4A	AX-109393001	0.4202	67.7963
14	4A	AX-111168139	0.4202	67.7963

14	4A	AX-111542148	0.4202	67.7963
14	4A	AX-109993853	0.4202	67.7963
14	4A	AX-111522375	0.4202	67.7963
14	4A	AX-110437706	0.4202	67.7963
14	4A	AX-110908601	0.4202	67.7963
14	4A	AX-111258230	0.4202	67.7963
14	4A	AX-110990610	0.4202	67.7963
14	4A	AX-109405494	0.4202	67.7963
14	4A	AX-108886559	0.4202	67.7963
14	4A	AX-109911543	0.4202	67.7963
14	4A	AX-110371373	0.4202	67.7963
14	4A	AX-109323518	0.4202	67.7963
14	4A	AX-109336941	0.4202	67.7963
14	4A	AX-111152914	0.4202	67.7963
14	4A	AX-111141923	0.4202	67.7963
14	4A	AX-109584620	0.4202	67.7963
14	4A	AX-109886151	0.4202	67.7963
14	4A	AX-109835076	0.4202	67.7963
14	4A	AX-109940361	0.4202	67.7963
14	4A	AX-111143443	0.4202	67.7963
14	4A	AX-111255436	0.4202	67.7963
14	4A	AX-108761870	0.4202	67.7963
14	4A	AX-108962808	0.4202	67.7963
14	4A	AX-110035014	0.4202	67.7963
14	4A	AX-110391635	0.4202	67.7963
14	4A	AX-111119047	0.4202	67.7963
14	4A	AX-110556325	0.4202	67.7963
14	4A	AX-109937843	0.4202	67.7963
14	4A	AX-111637906	0.4202	67.7963
14	4A	AX-111067027	0.4202	67.7963
14	4A	AX-110962575	0.4202	67.7963
14	4A	AX-109533701	0.4202	67.7963
14	4A	AX-110070806	0.4202	67.7963
14	4A	AX-109078046	0.4202	67.7963
14	4A	AX-108754654	0.4202	67.7963
14	4A	AX-108831077	0.4202	67.7963
14	4A	AX-109912394	0.4202	67.7963
14	4A	AX-109962698	0.4202	67.7963
14	4A	AX-111043288	0.4202	67.7963
14	4A	AX-111048472	0.4202	67.7963
14	4A	AX-111560935	0.4202	67.7963
14	4A	AX-111632091	0.4202	67.7963
14	4A	AX-94991164	0.4202	67.7963
14	4A	AX-108905378	0.4202	67.7963

14	4A	AX-109853300	0.4202	67.7963
14	4A	AX-110109210	0.4202	67.7963
14	4A	AX-110361588	0.4202	67.7963
14	4A	AX-110484635	0.4202	67.7963
14	4A	AX-86172722	0.4202	67.7963
14	4A	AX-108775441	0.4202	67.7963
14	4A	AX-108734452	0.4202	67.7963
14	4A	AX-109507450	0.4202	67.7963
14	4A	AX-110535966	0.4202	67.7963
14	4A	AX-110618464	0.4202	67.7963
14	4A	AX-111089399	0.4202	67.7963
14	4A	AX-111653604	0.4202	67.7963
14	4A	AX-111800945	0.4202	67.7963
14	4A	AX-94661891	0.4202	67.7963
14	4A	AX-94969149	0.4202	67.7963
14	4A	AX-95201383	0.4202	67.7963
14	4A	AX-109818088	0.8548	68.6511
14	4A	AX-108784859	0.2101	68.8612
14	4A	AX-109319707	0.2101	68.8612
14	4A	AX-111648865	0.2101	68.8612
14	4A	AX-110043131	0.6276	69.4889
14	4A	AX-109270432	0.6276	69.4889
14	4A	AX-109484919	0.6276	69.4889
14	4A	AX-111144687	0.6276	69.4889
14	4A	AX-110105180	0.6276	69.4889
14	4A	AX-111020436	0.6276	69.4889
14	4A	AX-110978991	0.6276	69.4889
14	4A	AX-108842463	0.6276	69.4889
14	4A	AX-109303147	0.6276	69.4889
14	4A	AX-111233700	0.6276	69.4889
14	4A	AX-108881371	0.6276	69.4889
14	4A	AX-110957374	0.6276	69.4889
14	4A	AX-109930648	0.6276	69.4889
14	4A	AX-110554909	0.6276	69.4889
14	4A	AX-108821917	0.6276	69.4889
14	4A	AX-111088720	0.6276	69.4889
14	4A	AX-109378449	0.6276	69.4889
14	4A	AX-111265391	0.6276	69.4889
14	4A	AX-111268934	0.6276	69.4889
14	4A	AX-110714502	0.6276	69.4889
14	4A	AX-108984505	0.6276	69.4889
14	4A	AX-108887341	0.6276	69.4889
14	4A	AX-111581462	0.6276	69.4889
14	4A	AX-109896933	0.6276	69.4889

14	4A	AX-111473407	0.6276	69.4889
14	4A	AX-110378497	0.6276	69.4889
14	4A	AX-109937358	0.6276	69.4889
14	4A	AX-110613695	0.6276	69.4889
14	4A	AX-108965179	0.6276	69.4889
14	4A	AX-111487200	0.6276	69.4889
14	4A	AX-110534913	0.6276	69.4889
14	4A	AX-108750936	0.6276	69.4889
14	4A	AX-111491720	0.6276	69.4889
14	4A	AX-109479762	0.6276	69.4889
14	4A	AX-110140778	0.6276	69.4889
14	4A	AX-111614846	0.6276	69.4889
14	4A	AX-111135351	0.6276	69.4889
14	4A	AX-109345287	0.6276	69.4889
14	4A	AX-110431843	0.6276	69.4889
14	4A	AX-110599530	0.6276	69.4889
14	4A	AX-110424357	0.6276	69.4889
14	4A	AX-111247327	0.6276	69.4889
14	4A	AX-108776413	0.6276	69.4889
14	4A	AX-111596219	0.6276	69.4889
14	4A	AX-108763086	0.6276	69.4889
14	4A	AX-109474997	0.6276	69.4889
14	4A	AX-109531016	0.6276	69.4889
14	4A	AX-110369586	0.6276	69.4889
14	4A	AX-109300655	0.6276	69.4889
14	4A	AX-111010177	0.6276	69.4889
14	4A	AX-108819037	0.6276	69.4889
14	4A	AX-108798136	0.6276	69.4889
14	4A	AX-110735104	0.6276	69.4889
14	4A	AX-111559227	0.6276	69.4889
14	4A	AX-108734258	0.6276	69.4889
14	4A	AX-110935752	0.6276	69.4889
14	4A	AX-111501575	0.6276	69.4889
14	4A	AX-109935326	0.6276	69.4889
14	4A	AX-111608459	0.6276	69.4889
14	4A	AX-111045592	0.6276	69.4889
14	4A	AX-94432315	0.6276	69.4889
14	4A	AX-94459938	0.6276	69.4889
14	4A	AX-95629315	0.6276	69.4889
14	4A	AX-108892678	0.2101	69.6989
14	4A	AX-108899858	0.2101	69.6989
14	4A	AX-110574688	0.2101	69.909
14	4A	AX-109391536	0.211	70.12
14	4A	AX-110389563	1.2934	71.4134

14	4A	AX-110571030	0.8512	72.2645
14	4A	AX-110021685	0.2092	72.4737
14	4A	AX-108936363	0.2092	72.4737
14	4A	AX-110173837	0.2066	72.6804
14	4A	AX-111254715	0.2066	72.6804
14	4A	AX-110533428	0.2066	72.6804
14	4A	AX-108794783	5.8131	78.4934
14	4A	AX-111548034	5.8131	78.4934
14	4A	AX-108935127	5.8131	78.4934
14	4A	AX-109629907	0.6329	79.1264
14	4A	AX-109379589	0.8512	79.9775
14	4A	AX-111604302	4.6001	84.5776
14	4A	AX-110557238	4.6001	84.5776
14	4A	AX-110671320	4.6001	84.5776
14	4A	AX-109508535	1.064	85.6416
14	4A	AX-109947264	1.7101	87.3517
14	4A	AX-108959856	1.7101	87.3517
14	4A	AX-111070169	1.055	88.4067
14	4A	AX-108774567	1.055	88.4067
14	4A	AX-111642585	1.055	88.4067
14	4A	AX-110126187	1.924	90.3307
14	4A	AX-109518754	1.2769	91.6076
14	4A	AX-110946610	1.5026	93.1102
14	4A	AX-109509317	0.6329	93.7431
14	4A	AX-110484132	0.6329	93.7431
14	4A	AX-110914112	0.422	94.1651
14	4A	AX-111015902	1.9747	96.1398
14	4A	AX-109589014	0.2092	96.349
14	4A	AX-111493275	0.2092	96.349
14	4A	AX-111053500	0.2092	96.349
14	4A	AX-110432011	0.2092	96.349
14	4A	AX-109456475	0.422	96.771
14	4A	AX-111537186	0.2101	96.981
14	4A	AX-109830528	0.2101	96.981
14	4A	AX-110487724	0.2101	96.981
14	4A	AX-111548209	0.2101	96.981
14	4A	AX-111093305	0.2101	96.981
14	4A	AX-111056819	2.6574	99.6384
14	4A	AX-109818753	2.6574	99.6384
14	4A	AX-108970603	2.6574	99.6384
14	4A	AX-89606215	2.6574	99.6384
14	4A	AX-109451589	0.2083	99.8467
14	4A	AX-109489523	0.2083	99.8467
14	4A	AX-110565260	0.4292	100.2759

14	4A	AX-108907490	0.4386	100.7145
14	4A	AX-110102976	0.8659	101.5804
14	4A	AX-111054150	0.2101	101.7905
14	4A	AX-110492377	2.2138	104.0044
14	4A	AX-109875904	1.5289	105.5332
14	4A	AX-111256422	0.211	105.7442
14	4A	AX-110579138	0.211	105.7442
14	4A	AX-109963277	0.211	105.7442
14	4A	AX-111052249	0.2092	105.9534
14	4A	AX-111080657	0.2075	106.1609
14	4A	AX-111118482	0.2075	106.1609
14	4A	AX-110907767	0.2075	106.1609
14	4A	AX-111529483	0.2075	106.1609
14	4A	AX-110463783	0.2075	106.1609
14	4A	AX-109440196	0.2075	106.1609
14	4A	AX-111074205	0.2075	106.1609
14	4A	AX-108819003	0.2075	106.1609
14	4A	AX-108812767	0.2083	106.3692
14	4A	AX-109491342	0.2083	106.3692
14	4A	AX-110100721	0.2083	106.3692
14	4A	AX-109950963	0.4348	106.804
14	4A	AX-111479307	2.2743	109.0783
14	4A	AX-110497944	1.064	110.1423
14	4A	AX-110122247	1.064	110.1423
14	4A	AX-110587726	1.064	110.1423
14	4A	AX-110008463	0.2058	110.348
14	4A	AX-110372380	4.9224	115.2704
14	4A	AX-111465498	0.2119	115.4823
14	4A	AX-110606045	0.2119	115.4823
14	4A	AX-110407607	0.2083	115.6906
14	4A	AX-111776748	0.2083	115.6906
14	4A	AX-110567219	0.2083	115.6906
14	4A	AX-111514117	0.2083	115.6906
14	4A	AX-109936805	0.2083	115.6906
14	4A	AX-109289084	0.4167	116.1073
14	4A	AX-109272432	0.4167	116.1073
14	4A	AX-111151933	0.4167	116.1073
14	4A	AX-110649064	0.6303	116.7376
14	4A	AX-110523957	0.6303	117.3679
14	4A	AX-111170720	0.6303	117.3679
14	4A	AX-111069221	0.6303	117.3679
14	4A	AX-109916617	0.6303	117.3679
14	4A	AX-111109184	0.6303	117.3679
14	4A	AX-110449014	0.6303	117.3679

14	4A	AX-110391454	0.6303	117.3679
14	4A	AX-111061531	0.6303	117.3679
14	4A	AX-108866026	0.6303	117.3679
14	4A	AX-111526124	0.2066	117.5745
14	4A	AX-109294694	1.9406	119.5151
14	4A	AX-111522430	1.055	120.5701
14	4A	AX-109871319	1.055	120.5701
14	4A	AX-109061617	1.055	120.5701
14	4A	AX-110573633	1.055	120.5701
14	4A	AX-109924488	1.055	120.5701
14	4A	AX-110171938	1.055	120.5701
14	4A	AX-109884850	1.055	120.5701
14	4A	AX-110965230	1.055	120.5701
14	4A	AX-111508583	0.8334	121.4035
14	4A	AX-109926421	0.8334	121.4035
14	4A	AX-110504741	0.8334	121.4035
14	4A	AX-111207003	0.8334	121.4035
14	4A	AX-111025153	0.8334	121.4035
14	4A	AX-110389720	0.8334	121.4035
14	4A	AX-110675502	0.8334	121.4035
14	4A	AX-109956400	0.8334	121.4035
14	4A	AX-110932543	0.8334	121.4035
14	4A	AX-109386689	0.8334	121.4035
14	4A	AX-110585294	0.8334	121.4035
14	4A	AX-109049937	0.625	122.0286
14	4A	AX-110540586	0.625	122.0286
14	4A	AX-108908317	0.625	122.0286
14	4A	AX-109363749	0.625	122.0286
14	4A	AX-110577792	0.625	122.0286
14	4A	AX-111067369	0.2066	122.2352
14	4A	AX-111600193	0.2066	122.2352
14	4A	AX-109332913	17.7561	139.9912
14	4A	AX-109863601	0.4255	140.4168
14	4A	AX-109419476	20.2208	160.6376
14	4A	AX-108840096	0.4167	161.0542
14	4A	AX-109890636	0.4167	161.0542
14	4A	AX-111488174	0.4167	161.0542
14	4A	AX-111055135	0.2092	161.2635
14	4A	AX-109459874	0.422	161.6854
14	4A	AX-111162071	0.422	161.6854
14	4A	AX-109290964	0.2083	161.8937
14	4A	AX-109926385	6.4643	168.3581
14	4A	AX-110508187	2.4142	170.7722
14	4A	AX-108728790	2.4142	170.7722

14	4A	AX-111074992	2.4142	170.7722
14	4A	AX-111474672	2.4142	170.7722
14	4A	AX-111015766	2.4142	170.7722
14	4A	AX-110925416	0.8512	171.6234
14	4A	AX-110936251	0.8512	171.6234
14	4A	AX-108728455	0.8512	171.6234
14	4A	AX-111049826	0.8512	171.6234
14	4A	AX-109921457	0.8512	171.6234
14	4A	AX-110036632	0.8512	171.6234
14	4A	AX-110736225	0.8512	171.6234
14	4A	AX-110390510	0.8512	171.6234
14	4A	AX-109385065	0.8512	171.6234
14	4A	AX-111140519	0.8512	171.6234
14	4A	AX-108950742	0.8512	171.6234
14	4A	AX-111464038	0.8512	171.6234
14	4A	AX-108932708	0.8512	171.6234
14	4A	AX-109965096	0.8512	171.6234
14	4A	AX-109940327	0.8512	171.6234
14	4A	AX-109990978	0.8512	171.6234
14	4A	AX-109545247	0.8512	171.6234
14	4A	AX-111576665	0.8512	171.6234
14	4A	AX-111544737	0.8512	171.6234
14	4A	AX-110969201	0.8512	171.6234
14	4A	AX-89434097	0.8512	171.6234
14	4A	AX-110009502	0.8512	171.6234
15	4B	AX-111049420	0	0
15	4B	AX-108750428	0.211	0.211
15	4B	AX-95148627	0.211	0.211
15	4B	AX-110008956	2.1753	2.3862
15	4B	AX-110960528	10.0835	12.4697
15	4B	AX-110494559	0.4292	12.8989
15	4B	AX-110984553	0.4237	13.3227
15	4B	AX-111600938	0.4237	13.3227
15	4B	AX-111584191	0.4237	13.3227
15	4B	AX-108760910	0.4237	13.3227
15	4B	AX-111681231	0.4237	13.3227
15	4B	AX-111510337	0.4237	13.3227
15	4B	AX-109954164	0.4237	13.3227
15	4B	AX-108852650	0.4237	13.3227
15	4B	AX-111472806	0.8548	14.1774
15	4B	AX-110425375	0.8548	14.1774
15	4B	AX-109913880	0.8548	14.1774
15	4B	AX-109991473	0.8548	14.1774
15	4B	AX-111572615	0.8548	14.1774

15	4B	AX-110576785	0.422	14.5994
15	4B	AX-89469514	0.2101	14.8095
15	4B	AX-111481149	0.2083	15.0178
15	4B	AX-110383669	0.4202	15.438
15	4B	AX-109327593	0.4202	15.438
15	4B	AX-110076607	1.5026	16.9406
15	4B	AX-110161827	0.6276	17.5682
15	4B	AX-109323724	0.6276	17.5682
15	4B	AX-109348034	3.802	21.3702
15	4B	AX-110935897	3.802	21.3702
15	4B	AX-109074448	3.802	21.3702
15	4B	AX-108851428	3.802	21.3702
15	4B	AX-109986770	1.0595	22.4297
15	4B	AX-109901438	1.0595	22.4297
15	4B	AX-111640796	1.0595	22.4297
15	4B	AX-110675382	0.2092	22.6389
15	4B	AX-95684402	0.2092	22.6389
15	4B	AX-109867593	0.2092	22.6389
15	4B	AX-89414036	0.2092	22.6389
15	4B	AX-110548194	0.2092	22.6389
15	4B	AX-108746263	0.4255	23.0644
15	4B	AX-111694282	0.4237	23.4882
15	4B	AX-109401802	0.4237	23.4882
15	4B	AX-109901470	0.4237	23.4882
15	4B	AX-111107314	0.4237	23.4882
15	4B	AX-110542052	0.211	23.6991
15	4B	AX-111479123	0.211	23.6991
15	4B	AX-110473522	0.211	23.6991
15	4B	AX-111620933	0.211	23.6991
15	4B	AX-108750128	0.211	23.6991
15	4B	AX-109898875	0.211	23.6991
15	4B	AX-108819906	0.211	23.6991
15	4B	AX-109391268	0.211	23.6991
15	4B	AX-110582336	0.211	23.6991
15	4B	AX-109876993	0.211	23.6991
15	4B	AX-110427153	0.211	23.6991
15	4B	AX-111075819	0.211	23.6991
15	4B	AX-110561084	0.211	23.6991
15	4B	AX-110963107	5.6847	29.3839
15	4B	AX-111162353	5.6847	29.3839
15	4B	AX-110448600	5.6847	29.3839
15	4B	AX-108957851	5.6847	29.3839
15	4B	AX-111074077	5.6847	29.3839
15	4B	AX-111118705	0.2146	29.5985

15	4B	AX-110477582	4.1959	33.7943
15	4B	AX-108943201	4.1959	33.7943
15	4B	AX-110976982	4.1959	33.7943
15	4B	AX-108820969	4.1959	33.7943
15	4B	AX-109966618	4.1959	33.7943
15	4B	AX-109503821	4.1959	33.7943
15	4B	AX-111497396	3.1573	36.9517
15	4B	AX-108793833	3.1573	36.9517
15	4B	AX-109357821	3.1573	36.9517
15	4B	AX-111642482	3.1573	36.9517
15	4B	AX-89708030	7.1209	44.0726
15	4B	AX-111074167	1.2934	45.366
15	4B	AX-111517964	1.2934	45.366
15	4B	AX-89716448	1.7248	47.0908
15	4B	AX-110623354	0.4274	47.5182
15	4B	AX-110361956	0.4255	47.9437
15	4B	AX-110646091	0.6303	48.574
15	4B	AX-89362996	0.6303	48.574
15	4B	AX-109332533	0.6303	49.2043
15	4B	AX-108867943	0.6303	49.2043
15	4B	AX-111642793	0.6303	49.2043
15	4B	AX-111693091	0.6303	49.2043
15	4B	AX-109425122	0.6303	49.2043
15	4B	AX-108822594	0.6303	49.2043
15	4B	AX-109626810	0.6303	49.2043
15	4B	AX-108891179	0.6303	49.2043
15	4B	AX-89595080	0.6303	49.2043
15	4B	AX-110492111	0.6303	49.2043
15	4B	AX-110017436	0.6303	49.2043
15	4B	AX-109482022	0.6303	49.2043
15	4B	AX-109411735	0.6303	49.2043
15	4B	AX-109915480	0.6303	49.2043
15	4B	AX-109399994	0.6303	49.2043
15	4B	AX-110674260	0.6303	49.2043
15	4B	AX-111548935	0.6303	49.2043
15	4B	AX-111118883	0.6303	49.2043
15	4B	AX-111006270	0.6303	49.2043
15	4B	AX-111521785	0.6303	49.2043
15	4B	AX-111456523	0.6303	49.2043
15	4B	AX-109942088	0.6303	49.2043
15	4B	AX-108795059	0.6303	49.2043
15	4B	AX-109932578	0.6303	49.2043
15	4B	AX-111764167	0.6303	49.2043
15	4B	AX-111031196	0.6303	49.2043

15	4B	AX-110122702	0.6303	49.2043
15	4B	AX-110545516	0.6303	49.2043
15	4B	AX-109492046	0.6303	49.2043
15	4B	AX-111234988	0.6303	49.2043
15	4B	AX-109273896	0.6303	49.2043
15	4B	AX-110539311	0.6303	49.2043
15	4B	AX-110520184	0.6303	49.2043
15	4B	AX-111031234	0.6303	49.2043
15	4B	AX-108851370	0.6303	49.2043
15	4B	AX-109581787	0.6303	49.2043
15	4B	AX-111632313	0.6303	49.2043
15	4B	AX-110044007	0.6303	49.2043
15	4B	AX-109460416	0.6303	49.2043
15	4B	AX-111031248	0.6303	49.2043
15	4B	AX-111002957	0.6303	49.2043
15	4B	AX-109417462	0.6303	49.2043
15	4B	AX-111095898	0.6303	49.2043
15	4B	AX-110919561	0.6303	49.2043
15	4B	AX-108975695	0.6303	49.2043
15	4B	AX-109439294	0.6303	49.2043
15	4B	AX-110146279	0.6303	49.2043
15	4B	AX-111156970	0.6303	49.2043
15	4B	AX-111520885	0.6303	49.2043
15	4B	AX-110552600	0.6303	49.2043
15	4B	AX-109052518	0.6303	49.2043
15	4B	AX-108770960	0.6303	49.2043
15	4B	AX-109399857	0.6303	49.2043
15	4B	AX-110952722	0.6303	49.2043
15	4B	AX-109839862	0.6303	49.2043
15	4B	AX-108934061	0.6303	49.2043
15	4B	AX-111065695	0.6303	49.2043
15	4B	AX-108779986	0.6303	49.2043
15	4B	AX-111542971	0.6303	49.2043
15	4B	AX-111079195	0.6303	49.2043
15	4B	AX-110032256	0.6303	49.2043
15	4B	AX-109954310	0.6303	49.2043
15	4B	AX-109383400	0.6303	49.2043
15	4B	AX-110054702	0.6303	49.2043
15	4B	AX-109521781	0.6303	49.2043
15	4B	AX-109971627	0.6303	49.2043
15	4B	AX-111781809	0.6303	49.2043
15	4B	AX-110538202	0.6303	49.2043
15	4B	AX-109869391	0.6303	49.2043
15	4B	AX-108776557	0.6303	49.2043

15	4B	AX-110598981	0.6303	49.2043
15	4B	AX-110556966	0.6303	49.2043
15	4B	AX-109324089	0.6303	49.2043
15	4B	AX-110670562	0.6303	49.2043
15	4B	AX-110067455	0.6303	49.2043
15	4B	AX-109583279	0.6303	49.2043
15	4B	AX-108859072	0.6303	49.2043
15	4B	AX-109387541	0.6303	49.2043
15	4B	AX-109423611	0.6303	49.2043
15	4B	AX-109878255	0.6303	49.2043
15	4B	AX-110508288	0.6303	49.2043
15	4B	AX-110552774	0.6303	49.2043
15	4B	AX-110126630	0.6303	49.2043
15	4B	AX-111058231	0.6303	49.2043
15	4B	AX-111580212	0.6303	49.2043
15	4B	AX-109504036	0.6303	49.2043
15	4B	AX-109039878	0.6303	49.2043
15	4B	AX-110448750	0.6303	49.2043
15	4B	AX-110983098	0.6303	49.2043
15	4B	AX-108913050	0.6303	49.2043
15	4B	AX-109442056	0.6303	49.2043
15	4B	AX-109560724	0.6303	49.2043
15	4B	AX-110066286	0.6303	49.2043
15	4B	AX-109325792	0.6303	49.2043
15	4B	AX-110566634	0.6303	49.2043
15	4B	AX-111627904	0.6303	49.2043
15	4B	AX-109989278	0.6303	49.2043
15	4B	AX-109893091	0.6303	49.2043
15	4B	AX-109038212	0.6303	49.2043
15	4B	AX-109482097	0.6303	49.2043
15	4B	AX-109455743	0.6303	49.2043
15	4B	AX-109489399	0.6303	49.2043
15	4B	AX-111014573	0.6303	49.2043
15	4B	AX-109337036	0.6303	49.2043
15	4B	AX-110573292	0.6303	49.2043
15	4B	AX-109053550	0.6303	49.2043
15	4B	AX-109984793	0.6303	49.2043
15	4B	AX-110447146	0.6303	49.2043
15	4B	AX-111729891	0.6303	49.2043
15	4B	AX-109371297	0.6303	49.2043
15	4B	AX-111096718	0.6303	49.2043
15	4B	AX-110965684	0.6303	49.2043
15	4B	AX-110598173	0.6303	49.2043
15	4B	AX-111648845	0.6303	49.2043

15	4B	AX-111003696	0.6303	49.2043
15	4B	AX-110578412	0.6303	49.2043
15	4B	AX-109461512	0.6303	49.2043
15	4B	AX-109969027	0.6303	49.2043
15	4B	AX-108843012	0.6303	49.2043
15	4B	AX-109456115	0.6303	49.2043
15	4B	AX-109509473	0.6303	49.2043
15	4B	AX-108894315	0.6303	49.2043
15	4B	AX-111030221	0.6303	49.2043
15	4B	AX-108733620	0.6303	49.2043
15	4B	AX-110409166	0.6303	49.2043
15	4B	AX-109031246	0.6303	49.2043
15	4B	AX-111591855	0.6303	49.2043
15	4B	AX-108885338	0.6303	49.2043
15	4B	AX-111486436	0.6303	49.2043
15	4B	AX-110489625	0.6303	49.2043
15	4B	AX-109602320	0.6303	49.2043
15	4B	AX-110053953	0.6303	49.2043
15	4B	AX-110435837	0.6303	49.2043
15	4B	AX-110041102	0.6303	49.2043
15	4B	AX-110399870	0.6303	49.2043
15	4B	AX-109888756	0.6303	49.2043
15	4B	AX-110975736	0.6303	49.2043
15	4B	AX-109327751	0.6303	49.2043
15	4B	AX-109348064	0.6303	49.2043
15	4B	AX-111680631	0.6303	49.2043
15	4B	AX-111114401	0.6303	49.2043
15	4B	AX-108851021	0.6303	49.2043
15	4B	AX-109857515	0.6303	49.2043
15	4B	AX-108909057	0.6303	49.2043
15	4B	AX-108789337	0.6303	49.2043
15	4B	AX-108872710	0.6303	49.2043
15	4B	AX-110424427	0.6303	49.2043
15	4B	AX-109534445	0.6303	49.2043
15	4B	AX-110688360	0.6303	49.2043
15	4B	AX-108907582	0.6303	49.2043
15	4B	AX-111046935	0.6303	49.2043
15	4B	AX-110530150	0.6303	49.2043
15	4B	AX-111510412	0.6303	49.2043
15	4B	AX-111583137	0.6303	49.2043
15	4B	AX-111619190	0.6303	49.2043
15	4B	AX-109296057	0.6303	49.2043
15	4B	AX-110607900	0.6303	49.2043
15	4B	AX-108976944	0.6303	49.2043

15	4B	AX-110407111	0.6303	49.2043
15	4B	AX-110975012	0.6303	49.2043
15	4B	AX-111510948	0.6303	49.2043
15	4B	AX-110548442	0.6303	49.2043
15	4B	AX-109035295	0.6303	49.2043
15	4B	AX-108918930	0.6303	49.2043
15	4B	AX-108987253	0.6303	49.2043
15	4B	AX-110913839	0.6303	49.2043
15	4B	AX-111141653	0.6303	49.2043
15	4B	AX-108766235	0.6303	49.2043
15	4B	AX-109936813	0.6303	49.2043
15	4B	AX-110032284	0.6303	49.2043
15	4B	AX-111760248	0.6303	49.2043
15	4B	AX-108926622	0.6303	49.2043
15	4B	AX-108977377	0.6303	49.2043
15	4B	AX-111680609	0.6303	49.2043
15	4B	AX-109374467	0.6303	49.2043
15	4B	AX-108904426	0.6303	49.2043
15	4B	AX-111093865	0.6303	49.2043
15	4B	AX-109580702	0.6303	49.2043
15	4B	AX-111654768	0.6303	49.2043
15	4B	AX-111077863	0.6303	49.2043
15	4B	AX-110393452	0.6303	49.2043
15	4B	AX-108843011	0.6303	49.2043
15	4B	AX-111013455	0.6303	49.2043
15	4B	AX-111449697	0.6303	49.2043
15	4B	AX-110374612	0.6303	49.2043
15	4B	AX-108942655	0.6303	49.2043
15	4B	AX-111543042	0.6303	49.2043
15	4B	AX-109400136	0.6303	49.2043
15	4B	AX-110141565	0.6303	49.2043
15	4B	AX-109434114	0.6303	49.2043
15	4B	AX-111566580	0.6303	49.2043
15	4B	AX-111760569	0.6303	49.2043
15	4B	AX-108952461	0.6303	49.2043
15	4B	AX-111156928	0.6303	49.2043
15	4B	AX-111529675	0.6303	49.2043
15	4B	AX-110536290	0.6303	49.2043
15	4B	AX-110945288	0.6303	49.2043
15	4B	AX-110565938	0.6303	49.2043
15	4B	AX-111099644	0.6303	49.2043
15	4B	AX-110443031	0.6303	49.2043
15	4B	AX-109885871	0.6303	49.2043
15	4B	AX-110058582	0.6303	49.2043

15	4B	AX-111579188	0.6303	49.2043
15	4B	AX-109927694	0.6303	49.2043
15	4B	AX-110398739	0.6303	49.2043
15	4B	AX-111510896	0.6303	49.2043
15	4B	AX-111672289	0.6303	49.2043
15	4B	AX-111094270	0.6303	49.2043
15	4B	AX-110124108	0.6303	49.2043
15	4B	AX-110145260	0.6303	49.2043
15	4B	AX-111061339	0.6303	49.2043
15	4B	AX-110953230	0.6303	49.2043
15	4B	AX-109034996	0.6303	49.2043
15	4B	AX-109277201	0.6303	49.2043
15	4B	AX-111136098	0.6303	49.2043
15	4B	AX-109305419	0.6303	49.2043
15	4B	AX-109622086	0.6303	49.2043
15	4B	AX-109844893	0.6303	49.2043
15	4B	AX-111108797	0.6303	49.2043
15	4B	AX-109360848	0.6303	49.2043
15	4B	AX-111776570	0.6303	49.2043
15	4B	AX-111780488	0.6303	49.2043
15	4B	AX-109479017	0.6303	49.2043
15	4B	AX-110958679	0.6303	49.2043
15	4B	AX-111675088	0.6303	49.2043
15	4B	AX-110030973	0.6303	49.2043
15	4B	AX-110429345	0.6303	49.2043
15	4B	AX-109326975	0.6303	49.2043
15	4B	AX-111623502	0.6303	49.2043
15	4B	AX-108770803	0.6303	49.2043
15	4B	AX-109341791	0.6303	49.2043
15	4B	AX-110037851	0.6303	49.2043
15	4B	AX-109007158	0.6303	49.2043
15	4B	AX-111732875	0.6303	49.2043
15	4B	AX-110467722	0.6303	49.2043
15	4B	AX-110125775	0.6303	49.2043
15	4B	AX-108830847	0.6303	49.2043
15	4B	AX-111004994	0.6303	49.2043
15	4B	AX-110978688	0.6303	49.2043
15	4B	AX-110918826	0.6303	49.2043
15	4B	AX-109968923	0.6303	49.2043
15	4B	AX-108988131	0.6303	49.2043
15	4B	AX-109435753	0.6303	49.2043
15	4B	AX-110388166	0.6303	49.2043
15	4B	AX-108771521	0.6303	49.2043
15	4B	AX-109843718	0.6303	49.2043

15	4B	AX-110080710	0.6303	49.2043
15	4B	AX-111536549	0.6303	49.2043
15	4B	AX-111170877	0.6303	49.2043
15	4B	AX-111212427	0.6303	49.2043
15	4B	AX-111166447	0.6303	49.2043
15	4B	AX-109578138	0.6303	49.2043
15	4B	AX-108955158	0.6303	49.2043
15	4B	AX-111014886	0.6303	49.2043
15	4B	AX-110957362	0.6303	49.2043
15	4B	AX-108801153	0.6303	49.2043
15	4B	AX-111234110	0.6303	49.2043
15	4B	AX-109898569	0.6303	49.2043
15	4B	AX-111172685	0.6303	49.2043
15	4B	AX-111567546	0.6303	49.2043
15	4B	AX-109298161	0.6303	49.2043
15	4B	AX-109972799	0.6303	49.2043
15	4B	AX-111022364	0.6303	49.2043
15	4B	AX-111644194	0.6303	49.2043
15	4B	AX-110472613	0.6303	49.2043
15	4B	AX-111777959	0.6303	49.2043
15	4B	AX-94399941	0.6303	49.2043
15	4B	AX-111104382	0.6303	49.2043
15	4B	AX-111009056	0.6303	49.2043
15	4B	AX-109885115	0.6303	49.2043
15	4B	AX-110419388	0.6303	49.2043
15	4B	AX-110712620	0.6303	49.2043
15	4B	AX-110389078	0.6303	49.2043
15	4B	AX-108983935	0.6303	49.2043
15	4B	AX-110381216	0.6303	49.2043
15	4B	AX-110511397	0.6303	49.2043
15	4B	AX-110625547	0.6303	49.2043
15	4B	AX-111129840	0.6303	49.2043
15	4B	AX-111483086	0.6303	49.2043
15	4B	AX-111780236	0.6303	49.2043
15	4B	AX-108879881	0.6303	49.2043
15	4B	AX-108899559	0.6303	49.2043
15	4B	AX-109839900	0.6303	49.2043
15	4B	AX-110946879	0.6303	49.2043
15	4B	AX-110962042	0.6303	49.2043
15	4B	AX-111047274	0.6303	49.2043
15	4B	AX-111567365	0.6303	49.2043
15	4B	AX-109519900	0.6303	49.2043
15	4B	AX-111589692	0.6303	49.2043
15	4B	AX-94613145	0.6303	49.2043

15	4B	AX-110497642	0.422	49.6263
15	4B	AX-109915467	0.431	50.0573
15	4B	AX-109924475	0.2137	50.271
15	4B	AX-109015565	0.2101	50.4811
15	4B	AX-109431795	0.4184	50.8995
15	4B	AX-111180671	0.4184	50.8995
15	4B	AX-109477970	0.2083	51.1078
15	4B	AX-109477553	0.2083	51.1078
15	4B	AX-110390949	0.2083	51.1078
15	4B	AX-110125717	0.2083	51.1078
15	4B	AX-111051657	0.2083	51.1078
15	4B	AX-110615646	0.2083	51.1078
15	4B	AX-109956822	1.2769	52.3847
15	4B	AX-111572501	1.2769	52.3847
15	4B	AX-111610520	1.2769	52.3847
15	4B	AX-110388384	1.2769	52.3847
15	4B	AX-94510415	1.2769	52.3847
15	4B	AX-111724067	1.2769	52.3847
15	4B	AX-110917271	1.2769	52.3847
15	4B	AX-109934724	1.2769	52.3847
15	4B	AX-108810416	1.2769	52.3847
15	4B	AX-111596153	1.2769	52.3847
15	4B	AX-111689298	1.2769	52.3847
15	4B	AX-109273501	1.2769	52.3847
15	4B	AX-110440313	1.2769	52.3847
15	4B	AX-110126457	1.2769	52.3847
15	4B	AX-109005253	1.2769	52.3847
15	4B	AX-111014468	1.2769	52.3847
15	4B	AX-110062074	1.2769	52.3847
15	4B	AX-110089284	1.2769	52.3847
15	4B	AX-110129554	1.2769	52.3847
15	4B	AX-108774257	1.2769	52.3847
15	4B	AX-111669008	1.2769	52.3847
15	4B	AX-111251314	1.2769	52.3847
15	4B	AX-111216202	1.2769	52.3847
15	4B	AX-110965179	1.2769	52.3847
15	4B	AX-109313093	1.2769	52.3847
15	4B	AX-110570384	1.2769	52.3847
15	4B	AX-110050889	1.2769	52.3847
15	4B	AX-111235683	1.2769	52.3847
15	4B	AX-109843364	1.2769	52.3847
15	4B	AX-111763569	1.2769	52.3847
15	4B	AX-108920897	1.2769	52.3847
15	4B	AX-111098806	1.2769	52.3847

15	4B	AX-111547843	1.2769	52.3847
15	4B	AX-108756240	1.2769	52.3847
15	4B	AX-111574562	1.2769	52.3847
15	4B	AX-111026902	1.2769	52.3847
15	4B	AX-111562232	1.2769	52.3847
15	4B	AX-109444920	1.2769	52.3847
15	4B	AX-109352658	1.2769	52.3847
15	4B	AX-108876485	1.2769	52.3847
15	4B	AX-111058252	1.2769	52.3847
15	4B	AX-111763540	1.2769	52.3847
15	4B	AX-108810932	1.2769	52.3847
15	4B	AX-110396451	1.2769	52.3847
15	4B	AX-109319795	1.2769	52.3847
15	4B	AX-111717124	1.2769	52.3847
15	4B	AX-109983571	1.2769	52.3847
15	4B	AX-111576752	1.2769	52.3847
15	4B	AX-109270209	1.2769	52.3847
15	4B	AX-110395087	1.2769	52.3847
15	4B	AX-111458629	1.2769	52.3847
15	4B	AX-109410709	1.2769	52.3847
15	4B	AX-111605772	1.2769	52.3847
15	4B	AX-110940745	1.2769	52.3847
15	4B	AX-109959574	1.2769	52.3847
15	4B	AX-110604204	1.2769	52.3847
15	4B	AX-108932466	1.2769	52.3847
15	4B	AX-108872294	1.2769	52.3847
15	4B	AX-108963565	1.2769	52.3847
15	4B	AX-111014334	1.2769	52.3847
15	4B	AX-109615458	1.2769	52.3847
15	4B	AX-109911461	1.2769	52.3847
15	4B	AX-109868752	1.2769	52.3847
15	4B	AX-111578160	1.2769	52.3847
15	4B	AX-111547711	1.2769	52.3847
15	4B	AX-111756841	1.2769	52.3847
15	4B	AX-95242684	1.2769	52.3847
15	4B	AX-109270926	1.2769	52.3847
15	4B	AX-111102919	1.2769	52.3847
15	4B	AX-109864576	1.2769	52.3847
15	4B	AX-111070464	1.2769	52.3847
15	4B	AX-110483607	1.2769	52.3847
15	4B	AX-109600644	1.2769	52.3847
15	4B	AX-111653076	1.2769	52.3847
15	4B	AX-111087076	1.2769	52.3847
15	4B	AX-108938270	1.2769	52.3847

15	4B	AX-110539042	1.2769	52.3847
15	4B	AX-110560467	1.2769	52.3847
15	4B	AX-111494454	1.2769	52.3847
15	4B	AX-109516755	1.2769	52.3847
15	4B	AX-111529469	1.2769	52.3847
15	4B	AX-109463053	1.2769	52.3847
15	4B	AX-111031306	1.2769	52.3847
15	4B	AX-110104281	1.2769	52.3847
15	4B	AX-109941318	1.2769	52.3847
15	4B	AX-109392725	1.2769	52.3847
15	4B	AX-111241403	1.2769	52.3847
15	4B	AX-109516816	1.2769	52.3847
15	4B	AX-110146053	1.2769	52.3847
15	4B	AX-111091451	1.2769	52.3847
15	4B	AX-109278517	1.2769	52.3847
15	4B	AX-111043077	1.2769	52.3847
15	4B	AX-110525016	1.2769	52.3847
15	4B	AX-109833338	1.2769	52.3847
15	4B	AX-109348033	1.2769	52.3847
15	4B	AX-109054774	1.2769	52.3847
15	4B	AX-111025767	1.2769	52.3847
15	4B	AX-111039690	1.2769	52.3847
15	4B	AX-109425196	1.2769	52.3847
15	4B	AX-109945364	1.2769	52.3847
15	4B	AX-109454212	1.2769	52.3847
15	4B	AX-111522580	1.2769	52.3847
15	4B	AX-111051638	1.2769	52.3847
15	4B	AX-111462658	1.2769	52.3847
15	4B	AX-111218553	1.2769	52.3847
15	4B	AX-111740846	1.2769	52.3847
15	4B	AX-110366905	1.2769	52.3847
15	4B	AX-108819049	1.2769	52.3847
15	4B	AX-108817888	1.2769	52.3847
15	4B	AX-110512536	1.2769	52.3847
15	4B	AX-109976390	1.2769	52.3847
15	4B	AX-108876645	1.2769	52.3847
15	4B	AX-110502624	1.2769	52.3847
15	4B	AX-109844452	1.2769	52.3847
15	4B	AX-111028236	1.2769	52.3847
15	4B	AX-110122470	1.2769	52.3847
15	4B	AX-111078484	1.2769	52.3847
15	4B	AX-95684605	1.2769	52.3847
15	4B	AX-111776095	1.2769	52.3847
15	4B	AX-111004865	1.2769	52.3847

15	4B	AX-111144552	1.2769	52.3847
15	4B	AX-111627646	1.2769	52.3847
15	4B	AX-110489179	1.2769	52.3847
15	4B	AX-111700309	1.2769	52.3847
15	4B	AX-111558122	1.2769	52.3847
15	4B	AX-111627676	1.2769	52.3847
15	4B	AX-110538149	1.2769	52.3847
15	4B	AX-109554416	1.2769	52.3847
15	4B	AX-109414168	1.2769	52.3847
15	4B	AX-109011850	1.2769	52.3847
15	4B	AX-108773581	1.2769	52.3847
15	4B	AX-110522717	1.2769	52.3847
15	4B	AX-111477972	1.2769	52.3847
15	4B	AX-109496466	1.2769	52.3847
15	4B	AX-109031084	1.2769	52.3847
15	4B	AX-111518553	1.2769	52.3847
15	4B	AX-110979324	1.2769	52.3847
15	4B	AX-111785675	1.2769	52.3847
15	4B	AX-110020293	1.2769	52.3847
15	4B	AX-110520007	1.2769	52.3847
15	4B	AX-110391798	1.2769	52.3847
15	4B	AX-108900130	1.2769	52.3847
15	4B	AX-110919776	1.2769	52.3847
15	4B	AX-110126904	1.2769	52.3847
15	4B	AX-110011440	1.2769	52.3847
15	4B	AX-111517376	1.2769	52.3847
15	4B	AX-110473293	1.2769	52.3847
15	4B	AX-110975765	1.2769	52.3847
15	4B	AX-110969708	1.2769	52.3847
15	4B	AX-110667870	1.2769	52.3847
15	4B	AX-109961975	1.2769	52.3847
15	4B	AX-111160460	1.2769	52.3847
15	4B	AX-109843733	1.2769	52.3847
15	4B	AX-111131801	1.2769	52.3847
15	4B	AX-111474716	1.2769	52.3847
15	4B	AX-111538433	1.2769	52.3847
15	4B	AX-110960570	1.2769	52.3847
15	4B	AX-111660331	1.2769	52.3847
15	4B	AX-110904766	1.2769	52.3847
15	4B	AX-109870656	1.2769	52.3847
15	4B	AX-111505357	1.2769	52.3847
15	4B	AX-109027703	1.2769	52.3847
15	4B	AX-111064153	1.2769	52.3847
15	4B	AX-110506412	1.2769	52.3847

15	4B	AX-109883287	1.2769	52.3847
15	4B	AX-109335683	1.2769	52.3847
15	4B	AX-109919972	1.2769	52.3847
15	4B	AX-110396773	1.2769	52.3847
15	4B	AX-110952706	1.2769	52.3847
15	4B	AX-110537775	1.2769	52.3847
15	4B	AX-109394806	1.2769	52.3847
15	4B	AX-109550772	1.2769	52.3847
15	4B	AX-111494884	1.2769	52.3847
15	4B	AX-111608979	1.2769	52.3847
15	4B	AX-109313216	1.2769	52.3847
15	4B	AX-111719562	1.2769	52.3847
15	4B	AX-109426290	1.2769	52.3847
15	4B	AX-109493306	1.2769	52.3847
15	4B	AX-110621216	1.2769	52.3847
15	4B	AX-108838785	1.2769	52.3847
15	4B	AX-109948570	1.2769	52.3847
15	4B	AX-111780380	1.2769	52.3847
15	4B	AX-108818165	1.2769	52.3847
15	4B	AX-110651102	1.2769	52.3847
15	4B	AX-111474390	1.2769	52.3847
15	4B	AX-108920279	1.2769	52.3847
15	4B	AX-110450356	1.2769	52.3847
15	4B	AX-111526492	1.2769	52.3847
15	4B	AX-109931786	0.6329	53.0176
15	4B	AX-111623649	0.6329	53.0176
15	4B	AX-109405572	0.6329	53.0176
15	4B	AX-109866370	0.6329	53.0176
15	4B	AX-109296842	0.6329	53.0176
15	4B	AX-111631871	0.6329	53.0176
15	4B	AX-111042954	0.6329	53.0176
15	4B	AX-108972236	0.6329	53.0176
15	4B	AX-108974756	0.6329	53.0176
15	4B	AX-111614541	0.6329	53.0176
15	4B	AX-111507276	0.6329	53.0176
15	4B	AX-111474354	0.6329	53.0176
15	4B	AX-109310564	0.6329	53.0176
15	4B	AX-110385868	0.6329	53.0176
15	4B	AX-111127678	0.6329	53.0176
15	4B	AX-108867036	0.6329	53.0176
15	4B	AX-108952454	0.6329	53.0176
15	4B	AX-108781802	0.6329	53.0176
15	4B	AX-109836405	0.6329	53.0176
15	4B	AX-109496762	0.6329	53.0176

15	4B	AX-110486518	0.6329	53.0176
15	4B	AX-109341809	0.6329	53.0176
15	4B	AX-110954179	0.6329	53.0176
15	4B	AX-110744838	0.6329	53.0176
15	4B	AX-110452596	0.6329	53.0176
15	4B	AX-110118973	0.6329	53.0176
15	4B	AX-109334541	0.6329	53.0176
15	4B	AX-108993444	0.6329	53.0176
15	4B	AX-110019365	0.6329	53.0176
15	4B	AX-109391822	0.6329	53.0176
15	4B	AX-110059285	0.6329	53.0176
15	4B	AX-110985700	0.6329	53.0176
15	4B	AX-111114656	0.6329	53.0176
15	4B	AX-109970298	0.6329	53.0176
15	4B	AX-108886822	0.6329	53.0176
15	4B	AX-108935256	0.6329	53.0176
15	4B	AX-109996117	0.6329	53.0176
15	4B	AX-111451315	0.6329	53.0176
15	4B	AX-108955591	0.6329	53.0176
15	4B	AX-95684558	0.6329	53.0176
15	4B	AX-110636079	0.6329	53.0176
15	4B	AX-109878998	0.4202	53.4378
15	4B	AX-108778637	0.4202	53.4378
15	4B	AX-110560008	0.4202	53.4378
15	4B	AX-111137716	0.4202	53.4378
15	4B	AX-111091687	0.4202	53.4378
15	4B	AX-108850496	0.4202	53.4378
15	4B	AX-110959504	0.4202	53.4378
15	4B	AX-109520695	0.4202	53.4378
15	4B	AX-110465483	0.4202	53.4378
15	4B	AX-111014171	0.4202	53.4378
15	4B	AX-110158288	0.4202	53.4378
15	4B	AX-110057810	0.4202	53.4378
15	4B	AX-108801401	0.4202	53.4378
15	4B	AX-109409008	0.4202	53.4378
15	4B	AX-110447227	0.4202	53.4378
15	4B	AX-109400188	0.4202	53.4378
15	4B	AX-108765024	0.4202	53.4378
15	4B	AX-111068940	0.4202	53.4378
15	4B	AX-110525046	0.4202	53.4378
15	4B	AX-110522976	0.4202	53.4378
15	4B	AX-108846403	0.4202	53.4378
15	4B	AX-111026095	0.4202	53.4378
15	4B	AX-109077371	0.4202	53.4378

15	4B	AX-108727648	0.4202	53.4378
15	4B	AX-111135027	0.4202	53.4378
15	4B	AX-109367178	0.4202	53.4378
15	4B	AX-111123505	0.4202	53.4378
15	4B	AX-111172501	0.4202	53.4378
15	4B	AX-111612712	0.4202	53.4378
15	4B	AX-109460233	0.4202	53.858
15	4B	AX-108822622	0.211	54.069
15	4B	AX-110430740	0.211	54.069
15	4B	AX-109401270	0.211	54.069
15	4B	AX-108765412	0.211	54.069
15	4B	AX-109419005	0.211	54.069
15	4B	AX-111585818	0.211	54.069
15	4B	AX-109537396	0.4255	54.4945
15	4B	AX-108922248	0.4255	54.4945
15	4B	AX-109985045	0.4255	54.4945
15	4B	AX-109439249	0.4255	54.4945
15	4B	AX-111115843	0.4255	54.4945
15	4B	AX-110025957	0.4255	54.4945
15	4B	AX-111662892	0.4255	54.4945
15	4B	AX-110956540	0.4255	54.4945
15	4B	AX-109340409	0.4255	54.4945
15	4B	AX-111464523	0.4255	54.4945
15	4B	AX-111628982	0.4255	54.4945
15	4B	AX-109857342	0.4255	54.4945
15	4B	AX-109306679	0.4255	54.4945
15	4B	AX-111587832	0.4255	54.4945
15	4B	AX-110036939	0.4255	54.4945
15	4B	AX-109873109	0.4255	54.4945
15	4B	AX-109324752	0.4255	54.4945
15	4B	AX-111069515	0.4255	54.4945
15	4B	AX-109585756	0.4255	54.4945
15	4B	AX-110995806	0.4255	54.4945
15	4B	AX-108810685	0.4255	54.4945
15	4B	AX-111581304	0.4255	54.4945
15	4B	AX-111185984	0.4255	54.4945
15	4B	AX-111472648	0.4255	54.4945
15	4B	AX-109867071	0.4255	54.4945
15	4B	AX-110933484	0.4255	54.4945
15	4B	AX-109526320	0.4255	54.4945
15	4B	AX-110530367	0.4255	54.4945
15	4B	AX-111478022	0.4255	54.4945
15	4B	AX-110973841	0.4255	54.4945
15	4B	AX-108872973	0.4255	54.4945

15	4B	AX-109583469	0.4255	54.4945
15	4B	AX-109103825	0.4255	54.4945
15	4B	AX-109358334	0.4255	54.4945
15	4B	AX-110910808	0.4255	54.4945
15	4B	AX-110506612	0.4255	54.4945
15	4B	AX-109900966	0.4255	54.4945
15	4B	AX-110153527	0.4255	54.4945
15	4B	AX-109509067	0.4255	54.4945
15	4B	AX-109993754	0.4255	54.4945
15	4B	AX-111461404	0.4255	54.4945
15	4B	AX-111488369	0.4255	54.4945
15	4B	AX-111633621	0.4255	54.4945
15	4B	AX-111539361	0.4255	54.4945
15	4B	AX-110942625	0.4255	54.4945
15	4B	AX-110533351	0.4255	54.4945
15	4B	AX-109305229	0.4255	54.4945
15	4B	AX-111007900	0.4255	54.4945
15	4B	AX-110589591	0.4255	54.4945
15	4B	AX-111622452	0.4255	54.4945
15	4B	AX-110935762	0.4255	54.4945
15	4B	AX-109890177	0.4255	54.4945
15	4B	AX-108904200	0.4255	54.4945
15	4B	AX-110598973	0.4255	54.4945
15	4B	AX-110476859	0.4255	54.4945
15	4B	AX-95129444	0.4255	54.4945
15	4B	AX-95632257	0.4255	54.4945
15	4B	AX-109468541	0.2128	54.7073
15	4B	AX-110972316	0.2128	54.7073
15	4B	AX-108819885	0.2119	54.9191
15	4B	AX-111610113	0.6356	55.5548
15	4B	AX-109386770	0.6356	55.5548
15	4B	AX-110512522	0.6356	55.5548
15	4B	AX-109899495	0.6356	55.5548
15	4B	AX-110921268	0.6356	55.5548
15	4B	AX-111496693	0.6356	55.5548
15	4B	AX-111125194	0.6356	55.5548
15	4B	AX-108870015	0.6356	55.5548
15	4B	AX-111833370	0.6356	55.5548
15	4B	AX-108830530	0.6356	55.5548
15	4B	AX-109375216	0.6356	55.5548
15	4B	AX-108813019	0.6356	55.5548
15	4B	AX-111012648	0.6356	55.5548
15	4B	AX-110986444	0.6356	55.5548
15	4B	AX-111062159	0.6356	55.5548

15	4B	AX-108796873	0.6356	55.5548
15	4B	AX-111642783	0.6356	55.5548
15	4B	AX-111642243	0.6356	55.5548
15	4B	AX-109370042	0.6356	56.1904
15	4B	AX-109930068	0.6356	56.1904
15	4B	AX-110384011	0.6356	56.1904
15	4B	AX-109285672	0.6356	56.1904
15	4B	AX-94533044	0.6356	56.1904
15	4B	AX-111043532	0.6356	56.1904
15	4B	AX-109999593	0.6356	56.1904
15	4B	AX-111671995	0.6356	56.1904
15	4B	AX-110973424	0.6356	56.1904
15	4B	AX-110500412	0.6356	56.1904
15	4B	AX-110039373	0.6356	56.1904
15	4B	AX-110948730	0.6356	56.1904
15	4B	AX-109342729	0.6356	56.1904
15	4B	AX-111233436	0.6356	56.1904
15	4B	AX-109272358	0.6356	56.1904
15	4B	AX-109479856	0.6356	56.1904
15	4B	AX-111018149	0.6356	56.1904
15	4B	AX-111724366	0.6356	56.1904
15	4B	AX-111055753	0.6356	56.1904
15	4B	AX-110417576	0.6356	56.1904
15	4B	AX-109954892	0.6356	56.1904
15	4B	AX-111044642	0.6356	56.1904
15	4B	AX-110462886	0.6356	56.1904
15	4B	AX-108966529	0.6356	56.1904
15	4B	AX-110496718	0.6356	56.1904
15	4B	AX-109344607	0.6356	56.1904
15	4B	AX-109843402	0.6356	56.1904
15	4B	AX-110547809	0.6356	56.1904
15	4B	AX-108859876	0.6356	56.1904
15	4B	AX-111127861	0.6356	56.1904
15	4B	AX-111025971	0.6356	56.1904
15	4B	AX-110906507	0.6356	56.1904
15	4B	AX-110395062	0.6356	56.1904
15	4B	AX-111554612	0.6356	56.1904
15	4B	AX-108750065	0.6356	56.1904
15	4B	AX-109101993	0.6356	56.1904
15	4B	AX-110666528	0.6356	56.1904
15	4B	AX-111783659	0.6356	56.1904
15	4B	AX-109327739	0.6356	56.1904
15	4B	AX-109842310	0.6356	56.1904
15	4B	AX-111687476	0.6356	56.1904

15	4B	AX-111544032	0.6356	56.1904
15	4B	AX-110520157	0.6356	56.1904
15	4B	AX-110505116	0.6356	56.1904
15	4B	AX-110671584	0.6356	56.1904
15	4B	AX-111076835	0.6356	56.1904
15	4B	AX-110574019	0.6356	56.1904
15	4B	AX-110667705	0.6356	56.1904
15	4B	AX-109953958	0.6356	56.1904
15	4B	AX-109330857	0.6356	56.1904
15	4B	AX-111588547	0.6356	56.1904
15	4B	AX-110026879	0.6356	56.1904
15	4B	AX-94482226	0.6356	56.1904
15	4B	AX-109846329	0.422	56.6123
15	4B	AX-109473487	0.422	56.6123
15	4B	AX-111509085	0.422	56.6123
15	4B	AX-111134446	0.422	56.6123
15	4B	AX-111153083	0.422	56.6123
15	4B	AX-108756648	0.422	56.6123
15	4B	AX-109339175	0.422	56.6123
15	4B	AX-109391034	0.2137	56.826
15	4B	AX-109330683	0.4405	57.2665
15	4B	AX-111486674	0.6579	57.9245
15	4B	AX-110374212	0.6579	57.9245
15	4B	AX-111480322	0.6579	57.9245
15	4B	AX-108786930	0.6579	57.9245
15	4B	AX-109003686	0.6579	57.9245
15	4B	AX-109842834	0.6579	57.9245
15	4B	AX-110009782	0.6579	57.9245
15	4B	AX-108737791	0.6579	57.9245
15	4B	AX-109541334	0.6579	57.9245
15	4B	AX-108804704	0.6579	57.9245
15	4B	AX-109426366	0.6579	57.9245
15	4B	AX-109983775	0.6579	57.9245
15	4B	AX-111662588	0.6579	57.9245
15	4B	AX-111484246	0.6579	57.9245
15	4B	AX-110982928	0.6579	57.9245
15	4B	AX-110494236	0.6579	57.9245
15	4B	AX-110404018	1.7398	59.6643
15	4B	AX-111168889	1.7398	59.6643
15	4B	AX-108815576	1.7398	59.6643
15	4B	AX-111709513	1.7398	59.6643
15	4B	AX-110617743	1.7398	59.6643
15	4B	AX-109868090	1.7398	59.6643
15	4B	AX-111679434	1.7398	59.6643

15	4B	AX-109915466	1.7398	59.6643
15	4B	AX-111542372	1.7398	59.6643
15	4B	AX-110420707	1.7398	59.6643
15	4B	AX-111776628	1.7398	59.6643
15	4B	AX-110447221	1.7398	59.6643
15	4B	AX-111114232	1.7398	59.6643
15	4B	AX-109848720	1.7398	59.6643
15	4B	AX-108818834	1.7398	59.6643
15	4B	AX-111264992	1.7398	59.6643
15	4B	AX-108838620	1.7398	59.6643
15	4B	AX-109865844	1.7398	59.6643
15	4B	AX-111525837	1.7398	59.6643
15	4B	AX-109397120	1.7398	59.6643
15	4B	AX-110558461	1.7398	59.6643
15	4B	AX-109861624	1.7398	59.6643
15	4B	AX-108792295	1.7398	59.6643
15	4B	AX-110492211	1.7398	59.6643
15	4B	AX-111010159	1.7398	59.6643
15	4B	AX-109589183	1.7398	59.6643
15	4B	AX-111654983	1.7398	59.6643
15	4B	AX-111542943	1.7398	59.6643
15	4B	AX-108949865	1.7398	59.6643
15	4B	AX-95683924	1.7398	59.6643
15	4B	AX-111472597	0.6329	60.2973
15	4B	AX-108855305	0.6329	60.2973
15	4B	AX-111160024	0.6329	60.2973
15	4B	AX-110538921	0.6329	60.2973
15	4B	AX-108759846	0.6329	60.2973
15	4B	AX-89628493	0.6329	60.2973
15	4B	AX-110977154	0.6329	60.2973
15	4B	AX-110915067	0.6329	60.2973
15	4B	AX-109461383	0.6329	60.2973
15	4B	AX-109893021	0.6329	60.2973
15	4B	AX-111487673	0.6329	60.2973
15	4B	AX-110167445	0.6329	60.2973
15	4B	AX-109839454	0.6329	60.2973
15	4B	AX-110522681	0.6329	60.2973
15	4B	AX-109317128	0.6329	60.2973
15	4B	AX-109826428	0.6329	60.2973
15	4B	AX-109888584	0.6329	60.2973
15	4B	AX-109948933	0.6329	60.2973
15	4B	AX-111236223	0.6329	60.2973
15	4B	AX-111214789	0.6329	60.2973
15	4B	AX-108888210	0.6329	60.2973

15	4B	AX-110388342	0.6329	60.2973
15	4B	AX-111464552	0.6329	60.2973
15	4B	AX-111461846	0.6329	60.2973
15	4B	AX-108917762	0.6329	60.2973
15	4B	AX-111639722	0.6329	60.2973
15	4B	AX-111535446	0.6329	60.2973
15	4B	AX-109053883	0.6329	60.2973
15	4B	AX-111564806	0.6329	60.2973
15	4B	AX-109382842	0.6329	60.2973
15	4B	AX-109075711	0.6329	60.2973
15	4B	AX-108749081	0.6329	60.2973
15	4B	AX-110372057	0.6329	60.2973
15	4B	AX-109864144	0.6329	60.2973
15	4B	AX-110492518	0.6329	60.2973
15	4B	AX-108770645	0.6329	60.2973
15	4B	AX-110976035	0.6329	60.2973
15	4B	AX-109548189	0.6329	60.2973
15	4B	AX-111123332	0.6329	60.2973
15	4B	AX-111575023	0.6329	60.2973
15	4B	AX-109362282	0.6329	60.2973
15	4B	AX-110591267	0.6329	60.2973
15	4B	AX-110130632	0.6329	60.2973
15	4B	AX-109350990	0.6329	60.2973
15	4B	AX-111528005	0.6329	60.2973
15	4B	AX-111039449	0.6329	60.2973
15	4B	AX-110978194	0.6329	60.2973
15	4B	AX-86174001	0.6329	60.2973
15	4B	AX-111605906	0.6329	60.2973
15	4B	AX-111806894	0.6329	60.2973
15	4B	AX-108725467	0.6329	60.2973
15	4B	AX-109883743	0.6329	60.2973
15	4B	AX-111484501	0.6329	60.2973
15	4B	AX-109470797	0.6329	60.2973
15	4B	AX-111662410	0.6329	60.2973
15	4B	AX-109369802	0.6329	60.2973
15	4B	AX-111218721	0.6329	60.2973
15	4B	AX-111083594	0.6329	60.2973
15	4B	AX-110999410	0.6329	60.2973
15	4B	AX-110930571	0.6329	60.2973
15	4B	AX-109486127	0.6329	60.2973
15	4B	AX-110441986	0.6329	60.2973
15	4B	AX-111725524	0.6329	60.2973
15	4B	AX-110144838	0.6329	60.2973
15	4B	AX-111489900	0.6329	60.2973

15	4B	AX-111483821	0.6329	60.2973
15	4B	AX-111460556	0.6329	60.2973
15	4B	AX-110969696	0.6329	60.2973
15	4B	AX-110370867	0.6329	60.2973
15	4B	AX-109584489	0.6329	60.2973
15	4B	AX-109601378	0.6329	60.2973
15	4B	AX-108846181	0.6329	60.2973
15	4B	AX-110057700	0.6329	60.2973
15	4B	AX-109953286	0.6329	60.2973
15	4B	AX-109103456	0.6329	60.2973
15	4B	AX-110569304	0.6329	60.2973
15	4B	AX-111463679	0.6329	60.2973
15	4B	AX-111052385	0.6329	60.2973
15	4B	AX-111111551	0.6329	60.2973
15	4B	AX-111514214	0.6329	60.2973
15	4B	AX-110375051	0.6329	60.2973
15	4B	AX-108806381	0.6329	60.2973
15	4B	AX-110532071	0.6329	60.2973
15	4B	AX-109447978	0.6329	60.2973
15	4B	AX-108951128	0.6329	60.2973
15	4B	AX-108817437	0.6329	60.2973
15	4B	AX-111006678	0.6329	60.2973
15	4B	AX-110016751	0.6329	60.2973
15	4B	AX-110455383	0.6329	60.2973
15	4B	AX-111623537	0.6329	60.2973
15	4B	AX-111732680	0.6329	60.2973
15	4B	AX-109433589	0.6329	60.2973
15	4B	AX-95683711	0.6329	60.2973
15	4B	AX-94984317	0.6329	60.2973
15	4B	AX-109404379	0.6329	60.2973
15	4B	AX-108951002	0.6329	60.2973
15	4B	AX-110565266	0.6329	60.2973
15	4B	AX-111030249	0.6329	60.2973
15	4B	AX-110454444	0.6329	60.2973
15	4B	AX-108857648	0.6329	60.2973
15	4B	AX-109310208	0.6329	60.2973
15	4B	AX-109439557	0.6329	60.2973
15	4B	AX-109844491	0.6329	60.2973
15	4B	AX-111718053	0.6329	60.2973
15	4B	AX-110414026	0.6329	60.2973
15	4B	AX-109833013	0.6329	60.2973
15	4B	AX-110959085	0.6329	60.2973
15	4B	AX-108744638	0.6329	60.2973
15	4B	AX-109057789	0.6329	60.2973

15	4B	AX-111220208	0.6329	60.2973
15	4B	AX-111506685	0.6329	60.2973
15	4B	AX-111469004	0.6329	60.2973
15	4B	AX-110430111	0.6329	60.2973
15	4B	AX-108740622	0.6329	60.2973
15	4B	AX-108950889	0.6329	60.2973
15	4B	AX-108946167	0.6329	60.2973
15	4B	AX-111233935	0.6329	60.2973
15	4B	AX-110979987	0.6329	60.2973
15	4B	AX-110065819	0.6329	60.2973
15	4B	AX-111030278	0.6329	60.2973
15	4B	AX-110563196	0.6329	60.2973
15	4B	AX-109548539	0.6329	60.2973
15	4B	AX-109962441	0.6329	60.2973
15	4B	AX-109852597	0.6329	60.2973
15	4B	AX-111535175	0.6329	60.2973
15	4B	AX-111502947	0.6329	60.2973
15	4B	AX-111517823	0.6329	60.2973
15	4B	AX-109035438	0.6329	60.2973
15	4B	AX-111123110	0.6329	60.2973
15	4B	AX-111679575	0.6329	60.2973
15	4B	AX-111763220	0.6329	60.2973
15	4B	AX-109376685	0.6329	60.2973
15	4B	AX-109428116	0.6329	60.2973
15	4B	AX-111603295	0.6329	60.2973
15	4B	AX-109412041	0.6329	60.2973
15	4B	AX-110931765	0.6329	60.2973
15	4B	AX-111516732	0.6329	60.2973
15	4B	AX-109472157	0.6329	60.2973
15	4B	AX-108903411	0.6329	60.2973
15	4B	AX-110503470	0.6329	60.2973
15	4B	AX-109348616	0.6329	60.2973
15	4B	AX-109904669	0.6329	60.2973
15	4B	AX-109540396	0.6329	60.2973
15	4B	AX-108826066	0.6329	60.2973
15	4B	AX-108788253	0.6329	60.2973
15	4B	AX-108922019	0.6329	60.2973
15	4B	AX-111526146	0.6329	60.2973
15	4B	AX-110507006	0.6329	60.2973
15	4B	AX-110126369	0.6329	60.2973
15	4B	AX-109306109	0.6329	60.2973
15	4B	AX-111504094	0.6329	60.2973
15	4B	AX-108955555	0.6329	60.2973
15	4B	AX-94513518	0.6329	60.2973

15	4B	AX-110984143	0.6329	60.2973
15	4B	AX-111166040	0.6329	60.2973
15	4B	AX-110691633	0.6329	60.2973
15	4B	AX-109366166	0.6329	60.2973
15	4B	AX-111287942	0.6329	60.2973
15	4B	AX-108940901	0.6329	60.2973
15	4B	AX-111026917	0.6329	60.2973
15	4B	AX-109852815	0.6329	60.2973
15	4B	AX-89461386	0.6329	60.2973
15	4B	AX-108895395	0.6329	60.2973
15	4B	AX-108929144	0.6329	60.2973
15	4B	AX-110564627	0.6329	60.2973
15	4B	AX-108911893	0.6329	60.2973
15	4B	AX-109286011	0.6329	60.2973
15	4B	AX-110607041	0.6329	60.2973
15	4B	AX-108855711	0.6329	60.2973
15	4B	AX-109339966	0.6329	60.2973
15	4B	AX-109848007	0.6329	60.2973
15	4B	AX-110415228	0.6329	60.2973
15	4B	AX-110582804	0.6329	60.2973
15	4B	AX-110948513	0.6329	60.2973
15	4B	AX-110950091	0.6329	60.2973
15	4B	AX-95069191	0.6329	60.2973
15	4B	AX-111176940	0.6329	60.2973
15	4B	AX-94634070	0.6329	60.2973
15	4B	AX-110679555	0.6383	60.9356
15	4B	AX-110194207	0.6383	60.9356
15	4B	AX-111011650	0.6383	60.9356
15	4B	AX-109927119	0.6383	60.9356
15	4B	AX-111760190	0.6383	60.9356
15	4B	AX-110436318	0.2119	61.1475
15	4B	AX-110536593	0.6494	61.7969
15	4B	AX-108871853	0.2155	62.0124
15	4B	AX-94955456	0.2137	62.2261
15	4B	AX-95658048	0.2137	62.2261
15	4B	AX-112289571	0.2137	62.2261
15	4B	AX-110485684	0.2137	62.2261
15	4B	AX-109560309	0.2137	62.2261
15	4B	AX-110432523	0.2137	62.2261
15	4B	AX-95658062	0.2137	62.2261
15	4B	AX-109364739	0.2137	62.2261
15	4B	AX-111480041	0.2137	62.2261
15	4B	AX-111614173	0.2137	62.2261
15	4B	AX-109887553	0.2137	62.2261

15	4B	AX-108884283	0.2137	62.2261
15	4B	AX-111577080	0.2137	62.2261
15	4B	AX-94498674	0.2137	62.2261
15	4B	AX-111113300	0.2137	62.2261
15	4B	AX-94479174	0.2137	62.2261
15	4B	AX-108807261	0.2137	62.2261
15	4B	AX-108758643	0.2137	62.2261
15	4B	AX-111011125	0.2137	62.2261
15	4B	AX-108855488	0.2137	62.2261
15	4B	AX-110941673	0.2137	62.2261
15	4B	AX-111730654	0.2137	62.2261
15	4B	AX-108730982	0.2137	62.2261
15	4B	AX-111193334	0.2137	62.2261
15	4B	AX-110513231	0.2137	62.2261
15	4B	AX-109911996	0.2128	62.4388
15	4B	AX-89487102	0.2128	62.4388
15	4B	AX-89572432	0.2128	62.4388
15	4B	AX-109312860	0.2128	62.4388
15	4B	AX-108769486	1.5356	63.9744
15	4B	AX-109507081	1.5356	63.9744
15	4B	AX-109270000	1.5356	63.9744
15	4B	AX-111515009	1.5356	63.9744
15	4B	AX-110456665	1.5356	63.9744
15	4B	AX-109492317	1.5356	63.9744
15	4B	AX-109504383	1.5356	63.9744
15	4B	AX-109384691	1.5356	63.9744
15	4B	AX-110428555	1.5356	63.9744
15	4B	AX-109971821	1.5356	63.9744
15	4B	AX-109966346	1.5356	63.9744
15	4B	AX-111040979	1.5356	63.9744
15	4B	AX-110144677	1.5356	63.9744
15	4B	AX-111714854	1.5356	63.9744
15	4B	AX-110561333	1.5356	63.9744
15	4B	AX-108758620	1.5356	63.9744
15	4B	AX-110498186	1.5356	63.9744
15	4B	AX-110712645	1.5356	63.9744
15	4B	AX-108963916	1.5356	63.9744
15	4B	AX-111080767	0.2119	64.1863
15	4B	AX-108941085	0.2119	64.1863
15	4B	AX-111595997	0.2119	64.1863
15	4B	AX-111190784	0.2119	64.1863
15	4B	AX-109273321	0.2101	64.3963
15	4B	AX-111470896	0.2101	64.3963
15	4B	AX-111614542	0.2101	64.3963

15	4B	AX-111649698	0.2101	64.3963
15	4B	AX-110073859	0.8512	65.2475
15	4B	AX-110477194	0.8512	65.2475
15	4B	AX-111571430	0.8512	65.2475
15	4B	AX-110371471	0.8512	65.2475
15	4B	AX-108908364	0.8512	65.2475
15	4B	AX-110481349	0.8512	65.2475
15	4B	AX-111688156	0.8512	65.2475
15	4B	AX-110365797	0.8512	65.2475
15	4B	AX-109438107	0.8512	65.2475
15	4B	AX-109360236	0.8512	65.2475
15	4B	AX-109957494	0.8512	65.2475
15	4B	AX-108879566	0.8512	65.2475
15	4B	AX-94939309	0.8512	65.2475
15	4B	AX-110127992	0.6356	65.8831
15	4B	AX-109480329	0.6356	65.8831
15	4B	AX-111190382	0.6356	65.8831
15	4B	AX-108772821	0.6356	65.8831
15	4B	AX-109324513	0.2128	66.0959
15	4B	AX-110385750	0.4274	66.5232
15	4B	AX-89511402	0.422	66.9452
15	4B	AX-108907053	0.422	66.9452
15	4B	AX-109499393	0.422	66.9452
15	4B	AX-108891115	0.422	66.9452
15	4B	AX-109921641	0.422	66.9452
15	4B	AX-111563823	0.422	66.9452
15	4B	AX-109837690	0.422	66.9452
15	4B	AX-109841712	0.422	66.9452
15	4B	AX-111578765	0.422	66.9452
15	4B	AX-95233137	0.422	66.9452
15	4B	AX-111135044	5.1103	72.0555
15	4B	AX-110565007	5.1103	72.0555
15	4B	AX-110430517	5.1103	72.0555
15	4B	AX-109457891	5.1103	72.0555
15	4B	AX-111492999	1.0685	73.1241
15	4B	AX-110131125	1.0685	73.1241
15	4B	AX-110598531	1.0685	73.1241
15	4B	AX-109386080	0.2092	73.3333
15	4B	AX-109033629	0.2092	73.3333
15	4B	AX-108976434	0.2092	73.3333
15	4B	AX-108820559	0.2092	73.3333
15	4B	AX-111777271	0.2092	73.3333
15	4B	AX-110574154	0.2092	73.3333
15	4B	AX-109054720	0.2092	73.3333

15	4B	AX-110167057	0.2092	73.3333
15	4B	AX-109815865	0.2092	73.3333
15	4B	AX-111081984	0.2092	73.3333
15	4B	AX-110940415	0.2092	73.3333
15	4B	AX-109376424	0.6438	73.9771
15	4B	AX-110052025	0.2128	74.1898
15	4B	AX-109306009	0.2128	74.1898
15	4B	AX-109430069	0.2128	74.1898
15	4B	AX-109847052	0.2128	74.1898
15	4B	AX-86174787	0.422	74.6118
15	4B	AX-109344792	0.422	74.6118
15	4B	AX-110128814	0.422	74.6118
15	4B	AX-111034680	2.4356	77.0473
15	4B	AX-110621521	0.8548	77.9021
15	4B	AX-109479195	4.8765	82.7786
15	4B	AX-110566246	4.8765	82.7786
15	4B	AX-111559221	0.211	82.9896
15	4B	AX-109314176	0.211	82.9896
15	4B	AX-111064477	0.211	82.9896
15	4B	AX-108828519	0.211	82.9896
15	4B	AX-109283891	0.211	82.9896
15	4B	AX-111633224	0.211	82.9896
15	4B	AX-111560086	0.211	82.9896
15	4B	AX-111060717	0.2092	83.1988
15	4B	AX-110963704	0.2092	83.1988
15	4B	AX-95659226	0.2092	83.1988
15	4B	AX-111615981	0.6329	83.8318
15	4B	AX-111058090	0.6329	83.8318
15	4B	AX-109411152	3.8714	87.7031
15	4B	AX-110075169	3.8714	87.7031
15	4B	AX-108801712	0.211	87.9141
15	4B	AX-110472645	0.211	87.9141
15	4B	AX-111520953	1.7398	89.6539
15	4B	AX-110028480	0.2101	89.864
15	4B	AX-109618065	0.2101	89.864
15	4B	AX-110483442	0.2101	89.864
15	4B	AX-109935637	0.2101	89.864
15	4B	AX-109035659	0.2101	89.864
15	4B	AX-111566639	0.2101	89.864
15	4B	AX-108726239	0.2101	89.864
15	4B	AX-109924201	0.2101	89.864
15	4B	AX-111111185	0.2101	89.864
15	4B	AX-110617578	0.6356	90.4996
15	4B	AX-111160295	0.8512	91.3508

15	4B	AX-110918369	0.8512	91.3508
15	4B	AX-94484839	0.8512	91.3508
15	4B	AX-110533856	0.8512	91.3508
15	4B	AX-111148730	0.8512	91.3508
15	4B	AX-109403778	0.2092	91.56
15	4B	AX-111130103	0.8475	92.4075
15	4B	AX-108756572	0.8475	92.4075
15	4B	AX-111494463	0.2083	92.6159
15	4B	AX-109862094	0.2083	92.6159
15	4B	AX-112286209	0.2083	92.6159
15	4B	AX-108884340	0.4184	93.0343
15	4B	AX-110967502	0.8475	93.8818
15	4B	AX-109865405	0.8475	93.8818
15	4B	AX-110620239	0.2092	94.091
15	4B	AX-109425204	0.2092	94.091
15	4B	AX-110960396	0.2092	94.091
15	4B	AX-109971274	0.2083	94.2994
15	4B	AX-111079978	0.2083	94.2994
15	4B	AX-111613340	0.2092	94.5086
15	4B	AX-109270871	0.431	94.9396
15	4B	AX-111074651	0.2137	95.1533
15	4B	AX-109287178	0.2137	95.1533
15	4B	AX-111120836	0.2137	95.1533
15	4B	AX-108780079	0.2137	95.1533
15	4B	AX-110060184	0.2137	95.1533
15	4B	AX-111495296	0.2137	95.1533
15	4B	AX-111039021	0.4202	95.5735
15	4B	AX-94448564	0.4202	95.5735
15	4B	AX-111041469	0.4202	95.5735
15	4B	AX-109363329	3.6428	99.2163
15	4B	AX-110070905	3.1432	102.3594
15	4B	AX-110673891	1.2715	103.6309
15	4B	AX-109455450	1.2715	103.6309
15	4B	AX-111057050	1.2715	103.6309
15	4B	AX-111662284	1.2715	103.6309
15	4B	AX-110046789	1.2715	103.6309
15	4B	AX-108901659	1.2715	103.6309
15	4B	AX-109320907	1.2715	103.6309
15	4B	AX-109404256	1.2715	103.6309
15	4B	AX-110367748	1.2715	103.6309
15	4B	AX-110167478	1.2715	103.6309
15	4B	AX-111098830	1.2715	103.6309
15	4B	AX-108824344	1.2715	103.6309
15	4B	AX-111502259	1.2715	103.6309

15	4B	AX-109468664	1.2715	103.6309
15	4B	AX-110019372	1.2715	103.6309
15	4B	AX-110556474	1.2715	103.6309
15	4B	AX-111073679	1.2715	103.6309
15	4B	AX-110673680	1.2715	103.6309
15	4B	AX-111256510	1.2715	103.6309
15	4B	AX-95178631	1.2715	103.6309
15	4B	AX-108756025	2.8793	106.5102
15	4B	AX-110573767	0.6329	107.1431
15	4B	AX-109440683	0.6329	107.1431
15	4B	AX-111150060	11.3299	118.473
15	4B	AX-111556599	0.211	118.684
15	4B	AX-111159798	1.299	119.983
15	4B	AX-109888571	6.2224	126.2054
15	4B	AX-111696126	6.2224	126.2054
15	4B	AX-109274025	1.5356	127.741
15	4B	AX-109102843	11.2381	138.9791
15	4B	AX-110472490	11.2381	138.9791
15	4B	AX-94486277	0.2092	139.1883
15	4B	AX-111497363	0.8512	140.0395
15	4B	AX-108755302	0.8512	140.0395
16	4D	AX-110496172	0	0
16	4D	AX-110941433	0	0
16	4D	AX-110389905	0	0
16	4D	AX-110320735	0	0
16	4D	AX-109449898	0.8404	0.8404
16	4D	AX-109559916	0.8404	0.8404
16	4D	AX-109418321	5.3195	6.1599
16	4D	AX-108815871	1.949	8.109
16	4D	AX-111634426	1.949	8.109
16	4D	AX-111543816	6.5272	14.6362
17	4D	AX-111677925	0	0
17	4D	AX-110017824	1.299	1.299
17	4D	AX-89456734	2.4142	3.7132
17	4D	AX-110937266	2.4142	3.7132
17	4D	AX-111093894	1.064	4.7771
17	4D	AX-109284502	1.064	4.7771
17	4D	AX-109290185	1.064	4.7771
17	4D	AX-109265559	1.064	4.7771
17	4D	AX-109834052	1.064	4.7771
17	4D	AX-108772719	1.064	4.7771
17	4D	AX-111807691	1.064	4.7771
17	4D	AX-111683909	1.064	4.7771
17	4D	AX-111651065	1.064	4.7771

17	4D	AX-111546862	1.064	4.7771
17	4D	AX-109752646	1.064	4.7771
17	4D	AX-111615455	1.064	4.7771
17	4D	AX-109529178	1.064	4.7771
17	4D	AX-111536532	1.064	4.7771
17	4D	AX-111644260	1.064	4.7771
17	4D	AX-110086640	8.3286	13.1058
17	4D	AX-110622167	4.1001	17.2058
17	4D	AX-111567155	4.6001	21.8059
17	4D	AX-110005953	1.0685	22.8745
17	4D	AX-111494342	4.6023	27.4768
17	4D	AX-110984743	7.192	34.6688
17	4D	AX-109924587	37.3873	72.0562
17	4D	AX-110443787	37.3873	72.0562
17	4D	AX-111978711	37.3873	72.0562
17	4D	AX-108913628	0.8548	72.911
17	4D	AX-110965371	0.8548	72.911
17	4D	AX-109265317	3.9433	76.8543
17	4D	AX-109985668	0.4329	77.2872
17	4D	AX-110924545	0.8512	78.1384
17	4D	AX-108828678	0.422	78.5603
17	4D	AX-108766535	0.2101	78.7704
17	4D	AX-109122044	0.2101	78.7704
17	4D	AX-110466464	0.4237	79.1941
17	4D	AX-109366488	0.4237	79.1941
17	4D	AX-109887289	0.211	79.4051
17	4D	AX-110389983	0.4274	79.8325
17	4D	AX-110934311	0.4274	79.8325
17	4D	AX-111705921	0.4274	79.8325
17	4D	AX-94509763	0.2128	80.0452
17	4D	AX-110366955	0.2128	80.258
17	4D	AX-111533335	0.2146	80.4726
17	4D	AX-109293367	0.6494	81.122
17	4D	AX-111473168	0.2137	81.3357
17	4D	AX-110027874	0.2137	81.3357
17	4D	AX-110289945	0.2128	81.5484
17	4D	AX-109170501	0.2128	81.5484
17	4D	AX-111559890	0.4274	81.9758
17	4D	AX-109305626	0.4274	81.9758
17	4D	AX-111707375	0.4255	82.4013
17	4D	AX-110164450	2.2041	84.6054
17	4D	AX-110631743	1.5423	86.1477
17	4D	AX-111300023	1.5423	86.1477
17	4D	AX-109494858	1.5423	86.1477

17	4D	AX-111092299	0.2128	86.3605
17	4D	AX-108781235	0.2128	86.3605
17	4D	AX-111002463	0.211	86.5715
17	4D	AX-89611245	0.6383	87.2098
17	4D	AX-110476142	0.6383	87.2098
17	4D	AX-111007239	0.211	87.4208
17	4D	AX-109818999	0.6383	88.0591
17	4D	AX-111444133	0.2101	88.2692
17	4D	AX-89349130	0.211	88.4802
17	4D	AX-169337603	0.2119	88.692
17	4D	AX-111601811	1.7707	90.4627
17	4D	AX-109880021	1.7628	92.2255
17	4D	AX-89421399	0.4202	92.6457
17	4D	AX-109431840	0.6329	93.2787
17	4D	AX-111952169	0.6329	93.2787
17	4D	AX-109325949	0.6329	93.2787
17	4D	AX-109390946	1.5156	94.7943
17	4D	AX-110425112	1.5156	94.7943
17	4D	AX-109376608	1.5156	94.7943
17	4D	AX-110427760	1.5156	94.7943
17	4D	AX-109383409	1.5156	94.7943
17	4D	AX-111009002	1.5156	94.7943
17	4D	AX-109852431	1.5156	94.7943
17	4D	AX-111522626	1.5156	94.7943
17	4D	AX-111008716	1.5156	94.7943
17	4D	AX-109861250	1.5156	94.7943
17	4D	AX-111527211	1.5156	94.7943
17	4D	AX-108810050	1.5156	94.7943
17	4D	AX-108820605	1.5156	94.7943
17	4D	AX-109372402	1.5156	94.7943
17	4D	AX-109052044	1.5156	94.7943
17	4D	AX-110378245	1.5156	94.7943
17	4D	AX-109974147	1.5156	94.7943
17	4D	AX-111531162	1.5156	94.7943
17	4D	AX-86166445	1.5156	94.7943
17	4D	AX-110412445	0.422	95.2162
17	4D	AX-111457534	0.422	95.2162
17	4D	AX-109155422	0.422	95.2162
17	4D	AX-108762041	0.422	95.2162
17	4D	AX-111083401	0.422	95.2162
17	4D	AX-109893265	0.422	95.2162
17	4D	AX-110536742	0.422	95.2162
17	4D	AX-111157475	0.422	95.2162
17	4D	AX-110010928	0.422	95.2162

17	4D	AX-109511556	0.422	95.2162
17	4D	AX-108801189	0.422	95.2162
17	4D	AX-109883270	0.422	95.2162
17	4D	AX-111057245	0.422	95.2162
17	4D	AX-109737407	0.422	95.2162
17	4D	AX-110445730	0.422	95.2162
17	4D	AX-111014520	0.422	95.2162
17	4D	AX-109313992	0.422	95.2162
17	4D	AX-111907789	0.422	95.2162
17	4D	AX-109732736	0.422	95.2162
17	4D	AX-109455171	0.422	95.2162
17	4D	AX-111864095	0.422	95.2162
17	4D	AX-109317713	0.422	95.2162
17	4D	AX-109190913	0.422	95.2162
17	4D	AX-110347270	0.422	95.2162
17	4D	AX-108824655	0.422	95.2162
17	4D	AX-110837046	0.422	95.2162
17	4D	AX-110593292	0.422	95.2162
17	4D	AX-110973711	0.422	95.2162
17	4D	AX-111681849	0.422	95.2162
17	4D	AX-95211683	0.422	95.2162
17	4D	AX-111550963	0.422	95.2162
17	4D	AX-109492050	0.422	95.2162
17	4D	AX-110030535	0.422	95.2162
17	4D	AX-109688946	0.422	95.2162
17	4D	AX-109911744	0.422	95.2162
17	4D	AX-109683615	0.422	95.2162
17	4D	AX-109660669	0.422	95.2162
17	4D	AX-111715250	0.422	95.2162
17	4D	AX-109448234	0.2101	95.4263
17	4D	AX-89687758	0.2101	95.4263
17	4D	AX-109405031	0.2101	95.4263
17	4D	AX-111003706	0.2101	95.4263
17	4D	AX-110956578	0.2101	95.4263
17	4D	AX-108867384	0.2101	95.4263
17	4D	AX-111453694	0.2101	95.4263
17	4D	AX-109351119	0.2101	95.4263
17	4D	AX-111562218	0.2101	95.4263
17	4D	AX-110466723	0.2101	95.4263
17	4D	AX-110446042	0.2101	95.4263
17	4D	AX-109880491	0.2101	95.4263
17	4D	AX-111637305	0.2101	95.4263
17	4D	AX-110610376	0.2101	95.4263
17	4D	AX-109814897	0.2101	95.4263

17	4D	AX-109860907	0.2101	95.4263
17	4D	AX-111677282	0.2101	95.4263
17	4D	AX-111921300	5.1342	100.5606
17	4D	AX-108786137	7.3337	107.8943
17	4D	AX-111032296	4.8315	112.7258
17	4D	AX-109989520	2.8793	115.605
17	4D	AX-110450682	1.9661	117.5711
17	4D	AX-89624188	23.0134	140.5845
17	4D	AX-109303311	0.4202	141.0047
17	4D	AX-111024002	0.4202	141.0047
17	4D	AX-109194168	0.4202	141.0047
17	4D	AX-111480164	1.0685	142.0733
17	4D	AX-110479846	10.6159	152.6891
18	5A	AX-108953717	0	0
18	5A	AX-111067709	0	0
18	5A	AX-108793692	0.2092	0.2092
18	5A	AX-109304999	0.2092	0.2092
18	5A	AX-111275827	1.0731	1.2823
18	5A	AX-109926388	7.8692	9.1515
18	5A	AX-110144281	7.8692	9.1515
18	5A	AX-110463209	7.8692	9.1515
18	5A	AX-111471149	7.8692	9.1515
18	5A	AX-109505995	0.2092	9.3608
18	5A	AX-109959535	7.5078	16.8686
18	5A	AX-108846531	7.5078	16.8686
18	5A	AX-111533410	2.4036	19.2722
18	5A	AX-111086728	3.6263	22.8984
18	5A	AX-110529161	3.6263	22.8984
18	5A	AX-109963060	3.6263	22.8984
18	5A	AX-109928034	2.1848	25.0832
18	5A	AX-109490163	2.1848	25.0832
18	5A	AX-110529159	2.1848	25.0832
18	5A	AX-109995006	0.4237	25.507
18	5A	AX-110434983	0.6383	26.1453
18	5A	AX-109322525	0.4202	26.5655
18	5A	AX-108938452	0.6383	27.2038
18	5A	AX-109465909	4.6214	31.8252
18	5A	AX-110006805	0.2101	32.0353
18	5A	AX-109944695	0.2101	32.0353
18	5A	AX-108856072	0.2101	32.0353
18	5A	AX-108987114	4.0814	36.1168
18	5A	AX-109284303	2.4248	38.5416
18	5A	AX-110964036	0.2101	38.7516
18	5A	AX-110561468	0.2101	38.7516

18	5A	AX-111031828	0.2101	38.7516
18	5A	AX-111145365	0.2101	38.7516
18	5A	AX-110551659	0.2101	38.7516
18	5A	AX-110394993	0.2101	38.7516
18	5A	AX-108827974	0.2101	38.7516
18	5A	AX-111520163	0.2101	38.7516
18	5A	AX-109025083	0.2101	38.7516
18	5A	AX-110470906	0.2101	38.7516
18	5A	AX-109337769	0.2101	38.7516
18	5A	AX-110440664	2.6692	41.4208
18	5A	AX-110396568	0.2101	41.6309
18	5A	AX-111012018	0.2101	41.6309
18	5A	AX-109599808	0.2083	41.8393
18	5A	AX-108936302	0.2101	42.0493
18	5A	AX-110547149	0.2101	42.0493
18	5A	AX-111054067	0.2101	42.0493
18	5A	AX-111519092	0.2101	42.0493
18	5A	AX-108734535	0.2101	42.0493
18	5A	AX-110982869	0.2101	42.0493
18	5A	AX-111103432	0.2101	42.0493
18	5A	AX-111580908	0.2101	42.0493
18	5A	AX-110905496	0.2101	42.0493
18	5A	AX-109271112	0.2101	42.0493
18	5A	AX-108920706	0.2101	42.0493
18	5A	AX-110454232	0.2101	42.0493
18	5A	AX-109857484	0.2101	42.0493
18	5A	AX-110067492	0.2101	42.0493
18	5A	AX-110717565	0.2101	42.0493
18	5A	AX-111018293	0.2101	42.0493
18	5A	AX-108742869	0.2101	42.0493
18	5A	AX-109327589	0.2101	42.0493
18	5A	AX-110970894	0.2101	42.2594
18	5A	AX-110525457	0.2083	42.4678
18	5A	AX-109321224	0.2083	42.4678
18	5A	AX-111087942	0.2083	42.4678
18	5A	AX-108828572	0.2083	42.4678
18	5A	AX-110033504	0.2083	42.4678
18	5A	AX-108785757	0.2075	42.6752
18	5A	AX-109545993	0.844	43.5192
18	5A	AX-111101565	0.844	43.5192
18	5A	AX-110688523	0.2083	43.7275
18	5A	AX-111014124	0.2083	43.7275
18	5A	AX-111619095	0.2083	43.7275
18	5A	AX-109440531	0.2083	43.7275

18	5A	AX-108901608	0.2083	43.7275
18	5A	AX-110932081	0.2083	43.7275
18	5A	AX-110422901	0.4184	44.146
18	5A	AX-109307590	0.4184	44.146
18	5A	AX-109040666	0.4184	44.146
18	5A	AX-109317915	0.4184	44.146
18	5A	AX-110970581	0.4184	44.146
18	5A	AX-111589294	0.4184	44.146
18	5A	AX-111015371	0.4184	44.146
18	5A	AX-108969591	0.4184	44.146
18	5A	AX-109906435	0.4184	44.146
18	5A	AX-111079496	0.4184	44.146
18	5A	AX-109397362	0.4184	44.146
18	5A	AX-109448093	0.4184	44.146
18	5A	AX-110438266	0.4184	44.146
18	5A	AX-110122255	0.4184	44.146
18	5A	AX-110368941	0.4184	44.146
18	5A	AX-110454701	0.4184	44.146
18	5A	AX-110524632	0.4184	44.146
18	5A	AX-110368156	0.4184	44.146
18	5A	AX-109988896	0.4184	44.146
18	5A	AX-111135243	0.4184	44.146
18	5A	AX-109451388	0.4184	44.146
18	5A	AX-109295959	0.4184	44.146
18	5A	AX-110460517	0.4184	44.146
18	5A	AX-108939482	0.4184	44.146
18	5A	AX-108977375	0.4184	44.146
18	5A	AX-110596127	0.4184	44.146
18	5A	AX-110942488	0.4184	44.146
18	5A	AX-110002379	0.4184	44.146
18	5A	AX-109395451	0.4184	44.146
18	5A	AX-111573264	0.4184	44.146
18	5A	AX-110404212	0.4184	44.146
18	5A	AX-109416283	0.4184	44.146
18	5A	AX-108842539	0.4184	44.146
18	5A	AX-111484674	0.4184	44.146
18	5A	AX-108901353	0.4184	44.146
18	5A	AX-109479127	0.4184	44.146
18	5A	AX-111130929	0.4184	44.146
18	5A	AX-111220129	0.4184	44.146
18	5A	AX-109520427	0.4184	44.146
18	5A	AX-111451340	0.4184	44.146
18	5A	AX-109880691	0.4184	44.146
18	5A	AX-111623810	0.4184	44.146

18	5A	AX-109858950	0.4184	44.146
18	5A	AX-109654287	0.4202	44.5661
18	5A	AX-108973040	0.4202	44.5661
18	5A	AX-110980253	0.4202	44.5661
18	5A	AX-108769900	0.4202	44.5661
18	5A	AX-108897346	0.6329	45.1991
18	5A	AX-111472634	0.6329	45.1991
18	5A	AX-109440590	0.6329	45.1991
18	5A	AX-108754051	1.7865	46.9855
18	5A	AX-109937099	0.4348	47.4203
18	5A	AX-111489714	0.4348	47.4203
18	5A	AX-111215736	0.211	47.6313
18	5A	AX-111509179	0.211	47.6313
18	5A	AX-110939747	2.2538	49.8851
18	5A	AX-111028216	0.6551	50.5401
18	5A	AX-110607280	0.2137	50.7538
18	5A	AX-111567004	0.2128	50.9666
18	5A	AX-111097570	0.2128	50.9666
18	5A	AX-109490942	0.2128	50.9666
18	5A	AX-111139472	0.2128	50.9666
18	5A	AX-111570320	0.2128	50.9666
18	5A	AX-108770081	0.2128	50.9666
18	5A	AX-110911876	0.6438	51.6104
18	5A	AX-110374482	0.2128	51.8232
18	5A	AX-110127168	0.2119	52.035
18	5A	AX-110430693	0.2119	52.035
18	5A	AX-109580054	0.2119	52.035
18	5A	AX-109446332	0.2119	52.035
18	5A	AX-110583474	0.2119	52.035
18	5A	AX-109357437	0.2119	52.035
18	5A	AX-111041444	0.2119	52.035
18	5A	AX-111547085	0.2119	52.035
18	5A	AX-109459767	0.2137	52.2487
18	5A	AX-109885906	0.2137	52.4624
18	5A	AX-109879982	0.2137	52.4624
18	5A	AX-110995710	0.2137	52.4624
18	5A	AX-109538251	0.2137	52.4624
18	5A	AX-108833915	0.2119	52.6743
18	5A	AX-111511879	0.2119	52.6743
18	5A	AX-110537939	0.2119	52.6743
18	5A	AX-110737781	0.2119	52.6743
18	5A	AX-110542873	0.2119	52.6743
18	5A	AX-109327797	0.2119	52.6743
18	5A	AX-109016531	0.2137	52.888

18	5A	AX-109892612	0.4329	53.3209
18	5A	AX-109362376	0.2155	53.5364
18	5A	AX-110735186	0.2155	53.5364
18	5A	AX-111002856	0.2155	53.5364
18	5A	AX-111513381	0.2155	53.5364
18	5A	AX-110569230	0.4292	53.9656
18	5A	AX-111584250	0.4292	53.9656
18	5A	AX-109076623	0.4292	53.9656
18	5A	AX-111114587	0.4292	53.9656
18	5A	AX-110641373	1.7865	55.752
18	5A	AX-111557804	1.7865	55.752
18	5A	AX-109994159	2.2847	58.0367
18	5A	AX-108905381	0.2155	58.2523
18	5A	AX-109286286	0.2155	58.2523
18	5A	AX-108813905	0.2155	58.2523
18	5A	AX-109836885	0.2155	58.2523
18	5A	AX-109997529	0.2155	58.2523
18	5A	AX-108787312	0.2155	58.2523
18	5A	AX-108966803	0.2155	58.2523
18	5A	AX-110710201	0.2155	58.2523
18	5A	AX-111258544	0.2155	58.2523
18	5A	AX-94985037	0.2155	58.2523
18	5A	AX-111003109	0.2155	58.2523
18	5A	AX-110607073	0.4274	58.6796
18	5A	AX-110561654	0.4274	58.6796
18	5A	AX-109379355	0.4274	58.6796
18	5A	AX-109864923	0.4274	58.6796
18	5A	AX-110028038	0.4274	58.6796
18	5A	AX-109469143	0.4274	58.6796
18	5A	AX-111165469	0.2128	58.8924
18	5A	AX-110382320	0.2128	58.8924
18	5A	AX-109921363	0.2128	58.8924
18	5A	AX-111525382	0.2128	58.8924
18	5A	AX-108738893	0.2128	58.8924
18	5A	AX-108990192	0.2128	58.8924
18	5A	AX-109297951	0.2128	58.8924
18	5A	AX-110369666	0.2128	58.8924
18	5A	AX-110476688	0.2128	58.8924
18	5A	AX-110390809	0.2128	58.8924
18	5A	AX-111024628	0.2128	58.8924
18	5A	AX-109852151	0.2128	58.8924
18	5A	AX-109653659	0.2128	58.8924
18	5A	AX-110200309	0.2128	58.8924
18	5A	AX-110906920	0.2128	58.8924

18	5A	AX-108926681	0.2128	58.8924
18	5A	AX-108872310	0.2128	58.8924
18	5A	AX-109371064	0.2128	58.8924
18	5A	AX-109870540	0.2128	58.8924
18	5A	AX-110490444	0.2128	58.8924
18	5A	AX-94416941	0.2128	58.8924
18	5A	AX-94731008	0.2128	58.8924
18	5A	AX-110954624	1.0871	59.9795
18	5A	AX-108847321	0.6438	60.6233
18	5A	AX-110050812	0.6438	60.6233
18	5A	AX-109343369	0.6438	60.6233
18	5A	AX-109524688	0.6438	60.6233
18	5A	AX-109334693	0.6438	60.6233
18	5A	AX-110393094	0.6438	60.6233
18	5A	AX-109329647	0.6438	60.6233
18	5A	AX-110175915	0.6438	60.6233
18	5A	AX-109403569	0.6438	60.6233
18	5A	AX-111012029	2.1944	62.8177
18	5A	AX-108972668	2.1944	62.8177
18	5A	AX-111566729	2.1944	62.8177
18	5A	AX-111216808	2.1944	62.8177
18	5A	AX-111168284	2.1944	62.8177
18	5A	AX-110950060	2.1944	62.8177
18	5A	AX-111477039	2.1944	62.8177
18	5A	AX-110979282	2.1944	62.8177
18	5A	AX-110468220	2.1944	62.8177
18	5A	AX-111118131	2.1944	62.8177
18	5A	AX-111001073	2.1944	62.8177
18	5A	AX-111478455	2.1944	62.8177
18	5A	AX-108786218	2.1944	62.8177
18	5A	AX-111579554	2.1944	62.8177
18	5A	AX-111574807	2.1944	62.8177
18	5A	AX-111060316	2.1944	62.8177
18	5A	AX-110577137	2.1944	62.8177
18	5A	AX-109900699	2.1944	62.8177
18	5A	AX-108747093	2.1944	62.8177
18	5A	AX-109836390	2.1944	62.8177
18	5A	AX-111495320	2.1944	62.8177
18	5A	AX-111049338	2.1944	62.8177
18	5A	AX-110024137	2.1944	62.8177
18	5A	AX-111172588	0.8404	63.6581
18	5A	AX-111592550	0.2075	63.8656
18	5A	AX-111136203	0.2075	63.8656
18	5A	AX-108925104	0.2075	63.8656

18	5A	AX-111102252	0.2066	64.0722
18	5A	AX-108938796	0.2066	64.0722
18	5A	AX-110508884	0.2066	64.0722
18	5A	AX-111614615	0.2066	64.0722
18	5A	AX-108921148	5.6578	69.7301
18	5A	AX-109492414	5.6578	69.7301
18	5A	AX-110544530	5.6578	69.7301
18	5A	AX-108756770	2.1753	71.9053
18	5A	AX-110454673	2.1753	71.9053
18	5A	AX-111113177	2.1753	71.9053
18	5A	AX-109509847	2.1753	71.9053
18	5A	AX-109035929	2.1753	71.9053
18	5A	AX-111458125	2.1753	71.9053
18	5A	AX-110004899	0.2092	72.1145
18	5A	AX-109446853	0.2101	72.3246
18	5A	AX-108969605	0.422	72.7466
18	5A	AX-111563111	2.6456	75.3922
18	5A	AX-108891432	2.6456	75.3922
18	5A	AX-109512930	2.6456	75.3922
18	5A	AX-109482281	1.0595	76.4517
18	5A	AX-110616505	1.2715	77.7232
18	5A	AX-110012348	1.2715	77.7232
18	5A	AX-110931671	1.2715	77.7232
18	5A	AX-110414195	1.2715	77.7232
18	5A	AX-108946596	1.2715	77.7232
18	5A	AX-110534149	1.2715	77.7232
18	5A	AX-110051038	1.2715	77.7232
18	5A	AX-110908103	1.2715	77.7232
18	5A	AX-108844453	1.2715	77.7232
18	5A	AX-110960939	1.2715	77.7232
18	5A	AX-109457191	1.2715	77.7232
18	5A	AX-111556159	0.4167	78.1398
18	5A	AX-109897379	0.4167	78.1398
18	5A	AX-110169212	0.4167	78.1398
18	5A	AX-109032399	0.4167	78.1398
18	5A	AX-108966524	0.4167	78.1398
18	5A	AX-110360199	0.4167	78.1398
18	5A	AX-110948784	0.4167	78.1398
18	5A	AX-109880001	0.4167	78.1398
18	5A	AX-108899169	0.4167	78.1398
18	5A	AX-110712717	0.4167	78.1398
18	5A	AX-111102603	0.4167	78.1398
18	5A	AX-109488189	0.4167	78.1398
18	5A	AX-110961832	0.4167	78.1398

18	5A	AX-110674421	0.4167	78.1398
18	5A	AX-109418106	0.4167	78.1398
18	5A	AX-111557124	0.4167	78.1398
18	5A	AX-110683877	0.4167	78.5565
18	5A	AX-109466244	1.9575	80.514
18	5A	AX-110985623	0.2101	80.7241
18	5A	AX-109378245	0.2101	80.7241
18	5A	AX-111521631	0.2101	80.7241
18	5A	AX-109490721	0.2101	80.7241
18	5A	AX-109278999	0.2101	80.7241
18	5A	AX-110997238	0.2101	80.7241
18	5A	AX-108832414	0.2101	80.7241
18	5A	AX-109941244	0.2101	80.7241
18	5A	AX-111256928	0.2101	80.7241
18	5A	AX-109944510	0.2101	80.7241
18	5A	AX-109864419	0.2101	80.7241
18	5A	AX-109999956	0.2101	80.7241
18	5A	AX-111535607	0.2101	80.7241
18	5A	AX-109390832	0.2101	80.7241
18	5A	AX-111056986	0.2101	80.7241
18	5A	AX-108821399	0.2101	80.7241
18	5A	AX-111514256	0.2101	80.7241
18	5A	AX-111570835	0.2101	80.7241
18	5A	AX-109042952	0.2101	80.7241
18	5A	AX-110979994	0.2101	80.7241
18	5A	AX-108737976	0.422	81.1461
18	5A	AX-111186399	0.422	81.1461
18	5A	AX-110003296	0.422	81.1461
18	5A	AX-108854151	0.422	81.1461
18	5A	AX-111464011	0.422	81.1461
18	5A	AX-110508475	0.422	81.1461
18	5A	AX-108937232	0.422	81.1461
18	5A	AX-110521294	0.422	81.1461
18	5A	AX-109894368	0.422	81.1461
18	5A	AX-108809668	0.422	81.1461
18	5A	AX-108738095	0.422	81.1461
18	5A	AX-108970949	0.422	81.1461
18	5A	AX-108931449	0.422	81.1461
18	5A	AX-110370324	0.422	81.1461
18	5A	AX-110433931	0.422	81.1461
18	5A	AX-109418058	0.422	81.1461
18	5A	AX-109078650	0.422	81.1461
18	5A	AX-110154163	0.422	81.1461
18	5A	AX-110426159	0.422	81.1461

18	5A	AX-110044116	0.422	81.1461
18	5A	AX-109932358	0.422	81.1461
18	5A	AX-110930295	0.422	81.1461
18	5A	AX-108892277	0.422	81.1461
18	5A	AX-109340672	0.422	81.1461
18	5A	AX-109749260	0.422	81.1461
18	5A	AX-109822164	0.422	81.1461
18	5A	AX-110191201	0.422	81.1461
18	5A	AX-109942416	0.422	81.1461
18	5A	AX-108779016	0.422	81.1461
18	5A	AX-108910752	0.422	81.1461
18	5A	AX-109331493	0.422	81.1461
18	5A	AX-108760364	0.422	81.1461
18	5A	AX-111165483	0.422	81.1461
18	5A	AX-110634350	0.422	81.1461
18	5A	AX-110999146	0.422	81.1461
18	5A	AX-108832296	0.422	81.1461
18	5A	AX-109320799	0.422	81.1461
18	5A	AX-111554972	0.422	81.1461
18	5A	AX-109948025	0.422	81.1461
18	5A	AX-109309770	0.422	81.1461
18	5A	AX-108978359	0.422	81.1461
18	5A	AX-109941450	0.422	81.1461
18	5A	AX-110028672	0.422	81.1461
18	5A	AX-110597075	0.422	81.1461
18	5A	AX-110610151	0.422	81.1461
18	5A	AX-109845949	0.422	81.1461
18	5A	AX-108889203	0.422	81.1461
18	5A	AX-110383644	0.422	81.1461
18	5A	AX-110521280	0.422	81.1461
18	5A	AX-111212447	0.422	81.1461
18	5A	AX-110498121	0.422	81.1461
18	5A	AX-111460610	0.422	81.1461
18	5A	AX-109440091	0.422	81.1461
18	5A	AX-109834514	0.422	81.1461
18	5A	AX-111112941	0.422	81.1461
18	5A	AX-108848765	0.422	81.1461
18	5A	AX-108863758	0.422	81.1461
18	5A	AX-108789207	0.422	81.1461
18	5A	AX-110045648	0.422	81.1461
18	5A	AX-110048492	0.422	81.1461
18	5A	AX-110679088	0.422	81.1461
18	5A	AX-110490632	0.422	81.1461
18	5A	AX-109824289	0.422	81.1461

18	5A	AX-111731911	0.422	81.1461
18	5A	AX-108905278	0.422	81.1461
18	5A	AX-109278551	0.422	81.1461
18	5A	AX-94615731	0.422	81.1461
18	5A	AX-94423065	0.422	81.1461
18	5A	AX-95231152	0.422	81.1461
18	5A	AX-109901375	0.422	81.1461
18	5A	AX-110948882	0.422	81.1461
18	5A	AX-95109370	0.422	81.1461
18	5A	AX-111702691	0.422	81.1461
18	5A	AX-111175437	0.422	81.1461
18	5A	AX-109914855	0.422	81.1461
18	5A	AX-110377479	0.422	81.1461
18	5A	AX-111569540	0.422	81.1461
18	5A	AX-94575846	0.422	81.1461
18	5A	AX-95193117	0.422	81.1461
18	5A	AX-95203876	0.422	81.1461
18	5A	AX-110413381	0.4237	81.5698
18	5A	AX-111459591	0.4237	81.5698
18	5A	AX-111498021	0.4237	81.5698
18	5A	AX-111216294	0.4237	81.5698
18	5A	AX-109509040	0.4237	81.5698
18	5A	AX-110506483	0.4237	81.5698
18	5A	AX-108867337	0.4237	81.5698
18	5A	AX-110973938	0.4237	81.5698
18	5A	AX-108759522	0.4237	81.5698
18	5A	AX-111673092	0.4237	81.5698
18	5A	AX-108808786	0.4237	81.5698
18	5A	AX-111568499	0.4237	81.5698
18	5A	AX-109511251	0.4237	81.5698
18	5A	AX-110944663	0.4237	81.5698
18	5A	AX-111540346	0.4237	81.5698
18	5A	AX-95225298	0.4237	81.5698
18	5A	AX-94949102	0.4237	81.5698
18	5A	AX-109285497	7.37	88.9398
18	5A	AX-110173288	1.9747	90.9145
18	5A	AX-109855396	1.299	92.2135
18	5A	AX-108861269	1.5091	93.7226
18	5A	AX-109281221	1.5091	93.7226
18	5A	AX-110918930	0.2083	93.9309
18	5A	AX-108760060	0.2083	93.9309
18	5A	AX-110408704	0.2083	93.9309
18	5A	AX-109400961	0.2083	93.9309
18	5A	AX-108937402	0.2083	93.9309

18	5A	AX-110667672	0.2083	93.9309
18	5A	AX-110192339	0.2083	93.9309
18	5A	AX-111100693	0.2083	93.9309
18	5A	AX-111584190	0.2083	93.9309
18	5A	AX-110957544	0.2083	93.9309
18	5A	AX-110071060	0.2083	93.9309
18	5A	AX-109622137	0.2083	93.9309
18	5A	AX-108964722	0.2083	93.9309
18	5A	AX-109991235	0.2083	93.9309
18	5A	AX-110739984	0.2083	93.9309
18	5A	AX-111104892	0.2083	94.1393
18	5A	AX-110593436	0.4202	94.5594
18	5A	AX-109486542	0.4202	94.5594
18	5A	AX-110199675	0.4202	94.5594
18	5A	AX-110456705	0.4202	94.5594
18	5A	AX-111537341	0.4202	94.5594
18	5A	AX-111122310	0.4202	94.5594
18	5A	AX-110906234	0.4202	94.5594
18	5A	AX-110549800	0.4202	94.5594
18	5A	AX-108807482	0.4202	94.5594
18	5A	AX-108735572	0.4202	94.5594
18	5A	AX-95013869	0.4202	94.5594
18	5A	AX-110437938	2.8666	97.426
18	5A	AX-109346674	2.8666	97.426
18	5A	AX-110595198	2.8666	97.426
18	5A	AX-108958924	2.8666	97.426
18	5A	AX-110927950	0.2092	97.6352
18	5A	AX-108810565	0.211	97.8462
18	5A	AX-111083486	0.2119	98.0581
18	5A	AX-109325755	0.2119	98.0581
18	5A	AX-110125815	0.2119	98.0581
18	5A	AX-109881260	0.2119	98.0581
18	5A	AX-108778477	0.2119	98.0581
18	5A	AX-111052396	0.2119	98.0581
18	5A	AX-110991433	0.2119	98.0581
18	5A	AX-109894868	0.2119	98.0581
18	5A	AX-110527123	0.2119	98.0581
18	5A	AX-110480590	0.2119	98.0581
18	5A	AX-111100071	0.2119	98.0581
18	5A	AX-111520340	0.2119	98.0581
18	5A	AX-111103773	0.2119	98.0581
18	5A	AX-110478402	0.2119	98.0581
18	5A	AX-109275686	0.2119	98.0581
18	5A	AX-108891532	0.2119	98.0581

18	5A	AX-109837673	0.2119	98.0581
18	5A	AX-111539014	0.2119	98.0581
18	5A	AX-108791192	0.2119	98.0581
18	5A	AX-109466196	0.2119	98.0581
18	5A	AX-111543503	0.2119	98.0581
18	5A	AX-109309205	0.2119	98.0581
18	5A	AX-109911741	0.2119	98.0581
18	5A	AX-108849904	0.2119	98.0581
18	5A	AX-110462055	0.2119	98.0581
18	5A	AX-108867633	0.2119	98.0581
18	5A	AX-110584329	0.2119	98.0581
18	5A	AX-111512733	0.2119	98.0581
18	5A	AX-109905084	0.2119	98.0581
18	5A	AX-110926692	0.2119	98.0581
18	5A	AX-109305830	0.2119	98.0581
18	5A	AX-110048398	0.2119	98.0581
18	5A	AX-109300443	0.2119	98.0581
18	5A	AX-108784916	0.2119	98.0581
18	5A	AX-108855539	0.2119	98.0581
18	5A	AX-110439469	0.2119	98.0581
18	5A	AX-110442010	0.2119	98.0581
18	5A	AX-111039686	0.2119	98.0581
18	5A	AX-110969110	0.2119	98.0581
18	5A	AX-109327091	0.2119	98.0581
18	5A	AX-110982973	0.2119	98.0581
18	5A	AX-111485276	0.2119	98.0581
18	5A	AX-108917315	0.2119	98.0581
18	5A	AX-108904353	0.2119	98.0581
18	5A	AX-111110440	0.2119	98.0581
18	5A	AX-109392935	0.2119	98.0581
18	5A	AX-109038371	0.2119	98.0581
18	5A	AX-110411073	0.2119	98.0581
18	5A	AX-111090974	0.2119	98.0581
18	5A	AX-109367047	0.2119	98.0581
18	5A	AX-108847062	0.2119	98.0581
18	5A	AX-110985794	0.2119	98.0581
18	5A	AX-109355412	0.2119	98.0581
18	5A	AX-109270283	0.2119	98.0581
18	5A	AX-111575475	0.2119	98.0581
18	5A	AX-110929014	0.2119	98.0581
18	5A	AX-111570699	0.2119	98.0581
18	5A	AX-110443555	0.2119	98.0581
18	5A	AX-109934392	0.2119	98.0581
18	5A	AX-111618105	0.2119	98.0581

18	5A	AX-110990493	0.2119	98.0581
18	5A	AX-111115088	0.2119	98.0581
18	5A	AX-110941592	0.2119	98.0581
18	5A	AX-108935540	0.2119	98.0581
18	5A	AX-109857378	0.2119	98.0581
18	5A	AX-111744186	0.2119	98.0581
18	5A	AX-109451305	0.2119	98.0581
18	5A	AX-109915142	0.2119	98.0581
18	5A	AX-94664087	0.2119	98.0581
18	5A	AX-109955961	0.2119	98.0581
18	5A	AX-110072179	0.2119	98.0581
18	5A	AX-111213664	0.2119	98.0581
18	5A	AX-109817972	0.6551	98.7131
18	5A	AX-110949650	0.2165	98.9296
18	5A	AX-109426068	0.2165	98.9296
18	5A	AX-108861187	0.2165	98.9296
18	5A	AX-110076201	0.2165	98.9296
18	5A	AX-109919444	0.2165	98.9296
18	5A	AX-109898601	0.2083	99.1379
18	5A	AX-109369427	2.6456	101.7835
18	5A	AX-111475234	1.5091	103.2926
18	5A	AX-111451573	1.2823	104.575
18	5A	AX-110016633	0.2092	104.7842
18	5A	AX-109879378	0.2092	104.7842
18	5A	AX-110973091	0.2092	104.7842
18	5A	AX-111037298	0.2092	104.7842
18	5A	AX-109814839	0.2092	104.7842
18	5A	AX-109980237	0.2092	104.9934
18	5A	AX-110472228	1.5091	106.5024
18	5A	AX-110121838	1.5091	106.5024
18	5A	AX-110373243	1.5091	106.5024
18	5A	AX-111506162	0.2092	106.7117
18	5A	AX-110905005	0.2092	106.7117
18	5A	AX-111463331	0.6329	107.3446
18	5A	AX-111451217	0.2092	107.5538
18	5A	AX-110595882	0.2092	107.5538
18	5A	AX-110387834	0.2092	107.5538
18	5A	AX-108881065	0.2092	107.5538
18	5A	AX-110442218	0.2092	107.5538
18	5A	AX-108894206	0.2092	107.5538
18	5A	AX-108879222	0.2092	107.5538
18	5A	AX-109824014	0.2092	107.5538
18	5A	AX-111010669	0.2092	107.5538
18	5A	AX-111517269	0.2092	107.5538

18	5A	AX-108790581	0.2092	107.5538
18	5A	AX-109535868	0.2092	107.5538
18	5A	AX-109340261	0.2092	107.5538
18	5A	AX-111554770	0.2092	107.5538
18	5A	AX-108912268	0.2119	107.7657
18	5A	AX-111514440	3.8538	111.6194
18	5A	AX-109494596	2.9852	114.6046
18	5A	AX-110460283	0.6303	115.2349
18	5A	AX-109398903	0.2075	115.4424
18	5A	AX-109271586	0.2075	115.4424
18	5A	AX-109960078	0.4184	115.8608
18	5A	AX-109819591	0.8548	116.7156
18	5A	AX-109102595	1.5091	118.2247
18	5A	AX-111596791	0.2092	118.4339
18	5A	AX-110954181	0.4202	118.8541
18	5A	AX-110947807	1.2878	120.1419
18	5A	AX-111253877	1.2878	120.1419
18	5A	AX-109952547	1.5356	121.6775
18	5A	AX-109880667	0.2128	121.8902
18	5A	AX-110051624	0.2128	121.8902
18	5A	AX-111453179	0.2128	121.8902
18	5A	AX-110408057	0.2128	121.8902
18	5A	AX-109940654	0.2128	121.8902
18	5A	AX-108854725	0.2128	121.8902
18	5A	AX-111235464	0.2128	121.8902
18	5A	AX-111152617	0.6466	122.5368
18	5A	AX-110463206	0.2146	122.7514
18	5A	AX-111463040	1.3219	124.0733
18	5A	AX-111031457	1.3219	124.0733
18	5A	AX-110713956	1.3219	124.0733
18	5A	AX-111141678	1.3219	124.0733
18	5A	AX-109895415	1.3219	124.0733
18	5A	AX-109639015	1.3219	124.0733
18	5A	AX-111078733	1.3219	124.0733
18	5A	AX-109987807	0.6522	124.7255
18	5A	AX-111175009	0.2155	124.941
18	5A	AX-109858577	0.2155	124.941
18	5A	AX-111102726	0.2155	124.941
18	5A	AX-111135802	0.2155	124.941
18	5A	AX-109876198	0.2165	125.1575
18	5A	AX-109514517	0.2165	125.374
18	5A	AX-111488805	0.2137	125.5876
18	5A	AX-111091022	0.2137	125.5876
18	5A	AX-111590180	0.4292	126.0168

18	5A	AX-109411991	0.4292	126.0168
18	5A	AX-109900224	0.431	126.4479
18	5A	AX-111472310	0.431	126.4479
18	5A	AX-110915393	0.2128	126.6606
18	5A	AX-108805233	0.2128	126.6606
18	5A	AX-111117681	0.2128	126.6606
18	5A	AX-109428297	0.2119	126.8725
18	5A	AX-110469756	4.1189	130.9914
18	5A	AX-109356883	4.1189	130.9914
18	5A	AX-109970129	4.1189	130.9914
18	5A	AX-109600232	4.1189	130.9914
18	5A	AX-108976406	4.1189	130.9914
18	5A	AX-108939101	4.1189	130.9914
18	5A	AX-109840534	4.1189	130.9914
18	5A	AX-108762422	4.1189	130.9914
18	5A	AX-110508321	4.1189	130.9914
18	5A	AX-111584553	4.1189	130.9914
18	5A	AX-109429589	4.1189	130.9914
18	5A	AX-111569463	4.1189	130.9914
18	5A	AX-110374801	4.1189	130.9914
18	5A	AX-110598576	0.2083	131.1997
18	5A	AX-108892891	0.2083	131.1997
18	5A	AX-109856068	0.2083	131.1997
18	5A	AX-108821489	0.2164	131.4162
18	5A	AX-109919064	0.2164	131.6326
18	5A	AX-109961496	0.2164	131.6326
18	5A	AX-108922280	0.2164	131.6326
18	5A	AX-109820933	0.2164	131.6326
18	5A	AX-109892753	0.2164	131.6326
18	5A	AX-111621976	0.2164	131.6326
18	5A	AX-111095417	0.2164	131.6326
18	5A	AX-109831701	0.2164	131.6326
18	5A	AX-109407713	0.2164	131.6326
18	5A	AX-109375717	0.2164	131.6326
18	5A	AX-109512380	0.2164	131.6326
18	5A	AX-110957925	0.2164	131.6326
18	5A	AX-108885457	0.2164	131.6326
18	5A	AX-111031529	0.2164	131.6326
18	5A	AX-110147509	0.2164	131.6326
18	5A	AX-109434378	0.2164	131.6326
18	5A	AX-109510466	0.2164	131.6326
18	5A	AX-108849763	0.2164	131.6326
18	5A	AX-94837095	0.2164	131.6326
18	5A	AX-110012818	0.2164	131.6326

18	5A	AX-110644830	0.4184	132.051
18	5A	AX-108871400	0.4184	132.051
18	5A	AX-110508849	0.4184	132.051
18	5A	AX-110165981	0.4184	132.051
18	5A	AX-110504938	0.4184	132.051
18	5A	AX-110605582	0.4184	132.051
18	5A	AX-109328544	0.4184	132.051
18	5A	AX-109581579	0.4184	132.051
18	5A	AX-111615251	0.4184	132.051
18	5A	AX-111074385	0.4184	132.051
18	5A	AX-111469848	0.4184	132.051
18	5A	AX-110976471	0.4184	132.051
18	5A	AX-109431955	0.4184	132.051
18	5A	AX-111572088	0.4184	132.051
18	5A	AX-108775797	0.4184	132.051
18	5A	AX-110949372	0.4184	132.051
18	5A	AX-110557609	0.4184	132.051
18	5A	AX-111473332	0.4184	132.051
18	5A	AX-110457990	0.4184	132.051
18	5A	AX-108849758	0.4184	132.051
18	5A	AX-111487688	0.4184	132.051
18	5A	AX-109184753	0.4184	132.051
18	5A	AX-94726381	0.4184	132.051
18	5A	AX-110513367	0.4184	132.051
18	5A	AX-111207825	0.4184	132.051
18	5A	AX-94690469	0.4184	132.051
18	5A	AX-109289696	0.2083	132.2594
18	5A	AX-111505940	0.2083	132.2594
18	5A	AX-109402264	0.2083	132.2594
18	5A	AX-111548351	0.2083	132.2594
18	5A	AX-110477113	0.2083	132.2594
18	5A	AX-110939030	0.2083	132.2594
18	5A	AX-111107160	0.2083	132.2594
18	5A	AX-109384702	0.4202	132.6795
18	5A	AX-109628516	0.4202	132.6795
18	5A	AX-110642337	4.0814	136.761
18	5A	AX-111096820	4.0814	136.761
18	5A	AX-110976602	4.0814	136.761
18	5A	AX-111101296	4.0814	136.761
18	5A	AX-108760528	5.0866	141.8476
18	5A	AX-110199626	0.2075	142.055
18	5A	AX-108726875	0.2075	142.055
18	5A	AX-110996595	0.2075	142.055
18	5A	AX-108977827	0.625	142.6801

18	5A	AX-109463780	0.625	142.6801
18	5A	AX-110002364	0.2075	142.8875
18	5A	AX-109313646	0.6277	143.5152
18	5A	AX-111559378	0.6277	143.5152
18	5A	AX-109417217	0.6277	143.5152
18	5A	AX-109284112	0.6277	143.5152
18	5A	AX-108839521	0.6277	143.5152
18	5A	AX-110931690	0.6277	143.5152
18	5A	AX-110010456	0.6277	143.5152
18	5A	AX-111124959	0.6277	143.5152
18	5A	AX-110957187	0.2075	143.7227
18	5A	AX-109284590	0.2075	143.7227
18	5A	AX-108803879	0.2075	143.7227
18	5A	AX-109339422	0.2075	143.9301
18	5A	AX-110966247	0.2075	143.9301
18	5A	AX-108856756	0.2075	143.9301
18	5A	AX-110598597	0.2075	143.9301
18	5A	AX-110378212	0.2075	143.9301
18	5A	AX-110475485	0.2075	143.9301
18	5A	AX-108739016	0.2075	143.9301
18	5A	AX-111101411	0.2075	143.9301
18	5A	AX-112285824	0.2075	143.9301
18	5A	AX-110007191	0.2092	144.1394
18	5A	AX-111556070	1.7398	145.8792
18	5A	AX-110975496	1.7398	145.8792
18	5A	AX-108762346	1.7398	145.8792
18	5A	AX-108796702	1.7398	145.8792
18	5A	AX-110972066	0.2101	146.0893
18	5A	AX-109624254	0.422	146.5112
18	5A	AX-110422043	0.422	146.9332
18	5A	AX-109365992	0.2101	147.1433
18	5A	AX-109864964	0.2101	147.1433
18	5A	AX-109315829	0.2101	147.1433
18	5A	AX-110062429	0.6356	147.7789
18	5A	AX-110021952	0.6356	147.7789
18	5A	AX-95001743	0.6356	147.7789
18	5A	AX-111106109	1.2878	149.0667
18	5A	AX-111594503	1.2878	149.0667
18	5A	AX-111470543	1.2878	149.0667
18	5A	AX-110027672	9.0529	158.1196
18	5A	AX-111512110	2.3931	160.5127
18	5A	AX-111585657	2.3931	160.5127
18	5A	AX-111029506	2.3931	160.5127
18	5A	AX-89420656	2.3931	160.5127

18	5A	AX-109373034	2.3931	160.5127
18	5A	AX-109278524	0.4167	160.9294
18	5A	AX-111583791	0.4167	160.9294
18	5A	AX-109950944	0.4167	160.9294
18	5A	AX-109918926	1.4962	162.4256
18	5A	AX-111037319	1.4962	162.4256
18	5A	AX-109406000	1.4962	162.4256
18	5A	AX-111258952	1.4962	162.4256
18	5A	AX-110983833	1.4962	162.4256
18	5A	AX-110414471	1.4962	162.4256
18	5A	AX-109940222	1.4962	162.4256
18	5A	AX-108761796	1.4962	162.4256
18	5A	AX-111680823	1.4962	162.4256
18	5A	AX-109511993	1.4962	162.4256
18	5A	AX-108784774	1.4962	162.4256
18	5A	AX-110399181	1.4962	162.4256
18	5A	AX-108898706	1.4962	162.4256
18	5A	AX-110546458	1.4962	162.4256
18	5A	AX-110503841	1.4962	162.4256
18	5A	AX-109842825	1.4962	162.4256
18	5A	AX-109326350	1.4962	162.4256
18	5A	AX-111488342	0.2128	162.6384
18	5A	AX-108951190	0.4274	163.0657
18	5A	AX-108764806	0.2075	163.2732
18	5A	AX-109341558	0.2066	163.4798
18	5A	AX-110035703	0.2066	163.4798
18	5A	AX-108929871	0.2075	163.6873
18	5A	AX-109891142	3.1013	166.7886
18	5A	AX-109302500	0.4149	167.2036
18	5A	AX-110449161	4.2704	171.474
18	5A	AX-109316072	0.2092	171.6832
18	5A	AX-111496163	0.4184	172.1016
18	5A	AX-110647322	0.4184	172.1016
18	5A	AX-110936436	0.2058	172.3074
18	5A	AX-110060964	0.2058	172.3074
18	5A	AX-111190259	0.2058	172.3074
18	5A	AX-109964117	0.2058	172.3074
18	5A	AX-110581315	0.2058	172.3074
18	5A	AX-110703819	0.2058	172.3074
18	5A	AX-111097689	0.2058	172.3074
18	5A	AX-109273136	0.2058	172.3074
18	5A	AX-111034326	0.2058	172.3074
18	5A	AX-111573142	16.1284	188.4359
18	5A	AX-109451992	0.4099	188.8457

18	5A	AX-110533502	3.2942	192.1399
18	5A	AX-109349390	0.2083	192.3483
18	5A	AX-109547614	0.8404	193.1887
18	5A	AX-108801536	0.4132	193.6019
18	5A	AX-110449012	0.4132	193.6019
18	5A	AX-109277512	0.8334	194.4353
18	5A	AX-111038387	0.8334	194.4353
18	5A	AX-110712036	0.8334	194.4353
19	5B	AX-108924985	0	0
19	5B	AX-111081066	0	0
19	5B	AX-111572909	0	0
19	5B	AX-109456129	0	0
19	5B	AX-111806645	0	0
19	5B	AX-109272590	0.6276	0.6276
19	5B	AX-111453162	0.6276	0.6276
19	5B	AX-111055724	0.6276	0.6276
19	5B	AX-108750892	0.2075	0.8351
19	5B	AX-109476383	0.2075	0.8351
19	5B	AX-111104852	0.2075	0.8351
19	5B	AX-111611953	0.2075	0.8351
19	5B	AX-110144085	0.2075	1.0426
19	5B	AX-109463720	9.0991	10.1417
19	5B	AX-110042137	9.0991	10.1417
19	5B	AX-111627154	9.0991	10.1417
19	5B	AX-110050262	3.1861	13.3279
19	5B	AX-110433617	3.1861	13.3279
19	5B	AX-111584327	3.1861	13.3279
19	5B	AX-111560874	0.6551	13.9829
19	5B	AX-110002859	0.4348	14.4177
19	5B	AX-109650482	0.4348	14.4177
19	5B	AX-111465621	0.4348	14.4177
19	5B	AX-111730173	0.4348	14.4177
19	5B	AX-111745537	0.4348	14.4177
19	5B	AX-108876612	0.4348	14.4177
19	5B	AX-110941890	0.4348	14.4177
19	5B	AX-109277023	0.4348	14.4177
19	5B	AX-109818702	0.4348	14.4177
19	5B	AX-111482700	0.6494	15.0671
19	5B	AX-110364029	0.6494	15.0671
19	5B	AX-108941296	0.6494	15.0671
19	5B	AX-111607086	0.6494	15.0671
19	5B	AX-108769798	0.6494	15.0671
19	5B	AX-109366230	0.6494	15.0671
19	5B	AX-109823123	1.1015	16.1686

19	5B	AX-108867348	0.2155	16.3841
19	5B	AX-109648386	0.2155	16.3841
19	5B	AX-94821777	0.2155	16.3841
19	5B	AX-111536293	0.2203	16.6044
19	5B	AX-111029992	2.7936	19.398
19	5B	AX-109429755	2.7936	19.398
19	5B	AX-111599857	2.7936	19.398
19	5B	AX-111454545	2.7936	19.398
19	5B	AX-108749262	2.7936	19.398
19	5B	AX-109839576	2.7936	19.398
19	5B	AX-108726243	9.1459	28.5439
19	5B	AX-108736450	9.1459	28.5439
19	5B	AX-109431791	9.1459	28.5439
19	5B	AX-111481616	9.1459	28.5439
19	5B	AX-110164030	9.1459	28.5439
19	5B	AX-110004724	9.1459	28.5439
19	5B	AX-109077353	9.1459	28.5439
19	5B	AX-111023718	0.2128	28.7566
19	5B	AX-111699672	0.2137	28.9703
19	5B	AX-110934052	0.6608	29.6311
19	5B	AX-110412485	1.5771	31.2082
19	5B	AX-111100176	1.5771	31.2082
19	5B	AX-108851631	4.7087	35.9169
19	5B	AX-108791526	4.7087	35.9169
19	5B	AX-109518159	4.7087	35.9169
19	5B	AX-110675968	0.2155	36.1325
19	5B	AX-111490382	0.2155	36.1325
19	5B	AX-109856154	4.5146	40.6471
19	5B	AX-108769780	0.4237	41.0708
19	5B	AX-111466848	0.6411	41.7119
19	5B	AX-109817256	0.4255	42.1374
19	5B	AX-109981797	0.4255	42.1374
19	5B	AX-111011397	0.4255	42.1374
19	5B	AX-89463242	0.4255	42.1374
19	5B	AX-95683862	0.4255	42.1374
19	5B	AX-109839557	0.4237	42.5611
19	5B	AX-109339994	0.4237	42.5611
19	5B	AX-108922541	0.2119	42.773
19	5B	AX-111051748	0.2119	42.773
19	5B	AX-110549972	2.7176	45.4906
19	5B	AX-111143540	0.4274	45.918
19	5B	AX-109385815	1.5091	47.427
19	5B	AX-110384830	1.5091	47.427
19	5B	AX-108818304	1.5091	47.427

19	5B	AX-109900605	1.5091	47.427
19	5B	AX-109070804	1.5091	47.427
19	5B	AX-110931249	1.5091	47.427
19	5B	AX-110971857	1.5091	47.427
19	5B	AX-110141414	1.5091	47.427
19	5B	AX-109355449	1.5091	47.427
19	5B	AX-109421373	1.5091	47.427
19	5B	AX-110405101	1.5091	47.427
19	5B	AX-109278970	1.5091	47.427
19	5B	AX-109953350	1.5091	47.427
19	5B	AX-111689113	1.5091	47.427
19	5B	AX-110587485	1.5091	47.427
19	5B	AX-108754014	1.5091	47.427
19	5B	AX-111579272	1.5091	47.427
19	5B	AX-111761001	1.5091	47.427
19	5B	AX-110002288	1.5091	47.427
19	5B	AX-109900896	1.5091	47.427
19	5B	AX-109507546	0.4202	47.8472
19	5B	AX-110523128	0.4202	47.8472
19	5B	AX-109482700	0.4202	47.8472
19	5B	AX-110489640	0.4202	47.8472
19	5B	AX-110673377	0.4202	47.8472
19	5B	AX-110705974	0.4202	47.8472
19	5B	AX-109048125	0.4202	47.8472
19	5B	AX-110671243	0.4202	47.8472
19	5B	AX-108734168	0.4202	47.8472
19	5B	AX-110569067	0.4202	48.2674
19	5B	AX-108973476	0.4202	48.2674
19	5B	AX-108728394	0.4202	48.2674
19	5B	AX-109278051	0.4202	48.2674
19	5B	AX-110050584	0.4202	48.2674
19	5B	AX-108774582	0.4202	48.2674
19	5B	AX-109396083	0.4202	48.2674
19	5B	AX-108959845	0.4202	48.2674
19	5B	AX-108818768	0.4202	48.2674
19	5B	AX-109942729	0.4202	48.2674
19	5B	AX-110908996	0.4202	48.2674
19	5B	AX-111023017	0.4202	48.2674
19	5B	AX-110997284	0.4202	48.2674
19	5B	AX-111123197	0.4202	48.2674
19	5B	AX-109056229	0.4202	48.2674
19	5B	AX-108805572	0.4202	48.2674
19	5B	AX-109583119	0.4202	48.2674
19	5B	AX-109419514	0.4202	48.2674

19	5B	AX-109966081	0.4202	48.2674
19	5B	AX-108849200	0.4202	48.2674
19	5B	AX-108815271	0.4202	48.2674
19	5B	AX-111614446	0.4202	48.2674
19	5B	AX-110032089	0.4202	48.2674
19	5B	AX-111453141	0.4202	48.2674
19	5B	AX-110508540	0.4202	48.2674
19	5B	AX-108899316	0.4202	48.2674
19	5B	AX-109583864	0.4202	48.2674
19	5B	AX-108959579	0.4202	48.2674
19	5B	AX-110386578	0.4202	48.2674
19	5B	AX-108811852	0.4202	48.2674
19	5B	AX-110688395	0.4202	48.2674
19	5B	AX-111038457	0.4202	48.2674
19	5B	AX-109415750	0.4202	48.2674
19	5B	AX-109509772	0.4202	48.2674
19	5B	AX-108965399	0.4202	48.2674
19	5B	AX-109423529	0.4202	48.2674
19	5B	AX-111157487	0.4202	48.2674
19	5B	AX-110486498	0.4202	48.2674
19	5B	AX-109580745	0.4202	48.2674
19	5B	AX-111600286	0.4202	48.2674
19	5B	AX-111091643	0.4202	48.2674
19	5B	AX-111014483	0.4202	48.2674
19	5B	AX-109491777	0.4202	48.2674
19	5B	AX-111114646	0.4202	48.2674
19	5B	AX-108825923	0.4202	48.2674
19	5B	AX-109905918	0.4202	48.2674
19	5B	AX-108792794	0.4202	48.2674
19	5B	AX-108741022	0.4202	48.2674
19	5B	AX-111531033	0.4202	48.2674
19	5B	AX-109452679	0.4202	48.2674
19	5B	AX-109385122	0.4202	48.2674
19	5B	AX-109357056	0.4202	48.2674
19	5B	AX-109336757	0.4202	48.2674
19	5B	AX-109401436	0.4202	48.2674
19	5B	AX-110511353	0.4202	48.2674
19	5B	AX-111017571	0.4202	48.2674
19	5B	AX-110579133	0.4202	48.2674
19	5B	AX-109640888	0.4202	48.2674
19	5B	AX-111194981	0.4202	48.2674
19	5B	AX-111491815	0.4202	48.2674
19	5B	AX-110921633	0.4202	48.2674
19	5B	AX-111600319	0.4202	48.2674

19	5B	AX-110002756	0.4202	48.2674
19	5B	AX-108912482	0.4202	48.2674
19	5B	AX-111107749	0.4202	48.2674
19	5B	AX-110497947	0.4202	48.2674
19	5B	AX-111504551	0.4202	48.2674
19	5B	AX-111547195	0.4202	48.2674
19	5B	AX-111458311	0.4202	48.2674
19	5B	AX-108796034	0.4202	48.2674
19	5B	AX-110986746	0.4202	48.2674
19	5B	AX-110032721	0.4202	48.2674
19	5B	AX-111489947	0.4202	48.2674
19	5B	AX-110636907	0.4202	48.2674
19	5B	AX-109568488	0.4202	48.2674
19	5B	AX-111061195	0.4202	48.2674
19	5B	AX-110369643	0.4202	48.2674
19	5B	AX-109341336	0.4202	48.2674
19	5B	AX-110363637	0.4202	48.2674
19	5B	AX-111070012	0.4202	48.2674
19	5B	AX-111091404	0.4202	48.2674
19	5B	AX-109876647	0.4202	48.2674
19	5B	AX-109600924	0.4202	48.2674
19	5B	AX-108953143	0.4202	48.2674
19	5B	AX-108976093	0.4202	48.2674
19	5B	AX-111449487	0.4202	48.2674
19	5B	AX-111689338	0.4202	48.2674
19	5B	AX-110146396	0.4202	48.2674
19	5B	AX-111626784	0.4202	48.2674
19	5B	AX-108863198	0.4202	48.2674
19	5B	AX-110434226	0.4202	48.2674
19	5B	AX-110696432	0.4202	48.2674
19	5B	AX-109353096	0.4202	48.2674
19	5B	AX-110582074	0.4202	48.2674
19	5B	AX-109481992	0.4202	48.2674
19	5B	AX-109434112	0.4202	48.2674
19	5B	AX-110384843	0.4202	48.2674
19	5B	AX-110521602	0.4202	48.2674
19	5B	AX-109357419	0.4202	48.2674
19	5B	AX-110490543	0.4202	48.2674
19	5B	AX-111096992	0.4202	48.2674
19	5B	AX-109597973	0.4202	48.2674
19	5B	AX-110425180	0.4202	48.2674
19	5B	AX-109600416	0.4202	48.2674
19	5B	AX-111236158	0.4202	48.2674
19	5B	AX-111078255	0.4202	48.2674

19	5B	AX-108766891	0.4202	48.2674
19	5B	AX-108856405	0.4202	48.2674
19	5B	AX-108833699	0.4202	48.2674
19	5B	AX-110527633	0.4202	48.2674
19	5B	AX-110581283	0.4202	48.2674
19	5B	AX-111649592	0.4202	48.2674
19	5B	AX-111143503	0.4202	48.2674
19	5B	AX-110009409	0.4202	48.2674
19	5B	AX-108806688	0.4202	48.2674
19	5B	AX-110364887	0.4202	48.2674
19	5B	AX-108814866	0.4202	48.2674
19	5B	AX-109376969	0.4202	48.2674
19	5B	AX-110500435	0.4202	48.2674
19	5B	AX-111252462	0.4202	48.2674
19	5B	AX-110578028	0.4202	48.2674
19	5B	AX-110923531	0.4202	48.2674
19	5B	AX-109085274	0.4202	48.2674
19	5B	AX-110567912	0.4202	48.2674
19	5B	AX-111112569	0.4202	48.2674
19	5B	AX-109469134	0.4202	48.2674
19	5B	AX-111060117	0.4202	48.2674
19	5B	AX-110585414	0.4202	48.2674
19	5B	AX-109477141	0.4202	48.2674
19	5B	AX-111659492	0.4202	48.2674
19	5B	AX-111069778	0.4202	48.2674
19	5B	AX-108766237	0.4202	48.2674
19	5B	AX-111004963	0.4202	48.2674
19	5B	AX-110988886	0.4202	48.2674
19	5B	AX-109461978	0.4202	48.2674
19	5B	AX-111504181	0.4202	48.2674
19	5B	AX-110414818	0.4202	48.2674
19	5B	AX-108983046	0.4202	48.2674
19	5B	AX-108963974	0.4202	48.2674
19	5B	AX-109581594	0.4202	48.2674
19	5B	AX-109603392	0.4202	48.2674
19	5B	AX-109360057	0.4202	48.2674
19	5B	AX-108779962	0.4202	48.2674
19	5B	AX-111598126	0.4202	48.2674
19	5B	AX-108775382	0.4202	48.2674
19	5B	AX-110935192	0.4202	48.2674
19	5B	AX-109284215	0.4202	48.2674
19	5B	AX-109334323	0.4202	48.2674
19	5B	AX-109402291	0.4202	48.2674
19	5B	AX-109937011	0.4202	48.2674

19	5B	AX-109996126	0.4202	48.2674
19	5B	AX-110369626	0.4202	48.2674
19	5B	AX-109440609	0.4202	48.2674
19	5B	AX-109576954	0.4202	48.2674
19	5B	AX-110080289	0.4202	48.2674
19	5B	AX-110928366	0.4202	48.2674
19	5B	AX-95222832	0.4202	48.2674
19	5B	AX-108902352	0.8511	49.1185
19	5B	AX-111637148	0.8511	49.1185
19	5B	AX-109301146	0.8511	49.1185
19	5B	AX-110376463	0.2101	49.3286
19	5B	AX-110467281	0.2101	49.3286
19	5B	AX-109516452	0.2101	49.3286
19	5B	AX-108816702	0.6356	49.9643
19	5B	AX-111170310	0.422	50.3862
19	5B	AX-110065346	0.422	50.3862
19	5B	AX-111044944	0.422	50.3862
19	5B	AX-108735405	0.422	50.3862
19	5B	AX-110005277	0.422	50.3862
19	5B	AX-111608790	0.422	50.3862
19	5B	AX-110509199	0.422	50.3862
19	5B	AX-111465688	0.422	50.3862
19	5B	AX-109607704	0.422	50.3862
19	5B	AX-111670143	0.422	50.3862
19	5B	AX-111700499	0.2092	50.5954
19	5B	AX-109276765	0.2092	50.5954
19	5B	AX-108937690	0.2092	50.5954
19	5B	AX-109987113	0.2092	50.5954
19	5B	AX-94464288	0.2092	50.5954
19	5B	AX-108786292	0.2092	50.5954
19	5B	AX-109815720	0.2092	50.5954
19	5B	AX-111166958	0.8475	51.443
19	5B	AX-109481117	0.8622	52.3051
19	5B	AX-109532741	0.8622	52.3051
19	5B	AX-110546707	0.8622	52.3051
19	5B	AX-109330727	0.8622	52.3051
19	5B	AX-110071375	0.8622	52.3051
19	5B	AX-111107210	0.8622	52.3051
19	5B	AX-111260909	0.8622	52.3051
19	5B	AX-111587063	0.8622	52.3051
19	5B	AX-109961497	0.8622	52.3051
19	5B	AX-111105531	0.8622	52.3051
19	5B	AX-109302342	0.8622	52.3051
19	5B	AX-109062488	0.8622	52.3051

19	5B	AX-109516324	0.8622	52.3051
19	5B	AX-109585165	0.8622	52.3051
19	5B	AX-111040015	0.8622	52.3051
19	5B	AX-109470637	0.8622	52.3051
19	5B	AX-111649791	0.8622	52.3051
19	5B	AX-110020239	0.8622	52.3051
19	5B	AX-111552615	0.8622	52.3051
19	5B	AX-109961107	0.8622	52.3051
19	5B	AX-95187318	0.8622	52.3051
19	5B	AX-86172327	0.8622	52.3051
19	5B	AX-95657974	0.8622	52.3051
19	5B	AX-111008799	1.5222	53.8273
19	5B	AX-109483736	0.6356	54.463
19	5B	AX-108809180	0.6356	54.463
19	5B	AX-109008593	0.6356	54.463
19	5B	AX-110078184	0.6356	54.463
19	5B	AX-111761123	0.6356	54.463
19	5B	AX-111077770	0.6356	54.463
19	5B	AX-110956143	0.6356	54.463
19	5B	AX-111547026	0.6356	54.463
19	5B	AX-109000820	0.6356	54.463
19	5B	AX-110471566	0.6356	54.463
19	5B	AX-110907119	0.6356	54.463
19	5B	AX-109040704	0.6356	54.463
19	5B	AX-109897538	0.6356	54.463
19	5B	AX-109583014	0.6356	54.463
19	5B	AX-111218180	0.6356	54.463
19	5B	AX-111274399	0.6356	54.463
19	5B	AX-109564545	0.6356	54.463
19	5B	AX-110393750	0.6356	54.463
19	5B	AX-110175299	0.6356	54.463
19	5B	AX-110975676	0.6356	54.463
19	5B	AX-109893251	0.6356	54.463
19	5B	AX-111461209	0.6356	54.463
19	5B	AX-109364530	0.6356	54.463
19	5B	AX-110372119	0.6356	54.463
19	5B	AX-110003910	0.6356	54.463
19	5B	AX-109882611	0.6356	54.463
19	5B	AX-109936109	0.6356	54.463
19	5B	AX-111054273	0.6356	54.463
19	5B	AX-109847009	0.6356	54.463
19	5B	AX-108748929	0.6356	54.463
19	5B	AX-109553643	0.6356	54.463
19	5B	AX-111029789	0.6356	54.463

19	5B	AX-111069583	0.6356	54.463
19	5B	AX-108969498	0.6356	54.463
19	5B	AX-110490898	0.6356	54.463
19	5B	AX-108976225	0.6356	54.463
19	5B	AX-111181463	0.6356	54.463
19	5B	AX-111567348	0.6356	54.463
19	5B	AX-111697471	0.6356	54.463
19	5B	AX-110456475	0.6356	54.463
19	5B	AX-109378436	0.6356	54.463
19	5B	AX-109385238	0.6356	54.463
19	5B	AX-108876434	0.6356	54.463
19	5B	AX-110361811	0.6356	54.463
19	5B	AX-110520629	0.6356	54.463
19	5B	AX-109950063	0.6356	54.463
19	5B	AX-109416401	0.6356	54.463
19	5B	AX-111763500	0.6356	54.463
19	5B	AX-111055709	0.6356	54.463
19	5B	AX-110362123	0.6356	54.463
19	5B	AX-111451969	0.6356	54.463
19	5B	AX-108838982	0.6356	54.463
19	5B	AX-109942457	0.6356	54.463
19	5B	AX-109281809	0.6356	54.463
19	5B	AX-108885332	0.6356	54.463
19	5B	AX-109476209	0.6356	54.463
19	5B	AX-111139678	0.6356	54.463
19	5B	AX-110015742	0.6356	54.463
19	5B	AX-110968967	0.6356	54.463
19	5B	AX-111672195	0.6356	54.463
19	5B	AX-111062682	0.6356	54.463
19	5B	AX-110709849	0.6356	54.463
19	5B	AX-108744431	0.6356	54.463
19	5B	AX-110038652	0.6356	54.463
19	5B	AX-109353285	0.6356	54.463
19	5B	AX-108739778	0.6356	54.463
19	5B	AX-110744235	0.6356	54.463
19	5B	AX-110512516	0.6356	54.463
19	5B	AX-111498566	0.6356	54.463
19	5B	AX-108856056	0.6356	54.463
19	5B	AX-109512386	0.6356	54.463
19	5B	AX-111562114	0.6356	54.463
19	5B	AX-111602877	0.6356	54.463
19	5B	AX-110980299	0.6356	54.463
19	5B	AX-109421834	0.6356	54.463
19	5B	AX-111522327	0.6356	54.463

19	5B	AX-108890228	0.6356	54.463
19	5B	AX-110430683	0.6356	54.463
19	5B	AX-108843167	0.6356	54.463
19	5B	AX-111091433	0.6356	54.463
19	5B	AX-109915847	0.6356	54.463
19	5B	AX-111458358	0.6356	54.463
19	5B	AX-109413810	0.6356	54.463
19	5B	AX-109350487	0.6356	54.463
19	5B	AX-111580009	0.6356	54.463
19	5B	AX-110989689	0.6356	54.463
19	5B	AX-108780325	0.6356	54.463
19	5B	AX-110364562	0.6356	54.463
19	5B	AX-108747094	0.6356	54.463
19	5B	AX-109942290	0.6356	54.463
19	5B	AX-109859659	0.6356	54.463
19	5B	AX-110436510	0.6356	54.463
19	5B	AX-108888509	0.6356	54.463
19	5B	AX-108740462	0.6356	54.463
19	5B	AX-109418455	0.6356	54.463
19	5B	AX-110504123	0.6356	54.463
19	5B	AX-108776166	0.6356	54.463
19	5B	AX-108848982	0.6356	54.463
19	5B	AX-94529055	0.6356	54.463
19	5B	AX-110448034	0.6356	54.463
19	5B	AX-94426922	0.6356	54.463
19	5B	AX-94730020	0.6356	54.463
19	5B	AX-109323554	0.8511	55.3141
19	5B	AX-109312330	0.8511	55.3141
19	5B	AX-109057599	0.8511	55.3141
19	5B	AX-110630160	0.8511	55.3141
19	5B	AX-109524434	0.422	55.7361
19	5B	AX-110468936	0.422	55.7361
19	5B	AX-108741072	0.422	55.7361
19	5B	AX-111117922	0.422	55.7361
19	5B	AX-111529353	0.422	55.7361
19	5B	AX-109857907	0.422	55.7361
19	5B	AX-108890911	0.422	55.7361
19	5B	AX-109309705	0.422	55.7361
19	5B	AX-108760512	0.422	55.7361
19	5B	AX-108780883	0.422	55.7361
19	5B	AX-110523249	0.422	55.7361
19	5B	AX-111626188	0.422	55.7361
19	5B	AX-111722733	0.422	55.7361
19	5B	AX-110460968	0.422	55.7361

19	5B	AX-109351103	0.422	55.7361
19	5B	AX-109512214	0.422	55.7361
19	5B	AX-110400946	2.4464	58.1825
19	5B	AX-110460557	0.6411	58.8235
19	5B	AX-109939697	1.299	60.1225
19	5B	AX-94524014	1.299	60.1225
19	5B	AX-110562503	0.4237	60.5463
19	5B	AX-110531191	1.299	61.8452
19	5B	AX-110942939	2.4573	64.3026
19	5B	AX-108834390	1.3161	65.6187
19	5B	AX-111041175	1.3161	65.6187
19	5B	AX-108823103	1.3161	65.6187
19	5B	AX-111123056	1.3161	65.6187
19	5B	AX-111064457	1.3161	65.6187
19	5B	AX-111464585	1.3161	65.6187
19	5B	AX-110916580	1.3161	65.6187
19	5B	AX-108761083	1.3161	65.6187
19	5B	AX-111084700	1.3161	65.6187
19	5B	AX-109365562	1.3161	65.6187
19	5B	AX-108921794	1.3161	65.6187
19	5B	AX-109318862	1.3161	65.6187
19	5B	AX-110464931	1.3161	65.6187
19	5B	AX-110060253	1.3161	65.6187
19	5B	AX-111482689	1.3161	65.6187
19	5B	AX-110478907	1.3161	65.6187
19	5B	AX-108987268	1.3161	65.6187
19	5B	AX-108846370	1.3161	65.6187
19	5B	AX-110443905	1.3161	65.6187
19	5B	AX-111051645	1.3161	65.6187
19	5B	AX-111757415	1.3161	65.6187
19	5B	AX-111228330	1.3161	65.6187
19	5B	AX-109444078	1.3161	65.6187
19	5B	AX-109397635	1.3161	65.6187
19	5B	AX-111610542	1.3161	65.6187
19	5B	AX-111572956	1.3161	65.6187
19	5B	AX-108790507	1.3161	65.6187
19	5B	AX-111080778	1.3161	65.6187
19	5B	AX-110078350	1.3161	65.6187
19	5B	AX-95078562	1.3161	65.6187
19	5B	AX-108973826	0.4237	66.0424
19	5B	AX-111006113	0.4237	66.0424
19	5B	AX-109839495	0.4237	66.0424
19	5B	AX-109987949	0.4237	66.0424
19	5B	AX-109320330	0.4367	66.4791

19	5B	AX-110506915	1.3396	67.8187
19	5B	AX-108879462	1.3396	67.8187
19	5B	AX-111136480	1.3396	67.8187
19	5B	AX-111067739	1.5091	69.3278
19	5B	AX-110435660	1.5091	69.3278
19	5B	AX-111052445	1.5091	69.3278
19	5B	AX-109879639	1.5091	69.3278
19	5B	AX-110945112	0.2119	69.5397
19	5B	AX-109941486	0.2119	69.5397
19	5B	AX-110927083	0.2119	69.7515
19	5B	AX-111714815	0.2119	69.7515
19	5B	AX-108949983	0.2119	69.7515
19	5B	AX-111653389	0.2119	69.7515
19	5B	AX-109954848	0.4184	70.1699
19	5B	AX-108822759	0.4184	70.1699
19	5B	AX-108915482	0.4184	70.1699
19	5B	AX-110401611	0.4184	70.1699
19	5B	AX-111214466	0.4184	70.1699
19	5B	AX-108800607	0.4184	70.1699
19	5B	AX-111603943	0.4184	70.1699
19	5B	AX-110675247	0.4184	70.1699
19	5B	AX-109430858	0.4184	70.1699
19	5B	AX-111608383	0.4184	70.1699
19	5B	AX-109567220	0.4184	70.1699
19	5B	AX-109482115	0.4184	70.1699
19	5B	AX-110032100	0.4184	70.1699
19	5B	AX-111627941	0.4184	70.1699
19	5B	AX-109881631	0.4184	70.1699
19	5B	AX-111495996	0.4184	70.1699
19	5B	AX-111109056	0.4184	70.1699
19	5B	AX-111108344	0.4184	70.1699
19	5B	AX-111670839	0.4184	70.1699
19	5B	AX-110926265	0.4184	70.1699
19	5B	AX-110042200	0.4184	70.1699
19	5B	AX-110447570	0.4184	70.1699
19	5B	AX-111582953	0.4184	70.1699
19	5B	AX-108885871	0.4184	70.1699
19	5B	AX-108914468	0.4184	70.1699
19	5B	AX-94409094	0.4184	70.1699
19	5B	AX-110412002	0.4184	70.1699
19	5B	AX-110439529	0.4184	70.1699
19	5B	AX-111479573	0.4184	70.1699
19	5B	AX-111495304	0.2083	70.3783
19	5B	AX-111760682	0.4167	70.795

19	5B	AX-110511820	0.4167	70.795
19	5B	AX-111556818	0.4167	70.795
19	5B	AX-110408370	0.4167	70.795
19	5B	AX-108779200	0.4167	70.795
19	5B	AX-111066148	0.4167	70.795
19	5B	AX-111476338	0.4167	70.795
19	5B	AX-109448182	0.4167	70.795
19	5B	AX-111001988	0.4167	70.795
19	5B	AX-108729706	0.4167	70.795
19	5B	AX-110397910	0.4167	70.795
19	5B	AX-111454412	0.4167	70.795
19	5B	AX-95192291	0.4167	70.795
19	5B	AX-110482128	0.2092	71.0042
19	5B	AX-109294554	0.4202	71.4243
19	5B	AX-109617127	0.4202	71.4243
19	5B	AX-110469146	0.4202	71.4243
19	5B	AX-110363233	0.2083	71.6327
19	5B	AX-110532317	0.211	71.8437
19	5B	AX-110588422	0.211	71.8437
19	5B	AX-108886889	1.0778	72.9214
19	5B	AX-110433913	1.0778	72.9214
19	5B	AX-109307624	1.0778	72.9214
19	5B	AX-110502417	1.0778	72.9214
19	5B	AX-109842839	0.6356	73.557
19	5B	AX-109449543	0.4184	73.9754
19	5B	AX-110615540	0.4184	73.9754
19	5B	AX-111002136	0.2092	74.1847
19	5B	AX-111039023	0.2092	74.1847
19	5B	AX-108756763	0.2092	74.1847
19	5B	AX-109819102	0.4237	74.6084
19	5B	AX-110450012	0.4237	74.6084
19	5B	AX-108961268	0.4237	74.6084
19	5B	AX-109997727	0.4237	74.6084
19	5B	AX-109500107	0.4237	74.6084
19	5B	AX-109475499	0.4237	74.6084
19	5B	AX-109526372	0.4237	74.6084
19	5B	AX-110085797	0.4237	74.6084
19	5B	AX-111574616	0.4237	74.6084
19	5B	AX-110093097	0.4237	74.6084
19	5B	AX-110382685	0.4237	74.6084
19	5B	AX-110029474	0.4237	74.6084
19	5B	AX-108737496	0.4237	74.6084
19	5B	AX-109549994	0.4237	74.6084
19	5B	AX-109891271	0.4237	74.6084

19	5B	AX-109035690	0.4237	74.6084
19	5B	AX-111599205	0.4237	74.6084
19	5B	AX-108782003	0.4237	74.6084
19	5B	AX-110423840	0.4237	74.6084
19	5B	AX-110999405	0.4237	74.6084
19	5B	AX-108752448	0.4237	74.6084
19	5B	AX-109914005	0.4237	74.6084
19	5B	AX-108855043	0.4237	74.6084
19	5B	AX-109903615	0.4237	74.6084
19	5B	AX-111181093	0.4237	74.6084
19	5B	AX-109400878	0.4237	74.6084
19	5B	AX-111098527	0.4237	74.6084
19	5B	AX-94466025	0.4237	74.6084
19	5B	AX-110406590	0.4237	74.6084
19	5B	AX-110012505	0.4237	74.6084
19	5B	AX-111712445	0.4237	74.6084
19	5B	AX-109411601	0.4237	74.6084
19	5B	AX-109556435	0.4237	74.6084
19	5B	AX-111080316	0.4237	74.6084
19	5B	AX-110958685	0.4237	74.6084
19	5B	AX-109950997	0.4237	74.6084
19	5B	AX-108954834	0.4237	74.6084
19	5B	AX-111596267	0.4237	74.6084
19	5B	AX-108943772	0.4237	74.6084
19	5B	AX-109506288	0.4237	74.6084
19	5B	AX-109286283	0.4237	74.6084
19	5B	AX-109348971	0.4237	74.6084
19	5B	AX-109871112	0.4237	74.6084
19	5B	AX-110439426	0.4237	74.6084
19	5B	AX-109284620	0.4237	74.6084
19	5B	AX-108766482	0.4237	74.6084
19	5B	AX-108897042	0.4237	74.6084
19	5B	AX-110585070	0.4237	74.6084
19	5B	AX-111077103	0.4237	74.6084
19	5B	AX-111126578	0.4237	74.6084
19	5B	AX-111180110	0.4237	74.6084
19	5B	AX-109822039	0.4237	74.6084
19	5B	AX-94937534	0.4237	74.6084
19	5B	AX-108781821	0.4237	74.6084
19	5B	AX-95144641	0.4237	74.6084
19	5B	AX-112290306	0.4237	74.6084
19	5B	AX-111011634	0.4237	74.6084
19	5B	AX-95172465	0.4237	74.6084
19	5B	AX-109724542	0.4237	74.6084

19	5B	AX-110161029	0.4237	74.6084
19	5B	AX-94476971	0.4237	74.6084
19	5B	AX-109445467	0.4237	74.6084
19	5B	AX-109526449	0.4237	74.6084
19	5B	AX-111170707	0.4237	74.6084
19	5B	AX-109888874	0.4237	74.6084
19	5B	AX-109867790	0.4237	74.6084
19	5B	AX-95073334	0.4237	74.6084
19	5B	AX-111094521	0.4237	74.6084
19	5B	AX-108978441	0.4237	74.6084
19	5B	AX-109336014	0.4237	74.6084
19	5B	AX-110628300	0.4237	74.6084
19	5B	AX-95022681	0.4237	74.6084
19	5B	AX-109527199	0.4237	74.6084
19	5B	AX-111035187	0.4237	74.6084
19	5B	AX-108764886	0.4237	74.6084
19	5B	AX-94537482	0.4237	74.6084
19	5B	AX-94439861	0.4237	74.6084
19	5B	AX-94707318	0.4237	74.6084
19	5B	AX-111707113	0.4237	74.6084
19	5B	AX-111519040	0.4237	74.6084
19	5B	AX-110053257	0.4237	74.6084
19	5B	AX-111641548	0.4237	74.6084
19	5B	AX-110616233	0.4237	74.6084
19	5B	AX-110558030	0.4237	74.6084
19	5B	AX-108787594	0.4237	74.6084
19	5B	AX-111061756	0.4237	74.6084
19	5B	AX-110387496	0.4237	74.6084
19	5B	AX-110630741	0.4237	74.6084
19	5B	AX-110620972	0.4237	74.6084
19	5B	AX-110535151	0.4237	74.6084
19	5B	AX-110951278	0.4237	74.6084
19	5B	AX-108831539	0.4237	74.6084
19	5B	AX-110324477	0.4237	74.6084
19	5B	AX-108865212	0.4237	74.6084
19	5B	AX-109278592	0.4237	74.6084
19	5B	AX-109394277	0.4237	74.6084
19	5B	AX-109418971	0.4237	74.6084
19	5B	AX-109819042	0.4237	74.6084
19	5B	AX-109883144	0.4237	74.6084
19	5B	AX-110097743	0.4237	74.6084
19	5B	AX-110285872	0.4237	74.6084
19	5B	AX-110394652	0.4237	74.6084
19	5B	AX-110423321	0.4237	74.6084

19	5B	AX-110576699	0.4237	74.6084
19	5B	AX-110614136	0.4237	74.6084
19	5B	AX-110614450	0.4237	74.6084
19	5B	AX-111106187	0.4237	74.6084
19	5B	AX-111451774	0.4237	74.6084
19	5B	AX-111730624	0.4237	74.6084
19	5B	AX-94412398	0.4237	74.6084
19	5B	AX-94440933	0.4237	74.6084
19	5B	AX-94451691	0.4237	74.6084
19	5B	AX-94558969	0.4237	74.6084
19	5B	AX-94606276	0.4237	74.6084
19	5B	AX-94643687	0.4237	74.6084
19	5B	AX-94826759	0.4237	74.6084
19	5B	AX-95683717	0.4237	74.6084
19	5B	AX-111711727	0.4255	75.0339
19	5B	AX-110984257	0.6383	75.6723
19	5B	AX-108781101	0.6383	75.6723
19	5B	AX-109530360	0.6383	75.6723
19	5B	AX-109513743	0.6383	75.6723
19	5B	AX-109184312	0.6383	75.6723
19	5B	AX-109423081	0.2092	75.8815
19	5B	AX-110550049	0.2092	75.8815
19	5B	AX-110413197	0.2092	75.8815
19	5B	AX-110447436	0.2092	75.8815
19	5B	AX-95209196	0.2092	75.8815
19	5B	AX-111000973	0.4184	76.2999
19	5B	AX-111132820	0.4184	76.2999
19	5B	AX-108976112	0.4184	76.2999
19	5B	AX-109907067	0.4184	76.2999
19	5B	AX-94563546	0.4184	76.2999
19	5B	AX-109482618	0.2083	76.5082
19	5B	AX-110948117	0.2083	76.5082
19	5B	AX-110058364	0.2083	76.5082
19	5B	AX-110656501	0.2083	76.5082
19	5B	AX-110520632	0.2083	76.5082
19	5B	AX-111576625	1.2878	77.7961
19	5B	AX-111472988	0.8659	78.6619
19	5B	AX-110921023	0.2128	78.8747
19	5B	AX-109429484	0.8512	79.7259
19	5B	AX-111002152	0.8512	79.7259
19	5B	AX-110593685	0.8512	79.7259
19	5B	AX-109978780	0.8512	79.7259
19	5B	AX-109396254	0.8512	79.7259
19	5B	AX-109380591	0.8512	79.7259

19	5B	AX-109907389	0.8512	79.7259
19	5B	AX-110130968	0.8512	79.7259
19	5B	AX-111687279	0.8512	79.7259
19	5B	AX-110686573	1.7398	81.4657
19	5B	AX-110954915	0.2128	81.6785
19	5B	AX-109845629	1.299	82.9775
19	5B	AX-108814349	0.2119	83.1893
19	5B	AX-110502620	1.9747	85.164
19	5B	AX-109068105	0.844	86.008
19	5B	AX-111123352	0.844	86.008
19	5B	AX-111484893	0.844	86.008
19	5B	AX-109824044	0.844	86.008
19	5B	AX-110952567	3.3683	89.3763
19	5B	AX-110483449	0.6411	90.0174
19	5B	AX-110388049	0.6411	90.0174
19	5B	AX-108976763	0.6411	90.0174
19	5B	AX-110403012	0.6411	90.0174
19	5B	AX-109528195	0.6411	90.0174
19	5B	AX-110931211	0.6411	90.0174
19	5B	AX-111530787	0.6411	90.0174
19	5B	AX-111457500	0.6411	90.0174
19	5B	AX-110406892	0.2128	90.2302
19	5B	AX-108805734	0.6438	90.874
19	5B	AX-111692057	0.6438	90.874
19	5B	AX-110451519	0.6438	90.874
19	5B	AX-110363571	0.6438	90.874
19	5B	AX-111104649	0.6438	90.874
19	5B	AX-111507926	0.6438	90.874
19	5B	AX-108870907	0.6438	90.874
19	5B	AX-109936477	0.6438	90.874
19	5B	AX-111051502	0.4274	91.3013
19	5B	AX-110734952	0.4274	91.3013
19	5B	AX-109983060	0.2128	91.5141
19	5B	AX-111761048	0.4274	91.9415
19	5B	AX-109358689	1.5423	93.4838
19	5B	AX-108829846	1.5423	93.4838
19	5B	AX-111662281	0.2174	93.7012
19	5B	AX-108961580	0.4425	94.1437
19	5B	AX-110405868	0.2193	94.363
19	5B	AX-109972990	0.2193	94.363
19	5B	AX-111691106	0.2193	94.363
19	5B	AX-108822520	0.2183	94.5813
19	5B	AX-109568059	0.2193	94.8006
19	5B	AX-109847562	0.2193	94.8006

19	5B	AX-111543232	0.2193	94.8006
19	5B	AX-109455413	0.2193	94.8006
19	5B	AX-109333543	0.2193	94.8006
19	5B	AX-108835880	0.2193	95.0199
19	5B	AX-111097453	0.2183	95.2383
19	5B	AX-111709741	0.2183	95.2383
19	5B	AX-108773010	0.2183	95.2383
19	5B	AX-108727517	0.2183	95.2383
19	5B	AX-110588581	0.2183	95.2383
19	5B	AX-110676363	0.2183	95.2383
19	5B	AX-110202071	0.2183	95.2383
19	5B	AX-111566304	0.2183	95.4566
19	5B	AX-109038008	0.6551	96.1117
19	5B	AX-108831560	0.6551	96.1117
19	5B	AX-110562555	1.9922	98.1039
19	5B	AX-109820694	1.9922	98.1039
19	5B	AX-110989649	1.9922	98.1039
19	5B	AX-109055754	1.9922	98.1039
19	5B	AX-109336932	1.9922	98.1039
19	5B	AX-110667533	1.9922	98.1039
19	5B	AX-108879525	1.9922	98.1039
19	5B	AX-108807464	1.9922	98.1039
19	5B	AX-109347431	1.9922	98.1039
19	5B	AX-109446557	1.9922	98.1039
19	5B	AX-108897432	1.9922	98.1039
19	5B	AX-109443194	1.9922	98.1039
19	5B	AX-111055718	1.9922	98.1039
19	5B	AX-110997009	1.9922	98.1039
19	5B	AX-110033612	1.9922	98.1039
19	5B	AX-108898461	1.9922	98.1039
19	5B	AX-110988901	1.9922	98.1039
19	5B	AX-109885005	1.9922	98.1039
19	5B	AX-111578942	1.9922	98.1039
19	5B	AX-111617807	1.9922	98.1039
19	5B	AX-111001969	1.9922	98.1039
19	5B	AX-110127021	1.9922	98.1039
19	5B	AX-111116004	1.9922	98.1039
19	5B	AX-109334161	1.9922	98.1039
19	5B	AX-109448123	1.9922	98.1039
19	5B	AX-111651764	1.9922	98.1039
19	5B	AX-109382206	1.9922	98.1039
19	5B	AX-108837826	1.9922	98.1039
19	5B	AX-110947245	1.9922	98.1039
19	5B	AX-109006558	1.9922	98.1039

19	5B	AX-111686529	1.9922	98.1039
19	5B	AX-109468483	1.9922	98.1039
19	5B	AX-109478383	1.9922	98.1039
19	5B	AX-110941899	1.9922	98.1039
19	5B	AX-95629018	1.9922	98.1039
19	5B	AX-95631528	1.9922	98.1039
19	5B	AX-111755548	1.9922	98.1039
19	5B	AX-111001298	0.2146	98.3185
19	5B	AX-111685060	0.6438	98.9623
19	5B	AX-108738376	0.6438	98.9623
19	5B	AX-109816274	0.6438	98.9623
19	5B	AX-94547820	0.6411	99.6033
19	5B	AX-110384536	0.6411	99.6033
19	5B	AX-110691328	0.6411	99.6033
19	5B	AX-109406062	0.6411	99.6033
19	5B	AX-110600267	0.4292	100.0325
19	5B	AX-108743255	0.4292	100.0325
19	5B	AX-110409747	0.4292	100.0325
19	5B	AX-109941615	0.4292	100.0325
19	5B	AX-109996563	0.6494	100.6819
19	5B	AX-109276428	0.6494	100.6819
19	5B	AX-110952221	0.6494	100.6819
19	5B	AX-110961703	2.2138	102.8958
19	5B	AX-109282927	2.2138	102.8958
19	5B	AX-110490429	0.6411	103.5368
19	5B	AX-109526597	1.3103	104.8472
19	5B	AX-108812926	0.6494	105.4966
19	5B	AX-109895549	4.9224	110.4189
19	5B	AX-108872409	4.9224	110.4189
19	5B	AX-108857263	4.9224	110.4189
19	5B	AX-110402737	0.885	111.304
19	5B	AX-111576443	0.6551	111.9591
19	5B	AX-109951695	0.4237	112.3828
19	5B	AX-111152749	0.422	112.8047
19	5B	AX-109349709	0.6303	113.435
19	5B	AX-109559173	0.4202	113.8552
19	5B	AX-109900321	0.4445	114.2997
19	5B	AX-110370626	0.2222	114.5219
19	5B	AX-109976424	0.4255	114.9474
19	5B	AX-109853609	0.6383	115.5857
19	5B	AX-110480025	3.5936	119.1794
19	5B	AX-110464594	0.4167	119.596
19	5B	AX-111473825	0.4167	119.596
19	5B	AX-109601421	0.4167	119.596

19	5B	AX-111065169	0.4167	119.596
19	5B	AX-110508006	0.4167	119.596
19	5B	AX-94964759	0.4167	119.596
19	5B	AX-109321892	0.4149	120.011
19	5B	AX-108782835	0.4149	120.011
19	5B	AX-111103257	0.4149	120.011
19	5B	AX-110423832	0.4149	120.011
19	5B	AX-111551279	0.2075	120.2185
19	5B	AX-109997800	0.4167	120.6351
19	5B	AX-108880962	0.4167	120.6351
19	5B	AX-111715324	0.4167	120.6351
19	5B	AX-110913121	0.4167	120.6351
19	5B	AX-110914955	0.4167	120.6351
19	5B	AX-111045951	0.4167	120.6351
19	5B	AX-111090273	0.4167	120.6351
19	5B	AX-94670687	0.4167	120.6351
19	5B	AX-110167647	0.4167	120.6351
19	5B	AX-94472448	0.4167	120.6351
19	5B	AX-95118628	0.4167	120.6351
19	5B	AX-110951480	0.4149	121.0501
19	5B	AX-109977810	0.4149	121.0501
19	5B	AX-110379138	0.4149	121.0501
19	5B	AX-111489106	0.4149	121.0501
19	5B	AX-109939306	0.4149	121.0501
19	5B	AX-111658687	0.4149	121.0501
19	5B	AX-94724456	0.4149	121.0501
19	5B	AX-110404510	1.7028	122.7529
19	5B	AX-111201355	1.7028	122.7529
19	5B	AX-110102298	0.2119	122.9648
19	5B	AX-110554992	36.4438	159.4086
19	5B	AX-109460153	0.4202	159.8288
19	5B	AX-109834857	0.4202	159.8288
19	5B	AX-109827342	0.8475	160.6763
19	5B	AX-111471893	0.8475	161.5238
19	5B	AX-110129017	0.8475	161.5238
19	5B	AX-111582060	0.8475	161.5238
19	5B	AX-111024571	0.8475	161.5238
19	5B	AX-110640726	0.8475	161.5238
19	5B	AX-110953444	1.4962	163.02
19	5B	AX-111664059	1.4962	163.02
19	5B	AX-111579934	1.4962	163.02
19	5B	AX-111198868	1.4962	163.02
19	5B	AX-108936241	1.0685	164.0885
19	5B	AX-109001427	0.211	164.2995

19	5B	AX-110127068	0.2119	164.5114
19	5B	AX-110673021	0.2119	164.5114
19	5B	AX-108930776	0.2092	164.7206
19	5B	AX-110525041	0.2083	164.9289
19	5B	AX-108922450	0.2083	164.9289
19	5B	AX-108863693	0.2083	164.9289
19	5B	AX-109651702	0.2083	164.9289
19	5B	AX-110482984	0.2092	165.1381
19	5B	AX-111462836	0.8584	165.9966
19	5B	AX-109354765	5.9521	171.9487
19	5B	AX-111143003	5.9521	171.9487
19	5B	AX-110045455	1.5026	173.4513
19	5B	AX-111514083	1.5026	173.4513
19	5B	AX-109520682	0.2083	173.6596
19	5B	AX-110439607	0.8659	174.5255
19	5B	AX-109506439	1.5222	176.0477
19	5B	AX-110128751	1.5222	176.0477
19	5B	AX-111610585	1.5222	176.0477
19	5B	AX-109585386	1.5222	176.0477
19	5B	AX-109863463	1.5222	176.0477
19	5B	AX-109281175	1.5222	176.0477
19	5B	AX-110370454	4.0265	180.0743
19	5B	AX-109921910	4.0265	180.0743
19	5B	AX-108750107	0.2075	180.2818
19	5B	AX-109479506	7.8692	188.151
19	5B	AX-111635620	0.4167	188.5676
19	5B	AX-111711490	0.4167	188.5676
19	5B	AX-109386760	0.4167	188.5676
19	5B	AX-95684549	0.4167	188.5676
19	5B	AX-109954999	0.2075	188.7751
19	5B	AX-108963243	0.2075	188.7751
19	5B	AX-108776572	0.2083	188.9834
19	5B	AX-108757760	0.6303	189.6137
19	5B	AX-108733256	0.6303	189.6137
19	5B	AX-111688292	0.6303	189.6137
19	5B	AX-110924699	0.6303	189.6137
19	5B	AX-110946146	0.6303	189.6137
19	5B	AX-110686186	0.2092	189.823
19	5B	AX-109826149	0.2092	189.823
19	5B	AX-109327169	0.2092	189.823
19	5B	AX-108895416	0.422	190.2449
19	5B	AX-109479931	0.2146	190.4595
19	5B	AX-109865357	6.6238	197.0833
19	5B	AX-111015752	6.6238	197.0833

19	5B	AX-108855042	6.6238	197.0833
19	5B	AX-111074180	9.0991	206.1825
19	5B	AX-110946148	2.6692	208.8517
19	5B	AX-109972054	0.2083	209.06
19	5B	AX-109270022	0.2101	209.2701
20	5D	AX-110565536	0	0
20	5D	AX-89633041	15.3849	15.3849
20	5D	AX-89489968	13.4145	28.7994
20	5D	AX-111917292	6.2224	35.0218
20	5D	AX-89752452	2.6692	37.691
20	5D	AX-111193198	6.4334	44.1244
20	5D	AX-89640152	0.4202	44.5446
20	5D	AX-109431959	0.4202	44.5446
20	5D	AX-110050695	0.2101	44.7547
20	5D	AX-110024506	0.2101	44.7547
20	5D	AX-111597176	0.2101	44.7547
20	5D	AX-108879232	0.2101	44.7547
20	5D	AX-111876811	0.2101	44.7547
20	5D	AX-109569467	0.2101	44.7547
20	5D	AX-110603986	0.2101	44.7547
20	5D	AX-111718644	0.2101	44.7547
20	5D	AX-108762744	0.2101	44.7547
20	5D	AX-108749884	0.2101	44.7547
20	5D	AX-109750115	0.2101	44.7547
20	5D	AX-111455654	0.2101	44.7547
20	5D	AX-109943729	0.2101	44.7547
20	5D	AX-109051666	0.2101	44.7547
20	5D	AX-111160467	0.2101	44.7547
20	5D	AX-111655632	0.2101	44.7547
20	5D	AX-111289304	0.2101	44.7547
20	5D	AX-86185855	0.2101	44.7547
20	5D	AX-111261471	0.2101	44.7547
20	5D	AX-110677922	0.2101	44.7547
20	5D	AX-111072780	0.2101	44.7547
20	5D	AX-108941261	0.2101	44.7547
20	5D	AX-111032486	0.2101	44.7547
20	5D	AX-111488807	0.2101	44.7547
20	5D	AX-111533201	0.2101	44.7547
20	5D	AX-109904168	0.2101	44.7547
20	5D	AX-89420236	0.2101	44.7547
20	5D	AX-108765683	0.2101	44.7547
20	5D	AX-109714016	0.2101	44.7547
20	5D	AX-111980450	0.2101	44.7547
20	5D	AX-111332622	0.2101	44.7547

20	5D	AX-109924593	0.2101	44.7547
20	5D	AX-111299554	0.2101	44.7547
20	5D	AX-111802962	0.2101	44.7547
20	5D	AX-110497303	0.2101	44.7547
20	5D	AX-110717039	0.2101	44.7547
20	5D	AX-109323735	0.2101	44.7547
20	5D	AX-108801222	0.2101	44.7547
20	5D	AX-110997063	0.2101	44.7547
20	5D	AX-111390298	0.2101	44.7547
20	5D	AX-110172785	0.2101	44.7547
20	5D	AX-110613125	0.2101	44.7547
20	5D	AX-111906637	0.2101	44.7547
20	5D	AX-111508579	0.2101	44.7547
20	5D	AX-111833356	0.2101	44.7547
20	5D	AX-109809884	0.2101	44.7547
20	5D	AX-111313972	0.2101	44.7547
20	5D	AX-108776047	0.2101	44.7547
20	5D	AX-111465868	0.422	45.1766
20	5D	AX-110899362	0.2101	45.3867
20	5D	AX-109878225	0.2101	45.3867
20	5D	AX-109541444	0.2101	45.3867
20	5D	AX-109243867	0.2101	45.3867
20	5D	AX-110132720	0.2101	45.3867
20	5D	AX-109307483	0.2101	45.3867
20	5D	AX-108773257	0.2101	45.3867
20	5D	AX-109428691	0.2137	45.6004
20	5D	AX-109492086	0.4329	46.0333
20	5D	AX-111906978	0.2092	46.2425
20	5D	AX-110121727	0.2092	46.2425
20	5D	AX-109477622	0.2092	46.2425
20	5D	AX-111481432	0.2092	46.2425
20	5D	AX-111520160	0.2092	46.2425
20	5D	AX-110575891	0.2092	46.2425
20	5D	AX-111519825	0.2092	46.2425
20	5D	AX-89438182	0.2092	46.2425
20	5D	AX-111003547	0.2092	46.2425
20	5D	AX-109164517	0.2083	46.4508
20	5D	AX-111643347	0.2083	46.4508
20	5D	AX-111577847	0.2083	46.4508
20	5D	AX-95658716	0.2083	46.4508
20	5D	AX-109544160	0.6303	47.0811
20	5D	AX-110271557	0.6303	47.0811
20	5D	AX-109915792	0.6303	47.0811
20	5D	AX-109418073	0.6303	47.0811

20	5D	AX-110550596	0.2075	47.2886
20	5D	AX-110460040	0.2075	47.2886
20	5D	AX-111912158	0.4167	47.7053
20	5D	AX-108820694	0.4167	47.7053
20	5D	AX-111137392	0.4167	47.7053
20	5D	AX-109629542	0.4167	47.7053
20	5D	AX-108737933	0.4167	47.7053
20	5D	AX-111496275	0.844	48.5492
20	5D	AX-111752399	0.2092	48.7584
20	5D	AX-109373971	0.2092	48.7584
20	5D	AX-110642398	0.2092	48.7584
20	5D	AX-89688493	0.6303	49.3887
20	5D	AX-110186027	0.6303	49.3887
20	5D	AX-109586392	0.6303	49.3887
20	5D	AX-110035446	0.2075	49.5962
20	5D	AX-89591395	0.2075	49.5962
20	5D	AX-111260934	0.2075	49.5962
20	5D	AX-110758473	0.2075	49.5962
20	5D	AX-110039178	1.055	50.6512
20	5D	AX-109900028	1.055	50.6512
20	5D	AX-111638065	1.055	50.6512
20	5D	AX-110793386	0.2075	50.8587
20	5D	AX-110041896	1.7398	52.5985
20	5D	AX-109838452	0.6438	53.2423
20	5D	AX-109380070	1.7323	54.9746
20	5D	AX-111540030	0.2092	55.1839
20	5D	AX-89642798	1.7323	56.9162
20	5D	AX-86178163	4.558	61.4742
20	5D	AX-110676362	1.5289	63.0031
20	5D	AX-108877411	1.3277	64.3308
20	5D	AX-108740467	0.4202	64.751
20	5D	AX-110772137	0.4202	64.751
20	5D	AX-109401717	1.0595	65.8105
20	5D	AX-108947746	2.634	68.4445
20	5D	AX-109929775	2.01	70.4545
20	5D	AX-110147986	5.2827	75.7372
20	5D	AX-109447373	1.5356	77.2728
20	5D	AX-109006940	3.0699	80.3427
20	5D	AX-111340395	10.355	90.6976
20	5D	AX-110570350	10.355	90.6976
20	5D	AX-109897023	1.2661	91.9637
20	5D	AX-109947280	1.2661	91.9637
20	5D	AX-89369050	9.8261	101.7898
20	5D	AX-109871258	8.0003	109.7901

20	5D	AX-109272147	8.0003	109.7901
20	5D	AX-89331886	3.034	112.8241
20	5D	AX-108974123	12.6333	125.4574
20	5D	AX-108760983	15.3276	140.785
20	5D	AX-110409786	0.8369	141.6219
20	5D	AX-94969919	24.7282	166.3501
20	5D	AX-109923560	3.5616	169.9117
20	5D	AX-110468871	0.2075	170.1191
20	5D	AX-89417887	0.2075	170.1191
20	5D	AX-111022479	17.0168	187.136
20	5D	AX-89451602	6.6426	193.7785
20	5D	AX-111544605	2.6225	196.401
20	5D	AX-108822269	0.2092	196.6102
20	5D	AX-111325396	1.4962	198.1064
20	5D	AX-110638920	1.4962	198.1064
20	5D	AX-111048593	1.9077	200.0141
20	5D	AX-109987937	1.9077	200.0141
20	5D	AX-109160499	1.9077	200.0141
20	5D	AX-110476682	1.9077	200.0141
20	5D	AX-109978421	1.9077	200.0141
20	5D	AX-108963458	1.9077	200.0141
20	5D	AX-110058454	1.9077	200.0141
20	5D	AX-109887356	1.9077	200.0141
20	5D	AX-109942010	1.9077	200.0141
20	5D	AX-94827316	1.9077	200.0141
20	5D	AX-109386369	1.9077	200.0141
20	5D	AX-110410822	1.9077	200.0141
20	5D	AX-109874244	1.9077	200.0141
20	5D	AX-111602250	0.2057	200.2199
20	5D	AX-110023145	0.2058	200.4257
20	5D	AX-108844514	0.2058	200.4257
20	5D	AX-109259697	12.4932	212.9189
20	5D	AX-109207441	14.57	227.4889
20	5D	AX-110979095	12.5614	240.0503
20	5D	AX-110930601	12.5614	240.0503
20	5D	AX-111547900	12.5614	240.0503
20	5D	AX-108936199	0.4184	240.4687
20	5D	AX-94686257	0.4184	240.4687
20	5D	AX-109968761	0.8404	241.3091
20	5D	AX-109350576	0.2058	241.5149
20	5D	AX-110369382	0.2058	241.5149
20	5D	AX-108804982	0.2058	241.5149
20	5D	AX-109341998	10.0335	251.5484
20	5D	AX-94914596	10.0335	251.5484

20	5D	AX-110130663	1.0418	252.5903
20	5D	AX-108853012	1.0418	252.5903
20	5D	AX-94940447	1.0418	252.5903
20	5D	AX-94896513	1.0418	252.5903
20	5D	AX-94708866	1.0418	252.5903
20	5D	AX-109856868	1.0418	252.5903
20	5D	AX-111559803	1.0418	252.5903
20	5D	AX-109290471	1.0418	252.5903
20	5D	AX-110091168	1.0418	252.5903
20	5D	AX-95198125	1.0418	252.5903
20	5D	AX-111023362	0.2075	252.7977
20	5D	AX-110480176	0.2075	252.7977
20	5D	AX-108922893	0.2075	252.7977
20	5D	AX-109737867	0.625	253.4228
20	5D	AX-108879112	0.625	253.4228
20	5D	AX-109487394	0.625	253.4228
20	5D	AX-111015879	0.625	253.4228
20	5D	AX-110383774	0.625	253.4228
20	5D	AX-109991906	0.625	253.4228
20	5D	AX-110382603	0.2066	253.6294
20	5D	AX-94637193	14.4881	268.1174
20	5D	AX-110426248	14.4881	268.1174
20	5D	AX-108726018	14.4881	268.1174
20	5D	AX-109972971	14.4881	268.1174
20	5D	AX-109458485	14.4881	268.1174
20	5D	AX-110103365	14.4881	268.1174
20	5D	AX-111367481	14.4881	268.1174
20	5D	AX-109419749	14.4881	268.1174
20	5D	AX-94511461	14.4881	268.1174
20	5D	AX-108934687	14.4881	268.1174
20	5D	AX-111511813	14.4881	268.1174
20	5D	AX-110386894	14.4881	268.1174
20	5D	AX-111356182	14.4881	268.1174
20	5D	AX-110401749	14.4881	268.1174
20	5D	AX-109078290	14.4881	268.1174
20	5D	AX-111563263	14.4881	268.1174
20	5D	AX-108993493	14.4881	268.1174
20	5D	AX-109290311	14.4881	268.1174
20	5D	AX-110467853	14.4881	268.1174
20	5D	AX-110833965	14.4881	268.1174
20	5D	AX-111071670	14.4881	268.1174
20	5D	AX-109443651	0.4149	268.5324
20	5D	AX-89322127	0.4149	268.5324
20	5D	AX-109436066	0.4149	268.5324

20	5D	AX-108999004	0.4149	268.5324
20	5D	AX-111413877	0.4149	268.5324
21	5D	AX-109998956	0	0
21	5D	AX-109940376	0	0
21	5D	AX-95194221	0	0
21	5D	AX-111170049	0.2165	0.2165
21	5D	AX-95658300	0.2165	0.2165
21	5D	AX-94764048	0.2165	0.2165
21	5D	AX-110365495	7.813	8.0294
21	5D	AX-109932470	7.813	8.0294
21	5D	AX-109368530	7.813	8.0294
21	5D	AX-109839072	7.813	8.0294
21	5D	AX-86177373	11.1773	19.2067
21	5D	AX-109364453	4.451	23.6577
21	5D	AX-110985580	5.3083	28.966
21	5D	AX-109118710	5.3083	28.966
21	5D	AX-94562565	5.3083	28.966
21	5D	AX-108876564	8.5833	37.5494
21	5D	AX-94509671	1.064	38.6133
21	5D	AX-109356547	9.3378	47.9512
21	5D	AX-111583250	1.3161	49.2673
21	5D	AX-111628117	1.3161	49.2673
21	5D	AX-109581736	1.3161	49.2673
21	5D	AX-111668191	1.3161	49.2673
21	5D	AX-110534995	1.3161	49.2673
21	5D	AX-111560608	1.3161	49.2673
21	5D	AX-111151792	1.3161	49.2673
21	5D	AX-111041702	1.3161	49.2673
21	5D	AX-110386625	1.3161	49.2673
21	5D	AX-109442398	1.3161	49.2673
21	5D	AX-108868834	1.3161	49.2673
21	5D	AX-110106626	1.3161	49.2673
21	5D	AX-109932657	1.3161	49.2673
21	5D	AX-109889753	1.3161	49.2673
21	5D	AX-109387209	1.3161	49.2673
21	5D	AX-108798928	1.3161	49.2673
21	5D	AX-109198167	1.3161	49.2673
21	5D	AX-108795482	1.3161	49.2673
21	5D	AX-110954265	1.3161	49.2673
21	5D	AX-86174014	1.3161	49.2673
21	5D	AX-94848716	1.3161	49.2673
21	5D	AX-94832851	1.5289	50.7961
21	5D	AX-112288030	1.0778	51.8739
21	5D	AX-111628138	2.9051	54.7789

21	5D	AX-109901647	2.9051	54.7789
21	5D	AX-109841868	2.9051	54.7789
21	5D	AX-109864334	2.9051	54.7789
21	5D	AX-108905558	2.9051	54.7789
21	5D	AX-108831745	2.9051	54.7789
21	5D	AX-110907796	2.9051	54.7789
21	5D	AX-110527974	2.9051	54.7789
21	5D	AX-109498480	2.9051	54.7789
21	5D	AX-109337325	2.9051	54.7789
21	5D	AX-110932823	2.9051	54.7789
21	5D	AX-109933011	2.9051	54.7789
21	5D	AX-111504246	2.9051	54.7789
21	5D	AX-111129892	2.9051	54.7789
21	5D	AX-111127748	2.9051	54.7789
21	5D	AX-110420662	2.9051	54.7789
21	5D	AX-110907340	2.9051	54.7789
21	5D	AX-109350042	2.9051	54.7789
21	5D	AX-111626194	2.9051	54.7789
21	5D	AX-95098365	2.9051	54.7789
21	5D	AX-108970025	2.9051	54.7789
21	5D	AX-109291076	2.9051	54.7789
21	5D	AX-109504457	2.9051	54.7789
21	5D	AX-94831039	1.5289	56.3078
21	5D	AX-111095540	15.8515	72.1593
21	5D	AX-110959860	15.8515	72.1593
21	5D	AX-111118810	15.8515	72.1593
21	5D	AX-109342361	15.8515	72.1593
21	5D	AX-108882272	15.8515	72.1593
21	5D	AX-108875349	15.8515	72.1593
21	5D	AX-108902185	15.8515	72.1593
21	5D	AX-111529524	15.8515	72.1593
21	5D	AX-109349375	15.8515	72.1593
21	5D	AX-109836660	15.8515	72.1593
21	5D	AX-108932393	15.8515	72.1593
21	5D	AX-110390780	15.8515	72.1593
21	5D	AX-109303722	15.8515	72.1593
21	5D	AX-111693311	15.8515	72.1593
21	5D	AX-111045033	15.8515	72.1593
21	5D	AX-109894149	15.8515	72.1593
21	5D	AX-108997944	15.8515	72.1593
21	5D	AX-110088111	15.8515	72.1593
21	5D	AX-95658824	15.8515	72.1593
21	5D	AX-109584999	8.0285	80.1878
21	5D	AX-109074691	8.0285	80.1878

21	5D	AX-110600694	8.0285	80.1878
21	5D	AX-111173070	8.0285	80.1878
21	5D	AX-111717509	8.0285	80.1878
21	5D	AX-110997116	7.0169	87.2046
22	6A	AX-111652496	0	0
22	6A	AX-110942612	0	0
22	6A	AX-110401321	0.4367	0.4367
22	6A	AX-110128714	0.6494	1.0861
22	6A	AX-109292107	0.6494	1.0861
22	6A	AX-111540847	0.6494	1.0861
22	6A	AX-109453013	0.6494	1.0861
22	6A	AX-109552826	0.6494	1.0861
22	6A	AX-108936851	0.6494	1.0861
22	6A	AX-110492595	0.6494	1.0861
22	6A	AX-109864085	0.6494	1.0861
22	6A	AX-110126107	0.6494	1.0861
22	6A	AX-111358884	0.6494	1.0861
22	6A	AX-94666378	0.6494	1.0861
22	6A	AX-109329400	0.6494	1.0861
22	6A	AX-109293758	0.6494	1.0861
22	6A	AX-110399963	0.6494	1.0861
22	6A	AX-109854030	0.6494	1.0861
22	6A	AX-111579396	0.6494	1.0861
22	6A	AX-94598997	0.6494	1.0861
22	6A	AX-95009613	0.6494	1.0861
22	6A	AX-110426515	14.0696	15.1557
22	6A	AX-109533257	37.6019	52.7576
22	6A	AX-110637693	0.4167	53.1743
22	6A	AX-111053235	0.844	54.0183
22	6A	AX-109869394	0.844	54.0183
22	6A	AX-110451544	0.844	54.0183
22	6A	AX-109450894	1.9323	55.9506
22	6A	AX-108947474	3.2655	59.2161
22	6A	AX-95659559	3.2655	59.2161
22	6A	AX-109841642	0.4132	59.6293
22	6A	AX-86182466	3.7682	63.3975
22	6A	AX-111041695	3.7682	63.3975
22	6A	AX-110452864	3.7682	63.3975
22	6A	AX-110936625	3.7682	63.3975
22	6A	AX-111066934	3.7682	63.3975
22	6A	AX-109456103	3.7682	63.3975
22	6A	AX-110931567	3.7682	63.3975
22	6A	AX-109959942	3.7682	63.3975
22	6A	AX-109405723	3.7682	63.3975

22	6A	AX-108869759	3.7682	63.3975
22	6A	AX-89756375	3.7682	63.3975
22	6A	AX-110163062	0.2066	63.6041
22	6A	AX-109926578	1.7028	65.3069
22	6A	AX-110067051	1.7028	65.3069
22	6A	AX-111047262	1.7028	65.3069
22	6A	AX-108803666	1.7028	65.3069
22	6A	AX-111129045	1.7028	65.3069
22	6A	AX-109067536	0.2066	65.5135
22	6A	AX-108757665	0.2066	65.5135
22	6A	AX-110937386	0.4167	65.9302
22	6A	AX-108823309	1.2769	67.2071
22	6A	AX-110516770	1.2769	67.2071
22	6A	AX-110987915	1.055	68.2621
22	6A	AX-108813367	0.6303	68.8923
22	6A	AX-108936268	0.4184	69.3108
22	6A	AX-109842268	0.4184	69.3108
22	6A	AX-110451033	0.4184	69.3108
22	6A	AX-112288671	0.4184	69.3108
22	6A	AX-108999267	0.4184	69.3108
22	6A	AX-108751727	0.4184	69.3108
22	6A	AX-111062217	0.844	70.1547
22	6A	AX-109829293	0.844	70.1547
22	6A	AX-110174295	0.844	70.1547
22	6A	AX-110494629	0.844	70.1547
22	6A	AX-110636327	0.844	70.1547
22	6A	AX-109815878	0.844	70.1547
22	6A	AX-111727984	0.844	70.1547
22	6A	AX-110516661	0.844	70.1547
22	6A	AX-109936606	0.844	70.1547
22	6A	AX-110975704	0.844	70.1547
22	6A	AX-110959446	0.844	70.1547
22	6A	AX-109869385	0.844	70.1547
22	6A	AX-108729121	0.844	70.1547
22	6A	AX-111589688	0.844	70.1547
22	6A	AX-109862694	0.844	70.1547
22	6A	AX-111468556	0.844	70.1547
22	6A	AX-110428458	0.844	70.1547
22	6A	AX-111238130	0.844	70.1547
22	6A	AX-111631107	0.844	70.1547
22	6A	AX-111041045	0.844	70.1547
22	6A	AX-110905863	0.844	70.1547
22	6A	AX-109300313	0.844	70.1547
22	6A	AX-109329440	0.844	70.1547

22	6A	AX-111782559	0.844	70.1547
22	6A	AX-110578161	0.844	70.1547
22	6A	AX-111537245	0.844	70.1547
22	6A	AX-110475358	0.844	70.1547
22	6A	AX-110641980	0.844	70.1547
22	6A	AX-108833703	0.844	70.1547
22	6A	AX-109345228	0.844	70.1547
22	6A	AX-111562129	0.844	70.1547
22	6A	AX-110103615	0.844	70.1547
22	6A	AX-108800982	0.844	70.1547
22	6A	AX-110926771	0.844	70.1547
22	6A	AX-111576549	0.844	70.1547
22	6A	AX-108770270	0.844	70.1547
22	6A	AX-111561892	0.844	70.1547
22	6A	AX-110631952	0.844	70.1547
22	6A	AX-111560481	0.844	70.1547
22	6A	AX-111467340	0.844	70.1547
22	6A	AX-109335151	0.844	70.1547
22	6A	AX-110455630	0.844	70.1547
22	6A	AX-108843004	0.844	70.1547
22	6A	AX-111085374	0.844	70.1547
22	6A	AX-108786424	0.844	70.1547
22	6A	AX-109940869	0.844	70.1547
22	6A	AX-110998822	0.844	70.1547
22	6A	AX-111158920	0.844	70.1547
22	6A	AX-109319663	0.844	70.1547
22	6A	AX-110986476	0.844	70.1547
22	6A	AX-108794894	0.844	70.1547
22	6A	AX-108876918	0.844	70.1547
22	6A	AX-111603764	0.844	70.1547
22	6A	AX-109386845	0.844	70.1547
22	6A	AX-108856496	0.844	70.1547
22	6A	AX-109368197	0.844	70.1547
22	6A	AX-86171210	0.844	70.1547
22	6A	AX-110459788	0.844	70.1547
22	6A	AX-111714315	0.844	70.1547
22	6A	AX-110081145	0.844	70.1547
22	6A	AX-109959237	0.844	70.1547
22	6A	AX-109559781	0.844	70.1547
22	6A	AX-111561134	0.844	70.1547
22	6A	AX-110019288	0.844	70.1547
22	6A	AX-111582627	0.844	70.1547
22	6A	AX-110431636	0.844	70.1547
22	6A	AX-108892193	0.844	70.1547

22	6A	AX-109324047	0.844	70.1547
22	6A	AX-111470323	0.844	70.1547
22	6A	AX-109280410	0.844	70.1547
22	6A	AX-111472315	0.844	70.1547
22	6A	AX-108964679	0.844	70.1547
22	6A	AX-109912972	0.844	70.1547
22	6A	AX-109912861	0.844	70.1547
22	6A	AX-110580314	0.844	70.1547
22	6A	AX-110555407	0.844	70.1547
22	6A	AX-111653048	0.844	70.1547
22	6A	AX-86177686	0.844	70.1547
22	6A	AX-89586005	0.844	70.1547
22	6A	AX-111475401	0.844	70.1547
22	6A	AX-109873015	0.844	70.1547
22	6A	AX-110696918	0.844	70.1547
22	6A	AX-111068427	0.844	70.1547
22	6A	AX-110632560	0.844	70.1547
22	6A	AX-109038195	0.844	70.1547
22	6A	AX-110419286	0.844	70.1547
22	6A	AX-110739764	0.844	70.1547
22	6A	AX-109994317	0.844	70.1547
22	6A	AX-110024896	0.844	70.1547
22	6A	AX-110923949	0.844	70.1547
22	6A	AX-108869443	0.844	70.1547
22	6A	AX-109834501	0.844	70.1547
22	6A	AX-108979180	0.844	70.1547
22	6A	AX-109484160	0.844	70.1547
22	6A	AX-109461325	0.844	70.1547
22	6A	AX-110043640	0.844	70.1547
22	6A	AX-110981630	0.844	70.1547
22	6A	AX-109833877	0.844	70.1547
22	6A	AX-111641373	0.844	70.1547
22	6A	AX-109869040	0.844	70.1547
22	6A	AX-109864907	0.844	70.1547
22	6A	AX-111258881	0.844	70.1547
22	6A	AX-111083082	0.844	70.1547
22	6A	AX-109575335	0.844	70.1547
22	6A	AX-86176620	0.844	70.1547
22	6A	AX-109546954	0.844	70.1547
22	6A	AX-111015762	0.844	70.1547
22	6A	AX-109914406	0.844	70.1547
22	6A	AX-111613059	0.844	70.1547
22	6A	AX-110489678	0.844	70.1547
22	6A	AX-109329219	0.844	70.1547

22	6A	AX-109480324	0.844	70.1547
22	6A	AX-110007785	0.844	70.1547
22	6A	AX-110196810	0.844	70.1547
22	6A	AX-109564670	0.844	70.1547
22	6A	AX-111573430	0.844	70.1547
22	6A	AX-111607778	0.844	70.1547
22	6A	AX-109859423	0.844	70.1547
22	6A	AX-111509102	0.844	70.1547
22	6A	AX-94433057	0.844	70.1547
22	6A	AX-86165116	0.6303	70.785
22	6A	AX-108892196	0.6303	70.785
22	6A	AX-109490063	0.6303	70.785
22	6A	AX-110960515	0.6303	70.785
22	6A	AX-111094684	0.6303	70.785
22	6A	AX-111237921	0.6303	70.785
22	6A	AX-108844308	0.6303	70.785
22	6A	AX-109409195	0.6303	70.785
22	6A	AX-111137643	0.6303	70.785
22	6A	AX-109962980	0.6303	70.785
22	6A	AX-111629868	0.6303	70.785
22	6A	AX-110406053	0.6303	70.785
22	6A	AX-110034277	0.6303	70.785
22	6A	AX-108863158	0.6303	70.785
22	6A	AX-108873107	0.6303	70.785
22	6A	AX-109292338	0.6303	70.785
22	6A	AX-110955246	0.6303	70.785
22	6A	AX-111606694	0.6303	70.785
22	6A	AX-110514475	0.6303	70.785
22	6A	AX-110361606	0.6303	70.785
22	6A	AX-111579006	0.6303	70.785
22	6A	AX-109588190	0.6303	70.785
22	6A	AX-86171191	0.6303	70.785
22	6A	AX-109825019	0.6303	70.785
22	6A	AX-111012430	0.6303	70.785
22	6A	AX-109582488	0.6303	70.785
22	6A	AX-111655495	0.6303	70.785
22	6A	AX-111455304	0.6303	70.785
22	6A	AX-110919649	0.6303	70.785
22	6A	AX-108768553	0.6303	70.785
22	6A	AX-108863742	0.6303	70.785
22	6A	AX-111217075	0.6303	70.785
22	6A	AX-109401268	0.6303	70.785
22	6A	AX-109008942	0.6303	70.785
22	6A	AX-110099563	0.6303	70.785

22	6A	AX-110930748	0.6303	70.785
22	6A	AX-109071668	0.6303	70.785
22	6A	AX-110135639	0.6303	70.785
22	6A	AX-110740737	0.6303	70.785
22	6A	AX-111503562	0.6303	70.785
22	6A	AX-111198806	0.6303	70.785
22	6A	AX-111519066	0.6303	70.785
22	6A	AX-110433660	0.6303	70.785
22	6A	AX-108834458	0.6303	70.785
22	6A	AX-110611990	0.6303	70.785
22	6A	AX-110591570	0.6303	70.785
22	6A	AX-86173103	0.6303	70.785
22	6A	AX-110992859	0.6303	70.785
22	6A	AX-110505412	0.6303	70.785
22	6A	AX-109378246	0.6303	70.785
22	6A	AX-109320814	0.6303	70.785
22	6A	AX-110476357	0.6303	70.785
22	6A	AX-109924089	0.6303	70.785
22	6A	AX-108803180	0.6303	70.785
22	6A	AX-110726653	0.6303	70.785
22	6A	AX-109488723	0.6303	70.785
22	6A	AX-109418600	0.6303	70.785
22	6A	AX-111024457	0.6303	70.785
22	6A	AX-108756035	0.6303	70.785
22	6A	AX-94446421	0.6303	70.785
22	6A	AX-109528569	0.6303	70.785
22	6A	AX-108766833	0.6303	70.785
22	6A	AX-110645584	0.6303	70.785
22	6A	AX-111475262	0.6303	70.785
22	6A	AX-109022946	0.6303	70.785
22	6A	AX-109471829	0.6303	70.785
22	6A	AX-111503454	0.6303	70.785
22	6A	AX-110954888	0.6303	70.785
22	6A	AX-109935137	0.6303	70.785
22	6A	AX-108861011	0.6303	70.785
22	6A	AX-110926805	0.6303	70.785
22	6A	AX-110596613	0.6303	70.785
22	6A	AX-109538609	0.6303	70.785
22	6A	AX-110584729	0.6303	70.785
22	6A	AX-110918280	0.6303	70.785
22	6A	AX-86173102	0.6303	70.785
22	6A	AX-111537438	0.2075	70.9925
22	6A	AX-111493438	0.2075	70.9925
22	6A	AX-108755600	0.2075	70.9925

22	6A	AX-109355068	0.2075	70.9925
22	6A	AX-108868726	0.2075	70.9925
22	6A	AX-111695215	0.2075	70.9925
22	6A	AX-110158467	0.2075	70.9925
22	6A	AX-111616406	0.2075	70.9925
22	6A	AX-110035088	0.2075	70.9925
22	6A	AX-110983394	0.2075	70.9925
22	6A	AX-110400957	0.2075	70.9925
22	6A	AX-109967508	0.2075	70.9925
22	6A	AX-110378561	0.2075	70.9925
22	6A	AX-110445151	0.2075	70.9925
22	6A	AX-109416976	0.2075	70.9925
22	6A	AX-108778239	0.2075	70.9925
22	6A	AX-110510842	0.2075	70.9925
22	6A	AX-111119782	0.2075	70.9925
22	6A	AX-110032974	0.2075	70.9925
22	6A	AX-109441839	0.2075	70.9925
22	6A	AX-109270709	0.2075	70.9925
22	6A	AX-111189374	0.2075	70.9925
22	6A	AX-110936312	0.2075	70.9925
22	6A	AX-110018729	0.2075	70.9925
22	6A	AX-109556097	0.2075	70.9925
22	6A	AX-111687445	0.2075	70.9925
22	6A	AX-109535576	1.2715	72.264
22	6A	AX-111157494	1.2715	72.264
22	6A	AX-109947015	1.2715	72.264
22	6A	AX-108837322	1.2715	72.264
22	6A	AX-109416581	1.2715	72.264
22	6A	AX-111532746	1.2715	72.264
22	6A	AX-109500945	1.2715	72.264
22	6A	AX-109884699	1.2715	72.264
22	6A	AX-109355603	1.2715	72.264
22	6A	AX-108760790	1.2715	72.264
22	6A	AX-110920837	1.2715	72.264
22	6A	AX-86185030	1.2715	72.264
22	6A	AX-111460761	1.2715	72.264
22	6A	AX-109365121	1.2715	72.264
22	6A	AX-108944688	1.2715	72.264
22	6A	AX-110124435	1.2715	72.264
22	6A	AX-111537740	1.2715	72.264
22	6A	AX-111133649	1.2715	72.264
22	6A	AX-109555430	1.2715	72.264
22	6A	AX-111221529	1.2715	72.264
22	6A	AX-110521722	1.2715	72.264

22	6A	AX-109587414	1.2715	72.264
22	6A	AX-110993884	1.2715	72.264
22	6A	AX-110416028	1.2715	72.264
22	6A	AX-111628659	1.2715	72.264
22	6A	AX-110939352	1.2715	72.264
22	6A	AX-111070549	1.2715	72.264
22	6A	AX-110593071	1.2715	72.264
22	6A	AX-111082478	1.2715	72.264
22	6A	AX-108775871	1.2715	72.264
22	6A	AX-110966748	1.2715	72.264
22	6A	AX-108810516	1.2715	72.264
22	6A	AX-109886765	1.2715	72.264
22	6A	AX-108831922	1.2715	72.264
22	6A	AX-109288715	1.2715	72.264
22	6A	AX-111472542	1.2715	72.264
22	6A	AX-111658916	1.2715	72.264
22	6A	AX-108863697	1.2715	72.264
22	6A	AX-110523092	1.2715	72.264
22	6A	AX-111233423	1.2715	72.264
22	6A	AX-109458778	1.2715	72.264
22	6A	AX-110087220	1.2715	72.264
22	6A	AX-109859314	0.2066	72.4706
22	6A	AX-111474283	0.2066	72.4706
22	6A	AX-108957592	0.2066	72.4706
22	6A	AX-108808389	0.2066	72.4706
22	6A	AX-111219980	0.2066	72.4706
22	6A	AX-109816368	0.2066	72.4706
22	6A	AX-111219632	0.2066	72.4706
22	6A	AX-110540721	0.2066	72.4706
22	6A	AX-108877904	0.2066	72.4706
22	6A	AX-108803768	0.2066	72.4706
22	6A	AX-110406728	0.2066	72.4706
22	6A	AX-109864541	0.2066	72.4706
22	6A	AX-110068739	0.2066	72.4706
22	6A	AX-109458626	0.2066	72.4706
22	6A	AX-111722777	0.2066	72.4706
22	6A	AX-111044766	0.2066	72.4706
22	6A	AX-109304197	0.2066	72.4706
22	6A	AX-111184795	0.2066	72.4706
22	6A	AX-94476856	0.2066	72.4706
22	6A	AX-109842718	0.2066	72.6772
22	6A	AX-111177711	0.2066	72.6772
22	6A	AX-109469179	0.2066	72.6772
22	6A	AX-109332464	0.2066	72.6772

22	6A	AX-108770250	0.2066	72.6772
22	6A	AX-109852180	0.2066	72.6772
22	6A	AX-109456124	0.2066	72.6772
22	6A	AX-111131762	1.0506	73.7278
22	6A	AX-111764165	2.1472	75.875
22	6A	AX-111019702	2.1472	75.875
22	6A	AX-108799082	2.1472	75.875
22	6A	AX-110383439	2.1472	75.875
22	6A	AX-111037916	2.1472	75.875
22	6A	AX-112288721	2.1472	75.875
22	6A	AX-110165332	2.1472	75.875
22	6A	AX-110422751	2.1472	75.875
22	6A	AX-109291122	2.1472	75.875
22	6A	AX-109858561	2.1472	75.875
22	6A	AX-110954166	2.1472	75.875
22	6A	AX-111058445	2.1472	75.875
22	6A	AX-111695420	2.1472	75.875
22	6A	AX-111819846	2.1472	75.875
22	6A	AX-110928492	0.8334	76.7084
22	6A	AX-111168832	0.8334	76.7084
22	6A	AX-89439930	0.8334	76.7084
22	6A	AX-94418601	0.8334	76.7084
22	6A	AX-109040041	0.4149	77.1234
22	6A	AX-110496683	5.2706	82.3939
22	6A	AX-110577250	5.2706	82.3939
22	6A	AX-110392325	5.2706	82.3939
22	6A	AX-110945767	5.2706	82.3939
22	6A	AX-108987567	5.2706	82.3939
22	6A	AX-111780081	5.2706	82.3939
22	6A	AX-109389299	5.2706	82.3939
22	6A	AX-111450286	0.2075	82.6014
22	6A	AX-111655602	0.4167	83.0181
22	6A	AX-111204958	0.4167	83.0181
22	6A	AX-111569157	0.4167	83.0181
22	6A	AX-110442127	0.4167	83.0181
22	6A	AX-108997936	0.4167	83.0181
22	6A	AX-111053903	0.2066	83.2247
22	6A	AX-110926984	0.2066	83.4313
22	6A	AX-111520882	0.2066	83.4313
22	6A	AX-111507791	0.2066	83.4313
22	6A	AX-111496661	0.2066	83.4313
22	6A	AX-108762749	0.2066	83.4313
22	6A	AX-109934181	0.2066	83.4313
22	6A	AX-110947227	0.2066	83.4313

22	6A	AX-94499080	0.2066	83.4313
22	6A	AX-111181100	0.2066	83.6379
22	6A	AX-109847419	0.2066	83.6379
22	6A	AX-108751655	0.2066	83.6379
22	6A	AX-110970369	0.2066	83.6379
22	6A	AX-95159098	0.2066	83.6379
22	6A	AX-109295804	2.1848	85.8227
22	6A	AX-109370898	2.6811	88.5039
22	6A	AX-111134280	1.2878	89.7917
22	6A	AX-108891954	0.6329	90.4246
22	6A	AX-110371990	1.9747	92.3993
22	6A	AX-109837110	0.2146	92.6139
22	6A	AX-111665420	3.1573	95.7713
22	6A	AX-108932983	10.9406	106.7118
22	6A	AX-110581340	10.9406	106.7118
22	6A	AX-111627964	10.9406	106.7118
22	6A	AX-108914846	10.9406	106.7118
22	6A	AX-110736945	10.9406	106.7118
22	6A	AX-108904507	10.9406	106.7118
22	6A	AX-110041535	10.9406	106.7118
22	6A	AX-110072628	5.8404	112.5522
22	6A	AX-108811654	5.8404	112.5522
22	6A	AX-109921374	5.8404	112.5522
22	6A	AX-108917469	1.9661	114.5183
22	6A	AX-111063393	1.9661	114.5183
22	6A	AX-110547197	1.9661	114.5183
22	6A	AX-111548029	1.9661	114.5183
22	6A	AX-109373226	0.2092	114.7275
22	6A	AX-110589025	0.2092	114.7275
22	6A	AX-110569267	0.2092	114.7275
22	6A	AX-111574882	0.2092	114.7275
22	6A	AX-110101588	0.2083	114.9358
22	6A	AX-111550942	0.2083	114.9358
22	6A	AX-109984471	0.2083	114.9358
22	6A	AX-108729874	12.0713	127.0071
22	6A	AX-108769468	12.0713	127.0071
22	6A	AX-109820371	0.431	127.4382
22	6A	AX-111489359	0.4237	127.8619
22	6A	AX-109029878	0.4237	127.8619
22	6A	AX-110495378	0.4237	127.8619
22	6A	AX-109352951	0.4237	127.8619
22	6A	AX-111562681	0.4237	127.8619
22	6A	AX-110576642	0.4237	127.8619
22	6A	AX-111759473	0.4237	127.8619

22	6A	AX-109542604	0.4237	127.8619
22	6A	AX-94395775	0.4237	127.8619
22	6A	AX-109558600	1.0824	128.9443
22	6A	AX-94422432	0.2128	129.1571
22	6A	AX-95090235	1.5356	130.6927
22	6A	AX-110978430	0.431	131.1237
22	6A	AX-110987369	0.6466	131.7703
23	6B	AX-111567193	0	0
23	6B	AX-110966371	1.1113	1.1113
23	6B	AX-110922381	0.422	1.5332
23	6B	AX-109499082	0.422	1.5332
23	6B	AX-109598916	1.7248	3.2581
23	6B	AX-89682312	0.4167	3.6747
23	6B	AX-109841359	2.8666	6.5413
23	6B	AX-109491273	2.8666	6.5413
23	6B	AX-94621559	2.8666	6.5413
23	6B	AX-111594421	1.0685	7.6099
23	6B	AX-111109328	0.2092	7.8191
23	6B	AX-109993622	0.2092	7.8191
23	6B	AX-109905689	0.2092	7.8191
23	6B	AX-109900320	0.422	8.241
23	6B	AX-109353209	0.4237	8.6648
23	6B	AX-108736357	0.4237	8.6648
23	6B	AX-110960130	0.4237	8.6648
23	6B	AX-109330624	0.4237	8.6648
23	6B	AX-111594536	10.0806	18.7454
23	6B	AX-109931880	2.0845	20.8299
23	6B	AX-109270335	2.0845	20.8299
23	6B	AX-108823992	0.4202	21.2501
23	6B	AX-110996072	4.063	25.3131
23	6B	AX-108943581	0.2092	25.5223
23	6B	AX-110382125	0.6383	26.1606
23	6B	AX-109599638	1.064	27.2246
23	6B	AX-109825294	0.2128	27.4374
23	6B	AX-109330846	0.2146	27.652
23	6B	AX-111129000	0.4202	28.0722
23	6B	AX-109940549	0.4202	28.0722
23	6B	AX-111564510	0.4202	28.0722
23	6B	AX-108853850	1.2823	29.3545
23	6B	AX-89344223	1.2823	29.3545
23	6B	AX-110472291	3.3683	32.7228
23	6B	AX-109325937	15.295	48.0178
23	6B	AX-109370998	0.2092	48.227
23	6B	AX-111647591	0.2092	48.227

23	6B	AX-109953676	0.2092	48.227
23	6B	AX-109332231	0.2092	48.227
23	6B	AX-110510046	0.4202	48.6472
23	6B	AX-110018933	0.4202	48.6472
23	6B	AX-108744211	0.4202	48.6472
23	6B	AX-109847757	0.4202	48.6472
23	6B	AX-108770725	0.4202	48.6472
23	6B	AX-109885910	0.4184	49.0656
23	6B	AX-108936780	0.4184	49.0656
23	6B	AX-109371154	0.4184	49.0656
23	6B	AX-109357371	0.4184	49.0656
23	6B	AX-110442365	0.4184	49.4841
23	6B	AX-109895765	0.4184	49.4841
23	6B	AX-109858727	0.4184	49.4841
23	6B	AX-111764175	0.4184	49.4841
23	6B	AX-109500087	0.4184	49.4841
23	6B	AX-110149310	0.4184	49.4841
23	6B	AX-111139422	0.4184	49.4841
23	6B	AX-110455298	0.4184	49.4841
23	6B	AX-110384791	0.4184	49.9025
23	6B	AX-110432618	0.4184	49.9025
23	6B	AX-108736410	0.4184	49.9025
23	6B	AX-109580560	0.4184	50.3209
23	6B	AX-108732339	0.4184	50.3209
23	6B	AX-109317417	0.4184	50.3209
23	6B	AX-109421706	0.2092	50.5301
23	6B	AX-111604322	0.4202	50.9503
23	6B	AX-109846005	0.4202	50.9503
23	6B	AX-111054484	0.4202	50.9503
23	6B	AX-108913374	0.4202	50.9503
23	6B	AX-109938252	0.4202	50.9503
23	6B	AX-109951417	0.4202	50.9503
23	6B	AX-111516223	0.4202	50.9503
23	6B	AX-111004330	0.4202	50.9503
23	6B	AX-109273656	0.4202	50.9503
23	6B	AX-110086207	0.4202	50.9503
23	6B	AX-108762758	0.2101	51.1604
23	6B	AX-109408478	0.2101	51.3705
23	6B	AX-89550449	0.2101	51.3705
23	6B	AX-109882390	0.4237	51.7942
23	6B	AX-109362357	0.6383	52.4325
23	6B	AX-109883185	0.6383	52.4325
23	6B	AX-110032118	0.6383	52.4325
23	6B	AX-108946754	0.6383	52.4325

23	6B	AX-109305172	0.6383	52.4325
23	6B	AX-111543278	0.6383	52.4325
23	6B	AX-111029292	0.6383	52.4325
23	6B	AX-95256822	0.6383	52.4325
23	6B	AX-108733957	0.4167	52.8492
23	6B	AX-111536663	0.4167	52.8492
23	6B	AX-111700475	0.4167	52.8492
23	6B	AX-110502033	0.4167	52.8492
23	6B	AX-111001967	0.4167	52.8492
23	6B	AX-109314339	0.4167	52.8492
23	6B	AX-110959650	0.4167	52.8492
23	6B	AX-108782645	0.4167	52.8492
23	6B	AX-95181453	0.4167	52.8492
23	6B	AX-108855625	0.4167	52.8492
23	6B	AX-109932370	0.4167	52.8492
23	6B	AX-108868603	0.4167	52.8492
23	6B	AX-110624733	0.4167	52.8492
23	6B	AX-111648853	0.4167	52.8492
23	6B	AX-109398995	0.4167	52.8492
23	6B	AX-111501011	0.4167	52.8492
23	6B	AX-109815710	0.4167	52.8492
23	6B	AX-110672473	0.4167	52.8492
23	6B	AX-109391750	0.4167	52.8492
23	6B	AX-110079376	0.4167	52.8492
23	6B	AX-111592951	0.4167	52.8492
23	6B	AX-109336814	0.4167	52.8492
23	6B	AX-109525611	0.4167	52.8492
23	6B	AX-109292852	0.2075	53.0567
23	6B	AX-111649072	0.2075	53.0567
23	6B	AX-111473381	0.4184	53.4751
23	6B	AX-108747518	0.6303	54.1054
23	6B	AX-108993377	0.6303	54.1054
23	6B	AX-110580782	0.6303	54.1054
23	6B	AX-109508249	0.2083	54.3137
23	6B	AX-110404003	0.2101	54.5238
23	6B	AX-111542357	0.2101	54.5238
23	6B	AX-108902410	0.6383	55.1621
23	6B	AX-109823731	1.0685	56.2307
23	6B	AX-111513561	1.0685	56.2307
23	6B	AX-111022908	1.0685	56.2307
23	6B	AX-109393472	1.0685	56.2307
23	6B	AX-111555747	1.0685	56.2307
23	6B	AX-110386321	1.0685	56.2307
23	6B	AX-111480682	1.0685	56.2307

23	6B	AX-110925228	1.0685	56.2307
23	6B	AX-110013870	1.0685	56.2307
23	6B	AX-110362828	0.6356	56.8663
23	6B	AX-110199811	0.6356	56.8663
23	6B	AX-109871677	0.4255	57.2918
23	6B	AX-108750117	0.4255	57.2918
23	6B	AX-110974008	0.4255	57.2918
23	6B	AX-109601574	0.4255	57.2918
23	6B	AX-109354285	0.4255	57.2918
23	6B	AX-109356924	0.4255	57.2918
23	6B	AX-109602187	0.4255	57.2918
23	6B	AX-109370111	0.4255	57.2918
23	6B	AX-109317615	0.4255	57.2918
23	6B	AX-110027684	0.4255	57.2918
23	6B	AX-108872433	0.4255	57.2918
23	6B	AX-109423000	0.4255	57.2918
23	6B	AX-89311750	0.4255	57.2918
23	6B	AX-110411078	0.4255	57.2918
23	6B	AX-109458881	0.4255	57.2918
23	6B	AX-95009966	0.4255	57.2918
23	6B	AX-110928644	0.4274	57.7192
23	6B	AX-110987417	0.8622	58.5814
23	6B	AX-108883027	0.2128	58.7941
23	6B	AX-108816205	0.2128	58.7941
23	6B	AX-111554103	0.2128	58.7941
23	6B	AX-110024866	0.6356	59.4297
23	6B	AX-109372346	0.6356	59.4297
23	6B	AX-111045757	0.6356	59.4297
23	6B	AX-108926385	0.6411	60.0708
23	6B	AX-111039680	0.6411	60.0708
23	6B	AX-108744137	0.6411	60.0708
23	6B	AX-109834904	0.6438	60.7146
23	6B	AX-108854333	0.6438	60.7146
23	6B	AX-109040118	0.211	60.9256
23	6B	AX-110407894	0.211	60.9256
23	6B	AX-109464666	0.211	60.9256
23	6B	AX-109562350	0.2092	61.1348
23	6B	AX-108733633	0.2092	61.1348
23	6B	AX-108934604	0.2083	61.3431
23	6B	AX-108781304	0.2083	61.3431
23	6B	AX-109317829	0.4202	61.7633
23	6B	AX-109513023	0.2101	61.9734
23	6B	AX-108753446	0.2101	61.9734
23	6B	AX-109107826	0.2101	61.9734

23	6B	AX-111587040	0.2101	61.9734
23	6B	AX-110002353	0.2101	61.9734
23	6B	AX-110427037	0.2101	61.9734
23	6B	AX-109854760	0.2101	61.9734
23	6B	AX-108952507	0.2101	61.9734
23	6B	AX-110690584	0.2101	61.9734
23	6B	AX-108868224	0.2101	61.9734
23	6B	AX-109303140	0.2101	61.9734
23	6B	AX-109513034	0.2101	61.9734
23	6B	AX-109990947	0.2101	61.9734
23	6B	AX-109997676	0.2101	61.9734
23	6B	AX-108885091	0.2101	61.9734
23	6B	AX-111674902	0.2101	61.9734
23	6B	AX-110536146	0.2101	61.9734
23	6B	AX-109445152	0.2101	61.9734
23	6B	AX-109033857	0.2101	61.9734
23	6B	AX-109511525	0.2101	61.9734
23	6B	AX-111562733	0.2101	61.9734
23	6B	AX-109073134	0.2101	61.9734
23	6B	AX-109302903	0.2101	61.9734
23	6B	AX-109539352	0.2101	61.9734
23	6B	AX-108867815	0.2101	61.9734
23	6B	AX-108761692	0.2101	61.9734
23	6B	AX-110621549	0.2101	61.9734
23	6B	AX-108943177	0.2101	61.9734
23	6B	AX-111283251	0.2101	61.9734
23	6B	AX-109871378	0.2101	61.9734
23	6B	AX-94495507	0.2101	61.9734
23	6B	AX-94732905	0.2101	61.9734
23	6B	AX-94891168	0.2101	61.9734
23	6B	AX-110490610	0.2092	62.1826
23	6B	AX-110923823	0.2092	62.1826
23	6B	AX-111528309	0.2092	62.1826
23	6B	AX-111473979	0.4202	62.6028
23	6B	AX-108733139	0.4202	62.6028
23	6B	AX-111584479	0.4202	62.6028
23	6B	AX-109438035	0.4202	62.6028
23	6B	AX-110920236	0.4202	62.6028
23	6B	AX-108786414	0.4202	62.6028
23	6B	AX-111076083	0.4202	62.6028
23	6B	AX-110690650	0.4202	62.6028
23	6B	AX-109397717	0.4202	62.6028
23	6B	AX-110516589	0.4202	62.6028
23	6B	AX-110937917	0.4202	62.6028

23	6B	AX-109411853	0.4202	62.6028
23	6B	AX-110364627	0.4202	62.6028
23	6B	AX-111074264	0.4202	62.6028
23	6B	AX-109841246	0.4202	62.6028
23	6B	AX-110059387	0.4202	62.6028
23	6B	AX-109102036	0.4202	62.6028
23	6B	AX-108741450	0.4202	62.6028
23	6B	AX-110560655	0.4202	62.6028
23	6B	AX-109821025	0.4202	62.6028
23	6B	AX-110408267	0.4202	62.6028
23	6B	AX-110594031	0.4202	62.6028
23	6B	AX-109896983	0.4202	62.6028
23	6B	AX-111609048	0.4202	62.6028
23	6B	AX-110569959	0.4202	62.6028
23	6B	AX-111605867	0.4202	62.6028
23	6B	AX-111452922	0.4202	62.6028
23	6B	AX-95631491	0.4202	62.6028
23	6B	AX-95684214	0.4202	62.6028
23	6B	AX-111109946	0.211	62.8138
23	6B	AX-110400198	0.211	62.8138
23	6B	AX-109821421	0.211	62.8138
23	6B	AX-109651367	0.211	62.8138
23	6B	AX-110524380	0.211	62.8138
23	6B	AX-111027042	0.211	62.8138
23	6B	AX-111127728	0.211	62.8138
23	6B	AX-108895343	0.211	62.8138
23	6B	AX-110569296	0.211	62.8138
23	6B	AX-111022861	0.211	62.8138
23	6B	AX-111670599	0.211	62.8138
23	6B	AX-111786324	0.211	62.8138
23	6B	AX-109857181	0.211	62.8138
23	6B	AX-109300656	0.211	62.8138
23	6B	AX-110989330	0.211	62.8138
23	6B	AX-111263716	0.211	62.8138
23	6B	AX-108955912	0.211	62.8138
23	6B	AX-109442365	0.211	62.8138
23	6B	AX-108947086	0.211	62.8138
23	6B	AX-110476917	0.211	62.8138
23	6B	AX-109337275	0.211	62.8138
23	6B	AX-111503905	0.211	62.8138
23	6B	AX-111125920	0.211	62.8138
23	6B	AX-111069833	0.211	62.8138
23	6B	AX-109102926	0.211	62.8138
23	6B	AX-111130352	0.211	62.8138

23	6B	AX-110010345	0.211	62.8138
23	6B	AX-109838811	0.4202	63.2339
23	6B	AX-108976149	2.0011	65.235
23	6B	AX-109388519	0.2232	65.4582
23	6B	AX-111691729	1.57	67.0282
23	6B	AX-110515867	0.4202	67.4484
23	6B	AX-111078839	0.4202	67.4484
23	6B	AX-108977247	0.4202	67.4484
23	6B	AX-110462511	0.4202	67.4484
23	6B	AX-111126861	0.4202	67.4484
23	6B	AX-109382255	0.4202	67.4484
23	6B	AX-110042303	0.211	67.6594
23	6B	AX-110927675	0.211	67.6594
23	6B	AX-109912526	0.4237	68.0831
23	6B	AX-110674927	0.4237	68.0831
23	6B	AX-111548422	0.4237	68.0831
23	6B	AX-111549094	0.4237	68.0831
23	6B	AX-111555213	0.4237	68.0831
23	6B	AX-111471114	0.4237	68.0831
23	6B	AX-109512103	0.6383	68.7215
23	6B	AX-108793024	0.6383	68.7215
23	6B	AX-109304547	0.8548	69.5762
23	6B	AX-111182870	0.8548	69.5762
23	6B	AX-89417256	0.8548	69.5762
23	6B	AX-111143764	0.8548	69.5762
23	6B	AX-108767768	0.8548	69.5762
23	6B	AX-109454515	0.8548	69.5762
23	6B	AX-108728623	0.8548	69.5762
23	6B	AX-110594337	0.8548	69.5762
23	6B	AX-108940703	0.8548	69.5762
23	6B	AX-110997759	0.8548	69.5762
23	6B	AX-110905943	0.8548	69.5762
23	6B	AX-111510071	0.8548	69.5762
23	6B	AX-109081636	0.8548	69.5762
23	6B	AX-108781555	0.8548	69.5762
23	6B	AX-111099951	0.8548	69.5762
23	6B	AX-109347382	0.8548	69.5762
23	6B	AX-111483552	0.8548	69.5762
23	6B	AX-110741329	0.8548	69.5762
23	6B	AX-111154160	0.8548	69.5762
23	6B	AX-110531044	0.8548	69.5762
23	6B	AX-111000593	0.8548	69.5762
23	6B	AX-111473192	0.8548	69.5762
23	6B	AX-94600652	0.8548	69.5762

23	6B	AX-109508827	0.4255	70.0018
23	6B	AX-111468538	0.4255	70.0018
23	6B	AX-109084084	0.4255	70.0018
23	6B	AX-110574471	0.4255	70.0018
23	6B	AX-110612125	0.4255	70.0018
23	6B	AX-110174422	0.4255	70.0018
23	6B	AX-109847053	0.4255	70.0018
23	6B	AX-109275651	0.4292	70.431
23	6B	AX-110039192	0.4292	70.431
23	6B	AX-109478237	0.4292	70.431
23	6B	AX-109931771	0.2137	70.6446
23	6B	AX-111042332	0.2137	70.6446
23	6B	AX-111102500	0.2137	70.6446
23	6B	AX-89322751	0.2137	70.6446
23	6B	AX-111204801	0.2137	70.6446
23	6B	AX-110496736	0.4292	71.0738
23	6B	AX-110559317	0.4292	71.0738
23	6B	AX-110456402	0.4292	71.0738
23	6B	AX-110582354	0.4292	71.0738
23	6B	AX-109945157	0.4292	71.0738
23	6B	AX-108770675	0.4292	71.0738
23	6B	AX-110546971	0.4292	71.0738
23	6B	AX-111486170	0.4292	71.0738
23	6B	AX-110986682	0.4292	71.0738
23	6B	AX-110405253	0.4292	71.0738
23	6B	AX-111652073	0.4292	71.0738
23	6B	AX-110539376	0.4292	71.0738
23	6B	AX-111105825	0.4292	71.0738
23	6B	AX-111288797	0.4292	71.0738
23	6B	AX-111074553	0.4292	71.0738
23	6B	AX-109053992	0.4292	71.0738
23	6B	AX-109270380	0.4292	71.0738
23	6B	AX-108766872	0.4292	71.0738
23	6B	AX-111020319	0.4292	71.0738
23	6B	AX-110131308	0.4292	71.0738
23	6B	AX-109272061	0.4292	71.0738
23	6B	AX-111566434	0.4292	71.0738
23	6B	AX-110369581	0.4292	71.0738
23	6B	AX-109904570	0.4292	71.0738
23	6B	AX-109467528	0.4292	71.0738
23	6B	AX-109109402	0.4292	71.0738
23	6B	AX-109463468	0.4292	71.0738
23	6B	AX-109532654	0.4292	71.0738
23	6B	AX-109996579	0.2137	71.2875

23	6B	AX-110523220	0.4292	71.7167
23	6B	AX-108972276	0.211	71.9277
23	6B	AX-110417565	0.211	71.9277
23	6B	AX-111247484	0.211	71.9277
23	6B	AX-111487908	0.211	71.9277
23	6B	AX-109421662	0.211	71.9277
23	6B	AX-111155859	0.211	71.9277
23	6B	AX-109604392	0.211	71.9277
23	6B	AX-111517688	0.211	71.9277
23	6B	AX-110013167	0.211	71.9277
23	6B	AX-108942733	0.211	71.9277
23	6B	AX-109305096	0.211	71.9277
23	6B	AX-111605648	0.4202	72.3479
23	6B	AX-109946836	0.4202	72.3479
23	6B	AX-109274477	0.4202	72.3479
23	6B	AX-111020201	0.4202	72.3479
23	6B	AX-111585519	0.4202	72.3479
23	6B	AX-109387669	0.4202	72.3479
23	6B	AX-108912101	0.4202	72.3479
23	6B	AX-109476705	0.4202	72.3479
23	6B	AX-109307431	0.4202	72.3479
23	6B	AX-109293207	0.4202	72.3479
23	6B	AX-111568425	0.4202	72.3479
23	6B	AX-111489972	0.4202	72.3479
23	6B	AX-110028092	0.4202	72.3479
23	6B	AX-111762280	0.4202	72.3479
23	6B	AX-109836669	0.4202	72.3479
23	6B	AX-110738211	0.4202	72.3479
23	6B	AX-109055139	0.4202	72.3479
23	6B	AX-109494802	0.4202	72.3479
23	6B	AX-109504729	0.4202	72.3479
23	6B	AX-111491718	0.4202	72.3479
23	6B	AX-110997970	0.4202	72.3479
23	6B	AX-108729524	0.4202	72.3479
23	6B	AX-110507188	0.4202	72.3479
23	6B	AX-109903823	0.4202	72.3479
23	6B	AX-110455345	0.4202	72.3479
23	6B	AX-111636861	0.4202	72.3479
23	6B	AX-111516936	0.4202	72.3479
23	6B	AX-111059006	0.4202	72.3479
23	6B	AX-110027817	0.4202	72.3479
23	6B	AX-110434914	0.4202	72.3479
23	6B	AX-110611543	0.4202	72.3479
23	6B	AX-111783730	0.4202	72.3479

23	6B	AX-108741111	0.4202	72.3479
23	6B	AX-110163419	0.4202	72.3479
23	6B	AX-108969411	0.4202	72.3479
23	6B	AX-109650838	0.4202	72.3479
23	6B	AX-110649297	0.4202	72.3479
23	6B	AX-109820444	0.4202	72.3479
23	6B	AX-110426734	0.4202	72.3479
23	6B	AX-108912179	0.4202	72.3479
23	6B	AX-109942957	0.4202	72.3479
23	6B	AX-111764499	0.4202	72.3479
23	6B	AX-111579957	0.4202	72.3479
23	6B	AX-109438903	0.4202	72.3479
23	6B	AX-110505530	0.4202	72.3479
23	6B	AX-109983953	0.4202	72.3479
23	6B	AX-109944681	0.4202	72.3479
23	6B	AX-110036577	0.4202	72.3479
23	6B	AX-109313297	0.4202	72.3479
23	6B	AX-110059618	0.4202	72.3479
23	6B	AX-110386602	0.4202	72.3479
23	6B	AX-109033482	0.4202	72.3479
23	6B	AX-111574778	0.4202	72.3479
23	6B	AX-111090845	0.4202	72.3479
23	6B	AX-109032304	0.4202	72.3479
23	6B	AX-109885071	0.4202	72.3479
23	6B	AX-111067772	0.4202	72.3479
23	6B	AX-109400480	0.4202	72.3479
23	6B	AX-111831763	0.4202	72.3479
23	6B	AX-111483002	0.4202	72.3479
23	6B	AX-111005029	0.4202	72.3479
23	6B	AX-110952186	0.4202	72.3479
23	6B	AX-110442184	0.4202	72.3479
23	6B	AX-111578979	0.4202	72.3479
23	6B	AX-111232828	0.4202	72.3479
23	6B	AX-109104544	0.4202	72.3479
23	6B	AX-111779490	0.4202	72.3479
23	6B	AX-111079963	0.4202	72.3479
23	6B	AX-94513779	0.4202	72.3479
23	6B	AX-110638152	0.6356	72.9835
23	6B	AX-110428488	0.6356	72.9835
23	6B	AX-109939212	0.6356	72.9835
23	6B	AX-109450326	0.6356	72.9835
23	6B	AX-108876234	0.6356	72.9835
23	6B	AX-111609846	0.6356	72.9835
23	6B	AX-109295483	0.6356	72.9835

23	6B	AX-109892644	0.6356	72.9835
23	6B	AX-109342369	0.6356	72.9835
23	6B	AX-109451636	0.6356	72.9835
23	6B	AX-109404434	0.6356	72.9835
23	6B	AX-111136300	0.6356	72.9835
23	6B	AX-111005385	0.6356	72.9835
23	6B	AX-109487343	0.6356	72.9835
23	6B	AX-110472896	0.6356	72.9835
23	6B	AX-110494461	0.6356	72.9835
23	6B	AX-110996384	0.6356	72.9835
23	6B	AX-110520516	0.2083	73.1918
23	6B	AX-110500742	0.2083	73.1918
23	6B	AX-108851157	0.2083	73.1918
23	6B	AX-109039687	0.2083	73.1918
23	6B	AX-111223108	0.2083	73.1918
23	6B	AX-109052337	0.2083	73.1918
23	6B	AX-110576978	0.2083	73.1918
23	6B	AX-110038697	1.2823	74.4742
23	6B	AX-89357538	1.064	75.5381
23	6B	AX-110475129	0.844	76.3821
23	6B	AX-110198215	0.844	76.3821
23	6B	AX-110010008	0.844	76.3821
23	6B	AX-110003884	0.844	76.3821
23	6B	AX-109397478	0.844	76.3821
23	6B	AX-111109106	0.844	76.3821
23	6B	AX-109403674	0.844	76.3821
23	6B	AX-111567801	0.844	76.3821
23	6B	AX-110513697	0.844	76.3821
23	6B	AX-110412737	0.844	76.3821
23	6B	AX-109035988	0.844	76.3821
23	6B	AX-111546392	0.844	76.3821
23	6B	AX-111457109	0.844	76.3821
23	6B	AX-108884142	0.844	76.3821
23	6B	AX-111594061	0.844	76.3821
23	6B	AX-111677894	0.844	76.3821
23	6B	AX-111501351	0.844	76.3821
23	6B	AX-108967879	0.844	76.3821
23	6B	AX-109487902	0.844	76.3821
23	6B	AX-108786465	0.844	76.3821
23	6B	AX-109985262	0.844	76.3821
23	6B	AX-110489742	0.844	76.3821
23	6B	AX-111038892	0.844	76.3821
23	6B	AX-110909514	0.844	76.3821
23	6B	AX-110611616	0.844	76.3821

23	6B	AX-111650551	0.844	76.3821
23	6B	AX-109416493	0.844	76.3821
23	6B	AX-109599605	0.844	76.3821
23	6B	AX-108921741	0.844	76.3821
23	6B	AX-111614780	0.844	76.3821
23	6B	AX-110598414	0.844	76.3821
23	6B	AX-109360102	0.844	76.3821
23	6B	AX-110054669	0.2083	76.5904
23	6B	AX-108768271	0.2083	76.5904
23	6B	AX-110548059	0.2083	76.5904
23	6B	AX-108766037	0.2083	76.5904
23	6B	AX-109053263	0.2083	76.5904
23	6B	AX-110933626	0.4167	77.0071
23	6B	AX-86166312	0.4167	77.0071
23	6B	AX-108872199	0.4167	77.0071
23	6B	AX-110364554	0.4167	77.0071
23	6B	AX-110984386	0.4167	77.0071
23	6B	AX-109829873	0.4167	77.0071
23	6B	AX-111610327	0.4167	77.0071
23	6B	AX-109366962	0.4167	77.0071
23	6B	AX-109496448	0.4167	77.0071
23	6B	AX-108742506	0.4167	77.0071
23	6B	AX-111465031	0.4167	77.0071
23	6B	AX-109520609	0.4167	77.0071
23	6B	AX-111675916	0.4167	77.0071
23	6B	AX-109481828	0.4167	77.0071
23	6B	AX-109538060	0.4167	77.0071
23	6B	AX-110196364	0.4167	77.0071
23	6B	AX-108856152	0.4167	77.0071
23	6B	AX-111041754	0.4167	77.0071
23	6B	AX-109394865	0.4167	77.0071
23	6B	AX-108834761	0.4167	77.0071
23	6B	AX-111601332	0.4167	77.0071
23	6B	AX-109435918	0.6329	77.6401
23	6B	AX-111017192	0.6329	77.6401
23	6B	AX-110024963	0.6329	77.6401
23	6B	AX-111520648	0.6329	77.6401
23	6B	AX-109976062	0.6329	77.6401
23	6B	AX-111675950	0.6329	77.6401
23	6B	AX-111162839	0.6329	77.6401
23	6B	AX-111696768	1.9575	79.5976
23	6B	AX-111757493	1.9575	79.5976
23	6B	AX-110527810	1.9575	79.5976
23	6B	AX-111528935	1.9575	79.5976

23	6B	AX-108763452	1.9575	79.5976
23	6B	AX-108948208	1.9575	79.5976
23	6B	AX-109839343	1.9575	79.5976
23	6B	AX-111100724	1.9575	79.5976
23	6B	AX-109509816	1.9575	79.5976
23	6B	AX-110460977	1.9575	79.5976
23	6B	AX-111029754	1.9575	79.5976
23	6B	AX-109366225	1.9575	79.5976
23	6B	AX-109289083	1.9575	79.5976
23	6B	AX-111665260	1.9575	79.5976
23	6B	AX-109292022	0.4202	80.0178
23	6B	AX-109034932	0.4202	80.0178
23	6B	AX-111697339	0.4202	80.0178
23	6B	AX-111662897	0.4202	80.0178
23	6B	AX-111777434	0.4202	80.0178
23	6B	AX-112289029	0.4202	80.0178
23	6B	AX-109994687	0.4202	80.0178
23	6B	AX-109876892	0.4202	80.0178
23	6B	AX-111044027	0.2092	80.227
23	6B	AX-109335921	0.2092	80.227
23	6B	AX-110672397	0.2092	80.227
23	6B	AX-108859165	0.2092	80.227
23	6B	AX-108789589	0.2092	80.227
23	6B	AX-110611557	0.2092	80.227
23	6B	AX-111799370	0.2092	80.227
23	6B	AX-109410029	0.2092	80.227
23	6B	AX-109287224	0.2092	80.227
23	6B	AX-108972799	0.2092	80.227
23	6B	AX-110020285	0.2092	80.227
23	6B	AX-94981854	0.2092	80.227
23	6B	AX-109900030	4.6001	84.8271
23	6B	AX-111142565	2.1753	87.0023
23	6B	AX-110384871	2.1753	87.0023
23	6B	AX-110512495	0.2058	87.2081
23	6B	AX-109469027	0.2058	87.2081
23	6B	AX-110548110	0.2058	87.2081
23	6B	AX-108828297	1.4835	88.6916
23	6B	AX-108751809	1.4835	88.6916
23	6B	AX-109859491	1.4835	88.6916
23	6B	AX-110385245	1.4835	88.6916
23	6B	AX-111162124	1.2823	89.9739
23	6B	AX-111630862	2.4142	92.3881
23	6B	AX-109461489	2.4142	92.3881
23	6B	AX-111657524	0.4202	92.8083

23	6B	AX-111547303	0.4202	92.8083
23	6B	AX-109484896	0.4202	92.8083
23	6B	AX-109888530	0.2119	93.0201
23	6B	AX-110092774	2.4356	95.4557
23	6B	AX-109853121	0.6411	96.0967
23	6B	AX-108872892	1.3103	97.4071
23	6B	AX-110929675	0.4184	97.8255
23	6B	AX-109383520	0.4184	97.8255
23	6B	AX-109101466	0.4184	97.8255
23	6B	AX-111763420	0.4184	97.8255
23	6B	AX-108748222	11.1506	108.9761
23	6B	AX-109830719	11.1506	108.9761
23	6B	AX-110404840	11.1506	108.9761
23	6B	AX-111060484	11.1506	108.9761
23	6B	AX-108766416	11.1506	108.9761
23	6B	AX-108811786	11.1506	108.9761
23	6B	AX-110143566	4.3488	113.325
23	6B	AX-110420876	1.2934	114.6184
23	6B	AX-110383997	1.2934	114.6184
23	6B	AX-111020460	18.6333	133.2517
24	6D	AX-111026969	0	0
24	6D	AX-111505537	27.0451	27.0451
24	6D	AX-110420753	27.0451	27.0451
24	6D	AX-111084726	0.4184	27.4636
24	6D	AX-110535493	0.4184	27.4636
24	6D	AX-111193950	0.2083	27.6719
24	6D	AX-109419992	0.2083	27.8802
24	6D	AX-110382511	0.2083	27.8802
24	6D	AX-111085525	0.2083	27.8802
24	6D	AX-109353709	0.2083	27.8802
24	6D	AX-110274596	0.6329	28.5132
24	6D	AX-111484680	0.6329	28.5132
24	6D	AX-109846178	0.6329	28.5132
24	6D	AX-111056746	0.6329	28.5132
24	6D	AX-111674978	0.6329	28.5132
24	6D	AX-110496142	0.6329	28.5132
24	6D	AX-111450772	0.6329	28.5132
24	6D	AX-111154750	0.6329	28.5132
24	6D	AX-109347966	0.422	28.9351
24	6D	AX-110065704	0.422	28.9351
24	6D	AX-111379959	0.6329	29.5681
24	6D	AX-110667224	4.579	34.147
24	6D	AX-109852543	13.9904	48.1374
24	6D	AX-111571048	1.7474	49.8849

24	6D	AX-110469783	1.064	50.9489
24	6D	AX-109850104	1.2769	52.2257
24	6D	AX-109412721	0.4184	52.6441
24	6D	AX-89643022	1.9575	54.6017
24	6D	AX-109251918	1.0778	55.6794
24	6D	AX-110066157	0.422	56.1014
24	6D	AX-110261831	0.6466	56.748
24	6D	AX-109280994	6.5913	63.3393
24	6D	AX-111361676	3.3683	66.7076
24	6D	AX-110334567	3.3683	66.7076
24	6D	AX-111602987	3.3683	66.7076
24	6D	AX-111761852	1.5156	68.2232
24	6D	AX-111780375	0.8512	69.0744
24	6D	AX-109521374	0.8512	69.0744
24	6D	AX-110618611	0.2101	69.2845
24	6D	AX-109896910	0.2101	69.2845
24	6D	AX-109705668	0.4255	69.71
24	6D	AX-109534413	0.8585	70.5684
24	6D	AX-111104259	2.6574	73.2258
24	6D	AX-110558093	1.5156	74.7414
24	6D	AX-111168056	1.5156	74.7414
24	6D	AX-110416062	1.0731	75.8146
24	6D	AX-110636525	1.0731	75.8146
24	6D	AX-111530024	1.0731	75.8146
24	6D	AX-110485927	2.2237	78.0382
24	6D	AX-110989969	0.4292	78.4674
24	6D	AX-108821954	0.4292	78.4674
24	6D	AX-89508120	0.4237	78.8912
24	6D	AX-111931231	0.6411	79.5322
24	6D	AX-110950957	0.6411	79.5322
24	6D	AX-111285003	0.6411	79.5322
24	6D	AX-109393921	0.2119	79.7441
24	6D	AX-109891051	0.6411	80.3852
24	6D	AX-110711001	1.0731	81.4583
24	6D	AX-108933182	0.2092	81.6675
24	6D	AX-110564593	0.6303	82.2978
24	6D	AX-110431664	0.8512	83.1489
24	6D	AX-109821513	0.8548	84.0037
24	6D	AX-110871088	2.6932	86.6969
24	6D	AX-111716941	1.0731	87.77
24	6D	AX-109158832	1.949	89.7191
24	6D	AX-109088524	0.6356	90.3547
24	6D	AX-110425031	0.422	90.7766
24	6D	AX-111347442	4.2898	95.0664

24	6D	AX-95658492	4.2898	95.0664
24	6D	AX-111263460	0.6303	95.6967
24	6D	AX-108794230	0.6303	95.6967
24	6D	AX-110962527	0.2155	95.9122
24	6D	AX-109331000	0.2165	96.1287
24	6D	AX-109775045	0.2101	96.3388
24	6D	AX-110327591	0.2083	96.5471
24	6D	AX-109509128	1.5026	98.0497
24	6D	AX-110612307	1.5026	98.0497
24	6D	AX-108849732	1.5026	98.0497
24	6D	AX-110831860	1.5026	98.0497
24	6D	AX-109911446	1.5026	98.0497
24	6D	AX-109465489	3.9804	102.03
24	6D	AX-110571231	0.6579	102.688
24	6D	AX-110584360	0.6579	102.688
24	6D	AX-109308622	2.6692	105.3572
24	6D	AX-109128671	0.8659	106.2231
24	6D	AX-89564650	1.7628	107.9859
24	6D	AX-109532919	5.7671	113.753
24	6D	AX-108802393	1.3103	115.0633
24	6D	AX-111615506	1.3103	115.0633
24	6D	AX-111098417	1.3103	115.0633
24	6D	AX-110452797	0.2146	115.2779
24	6D	AX-109726088	0.2146	115.2779
24	6D	AX-110662688	1.7628	117.0408
24	6D	AX-111550880	0.4348	117.4755
24	6D	AX-111189875	1.0967	118.5722
24	6D	AX-110956800	3.5936	122.1658
24	6D	AX-109815305	3.5936	122.1658
24	6D	AX-109840078	3.5936	122.1658
24	6D	AX-109200636	7.0512	129.217
24	6D	AX-110438066	1.0731	130.2902
24	6D	AX-109898786	0.211	130.5011
24	6D	AX-109922847	1.0871	131.5883
24	6D	AX-110392637	1.5222	133.1105
24	6D	AX-110969004	1.5222	133.1105
24	6D	AX-109194293	1.5222	133.1105
24	6D	AX-111121905	1.5222	133.1105
24	6D	AX-108877832	1.7101	134.8205
24	6D	AX-111419172	1.7101	134.8205
24	6D	AX-110328874	1.7101	134.8205
24	6D	AX-109391798	1.7101	134.8205
24	6D	AX-109445592	9.4238	144.2443
24	6D	AX-108966812	1.9575	146.2018

24	6D	AX-110435301	0.6356	146.8374
24	6D	AX-108796868	4.8093	151.6467
24	6D	AX-110336440	24.8419	176.4887
24	6D	AX-111511882	1.7398	178.2285
24	6D	AX-110571474	1.7398	178.2285
24	6D	AX-95629093	1.7398	178.2285
24	6D	AX-109838417	1.7398	178.2285
24	6D	AX-109975102	1.7398	178.2285
24	6D	AX-108782483	1.7398	178.2285
24	6D	AX-110290567	1.7398	178.2285
24	6D	AX-109515038	1.7398	178.2285
24	6D	AX-108880282	1.7398	178.2285
24	6D	AX-110370481	1.7398	178.2285
24	6D	AX-110391379	1.7398	178.2285
24	6D	AX-110389623	1.7398	178.2285
24	6D	AX-111137381	1.7398	178.2285
24	6D	AX-94483986	1.7398	178.2285
24	6D	AX-111096954	1.7398	178.2285
24	6D	AX-111576284	1.7398	178.2285
24	6D	AX-111491806	1.7398	178.2285
24	6D	AX-111876852	1.7398	178.2285
24	6D	AX-111091884	1.7398	178.2285
24	6D	AX-108981000	1.7398	178.2285
24	6D	AX-111463606	0.2092	178.4377
24	6D	AX-110616686	0.2092	178.4377
24	6D	AX-110378727	0.2092	178.4377
24	6D	AX-109338425	0.2092	178.4377
24	6D	AX-110436926	0.2092	178.4377
24	6D	AX-108800206	0.2092	178.4377
24	6D	AX-108831730	1.2878	179.7255
24	6D	AX-109438733	0.422	180.1475
24	6D	AX-108809133	0.422	180.1475
24	6D	AX-109878889	0.422	180.1475
24	6D	AX-110827873	0.422	180.1475
24	6D	AX-110242843	0.422	180.1475
24	6D	AX-109437953	0.422	180.1475
24	6D	AX-111450561	0.422	180.1475
24	6D	AX-110416139	0.422	180.1475
24	6D	AX-111511880	0.2092	180.3567
24	6D	AX-108945214	0.2092	180.3567
24	6D	AX-111362420	0.2092	180.3567
24	6D	AX-110048049	0.2092	180.3567
24	6D	AX-111041573	0.2092	180.3567
24	6D	AX-110021686	0.2119	180.5686

24	6D	AX-109898436	0.2119	180.5686
24	6D	AX-109937951	0.4255	180.9941
24	6D	AX-111121480	0.4255	180.9941
24	6D	AX-109969243	4.0814	185.0755
24	6D	AX-109002268	4.0814	185.0755
24	6D	AX-94541184	4.0814	185.0755
24	6D	AX-111701850	4.0814	185.0755
24	6D	AX-109448694	4.0814	185.0755
24	6D	AX-109741930	0.4238	185.4993
24	6D	AX-108728939	0.4238	185.4993
24	6D	AX-110470922	0.4238	185.4993
24	6D	AX-110077105	0.6383	186.1376
24	6D	AX-108854329	0.6383	186.1376
24	6D	AX-108836577	0.6383	186.1376
24	6D	AX-108879722	0.6383	186.1376
24	6D	AX-110955035	0.211	186.3486
24	6D	AX-109303910	0.211	186.3486
24	6D	AX-110962508	0.211	186.3486
24	6D	AX-109846912	0.4238	186.7723
24	6D	AX-110099207	0.4238	186.7723
24	6D	AX-111041495	0.4238	186.7723
24	6D	AX-111369489	0.4238	186.7723
24	6D	AX-109181871	0.4238	186.7723
24	6D	AX-111511037	0.4238	186.7723
24	6D	AX-110969271	0.4238	186.7723
24	6D	AX-109345567	0.4238	186.7723
24	6D	AX-94574625	0.4238	186.7723
24	6D	AX-110461823	0.4238	186.7723
24	6D	AX-109436099	0.4238	186.7723
24	6D	AX-109196900	0.4238	186.7723
24	6D	AX-109318885	0.4238	186.7723
24	6D	AX-108800457	0.4238	186.7723
24	6D	AX-111709193	0.4238	186.7723
24	6D	AX-110905685	0.4238	186.7723
24	6D	AX-111333213	0.4238	186.7723
24	6D	AX-108966945	0.4238	186.7723
24	6D	AX-110960339	0.4238	186.7723
24	6D	AX-111865465	0.4238	186.7723
24	6D	AX-89577787	0.4238	186.7723
24	6D	AX-109324109	0.4238	186.7723
24	6D	AX-109362430	0.4238	186.7723
24	6D	AX-110402455	0.4238	186.7723
24	6D	AX-110534358	0.4238	186.7723
24	6D	AX-110387710	0.4238	186.7723

24	6D	AX-109335063	0.4238	186.7723
24	6D	AX-109968823	0.4238	186.7723
24	6D	AX-111015834	0.4238	186.7723
24	6D	AX-111867096	0.4238	186.7723
24	6D	AX-109968429	0.4238	186.7723
24	6D	AX-109142085	0.4238	186.7723
24	6D	AX-108935293	0.4238	186.7723
24	6D	AX-111708702	0.4238	186.7723
24	6D	AX-109360092	0.4238	186.7723
24	6D	AX-108954572	0.4238	186.7723
24	6D	AX-111259440	0.4238	186.7723
24	6D	AX-110289046	0.4238	186.7723
24	6D	AX-110001904	0.4238	186.7723
24	6D	AX-110818116	0.4238	186.7723
24	6D	AX-110232069	0.4238	186.7723
24	6D	AX-110940681	0.4238	186.7723
24	6D	AX-109901947	0.4238	186.7723
24	6D	AX-95238263	0.4238	186.7723
24	6D	AX-111419718	1.9747	188.747
24	6D	AX-109328766	1.9747	188.747
24	6D	AX-111043108	1.9747	188.747
24	6D	AX-110123236	1.9747	188.747
24	6D	AX-110830333	1.9747	188.747
24	6D	AX-109831577	1.9747	188.747
24	6D	AX-108917731	0.4255	189.1726
24	6D	AX-109886919	0.4255	189.1726
24	6D	AX-111564304	0.4255	189.1726
24	6D	AX-110612644	0.4255	189.1726
24	6D	AX-110436530	0.4255	189.1726
24	6D	AX-109547156	1.5289	190.7014
24	6D	AX-111184066	0.4274	191.1288
24	6D	AX-108747095	0.8697	191.9985
25	7A	AX-108838721	0	0
25	7A	AX-110400351	0.422	0.422
25	7A	AX-108928821	0.2101	0.632
25	7A	AX-111606970	0.4202	1.0522
25	7A	AX-110467153	0.2083	1.2605
25	7A	AX-108973008	0.4184	1.679
25	7A	AX-110467450	0.4184	1.679
25	7A	AX-111076418	0.4184	1.679
25	7A	AX-110441820	0.4184	1.679
25	7A	AX-111464820	0.4202	2.0992
25	7A	AX-110454453	0.2128	2.3119
25	7A	AX-108812645	0.2137	2.5256

25	7A	AX-111128855	0.6356	3.1612
25	7A	AX-110422738	0.6356	3.1612
25	7A	AX-109272090	0.6356	3.1612
25	7A	AX-111576650	0.6356	3.1612
25	7A	AX-111002562	0.8475	4.0088
25	7A	AX-108857941	0.8475	4.0088
25	7A	AX-108732288	0.8475	4.0088
25	7A	AX-110426647	1.3396	5.3484
25	7A	AX-110512897	1.1064	6.4548
25	7A	AX-108824499	1.1064	6.4548
25	7A	AX-108747076	1.1064	6.4548
25	7A	AX-110482447	0.211	6.6657
25	7A	AX-111697018	0.4237	7.0895
25	7A	AX-111697683	4.6214	11.7109
25	7A	AX-111597107	0.211	11.9218
25	7A	AX-110394120	0.6329	12.5548
25	7A	AX-109920906	0.6329	12.5548
25	7A	AX-111073167	0.6329	12.5548
25	7A	AX-111001599	0.6329	12.5548
25	7A	AX-108947924	0.6329	12.5548
25	7A	AX-110500856	0.6329	12.5548
25	7A	AX-109879380	0.8404	13.3952
25	7A	AX-109968537	0.8404	13.3952
25	7A	AX-108794630	0.4167	13.8119
25	7A	AX-109431738	0.4202	14.2321
25	7A	AX-109452823	0.6303	14.8624
25	7A	AX-110512712	2.1472	17.0096
25	7A	AX-109107488	0.625	17.6346
25	7A	AX-110152768	0.625	17.6346
25	7A	AX-109288783	0.625	17.6346
25	7A	AX-109869706	0.625	17.6346
25	7A	AX-110370362	0.625	17.6346
25	7A	AX-109470256	0.625	17.6346
25	7A	AX-111152694	0.6277	18.2623
25	7A	AX-110469352	0.6277	18.2623
25	7A	AX-109313511	0.6277	18.2623
25	7A	AX-111213259	1.0595	19.3218
25	7A	AX-108830715	1.0595	19.3218
25	7A	AX-111002491	1.0595	19.3218
25	7A	AX-111013289	1.0595	19.3218
25	7A	AX-108921078	1.055	20.3768
25	7A	AX-108791173	1.7101	22.0868
25	7A	AX-110417087	1.0595	23.1463
25	7A	AX-109352761	1.0595	23.1463

25	7A	AX-110503812	1.064	24.2103
25	7A	AX-111605589	1.064	24.2103
25	7A	AX-110395966	1.064	24.2103
25	7A	AX-108943065	1.064	24.2103
25	7A	AX-109055212	1.064	24.2103
25	7A	AX-109920626	1.064	24.2103
25	7A	AX-110434010	1.064	24.2103
25	7A	AX-110515818	1.064	24.2103
25	7A	AX-111114151	1.064	24.2103
25	7A	AX-111634177	1.064	24.2103
25	7A	AX-111564859	0.4184	24.6287
25	7A	AX-111116295	0.4184	24.6287
25	7A	AX-110973510	0.4184	24.6287
25	7A	AX-111488311	2.1659	26.7946
25	7A	AX-109916402	2.5885	29.3831
25	7A	AX-109512172	2.5885	29.3831
25	7A	AX-109334479	2.5885	29.3831
25	7A	AX-109844463	2.5885	29.3831
25	7A	AX-110464581	2.5885	29.3831
25	7A	AX-110018190	2.5885	29.3831
25	7A	AX-109290128	2.5885	29.3831
25	7A	AX-108810487	2.5885	29.3831
25	7A	AX-111149652	2.5885	29.3831
25	7A	AX-110960601	0.8334	30.2165
25	7A	AX-110109325	0.8334	30.2165
25	7A	AX-108775209	0.8334	30.2165
25	7A	AX-108791367	0.8334	30.2165
25	7A	AX-108824676	0.2066	30.4231
25	7A	AX-109984821	0.4167	30.8398
25	7A	AX-109891183	0.2075	31.0473
25	7A	AX-109289312	10.0848	41.1321
25	7A	AX-109492550	1.7101	42.8422
25	7A	AX-110429273	3.785	46.6272
25	7A	AX-110466947	0.625	47.2522
25	7A	AX-89542612	1.2661	48.5183
25	7A	AX-110377311	0.2075	48.7258
25	7A	AX-109853225	0.2075	48.7258
25	7A	AX-109527138	4.558	53.2838
25	7A	AX-111473783	0.8475	54.1313
25	7A	AX-109866298	9.263	63.3943
25	7A	AX-109971619	0.2092	63.6035
25	7A	AX-109913433	0.2092	63.6035
25	7A	AX-111639706	0.4202	64.0237
25	7A	AX-111699124	0.4202	64.4439

25	7A	AX-110011761	1.3103	65.7542
25	7A	AX-110109492	2.2138	67.968
25	7A	AX-108740541	9.1701	77.1381
25	7A	AX-108878957	0.2066	77.3447
25	7A	AX-109944192	0.2066	77.3447
25	7A	AX-109293159	0.2066	77.3447
25	7A	AX-108830189	0.2066	77.3447
25	7A	AX-110565150	0.2066	77.3447
25	7A	AX-110971829	0.2066	77.3447
25	7A	AX-109837966	0.2066	77.3447
25	7A	AX-110014864	0.2066	77.3447
25	7A	AX-110968250	0.2066	77.3447
25	7A	AX-108887847	0.2066	77.3447
25	7A	AX-108859361	0.2066	77.3447
25	7A	AX-110420796	0.2066	77.3447
25	7A	AX-109491905	0.2066	77.3447
25	7A	AX-109603569	0.2066	77.3447
25	7A	AX-111551571	0.2066	77.3447
25	7A	AX-110434970	0.2066	77.3447
25	7A	AX-110002337	0.2066	77.3447
25	7A	AX-109851026	0.2066	77.3447
25	7A	AX-110362441	0.2066	77.3447
25	7A	AX-110952157	0.2066	77.3447
25	7A	AX-111586562	0.2066	77.3447
25	7A	AX-108937340	0.2066	77.3447
25	7A	AX-110397134	0.2066	77.3447
25	7A	AX-109300677	0.2066	77.3447
25	7A	AX-111662651	0.2066	77.3447
25	7A	AX-111154340	0.2066	77.3447
25	7A	AX-108725239	0.2066	77.3447
25	7A	AX-111284705	0.2066	77.3447
25	7A	AX-110127149	0.2066	77.3447
25	7A	AX-94755613	0.2066	77.3447
25	7A	AX-109540788	0.2066	77.3447
25	7A	AX-109964200	0.2066	77.3447
25	7A	AX-109973472	0.2066	77.3447
25	7A	AX-110447646	0.2066	77.5514
25	7A	AX-111085055	0.2066	77.5514
25	7A	AX-110962367	0.2066	77.5514
25	7A	AX-108820762	0.2066	77.5514
25	7A	AX-109970826	0.2066	77.5514
25	7A	AX-111536100	0.2066	77.5514
25	7A	AX-110396771	0.2066	77.5514
25	7A	AX-109504503	0.2066	77.5514

25	7A	AX-109474489	0.2066	77.5514
25	7A	AX-111485137	0.2066	77.5514
25	7A	AX-110538185	0.2066	77.5514
25	7A	AX-109418588	0.2066	77.5514
25	7A	AX-111523931	0.2066	77.5514
25	7A	AX-111120480	0.2066	77.5514
25	7A	AX-110426981	0.2066	77.5514
25	7A	AX-111453793	0.2066	77.5514
25	7A	AX-109967664	0.2066	77.5514
25	7A	AX-108905169	0.2066	77.5514
25	7A	AX-109429555	0.2066	77.5514
25	7A	AX-109314571	0.2066	77.5514
25	7A	AX-111460177	0.2066	77.5514
25	7A	AX-109303551	0.2066	77.5514
25	7A	AX-109855326	0.2066	77.5514
25	7A	AX-111053964	0.2066	77.5514
25	7A	AX-110512318	0.2066	77.5514
25	7A	AX-110122450	0.2066	77.5514
25	7A	AX-111652639	0.2066	77.5514
25	7A	AX-110711022	0.2066	77.5514
25	7A	AX-108833648	0.2066	77.5514
25	7A	AX-109448723	0.2066	77.5514
25	7A	AX-109297928	0.2066	77.5514
25	7A	AX-109982987	0.2066	77.5514
25	7A	AX-111143419	0.2066	77.5514
25	7A	AX-110054283	0.2066	77.5514
25	7A	AX-111650336	0.2066	77.5514
25	7A	AX-108938437	0.2066	77.5514
25	7A	AX-108884040	0.2066	77.5514
25	7A	AX-111536482	0.2066	77.5514
25	7A	AX-111171332	0.2066	77.5514
25	7A	AX-108871582	0.2066	77.5514
25	7A	AX-109978887	0.2066	77.5514
25	7A	AX-109335420	0.2066	77.5514
25	7A	AX-109954946	0.2066	77.5514
25	7A	AX-111493829	0.2066	77.5514
25	7A	AX-109556972	0.2066	77.5514
25	7A	AX-109966388	0.2066	77.5514
25	7A	AX-108845152	0.2066	77.5514
25	7A	AX-111643658	0.2066	77.5514
25	7A	AX-111764490	0.844	78.3953
25	7A	AX-108829387	0.844	78.3953
25	7A	AX-110970302	0.844	78.3953
25	7A	AX-109931165	0.844	78.3953

25	7A	AX-108781261	0.844	78.3953
25	7A	AX-108743318	0.844	78.3953
25	7A	AX-110360771	0.844	78.3953
25	7A	AX-109600915	3.1013	81.4966
25	7A	AX-111079249	3.1013	81.4966
25	7A	AX-109617751	3.1013	81.4966
25	7A	AX-111462090	3.1013	81.4966
25	7A	AX-109593689	3.1013	81.4966
25	7A	AX-111516679	3.1013	81.4966
25	7A	AX-108730514	3.1013	81.4966
25	7A	AX-108902056	3.1013	81.4966
25	7A	AX-111469799	3.1013	81.4966
25	7A	AX-108839309	3.1013	81.4966
25	7A	AX-109927710	3.1013	81.4966
25	7A	AX-111582660	3.1013	81.4966
25	7A	AX-111157528	0.2075	81.7041
25	7A	AX-111113109	0.2075	81.7041
25	7A	AX-111619425	1.2661	82.9702
25	7A	AX-110505644	1.2661	82.9702
25	7A	AX-110128705	1.2661	82.9702
25	7A	AX-109388661	1.2661	82.9702
25	7A	AX-94797893	5.0168	87.987
25	7A	AX-109865315	5.0168	87.987
25	7A	AX-110090057	5.0168	87.987
25	7A	AX-108843529	5.0168	87.987
25	7A	AX-109930452	5.0168	87.987
25	7A	AX-109868790	5.0168	87.987
25	7A	AX-111059189	5.0168	87.987
25	7A	AX-108885515	5.0168	87.987
25	7A	AX-109469938	5.0168	87.987
25	7A	AX-109383597	5.0168	87.987
25	7A	AX-111644940	5.0168	87.987
25	7A	AX-110502666	5.0168	87.987
25	7A	AX-109513827	5.0168	87.987
25	7A	AX-111570877	5.0168	87.987
25	7A	AX-111455372	5.0168	87.987
25	7A	AX-109839906	5.0168	87.987
25	7A	AX-110422885	5.0168	87.987
25	7A	AX-111202438	5.0168	87.987
25	7A	AX-111151663	2.1753	90.1623
25	7A	AX-110534525	1.5156	91.6779
25	7A	AX-109848988	0.4237	92.1016
25	7A	AX-109389234	0.4237	92.1016
25	7A	AX-110088744	0.211	92.3126

25	7A	AX-109302546	0.4274	92.7399
25	7A	AX-108771714	0.4237	93.1637
25	7A	AX-108873906	1.9747	95.1384
25	7A	AX-110467294	4.1379	99.2762
25	7A	AX-110939226	0.2092	99.4854
25	7A	AX-108792253	0.2092	99.4854
25	7A	AX-109911760	0.2092	99.4854
25	7A	AX-108812384	0.2092	99.4854
25	7A	AX-109329195	0.2092	99.4854
25	7A	AX-109583395	0.2092	99.4854
25	7A	AX-110533843	2.4248	101.9102
25	7A	AX-110987482	2.4248	101.9102
25	7A	AX-109902411	0.211	102.1212
25	7A	AX-109624261	0.211	102.1212
25	7A	AX-111108929	1.7398	103.8611
25	7A	AX-109449565	1.9747	105.8358
25	7A	AX-110477239	0.8697	106.7054
25	7A	AX-110712776	0.4367	107.1421
25	7A	AX-109335244	3.1573	110.2995
25	7A	AX-108853295	3.1573	110.2995
25	7A	AX-111668009	3.1573	110.2995
25	7A	AX-110562934	3.1573	110.2995
25	7A	AX-110166014	3.1573	110.2995
25	7A	AX-110686503	3.1573	110.2995
25	7A	AX-110041698	3.1573	110.2995
25	7A	AX-109958913	3.1573	110.2995
25	7A	AX-108906781	3.1573	110.2995
25	7A	AX-111020859	3.1573	110.2995
25	7A	AX-109940462	1.0731	111.3726
25	7A	AX-109334274	1.0731	111.3726
25	7A	AX-109483253	1.0731	111.3726
25	7A	AX-109963196	1.0731	111.3726
25	7A	AX-108941518	1.0731	111.3726
25	7A	AX-109418641	1.0731	111.3726
25	7A	AX-111458361	1.0731	111.3726
25	7A	AX-108882533	1.0731	111.3726
25	7A	AX-110121765	1.0731	111.3726
25	7A	AX-111016140	1.0731	111.3726
25	7A	AX-109443884	0.422	111.7945
25	7A	AX-111491167	0.422	111.7945
25	7A	AX-111698786	0.422	111.7945
25	7A	AX-110411338	0.2101	112.0046
25	7A	AX-110058146	0.2101	112.0046
25	7A	AX-108881025	0.2101	112.0046

25	7A	AX-110960372	0.2101	112.0046
25	7A	AX-111610354	0.2101	112.0046
25	7A	AX-109354785	0.2101	112.0046
25	7A	AX-109864896	0.2101	112.0046
25	7A	AX-108892152	0.2101	112.0046
25	7A	AX-108764676	0.2101	112.0046
25	7A	AX-110987624	0.2101	112.0046
25	7A	AX-108734541	0.2101	112.0046
25	7A	AX-108805707	0.2101	112.0046
25	7A	AX-109302004	0.2092	112.2138
25	7A	AX-109312766	0.2092	112.2138
25	7A	AX-110951250	0.2092	112.2138
25	7A	AX-109958517	0.2092	112.2138
25	7A	AX-110131498	0.2092	112.2138
25	7A	AX-111038593	0.2092	112.2138
25	7A	AX-109438155	0.2092	112.2138
25	7A	AX-109504905	0.2092	112.2138
25	7A	AX-108958957	0.2092	112.2138
25	7A	AX-110487843	0.2092	112.2138
25	7A	AX-111087383	0.2092	112.2138
25	7A	AX-108863658	0.2092	112.2138
25	7A	AX-110538456	0.2092	112.2138
25	7A	AX-111218946	0.2092	112.2138
25	7A	AX-111106374	0.2092	112.2138
25	7A	AX-111031278	0.2092	112.2138
25	7A	AX-110555624	0.2092	112.2138
25	7A	AX-111691966	0.2092	112.2138
25	7A	AX-110599789	0.2092	112.2138
25	7A	AX-111675742	0.2092	112.2138
25	7A	AX-110173198	0.2092	112.2138
25	7A	AX-111637043	0.2092	112.2138
25	7A	AX-110122618	0.2092	112.2138
25	7A	AX-108921924	0.2092	112.2138
25	7A	AX-110090122	0.2092	112.2138
25	7A	AX-109438860	0.2092	112.2138
25	7A	AX-108938382	0.2092	112.2138
25	7A	AX-108751506	0.2092	112.2138
25	7A	AX-109919252	0.2092	112.2138
25	7A	AX-111449807	0.2092	112.2138
25	7A	AX-111028721	0.2092	112.2138
25	7A	AX-110023493	0.2092	112.2138
25	7A	AX-108798419	0.2092	112.2138
25	7A	AX-108794924	0.2092	112.2138
25	7A	AX-108799491	0.2092	112.2138

25	7A	AX-111527171	0.2092	112.2138
25	7A	AX-109894072	0.2092	112.2138
25	7A	AX-109627042	0.2092	112.2138
25	7A	AX-111655044	0.2092	112.2138
25	7A	AX-110551499	0.2092	112.2138
25	7A	AX-111460714	0.2092	112.2138
25	7A	AX-111572368	0.2092	112.2138
25	7A	AX-110011750	0.2092	112.2138
25	7A	AX-109465910	0.2092	112.2138
25	7A	AX-111654708	0.2092	112.2138
25	7A	AX-94724752	0.2092	112.2138
25	7A	AX-110462611	0.2092	112.2138
25	7A	AX-111573458	0.2092	112.2138
25	7A	AX-110664075	0.4202	112.634
25	7A	AX-111135268	0.4202	112.634
25	7A	AX-110591751	0.4202	112.634
25	7A	AX-109922456	0.4202	112.634
25	7A	AX-111697450	0.4202	112.634
25	7A	AX-109320362	0.4202	112.634
25	7A	AX-108779950	0.4202	112.634
25	7A	AX-109412011	0.4202	112.634
25	7A	AX-109455860	0.4202	112.634
25	7A	AX-110411112	0.4202	112.634
25	7A	AX-111510750	0.4202	112.634
25	7A	AX-111475110	0.4202	112.634
25	7A	AX-109871720	0.4202	112.634
25	7A	AX-111070378	0.4202	112.634
25	7A	AX-111247753	0.4202	112.634
25	7A	AX-109993614	0.4202	112.634
25	7A	AX-110430246	0.4202	112.634
25	7A	AX-110494040	0.4202	112.634
25	7A	AX-109273401	0.4202	112.634
25	7A	AX-108951250	0.4202	112.634
25	7A	AX-111034531	0.4202	112.634
25	7A	AX-110011564	0.4202	112.634
25	7A	AX-111055662	0.4202	112.634
25	7A	AX-110660898	0.4202	112.634
25	7A	AX-111636150	0.4202	112.634
25	7A	AX-109362221	0.4202	112.634
25	7A	AX-108934178	0.4202	112.634
25	7A	AX-110956250	0.4202	112.634
25	7A	AX-111081295	0.4202	112.634
25	7A	AX-111236006	0.4202	112.634
25	7A	AX-111262708	0.4202	112.634

25	7A	AX-111193397	0.4202	112.634
25	7A	AX-110386466	0.4202	112.634
25	7A	AX-110369401	0.4202	112.634
25	7A	AX-111073226	0.4202	112.634
25	7A	AX-109849741	0.4202	112.634
25	7A	AX-109897119	0.4202	112.634
25	7A	AX-108789462	0.4202	112.634
25	7A	AX-110982799	0.4202	112.634
25	7A	AX-108955077	0.4202	112.634
25	7A	AX-109505130	0.4202	112.634
25	7A	AX-111586310	0.4202	112.634
25	7A	AX-109356822	0.4202	112.634
25	7A	AX-110020391	0.4202	112.634
25	7A	AX-111039707	0.4202	112.634
25	7A	AX-111113709	0.4202	112.634
25	7A	AX-109925767	0.4202	112.634
25	7A	AX-110037258	0.4202	112.634
25	7A	AX-109963107	0.4202	112.634
25	7A	AX-108942861	0.4202	112.634
25	7A	AX-108751262	0.4202	112.634
25	7A	AX-111614884	0.4202	112.634
25	7A	AX-110045121	0.4202	112.634
25	7A	AX-111521081	0.4202	112.634
25	7A	AX-109583052	0.4202	112.634
25	7A	AX-111127504	0.4202	112.634
25	7A	AX-109961712	0.4202	112.634
25	7A	AX-109576825	0.4202	112.634
25	7A	AX-111650081	0.4202	112.634
25	7A	AX-111636354	0.4202	112.634
25	7A	AX-108865737	0.4202	112.634
25	7A	AX-110923765	0.4202	112.634
25	7A	AX-111591978	0.4202	112.634
25	7A	AX-111095734	0.4202	112.634
25	7A	AX-109443835	0.4202	112.634
25	7A	AX-109998119	0.4202	112.634
25	7A	AX-108789888	0.4202	112.634
25	7A	AX-109038137	0.4202	112.634
25	7A	AX-111101255	0.4202	112.634
25	7A	AX-109447701	0.4202	112.634
25	7A	AX-111039815	0.4202	112.634
25	7A	AX-109295485	0.4202	112.634
25	7A	AX-109379424	0.4202	112.634
25	7A	AX-110003007	0.4202	112.634
25	7A	AX-110496630	0.4202	112.634

25	7A	AX-109397917	0.4202	112.634
25	7A	AX-110602623	0.4202	112.634
25	7A	AX-110577867	0.4202	112.634
25	7A	AX-109504313	0.4202	112.634
25	7A	AX-109032854	0.4202	112.634
25	7A	AX-108857650	0.4202	112.634
25	7A	AX-108867568	0.4202	112.634
25	7A	AX-111467898	0.4202	112.634
25	7A	AX-111531170	0.4202	112.634
25	7A	AX-111145088	0.4202	112.634
25	7A	AX-109893366	0.4202	112.634
25	7A	AX-108779997	0.4202	112.634
25	7A	AX-109434485	0.4202	112.634
25	7A	AX-110518839	0.4202	112.634
25	7A	AX-110611360	0.4202	112.634
25	7A	AX-109059430	0.4202	112.634
25	7A	AX-110444154	0.4202	112.634
25	7A	AX-109861451	0.4202	112.634
25	7A	AX-109379483	0.4202	112.634
25	7A	AX-108799356	0.4202	112.634
25	7A	AX-109280076	0.4202	112.634
25	7A	AX-109958345	0.4202	112.634
25	7A	AX-110974324	0.4202	112.634
25	7A	AX-110601057	0.4202	112.634
25	7A	AX-108948827	0.4202	112.634
25	7A	AX-111029151	0.4202	112.634
25	7A	AX-110575134	0.4202	112.634
25	7A	AX-109367795	0.4202	112.634
25	7A	AX-109921242	0.4202	112.634
25	7A	AX-111129019	0.4202	112.634
25	7A	AX-111465512	0.4202	112.634
25	7A	AX-111477504	0.4202	112.634
25	7A	AX-111682197	0.4202	112.634
25	7A	AX-109848140	0.6411	113.2751
25	7A	AX-111476958	0.6411	113.2751
25	7A	AX-111047991	0.4274	113.7024
25	7A	AX-111073918	0.4274	113.7024
25	7A	AX-111563184	0.4274	113.7024
25	7A	AX-110442627	0.4274	113.7024
25	7A	AX-110583181	0.4274	113.7024
25	7A	AX-111179289	0.211	113.9134
25	7A	AX-110441972	0.211	113.9134
25	7A	AX-110385412	0.211	113.9134
25	7A	AX-111567521	0.211	113.9134

25	7A	AX-108896463	0.211	113.9134
25	7A	AX-109448957	0.211	113.9134
25	7A	AX-110495496	0.211	113.9134
25	7A	AX-108787348	0.211	113.9134
25	7A	AX-109888624	0.211	113.9134
25	7A	AX-110525330	0.211	113.9134
25	7A	AX-111040747	0.211	113.9134
25	7A	AX-110038044	0.211	113.9134
25	7A	AX-109361745	0.211	113.9134
25	7A	AX-111576358	0.211	113.9134
25	7A	AX-110120010	0.211	113.9134
25	7A	AX-111649077	0.211	113.9134
25	7A	AX-109447262	0.211	113.9134
25	7A	AX-109349746	0.211	113.9134
25	7A	AX-111469123	0.211	113.9134
25	7A	AX-111089323	0.211	113.9134
25	7A	AX-110499334	0.211	113.9134
25	7A	AX-110580257	0.211	113.9134
25	7A	AX-108798436	0.211	113.9134
25	7A	AX-110539439	0.211	113.9134
25	7A	AX-110422045	0.211	113.9134
25	7A	AX-109969335	0.211	113.9134
25	7A	AX-109845972	0.211	113.9134
25	7A	AX-111497147	0.211	113.9134
25	7A	AX-111550143	0.211	113.9134
25	7A	AX-109988204	0.211	113.9134
25	7A	AX-110479288	0.211	113.9134
25	7A	AX-109477251	0.211	113.9134
25	7A	AX-109958468	0.211	113.9134
25	7A	AX-109919591	0.211	113.9134
25	7A	AX-109539534	0.211	113.9134
25	7A	AX-109586298	0.211	113.9134
25	7A	AX-109436688	0.211	113.9134
25	7A	AX-109333979	0.211	113.9134
25	7A	AX-109406746	0.211	113.9134
25	7A	AX-109041107	0.211	113.9134
25	7A	AX-110090793	0.211	113.9134
25	7A	AX-111543103	0.211	113.9134
25	7A	AX-110463440	0.211	113.9134
25	7A	AX-111617422	0.211	113.9134
25	7A	AX-111622598	0.211	113.9134
25	7A	AX-109380978	0.211	113.9134
25	7A	AX-111508113	0.211	113.9134
25	7A	AX-109467404	0.211	113.9134

25	7A	AX-111009230	0.211	113.9134
25	7A	AX-108894526	0.211	113.9134
25	7A	AX-109869190	0.211	113.9134
25	7A	AX-110515851	0.211	113.9134
25	7A	AX-110931134	0.211	113.9134
25	7A	AX-111673795	0.211	113.9134
25	7A	AX-111516728	0.211	113.9134
25	7A	AX-108957734	0.211	113.9134
25	7A	AX-110973255	0.211	113.9134
25	7A	AX-111451257	0.211	113.9134
25	7A	AX-110041671	0.211	113.9134
25	7A	AX-110915804	0.211	113.9134
25	7A	AX-109432163	0.211	113.9134
25	7A	AX-109836817	0.211	113.9134
25	7A	AX-109392866	0.211	113.9134
25	7A	AX-111756393	0.211	113.9134
25	7A	AX-111453830	0.211	113.9134
25	7A	AX-110408068	0.211	113.9134
25	7A	AX-110382669	0.211	113.9134
25	7A	AX-109647888	0.211	113.9134
25	7A	AX-110437326	0.211	113.9134
25	7A	AX-108856896	0.211	113.9134
25	7A	AX-111128429	0.211	113.9134
25	7A	AX-110125915	0.211	113.9134
25	7A	AX-109914194	0.211	113.9134
25	7A	AX-111469158	0.211	113.9134
25	7A	AX-111502616	0.211	113.9134
25	7A	AX-109856219	0.211	113.9134
25	7A	AX-111524644	0.211	113.9134
25	7A	AX-111051677	0.211	113.9134
25	7A	AX-111049502	0.211	113.9134
25	7A	AX-111521096	0.211	113.9134
25	7A	AX-108758826	0.211	113.9134
25	7A	AX-110122893	0.211	113.9134
25	7A	AX-111582991	0.211	113.9134
25	7A	AX-111166292	0.211	113.9134
25	7A	AX-109422440	0.211	113.9134
25	7A	AX-110452477	0.211	113.9134
25	7A	AX-111449918	0.211	113.9134
25	7A	AX-108810662	0.211	113.9134
25	7A	AX-108846609	0.211	113.9134
25	7A	AX-109823975	0.211	113.9134
25	7A	AX-109399213	0.211	113.9134
25	7A	AX-111103296	0.211	113.9134

25	7A	AX-110470913	0.211	113.9134
25	7A	AX-109304091	0.211	113.9134
25	7A	AX-109427597	0.211	113.9134
25	7A	AX-110505746	0.211	113.9134
25	7A	AX-110506911	0.211	113.9134
25	7A	AX-110379470	0.211	113.9134
25	7A	AX-111155649	0.211	113.9134
25	7A	AX-110984827	0.211	113.9134
25	7A	AX-110473672	0.211	113.9134
25	7A	AX-110484254	0.211	113.9134
25	7A	AX-108882336	0.211	113.9134
25	7A	AX-110986614	0.211	113.9134
25	7A	AX-109272754	0.211	113.9134
25	7A	AX-109619743	0.211	113.9134
25	7A	AX-111459257	0.211	113.9134
25	7A	AX-109082063	0.211	113.9134
25	7A	AX-108867986	0.422	114.3354
25	7A	AX-111108939	0.422	114.3354
25	7A	AX-109509236	0.422	114.3354
25	7A	AX-110949208	0.422	114.3354
25	7A	AX-111637648	0.422	114.3354
25	7A	AX-109402090	0.422	114.3354
25	7A	AX-110073867	0.422	114.3354
25	7A	AX-110506206	0.422	114.3354
25	7A	AX-110538222	0.422	114.3354
25	7A	AX-111454416	0.422	114.3354
25	7A	AX-109431985	0.422	114.3354
25	7A	AX-111624271	0.422	114.3354
25	7A	AX-111158056	0.422	114.3354
25	7A	AX-108938454	0.422	114.3354
25	7A	AX-109941063	0.422	114.3354
25	7A	AX-108750483	0.422	114.3354
25	7A	AX-110531849	0.422	114.3354
25	7A	AX-111618073	0.422	114.3354
25	7A	AX-111526301	0.422	114.3354
25	7A	AX-110993490	0.422	114.3354
25	7A	AX-108733862	0.422	114.3354
25	7A	AX-109825345	0.422	114.3354
25	7A	AX-110988406	0.422	114.3354
25	7A	AX-110027799	0.422	114.3354
25	7A	AX-110164642	0.422	114.3354
25	7A	AX-109866377	0.422	114.3354
25	7A	AX-111020700	0.422	114.3354
25	7A	AX-109901863	0.422	114.3354

25	7A	AX-111080473	0.422	114.3354
25	7A	AX-110565937	0.422	114.3354
25	7A	AX-111230859	0.422	114.3354
25	7A	AX-108763007	0.4255	114.7609
25	7A	AX-110420947	0.4255	114.7609
25	7A	AX-109878181	0.4255	114.7609
25	7A	AX-108748724	0.4255	114.7609
25	7A	AX-110534783	0.4255	114.7609
25	7A	AX-109449750	0.4255	114.7609
25	7A	AX-109945192	0.4255	114.7609
25	7A	AX-111572122	0.4255	114.7609
25	7A	AX-109508288	0.4255	114.7609
25	7A	AX-109881638	0.4255	114.7609
25	7A	AX-109996877	0.4255	114.7609
25	7A	AX-111463776	0.4255	114.7609
25	7A	AX-110714027	0.4255	114.7609
25	7A	AX-109460155	0.4255	114.7609
25	7A	AX-110454282	0.4255	114.7609
25	7A	AX-109816639	0.4255	114.7609
25	7A	AX-108776429	0.4255	114.7609
25	7A	AX-108870513	0.4255	114.7609
25	7A	AX-108956032	0.4255	114.7609
25	7A	AX-108737488	0.4255	114.7609
25	7A	AX-108856870	0.4255	114.7609
25	7A	AX-109474836	0.4255	114.7609
25	7A	AX-111191169	0.4255	114.7609
25	7A	AX-109465295	0.4255	114.7609
25	7A	AX-111617417	0.4255	114.7609
25	7A	AX-110917694	0.4255	114.7609
25	7A	AX-109297084	0.2155	114.9764
25	7A	AX-110953654	0.2155	114.9764
25	7A	AX-110688444	0.2155	114.9764
25	7A	AX-109932583	0.2155	114.9764
25	7A	AX-94482773	0.2155	114.9764
25	7A	AX-109473691	0.2119	115.1883
25	7A	AX-110581292	0.2119	115.1883
25	7A	AX-111104949	0.2119	115.1883
25	7A	AX-109473893	0.4255	115.6138
25	7A	AX-109948609	0.2128	115.8266
25	7A	AX-110653046	0.2128	115.8266
25	7A	AX-109366536	0.2128	115.8266
25	7A	AX-108859039	0.2128	115.8266
25	7A	AX-111123530	0.2128	115.8266
25	7A	AX-109822004	0.2128	115.8266

25	7A	AX-110019545	0.2128	115.8266
25	7A	AX-109877129	0.2128	115.8266
25	7A	AX-111688235	0.2128	115.8266
25	7A	AX-108875867	0.2128	115.8266
25	7A	AX-109985312	0.2128	115.8266
25	7A	AX-109314553	0.2128	115.8266
25	7A	AX-109654998	0.2128	115.8266
25	7A	AX-111135407	0.2128	115.8266
25	7A	AX-111637159	0.2128	115.8266
25	7A	AX-110954396	0.2128	115.8266
25	7A	AX-111539265	0.2128	115.8266
25	7A	AX-111038846	0.2128	115.8266
25	7A	AX-108736971	0.2128	115.8266
25	7A	AX-109598170	0.2128	115.8266
25	7A	AX-108763286	0.2128	115.8266
25	7A	AX-111027195	0.2128	115.8266
25	7A	AX-111133844	0.2128	115.8266
25	7A	AX-108965103	0.2128	115.8266
25	7A	AX-108734018	0.2128	115.8266
25	7A	AX-110913554	0.2128	115.8266
25	7A	AX-109898505	0.2128	115.8266
25	7A	AX-111048148	0.2128	115.8266
25	7A	AX-111043049	0.2128	115.8266
25	7A	AX-109556617	0.2128	115.8266
25	7A	AX-108794808	0.2128	115.8266
25	7A	AX-109383675	0.2128	115.8266
25	7A	AX-109584930	0.2128	115.8266
25	7A	AX-110913122	0.2128	115.8266
25	7A	AX-111047035	0.2128	115.8266
25	7A	AX-110606974	0.2128	115.8266
25	7A	AX-108881635	0.2128	115.8266
25	7A	AX-109873735	0.2128	115.8266
25	7A	AX-110450237	0.2128	115.8266
25	7A	AX-111468733	0.2128	115.8266
25	7A	AX-110140715	0.2128	115.8266
25	7A	AX-110992096	0.2119	116.0385
25	7A	AX-111500963	0.2119	116.0385
25	7A	AX-111493624	0.2119	116.0385
25	7A	AX-109953034	0.2119	116.0385
25	7A	AX-108752076	0.4274	116.4658
25	7A	AX-110516759	1.7865	118.2523
25	7A	AX-111640994	1.7865	118.2523
25	7A	AX-111494115	0.6494	118.9017
25	7A	AX-111718430	0.6494	118.9017

25	7A	AX-109832507	0.6494	118.9017
25	7A	AX-111031978	2.1944	121.0961
25	7A	AX-109344087	2.1944	123.2905
25	7A	AX-108776518	0.4202	123.7106
25	7A	AX-108776052	0.4202	123.7106
25	7A	AX-111580121	0.4202	123.7106
25	7A	AX-109961515	0.4202	123.7106
25	7A	AX-109580938	0.4202	123.7106
25	7A	AX-110926343	0.4202	123.7106
25	7A	AX-111235463	0.4202	123.7106
25	7A	AX-110427622	0.4202	123.7106
25	7A	AX-111165139	0.4202	123.7106
25	7A	AX-111037016	0.4202	123.7106
25	7A	AX-109626877	0.4202	123.7106
25	7A	AX-110427241	0.4202	123.7106
25	7A	AX-111601377	0.4202	123.7106
25	7A	AX-109980723	0.4202	123.7106
25	7A	AX-109379418	0.4202	123.7106
25	7A	AX-110365679	0.4202	123.7106
25	7A	AX-110690944	0.4202	123.7106
25	7A	AX-110537501	0.4202	123.7106
25	7A	AX-108807618	0.4202	123.7106
25	7A	AX-108917971	0.4202	123.7106
25	7A	AX-111598459	0.4202	123.7106
25	7A	AX-111505833	0.4202	123.7106
25	7A	AX-110574731	0.4202	123.7106
25	7A	AX-110024998	0.4202	123.7106
25	7A	AX-109272720	0.4202	123.7106
25	7A	AX-110941854	0.4202	123.7106
25	7A	AX-109360436	0.4202	123.7106
25	7A	AX-110623050	0.4202	123.7106
25	7A	AX-110408635	0.4202	123.7106
25	7A	AX-111613934	0.4202	123.7106
25	7A	AX-108868591	0.4202	123.7106
25	7A	AX-109847703	0.4202	123.7106
25	7A	AX-111661047	0.4202	123.7106
25	7A	AX-111028422	0.4202	123.7106
25	7A	AX-111113643	0.4202	123.7106
25	7A	AX-108955101	0.4202	123.7106
25	7A	AX-110165986	0.4202	123.7106
25	7A	AX-109379134	0.4202	123.7106
25	7A	AX-109924604	0.4202	123.7106
25	7A	AX-110422422	0.4202	123.7106
25	7A	AX-110009414	0.4202	123.7106

25	7A	AX-110947493	0.4202	123.7106
25	7A	AX-109523749	0.4202	123.7106
25	7A	AX-109603593	0.4202	123.7106
25	7A	AX-110402599	0.4202	123.7106
25	7A	AX-111574582	0.4202	123.7106
25	7A	AX-111628095	0.4202	123.7106
25	7A	AX-111062075	0.4202	123.7106
25	7A	AX-109423826	0.4202	123.7106
25	7A	AX-110963650	0.4202	123.7106
25	7A	AX-111585247	0.4202	123.7106
25	7A	AX-109833374	0.4202	123.7106
25	7A	AX-110515663	0.4202	123.7106
25	7A	AX-110519549	0.4202	123.7106
25	7A	AX-111129875	0.4202	123.7106
25	7A	AX-111473789	0.4202	123.7106
25	7A	AX-111535638	0.4202	123.7106
25	7A	AX-94428857	0.4202	123.7106
25	7A	AX-108774391	0.4202	124.1308
25	7A	AX-111499051	0.6329	124.7638
25	7A	AX-111033133	0.6329	124.7638
25	7A	AX-109930449	0.6329	124.7638
25	7A	AX-109992963	0.6329	124.7638
25	7A	AX-111605774	0.6329	124.7638
25	7A	AX-109878395	0.6329	124.7638
25	7A	AX-108740774	0.6329	124.7638
25	7A	AX-110546809	0.6329	124.7638
25	7A	AX-110627601	0.6329	124.7638
25	7A	AX-111529512	0.6329	124.7638
25	7A	AX-108870838	0.6329	124.7638
25	7A	AX-110937220	0.6329	124.7638
25	7A	AX-110510020	0.6329	124.7638
25	7A	AX-109588412	0.6329	124.7638
25	7A	AX-110981750	0.6329	124.7638
25	7A	AX-110962280	0.6329	124.7638
25	7A	AX-110078831	0.6329	124.7638
25	7A	AX-111028390	0.6329	124.7638
25	7A	AX-110933157	0.6329	124.7638
25	7A	AX-111011830	0.6329	124.7638
25	7A	AX-109921979	0.6329	124.7638
25	7A	AX-108942746	0.6329	124.7638
25	7A	AX-111491830	0.6329	124.7638
25	7A	AX-109472974	0.6329	124.7638
25	7A	AX-111487691	0.6329	124.7638
25	7A	AX-109465127	0.6329	124.7638

25	7A	AX-109348978	0.6329	124.7638
25	7A	AX-110906391	0.6329	124.7638
25	7A	AX-110964808	0.6329	124.7638
25	7A	AX-110922774	0.6329	124.7638
25	7A	AX-110674245	0.6329	124.7638
25	7A	AX-108749139	0.6329	124.7638
25	7A	AX-108842149	0.6329	124.7638
25	7A	AX-108840015	0.6329	124.7638
25	7A	AX-108918469	0.6329	124.7638
25	7A	AX-109313220	0.6329	124.7638
25	7A	AX-108856877	0.6329	124.7638
25	7A	AX-111110151	0.6329	124.7638
25	7A	AX-110586106	0.6329	124.7638
25	7A	AX-110438380	0.6329	124.7638
25	7A	AX-108730154	0.6329	124.7638
25	7A	AX-111117481	0.6329	124.7638
25	7A	AX-109823569	0.6329	124.7638
25	7A	AX-109887328	0.6329	124.7638
25	7A	AX-109454973	0.6329	124.7638
25	7A	AX-109996548	0.6329	124.7638
25	7A	AX-110474696	0.6329	124.7638
25	7A	AX-109399734	0.6329	124.7638
25	7A	AX-109826496	0.6329	124.7638
25	7A	AX-110039820	0.6329	124.7638
25	7A	AX-109892074	0.6329	124.7638
25	7A	AX-110517626	0.6329	124.7638
25	7A	AX-110438075	0.6329	124.7638
25	7A	AX-110134684	0.6329	124.7638
25	7A	AX-108795673	0.6329	124.7638
25	7A	AX-111134025	0.6329	124.7638
25	7A	AX-110373955	0.6329	124.7638
25	7A	AX-110740059	0.6329	124.7638
25	7A	AX-109286613	0.6329	124.7638
25	7A	AX-110018694	0.6329	124.7638
25	7A	AX-111143956	0.6329	124.7638
25	7A	AX-109892953	0.6329	124.7638
25	7A	AX-110384821	0.6329	124.7638
25	7A	AX-110000740	0.6329	124.7638
25	7A	AX-110370079	0.6329	124.7638
25	7A	AX-111656385	0.6329	124.7638
25	7A	AX-111009900	0.6329	124.7638
25	7A	AX-110455795	0.6329	124.7638
25	7A	AX-111158373	0.6329	124.7638
25	7A	AX-110169498	0.6329	124.7638

25	7A	AX-109826698	0.6329	124.7638
25	7A	AX-111153755	0.6329	124.7638
25	7A	AX-110176351	0.6329	124.7638
25	7A	AX-109086025	0.6329	124.7638
25	7A	AX-108823705	0.6329	124.7638
25	7A	AX-111238272	0.6329	124.7638
25	7A	AX-111549301	0.6329	124.7638
25	7A	AX-110006765	0.6329	124.7638
25	7A	AX-111627395	0.6329	124.7638
25	7A	AX-109892431	0.6329	124.7638
25	7A	AX-110404014	0.6329	124.7638
25	7A	AX-108809088	0.6329	124.7638
25	7A	AX-109294920	0.2092	124.973
25	7A	AX-109944167	0.2092	124.973
25	7A	AX-109002083	0.2092	124.973
25	7A	AX-110362825	0.2092	125.1822
25	7A	AX-111625381	0.2092	125.1822
25	7A	AX-110540105	0.2092	125.1822
25	7A	AX-110926085	0.2092	125.3914
25	7A	AX-108934056	0.2092	125.3914
25	7A	AX-109405759	0.2092	125.3914
25	7A	AX-108859208	0.2092	125.3914
25	7A	AX-110401587	0.2092	125.3914
25	7A	AX-110954880	0.2092	125.3914
25	7A	AX-108805298	0.2092	125.3914
25	7A	AX-110445761	0.2092	125.3914
25	7A	AX-110979868	0.2092	125.3914
25	7A	AX-109405390	0.2092	125.3914
25	7A	AX-108774775	0.2092	125.3914
25	7A	AX-110645722	0.2092	125.3914
25	7A	AX-109968785	0.4184	125.8098
25	7A	AX-110064487	0.2092	126.019
25	7A	AX-110677006	0.8475	126.8665
25	7A	AX-110437365	0.8475	126.8665
25	7A	AX-111094864	0.8475	126.8665
25	7A	AX-109357532	0.2101	127.0766
25	7A	AX-108975568	0.2101	127.0766
25	7A	AX-111124288	0.2101	127.0766
25	7A	AX-110466533	0.211	127.2876
25	7A	AX-110389115	0.2101	127.4977
25	7A	AX-109081524	0.4202	127.9179
25	7A	AX-109862649	0.211	128.1288
25	7A	AX-111665233	0.211	128.3398
25	7A	AX-110376315	0.2101	128.5499

25	7A	AX-109824443	0.2101	128.5499
25	7A	AX-110419425	0.2101	128.5499
25	7A	AX-110060270	0.2101	128.5499
25	7A	AX-110551335	3.2007	131.7506
25	7A	AX-108899437	0.2146	131.9652
25	7A	AX-111083840	0.2146	131.9652
25	7A	AX-110972792	0.2146	131.9652
25	7A	AX-109973140	0.2146	131.9652
25	7A	AX-108882531	0.2146	131.9652
25	7A	AX-109844628	0.2146	131.9652
25	7A	AX-110981560	0.2146	131.9652
25	7A	AX-109510771	0.2146	131.9652
25	7A	AX-109110307	0.2146	131.9652
25	7A	AX-110999895	0.2146	131.9652
25	7A	AX-109509511	0.2146	131.9652
25	7A	AX-110977297	0.2146	131.9652
25	7A	AX-110483438	0.6438	132.609
25	7A	AX-110632223	0.8697	133.4787
25	7A	AX-108872106	1.0919	134.5705
25	7A	AX-109838679	0.8622	135.4327
25	7A	AX-110670940	0.8622	135.4327
25	7A	AX-110458473	0.8622	135.4327
25	7A	AX-110010122	0.8622	135.4327
25	7A	AX-109432267	0.2101	135.6428
25	7A	AX-109394965	0.2128	135.8555
25	7A	AX-110987675	0.2137	136.0692
25	7A	AX-108799303	0.2137	136.0692
25	7A	AX-108762348	0.6411	136.7103
25	7A	AX-111505383	0.4237	137.134
25	7A	AX-108839010	0.2128	137.3468
25	7A	AX-111535682	1.9834	139.3302
25	7A	AX-110584960	1.9834	139.3302
25	7A	AX-111149309	1.9834	139.3302
25	7A	AX-111157953	1.9834	139.3302
25	7A	AX-110479736	1.9834	139.3302
25	7A	AX-108835103	1.9834	139.3302
25	7A	AX-110607218	1.9834	139.3302
25	7A	AX-111474675	1.9834	139.3302
25	7A	AX-109421919	1.9834	139.3302
25	7A	AX-111034263	1.9834	139.3302
25	7A	AX-109537800	1.9834	139.3302
25	7A	AX-111045618	0.4202	139.7504
25	7A	AX-89605786	1.9491	141.6994
25	7A	AX-111511322	1.064	142.7634

25	7A	AX-108912349	1.7028	144.4662
25	7A	AX-109874881	0.4167	144.8829
25	7A	AX-111585581	2.854	147.7368
25	7A	AX-110978431	2.4355	150.1724
25	7A	AX-110483331	0.2128	150.3852
25	7A	AX-110430243	0.4237	150.8089
25	7A	AX-110021101	0.6383	151.4472
25	7A	AX-110449729	0.4237	151.871
25	7A	AX-111589705	0.4237	151.871
25	7A	AX-111659846	1.0731	152.9441
25	7A	AX-110518554	2.2041	155.1482
25	7A	AX-108735843	2.2041	155.1482
25	7A	AX-111706863	2.2041	155.1482
25	7A	AX-108837168	2.2041	155.1482
25	7A	AX-110360613	2.2041	155.1482
25	7A	AX-111628176	2.2041	155.1482
25	7A	AX-111604641	2.2041	155.1482
25	7A	AX-110973098	2.2041	155.1482
25	7A	AX-109585023	2.2041	155.1482
25	7A	AX-111137584	2.2041	155.1482
25	7A	AX-111452399	0.4292	155.5774
25	7A	AX-109921024	1.3219	156.8993
25	7A	AX-110711015	1.3219	156.8993
25	7A	AX-111625723	1.3219	156.8993
25	7A	AX-110094527	1.3219	156.8993
25	7A	AX-111659152	0.4237	157.323
25	7A	AX-108754004	6.2525	163.5755
25	7A	AX-109423906	0.6411	164.2166
25	7A	AX-109532920	0.6411	164.2166
25	7A	AX-110532208	0.4274	164.6439
25	7A	AX-109892640	0.4274	165.0713
25	7A	AX-110929011	4.0814	169.1527
25	7A	AX-111651569	2.4355	171.5883
25	7A	AX-110566631	1.5091	173.0973
25	7A	AX-108857319	1.5091	173.0973
25	7A	AX-109423390	1.5091	173.0973
25	7A	AX-111651884	1.5091	173.0973
25	7A	AX-111027755	2.5997	175.6971
25	7A	AX-109473518	2.5997	175.6971
25	7A	AX-109622474	0.2058	175.9028
25	7A	AX-110647923	0.2058	175.9028
25	7A	AX-110042725	3.5457	179.4486
25	7A	AX-111493620	2.1944	181.643
25	7A	AX-108958961	3.6595	185.3025

25	7A	AX-108748282	1.299	186.6015
25	7A	AX-111127771	1.0731	187.6746
25	7A	AX-109320934	0.211	187.8856
25	7A	AX-110439148	0.4255	188.3111
25	7A	AX-110516258	0.2092	188.5203
25	7A	AX-111455594	1.5156	190.0359
25	7A	AX-109518769	0.6411	190.677
25	7A	AX-108761661	0.2164	190.8934
25	7A	AX-109934742	0.2164	190.8934
25	7A	AX-110103164	0.2164	191.1099
25	7A	AX-109316663	0.2164	191.1099
25	7A	AX-109838868	4.6866	195.7965
25	7A	AX-111659056	1.7785	197.575
25	7A	AX-108947190	3.1573	200.7323
25	7A	AX-111032043	3.1573	200.7323
25	7A	AX-111550952	0.8659	201.5982
25	7A	AX-109984834	0.8659	201.5982
25	7A	AX-111631796	0.8659	201.5982
25	7A	AX-109987097	0.8659	201.5982
25	7A	AX-111498596	0.8659	201.5982
25	7A	AX-110499050	4.1559	205.7541
25	7A	AX-109300922	0.4608	206.215
25	7A	AX-111019691	0.2164	206.4314
25	7A	AX-108795572	2.958	209.3894
25	7A	AX-108775555	16.7979	226.1873
26	7B	AX-110629552	0	0
26	7B	AX-110954191	0	0
26	7B	AX-109011340	0	0
26	7B	AX-110997141	0.2101	0.2101
26	7B	AX-110533677	0.2101	0.2101
26	7B	AX-111213961	0.2101	0.2101
26	7B	AX-109848983	0.2101	0.2101
26	7B	AX-111502686	7.2978	7.5078
26	7B	AX-108932115	7.2978	7.5078
26	7B	AX-110438013	7.2978	7.5078
26	7B	AX-109398733	7.2978	7.5078
26	7B	AX-108889748	7.2978	7.5078
26	7B	AX-109880655	7.2978	7.5078
26	7B	AX-109968663	0.211	7.7188
26	7B	AX-109975845	0.2101	7.9289
26	7B	AX-109270081	0.2101	7.9289
26	7B	AX-111450645	1.0685	8.9974
26	7B	AX-89507637	10.9988	19.9962
26	7B	AX-111535289	2.1659	22.1621

26	7B	AX-109285209	7.5079	29.6699
26	7B	AX-110443220	0.2066	29.8765
26	7B	AX-109278740	0.2066	29.8765
26	7B	AX-111638601	0.6276	30.5042
26	7B	AX-111054336	0.4202	30.9244
26	7B	AX-110087763	1.7174	32.6418
26	7B	AX-111512545	3.074	35.7158
26	7B	AX-109864353	1.5156	37.2314
26	7B	AX-109964834	0.8512	38.0826
26	7B	AX-109275370	0.2066	38.2892
26	7B	AX-109372990	2.6456	40.9348
26	7B	AX-109983408	2.2336	43.1685
26	7B	AX-111569504	0.4274	43.5958
26	7B	AX-111016362	0.6383	44.2341
26	7B	AX-109431444	0.6383	44.2341
26	7B	AX-109478552	0.211	44.4451
26	7B	AX-109271886	0.211	44.4451
26	7B	AX-110460118	4.8315	49.2766
26	7B	AX-111141784	4.8315	49.2766
26	7B	AX-110921008	4.8315	49.2766
26	7B	AX-110585187	4.8315	49.2766
26	7B	AX-111603759	4.8315	49.2766
26	7B	AX-109881255	1.2823	50.559
26	7B	AX-111195772	1.2823	50.559
26	7B	AX-111458552	0.2092	50.7682
26	7B	AX-109966064	0.2092	50.7682
26	7B	AX-108932514	0.2092	50.7682
26	7B	AX-109925774	0.2092	50.7682
26	7B	AX-109513409	0.2092	50.7682
26	7B	AX-110028265	0.2092	50.7682
26	7B	AX-109940858	3.3532	54.1214
26	7B	AX-110086680	3.3532	54.1214
26	7B	AX-111139406	3.3532	54.1214
26	7B	AX-110031797	3.3532	54.1214
26	7B	AX-111003520	8.0003	62.1217
26	7B	AX-109943789	0.2058	62.3274
26	7B	AX-110516537	6.3124	68.6398
26	7B	AX-94498693	0.4167	69.0565
26	7B	AX-111153283	0.4167	69.0565
26	7B	AX-108941321	0.4167	69.0565
26	7B	AX-94421774	0.4167	69.0565
26	7B	AX-109477613	0.2058	69.2623
26	7B	AX-109966136	0.2058	69.2623
26	7B	AX-108952314	0.2058	69.2623

26	7B	AX-111588859	0.2058	69.468
26	7B	AX-108804333	0.2058	69.468
26	7B	AX-109937668	0.2058	69.468
26	7B	AX-108847234	0.4115	69.8796
26	7B	AX-110922928	0.4115	69.8796
26	7B	AX-109827619	0.4115	69.8796
26	7B	AX-111127794	0.4115	69.8796
26	7B	AX-111559157	0.4115	69.8796
26	7B	AX-111460341	0.4115	69.8796
26	7B	AX-110027495	0.4115	69.8796
26	7B	AX-109431681	0.4115	69.8796
26	7B	AX-109435147	0.4115	69.8796
26	7B	AX-108898855	0.4115	69.8796
26	7B	AX-111087950	0.4115	69.8796
26	7B	AX-109422009	0.4115	69.8796
26	7B	AX-110045544	0.4115	69.8796
26	7B	AX-110128330	0.4115	70.2911
26	7B	AX-111590325	0.4115	70.2911
26	7B	AX-109278676	0.4115	70.2911
26	7B	AX-111108626	0.4115	70.2911
26	7B	AX-111118930	0.4115	70.2911
26	7B	AX-110416968	0.4115	70.2911
26	7B	AX-110552616	0.4115	70.2911
26	7B	AX-109894553	0.4115	70.2911
26	7B	AX-94525735	0.4115	70.2911
26	7B	AX-109324855	0.2049	70.496
26	7B	AX-111723126	0.2049	70.496
26	7B	AX-110912865	0.2049	70.496
26	7B	AX-111009899	0.2049	70.496
26	7B	AX-109912165	0.4132	70.9092
26	7B	AX-110574606	0.4132	70.9092
26	7B	AX-110434237	0.4132	70.9092
26	7B	AX-111671128	0.4132	70.9092
26	7B	AX-111087819	0.2058	71.115
26	7B	AX-108965136	0.2066	71.3216
26	7B	AX-110511335	0.2066	71.3216
26	7B	AX-110372247	0.2075	71.5291
26	7B	AX-111601655	0.2058	71.7349
26	7B	AX-109598400	0.2058	71.7349
26	7B	AX-111671754	0.2058	71.7349
26	7B	AX-111024189	0.2058	71.7349
26	7B	AX-110957024	0.2049	71.9398
26	7B	AX-111701721	0.2049	71.9398
26	7B	AX-110419574	0.2049	71.9398

26	7B	AX-109577756	0.2049	71.9398
26	7B	AX-109396027	0.2049	71.9398
26	7B	AX-111283974	0.2049	71.9398
26	7B	AX-109351881	0.2049	71.9398
26	7B	AX-110920095	0.2049	71.9398
26	7B	AX-109324588	0.2049	71.9398
26	7B	AX-109862866	0.2049	71.9398
26	7B	AX-110573191	0.2049	71.9398
26	7B	AX-109363340	0.2049	71.9398
26	7B	AX-110611183	0.2049	71.9398
26	7B	AX-109388787	0.2049	72.1447
26	7B	AX-86167574	0.2049	72.1447
26	7B	AX-111525369	0.2049	72.1447
26	7B	AX-109450144	0.2049	72.1447
26	7B	AX-109428201	0.2049	72.1447
26	7B	AX-110130928	0.2049	72.3496
26	7B	AX-109451095	0.2075	72.5571
26	7B	AX-110023523	0.2083	72.7654
26	7B	AX-110956551	0.2083	72.7654
26	7B	AX-111474548	0.2083	72.7654
26	7B	AX-110134257	0.2083	72.7654
26	7B	AX-108766027	0.2083	72.7654
26	7B	AX-109930103	0.2083	72.7654
26	7B	AX-111574279	0.2083	72.7654
26	7B	AX-94382843	0.2058	72.9712
26	7B	AX-109009265	0.2058	72.9712
26	7B	AX-109856268	0.2058	72.9712
26	7B	AX-110420891	0.2058	72.9712
26	7B	AX-111666202	0.2058	72.9712
26	7B	AX-95180590	0.2058	72.9712
26	7B	AX-111827631	0.2058	72.9712
26	7B	AX-109330015	0.2058	72.9712
26	7B	AX-110533145	0.2058	72.9712
26	7B	AX-94442813	0.2058	72.9712
26	7B	AX-108912067	0.2058	72.9712
26	7B	AX-110418386	0.2058	72.9712
26	7B	AX-111634483	1.2608	74.232
26	7B	AX-108812129	1.2608	74.232
26	7B	AX-111056402	1.2608	74.232
26	7B	AX-110602843	1.2608	74.232
26	7B	AX-86173595	1.2608	74.232
26	7B	AX-109402029	1.2608	74.232
26	7B	AX-109927712	1.2608	74.232
26	7B	AX-110990976	1.2608	74.232

26	7B	AX-109890394	0.2049	74.4369
26	7B	AX-111690206	0.2049	74.4369
26	7B	AX-109959821	0.2049	74.4369
26	7B	AX-108983521	0.625	75.0619
26	7B	AX-94448136	0.625	75.0619
26	7B	AX-94390641	0.625	75.0619
26	7B	AX-110498108	0.2066	75.2685
26	7B	AX-111780551	0.2066	75.2685
26	7B	AX-108755073	0.2066	75.2685
26	7B	AX-111060003	0.2066	75.2685
26	7B	AX-111085159	0.4184	75.6869
26	7B	AX-110507021	1.5091	77.196
26	7B	AX-111571387	0.6383	77.8344
26	7B	AX-111232142	0.8659	78.7002
26	7B	AX-109279348	0.8659	78.7002
26	7B	AX-111700650	0.8659	78.7002
26	7B	AX-111009383	0.8659	78.7002
26	7B	AX-110557086	0.8659	78.7002
26	7B	AX-110427192	0.8659	78.7002
26	7B	AX-111287487	0.8659	78.7002
26	7B	AX-109380410	0.8659	78.7002
26	7B	AX-110368632	0.8659	78.7002
26	7B	AX-110578637	0.8659	78.7002
26	7B	AX-110506499	0.8659	78.7002
26	7B	AX-109582157	0.8659	78.7002
26	7B	AX-111517517	7.9481	86.6483
26	7B	AX-109325605	3.1431	89.7914
26	7B	AX-109450735	0.6329	90.4244
26	7B	AX-95152493	2.854	93.2784
26	7B	AX-108765522	2.854	93.2784
26	7B	AX-109347474	2.854	93.2784
26	7B	AX-109343619	2.854	93.2784
26	7B	AX-109295550	2.854	93.2784
26	7B	AX-109000697	2.854	93.2784
26	7B	AX-111092574	2.854	93.2784
26	7B	AX-111644539	2.854	93.2784
26	7B	AX-108780035	2.854	93.2784
26	7B	AX-95253477	0.8404	94.1188
26	7B	AX-109894589	0.8404	94.1188
26	7B	AX-94657532	0.8404	94.1188
26	7B	AX-110053591	1.2715	95.3902
26	7B	AX-111604672	1.2715	95.3902
26	7B	AX-109395510	1.2715	95.3902
26	7B	AX-108794357	1.2715	95.3902

26	7B	AX-109004774	0.8369	96.2271
26	7B	AX-109849135	3.0741	99.3012
26	7B	AX-111219595	0.4167	99.7178
26	7B	AX-109054263	0.4167	99.7178
26	7B	AX-109582661	0.4167	99.7178
26	7B	AX-109964198	0.4167	99.7178
26	7B	AX-108927437	0.4167	99.7178
26	7B	AX-111058347	0.4167	99.7178
26	7B	AX-108743917	0.2075	99.9253
26	7B	AX-108878887	0.2075	99.9253
26	7B	AX-111485326	0.2075	99.9253
26	7B	AX-108838778	0.2075	99.9253
26	7B	AX-111126608	0.2075	99.9253
26	7B	AX-110915122	0.2075	99.9253
26	7B	AX-110437442	0.2075	99.9253
26	7B	AX-110974083	0.2075	99.9253
26	7B	AX-89352773	0.2075	99.9253
26	7B	AX-110535820	0.2075	99.9253
26	7B	AX-94393306	0.2075	99.9253
26	7B	AX-111579906	0.2075	100.1328
26	7B	AX-110437542	0.2075	100.1328
26	7B	AX-109655855	0.2075	100.1328
26	7B	AX-111650464	0.2075	100.1328
26	7B	AX-111760043	0.2075	100.1328
26	7B	AX-108911784	0.2075	100.1328
26	7B	AX-111144765	0.2075	100.1328
26	7B	AX-110918846	0.2075	100.1328
26	7B	AX-111649134	0.2075	100.1328
26	7B	AX-110560654	0.2075	100.1328
26	7B	AX-109004191	0.2075	100.1328
26	7B	AX-108967887	0.2075	100.1328
26	7B	AX-110437582	0.2075	100.1328
26	7B	AX-108969519	0.4274	100.5602
26	7B	AX-108731004	0.2128	100.7729
26	7B	AX-109513110	0.2128	100.7729
26	7B	AX-109295183	0.2128	100.7729
26	7B	AX-110006349	0.2128	100.7729
26	7B	AX-109891027	0.2128	100.7729
26	7B	AX-95685082	0.844	101.6169
26	7B	AX-110059100	0.2137	101.8306
26	7B	AX-110360149	1.0824	102.913
26	7B	AX-109864927	1.0824	102.913
26	7B	AX-109389033	1.0824	102.913
26	7B	AX-94429568	1.0824	102.913

26	7B	AX-111487758	0.2092	103.1222
26	7B	AX-109276038	0.2092	103.1222
26	7B	AX-110592868	0.4202	103.5424
26	7B	AX-109818475	0.4202	103.5424
26	7B	AX-110944845	0.4202	103.5424
26	7B	AX-109495359	0.4202	103.5424
26	7B	AX-111761643	0.4202	103.5424
26	7B	AX-109851212	0.4202	103.5424
26	7B	AX-108861535	0.2101	103.7525
26	7B	AX-108910461	4.1379	107.8903
26	7B	AX-108833738	4.1379	107.8903
26	7B	AX-108901668	4.1379	107.8903
26	7B	AX-109404604	4.1379	107.8903
26	7B	AX-108762309	4.1379	107.8903
26	7B	AX-109294740	4.1379	107.8903
26	7B	AX-111587257	4.1379	107.8903
26	7B	AX-111737926	4.1379	107.8903
26	7B	AX-110485151	4.1379	107.8903
26	7B	AX-109404389	4.1379	107.8903
26	7B	AX-110410095	4.1379	107.8903
26	7B	AX-108960707	4.1379	107.8903
26	7B	AX-109961440	4.1379	107.8903
26	7B	AX-108746294	4.1379	107.8903
26	7B	AX-111538295	4.1379	107.8903
26	7B	AX-109421741	4.1379	107.8903
26	7B	AX-109484758	4.1379	107.8903
26	7B	AX-108896998	4.1379	107.8903
26	7B	AX-110473255	4.1379	107.8903
26	7B	AX-111740326	0.4237	108.3141
26	7B	AX-110958573	0.4237	108.3141
26	7B	AX-108910394	0.4237	108.3141
26	7B	AX-111761158	0.211	108.525
26	7B	AX-109492564	0.211	108.525
26	7B	AX-109914342	0.211	108.525
26	7B	AX-111217626	0.211	108.525
26	7B	AX-110488256	0.211	108.525
26	7B	AX-110931730	0.211	108.525
26	7B	AX-110123228	0.211	108.525
26	7B	AX-109395757	0.211	108.525
26	7B	AX-95685447	0.211	108.525
26	7B	AX-109873945	0.211	108.525
26	7B	AX-110390277	0.211	108.525
26	7B	AX-108916024	0.211	108.525
26	7B	AX-111632435	0.211	108.525

26	7B	AX-108938830	7.5819	116.1069
26	7B	AX-111451435	3.5616	119.6685
26	7B	AX-109300995	3.5616	119.6685
26	7B	AX-111765415	3.5616	119.6685
26	7B	AX-110976577	3.5616	119.6685
26	7B	AX-109354771	6.8828	126.5512
26	7B	AX-108945293	6.8828	126.5512
26	7B	AX-111699424	0.8404	127.3916
26	7B	AX-109975245	0.2075	127.5991
26	7B	AX-109922088	0.2075	127.5991
26	7B	AX-108787760	0.2075	127.8066
26	7B	AX-108758415	0.2075	127.8066
26	7B	AX-110960575	1.2769	129.0835
26	7B	AX-109915794	1.2769	129.0835
26	7B	AX-111597125	1.2769	129.0835
26	7B	AX-108850591	1.2769	129.0835
26	7B	AX-108759562	1.2769	129.0835
26	7B	AX-111036578	1.2769	129.0835
26	7B	AX-109345708	1.2769	129.0835
26	7B	AX-110581642	1.2769	129.0835
26	7B	AX-110382692	1.2769	129.0835
26	7B	AX-109312599	1.2769	129.0835
26	7B	AX-108799694	1.2769	129.0835
26	7B	AX-111538242	1.2769	129.0835
26	7B	AX-110129782	1.2769	129.0835
26	7B	AX-110975092	0.2092	129.2927
26	7B	AX-109921776	0.2092	129.2927
26	7B	AX-109420937	0.2092	129.2927
26	7B	AX-108729461	0.2092	129.2927
26	7B	AX-109818113	0.2092	129.2927
26	7B	AX-108789829	0.2092	129.2927
26	7B	AX-108922343	0.4184	129.7111
26	7B	AX-111488299	0.4184	129.7111
26	7B	AX-109426608	0.4184	129.7111
26	7B	AX-111111497	0.4184	129.7111
26	7B	AX-108865688	0.4184	129.7111
26	7B	AX-111102830	0.8512	130.5622
26	7B	AX-89364680	1.7398	132.3021
26	7B	AX-95658405	1.949	134.2511
26	7B	AX-111489045	1.949	134.2511
26	7B	AX-108955394	1.949	134.2511
26	7B	AX-111476984	1.949	134.2511
26	7B	AX-109939313	1.949	134.2511
26	7B	AX-109436656	1.949	134.2511

26	7B	AX-110520191	1.0595	135.3106
26	7B	AX-110493377	1.0595	135.3106
26	7B	AX-109368435	2.1848	137.4954
26	7B	AX-110422004	1.0685	138.5639
26	7B	AX-110362293	0.4202	138.9841
26	7B	AX-109867082	0.4202	138.9841
26	7B	AX-111703868	0.4202	138.9841
26	7B	AX-109412823	3.6263	142.6104
26	7B	AX-109440423	0.6356	143.246
26	7B	AX-109915998	0.6356	143.246
26	7B	AX-109823501	0.6356	143.246
26	7B	AX-89318179	0.422	143.6679
26	7B	AX-111729689	0.422	143.6679
26	7B	AX-109937689	0.422	143.6679
26	7B	AX-110638819	0.6329	144.3009
26	7B	AX-108806378	0.6329	144.3009
26	7B	AX-110058062	0.6329	144.3009
26	7B	AX-108798060	0.6329	144.3009
26	7B	AX-109362158	0.6329	144.3009
26	7B	AX-109820793	0.6329	144.3009
26	7B	AX-111078027	0.6329	144.3009
26	7B	AX-109395779	0.2101	144.511
26	7B	AX-111510102	0.2101	144.511
26	7B	AX-110476719	0.2101	144.511
26	7B	AX-111624029	0.2101	144.511
26	7B	AX-110671390	0.2101	144.511
26	7B	AX-108966862	0.2101	144.511
26	7B	AX-110933185	0.2092	144.7202
26	7B	AX-108840376	0.2092	144.7202
26	7B	AX-110912077	0.2092	144.7202
26	7B	AX-89609643	0.2092	144.7202
26	7B	AX-110470708	0.2092	144.7202
26	7B	AX-110544069	0.2092	144.7202
26	7B	AX-108877192	5.6048	150.3249
26	7B	AX-108778081	0.2092	150.5341
26	7B	AX-109383997	0.2092	150.5341
26	7B	AX-89658728	0.4238	150.9579
26	7B	AX-111758108	1.5091	152.4669
26	7B	AX-110958947	1.5091	152.4669
26	7B	AX-109861863	1.5091	152.4669
26	7B	AX-108733479	1.5091	152.4669
26	7B	AX-110595065	1.5091	152.4669
26	7B	AX-111042826	1.5091	152.4669
26	7B	AX-109887886	1.5091	152.4669

26	7B	AX-110534826	1.5091	152.4669
26	7B	AX-110506527	1.5091	152.4669
26	7B	AX-109915196	1.5091	152.4669
26	7B	AX-89540697	1.5091	152.4669
26	7B	AX-111538141	1.5091	152.4669
26	7B	AX-89745787	1.5091	152.4669
26	7B	AX-108871397	0.4184	152.8854
26	7B	AX-111521867	0.4184	152.8854
26	7B	AX-110442356	0.4184	152.8854
26	7B	AX-110027999	0.4184	152.8854
26	7B	AX-108957944	0.4184	152.8854
26	7B	AX-109926256	0.4184	152.8854
26	7B	AX-110432281	0.4184	152.8854
26	7B	AX-111591462	2.4248	155.3102
27	7D	AX-109995969	0	0
27	7D	AX-109394203	1.5289	1.5289
27	7D	AX-110367620	1.5289	1.5289
27	7D	AX-109440048	1.5289	1.5289
27	7D	AX-94591962	0.6356	2.1645
27	7D	AX-109396575	3.3383	5.5028
27	7D	AX-94988821	10.9988	16.5016
27	7D	AX-110576497	6.1926	22.6942
27	7D	AX-109308291	32.9113	55.6054
27	7D	AX-111068627	1.0595	56.6649
27	7D	AX-110534723	1.0595	56.6649
27	7D	AX-109925314	1.0595	56.6649
27	7D	AX-108934838	1.0595	56.6649
27	7D	AX-109822071	1.0595	56.6649
27	7D	AX-94532247	1.0595	56.6649
27	7D	AX-109414221	1.0595	56.6649
27	7D	AX-110939373	1.0595	56.6649
27	7D	AX-109973501	1.0595	56.6649
27	7D	AX-111542089	1.0595	56.6649
27	7D	AX-110585364	1.0595	56.6649
27	7D	AX-110930214	1.0595	56.6649
27	7D	AX-110955847	1.0595	56.6649
27	7D	AX-111049952	1.0595	56.6649
27	7D	AX-111574813	1.0595	56.6649
27	7D	AX-111495426	1.0595	56.6649
27	7D	AX-111455841	1.0595	56.6649
27	7D	AX-109357036	1.0595	56.6649
27	7D	AX-111529179	1.0595	56.6649
27	7D	AX-108831728	1.0595	56.6649
27	7D	AX-110011213	1.0595	56.6649

27	7D	AX-111576402	1.0595	56.6649
27	7D	AX-108896781	1.0595	56.6649
27	7D	AX-111547060	1.0595	56.6649
27	7D	AX-108974605	1.0595	56.6649
27	7D	AX-108761858	1.0595	56.6649
27	7D	AX-110531952	1.0595	56.6649
27	7D	AX-110405382	1.0595	56.6649
27	7D	AX-109456411	1.0595	56.6649
27	7D	AX-108965242	1.0595	56.6649
27	7D	AX-111545339	1.0595	56.6649
27	7D	AX-111513470	1.0595	56.6649
27	7D	AX-111668248	1.0595	56.6649
27	7D	AX-111825580	1.0595	56.6649
27	7D	AX-94991347	1.0595	56.6649
27	7D	AX-95017988	1.0595	56.6649
27	7D	AX-111024414	1.0595	56.6649
27	7D	AX-109930056	1.0595	56.6649
27	7D	AX-111028924	1.0595	56.6649
27	7D	AX-86162249	1.0595	56.6649
27	7D	AX-109850181	2.1565	58.8214
27	7D	AX-110454455	2.1565	58.8214
27	7D	AX-108995998	2.1565	58.8214
27	7D	AX-108803034	4.579	63.4004
27	7D	AX-110620835	4.579	63.4004
27	7D	AX-109920134	5.7394	69.1398
27	7D	AX-108745065	2.2336	71.3734
27	7D	AX-110558586	2.2336	71.3734
27	7D	AX-110994873	6.3422	77.7156
27	7D	AX-111474306	6.3422	77.7156
27	7D	AX-109538909	6.3422	77.7156
27	7D	AX-109304530	6.3422	77.7156
28	7D	AX-108858105	0	0
28	7D	AX-110196726	1.7248	1.7248
28	7D	AX-110124196	1.7248	1.7248
28	7D	AX-111266103	1.7248	1.7248
28	7D	AX-109334871	1.7248	1.7248
28	7D	AX-110945813	1.7248	1.7248
28	7D	AX-111481304	1.7248	1.7248
28	7D	AX-111490337	1.7248	1.7248
28	7D	AX-110028290	1.7248	1.7248
28	7D	AX-109864765	1.7248	1.7248
28	7D	AX-111623278	1.7248	1.7248
28	7D	AX-109189975	1.7248	1.7248
28	7D	AX-109475040	3.5775	5.3023

28	7D	AX-111094913	1.0685	6.3709
28	7D	AX-109916435	1.0685	6.3709
28	7D	AX-109420634	1.0685	6.3709
28	7D	AX-110018188	0.6277	6.9985
28	7D	AX-109394623	0.2075	7.206
28	7D	AX-108908338	0.2075	7.206
28	7D	AX-109848092	0.2075	7.206
28	7D	AX-110893366	0.2075	7.206
28	7D	AX-109346920	0.2075	7.206
28	7D	AX-111103049	0.2075	7.206
28	7D	AX-110360926	0.2075	7.206
28	7D	AX-110494409	0.4202	7.6262
28	7D	AX-109857530	0.4202	7.6262
28	7D	AX-110039699	0.4202	7.6262
28	7D	AX-111024475	0.4202	7.6262
28	7D	AX-109051270	0.4202	7.6262
28	7D	AX-109361131	0.4202	7.6262
28	7D	AX-109835872	0.4202	7.6262
28	7D	AX-89611863	0.4202	7.6262
28	7D	AX-109870077	0.4202	7.6262
28	7D	AX-111981305	0.4202	7.6262
28	7D	AX-110965928	0.4202	7.6262
28	7D	AX-110536550	0.4202	7.6262
28	7D	AX-108730550	0.4202	7.6262
28	7D	AX-110390054	0.4202	7.6262
28	7D	AX-108746549	0.4202	7.6262
28	7D	AX-111011867	0.4202	7.6262
28	7D	AX-111050143	0.4202	7.6262
28	7D	AX-108804985	0.4202	7.6262
28	7D	AX-89348006	0.4202	7.6262
28	7D	AX-108852664	0.4202	7.6262
28	7D	AX-108815937	0.4202	7.6262
28	7D	AX-110821845	0.4202	7.6262
28	7D	AX-111629375	0.4202	7.6262
28	7D	AX-110634161	0.4202	7.6262
28	7D	AX-111218818	0.2092	7.8354
28	7D	AX-111707392	0.2092	7.8354
28	7D	AX-111582863	0.2092	7.8354
28	7D	AX-111588688	0.2092	7.8354
28	7D	AX-109871879	0.4202	8.2556
28	7D	AX-110566065	0.4202	8.2556
28	7D	AX-111611031	0.4202	8.2556
28	7D	AX-86163287	0.4202	8.2556
28	7D	AX-111482485	0.4202	8.2556

28	7D	AX-109542801	0.2092	8.4648
28	7D	AX-108938077	0.2092	8.4648
28	7D	AX-110558556	0.2092	8.4648
28	7D	AX-111980205	0.4202	8.885
28	7D	AX-109301921	0.4202	8.885
28	7D	AX-109788290	0.4202	8.885
28	7D	AX-109946114	0.4202	8.885
28	7D	AX-108824829	0.4202	8.885
28	7D	AX-109165892	0.4202	8.885
28	7D	AX-110409258	0.4202	8.885
28	7D	AX-110421531	0.4202	8.885
28	7D	AX-108790265	0.4202	8.885
28	7D	AX-109396087	0.4202	8.885
28	7D	AX-110466318	0.4202	8.885
28	7D	AX-110501498	0.4202	8.885
28	7D	AX-110068714	0.4184	9.3034
28	7D	AX-111802529	0.2092	9.5126
28	7D	AX-109968184	0.2092	9.5126
28	7D	AX-109861831	0.2092	9.5126
28	7D	AX-110626454	0.2092	9.5126
28	7D	AX-111597983	0.2092	9.5126
28	7D	AX-109898989	0.2092	9.5126
28	7D	AX-111146616	0.4184	9.931
28	7D	AX-109475636	0.4184	9.931
28	7D	AX-110478860	0.4184	9.931
28	7D	AX-110527201	0.4184	9.931
28	7D	AX-111547701	0.4184	9.931
28	7D	AX-111500225	0.4184	9.931
28	7D	AX-109601394	0.4184	9.931
28	7D	AX-108786138	0.4184	9.931
28	7D	AX-110610460	0.4184	9.931
28	7D	AX-109815974	0.4184	9.931
28	7D	AX-89516827	0.4184	9.931
28	7D	AX-110488434	0.4184	10.3495
28	7D	AX-108929643	0.4184	10.3495
28	7D	AX-109384668	0.4184	10.3495
28	7D	AX-111965169	0.4184	10.3495
28	7D	AX-111658033	0.4184	10.3495
28	7D	AX-110274874	0.4184	10.3495
28	7D	AX-108784308	0.4184	10.3495
28	7D	AX-109385521	0.4184	10.3495
28	7D	AX-108759551	0.4184	10.3495
28	7D	AX-111532975	0.4184	10.3495
28	7D	AX-109501676	0.4184	10.3495

28	7D	AX-108973036	0.4184	10.3495
28	7D	AX-110908713	0.2092	10.5587
28	7D	AX-109956361	0.4202	10.9789
28	7D	AX-111438818	0.4184	11.3973
28	7D	AX-111671736	0.4184	11.3973
28	7D	AX-108934760	0.4184	11.3973
28	7D	AX-109943058	0.4202	11.8175
28	7D	AX-110993807	0.4202	11.8175
28	7D	AX-109407332	0.4202	11.8175
28	7D	AX-111039399	0.4202	11.8175
28	7D	AX-109280567	0.4202	11.8175
28	7D	AX-110978767	0.4202	11.8175
28	7D	AX-109856086	0.4202	11.8175
28	7D	AX-109399839	0.4202	11.8175
28	7D	AX-109316695	0.4202	11.8175
28	7D	AX-109917309	0.4202	11.8175
28	7D	AX-109408456	0.4202	11.8175
28	7D	AX-110714533	0.4202	11.8175
28	7D	AX-109730566	0.4202	11.8175
28	7D	AX-110364709	0.4202	11.8175
28	7D	AX-109966983	0.6303	12.4478
28	7D	AX-111607406	4.6646	17.1124
28	7D	AX-108759172	3.1717	20.2841
28	7D	AX-111629648	11.391	31.675
28	7D	AX-108881313	11.391	31.675
28	7D	AX-109196753	1.0778	32.7528
28	7D	AX-111467006	4.1379	36.8907
28	7D	AX-110002098	0.4274	37.318
28	7D	AX-109205685	4.8539	42.1719
28	7D	AX-110529080	0.8475	43.0195
28	7D	AX-111444364	0.8475	43.0195
28	7D	AX-111656163	22.8007	65.8201
28	7D	AX-111533232	0.4237	66.2439
28	7D	AX-89737284	1.2823	67.5262
28	7D	AX-108748734	31.9163	99.4425
28	7D	AX-111527690	7.948	107.3905
28	7D	AX-109740947	7.948	107.3905
28	7D	AX-89629840	0.2075	107.598
28	7D	AX-109188860	0.2075	107.598
28	7D	AX-109879725	0.2075	107.598
28	7D	AX-111682650	0.2075	107.598
28	7D	AX-111718685	0.2075	107.598
28	7D	AX-109894472	0.2075	107.598
28	7D	AX-110667549	2.8666	110.4646

28	7D	AX-89748459	0.2101	110.6747
28	7D	AX-111094029	0.6329	111.3076
28	7D	AX-111377844	0.6329	111.3076
28	7D	AX-110559985	0.6329	111.3076
28	7D	AX-109463522	0.6329	111.3076
28	7D	AX-110599032	0.6329	111.3076
28	7D	AX-109958755	0.2075	111.5151
28	7D	AX-110391604	0.2075	111.5151
28	7D	AX-109537193	0.2075	111.5151
28	7D	AX-108885806	0.2075	111.5151
28	7D	AX-109042871	0.4167	111.9317
28	7D	AX-110002663	0.4167	111.9317
28	7D	AX-109315691	0.4167	111.9317
28	7D	AX-108912002	0.4167	111.9317
28	7D	AX-110995197	0.4167	111.9317
28	7D	AX-109932219	0.4167	111.9317
28	7D	AX-110990504	0.4167	111.9317
28	7D	AX-111033480	0.2075	112.1392
28	7D	AX-89345397	0.2075	112.1392
28	7D	AX-110927321	0.2075	112.1392
28	7D	AX-108917929	0.2075	112.3467
28	7D	AX-110982010	0.2075	112.3467
28	7D	AX-109393113	0.2075	112.3467
28	7D	AX-109378018	0.2075	112.3467
28	7D	AX-109337466	0.2075	112.3467
28	7D	AX-109727691	0.2075	112.3467
28	7D	AX-110270636	0.2075	112.3467
28	7D	AX-109991178	0.2075	112.3467
28	7D	AX-108942812	0.2075	112.3467
28	7D	AX-108837773	0.2075	112.3467
28	7D	AX-109892956	0.6303	112.977
28	7D	AX-111570070	0.4202	113.3972
28	7D	AX-89713293	0.2101	113.6073
28	7D	AX-110611417	1.9661	115.5733
28	7D	AX-110021286	0.2101	115.7834
28	7D	AX-110441356	0.2101	115.7834
28	7D	AX-110930701	0.2101	115.7834
28	7D	AX-109369183	0.2101	115.7834
28	7D	AX-110769643	0.2101	115.7834
28	7D	AX-110910179	0.2101	115.7834
28	7D	AX-108892767	0.2101	115.7834
28	7D	AX-110906288	0.2101	115.7834
28	7D	AX-111121434	0.2101	115.7834
28	7D	AX-108738780	0.2101	115.7834

28	7D	AX-111011212	0.2101	115.7834
28	7D	AX-109404575	0.2101	115.7834
28	7D	AX-110544521	0.2101	115.7834
28	7D	AX-111456969	0.2101	115.7834
28	7D	AX-110447355	0.2101	115.7834
28	7D	AX-110173187	0.2101	115.7834
28	7D	AX-109940581	0.2101	115.7834
28	7D	AX-110791281	0.2101	115.7834
28	7D	AX-109895089	0.2101	115.7834
28	7D	AX-112288526	0.2101	115.7834
28	7D	AX-111607555	0.2101	115.7834
28	7D	AX-111079842	0.2101	115.7834
28	7D	AX-108839982	0.2101	115.7834
28	7D	AX-110245940	0.2101	115.7834
28	7D	AX-109309960	0.2101	115.7834
28	7D	AX-110036505	0.2101	115.7834
28	7D	AX-111542417	0.2101	115.7834
28	7D	AX-89681211	0.2101	115.7834
28	7D	AX-110556097	0.2101	115.7834
28	7D	AX-111508710	0.2101	115.7834
28	7D	AX-111855949	0.2101	115.7834
28	7D	AX-110172303	0.2101	115.7834
28	7D	AX-111687163	0.2101	115.7834
28	7D	AX-111511773	0.2101	115.7834
28	7D	AX-108766502	0.2101	115.7834
28	7D	AX-111143294	3.4617	119.2452
28	7D	AX-109418783	0.2155	119.4607
28	7D	AX-109891380	0.2155	119.4607
28	7D	AX-109548464	0.2155	119.4607
28	7D	AX-109728672	0.2155	119.4607
28	7D	AX-110359924	0.2155	119.4607
28	7D	AX-109074930	1.5026	120.9633
28	7D	AX-108817689	1.5026	120.9633
28	7D	AX-111361774	1.064	122.0273
28	7D	AX-108928265	1.2823	123.3096
28	7D	AX-110597684	1.2823	123.3096
28	7D	AX-108844280	1.2823	123.3096
28	7D	AX-108895252	1.2823	123.3096
28	7D	AX-89589386	0.2092	123.5188
28	7D	AX-109942885	0.4202	123.939
28	7D	AX-109627540	0.4202	123.939
28	7D	AX-111665954	0.6329	124.5719
28	7D	AX-109495093	1.0778	125.6497
28	7D	AX-110579501	0.6411	126.2908

28	7D	AX-111106049	0.4237	126.7145
28	7D	AX-110516489	0.8622	127.5766
28	7D	AX-111101483	1.3103	128.887
28	7D	AX-110281782	1.3103	128.887
28	7D	AX-110971794	0.2101	129.0971
28	7D	AX-111922613	0.2101	129.0971
28	7D	AX-110442381	0.2101	129.3072
28	7D	AX-109398922	0.2101	129.3072
28	7D	AX-110908746	0.4219	129.7291
28	7D	AX-111902534	0.8512	130.5803
28	7D	AX-111554668	0.8512	130.5803
28	7D	AX-110676782	0.8512	130.5803
28	7D	AX-109149684	0.8512	130.5803
28	7D	AX-109847755	0.8512	130.5803
28	7D	AX-111973087	0.8512	130.5803
28	7D	AX-110443275	0.8512	130.5803
28	7D	AX-109374178	0.2101	130.7903
28	7D	AX-108938253	0.2101	130.7903
28	7D	AX-111279859	0.2101	130.7903
28	7D	AX-109384830	1.3046	132.095
28	7D	AX-111460143	0.4311	132.526
28	7D	AX-109284163	0.2146	132.7406
28	7D	AX-111077878	1.0919	133.8325
28	7D	AX-110509545	0.4311	134.2635
28	7D	AX-111022995	1.9747	136.2383
28	7D	AX-110202444	0.2101	136.4483
28	7D	AX-110934330	0.2101	136.6584
28	7D	AX-110875183	0.2101	136.6584
28	7D	AX-111820217	1.0685	137.727
28	7D	AX-109723013	1.0685	137.727
28	7D	AX-110220756	0.2101	137.937
28	7D	AX-110585124	0.2101	137.937
28	7D	AX-109604940	0.422	138.359
28	7D	AX-109278070	0.6303	138.9893
28	7D	AX-108745974	0.6303	138.9893
28	7D	AX-110820533	0.6303	138.9893
28	7D	AX-111150898	0.6303	138.9893
28	7D	AX-111612050	0.6303	138.9893
28	7D	AX-111261489	0.6303	138.9893
28	7D	AX-109360054	0.6303	138.9893
28	7D	AX-111377746	0.6303	138.9893
28	7D	AX-110526200	0.6303	138.9893
28	7D	AX-111451592	0.6303	138.9893
28	7D	AX-109855278	0.6329	139.6222

28	7D	AX-111050077	0.6329	139.6222
28	7D	AX-89393548	0.8475	140.4698
28	7D	AX-109963034	0.2083	140.6781
28	7D	AX-111627051	0.2083	140.6781
28	7D	AX-108884138	0.2083	140.6781
28	7D	AX-109332089	0.2083	140.8864
28	7D	AX-109035303	0.2083	140.8864
28	7D	AX-110750491	0.2083	140.8864
28	7D	AX-110010796	0.2083	140.8864
28	7D	AX-111044062	0.2083	140.8864
28	7D	AX-109738363	0.2083	140.8864
28	7D	AX-109870865	0.2083	140.8864
28	7D	AX-111108835	0.2083	140.8864
28	7D	AX-109915283	0.2083	140.8864
28	7D	AX-111218083	0.2083	140.8864
28	7D	AX-111542234	0.2083	140.8864
28	7D	AX-110417203	0.2083	140.8864
28	7D	AX-111673313	0.2083	140.8864
28	7D	AX-109428601	0.2083	140.8864
28	7D	AX-108904641	0.2083	140.8864
28	7D	AX-111654079	0.2083	141.0948
28	7D	AX-109917900	0.4184	141.5132
28	7D	AX-111876137	0.2083	141.7215
28	7D	AX-111694371	0.2101	141.9316
28	7D	AX-111554046	0.2101	142.1417
28	7D	AX-110747494	0.2083	142.35
28	7D	AX-108838978	0.2083	142.35
28	7D	AX-109399434	0.2083	142.35
28	7D	AX-111014383	0.2083	142.5584
28	7D	AX-109113568	0.2083	142.5584
28	7D	AX-108814055	0.2083	142.5584
28	7D	AX-110251783	0.2083	142.5584
28	7D	AX-110766722	0.2083	142.5584
28	7D	AX-109680096	0.2083	142.5584
28	7D	AX-111839503	0.2083	142.5584
28	7D	AX-111034738	0.2083	142.5584
28	7D	AX-111348176	0.2083	142.5584
28	7D	AX-109416371	0.2083	142.7667
28	7D	AX-109865192	0.4202	143.1869
28	7D	AX-111576325	0.4202	143.1869
28	7D	AX-111003388	0.4202	143.1869
28	7D	AX-109342179	0.4202	143.1869
28	7D	AX-111607467	0.2092	143.3961
28	7D	AX-109862696	0.2092	143.3961

28	7D	AX-110254132	0.2092	143.3961
28	7D	AX-111038601	0.2092	143.3961
28	7D	AX-111038335	0.2092	143.3961
28	7D	AX-108834234	0.2092	143.3961
28	7D	AX-110021999	0.2092	143.3961
28	7D	AX-108747091	0.2092	143.3961
28	7D	AX-108956953	0.6303	144.0264
28	7D	AX-111481919	0.6303	144.0264
28	7D	AX-108962261	0.6303	144.0264
28	7D	AX-110400659	0.6303	144.0264
28	7D	AX-111594124	0.6303	144.0264
28	7D	AX-110975128	0.6303	144.0264
28	7D	AX-111518052	0.6303	144.0264
28	7D	AX-110391601	0.6303	144.0264
28	7D	AX-108823860	0.6303	144.0264
28	7D	AX-111203765	0.6303	144.0264
28	7D	AX-109955672	0.6303	144.0264
28	7D	AX-110397020	0.6303	144.0264
28	7D	AX-110509892	0.6303	144.0264
28	7D	AX-110829820	0.6303	144.0264
28	7D	AX-109963368	0.6303	144.0264
28	7D	AX-109935058	0.6303	144.0264
28	7D	AX-108902552	0.6303	144.0264
28	7D	AX-111034243	0.6303	144.0264
28	7D	AX-111361278	0.6303	144.0264
28	7D	AX-108934161	0.6303	144.0264
28	7D	AX-111601211	0.6303	144.0264
28	7D	AX-109521908	0.6303	144.0264
28	7D	AX-110146812	0.6303	144.0264
28	7D	AX-110666756	0.6303	144.0264
28	7D	AX-110026279	0.6303	144.0264
28	7D	AX-109390581	0.6303	144.0264
28	7D	AX-109698287	0.6303	144.0264
28	7D	AX-111026089	0.6303	144.0264
28	7D	AX-110287505	0.6303	144.0264
28	7D	AX-111415665	0.6303	144.0264
28	7D	AX-110287458	0.6303	144.0264
28	7D	AX-110276496	0.6303	144.0264
28	7D	AX-110514358	0.6303	144.0264
28	7D	AX-109580494	0.6303	144.0264
28	7D	AX-109162134	0.6303	144.0264
28	7D	AX-110928302	0.6303	144.0264
28	7D	AX-108803885	0.6303	144.0264
28	7D	AX-110784350	0.6303	144.0264

28	7D	AX-109374218	0.6303	144.0264
28	7D	AX-111219964	0.6303	144.0264
28	7D	AX-110257469	0.6303	144.0264
28	7D	AX-110212641	0.6303	144.0264
28	7D	AX-111609634	0.6303	144.0264
28	7D	AX-109658124	0.6303	144.0264
28	7D	AX-109694700	1.2823	145.3087
28	7D	AX-110278908	1.2823	145.3087
28	7D	AX-109623682	1.2823	145.3087
28	7D	AX-108934118	1.2823	145.3087
28	7D	AX-110901210	1.2823	145.3087
28	7D	AX-110631825	1.2823	145.3087
28	7D	AX-111887665	1.2823	145.3087
28	7D	AX-111052717	1.2823	145.3087
28	7D	AX-108759955	1.2823	145.3087
28	7D	AX-110220991	1.2823	145.3087
28	7D	AX-108938180	1.2823	145.3087
28	7D	AX-108753321	1.2823	145.3087
28	7D	AX-108775641	1.2823	145.3087
28	7D	AX-110993609	1.2823	145.3087
28	7D	AX-110830906	1.2823	145.3087
28	7D	AX-109354812	1.2823	145.3087
28	7D	AX-110489367	1.2823	145.3087
28	7D	AX-109583841	1.2823	145.3087
28	7D	AX-110823632	1.2823	145.3087
28	7D	AX-111063955	1.2823	145.3087
28	7D	AX-110527496	1.2823	145.3087
28	7D	AX-110841952	1.2823	145.3087
28	7D	AX-111374113	1.2823	145.3087
28	7D	AX-111264510	1.2823	145.3087
28	7D	AX-110481368	1.2823	145.3087
28	7D	AX-110243872	1.2823	145.3087
28	7D	AX-109333696	1.2823	145.3087
28	7D	AX-111558795	1.2823	145.3087
28	7D	AX-111537181	1.2823	145.3087
28	7D	AX-110097757	1.2823	145.3087
28	7D	AX-111030576	1.2823	145.3087
28	7D	AX-111126737	1.2823	145.3087
28	7D	AX-109924691	1.2823	145.3087
28	7D	AX-109288112	1.2823	145.3087
28	7D	AX-110790837	1.2823	145.3087
28	7D	AX-109905450	1.2823	145.3087
28	7D	AX-110356864	1.2823	145.3087
28	7D	AX-111140486	1.2823	145.3087

28	7D	AX-111310272	1.2823	145.3087
28	7D	AX-111092256	1.2823	145.3087
28	7D	AX-111402041	1.2823	145.3087
28	7D	AX-111532664	1.2823	145.3087
28	7D	AX-110066398	1.2823	145.3087
28	7D	AX-111647520	1.2823	145.3087
28	7D	AX-109910029	1.2823	145.3087
28	7D	AX-109837143	1.2823	145.3087
28	7D	AX-109430335	1.2823	145.3087
28	7D	AX-111762173	1.2823	145.3087
28	7D	AX-109580042	1.2823	145.3087
28	7D	AX-108934048	1.2823	145.3087
28	7D	AX-110415528	1.2823	145.3087
28	7D	AX-110938820	1.2823	145.3087
28	7D	AX-111322109	1.2823	145.3087
28	7D	AX-110245410	1.2823	145.3087
28	7D	AX-110772643	1.2823	145.3087
28	7D	AX-110928739	1.2823	145.3087
28	7D	AX-109464525	1.2823	145.3087
28	7D	AX-109457444	1.2823	145.3087
28	7D	AX-111550027	1.2823	145.3087
28	7D	AX-109278780	1.2823	145.3087
28	7D	AX-111083443	1.2823	145.3087
28	7D	AX-109321084	1.2823	145.3087
28	7D	AX-109778273	1.2823	145.3087
28	7D	AX-109496561	1.2823	145.3087
28	7D	AX-111588690	1.2823	145.3087
28	7D	AX-109852377	1.2823	145.3087
28	7D	AX-111094204	1.2823	145.3087
28	7D	AX-109192025	1.2823	145.3087
28	7D	AX-111065693	1.2823	145.3087
28	7D	AX-109777428	1.2823	145.3087
28	7D	AX-111408945	1.2823	145.3087
28	7D	AX-109791383	1.2823	145.3087
28	7D	AX-110388413	1.2823	145.3087
28	7D	AX-111140758	1.2823	145.3087
28	7D	AX-109336738	1.2823	145.3087
28	7D	AX-111011171	1.2823	145.3087
28	7D	AX-110334658	1.2823	145.3087
28	7D	AX-110541194	1.2823	145.3087
28	7D	AX-109483394	1.2823	145.3087
28	7D	AX-109190061	1.2823	145.3087
28	7D	AX-110457672	1.2823	145.3087
28	7D	AX-111296245	1.2823	145.3087

28	7D	AX-110754407	1.2823	145.3087
28	7D	AX-111876609	1.2823	145.3087
28	7D	AX-108910150	1.2823	145.3087
28	7D	AX-109508128	1.2823	145.3087
28	7D	AX-111756185	1.2823	145.3087
28	7D	AX-111247529	1.2823	145.3087
28	7D	AX-110829987	1.2823	145.3087
28	7D	AX-110240365	1.2823	145.3087
28	7D	AX-109279751	1.2823	145.3087
28	7D	AX-111023857	1.2823	145.3087
28	7D	AX-109885801	1.2823	145.3087
28	7D	AX-109988004	1.2823	145.3087
28	7D	AX-108739145	1.2823	145.3087
28	7D	AX-108802454	1.2823	145.3087
28	7D	AX-110792901	1.2823	145.3087
28	7D	AX-110483615	1.2823	145.3087
28	7D	AX-109822109	1.2823	145.3087
28	7D	AX-111100799	1.2823	145.3087
28	7D	AX-110539069	1.2823	145.3087
28	7D	AX-109379003	1.2823	145.3087
28	7D	AX-109619852	1.2823	145.3087
28	7D	AX-109640712	1.2823	145.3087
28	7D	AX-109851562	1.2823	145.3087
28	7D	AX-109741587	1.2823	145.3087
28	7D	AX-110397334	1.2823	145.3087
28	7D	AX-110779413	1.2823	145.3087
28	7D	AX-108976375	1.2823	145.3087
28	7D	AX-109398819	1.2823	145.3087
28	7D	AX-109178337	1.2823	145.3087
28	7D	AX-110962711	1.2823	145.3087
28	7D	AX-110824994	1.2823	145.3087
28	7D	AX-110501271	1.2823	145.3087
28	7D	AX-110503495	1.2823	145.3087
28	7D	AX-108738816	1.2823	145.3087
28	7D	AX-110258996	1.2823	145.3087
28	7D	AX-109132097	1.2823	145.3087
28	7D	AX-109715613	1.2823	145.3087
28	7D	AX-108959304	1.2823	145.3087
28	7D	AX-110285677	1.2823	145.3087
28	7D	AX-110804188	1.2823	145.3087
28	7D	AX-111839987	1.2823	145.3087
28	7D	AX-108822637	1.2823	145.3087
28	7D	AX-110245828	1.2823	145.3087
28	7D	AX-110059656	1.2823	145.3087

28	7D	AX-110078843	1.2823	145.3087
28	7D	AX-109368801	1.2823	145.3087
28	7D	AX-111048525	1.2823	145.3087
28	7D	AX-89764180	1.2823	145.3087
28	7D	AX-111485210	1.2823	145.3087
28	7D	AX-111649622	1.2823	145.3087
28	7D	AX-111157366	1.2823	145.3087
28	7D	AX-111546955	1.2823	145.3087
28	7D	AX-110513206	1.2823	145.3087
28	7D	AX-109496930	1.2823	145.3087
28	7D	AX-109272506	1.2823	145.3087
28	7D	AX-109380313	1.2823	145.3087
28	7D	AX-111507952	1.2823	145.3087
28	7D	AX-110774050	1.2823	145.3087
28	7D	AX-111368441	1.2823	145.3087
28	7D	AX-109946802	1.2823	145.3087
28	7D	AX-111044186	1.2823	145.3087
28	7D	AX-89385155	1.2823	145.3087
28	7D	AX-110474669	1.2823	145.3087
28	7D	AX-110767188	1.2823	145.3087
28	7D	AX-111847061	1.2823	145.3087
28	7D	AX-110045927	1.2823	145.3087
28	7D	AX-109161168	1.2823	145.3087
28	7D	AX-108873054	0.2092	145.5179
28	7D	AX-109318566	0.4202	145.9381
28	7D	AX-110082013	0.4184	146.3565
28	7D	AX-109967642	0.4184	146.3565
28	7D	AX-110923511	0.4184	146.3565
28	7D	AX-110200543	0.4184	146.3565
28	7D	AX-109660972	0.4184	146.3565
28	7D	AX-110460471	0.4184	146.3565
28	7D	AX-110610247	0.4184	146.3565
28	7D	AX-110125073	0.4184	146.3565
28	7D	AX-109576809	0.4184	146.3565
28	7D	AX-109493621	0.4184	146.3565
28	7D	AX-111114314	0.4184	146.3565
28	7D	AX-111032450	0.4184	146.3565
28	7D	AX-111542493	0.4184	146.3565
28	7D	AX-110667060	0.2083	146.5648
28	7D	AX-110946735	0.2083	146.5648
28	7D	AX-110450402	0.2083	146.5648
28	7D	AX-111454962	0.2083	146.5648
28	7D	AX-111049975	0.2083	146.5648
28	7D	AX-111780189	0.2083	146.5648

28	7D	AX-110525188	0.2083	146.5648
28	7D	AX-110030789	0.2101	146.7749
28	7D	AX-109178709	0.4219	147.1969
28	7D	AX-109284685	0.4219	147.1969
28	7D	AX-111903276	0.2146	147.4115
28	7D	AX-109324664	0.2155	147.627
28	7D	AX-110475145	0.2155	147.627
28	7D	AX-109194960	0.2155	147.627
28	7D	AX-111729400	0.2155	147.627
28	7D	AX-111645035	0.2155	147.627
28	7D	AX-111838399	0.2155	147.627
28	7D	AX-109657199	0.2155	147.627
28	7D	AX-110365047	0.2155	147.627
28	7D	AX-108816229	0.2155	147.627
28	7D	AX-111761327	0.2155	147.627
28	7D	AX-110336558	0.2155	147.627
28	7D	AX-111088204	0.2155	147.627
28	7D	AX-109030892	0.2155	147.627
28	7D	AX-110777349	0.2101	147.8371
28	7D	AX-111544235	0.2101	147.8371
28	7D	AX-110922121	0.422	148.259
28	7D	AX-110913995	0.422	148.259
28	7D	AX-110518345	0.422	148.259
28	7D	AX-109195214	0.4219	148.681
28	7D	AX-111924296	0.4219	148.681
28	7D	AX-109331077	0.4219	148.681
28	7D	AX-110222386	0.4219	148.681
28	7D	AX-111289968	0.4219	148.681
28	7D	AX-111363394	0.4219	148.681
28	7D	AX-111562975	0.4219	148.681
28	7D	AX-110815218	0.4219	148.681
28	7D	AX-109178084	0.2083	148.8893
28	7D	AX-110967309	0.2083	148.8893
28	7D	AX-109936012	0.2083	148.8893
28	7D	AX-108921665	0.2083	148.8893
28	7D	AX-111538655	0.2083	148.8893
28	7D	AX-110021566	0.2083	148.8893
28	7D	AX-110488274	0.2083	148.8893
28	7D	AX-109951714	0.2083	148.8893
28	7D	AX-109548259	0.2083	148.8893
28	7D	AX-110907703	0.2155	149.1048
28	7D	AX-110967909	0.4348	149.5396
28	7D	AX-109038840	0.4348	149.5396
28	7D	AX-111552105	0.4202	149.9598

28	7D	AX-108909298	0.2101	150.1699
28	7D	AX-111110452	0.2101	150.1699
28	7D	AX-111580200	0.2101	150.1699
28	7D	AX-110329072	0.2101	150.1699
28	7D	AX-109451619	0.2101	150.1699
28	7D	AX-109160430	0.2101	150.1699
28	7D	AX-109791875	0.211	150.3809
28	7D	AX-109112266	0.211	150.3809
28	7D	AX-110271452	0.8512	151.232
28	7D	AX-108798483	0.8512	151.232
28	7D	AX-109695722	0.8512	152.0832
28	7D	AX-110458085	0.8512	152.0832
28	7D	AX-111144869	0.8512	152.0832
28	7D	AX-109931701	0.2101	152.2932
28	7D	AX-110961272	0.2101	152.2932
28	7D	AX-111563832	0.2101	152.2932
28	7D	AX-109237711	0.2101	152.2932
28	7D	AX-111288880	0.2101	152.2932
28	7D	AX-109903353	0.2101	152.2932
28	7D	AX-109293148	0.2101	152.2932
28	7D	AX-110788687	0.2083	152.5016
28	7D	AX-108824683	0.2083	152.5016
28	7D	AX-108859395	0.2083	152.5016
28	7D	AX-111856876	0.2083	152.5016
28	7D	AX-110033734	0.2083	152.5016
28	7D	AX-111879582	0.2083	152.5016
28	7D	AX-111161034	0.2083	152.5016
28	7D	AX-109448523	0.2083	152.5016
28	7D	AX-111904404	0.2083	152.5016
28	7D	AX-108881008	0.2083	152.5016
28	7D	AX-109883207	0.2083	152.5016
28	7D	AX-111913853	0.2083	152.5016
28	7D	AX-109597560	0.2083	152.5016
28	7D	AX-111019040	0.2083	152.5016
28	7D	AX-111054723	0.2083	152.5016
28	7D	AX-111039914	0.2083	152.5016
28	7D	AX-109270581	0.2083	152.5016
28	7D	AX-111530891	0.2083	152.5016
28	7D	AX-110565645	0.2083	152.5016
28	7D	AX-110993532	0.2083	152.5016
28	7D	AX-108807696	0.2083	152.5016
28	7D	AX-110330405	0.2083	152.5016
28	7D	AX-110826342	0.2083	152.5016
28	7D	AX-110240876	0.2083	152.5016

28	7D	AX-111962004	0.2092	152.7108
28	7D	AX-111054838	0.2119	152.9227
28	7D	AX-110898812	0.211	153.1336
28	7D	AX-108904493	0.2092	153.3428
28	7D	AX-111327877	0.2092	153.3428
28	7D	AX-110272287	0.2092	153.3428
28	7D	AX-110223836	0.2092	153.3428
28	7D	AX-110712708	0.2092	153.3428
28	7D	AX-109247018	0.2092	153.3428
28	7D	AX-111101678	0.2092	153.3428
28	7D	AX-110785523	0.2092	153.3428
28	7D	AX-111527599	0.6329	153.9758
28	7D	AX-109199910	1.7323	155.7081
28	7D	AX-109551456	0.4255	156.1336
28	7D	AX-111559194	0.2101	156.3437
28	7D	AX-110603982	0.2101	156.3437
28	7D	AX-109276388	0.2101	156.3437
28	7D	AX-109783270	0.2101	156.3437
28	7D	AX-111310855	0.2101	156.3437
28	7D	AX-110281542	0.2101	156.3437
28	7D	AX-111217774	0.6303	156.974
28	7D	AX-109292798	0.6303	156.974
28	7D	AX-109953422	0.6303	156.974
28	7D	AX-109488029	0.6303	156.974
28	7D	AX-110912379	0.6303	156.974
28	7D	AX-108923671	0.2083	157.1823
28	7D	AX-111292556	0.2083	157.1823
28	7D	AX-110570975	0.2083	157.1823
28	7D	AX-109713409	0.2083	157.1823
28	7D	AX-109302344	0.2083	157.1823
28	7D	AX-111693228	0.2083	157.1823
28	7D	AX-108795515	0.4202	157.6025
28	7D	AX-110910978	0.2092	157.8117
28	7D	AX-110288612	0.2092	157.8117
28	7D	AX-109861779	0.2092	157.8117
28	7D	AX-109346970	0.2092	157.8117
28	7D	AX-108739865	0.2083	158.02
28	7D	AX-110296941	0.2083	158.02
28	7D	AX-108899993	0.2083	158.2284
28	7D	AX-110411683	0.2083	158.2284
28	7D	AX-108912162	0.2092	158.4376
28	7D	AX-111577597	4.1379	162.5754
28	7D	AX-110121123	4.1379	162.5754
28	7D	AX-109541707	2.0011	164.5765

28	7D	AX-109095044	0.8548	165.4313
28	7D	AX-111187784	0.8548	165.4313
28	7D	AX-110376559	0.8548	165.4313
28	7D	AX-109853847	0.8548	165.4313
28	7D	AX-111798949	0.8548	165.4313
28	7D	AX-111215243	0.8548	165.4313
28	7D	AX-110059327	2.2138	167.6451
28	7D	AX-108758043	2.2138	167.6451
28	7D	AX-110949705	2.2138	167.6451
28	7D	AX-89501112	0.4255	168.0707
28	7D	AX-89327031	0.211	168.2816
28	7D	AX-109742925	0.211	168.2816
28	7D	AX-111720315	0.211	168.2816
28	7D	AX-109240909	0.211	168.2816
28	7D	AX-111759227	0.211	168.2816
28	7D	AX-110387867	0.211	168.2816
28	7D	AX-110977636	0.211	168.2816
28	7D	AX-109908449	0.211	168.2816
28	7D	AX-110914509	0.211	168.2816
28	7D	AX-111802901	0.211	168.2816
28	7D	AX-110410208	0.8548	169.1364
28	7D	AX-109367830	0.211	169.3474
28	7D	AX-110084479	0.211	169.3474
28	7D	AX-94442995	0.211	169.3474
28	7D	AX-111797359	0.211	169.3474
28	7D	AX-111640147	0.211	169.5584
28	7D	AX-111684467	0.2119	169.7702
28	7D	AX-94413894	0.4274	170.1976
28	7D	AX-108766636	0.4274	170.1976
28	7D	AX-94696367	0.4274	170.1976
28	7D	AX-109818067	0.4274	170.1976
28	7D	AX-111655013	0.4274	170.1976
28	7D	AX-109269910	0.4274	170.1976
28	7D	AX-109593075	0.4274	170.1976
28	7D	AX-110982634	0.4274	170.1976
28	7D	AX-111691248	0.4274	170.1976
28	7D	AX-110310035	0.4274	170.1976
28	7D	AX-110376367	0.4274	170.1976
28	7D	AX-89394280	0.4274	170.1976
28	7D	AX-109353227	0.4274	170.1976
28	7D	AX-109956441	0.4274	170.1976
28	7D	AX-110467916	0.4274	170.1976
28	7D	AX-109500341	0.4274	170.1976
28	7D	AX-110811078	0.4274	170.1976

28	7D	AX-109796194	0.4274	170.1976
28	7D	AX-109468853	0.4274	170.1976
28	7D	AX-111011992	0.4274	170.1976
28	7D	AX-110837979	0.4274	170.1976
28	7D	AX-110540631	0.4274	170.1976
28	7D	AX-108937678	0.4292	170.6268
28	7D	AX-111175958	0.2137	170.8405
28	7D	AX-110038618	0.6383	171.4788
28	7D	AX-111255446	0.2101	171.6889
28	7D	AX-109515037	0.2101	171.6889
28	7D	AX-111151367	0.2101	171.6889
28	7D	AX-111438923	0.2101	171.6889
28	7D	AX-110993909	0.2101	171.6889
28	7D	AX-89384255	0.2101	171.6889
28	7D	AX-86179932	0.2101	171.6889
28	7D	AX-110010894	0.2101	171.6889
28	7D	AX-109744422	0.2101	171.6889
28	7D	AX-108727993	0.2101	171.6889
28	7D	AX-111565535	0.2101	171.6889
28	7D	AX-109631256	0.2101	171.6889
28	7D	AX-109868564	0.8585	172.5473
28	7D	AX-110497122	0.8585	172.5473
28	7D	AX-109504962	4.9456	177.493
28	7D	AX-111687821	0.211	177.7039
28	7D	AX-109390503	0.211	177.7039
28	7D	AX-110473775	0.2101	177.914
28	7D	AX-108920521	0.2101	177.914
28	7D	AX-109353712	0.2146	178.1286
28	7D	AX-110338241	0.6551	178.7837
28	7D	AX-109859092	2.9715	181.7552
28	7D	AX-109138262	2.9715	181.7552
28	7D	AX-109287932	2.9715	181.7552
28	7D	AX-110498045	2.9715	181.7552
28	7D	AX-89389306	2.9715	181.7552
28	7D	AX-108763519	0.4274	182.1826
28	7D	AX-110456494	0.4274	182.1826
28	7D	AX-109312905	9.4545	191.6371
28	7D	AX-111022287	11.6655	203.3026
28	7D	AX-111077348	2.8921	206.1947
28	7D	AX-89724749	0.431	206.6257
28	7D	AX-110425263	1.3578	207.9836
28	7D	AX-89691195	7.6345	215.6181
28	7D	AX-111728699	12.6274	228.2455
28	7D	AX-111800731	12.6274	228.2455

28	7D	AX-109905599	12.6274	228.2455
28	7D	AX-111133653	12.6274	228.2455
28	7D	AX-111730171	12.6274	228.2455
28	7D	AX-110967901	12.6274	228.2455
28	7D	AX-111363680	12.6274	228.2455
28	7D	AX-109841391	12.6274	228.2455
28	7D	AX-111593353	12.6274	228.2455
28	7D	AX-109462741	12.6274	228.2455
28	7D	AX-111640950	12.6274	228.2455
28	7D	AX-108981818	12.6274	228.2455
28	7D	AX-110681402	12.6274	228.2455
28	7D	AX-109303654	12.6274	228.2455
28	7D	AX-111729420	12.6274	228.2455
28	7D	AX-110373638	12.6274	228.2455
28	7D	AX-109334882	12.6274	228.2455
28	7D	AX-110474717	12.6274	228.2455
28	7D	AX-111141489	12.6274	228.2455
28	7D	AX-109363666	12.6274	228.2455
28	7D	AX-108914131	12.6274	228.2455
28	7D	AX-109983337	12.6274	228.2455
28	7D	AX-111926760	12.6274	228.2455
28	7D	AX-109012926	12.6274	228.2455
28	7D	AX-109372955	12.6274	228.2455
28	7D	AX-109399833	12.6274	228.2455
28	7D	AX-108737012	0.431	228.6765
28	7D	AX-108814669	2.2437	230.9202
28	7D	AX-110973196	2.2437	230.9202
28	7D	AX-109872474	12.4874	243.4075
28	7D	AX-109487073	3.4144	246.8219
28	7D	AX-108779800	0.2137	247.0356
28	7D	AX-110039359	5.4202	252.4558

Table S2 Phenotypic variation for SL and KNS in the RIL population

Trait	Environment	BainongAK58	Bima4	RIL			
				Min	Max	Mean	Stdev
SL	2017XI	8.01	8.38	5.44	12.74	8.58	1.32
	2018XI	9.06	8.18	5.74	14.74	9.16	1.58
	2018HU*	8.02	8.34	4.98	11.88	8.49	1.28
	2019XI	7.88	8.20	5.70	13.37	8.65	1.26
	Average	8.24	8.28	5.55	13.06	8.73	1.31
KNS	2017XI**	39.67	53.1	26.50	61.60	41.32	6.85
	2018XI	46.83	45.83	24.83	62.67	42.31	6.91
	2018HU**	55.17	44.67	31.83	61.00	45.00	6.02
	2019XI**	41.33	52.83	26.17	60.83	43.60	6.40

Average	45.75		49.11	30.11	57.67	43.06	5.45

SD, standard deviation; *and **, significant at the 0.05 and 0.01 level, respectively

Table S3 Seventy wheat cultivars derived from Bima4

Generation	Number	Cultivars
1st	12	Beijing8, Shijiazhuang54, Zhengzhou24, Dexuan1, Jinan2, Jinan4, Jinan8, Zhengzhou15, Shaannong17, 54405, Jinan5, Qingchun2
2nd	35	Xifeng9, Beijing14, Jingzuo208, Jingzuo210, Jingzuo236, Jingzuo278, Nongda198, Dai179, Kedong81, Kedong83, Kechun14, Youmangbai2, Youmangbai4, Youmanghong7, Youmanghong8, Youmanghong18, Xiangyang4, Shipin83, Xingxuan7, Weidong8, Jinan10, Jinan12, Taishan1, Changwei18, Jining3, Luzhan1, Beijing11, Beijing12, Beijing13, Jimai23, Weidong7, Beijing16, Qingxuan15, Youmangbai15, Jinan14
3rd	18	Fengkang1, Fengkang2, Fengkang4, Fengkang5, Fengkang7, Fengkang8, Fengkang11, Fengkang9, Fengkang10, Fengkang15, Jingshuang16, Jingdong1, Linfen5064, Lumai2, Shi4414, Jinghua1, Linfen10, Jingwang9
4th	5	Jing411, Jing437, Beijing841, Beijing8694, Jimai30
